# Rational design, optimization, and biological evaluation of novel α-Phosphonopropionic acids as covalent inhibitors of Rab geranylgeranyl transferase

**DOI:** 10.1080/14756366.2022.2053525

**Published:** 2022-03-30

**Authors:** Joanna Małolepsza, Aleksandra Marchwicka, Remigiusz A. Serwa, Sanna P. Niinivehmas, Olli T. Pentikäinen, Edyta Gendaszewska-Darmach, Katarzyna M. Błażewska

**Affiliations:** a Institute of Organic Chemistry, Faculty of Chemistry, Lodz University of Technology, Łódź, Poland; b Institute of Molecular and Industrial Biotechnology, Faculty of Biotechnology and Food Sciences, Lodz University of Technology, Łódź, Poland; c ReMedy International Research Agenda Unit, IMol Polish Academy of Sciences, Warsaw, Poland; d Institute of Biomedicine, University of Turku, Turku, Finland; e InFLAMES Research Flagship Center, University of Turku, Turku, Finland

**Keywords:** Phosphonocarboxylate, Rab geranylgeranyltransferase, prenylation, imidazo[1, 2-a]pyridine, covalent inhibitors

## Abstract

Rab geranylgeranyltransferase (GGTase-II, RGGT) catalyses the post-translational modification of eukaryotic Rab GTPases, proteins implicated in several pathologies, including cancer, diabetes, neurodegenerative, and infectious diseases. Thus, RGGT inhibitors are believed to be a potential platform for the development of drugs and tools for studying processes related to the abnormal activity of Rab GTPases. Here, a series of new α-phosphonocarboxylates have been prepared in the first attempt of rational design of covalent inhibitors of RGGT derived from non-covalent inhibitors. These compounds were equipped with electrophilic groups capable of binding cysteines, which are present in the catalytic cavity of RGGT. A few of these analogues have shown micromolar activity against RGGT, which correlated with their ability to inhibit the proliferation of the HeLa cancer cell line. The proposed mechanism of this inhibitory activity was rationalised by molecular docking and mass spectrometric measurements, supported by stability and reactivity studies.

## Graphical Abstract

**Figure:**
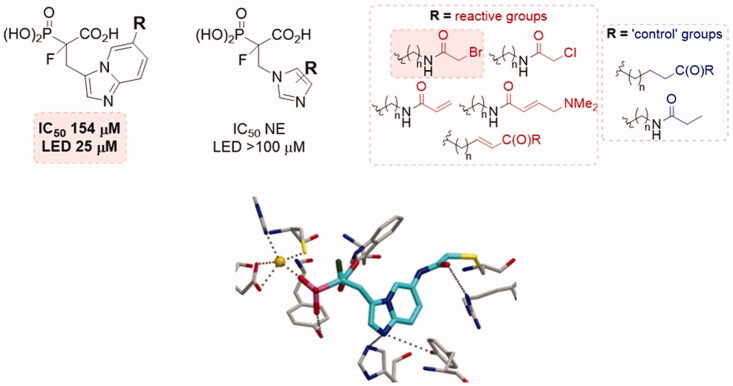

## 1. Introduction

Over the past two decades, targeted covalent inhibitors have gained recognition as an effective approach in drug discovery.^1^^,^^2^ Such trend results from the increased awareness of the benefits that this approach provides (e.g. improved efficacy and beneficial pharmacokinetics, resulting in a longer duration of action), as well as technological development, especially in the field of metabolomics and proteomics.^3^ The latter has enabled credible determination of mechanisms of action of new compounds, their effectiveness, and identification of potential side effects. Recent years have brought to the market new therapeutics with the covalent mechanism of action, e.g. carfilzomib (multiple myeloma), abiraterone (prostate cancer), and afatinib (lung cancer).^1^^,^^3^ The very last reports show the efficacy of covalent cysteine binding inhibitor paxlovid as the potential drug for COVID-19.^4^ This strategy is also applicable in the design of molecular probes for studying the function of biomolecules and biochemical processes.^5–7^

Encouraged by the possible benefits of this approach, we designed first covalent inhibitors of Rab geranylgeranyltransferase, derived from α-phosphonocarboxylates (PCs). RGGT is involved in the post-translational modification of eukaryotic Rab GTPases, the primary regulators of the formation, transport, docking, and fusion of vesicles during membrane transport.^8^ The dysfunction of Rab GTPases leads to various diseases ranging from infections to cancer.^9^ RGGT catalyses double prenylation of most Rab GTPases, with the formation of a thioether bond between two C-terminal cysteines and isoprenoid chains derived from geranylgeranyl pyrophosphate. This modification is necessary for the proper functioning of Rab GTPases, making RGGT a potential drug target. RGGT is a heterodimer composed of an α subunit encoded by the *RABGGTA* gene and a β subunit encoded by the *RABGGTB* gene.^10^

**Figure 1.**
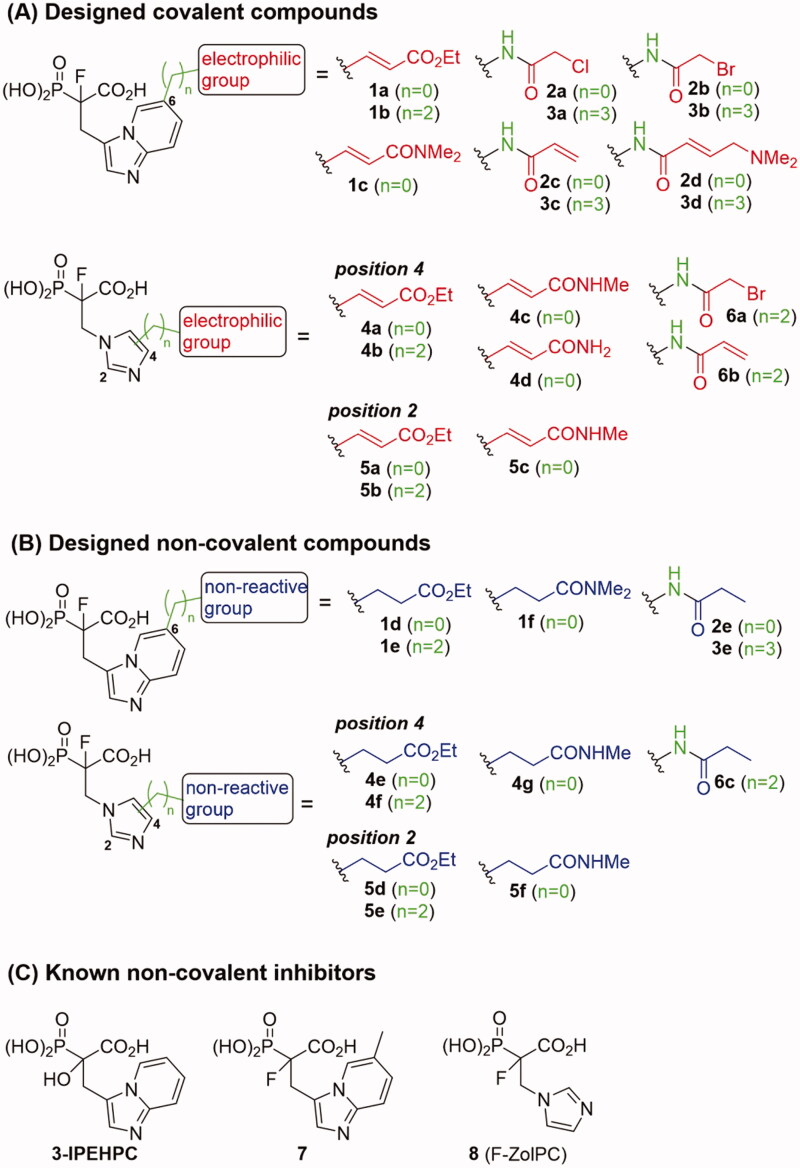
Structures of the studied compounds: (A) potential inhibitors with the covalent mode of action and (B) the non-covalent control analogues, derived from analogues with the non-covalent mechanism of action (C).

Five classes of RGGT inhibitors have been reported to date.^11–15^ Among them, tetrahydrobenzodiazepine derivatives show the highest inhibition efficiency,^13^ while the natural product, psoromic acid, is the only covalent inhibitor of RGGT.^14^ α-Phosphonocarboxylates inhibit the introduction of the second geranylgeranyl group to Rabs, making them more selective, by affecting only proteins that require double prenylation.^15^ Inhibition of Rab prenylation by PCs is also correlated with their cytotoxic properties.^16^^,^^17^ We demonstrated that the most active derivatives of currently known phosphonocarboxylates contain imidazo[1,2-*a*]pyridine^17^ or the imidazole ring^18^ (Figure 1). As no structural data describing phosphonocarboxylate – RGGT complexes exist, we proposed the model for their interaction based on the molecular docking results for imidazo[1,2-*a*]pyridine analogues.^17^ Importantly, a few cysteines are present near the proposed site of interaction that could be targeted by the electrophilic moiety of the covalent inhibitor. Developing a covalent inhibitor could increase the effectiveness of inhibition and confirm the place of interaction with the studied enzyme.

Herein we describe the first reported attempt to develop covalent inhibitors of RGGT. The novel compounds were derived from two PC inhibitors of RGGT: compound **7**, the 6-substituted analogue of 3–(3-pyridyl)-2-hydroxy-2-phosphonopropanoic acid (3-IPEHPC) and compound **8**, 2-fluoro-3-(1H-imidazol-1-yl)-2-phosphonopropanoic acid (F-ZolPC) (Figure 1C).^17^^,^^19^ The modifications vary in linker length, size, geometry, and reactivity of substituents, aiming at thiol residue of cysteine ​​located in the catalytic cavity of the studied enzyme (Figure 1). As electrophilic traps, we chose moieties present in the known drugs with a covalent mechanism of action, targeting cysteines.^6^^,^^20^ Most synthesised compounds were Michael acceptors but we have also studied chloro- and bromoacetamide fragments (Figure 1A). We also synthesised a group of negative controls, in which the electrophilic group was replaced by structurally similar but non-reactive moiety (Figure 1B).

Compounds **1–6** were examined for their potency against RGGT, using a procedure previously developed in our group.^17^ We also tested their selectivity by assessing the prenylation of Rap1A/Rap1B, a substrate of a structurally similar prenyltransferase, GGTase-I. The results were supported by covalent docking studies and the LC-MS-based peptide mapping.

## 2. Materials and methods

### 2.1. Chemistry experimental procedures

All reagents were purchased from commercial sources and used as obtained unless specified otherwise. Compounds **9–29** were purified using Gilson PLC 2250 purification system coupled with ‘The Advion expression compact mass spectrometer’ (mode of ionisation: APCI). Appropriate products’ fractions were identified based on the value of peak [M + H^+^]. Preparative HPLC for purification of compounds **1–6** was performed using Gilson Prep equipped with a UV − vis-156 detector and semipreparative column Kromasil 100 − 5-C18 (5 µm, 10 mm × 250 mm). NMR spectra were measured at 250.13 or 700 MHz for ^1^H NMR, 62.90 or 170 MHz for ^13 ^C NMR, 283 or 101.30 MHz for ^31 ^P NMR on Bruker Avance DPX 250 and Bruker Avance II Plus 700 spectrometers, respectively. Chemical shifts (δ) are reported in parts per million (ppm) relative to internal residual CHCl_3_ in CDCl^3^ (δ 7.26 ^1^H NMR) or CDCl_3_ signal in ^13 ^C NMR (δ 77.16); internal residual HDO in D_2_O (δ 4.79 ^1^H NMR) or external 85% H_3_PO_4_ (δ 0 ppm ^31 ^P NMR). ^31 ^P NMR and ^13 ^C NMR spectra were proton-decoupled. Coupling constants (*J*) are quoted in Hz. The assignment of the signals in ^1^H NMR and ^13 ^C NMR was supported by two-dimensional experiments (COSY, HMQC, HMBC). Several signals of quaternary ^13 ^C were only observed in the HMBC spectrum (signals from PCF or FCCO_2_H); therefore their chemical shifts (δ) are reported with an accuracy of 1 ppm. All compounds showing activity against RGGT are >95% pure by elemental analysis. A monomode microwave reactor (CEM Discover SP) equipped with an IntelliVent pressure control system was used. The standard method was applied, and maximum pressure was set to 250 psi. Temperatures of the reaction mixtures were measured with an external infra-red sensor. All synthetic procedures are included in Supplemental Material.

### 2.2. Stability studies

Compound **1–3** (about 1–1.5 mg, final concentration 5 mM) was dissolved in 10 mM PBS (PBS prepared by dissolving Gibco® PBS tablets in D_2_O according to the manufacturer's instructions) in the NMR tube (diameter 5 mm). Next, the sample’s pH was adjusted to 7 using 3–6 µL of 1 M NaOH/D_2_O. The sample was measured by ^1^H NMR immediately after its preparation and after 24, 48, and 72 h of incubation at 37° C. All compounds (except for analogues **2b** and **3b**) proved to be stable under the applied conditions. After 72 h, only about 35% of **2b** was left untouched, while **3b** completely decomposed. Numerous unidentified products of decomposition were observed in such reaction mixture. The molar ratio of starting compounds **2b**, **3b** compared with new ones, was estimated based on the integration of signals in the ^1^H NMR spectrum derived from -CH_2_Br protons and the summed up integrations of aromatic ring protons from compound **2b**/**3b** and its products of decomposition.

#### 2.2.1. Reactivity studies

Compounds **1–3** (about 1–1.5 mg), 100 mM PBS (500 µL, prepared by dissolving Gibco^®^ PBS tablets in D_2_O) and DTPA (diethylenetriaminepentaacetic acid, 10 µL of 10 mM solution in D_2_O) were placed in the NMR tube. As an internal standard dimethylformamide (DMF, 3 µL) was used and added to the sample [δ 8.2 ppm (s, HCONMe_2_, 1H)]. The prepared sample was mixed thoroughly by shaking and degassed by placing in an ultrasonic bath. To avoid the oxidation of glutathione, the tube was purged with argon. Next, the sample was measured by ^1^H NMR allowing observation of the integration ratio of signals of compound **1–3** and internal standard at t = 0 min. Then, GSH (100 µL) was added and mixed thoroughly, degassed and purged with argon. The final concentration of the test compound was 5 mM and glutathione was 40 mM (8 x concentration of the test compound). Next, the ^1^H NMR spectrum was recorded after 10–20 min at 37 °C and then every 1 h for 11–16 h. For the duration of the reaction, the sample was incubated at 37 °C. The assessment of the progress of the reaction was made based on the decreasing integration of signals from the electrophilic group of the test compounds or based on the increasing integration of signals from the reaction product with glutathione. The effect of the oxidation of GSH on its effective concentration during the reaction was negligible (approx. 5% of oxidised GSH after 24 h of incubation at 37 °C) and does not affect the speed of the reaction with the tested compounds.

### 2.3. Biological materials and methods

#### 2.3.1. General

PrestoBlue^®^ Cell Viability Reagent, Mem-PER™ Plus Membrane Protein Extraction Kit, and all reagents for cell culture were purchased from Life Technologies (Carlsbad, CA, USA). Bradford Protein Assay and Clarity™ Western ECL Substrate were obtained from Bio*-*Rad *(*Hercules, CA, USA). Protease inhibitor cocktail and lovastatin were purchased from Sigma (Saint Louis, MO, USA). Primary antibodies against Rab11A and Rap1A/Rap1B were obtained from Abcam (Cambridge, UK) and primary antibodies against β-actin along with secondary HRP-linked antibodies were purchased from Cell Signalling Technology (Beverly, MA, USA).

Iodoacetamide and WaLP were purchased from Sigma (Saint Louis, MO, USA), DTT, CuSO_4_, TCEP from Fluorochem (Derbyshire, United Kingdom) and THPTA, biotin-PEG_3_-azide from Click Chemistry Tools (Scottsdale, AZ, USA). Sequencing grade chymotrypsin was purchased from Promega (Madison, WI, USA).

#### 2.3.2. HeLa cell culture

The cervical epithelial carcinoma HeLa cell line was purchased from the American Type Cell Collection (ATCC). Cells were cultured in Dulbecco’s modified Eagle’s medium (DMEM) supplemented with 10% foetal bovine serum (FBS) containing 100 IU/mL penicillin, 0.25 µg/mL amphotericin B and 50 µg/mL neomycin. Cells were incubated at 37 °C in a humidified atmosphere of 95% air and 5% CO_2_. For biological studies, compounds were dissolved just before use in PBS at a stock concentration of 10 mM and then pH adjusted to about 7.

#### 2.3.3. Determination of cytotoxicity

HeLa cells were seeded into 96-well cell culture plates at a density of 4 × 10^3^ cells/well in 100 µL of complete, serum-containing culture medium. On the following day, cells were washed with phosphate-buffered saline (PBS) and 100 µL of serum-free (fasting) medium was added as described previously.^17^ Subsequently, HeLa cells were treated with PCs at eight concentrations and 72 h later PrestoBlue^®^ Cell Viability Reagent was applied. Following 50 min incubation time at 37 °C and 5% CO_2_, cell viability was determined by measuring the fluorescent signal (Ex/Em = 530/590 nm) on a Synergy 2 Microplate Reader (BioTek, Vermont, USA). The obtained fluorescence magnitudes were used to calculate cell viability expressed as a percent of the viability of the untreated control cells. The data expressed as the mean of at least 3 independent experiments were used to calculate the IC_50_ parameter.

#### 2.3.4. Assessment of inhibition of Rab11A and Rap1A/Rap1B prenylation

HeLa cells were seeded into 6-well cell culture plates at a density of 4 × 10^5^ cells/well in 3 ml of complete medium. On the following day, 1.5 ml of fresh serum-free medium was supplemented with PCs or lovastatin. The latter was used as a control acting as a hydroxymethylglutaryl (HMG)-coenzyme A (CoA) reductase inhibitor and preventing the downstream biosynthesis of cholesterol and prenylation with the geranylgeranyl or farnesyl moieties.^21^ After 48 h of incubation, cell monolayers were rinsed with PBS and detached using trypsin-EDTA solution. The cytosolic and membrane-rich fractions, containing protease inhibitor cocktail, were isolated from cell pellets using Mem-PER™ Plus Membrane Protein Extraction Kit according to the manufacturers’ instructions. The protein concentration in both fractions was determined using Bradford Protein Assay. Equal amounts of protein (30 µg) from cytosolic fractions were resolved by 12% SDS-PAGE gels and transferred to 0.2 µm nitrocellulose membrane. Membranes were probed with β-actin, Rab11A, or Rap1A/Rap1B antibodies and detected using the appropriate HRP-conjugated secondary antibody, followed by an ECL assay. Visualisation of the chemiluminescent protein bands was performed using ChemiDoc™ MP Imaging System (Bio-Rad). Densitometry analysis was performed with ImageLab™ Software (Bio-Rad) and relative unprenylated protein band intensity was normalised to β-actin and quantified with respect to controls (untreated cells).

### 2.4. Statistical analysis

Unless stated otherwise, all the biological results are presented as means of 3–6 repeated experiments ± SEM. Statistical differences between mean values of the inhibitor treated and untreated samples were analysed using one-way ANOVA followed by Dunnett’s multiple comparisons test using GraphPad Prism (version 6.01 for Windows, GraphPad Software, La Jolla California USA, www.graphpad.com). Confidence *p*-levels are indicated by asterisks, with * denoting *p* ≤ 0.05, ** denoting *p* ≤ 0.01, *** denoting *p* ≤ 0.001, and **** denoting *p* ≤ 0.0001.

### 2.5. Molecular docking

Compounds were drawn with Maestro 2019.2 (Schrödinger release 2019–2: Maestro, Schrödinger, LLC, New York, NY, 2019). Both stereoisomers were drawn for all compounds selected for molecular docking (covalent compounds **2a**, **2b**, **2c,** and **2d**, and reference compounds **1d**, **1e**, and **2e**; Table 2). Protein structure for docking (PDB 4GTS) was downloaded from the Protein Data Bank and prepared with Protein Preparation Wizard. In docking, the protein was held rigid, except hydroxyl and thiol groups were allowed to rotate. Binding modes/transition state poses were generated with Glide (Schrödinger release 2019–2: Glide, Schrödinger, LLC, New York, NY, 2019) with standard precision (SP) mode. Covalent docking was done with CovDock in Glide (Maestro 2019–2;^22^) with customised reaction chemistry. Pose for **7** was acquired with Induced fit docking (IFD; more details^17^).

**Table 2.** Effects of Imidazo[1,2-*a*]pyridine Analogues of α-phosphonocarboxylates on HeLa Cell Viability^a^ and Rab11A prenylation^b^

Compound	R	Reduction of HeLa cell viability (IC_50_/µM)^d^	Inhibition of Rab11A prenylation (LED/µM)^g^	Densitometry analyses of Rab11A in cytosolic fractions after treatment with 100 µM concentrations of compounds (% of control ± SD)^h^
7^f^	CH_3_	222^f^	10^f^	335 ± 69^f^
1a	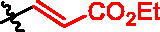	81	NE	–
1b	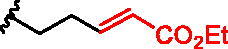	NE	NE	–
1c	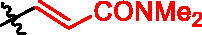	549	NE	–
2a	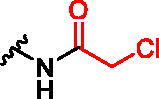	513	NE	–
3a	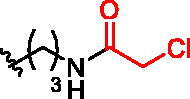	528	NE	–
**2b** ^e^	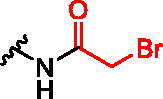	**154**	**25**	**608 ± 136**
2b incubated^c^		280	NE	–
3b	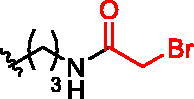	198	NE	–
3b incubated^c^		265	NE	–
2c	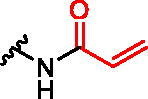	NE	NE	–
3c	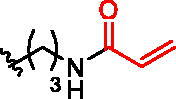	NE	NE	–
2d	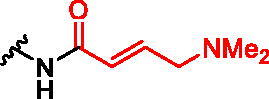	NE	NE	–
3d	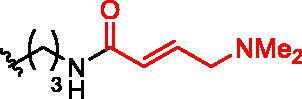	NE	NE	–
**1d** ^e^	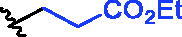	**755**	**50**	**440 ± 132**
**1e^e^**	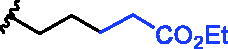	**996**	**25**	**244 ± 91**
1f	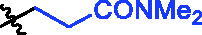	NE	NE	–
**2e^e^**	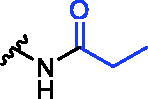	**154**	**25**	**659 ± 122**
3e	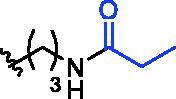	NE	NE	–

^a^Viability was measured after 72 h of incubation. Data represent the means from at least three independent experiments.

^b^Cells were treated for 48 h with 100 µM concentration of the compounds. Rab11A and β-actin were detected in cytosolic fraction using Western blot. Data, from at least three independent experiments, indicates which bands corresponding to Rab11A had higher intensity compared to non-treated control.

^c^Cells were treated with decomposition products of compounds **2b** and **3b** (PBS/D_2_O, 37 °C, 72 h).

^d^“NE” (not effective up to 2 mM concentration).

^e^Compounds in bold were evaluated to inhibit Rab11A and Rap1A/Rap1B prenylation in a wide concentration range.

^f^literature values determined using the same protocol.^17^.

^g^LED: lowest effective dose.”NE” (not effective at 100 µM concentration).

^h^Densitometry analysis representing relative unprenylated protein band intensity normalised to β-actin and quantified with respect to untreated cells.

### 2.6. Determination of 2b binding site to RGGT by mass spectrometry

IA-light and IA-heavy reactants were prepared according to the previously published procedure.^23^ RGGT, Rab7, and REP-1 were expressed in *Escherichia coli* and purified according to the published procedure (expression vectors were a kind gift from prof. Kirill Aleksandrov, University of Queensland, Australia).^24–27^ Protein complex formation was performed in 50 mM HEPES pH 7.2, 50 mM NaCl, 1 mM MgCl_2_ and 1 mM DTT in two quadruplicates (4 controls and 4 samples for inhibitor treatment). The reaction mixture containing 2 µM REP-1, 100 nM RGGT, 4 µM Rab7, and 100 µM inhibitor was incubated for 1 h at 37 °C with agitation. Simultaneously, inhibitor-free control samples were prepared. Solutions were treated with 0.2 mM probes (IA-light for inhibitor-treated or IA-heavy for control) at 25 °C for 1 h with constant mixing. Subsequently, the solutions were treated with 10 mM DTT at 65 °C for 15 min, and then 20 mM iodoacetamide at 37 °C for 30 min. One sample from the inhibitor-treated set was combined with one control sample and 250 µg of BSA after denaturation and alkylation steps. BSA was added to obtain a visible pellet and limit the loss of proteins of interest during the precipitation step. Methanol, chloroform, and ultrapure water were added sequentially (1:4:1:3 probe to methanol to chloroform to water ratio), the mixtures were vortexed and centrifuged (14 000 x g, 5 min). The top layers were removed, leaving the protein layers at the phase interface intact. Methanol (2 volumes) was added, the samples were gently mixed, and the protein pelleted as before. All supernatants were discarded, and the precipitated proteins were air-dried in an inverted tube for 5 min. Samples were resuspended in 2% SDS in PBS (100 µL). Once dissolved, the samples were diluted to a final concentration of 0.2% SDS by adding PBS. For each 1 ml of proteins solutions, click reagent mixture was prepared as follows: 20 µL CuSO_4_ (50 mM in H_2_O), 10 µL tris(2-carboxyethyl)phosphine hydrochloride (TCEP, 100 mM in H_2_O), 20 µL tris(3-hydroxypropyltriazolymethyl)amine (THPTA, 10 mM in DMSO) and 20 µL biotin-PEG_3_-azide (5 mM in DMSO) were added sequentially to a microcentrifuge tube and mixed. The click reagent mixtures were added to the protein solutions, giving the following final concentration of components: 1 mM CuSO_4_, 1 mM TCEP, 200 µM THPTA, 100 µM azide-PEG_3_-biotin, and the solutions were incubated on a shaker at 25 °C for 1 h. The click reactions were terminated by the addition of 10 mM EDTA. Protein samples were precipitated as described. Samples were resuspended in 100 µL of 6 M urea in 100 mM Tris-HCl pH 8.0 and 10 mM CaCl_2_, if they were digested with chymotrypsin, or 100 mM Tris-HCl pH 8.5, if they were digested with α-lytic protease, and then diluted to a final concentration of 0.6 M urea by addition of the buffer appropriate for selected proteases. Subsequently, after measurement of the total protein concentration in samples, chymotrypsin or α-lytic protease was added to a final protease:protein ratio of 1:30 (w/w) and incubated for 18 h at 25 °C for chymotrypsin or 37 °C for α-lytic protease. The reactions were terminated by the addition of 1:100 Protease Inhibitor Cocktail.

Peptide mixtures were then desalted with the use of AttractSPE™ Discs Bio – C18 (Affinisep, catalogue no. SPE-Discs-Bio-C18-100.T1.47.20) using a published stage-tip protocol,^28^ and dried in a centrifugal vacuum concentrator at 45 °C. Prior to LC-MS measurement, the samples were resuspended in 0.1% TFA, 2% acetonitrile in water. Chromatographic separation of peptides was performed on an Easy-Spray Acclaim PepMap column 50 cm long ×75 µm inner diameter (Thermo Fisher Scientific) at 45 °C by applying 60–90 min acetonitrile gradients in 0.1% aqueous formic acid at a flow rate of 300 nl/min. An UltiMate 3000 nano-LC system was coupled to a Q Exactive HF-X mass spectrometer via an easy-spray source (all Thermo Fisher Scientific). The Q Exactive HF-X was operated in data-dependent mode with survey scans acquired at a resolution of 60,000–120,000 at m/z 200 and MS/MS scans acquired at a resolution of 15,000–45,000 at m/z 200. Isotope patterns with charges 2–6 from the survey scan were selected (up to 12 per duty cycle) with an isolation window of 1.3 m/z and fragmented by higher-energy collision dissociation (HCD) with normalised collision energies of 27. Raw files generated from these measurements were processed in PEAKS Studio 10.5 (Bioinformatics Solutions Inc., Waterloo, Canada) and the peptides were identified from the MS/MS spectra searched against a short database containing the proteins subjected to incubation with **2b** as well as common proteinous contaminants. Methionine oxidation was set as variable modifications. For WaLP digested samples cleavages of Tyr/Ala/Ser/Val followed by any amino acid were allowed, for chymotrypsin digested samples cleavages of Phe/Leu/Tyr/Trp followed by any amino acid except Pro were allowed. Up to four missed cleavages were allowed. Parent mass error tolerance was set to 5 ppm and fragment mass error tolerance to 0.01 Da. The peptide-spectrum match quality threshold was set to *p* = 0.01. SILAC-based quantification method was applied with the light label set as Cys + 629.2996 Da and the heavy label was set as Cys + 635.3197 Da, and the maximal number of labelled amino acids per peptide set to 2. Other parameters were used as pre-set in the software. Peptide intensities were exported as csv files and formatted to the final version (Table 3) using Microsoft Office Excel 2016.

**Table 3.** Peptides identified in MS and intensity ratio of untreated vs treated with **2b** inhibitor samples.

Cys position in RABGGTB	Peptides identified	**Intensity ratio untreated vs 2b treated** ^a^
40	GSKKDDYEYCMSEY	0.83 (1)
78 & 82	IKSCQHECGGVSASIGHDPHLLY IKSCQHECGGVSASIGHDPHLL	1.06 ± 0.03 (6)
148	RFSFCAV SFCAVATL	0.90 ± 0.04 (7)
174	VLSCMNFDGGF VLSCMNF	0.91 ± 0.33 (2)
183	GCRPGSESHAGQIY	0.99 ± 0.03 (3)
196 & 197	GQIYCCTGFLA CCTGFLAITSQL	256^b^ (2)
220	GWWLCERQLPSGGL WLCERQLPSGGL LCERQLPSGGL	0.87 ± 0.53 (8)
240	NGRPEKLPDVCY	1.03 ± 0.10 (4)
270	ILACQDEETGGFADRPGDMVDPFHTLF ILACQDEETGGFADRPGDMVDPF ILACQDEETGGF	0.92 ± 0.06 (6)
314	SLLGEEQIKPVSPVFCMPEEVL CMPEEVL	0.93 ± 0.06 (5)

^a^Average value calculated based on data obtained from 8 LC-MS/MS measurements for the peptides listed ± StD, the number of individual ratios measured is given in parentheses; ^b^Signal of peptides from the **2b** treated samples was gone completely (in such cases, ratio = 256 is automatically assigned by the PEAKS software).

## 3. Results and discussion

**Scheme 1.**
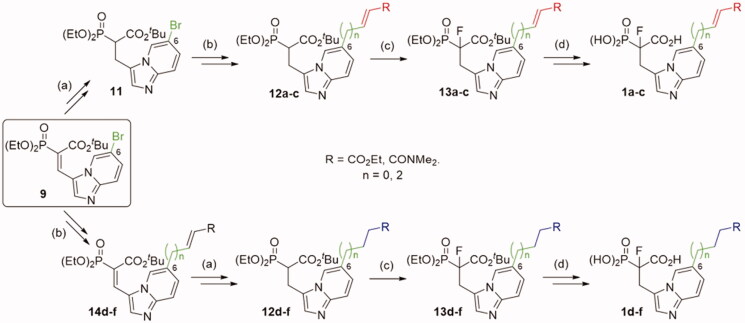
Strategies used for the synthesis of α-phosphonocarboxylates **1**. Reagents and conditions: (a) NaBH_4_, NiCl_2_·6H_2_O, MeOH; (b) for **12a,c** and **14d,f** (*n* = 0): *N,N*-dimethylacrylamide or ethyl acrylate, Pd(AcO)_2_, tri-*o*-tolylphosphine, DIPEA, propionitrile, microwave irradiation; for **12b** and **14e** (*n* = 2): (I) allyl alcohol, Pd(AcO)_2_, tri-*o*-tolylphosphine, DIPEA, propionitrile, microwave irradiation; (II) triethylphophonoacetate, NaH, THF; (c) NaH, NFSI, THF; (d) (I) bromotrimethylsilane, TEA, acetonitrile, (II) EtOH, (III) TFA.

**Scheme 2.**
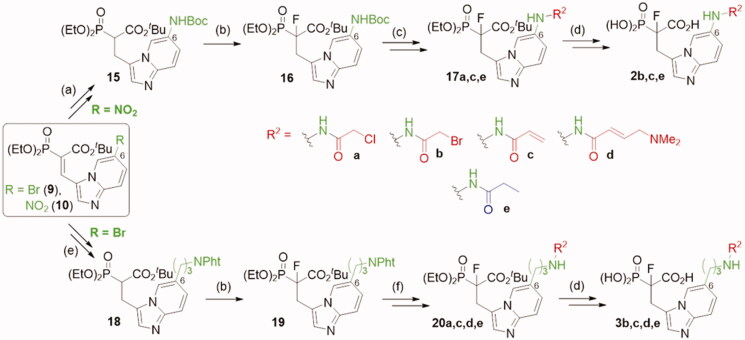
Strategies used for the synthesis of α-phosphonocarboxylates **2–3**.Reagents and conditions: (a) (I) **8**, H_2_, Pd/C, (II) Boc_2_O, DCM, (III) NaBH_4_, NiCl_2_·6H_2_O, MeOH; (b) NaH, NFSI, THF; (c) for **2 b,c**: (I) 3 M HCl/EtOH, (II) acyl halide, DCM; (d) (I) bromotrimethylsilane, TEA, acetonitrile, (II) EtOH, (III) TFA; (e) (I) **7**, 2-allylisoindoline-1,3-dione, Pd(AcO)_2_, tri-*o*-tolylphosphine, DIPEA, propionitrile, microwave irradiation, (II) H_2_, Pd/C, (III) NaBH_4_, NiCl_2_·6H_2_O, MeOH; (f) (I) hydrazine hydrate, MeOH; (II) acyl halide or carboxylic acid with coupling agent in DCM or DMF; For details of synthesis of **2a,d, 3a** see Schemes S7, S9.

Synthesis of novel imidazo[1,2-*a*]pyridine analogues of α-phosphonocarboxylates required four different routes (Schemes 1–2, for experimental details see: Schemes S2–S9). The first step involves the synthesis of 6-substituted-imidazo[1,2-*a*]pyridin-3-yl-2-(diethoxyphosphoryl)acrylates **9–10** according to the published procedure.^17^ In the synthesis of the compounds **1a,c** (Scheme 1), the double bond C_α_=C_β_ in **9** was reduced and thus obtained analogue **11** was subject to the Mizoroki-Heck reaction with appropriate olefin, giving compounds **12a,c**.^29^ In the synthesis of control analogues **1d,f**, the order of reactions was reversed, first the Mizoroki-Heck reaction^29^ and then the reduction of carbon-carbon double bonds was applied. In the case of analogues **1 b,e** with elongated carbon linker (*n* = 2), the Mizoroki-Heck reaction was carried out between allyl alcohol and **9**, giving appropriate aldehyde (Schemes S3 and S5),^29^ which was later used in Horner-Wadsworth-Emmons reaction with triethyl phosphonoacetate. Then, the products **12a-f** were fluorinated using N-fluorobenzenesulfonimide (NFSI) in the presence of sodium hydride.^19^ The ester groups of obtained compounds **13** were deprotected, applying bromotrimethylsilane for phosphonate, and TFA for *tert*-butyl carboxyester cleavage. After purification by HPLC, we obtained pure compounds **1a-f**.

Analogues **10** and **9** were used to synthesise compounds **2** and **3** (Scheme 2), which bear the amine group as an anchoring point for electrophilic modification, either directly connected with imidazo[1,2-*a*]pyridine ring or *via* a 3-carbon linker, respectively. For the synthesis of analogues **2**, compound **10** was first subjected to reduction of the nitro group, its protection by the Boc group, and the reduction of the double C=C bond, which led to compound **15**. In the case of analogues **3**, compound **9** was subjected to Mizoroki-Heck reaction with 2-allylisoindoline-1,3-dione^29^ and after reduction of two double C=C bonds, we obtained compound **18**. Next, analogues **15**, **18** were subjected to the fluorination with NFSI and NaH and the deprotection of the amine group, followed by its coupling with an acyl halide or a carboxylic acid. Thus obtained **17**, **20** were subjected to deprotection of the ester phosphonoacetate group, using bromotrimethylsilane and TFA. That approach worked for compounds **2 b,c,e,** and **3 b-e**, but failed for compounds **2a,d** and **3a**. Therefore, compounds **2a,d** were synthesised from **16** applying the reverse order of reactions, first deprotection of phosphonoacetate ester, followed by the introduction of the electrophilic moiety (Scheme S7). In the case of analogue **3a**, during the reaction of **20a** with bromotrimethylsilane, an exchange of chlorine atom for bromine was observed in chloroacetamide residue (Scheme S9). Therefore, **3b** was transformed into compound **3a** in the reaction with an excess of NaCl.

**Scheme 3.**
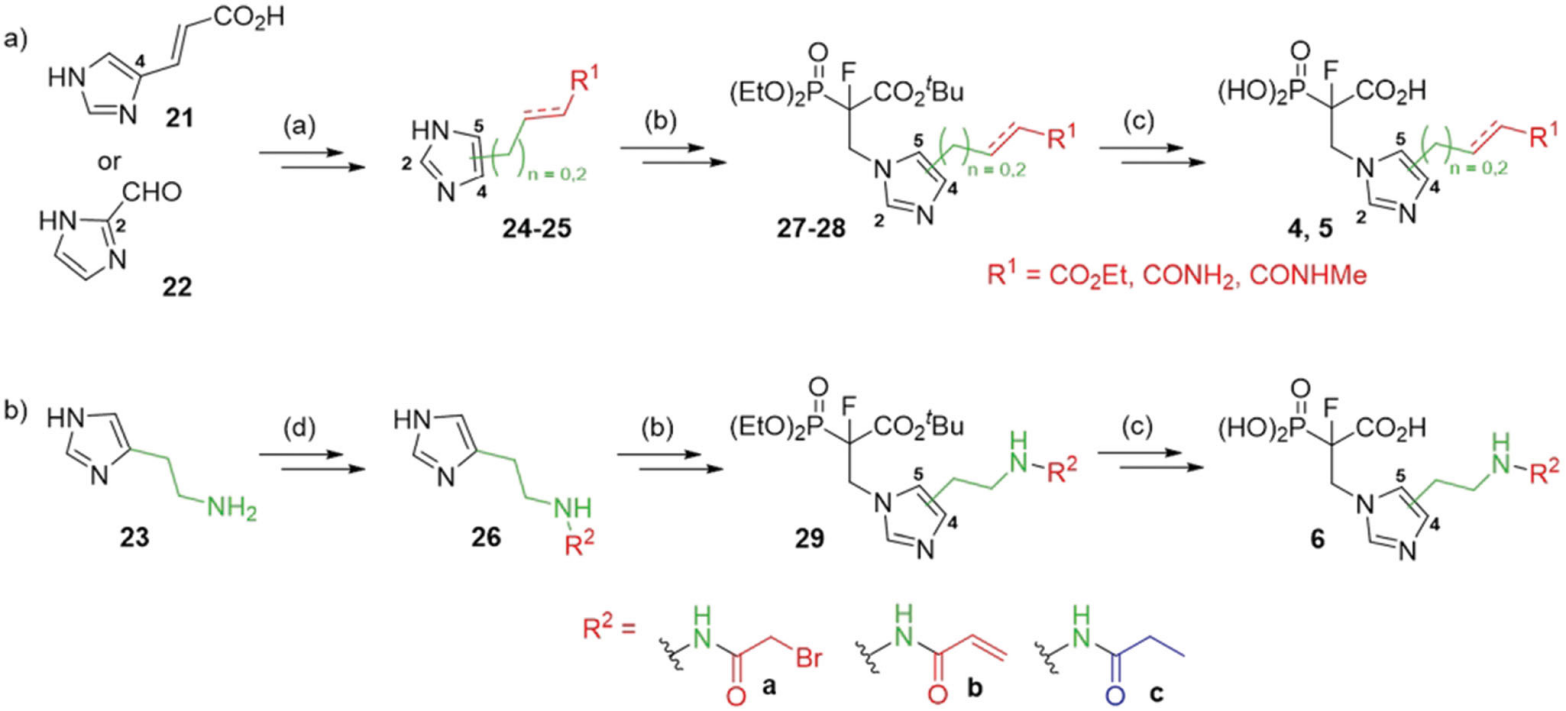
Strategies used for the synthesis of α-phosphonocarboxylates **4–6**. Reagents and conditions: (a) Scheme S10–14; (b) *tert*-butyl 2-(diethoxyphosphoryl)acrylate, NaH, THF, then NFSI, THF; (c) (I) bromotrimethylsilane, TEA, acetonitrile, (II) EtOH, (III) TFA; (d) (I) **23**, phthalic anhydride, AcOH, (II) Ph_3_Cl, TEA, (III) hydrazine hydrate, MeOH, (IV) acyl halide, DCM, (V) TFA, DCM.

We also synthesised analogues derived from **8**, modified at either the 2- or 4-position of the imidazole ring (Scheme 3). The first stages of the synthesis required the preparation of synthons **24–26,** which took from one to seven steps (Schemes S10–S14). The compounds **24–26** reacted with Michael acceptor, *tert*-butyl 2-(diethoxyphosphoryl)acrylate.^19^ The products were subjected to fluorination, using NaH and NFSI.^19^ Thus obtained **27–29** were subjected to deprotection of phosphonoacetate ester using the procedure optimised for analogues **1–3**.

We conducted stability studies of compounds **1–3** in phosphate buffer saline at physiological pH and temperature, monitored by ^1^H NMR. Compounds **4–6** were excluded from these studies as they did not show biological activity. All analogues were stable after 72 h, except for analogues of bromoacetamide **2b**, **3b**. After 72 h, only 35% of **2b** was left untouched, while **3b** completely decomposed.

We assessed the reactivity of **1–3** in the reaction with a model nucleophile containing cysteine, glutathione (GSH)^30^ at 37 °C in PBS/D_2_O by ^1^H NMR with dimethylformamide as a quantitative standard (Scheme S1). GSH has been used previously to screen and evaluate the reactivity of diverse electrophiles and as a model compound to establish a suitable ‘reactivity window’^31^ for covalent inhibitors. The progress of the reaction was based on the integration of disappearing signals corresponding with electrophilic groups, except for analogue **2a**, where the increase of integration of signals from the reaction product with GSH was measured. The highest reactivity was shown by bromoacetamide analogues **2b** and **3b**, whose complete conversion was observed within 15 min (Table 1).

**Table 1.** Effect of exchanging electrophilic residue on half-Life Values ​​t_1/2_ for reaction with GSH.

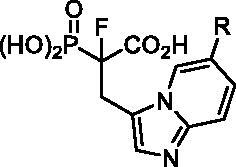		
compound	R^a^	t_1/2_^b^
**1a**	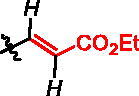	40 h
**1b**	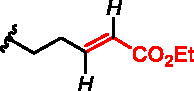	15 h
**1c**	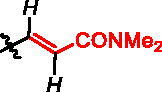	stable^e^
**2a** ^c^	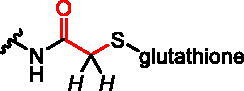	0.18 h
**2b**	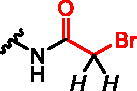	<0.25 h^d^
**2c**	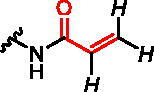	0.4 h
**2d**	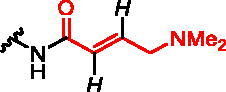	0.97 h
**3a**	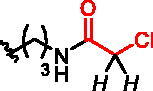	3.25 h
**3b**	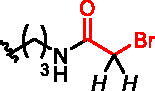	<0.25 h^d^
**3c**	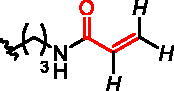	56 h
**3d**	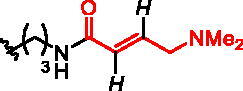	stable^e^

^a^in italic are indicated protons, which signals in the ^1^H NMR spectrum were used for estimation of the reaction’s progress; ^b^Obtained values were calculated by fitting to a pseudo-first-order kinetic equation: tested compound integration = a * exp (-k * time); where *a* and *k* - constants of a given reaction^31^; ^c^reaction progress based on increasing signal of reaction product with glutathione; ^d^observed complete conversion of the test compound in the first ^1^H NMR spectrum performed (after 15 min of reaction with GSH); ^e^no reaction during 11 h of incubation.

The biological activity of synthesised analogues was tested using human cervical carcinoma HeLa cells. Firstly, IC_50_ inhibitory concentrations of viability were estimated (Figures S1 and S2) since inhibition of prenyltransferases decreases cell growth.^15^^,^^17^

Among the first set of α-phosphonocarboxylates, only compounds **1a** and **1c**, bearing Cα = Cβ double bond adjacent to a heterocycle, showed antiproliferative activity, while **1b** with an elongated carbon linker (*n* = 2) was not active. Within their non-covalent counterparts (**1d-f**), only **1d** and its version with an elongated carbon linker, **1e**, reduced HeLa cells viability, however, **1f** was not active (Table 2).

**Figure 2.**
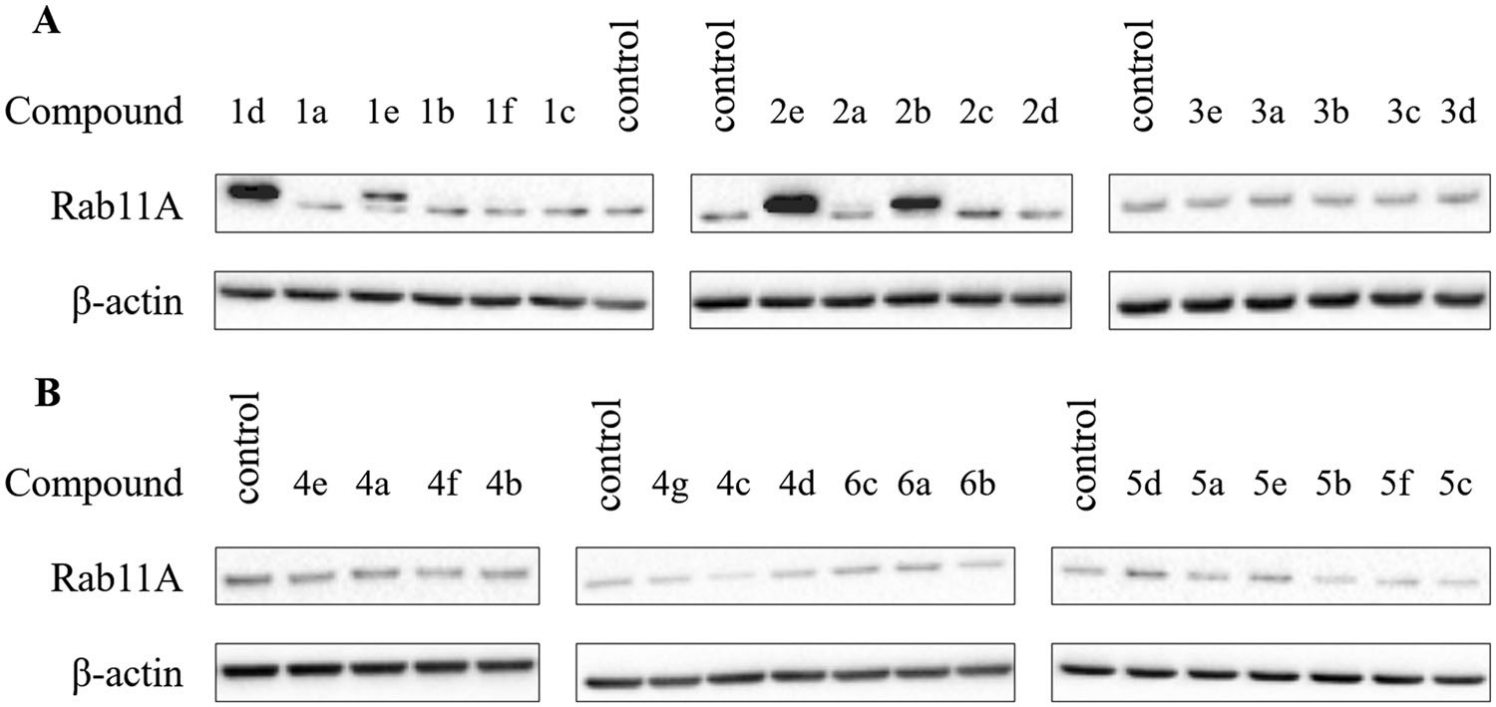
Effect of imidazo[1,2-*a*]pyridine (**1–3**; A) and imidazole (**4–6**; B) PC analogues on Rab11A prenylation in HeLa cells. Cells were treated for 48 h with PCs (100 µM). Rab11A and β-actin were detected in cytosolic fraction using Western blot.

Analogues **1d**, **1e**, **2b**, **2e** demonstrated inhibitory activity against RGGT at 100 µM (Figure 2) and therefore were selected to evaluate the lowest effective dose (LED) of inhibition of Rab11A and Rap1A/Rap1B prenylation (Table 3). Rap1A/Rap1B served as an indicator of PCs selectivity against RGGT because Rap1A/Rap1B is geranylgeranylated by GGTase-I.

**Figure 3.**
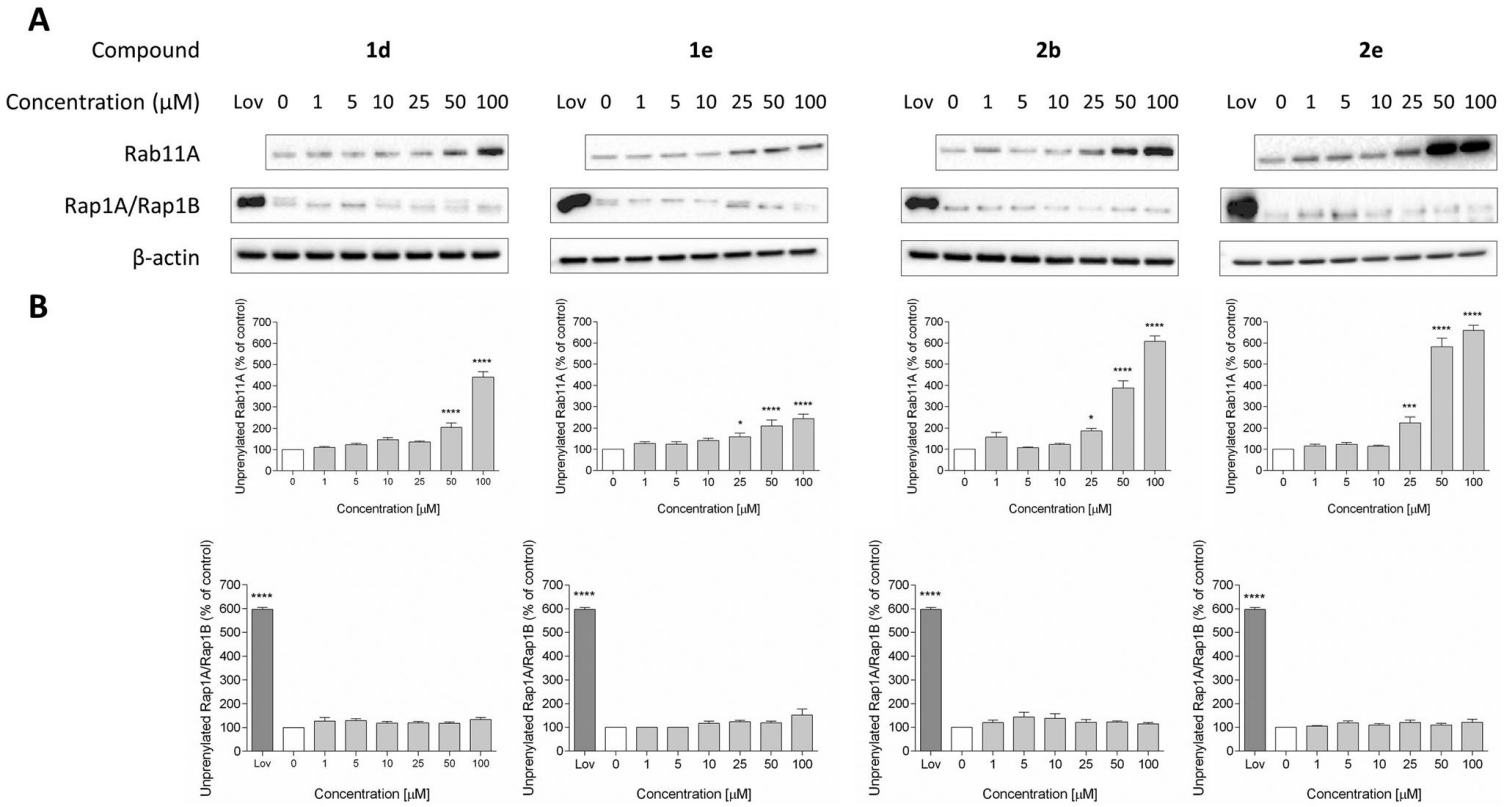
Effect of 6-substituted 3-IPEHPC analogue with the 3-ethoxy-3-oxopropyl group (**1d**), its version with an elongated carbon linker (*n* = 2; **1e**), bromoacetamide analogue (**2b**) and its non-covalent counterpart (**2e**) on Rab11A and Rap1A/Rap1B prenylation in HeLa cells. Cells were treated for 48 h with the indicated concentrations of PCs (µM) or 10 µM lovastatin (Lov), acting as a positive control. Cytosolic fractions containing unprenylated proteins were Western blotted for Rab11A, Rap1A/Rap1B, and β-actin (A), and the bands were quantified by densitometry, normalised to β-actin, and presented as a percentage of controls (B). Data represented mean ± SEM from at least three independent experiments, *p ≤ 0.05, ****p ≤ 0.0001.

Potential covalent inhibitors **1a, 1b,** and **1c** did not inhibit Rab11A prenylation. Therefore, their potency to reduce HeLa cell viability is probably associated with a different mechanism of action. Among the non-covalent counterparts **1d-f**, **1d,** and **1e** inhibited RGGT at 50 µM or 25 µM, respectively, and simultaneously, they did not affect the activity of GGTase-I (Figure 3). Non-covalent analogues of **7** (6-substituted analogues of 3-IPEHPC), **1d** and **1e,** are selective micromolar RGGT inhibitors with similar or slightly weaker activity than the reference compound **7**.^17^

The second group constituted compounds bearing amine group connected with imidazo[1,2-*a*]pyridine ring directly (**2**) or via a 3-carbon linker (**3**). **3e** and its covalent counterparts (**3a-d**) could not deplete Rab11A from HeLa cell membranes at 100 µM in contrary to the shorter non-covalent analogue, **2e**. Among analogues of **2e**, only **2b** was active against RGGT. It might be correlated with the highest reactivity of bromoacetamide modification observed in studies with GSH. Compound **2b** and its non-covalent version **2e** inhibited RGGT activity at 25 µM, but they were not active against other prenyltransferases or enzymes of the mevalonate pathway (Figure 3).

Analogues bearing bromoacetamide (**2b**, **3b**) and chloroacetamide (**2a**, **3a**) moieties were cytotoxic, while only **2b** showed inhibitory activity towards RGGT. The cytotoxicity of **2a** and **3a** was probably an effect of different mechanisms of action, due to the presence of highly reactive electrophilic moiety.^30^ Compounds **2c-3c** and **2d-3d** were the least reactive, which corresponded with their non-cytotoxic character.

**Figure 4.**
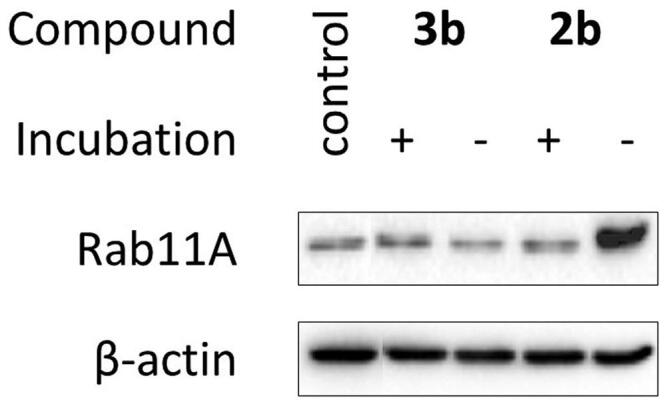
Effect of decomposition products of **2b** and **3b** on Rab11A prenylation in HeLa cells. Cells were treated for 48 h with 100 µM of freshly prepared **2b** and **3b** (-) and their solutions after incubation in PBS/D_2_O at 37 °C for 72 h (+). Cytosolic fractions containing unprenylated proteins were Western blotted for Rab11A and β-actin.

Because **2b** and **3b** were unstable, we supplemented HeLa cells with decomposition products. They turned out cytotoxic (Table 2), but none of them disrupted Rab11A prenylation (Figure 4). It indicates that inhibition of Rab11A protein prenylation by **2b** may arise from the action of the parent compound. Also, we cannot exclude that the biological effect of **2b** is comparable with its non-covalent analogue **2e**, only due to the instability of the former. The cytotoxic character of **3b** might result from the activity of degradation products and/or reactivity of the bromoacetamide group.

Imidazole analogues of α-phosphonocarboxylates **4–6**, did not exert any cytotoxic effect on HeLa cell proliferation (Figure S2) and they did not suppress prenylation of Rab11A at 100 µM (Figure 3B). It confirms data from our previous studies, where compounds with imidazole ring bearing two substituents, at nitrogen and C2 or C4, were inactive against RGGT.^19^

**Figure 5.**
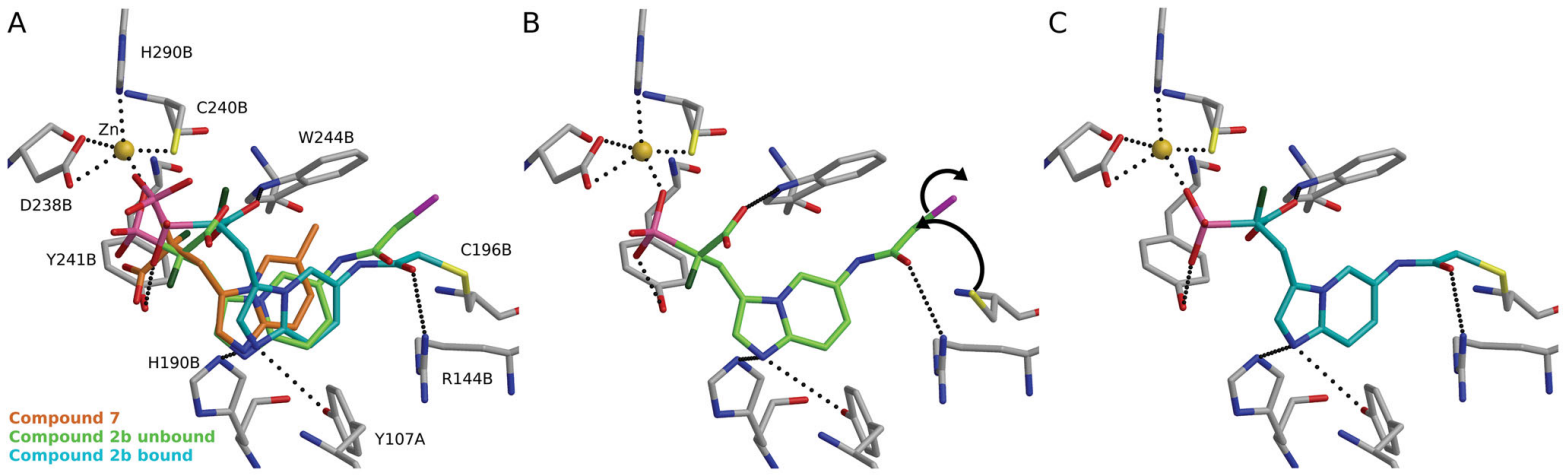
Possible covalent bond formation by compound **2b** suggested by docking simulations using 4GTS structure. **2b** adopts a similar binding mode as the non-covalent compound **7**^17^ (A). In not covalently bound state R144B stabilises **2b** into the proximity of C196B (B) before the formation of the covalent interaction (C). The black dashed lines indicate interactions between the inhibitor and the protein. Black arrows indicate the formation of covalent bond. Used atom colours: C in protein amino acids, **7**, **2b** in unbound state, and **2b** in bound state are grey, orange, light green, and light blue, respectively. O = red, N = blue, P = pink, S = yellow, F = dark green, Zn = golden, Br = magenta.

The proposed mechanism of the inhibitory activity of these compounds was rationalised with molecular docking. Several cysteines are present in the active site of the RGGT, which could be targeted by the electrophilic groups. We suggested that interactions of the acidic residues, phosphonic and carboxylic groups in α-phosphonocarboxylates, guide the location of the inhibitors in the RGGT binding area (Figure 5A; compound **7**). The interaction between REP-1-Rab complex and RGGT could change the conformation of the binding cavity,^10^ therefore in our previous studies, we applied induced-fit docking in which side chains of specific amino acids are allowed to move freely, and thus subtleties of the interactions between the ligand and the protein can be considered more accurately than with traditional molecular docking.^17^ Novel non-covalent compounds seem to behave similarly (Figure S3). The scaffold of the active covalent compound **2b** follows the suggested conformation of the previous compounds like **7** (Figure 5A). Additionally, the R144B could stabilise **2b** to the proximity of C196B in the transition state (Figure 5B). In addition, the hydrophobic part of the electrophilic moiety in position 6 faces the W244B indole side chain, yielding favourable packing. Finally, the covalent bond could be formed between **2b** and C196B (Figure 5C).

Also, other compounds (**2a**, **2c** and **2d**) seem to behave similarly in the docking experiment as **2b**. However, bond formation is sensitive to the correct angle and distance between the cysteine thiol group in RGGT and the electrophilic residue of an inhibitor. Therefore, small structural differences, such as the length of the linker or steric hindrance, should influence the efficiency of nucleophilic attack. Both compounds, **2c** and **2d** are longer than **2b** or **2a** (by one carbon) in terms of the carbon position, which is attacked. Additionally, **2d** bears steric hindrance making it less reactive. Interestingly, although **2a** and **2b** are structurally highly similar (the presence of either chlorine or bromine, in the electrophilic moiety), they significantly differ in activity, which might be the result of a weaker C-Br bond, making Br a better leaving group.^30^^,^^32^

Next, we performed mass spectrometry (MS) measurements to acquire further evidence for the covalent binding between **2b** and C196B. For the RABGGTB binding site determination, we adapted the active cysteine profiling method.^23^ Briefly, the proteins (RGGT, Rab7, REP-1) were incubated in the presence or absence of compound **2b**. Subsequently, the reaction mixtures were treated in parallel with a pan-cysteine reactive reagent, *N*-benzyl-2-iodo-*N*-(prop-2-yn-1-yl)acetamide, in two isotopic forms (IA-light: six ^12 ^C in benzene ring; IA-heavy: six ^13 ^C in benzene ring), readily distinguishable by MS (Figure S4). After denaturation and alkylation, the mixtures were combined, and proteins were digested with one of the two peptidases (chymotrypsin or wild-type α-lytic protease WaLP) to generate orthogonal peptidic fragments, with complete coverage of cysteines in RABGGTB. Ions corresponding to cysteine-containing peptides modified by IA-light and IA-heavy were identified and quantified based on the relative intensities measured for these isotopically distinct ions (Table 3). Any cysteine reacted with compound **2b** would lose its ability to subsequentially react with IA-light, and the concentration of the peptide containing that cysteine modified by IA-light would therefore decrease in the protein sample treated with **2b** in comparison with the sample not subjected to the treatment. The only RABGGTB cysteine-containing peptides for which intensities were severely decreased upon incubation with **2b** were peptides that correspond to sequences CCTGFLAITSQL (digestion with chymotrypsin) and GQIYCCTGFLA (digestion with WaLP), which contain C196B site, in addition to the inseparable vicinal C197B site. Guided by the molecular docking experiments, we anticipate the C196B as the major site of covalent binding to the inhibitor.

In conclusion, we present the first studies on the rational design of covalent Rab geranylgeranyltransferase inhibitor. For that purpose, we equipped the known non-covalent inhibitors with diverse electrophilic moieties. Using biological assays^17^ combined with mass spectrometric measurements^23^ and supported by molecular docking, we identified compound **2b** as an RGGT inhibitor. We propose that **2b** forms covalent bonding with the thiol group of the cysteine in the active site of RGGT.

The identification of the site of interaction has been achieved by using isotopically labelled probes and mass spectrometric analysis. It required optimisation in terms of finding the right digesting enzyme, as the standard trypsin could lead to very poor coverage of RABGGTB sequence, as predicted. Instead, we recommend the use of chymotrypsin and WaLP as they give complete coverage of cysteines. The experiment showed a significant decrease in the labelling of cysteines, C196 or/and C197, among 12 cysteines present in RABGGTB. While this experiment cannot distinguish between modifications of these neighbouring cysteines, the results of molecular docking indicate that the most optimal arrangement of bromoacetamide residue of the inhibitor and the thiol group of cysteine is possible for C196B. Additionally, the MS measurement did not reveal a substantial degree of modification by **2b** of the cysteine residues from the C-terminus of Rab7, the RGGT substrate, even though these are present in the active site during prenylation. This observation makes us think that the inhibitory character of **2b** results only from modification of RGGT.

Summarising, our data indicated that **2b** is the first selective RGGT inhibitor equipped with highly reactive bromoacetamide moiety, which presumably enables the formation of the covalent bond between analogue and the enzyme. The comparable activity against RGGT of **2b** and its non-covalent counterpart, propionamide analogue **2e**, might result from the low stability of bromoacetamide moiety.^33^ Efforts to increase the stability of inhibitor by changing bromine into chlorine in acetamide residue resulted in analogue **2a** with no activity against RGGT. Further work is needed in order to tune the reactivity of electrophilic moiety.

## Supplementary Material

Supplemental Material
