# Supplementary material for: Rational design, optimization, and biological evaluation of novel α-Phosphonopropionic acids as covalent inhibitors of Rab geranylgeranyl transferase
Source: J Enzyme Inhib Med Chem. 2022 Mar 30;37(1):940–51. doi: 10.1080/14756366.2022.2053525 (PMC8973367; doi:10.1080/14756366.2022.2053525)
Supplement: Supplemental Material [file IENZ_A_2053525_SM6153.pdf]

Supplemental Material

Rational Design, Optimization, and Biological Evaluation of Novel α–Phosphonopropionic Acids as Covalent Inhibitors of Rab Geranylgeranyl Transferase

Joanna Małolepsza,[a] Aleksandra Marchwicka,[b] Remigiusz A. Serwa,[c] Sanna P. Niinivehmas,[d,e] Olli T. Pentikäinen,[d,e] Edyta Gendaszewska-Darmach,[b] Katarzyna M. Błażewska [a] *

[a] Institute of Organic Chemistry, Faculty of Chemistry, Lodz University of Technology, Żeromskiego Street 116, 90-924 Łódź, Poland;

[b] Institute of Molecular and Industrial Biotechnology, Faculty of Biotechnology and Food Sciences, Lodz University of Technology, Stefanowskiego Street 2/22, 90-537 Łódź, Poland;

[c] ReMedy International Research Agenda Unit, IMol Polish Academy of Sciences, B. Smetany 2, 000-783 Warsaw, Poland

[d] Institute of Biomedicine, University of Turku, FI-20520 Turku, Finland

[e] InFLAMES Research Flagship Center, University of Turku, FI-20520 Turku, Finland

*Corresponding author: katarzyna.blazewska@p.lodz.pl

#These authors contributed equally.

Table of contents

Additional figures and schemes ______ *3*

Scheme S1. Model reaction between the inhibitor and glutathione presented on the example of compound 2c. ______ **3**

Figure S1. The cytotoxic efficacy of imidazo[1,2-a]pyridine analogues of α-phosphonocarboxylates against HeLa cell line. ______ **4**

Figure S2. The cytotoxic efficacy of imidazole analogues of α-phosphonocarboxylates against HeLa cell line. ______ **5**

Figure S3. Suggested binding modes of non-covalent reference compounds. ______ **6**

Figure S4. Schematic representation of workflow for the RABGGTB binding site determination using isotopically-labeled iodoacetamide-alkyne probes and quantitative mass spectrometry (MS). ______ **7**

Figure S4'. Sample MS/MS spectra and extracted ion chromatograms (XICx) for the Light (treated with compund 2b) and Heavy (untreated) peptides containing Cys270 (A) and Cys196/Cys197 (B). 8

Chemistry Experimental Procedures. 9

Scheme S2. Synthesis of compounds 1a,c. 10

Scheme S3. Synthesis of compound 1b. 14

Scheme S4. Synthesis of compounds 1d,f. 16

Scheme S5. Synthesis of compound 1e. 19

Scheme S6. Synthesis of compounds 2b,c,e. 21

Scheme S7. Synthesis of compounds 2a,d 25

Scheme S8. Synthesis of compounds 3b-e. 26

Scheme S9. Synthesis of compounds 3a 31

Scheme S10. Synthesis of compounds 24a,c,d,e,g. 31

Scheme S11. Synthesis of compounds 25a,c,d,f. 32

Scheme S12. Synthesis of compounds 24,b,f and 25b,f. 33

Scheme S13. Synthesis of compounds 4-5. 35

Scheme S14. Synthesis of compounds 6. 44

NMR spectra of selected compounds 49

References 145

Additional figures and schemes

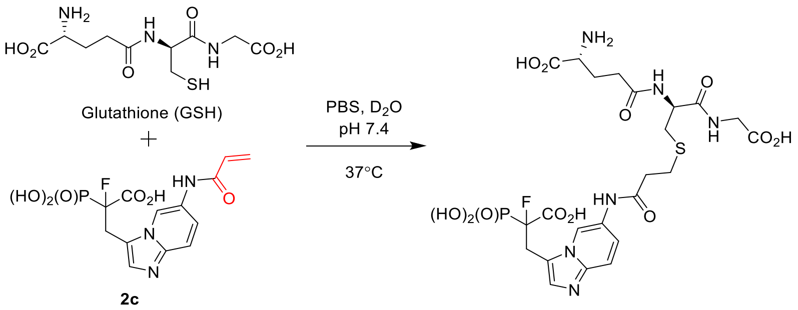

Scheme S1. Model reaction between the inhibitor and glutathione presented on the example of compound 2c.

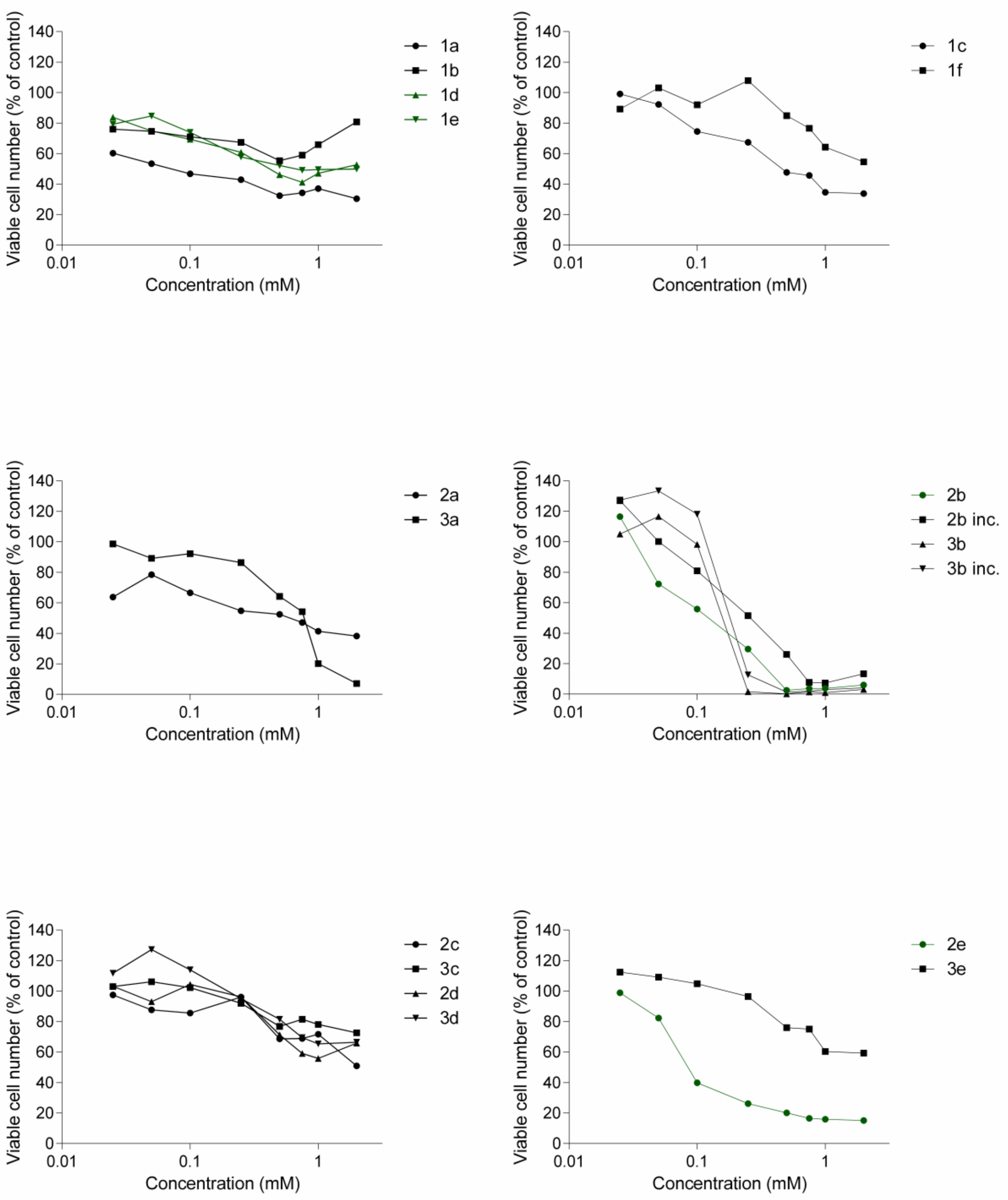

Figure S1. The cytotoxic efficacy of imidazo[1,2-a]pyridine analogues of α-phosphonocarboxylates against HeLa cell line. HeLa cells were treated with the compounds for 72 h and cell viability was determined with PrestoBlue® Cell Viability Reagent. Compounds which possessed ability to inhibit RGGT activity are highlighted in green. “inc.” abbreviation next to **2b** and **3b** denotes incubated variants of compounds.

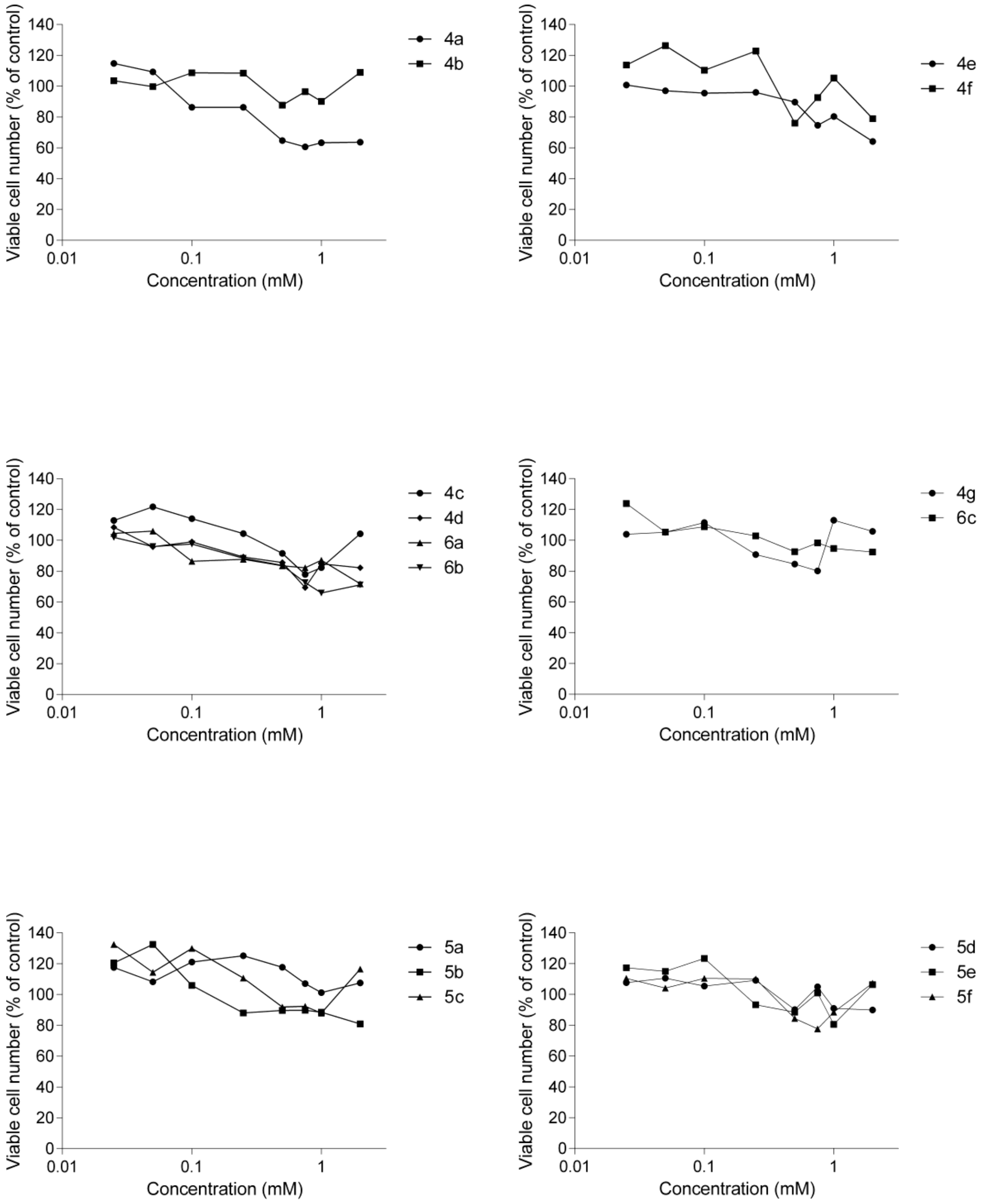

Figure S2. The cytotoxic efficacy of imidazole analogues of α-phosphonocarboxylates against HeLa cell line. HeLa cells were treated with the compounds for 72 h and cell viability was determined with PrestoBlue® Cell Viability Reagent.

The equations used to calculate the IC_{50} values for HeLa cell growth inhibition:

Compound	µM	Equation	Compound	µM	Equation
**1a**	81	y=-6,665ln(x)+33,272	**4a**	NE	y=-13,35ln(x)+63,306
**1b**	87	y=-1,64ln(x)+66,547	**4b**	NE	y=-1,4ln(x)+98,562
**1c**	549	y=-16,22ln(x)+40,266	**4c**	NE	y=-7,454ln(x)+91,198
**1d**	755	y=-9,049ln(x)+47,453	**4d**	NE	y=-6,555ln(x)+80,555

**1e**	996	y=-9,06ln(x)+49,966	**4e**	NE	y=-7,465ln(x)+77,291
**1f**	NE	y=-8,768ln(x)+72,374	**4f**	NE	y=-8,767ln(x)+91,545
**2a**	513	y=-7,998ln(x)+44,663	**4g**	NE	y=-1,814ln(x)+96,911
**2b**	154	y=-25,68ln(x)+2,0295	**5a**	NE	y=-2,479ln(x)+109,86
**2b inc.**	280	y=-29,17ln(x)+12,907	**5b**	NE	y=-10,71ln(x)+85,175
**2c**	NE	y=-8,958ln(x)+66,438	**5c**	NE	y=-7,562ln(x)+99,415
**2d**	NE	y=-11,36ln(x)+65,974	**5d**	NE	y=-4,52ln(x)+95,046
**2e**	154	y=-19,71ln(x)+13,089	**5e**	NE	y=-6,455ln(x)+94,556
**3a**	528	y=-20,18ln(x)+37,102	**5f**	NE	y=-4,562ln(x)+93,017
**3b**	198	y=-31,72ln(x)-1,3907	**6a**	NE	y=-6,627ln(x)+79,816
**3b inc.**	265	y=-37,36ln(x)+0,4339	**6b**	NE	y=-8,285ln(x)+73,602
**3c**	NE	y=-8,284ln(x)+78,012	**6c**	NE	y=-6,179ln(x)+94,102
**3d**	NE	y=-14,67ln(x)+71,806			
**3e**	NE	y=-13,55ln(x)+68,614			

x – concentration (mM)
y – viable cell number (% of control)

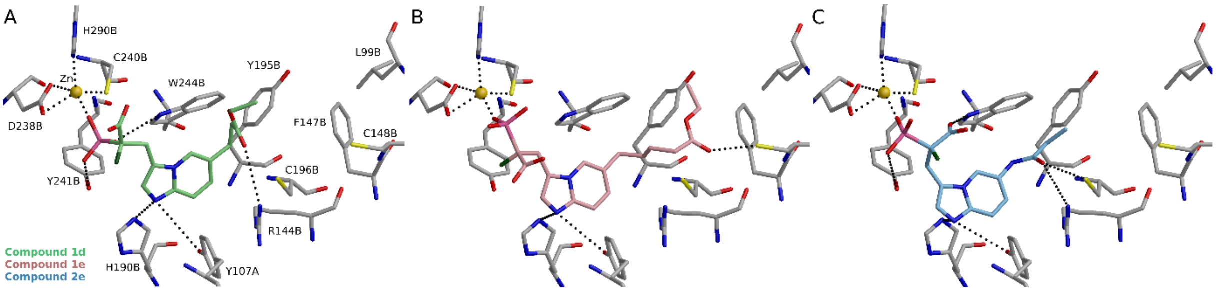

Figure S3. Suggested binding modes of non-covalent reference compounds. Non-covalent reference compounds **1e** (A), **1d** (B) and **2e** (C) adopt similar general binding mode as non-covalent compound **7**. Phosphonic and carboxylic groups in α-phosphonocarboxylates guide the location of the inhibitors in the RGGT binding area so that the phosphonic group coordinates to zinc ion. Furthermore, phosphonic group could also form a hydrogen bond with D238B and Y241B and W244B are in hydrogen bonding distance of the carboxylic acid group of the inhibitors. H190B and Y107A are in hydrogen bonding distance of N in the position 1 of the imidazo[1,2-*a*]pyridine ring. Binding of **1d** and **2e** is strengthened with a hydrogen bond of tail-part carboxylic acid and R144B and/or C196B. Longer compound **1e** finds a possible hydrogen bonding partner from C148B. In addition there are several hydrophopic residues, such as L99B, F147B, Y195B and W244B, that yield to favorable hydrophobic packing with the inhibitors. The black dashed lines indicate interactions between the inhibitor and the protein. Used atom colors: C in protein amino acids, **1d**, **1e**, and **2e** are grey, light green, light pink and light blue, respectively. O = red, N = blue, P = pink, S = yellow, F = dark green, Zn = golden.

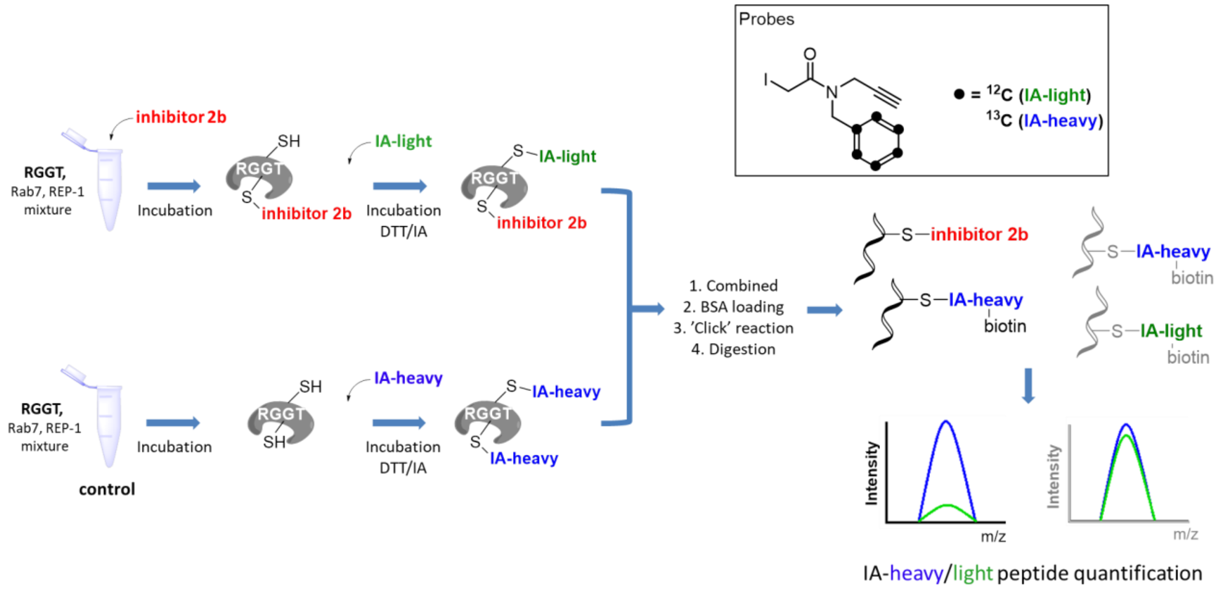

Figure S4. Schematic representation of workflow for the RABGGTB binding site determination using isotopically-labeled iodoacetamide-alkyne probes and quantitative mass spectrometry (MS). The MS quantification result indicates either labeling of proteome by inhibitor (graph described in black), or lack of labeling by inhibitor (graph described in grey).

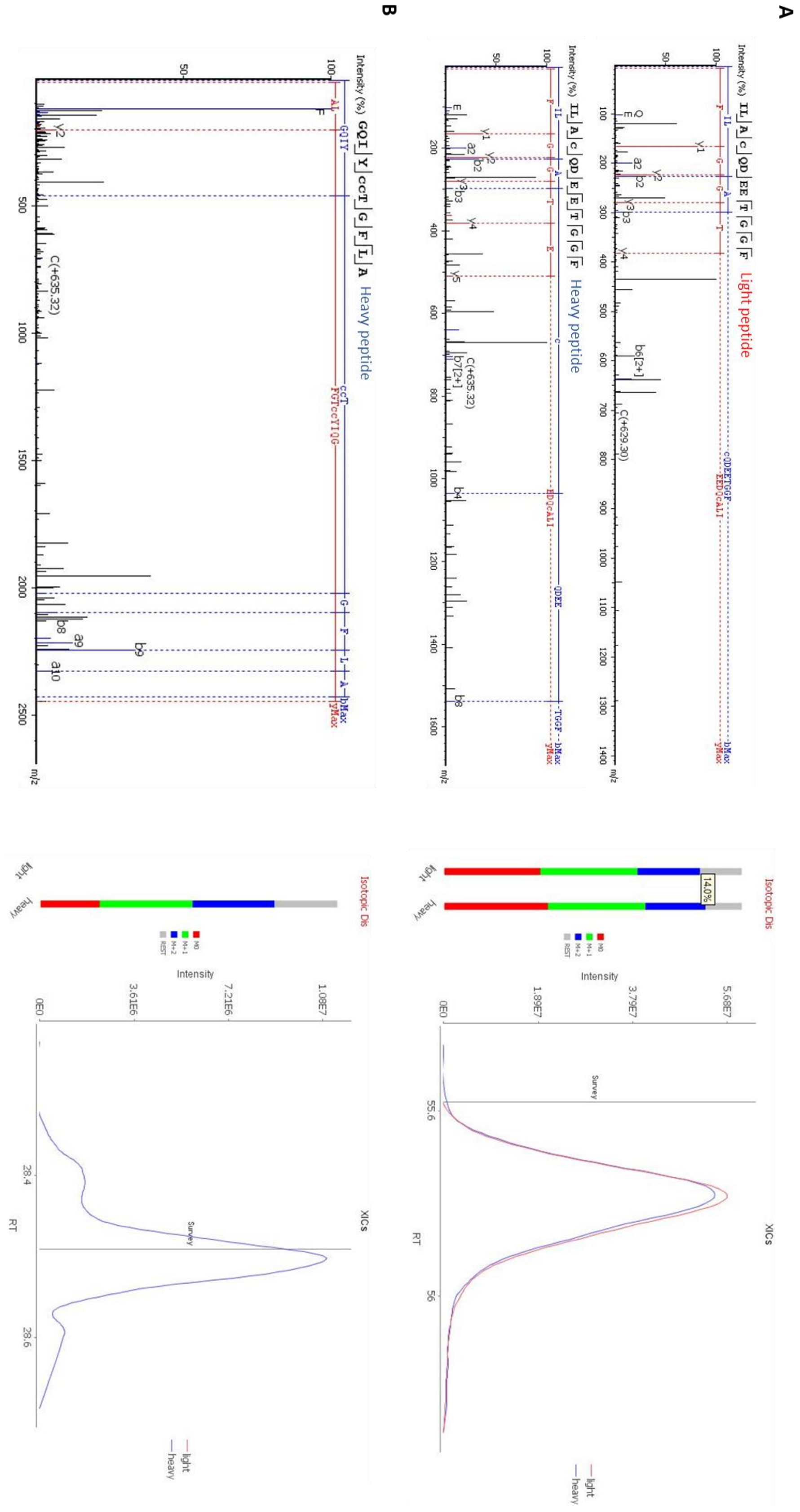

Figure S4'. Sample MS/MS spectra and extracted ion chromatograms (XICx) for the Light (treated with compund **2b**) and Heavy (untreated) peptides containing Cys270 (A) and Cys196/Cys197 (B). Areas under the curve for the XICs were used to calculate the „intensity ratio untreated vs **2b** treated" values reported in Table 3.

Chemistry Experimental Procedures.

*General procedure of ester deprotection - the synthesis of compounds **1-6***: appropriate substrate (compounds **13, 17, 20, 27-29**, 0.2 mmol) was placed in a single neck flask under an argon atmosphere in acetonitrile (2 mL). To the solution cooled to -20 °C bromotrimethylsilane (10 eq) was added dropwise within 5 minutes. The addition of TEA (10 eq) was required for selected reactions in order to avoid HBr addition to carbon double bonds. After 15 minutes, the reaction mixture was warmed to room temperature and continued stirring for 24-72 hours. Unreacted BTMS and solvent were evaporated providing anhydrous conditions. Next, EtOH (2 mL) was added to the obtained silyl ester for solvolysis. After 10 minutes of stirring the solvent was evaporated. Then, trifluoroacetic acid (TFA) (2 mL) was added and stirred for 2 hours at rt. The reagent was evaporated and the product was isolated using preparative HPLC using as eluents mixture of H_2O:ACN:TFA A= 95:5:0.1; B= 5:95:0.1 (gradient specified below) or by crystallization from EtOH or H_2O.

*General procedure of fluorination - the synthesis of compounds **13, 16, 19***: appropriate substrate (compound **12**, **15** or **18**, 0.18 mmol, 1 eq.) was added under argon atmosphere to a cooled (10 °C) suspension of NaH (2.6 mmol, 1.5 eq., 60% suspension in oil) in THF (4 mL) within 4 min. It was stirred for 50 min at 5 °C and then cooled to -70 °C followed by the addition of NFSI (1.3 eq.) in THF (4 mL) within 5 min. It was stirred for 20 min at -70 °C and overnight at rt. The reaction was quenched by the addition of H_2O (3 mL). After the addition of $CHCl_3$ (10 mL) the mixture was agitated and then the organic and aqueous phases were separated. The aqueous phase was additionally extracted with $CHCl_3$ (3x10 mL). Combined organic phases were dried over $MgSO_4$ and concentrated. Thus obtained oil was purified by column chromatography.

*General procedure of coupling reaction - the synthesis of compounds **17***: To compound **16** (100 mg) 3M HCl in EtOH (2 mL) was added. The mixture was stirred for 1h at rt. The mixture was concentrated under reduced pressure. Next, thus obtained amine derivative was dissolved in DCM (2 mL) and cooled to 0 °C. Then, TEA (5 eq.) was added followed by addition of appropriate acyl chloride (4 eq.). After 1h the reaction was quenched by addition of H_2O (3 mL). The organic and aqueous phases were separated. The aqueous phase (pH 9) was additionally extracted with $CHCl_3$ (3x10 mL). Combined organic phases were dried over $MgSO_4$ and concentrated. Thus obtained oil was purified by column chromatography.

*General procedure of coupling reaction - the synthesis of compounds **20***: To compound **19** (95 mg, 0.16 mmol) hydrazine hydrate (15 eq.) was added in MeOH (2 mL). The mixture was stirred at rt overnight. Obtained precipitate was removed by filtration and the filtrate was concentrated under reduced pressure. Next, thus obtained amine derivative was dissolved in DCM (2 mL) and cooled to 0°C. Then, TEA (5 eq.) was added followed by addition of appropriate acyl chloride (4 eq.). After 1h reaction was quenched by addition of H_2O (3 mL). The organic and aqueous phases were separated. The aqueous phase (pH 9) was additionally extracted with $CHCl_3$ (3x10 mL). Combined organic phases were dried over $MgSO_4$ and concentrated. Thus obtained oil was purified by column chromatography.

*General procedure of Michael addition and fluorination- the synthesis of compounds **27-29***: Reactions were carried out according to the literature procedure.[1] To *tert*-butyl 2-(diethoxyphosphoryl)acrylate (172 mg, 0.65 mmola, 1.0 eq.) in THF (4 mL), appropriate imidazole derivative (compounds **24-26**, 1.0 eq.) was added, and the resulting solution was stirred for 30 min at room temperature. The obtained adduct was directly subjected to fluorination. To the cooled (-20 °C) solution of aza-Michael adduct, NaH (30 mg, 0.76 mmol, 1.2 equiv, 60% suspension in oil) was added. The reaction mixture was stirred for 30 min at <10 °C and then cooled to -70 °C followed by the addition of NFSI (246 mg, 0.76 mmol, 1.2 equiv) in THF (2 mL) within 5 min. It was stirred for 20 min at -70 °C and then for 60 min at <20 °C. The reaction was quenched by the addition of H_2O (3 mL). After addition of $CHCl_3$ (10 mL) the mixture was agitated, and the organic and aqueous phases were separated. The aqueous phase was additionally extracted with $CHCl_3$ (3x10 mL). The combined organic phases were dried over $MgSO_4$ and concentrated. The thus obtained oil was purified by column chromatography.

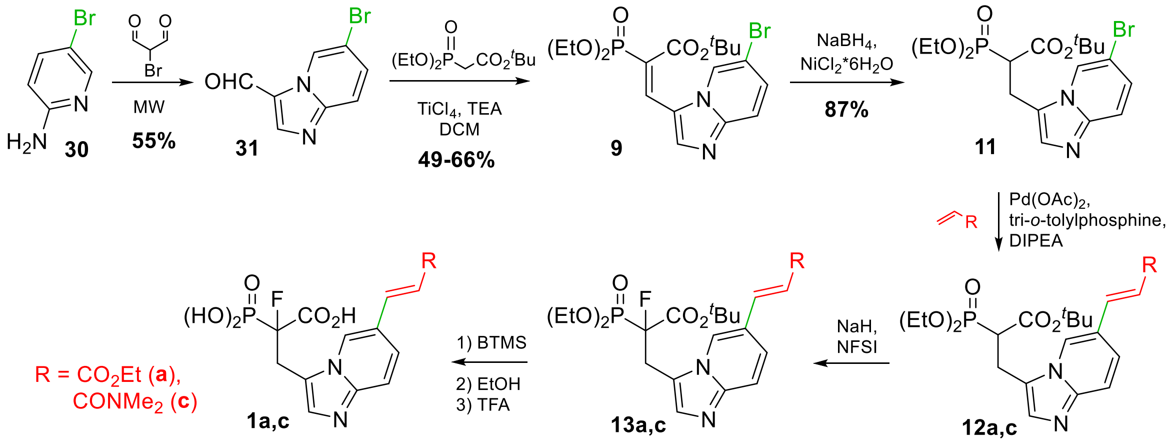

Scheme S2. Synthesis of compounds **1a,c.**

6-Bromoimidazo[1,2-*a*]pyridine-3-carbaldehyde (31): obtained according to the procedure from Kusy et al.[2]: 6-Bromo-2-aminopyridine (**30**) (414 mg, 1.2 mmol, 1 eq.) and 2-bromomalonaldehyde (1.5 eq.) were suspended in the mixture of ethanol and water (v/v 1:1, total 6 ml) and placed in the pressure vial, equipped with a magnetic bar. The mixture was stirred for 1 min and purge with argon via syringe. Then microwave (MW) irradiation (with initial 150 W power) was applied for 10 min. at 110 °C. Next, EtOH was evaporated, and the aqueous phase (pH 9) was extracted with DCM (5x10 mL). Combined organic phases were dried over $MgSO_4$ and concentrated. Product **31** was purified by column chromatography using gradient DCM/acetone (85:15, R_f = 0.4) as eluent to give product as yellow powder. Yield: 55%. **^{1}H NMR** (250 MHz, $CDCl_3$) δ 7.61 (dd, $^{3}J_{HH}$ = 9.4, $^{4}J_{HH}$ = 1.20, $C\underline{H}_{Ar(7)}$, 1H), 7.67 (d, $^{3}J_{HH}$ = = 9.40, $C\underline{H}_{Ar(8)}$, 1H), 8.28 (s, $C\underline{H}_{Ar(2)}$, 1H), 9.65 (bd, $^{4}J_{HH}$ = = 1.20 $C\underline{H}_{Ar(5)}$, 1H), 9.93 (s, $\underline{H}CO$, 1H), **^{13}C NMR** (63 MHz, $CDCl_3$) δ 110.36 (s, $\underline{C}_{Ar(6)}$, 1C), 118.40 (s, $\underline{C}H_{Ar(8)}$, 1C), 124.91 (s, $\underline{C}_{Ar(3)}$, 1C), 128.78 (s, $\underline{C}H_{Ar(5)}$, 1C), 133.48 (s, $\underline{C}H_{Ar(7)}$, 1C), 146.59 (s, $\underline{C}H_{Ar(2)}$, 1C), 147.71 (s, $\underline{C}_{Ar(9)}$, 1C), 178.02 (s, $H\underline{C}O$, 1C).

***Tert*-butyl 3-(6-bromoimidazo[1,2-*a*]pyridin-3-yl)-2-(diethoxyphosphoryl)acrylate (9):** In a dry and argon-purged double-neck flask equipped with thermometer and septum, *tert*-butyl 2-diethoxyacetate was placed (1.75 g, 6.9 mmol, 1,2 eq.) in DCM (20 mL). The solution was cooled to to -40 °C and then neat $TiCl_4$ (0.76 mL, 1.2 eq.) and TEA (2.25 mL, 16.2 mmol, 2.8 eq.) were added. After 15 min, a solution of aldehyde **31** (1.3 g, 5.78 mmol, 1.0 eq.) in DCM (20 mL) was added, and the reaction mixture was stirred overnight at room temperature. Next, water (25 mL) was the added, and the solution was adjusted to pH 9 with a saturated Na_2CO_3 solution. The product was extracted with $CHCl_3$ (4x25 mL). Combined organic phases were dried over $MgSO_4$ and concentrated. Compound **9** was purified by flash chromatography using Gilson PLC 2250 purification system. Eluents A: DCM and B: Acetone (gradient 0→30 min. 0→35% B, retention time 22 min.). Yield: 66%. The main fraction from flash chromatography contained only the (*E*)-isomer. **^{1}H NMR** (700 MHz, $CDCl_3$) δ 1.31 (t, $^{3}J_{HH}$ = 7.5, $C\underline{H}_3CH_2OP$, 6H), 1.51 (s, $C(C\underline{H}_3)_3$, 9H), 4.06 – 4.17 (m, $C\underline{H}_2OP$, 4H), 7.35 (dd, $^{3}J_{HH}$ = 9.4, $^{4}J_{HH}$ = 1.5, $C\underline{H}_{Ar(7)}$, 1H), 7.52 (d, $^{3}J_{HH}$ = 9.4, $C\underline{H}_{Ar(8)}$, 1H), 7.71 (d, $^{3}J_{PH}$ = 23.9, $C{=}C\underline{H}$, 1H), 8.36 (s, $C\underline{H}_{Ar(2)}$, 1H), 8.42 (bs, $C\underline{H}_{Ar(5)}$, 1H). **^{13}C NMR** (176 MHz, $CDCl_3$) δ 16.32 (d, $^{3}J_{PC}$ = 6.5, $\underline{C}H_3CH_2OP$, 2C), 28.04 (s, $C(\underline{C}H_3)_3$, 3C), 62.50 (d, $^{2}J_{PC}$ = 5.1, $\underline{C}H_2OP$, 2C), 82.83 (s, $\underline{C}(CH_3)_3$, 1C), 109.03 (s, $\underline{C}_{Ar(6)}$, 1C), 118.96 (s, $\underline{C}H_{Ar(8)}$, 1C), 119.78 (d, $^{1}J_{PC}$ = 181.4, $P\underline{C}$, 1C), 120.58 (d, $^{3}J_{PC}$ = 25.2, $\underline{C}_{Ar(3)}$, 1C), 124.09 (s, $\underline{C}H_{Ar(5)}$, 1C), 130.25 (s, $\underline{C}H_{Ar(7)}$, 1C), 131.54 (d, $^{2}J_{PC}$ = 10.1, $PC{=}\underline{C}H$, 1C), 140.59 (s, $\underline{C}H_{Ar(2)}$, 1C), 145.93 (s, $\underline{C}_{Ar(9)}$, 1C), 164.85 (d, $^{2}J_{PC}$ = 10.3, $\underline{C}O_2$, 1C). **^{31}P NMR** (283 MHz, $CDCl_3$) δ 15.93.

***Tert*-butyl 3-(6-bromoimidazo[1,2-*a*]pyridin-3-yl)-2-(diethoxyphosphoryl)propanoate (11):** To a solution of compound **9** (390 mg, 0.84 mmol, 1.0 eq.) in MeOH (8 mL) $NiCl_2{\cdot}6H_2O$ (240 mg, 1.2 eq.) was added. The solution was cooled to -50 °C, and $NaBH_4$ (0.083 g, 2.2 mmol, 1.2 eq.) was carefully added in three portions so as to maintain temperature below -20 °C. The mixture was then stirred for 10 min. Then, a few drops of saturated NH_4Cl amd water (5 mL) were added. The aqueous layer was extracted with CH_2Cl_2 (4x10 mL). The organic layer was dried over anhydrous Mg_2SO_4 and concentrated. Compound **11** was purified by flash chromatography using Gilson PLC 2250 purification system. Eluents A: DCM and B: Acetone (gradient 0→25 min. 0→40%B, retention time 22 min.). Yield: 87%. **^{1}H NMR** (700 MHz, $CDCl_3$) δ 8.18 (dd, $^{4}J_{HH}$ = 1.8, $^{5}J_{HH}$ = 0.8, $C\underline{H}_{Ar(5)}$, 1H), 7.49 (dd, $^{3}J_{HH}$ = 9.5, $^{5}J_{HH}$ = 0.8, $C\underline{H}_{Ar(8)}$, 1H), 7.45 (s, $C\underline{H}_{Ar(2)}$, 1H), 7.22 (dd, $^{3}J_{HH}$ = 9.5, $^{4}J_{HH}$ = 1.8, $C\underline{H}_{Ar(7)}$, 1H), 4.18-4.25 (m, $C\underline{H}_2OP$, 4H), 3.53 (ddd, $^{2}J_{HH}$ =15.8, $^{3}J_{HH}$ =11.5, $^{3}J_{PH}$ = 6.8 $C\underline{H}_2CHP$, 1H), 3.30 (ddd, $^{2}J_{HH}$ = 15.8, $^{3}J_{PH}$ = 10.0, $^{3}J_{HH}$ = 3.1 $C\underline{H}_2CHP$, 1H), 3.23 (ddd, $^{2}J_{PH}$ = 22.8, $^{3}J_{HH}$ = 11.5, $^{3}J_{HH}$ = 3.1 $CH_2C\underline{H}P$, 1H), 1.39 (s, $C(C\underline{H}_3)_3$, 9H), 1.35-1.38 (m, $C\underline{H}_3CH_2OP$, 6H). **^{13}C NMR** (176 MHz, $CDCl_3$) δ 167.34 (d, $^{2}J_{PC}$ = 5.1, $\underline{C}O_2$, 1C), 144.12 (s, $\underline{C}_{Ar(9)}$, 1C), 132.60 (d, $^{4}J_{PC}$ = 9.3, $\underline{C}H_{Ar(2)}$, 1C), 127.32 (s, $\underline{C}H_{Ar(7)}$, 1C), 123.57 (s, $\underline{C}H_{Ar(5)}$, 1C), 122.12 (d, $^{3}J_{PC}$ = 19.1, $\underline{C}_{Ar(3)}$, 1C), 118.72 (s, $\underline{C}H_{Ar(8)}$,

1C), 107.36 (s, $\underline{C}_{Ar(6)}$, 1C), 82.90 (s, $\underline{C}(CH_3)_3$, 1C), 63.24 (d, $^2J_{PC}$ = 6.8, $\underline{C}H_2OP$, 1C), 63.10 (d, $^2J_{PC}$ = 6.8, $\underline{C}H_2OP$, 1C), 45.35 (d, $^1J_{PC}$ = 128.5, $\underline{C}HP$, 1C), 27.94 (d, $^5J_{PC}$ = 3.5, $C(\underline{C}H_3)_3$, 3C), 16.53 (d, $^3J_{PC}$ = 3.1, $\underline{C}H_3CH_2OP$, 1C), 21.70 (s, $\underline{C}H_2CHP$, 1C), 16.56 (d, $^2J_{PC}$ = 3.1, $\underline{C}H_3CH_2OP$, 1C). **^{31}P NMR** (283 MHz, $CDCl_3$) δ 21.32.

Ethyl (*E*)-3-(3-(3-(*tert*-butoxy)-2-(diethoxyphosphoryl)-3-oxopropyl)imidazo[1,2-*a*]pyridin-6-yl)acrylate (12a): obtained according to Mizoroki–Heck reaction procedure from Kusy et al.[3] $Pd(OAc)_2$ (2.45 mg, 0.011 mmol, 0.2 eq.) and tri(*o*-tolyl)phosphine (0.18 eq.) were added to a solution of **11** (1 eq., 0,22 mmol, 100 mg), DIPEA (1.5 eq.), ethyl acrylate (1.1 equiv) in propiononitrile (2 mL), and the mixture placed in pressure vial equipped with a magnetic stirring bar. The mixture was stirred for 1 min and purged with argon via a syringe. Then, MW irradiation (with initial 150 W power) was applied for 50 min at 110 °C. Then, the reaction mixture was diluted with DCM (10 mL), and adsorbed on silica gel (≈ 3 g). Compound **12a** was purified by flash chromatography using Gilson PLC 2250 purification system. Eluents A: DCM and B: Acetone (gradient 5→35 min. 0→35%B, retention time 27 min.). Yield: 67%. **^{1}H NMR** (700 MHz, $CDCl_3$) *δ* 8.15 (bs, $C\underline{H}_{Ar(5)}$, 1H), 7.66 (d, $^3J_{HH}$ = 15.9, $C\underline{H}$=$CHCO_2$, 1H), 7.57 (bd, $^3J_{HH}$ = 9.5, $C\underline{H}_{Ar(8)}$, 1H), 7.43 (s, $C\underline{H}_{Ar(2)}$, 1H), 7.38 (dd, $^3J_{HH}$ = 9.5, $^4J_{HH}$ = 1.7, $C\underline{H}_{Ar(7)}$, 1H), 6.41 (d, $^3J_{HH}$ = 15.9, CH=$C\underline{H}CO_2$, 1H), 4.26 (q, $^3J_{HH}$ = 7.1, $CO_2C\underline{H}_2$, 2H), 4.15-4.22 (m, $C\underline{H}_2OP$, 4H), 3.55 (ddd, $^2J_{HH}$ = 15.7, $^3J_{HH}$ = 11.5, $^3J_{PH}$ = 6.9, $C\underline{H}_2CHP$, 1H), 3.33 (ddd, $^2J_{HH}$ = 15.7, $^3J_{PH}$ = 10.0, $^3J_{HH}$ = 3.2, $C\underline{H}_2CHP$, 1H), 3.23 (ddd, $^2J_{PH}$ = 22.8, $^3J_{HH}$ = 11.5, $^3J_{HH}$ = 3.2, $CH_2C\underline{H}P$, 1H), 1.35-1.37 (m, $C\underline{H}_3CH_2OP$, $C(C\underline{H}_3)_3$, 15H), 1.33 (t, $^3J_{HH}$ = 7.1, $CO_2CH_2C\underline{H}_3$, 3H). **^{13}C NMR** (176 MHz, $CDCl_3$) *δ* 167.32 (d, $^2J_{PC}$ = 5.1, $\underline{C}O_2{}^tBu$, 1C), 166.59 (s, $\underline{C}O_2Et$, 1C), 145.45 (s, $C_{Ar(9)}$, 1C), 140.64 (s, $\underline{C}H$=$CHCO_2$, 1C), 132.78 (s, $\underline{C}H_{Ar(2)}$, 1C), 125.13 (s, $\underline{C}H_{Ar(5)}$, 1C), 122.58 (d, $^3J_{PC}$ = 19.1, $\underline{C}_{Ar(3)}$, 1C), 121.22 (s, $\underline{C}H_{Ar(7)}$, 1C), 120.84 (s, $\underline{C}_{Ar(6)}$, 1C), 118.92 (s, CH=$\underline{C}HCO_2$, 1C), 118.37 (s, $\underline{C}H_{Ar(8)}$, 1C), 82.83 (s, $\underline{C}(CH_3)_3$, 1C), 63.22 (d, $^2J_{PC}$ = 6.4, $\underline{C}H_2OP$, 1C), 63.11 (d, $^2J_{PC}$ = 6.9, $\underline{C}H_2OP$, 1C), 60.78 (s, $CO_2\underline{C}H_2$, 1C), 45.41 (d, $^1J_{PC}$ = 129.2, $\underline{C}HP$, 1C), 27.90 (s, $C(\underline{C}H_3)_3$, 3C), 21.60 (d, $^2J_{PC}$ = 3.4, $\underline{C}H_2CHP$, 1C), 16.47-16.62 (m, $\underline{C}H_3CH_2OP$, 2C), 14.42 (s, $CO_2CH_2\underline{C}H_3$, 1C). **^{31}P NMR** (284 MHz, $CDCl_3$) *δ* 21.33. HRMS ($C_{23}H_{33}N_2O_7P + H^+$) m/z: calculated 481.2098, found 481.2097.

***Tert*-butyl (*E*)-2-(diethoxyphosphoryl)-3-(6-(3-(dimethylamino)-3-oxoprop-1-en-1-yl)imidazo[1,2-*a*]pyridin-3-yl)propanoate (12c):** obtained according to the Mizoroki–Heck reaction procedure for compound **12a**, using *N,N*-dimethyloacrylamide. Scale: 0.22 mmol of **11**. Compound **12c** was purified by flash chromatography using Gilson PLC 2250 purification system. Eluents A: DCM and B: Acetone (gradient 0→15 min. 0→30%B, retention time 33 min.). Yield: 69%. **^{1}H NMR** (700 MHz, $CDCl_3$) *δ* 8.16 (bs, $C\underline{H}_{Ar(5)}$, 1H) 7.67 (d, $^3J_{HH}$ = 15.3, $C\underline{H}$=CHCO, 1H), 7.58 (d, $^3J_{HH}$ = 9.4, $C\underline{H}_{Ar(8)}$, 1H), 7.44 (s, $C\underline{H}_{Ar(2)}$, 1H), 7.41 (dd, $^3J_{HH}$ = 9.4, $^4J_{HH}$ = 1.7, $C\underline{H}_{Ar(7)}$, 1H), 6.91 (d, $^3J_{HH}$ = 15.3, CH=$C\underline{H}$CO, 1H), 4.16 – 4.26 (m, $C\underline{H}_2OP$, 4H), 3.57 (ddd, $^2J_{HH}$ = 16.8, $^3J_{HH}$ = 11.6, $^3J_{PH}$ = 6.9, $C\underline{H}_2CHP$, 1H), 3.34 (ddd, $^2J_{HH}$ = 16.8, $^3J_{PH}$ = 9.8, $^3J_{HH}$ = 3.1, $C\underline{H}_2CHP$, 1H), 3.26 (ddd, $^2J_{PH}$ = 22.8, $^3J_{HH}$ = 11.6, $^3J_{HH}$ = 3.1, $C\underline{H}P$, 1H), 3.20 (s, $NC\underline{H}_3$, 3H), 3.09 (s, $NC\underline{H}_3$, 3H), 1.30-1.38 (m, $C\underline{H}_3CH_2OP$, $C(C\underline{H}_3)_3$, 15H). **^{13}C NMR** (176 MHz, $CDCl_3$) *δ* 167.25 (d, $^2J_{PC}$ = 5.1, $\underline{C}O_2$, 1C), 166.19 (s, $\underline{C}ONMe_2$, 1C), 145.32 (s, $C_{Ar(9)}$, 1C), 138.43 (s, $\underline{C}H$=CHCO, 1C), 132.51 (s, $\underline{C}H_{Ar(2)}$, 1C), 124.39 (s, $\underline{C}H_{Ar(5)}$, 1C), 122.40 (d, $^3J_{PC}$ = 19.0, $\underline{C}_{Ar(3)}$, 1C), 121.54 (s, $\underline{C}H_{Ar(7)}$, 1C), 121.48 (s, $\underline{C}_{Ar(6)}$, 1C), 118.07 (s, $\underline{C}H_{Ar(8)}$, 1C), 117.91 (s, $\underline{C}$HCON, 1C), 82.70 (s, $\underline{C}(CH_3)_3$, 1C), 63.15 (d, $^2J_{PC}$ = 6.4, $\underline{C}H_2OP$, 1C), 63.02 (d, $^2J_{PC}$ = 6.9, $\underline{C}H_2OP$, 1C), 45.27 (d, $^1J_{PC}$ = 129.1, $\underline{C}HP$, 1C), 37.49 (s, $N\underline{C}H_3$, 1C), 36.03 (s, $N\underline{C}H_3$, 1C), 27.85 (s, $C(\underline{C}H_3)_3$, 3C), 21.61 (d, $^2J_{PC}$ = 3.6, $\underline{C}H_2CHP$, 1C), 16.40-16.57 (m, $\underline{C}H_3CH_2OP$, 2C). **^{31}P NMR** (283 MHz, $CDCl_3$) *δ* 21.40. HRMS ($C_{23}H_{34}N_3O_6P + H^+$) m/z: calculated 480.2258, found 480.2261.

Ethyl (E)-3-(3-(3-(*ter*t-butoxy)-2-(diethoxyphosphoryl)-2-fluoro-3-oxopropyl)imidazo[1,2-*a*]pyridin-6-yl)acrylate (13a): obtained according to the *general procedure of fluorination*. Scale: 100 mg of **12a**. Compound **13a** was purified by flash chromatography using Gilson PLC 2250

purification system. Eluents A:DCM, B:Acetone, to both of them TEA was added, 1mL/(1L of eluent) (gradient 0→20 min. 0→30% B, retention time 9 min.). Yield: 49% (87 mg, purity 80% acourding to ^{31}P NMR spectrum). **^{1}H NMR** (700 MHz, $CDCl_3$) δ 8.23 (s, C$\underline{H}_{Ar(5)}$, 1H), 7.67 (d, $^{3}J_{HH}$ = 15.9, C$\underline{H}$=$CHCO_2$, 1H), 7.59 (d, $^{3}J_{HH}$ = 9.5, C$\underline{H}_{Ar}$, 1H), 7.53 (s, C$\underline{H}_{Ar(2)}$, 1H), 7.40 (dd, $^{3}J_{HH}$ = 9.4, $^{4}J_{HH}$=1.7, C$\underline{H}_{Ar}$, 1H), 6.42 (d, $^{3}J_{HH}$ = 15.9, CH=C$\underline{H}CO_2$, 1H), 4.32 – 4.25 (m, C$\underline{H}_2$OP, CO_2C$\underline{H}_2$, 6H), 3.80 (ddd, $^{3}J_{FH}$ = 37.4, $^{2}J_{HH}$ = 16.1, $^{3}J_{PH}$ = 5.8, C$\underline{H}_2$C(F)P, 1H), 3.70 (ddd, $^{2}J_{HH}$ = 15.9, $^{3}J_{FH}$ = 12.3, $^{3}J_{PH}$ = 6.8, C$\underline{H}_2$C(F)P, 1H), 1.40 (t, $^{3}J_{HH}$ = 7.1, C$\underline{H}_3CH_2$OP, 3H), 1.39 (s, C(C$\underline{H}_3)_3$, 9H), 1.38 (t, $^{3}J_{HH}$ = 7.1, C$\underline{H}_3CH_2$OP, 3H), 1.36 (t, $^{3}J_{HH}$ = 7.1, CO_2CH_2C$\underline{H}_3$, 3H). **^{31}P NMR** 12.04 (d, $^{2}J_{PF}$ = 82.4).

***Tert*-butyl (*E*)-2-(diethoxyphosphoryl)-3-(6-(3-(dimethylamino)-3-oxoprop-1-en-1-yl)imidazo[1,2-*a*]pyridin-3-yl)-2-fluoropropanoate (13c):** obtained according to the general procedure of fluorination. Scale: 150 mg (0.31 mmol) of **12c**. Compound **13c** was purified by flash chromatography using Gilson PLC 2250 purification system. Eluents A:DCM, B:Acetone, to both of them TEA was added, 1mL/(1L of eluent) (gradient 0→6 min. 0→30% B, retention time 27 min.). Yield: 64% (100 mg). **^{1}H NMR** (700 MHz, $CDCl_3$) δ 8.14 (s, C$\underline{H}_{Ar(5)}$, 1H), 7.58 (d, $^{3}J_{HH}$ = 15.3, C$\underline{H}$=CHCON, 1H), 7.49 (d, $^{3}J_{HH}$ = 9.4, C$\underline{H}_{Ar}$, 1H), 7.44 (s, C$\underline{H}_{Ar(2)}$, 1H), 7.36 (dd, $^{3}J_{HH}$ = 9.4, $^{4}J_{HH}$=1.7, C$\underline{H}_{Ar}$, 1H), 6.82 (d, $^{3}J_{HH}$ = 15.3, CH=C$\underline{H}$CON, 1H), 4.27 – 4.15 (m, C$\underline{H}_2$OP, 4H), 3.80 (ddd, $^{3}J_{FH}$ = 37.7, $^{2}J_{HH}$ = 16.2, $^{3}J_{PH}$ = 5.5, C$\underline{H}_2$C(F)P, 1H), 3.62 (ddd, $^{2}J_{HH}$ = 16.1, $^{3}J_{FH}$ = 11.9, $^{3}J_{PH}$ = 6.6, C$\underline{H}_2$C(F)P, 1H), 3.12 (s, C$\underline{H}_3$, 3H), 3.00 (s, C$\underline{H}_3$, 3H), 1.32 (t, $^{3}J_{HH}$ = 7.1, C$\underline{H}_3CH_2$OP, 6H), 1.30 (s, C(C$\underline{H}_3)_3$, 9H). **^{13}C NMR** (176 MHz, $CDCl_3$) δ 166.1 (s, $\underline{C}$ON, 1C), 164.9 (dd, $^{2}J_{FC}$ = 21.9, $^{2}J_{PC}$ = 3.7, $\underline{C}O_2t$Bu, 1C), 145.7 (s, $\underline{C}_{Ar(9)}$, 1C), 138.4 (s, CH=$\underline{C}$HCON, 1C), 134.8 (s, $\underline{C}H_{Ar(2)}$, 1C), 125.3 (d, J_{FC} = 5.0, $\underline{C}H_{Ar(5)}$, 1C), 121.7 (s, $\underline{C}H_{Ar}$, 1C), 121.3 (s, $\underline{C}_{Ar(6)}$, 1C), 117.9 (d, $^{3}J_{PC}$ = 18.4, $\underline{C}_{Ar(3)}$, 1C), 117.8 (s, $\underline{C}H_{Ar}$, $\underline{C}$H=CHCON, 2C), 95.6 (dd, $^{1}J_{FC}$ = 199.7, $^{1}J_{PC}$ = 159.6, $\underline{C}$(F)P, 1C), 84.5 (s, $CO_2\underline{C}Me_3$, 1C), 64.6 (d, $^{2}J_{PC}$ = 6.5, $\underline{C}H_2$OP, 1C), 64.4 (d, $^{2}J_{PC}$ = 6.4, $\underline{C}H_2$OP, 1C), 37.4 (bs, CH_3, 1C), 35.9 (bs, CH_3, 1C), 28.3 (d, $^{2}J_{FC}$ = 21.0, $\underline{C}H_2$C(F)P, 1C), 27.7 (s, C($\underline{C}H_3)_3$, 3C), 16.4 (d, $^{3}J_{PC}$ = 5.7, $\underline{C}H_3CH_2$OP, 2C). **^{31}P NMR** (283 MHz, $CDCl_3$) δ 12.62 (d, $^{2}J_{PF}$ = 83.2).

(*E*)-3-(6-(3-ethoxy-3-oxoprop-1-en-1-yl)imidazo[1,2-*a*]pyridin-3-yl)-2-fluoro-2-phosphonopropanoic acid (1a): obtained according to the general procedure of ester deprotection. Reaction with BTMS carried out in presence of TEA (10 eq). Scale: 87 mg of **13a**. Product **1a** was purified by preparative HPLC (gradient 3→20 min. 0→30% B, retention time 13.7 min.) followed by lyophilization. Yield: 59% (40 mg). **^{1}H NMR** (700 MHz, D_2O pH 3) δ 8.72 (s, C$\underline{H}_{Ar(5)}$, 1H), 7.84 (d, $^{3}J_{HH}$ = 16.0, CH=C$\underline{H}CO_2$, 1H), 7.71 (d, $^{3}J_{HH}$ = 9.0, C$\underline{H}_{Ar}$, 1H), 7.61 (d, $^{3}J_{HH}$ = 9.5, C$\underline{H}_{Ar}$, 1H), 7.50 (s, C$\underline{H}_{Ar(2)}$, 1H), 6.61 (d, $^{3}J_{HH}$ = 16.0, C$\underline{H}$=$CHCO_2$, 1H), 4.33 (q, $^{3}J_{HH}$ = 7.2, CO_2C$\underline{H}_2$, 2H), 4.05 (dd, $^{3}J_{FH}$ = 41.0, $^{2}J_{HH}$ = 16.1, C$\underline{H}_2$C(F)P, 1H), 3.71 – 3.63 (m, C$\underline{H}_2$C(F)P, 1H), 1.38 (t, $^{3}J_{HH}$ = 7.2, CO_2CH_2C$\underline{H}_3$, 3H). **^{13}C NMR** (176 MHz, D_2O pH 3) δ 174 (s, $\underline{C}O_2H$, 1C), 168.5 (s, $\underline{C}O_2Et$, 1C), 141 (s, $\underline{C}_{Ar(9)}$, 1C), 140.1 (s, CH=$\underline{C}HCO_2$, 1C), 128.5 (s, $\underline{C}H_{Ar}$, 1C), 127.6 (s, $\underline{C}H_{Ar(5)}$, 1C), 124.2 (s, $\underline{C}H_{Ar(2)}$, 1C), 123.6 (s, $\underline{C}_{Ar(6)}$, 1C), 122. 9 (s, $\underline{C}_{Ar(3)}$, 1C), 120.4 (s, $\underline{C}H$=$CHCO_2$, 1C), 113.3 (s, $\underline{C}H_{Ar}$, 1C), not visible ($\underline{C}$(F)P, 1C), 62.0 (s, $CO_2\underline{C}H_2$, 1C), 28.3 (d, $^{2}J_{FC}$ = 19.4, $\underline{C}H_2$C(F)P, 1C), 13.4 (s, $CO_2CH_2\underline{C}H_3$, 1C). **^{31}P NMR** (283 MHz, D_2O pH 3) δ 10.22 (d, $^{2}J_{PF}$ = 72.4). HR-MS: m/z [M+H^{+}] calculated 387.0752, found 387.0750.

(*E*)-3-(6-(3-(dimethylamino)-3-oxoprop-1-en-1-yl)imidazo[1,2-*a*]pyridin-3-yl)-2-fluoro-2-phosphonopropanoic acid (1c): obtained according to the general procedure of ester deprotection. Reaction with BTMS carried out in presence of TEA (10 eq). Scale: 100 mg of **13c**. Product **1c** was purified by preparative HPLC (gradient 3→20 min. 0→30% B, retention time 10.1 min.) followed by lyophilization. Yield: 56% (43 mg). **^{1}H NMR** (700 MHz, D_2O pH 4) δ 8.94 (s, C$\underline{H}_{Ar(5)}$, 1H), 8.26 (d, $^{3}J_{HH}$ = 9.6, C$\underline{H}_{Ar}$, 1H), 7.93 (d, $^{3}J_{HH}$ = 9.5, C$\underline{H}_{Ar}$, 1H), 7.87 (s, C$\underline{H}_{Ar(2)}$, 1H), 7.61 (d, $^{3}J_{HH}$ = 15.7, CH=C$\underline{H}$CON, 1H), 7.30 (d, $^{3}J_{HH}$ = 15.7, C$\underline{H}$=CHCON, 1H), 4.07 (ddd, $^{3}J_{FH}$ = 37.9, $^{2}J_{HH}$ = 16.4, $^{3}J_{PH}$ =

3.8, C$\underline{H}_2$C(F)P, 1H), 3.89 (ddd, $^2J_{HH}$ = 16.4, $^3J_{FH}$ = 9.4, $^3J_{PH}$ = 6.9, C$\underline{H}_2$C(F)P, 1H), 3.29 (s, C$\underline{H}_3$, 3H), 3.10 (s, C$\underline{H}_3$, 3H). **^{13}C NMR** (176 MHz, D_2O pH 4) δ 174.5 (d, $^2J_{FC}$ = 23, $\underline{C}O_2H$, 1C), 168.1 (s, $\underline{C}$ON, 1C), 142.0 (s, $\underline{C}_{Ar(9)}$, 1C), 137.4 (s, $\underline{C}$H=CHCON, 1C), 127.1 (s, $\underline{C}H_{Ar}$, 1C), 126.9 (s, $\underline{C}H_{Ar(5)}$, 1C), 126.1 (s, $\underline{C}H_{Ar(2)}$, 1C),123.4 (s, $\underline{C}_{Ar(6)}$, 1C), 122.6 (d, $^3J_{PC}$ = 14, $\underline{C}_{Ar(3)}$, 1C), 119. 6 (s, CH=$\underline{C}$HCON, 1C), 113.9 (s, $\underline{C}H_{Ar}$, 1C), 99 (m, $\underline{C}$(F)P, 1C, in HMBC spectrum), 37.6 (s, $\underline{C}H_3$, 1C), 35.9 (s, $\underline{C}H_3$, 1C), 28.4 (d, $^2J_{FC}$ = 19.7, $\underline{C}H_2$C(F)P, 1C). **^{31}P NMR** (283 MHz, D_2O pH 4) δ 7.9 (d, $^2J_{PF}$ = 69). HR-MS: m/z [M+H$^+$] calculated 386.0912, found 386.0911.

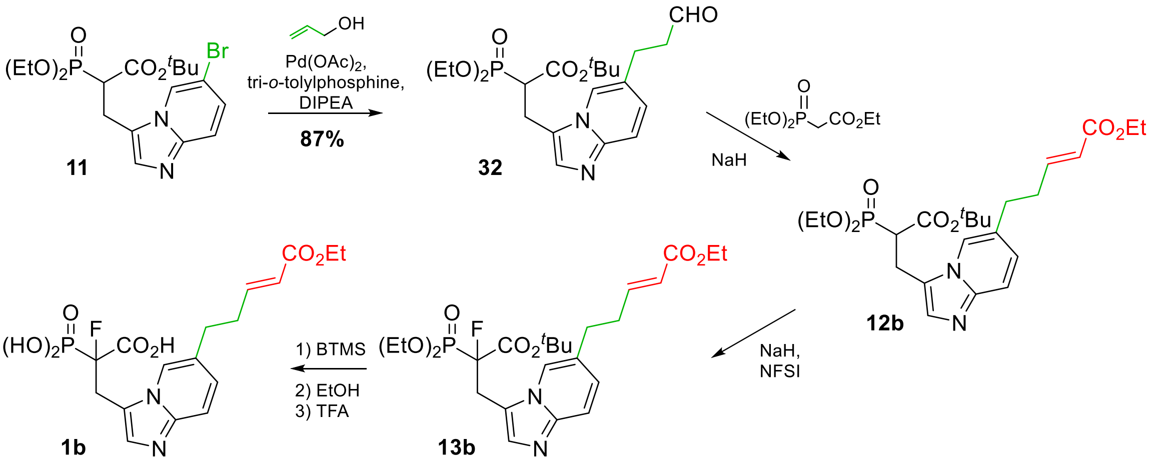

Scheme S3. Synthesis of compound **1b.**

***Tert*-butyl 2-(diethoxyphosphoryl)-3-(6-(3-oxopropyl)imidazo[1,2-*a*]pyridin-3-yl)propanoate (32):** obtained according to the Mizoroki–Heck reaction procedure for compound **12a**, using allyl alcohole. Scale: 0.22 mmol of **11**. Compound **32** was purified by flash chromatography using Gilson PLC 2250 purification system. As eluent mixture of A: DCM and B: Acetone was used (gradient 0→30 min. 10→50%B, retention time 33 min.). Yield: 57% (70% purity according to ^{31}P NMR spectrum). **^{1}H NMR** (250 MHz, $CDCl_3$) δ 9.85 (t, $^3J_{HH}$ = 0.9, C$\underline{H}$O, 1H), 7.93 (bs, C$\underline{H}_{Ar(5)}$, 1H), 7.66 (d, $^3J_{HH}$ = 9.3, $^5J_{HH}$ = 0.9, C$\underline{H}_{Ar(8)}$, 1H), 7.43 (s, C$\underline{H}_{Ar(2)}$, 1H), 7.13 (dd, $^3J_{HH}$ = 9.3, $^4J_{HH}$ = 1.7, C$\underline{H}_{Ar(7)}$, 1H), 4.08 – 4.29 (m, C$\underline{H}_2$OP, 4H), 3.44-3.65 (m, C$\underline{H}_2$CHP, 1H), 3.12-3.41 (m, C$\underline{H}_2$CHP, CH_2C$\underline{H}$P, 2H), 2.94-3.04 (m, CHOCH_2C$\underline{H}_2$, 2H), 2.76-2.90 (m, CHOC$\underline{H}_2$, 2H), 1.25-1.42 (m, C(C$\underline{H}_3$)$_3$, C$\underline{H}_3$CH_2OP, 15H). **^{31}P NMR** (101 MHz, $CDCl_3$) δ 21.87. HRMS ($C_{21}H_{31}N_2O_6P$ + H$^+$) m/z: calculated 439.1992, found 439.2006.

Ethyl (*E*)-5-(3-(3-(*tert*-butoxy)-2-(diethoxyphosphoryl)-3-oxopropyl)imidazo[1,2-*a*]pyridin-6-yl)pent-2-enoate (12b): obtained in Horner–Wadsworth–Emmons reaction. To a cooled (-20 °C) solution of triethyl phosphonoacetate (1.1 eq.) in THF (2 mL), NaH (1,1 eq., 60% suspension in mineral oil) was added, and the resulting solution was stirred for 30 min at room temperature. Next, the reaction mixture was cooled to -20 °C and compound **32** (1 eq., 0.27 mmol, 120 mg) was added. It was stirred for 1.5h at room temperature. The reaction was quenched by the addition of saturated NH_4Cl (0.5 mL) and water (5 mL). After addition of $CHCl_3$ (10 mL) the mixture was agitated, and the organic and aqueous phases were separated. The aqueous phase was additionally extracted with $CHCl_3$ (3x10 mL). The combined organic phases were dried over $MgSO_4$ and concentrated. Compound **12b** was purified by flash chromatography using Gilson PLC 2250 purification system. Eluents A: $CHCl_3$ and B: Acetone (gradient 0→50 min. 0→45%B, retention time 42 min.). Yield: 55% (76% purity according to ^{31}P NMR spectrum). **^{1}H NMR** (250 MHz, $CDCl_3$) δ 7.82 (s, C$\underline{H}_{Ar}$, 1H), 7.53 (d, $^3J_{HH}$ = 9.2, C$\underline{H}_{Ar}$, 1H), 7.40 (s, C$\underline{H}_{Ar}$, 1H), 7.03 (dd, $^3J_{HH}$ = 9.4, $^4J_{HH}$ = 1.6, C$\underline{H}_{Ar}$, 1H), 6.98 (dt, $^3J_{HH}$ = 15.7,

$^{3}J_{HH}$ = 6.8, $C\underline{H}CHCO_2$, 1H), 5.86 (d, $^{3}J_{HH}$ = 15.6, $CHC\underline{H}CO_2$, 1H), 4.31 – 4.09 (m, $POC\underline{H}_2$, CO_2CH_2, 6H), 3.54 (ddd, J = 15.9, $^{3}J_{HH}$ = 11.8, J = 6.2, $PC\underline{H}$ lub $PCHC\underline{H}_2$, 1H), 3.39 – 3.14 (m, $PC\underline{H}$ lub $PCHC\underline{H}_2$, 2H), 2.79 (t, $^{3}J_{HH}$ = 7.7, $C_{Ar(6)}C\underline{H}_2CH_2$, 2H), 2.56 (dt, $C\underline{H}_2CH$, 2H), 1.38 (s, $C(C\underline{H}_3)_3$, 9H), 1.37 (t, $^{3}J_{HH}$ = 7, $POCH_2C\underline{H}_3$, 6H), 1.28 (t, $^{3}J_{HH}$ = 7.1, $CO_2CH_2C\underline{H}_3$, 4H). **^{31}P NMR** (101 MHz, $CDCl_3$) δ 22.05.

Ethyl (*E*)-5-(3-(3-(*tert*-butoxy)-2-((diethoxyphosphaneyl)oxy)-2-fluoro-3-oxopropyl)imidazo[1,2-*a*]pyridin-6-yl)pent-2-enoate (13b): obtained according to the *general procedure of fluorination*. Scale: 140 mg of **12b**. Compound **13b** was purified by flash chromatography using Gilson PLC 2250 purification system. Eluents A:DCM, B:Acetone, to both of them TEA was added, 1mL/(1L of eluent) (gradient 0→35 min. 10→35% B, retention time 23 min.). Yield: 70% (102 mg, purity 80% acourding to ^{31}P NMR spectrum). **^{1}H NMR** (700 MHz, $CDCl_3$) δ 7.85 (s, $C\underline{H}_{Ar(5)}$, 1H), 7.44 (d, $^{3}J_{HH}$ = 9.2, $C\underline{H}_{Ar(8)}$, 1H), 7.40 (s, $C\underline{H}_{Ar(2)}$, 1H), 6.96 (dd, $^{3}J_{HH}$ = 9.2, $^{4}J_{HH}$=1.7, $C\underline{H}_{Ar(7)}$, 1H), 6.89 (dt, $^{3}J_{HH}$ = 15.6, 6.9, $C\underline{H}$=$CHCO_2$, 1H), 5.78 (dt, $^{3}J_{HH}$ = 15.6, $^{4}J_{HH}$ = 1.6, CH=$C\underline{H}CO_2$, 1H), 4.25 – 4.14 (m, $C\underline{H}_2OP$, 4H), 4.09 (q, $^{3}J_{HH}$ = 7.1, $CO_2C\underline{H}_2$, 2H), 3.77 (ddd, $^{3}J_{FH}$ = 37.9, $^{2}J_{HH}$ = 16.1, $^{3}J_{PH}$ = 5.7, $C\underline{H}_2C(F)P$, 1H), 3.60 (ddd, $^{2}J_{HH}$ = 15.8, $^{3}J_{FH}$ = 12.0, $^{3}J_{PH}$ = 6.5, $C\underline{H}_2C(F)P$, 1H), 2.75 – 2.67 (m, $C_{Ar(6)}C\underline{H}_2CH_2$, 2H), 2.47 (dt, $^{3}J_{HH}$ = 7.2, $C_{Ar(6)}CH_2C\underline{H}_2$, , 2H), 1.32 – 1.27 (m, $C\underline{H}_3CH_2OP$, $CO_2CH_2C\underline{H}_3$, $C(C\underline{H}_3)_3$, 15H). **^{13}C NMR** (176 MHz, $CDCl_3$) δ 166.3 (s, $\underline{C}O_2Et$, 1C), 165.0 (dd, $^{2}J_{FC}$ = 22.2, $^{2}J_{PC}$ = 3.8, $\underline{C}O_2{}^{t}Bu$, 1C), 146.8 (s, $C\underline{H}$=$CHCO_2$, 1C), 145.1 (s, $\underline{C}_{Ar(9)}$, 1C), 134.1 (s, $\underline{C}H_{Ar(2)}$, 1C), 125.9 (s, $\underline{C}H_{Ar(7)}$, 1C), 124.9 (s, $\underline{C}_{Ar(6)}$, 1C), 122.4 (s, CH=$C\underline{H}CO_2$, 1C), 121.7 (d, J_{FC} = 5.1, $\underline{C}H_{Ar(5)}$, 1C), 117.4 (s, $\underline{C}H_{Ar(8)}$, 1C), 116.7 (d, $^{3}J_{PC}$=15.0, $\underline{C}_{Ar(3)}$, 1C), 95.7 (dd, $^{1}J_{FC}$ = 199.1, $^{1}J_{PC}$ = 159.6, $\underline{C}(F)P$, 1C), 84.4 (s, $CO_2\underline{C}Me_3$, 1C), 64.6 (d, $^{2}J_{PC}$ = 6.4, $\underline{C}H_2OP$, 1C), 64.2 (d, $^{2}J_{PC}$ = 7.6, $\underline{C}H_2OP$, 1C), 60.2 (s, $CO_2\underline{C}H_2$, 1C), 33.2 (s, $C_{Ar(6)}CH_2\underline{C}H_2$, 1C), 31.3 (s, $C_{Ar(6)}\underline{C}H_2CH_2$, 1C), 28.3 (d, $^{2}J_{FC}$ = 20.8, $\underline{C}H_2C(F)P$, 1C), 27.7 (s, $C(\underline{C}H_3)_3$, 3C), 16.38 and 16.35 (2d, $^{3}J_{PC}$ = 5.3, $\underline{C}H_3CH_2OP$, 2C), 14.2 (s, $CO_2CH_2\underline{C}H_3$, 1C). **^{31}P NMR** (101 MHz, $CDCl_3$) δ 12.51 (d, $^{2}J_{PF}$ = 83.6).

(*E*)-3-(6-(5-ethoxy-5-oxopent-3-en-1-yl)imidazo[1,2-*a*]pyridin-3-yl)-2-fluoro-2-phosphonopropanoic acid (1b): obtained according to the general procedure of ester deprotection. Reaction with BTMS carried out in presence of TEA (10 eq). Scale: 102 mg of **13b**. Product **1b** was purified by preparative HPLC (gradient 1→15 min. 0→30% B, retention time 13.1 min.) followed by lyophilization. Yield: 26% (43 mg, purity 95% based on ^{31}P NMR spectrum). **^{1}H NMR (**700 MHz, D_2O pH 2**)** δ 8.58 (s, $C\underline{H}_{Ar(5)}$, 1H), 7.88 (d, $^{3}J_{HH}$ = 9.5, $C\underline{H}_{Ar(7)}$, 1H), 7.84 (s, $C\underline{H}_{Ar(2)}$, 1H), 7.84 (d, $^{3}J_{HH}$ = 9.2, $C\underline{H}_{Ar(8)}$, 1H), 7.06 (dt, $^{3}J_{HH}$ = 15.7, 7.0, $C\underline{H}$=$CHCO_2$, 1H), 5.90 (d, $^{3}J_{HH}$ = 15.8, CH=$C\underline{H}CO_2$, 1H), 4.21 (q, $^{3}J_{HH}$ = 7.2, $CO_2C\underline{H}_2$, 2H), 4.05 (ddd, $^{3}J_{FH}$ = 38.1, $^{2}J_{HH}$ = 16.3, $^{3}J_{PH}$ = 3.8, $C\underline{H}_2C(F)P$, 1H), 3.89 (ddd, $^{2}J_{HH}$ = 16.5, $^{3}J_{FH}$ = 9.7, $^{3}J_{PH}$ = 6.8, $C\underline{H}_2C(F)P$, 1H), 3.08 – 2.99 (m, $C_{Ar(6)}C\underline{H}_2CH_2$, 2H), 2.75 – 2.64 (m, $C_{Ar(6)}CH_2C\underline{H}_2$, 2H), 1.28 (t, $^{3}J_{HH}$ = 6.9, $CO_2CH_2C\underline{H}_3$, 3H). **^{13}C NMR** (176 MHz, D_2O pH 2) δ 172.0 (d, $^{2}J_{FC}$ = 21.8, $\underline{C}O_2H$, 1C), 168.9 (s, $\underline{C}O_2Et$, 1C), 149.0 (s, $C\underline{H}$=$CHCO_2$, 1C), 139.0 (s, $\underline{C}_{Ar(9)}$, 1C), 135.3 (s, $\underline{C}H_{Ar(7)}$, 1C), 130.6 (s, $\underline{C}_{Ar(6)}$, 1C), 124.7 (d, J_{FC} = 4.2, $\underline{C}H_{Ar(5)}$, 1C), 121.8 (s, CH=$C\underline{H}CO_2$, 1C), 121.3 (d, $^{3}J_{PC}$= 14.2, $\underline{C}_{Ar(3)}$, 1C), 121.1 (s, $\underline{C}H_{Ar(2)}$, 1C), 111.5 (s, $\underline{C}H_{Ar(8)}$, 1C),97.1 (dd, $^{1}J_{FC}$ = 192.4, $^{1}J_{PC}$ = 144.9, $\underline{C}(F)P$, 1C), 61.6 (s, $CO_2\underline{C}H_2$, 1C), 32.3 (s, $C_{Ar(6)}CH_2\underline{C}H_2$, 1C), 30.0 (s, $C_{Ar(6)}\underline{C}H_2CH_2$, 1C), 27.6 (d, $^{2}J_{FC}$ = 20.2, $\underline{C}H_2C(F)P$, 1C), 13.3 (s, $CO_2CH_2\underline{C}H_3$, 1C). **^{31}P NMR** (101 MHz, D_2O pH 2) 6.69 (d, $^{2}J_{PF}$ = 71.5); HR-MS: m/z [M+H^{+}] calculated 415.1065, found 415.1061.

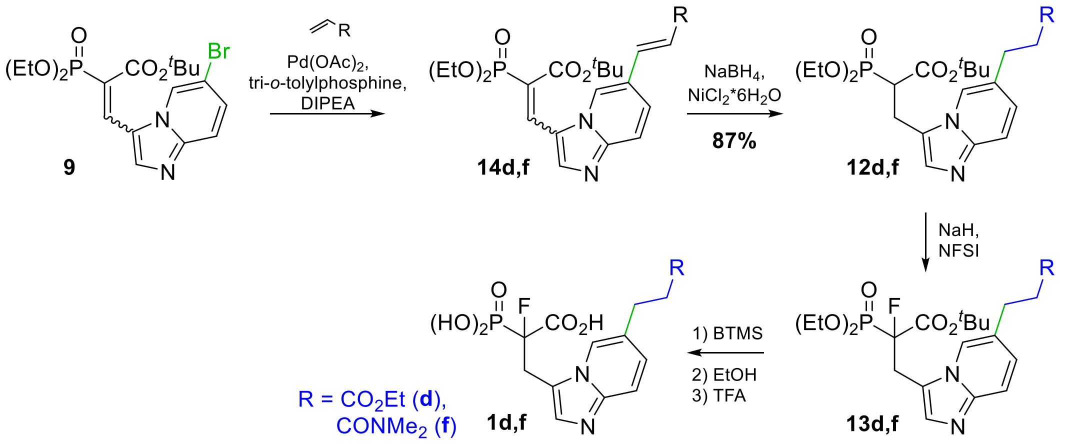

Scheme S4. Synthesis of compounds **1d,f.**

***Tert*-butyl (*E*)-2-(diethoxyphosphoryl)-3-(6-((*E*)-3-ethoxy-3-oxoprop-1-en-1-yl)imidazo[1,2-*a*]pyridin-3-yl)acrylate (14d):** obtained according to the Mizoroki–Heck reaction procedure for compound **12a**, using ethyl acrylate. Scale: 0.22 mmol of **7**. Compound **14d** was purified by flash chromatography using Gilson PLC 2250 purification system. Eluent A: DCM and B: Acetone, to both of them TEA was added, 1mL/(1L of eluent) (gradient 5→45 min. 0→50%B, retention time 35 min.). Yield: 74% (78 mg). **^{1}H NMR** (700 MHz, $CDCl_3$) δ 8.44 (s, $CH_{Ar(2)}$, 1H), 8.49 (s, $CH_{Ar(5)}$, 1H), 7.83 (d, $^3J_{PH}$ = 24.0, PCCH, 1H), 7.67 (d, $^3J_{HH}$ = 9.4, $CH_{Ar(8)}$, 1H), 7.66 (d, $^3J_{HH}$ = 15.9, CH=CHCO_2Et, 1H), 7.55 (dd, $^3J_{HH}$ = 9.4, $^4J_{HH}$ = 1.6, $CH_{Ar(7)}$, 1H), 6.46 (d, $^3J_{HH}$ = 15.9, CH=CHCON, 1H), 4.25 (q, $^3J_{HH}$ = 7.1, CO_2CH_2, 2H), 4.21 – 4.08 (m, CH_2OP, 4H), 1.55 (s, $C(CH_3)_3$, 9H), 1.35 (t, $^3J_{HH}$ = 7.1, CH_3CH_2OP, 6H), 1.32 (t, $^3J_{HH}$ = 7.1, $CO_2CH_2CH_3$, 3H). **^{13}C NMR** (176 MHz, $CDCl_3$) δ 166.10 (s, CO_2Et, 1C), 164.85 (d, $^2J_{PC}$ = 11.0, $CO_2{}^t$Bu, 1C), 147.26 (s, $C_{Ar(9)}$, 1C), 141.00 (s, $CH_{Ar(2)}$, 1C), 139.63 (s, CH=CHCO_2, 1C), 131.80 (d, $^2J_{PC}$ = 10.1, PCCH, 1C), 125.11 (s, $CH_{Ar(5)}$, 1C), 124.42 (s, $CH_{Ar(7)}$, 1C), 122.52 (s, $C_{Ar(6)}$, 1C), 121.05 (d, $^3J_{PC}$ = 24.3, $C_{Ar(3)}$, 1C), 120.18 (s, CH=CHCO_2, 1C), 119,57 (d, $^1J_{PC}$ = 181.5, PC, 1C), 118.64 (s, $CH_{Ar(8)}$, 1C), 82.83 (s, CMe_3, 1C), 62.57 (d, $^2J_{PC}$ = 5.2, CH_2OP, 2C), 60.81 (s, COCH_2, 1C), 28.07 (s, $C(CH_3)_3$, 3C), 16.34 (d, $^3J_{PC}$ = 6.5, CH_3CH_2OP, 2C), 14.30 (s, $CO_2CH_2CH_3$, 1C). **^{31}P NMR** (101 MHz, $CDCl_3$) δ 16.26. HRMS [$C_{23}H_{31}N_2O_7P+H^+$] m/z: calculated 479.1942, found 479.1953.

***Tert*-butyl (*E*)-2-(diethoxyphosphoryl)-3-(6-((*E*)-3-(dimethylamino)-3-oxoprop-1-en-1-yl)imidazo[1,2-*a*]pyridin-3-yl)acrylate (14f):** obtained according to the Mizoroki–Heck reaction procedure for compound **12a**, using *N,N*-dimethyloacrylamide. Scale: 0.22 mmol of **7**. Compound **14f** was purified by flash chromatography using Gilson PLC 2250 purification system. Eluents A: DCM and B: Acetone, to both of them TEA was added, 1mL/(1L of eluent) (gradient 10→45 min. 0→40%B, retention time 35 min.). Yield: 49% (83 mg). **^{1}H NMR** (700 MHz, $CDCl_3$) δ 8.44 (s, $CH_{Ar(2)}$, 1H), 8.43 (s, $CH_{Ar(5)}$, 1H), 7.82 (d, $^3J_{PH}$ = 23.8, PCCH, 1H), 7.69 (d, $^3J_{HH}$ = 9.3, $CH_{Ar(8)}$, 1H), 7.66 (d, $^3J_{HH}$ = 15.5, CH=CHCON, 1H), 7.56 (dd, $^3J_{HH}$ = 9.2, $^4J_{HH}$ = 1.6, $CH_{Ar(7)}$, 1H), 6.95 (d, $^3J_{HH}$ = 15.3, CH=CHCON, 1H), 4.24 – 4.13 (m, CH_2OP, 4H), 3.21 (s, CH_3, 3H), 3.09 (s, CH_3, 3H), 1.58 (s, $C(CH_3)_3$, 9H), 1.38 (2t, $^3J_{HH}$ = 7.1, CH_3CH_2OP, 3H), 1.38 (t, $^3J_{HH}$ = 7.1, CH_3CH_2OP, 3H). **^{13}C NMR** (176 MHz, $CDCl_3$ 77.16) δ 165.71 (s, CON, 2C), 164.67 (d, $^2J_{PC}$ = 11.3, CO_2tBu, 1C), 146.93 (s, $C_{Ar(9)}$, 1C), 140.60 (s, $CH_{Ar(2)}$, 1C), 137.30 (s, CH=CHCO_2, 1C), 131.82 (d, $^2J_{PC}$ = 10.2, PCCH, 1C), 125.15 (s, $CH_{Ar(7)}$, 1C), 124.39 (s, $CH_{Ar(5)}$, 1C), 123.11 (s, $C_{Ar(6)}$, 1C), 120.77 (d, $^3J_{PC}$ = 25.3, $C_{Ar(3)}$, 1C), 119.07 (s, CH=CHCO_2, 1C), 118.66 (d, $^1J_{PC}$ = 188.1, PC, 1C), 117.94 (s, $CH_{Ar(8)}$, 1C), 82.55 (s, CO_2CMe_3, 1C), 62.36 (d, $^2J_{PC}$ = 5.1, CH_2OP, 2C), 37.34 (s, CH_3, 1C), 35.78 (s, CH_3, 1C), 27.82 (s,

$C(\underline{C}H_3)_3$, 3C), 16.10 (d, $^3J_{PC}$ = 7.4, $\underline{C}H_3CH_2OP$, 2C). **^{31}P NMR** (101 MHz, $CDCl_3$) δ 16.07. HRMS ($C_{23}H_{32}N_3O_6P + H^+$) m/z: calculated 478.2101, found 478.2107.

***Tert*-butyl 2-(diethoxyphosphoryl)-3-(6-(3-ethoxy-3-oxopropyl)imidazo[1,2-*a*]pyridin-3-yl)propanoate (12d):** obtained according to the reduction procedure for compound **11**. Scale: 100 mg of **14d**. Compound **12d** was purified by flash chromatography using Gilson PLC 2250 purification system. Eluents A: DCM and B: Acetone, to both of them TEA was added, 1mL/(1L of eluent) (gradient 5→35 min. 0→35%B, retention time 31 min.). Yield: 61% (85 mg, purity 93% according to the ^{31}P NMR spectrum). **^{1}H NMR** (700 MHz, $CDCl_3$) δ 7.86 (s, $C\underline{H}_{Ar(5)}$, 1H), 7.46 (dd, $^3J_{HH}$ = 9.2, $^4J_{HH}$ = 1.0, $C\underline{H}_{Ar(8)}$, 1H), 7.40 (s, $C\underline{H}_{Ar(2)}$, 1H), 7.06 (dd, $^3J_{HH}$ = 9.2, $^4J_{HH}$ = 1.7, $C\underline{H}_{Ar(7)}$, 1H), 4.24 – 4.19 (m, $C\underline{H}_2OP$, 4H), 4.14 (t, $^3J_{HH}$ = 7.1, $CO_2C\underline{H}_2$, 2H), 3.53 (ddd, $^2J_{HH}$ = 16.4, $^3J_{HH}$ = 11.9, $^3J_{PH}$ = 6.6, $C\underline{H}_2C(H)P$, 1H), 3.32 – 3.23 (m, $C\underline{H}_2C(H)P$, $CH_2C(\underline{H})P$, 2H), 2.96 (t, $^3J_{HH}$ = 7.8, $C_{Ar(5)}C\underline{H}_2$, 2H), 2.65 (t, $^3J_{HH}$ = 7.6, $C\underline{H}_2CO_2$, 2H), 1.38 (s, $C(C\underline{H}_3)_3$, 9H), 1.40 – 1.35 (m, $C\underline{H}_3CH_2OP$, 6H), 1.24 (t, $^3J_{HH}$ = 7.1, $CO_2CH_2C\underline{H}_3$, 3H). **^{13}C NMR** (176 MHz, $CDCl_3$ 77.16) δ 172.34 (s, $\underline{C}O_2Et$, 1C), 167.24 (d, $^2J_{PC}$ = 5.1, $\underline{C}O_2t$Bu, 1C), 144.69 (s, $\underline{C}_{Ar(9)}$, 1C), 131.39 (s, $\underline{C}H_{Ar(2)}$, 1C), 125.72 (s, $\underline{C}_{Ar(6)}$, 1C), 124.96 (s, $\underline{CH}_{Ar(7)}$, 1C), 121.30 (d, $^3J_{PC}$ = 19.5, $\underline{C}_{Ar(3)}$, 1C), 121.17 (s, $\underline{C}H_{Ar(5)}$, 1C), 117.56 (s, $\underline{C}H_{Ar(8)}$, 1C), 82.59 (s, $CO_2\underline{C}Me_3$, 1C), 63.09 (d, $^2J_{PC}$ = 6.3, $\underline{C}H_2OP$, 1C), 62.98 (d, $^2J_{PC}$ = 6.9, $\underline{C}H_2OP$, 1C), 60.65 (s, $CO_2\underline{C}H_2$, 1C), 44.98 (d, $^2J_{PC}$ = 129.0, $CH_2\underline{C}(H)P$, 1C), 35.33 (s, $\underline{C}H_2CO_2Et$, 1C), 27.95 (s, $C_{Ar(6)}\underline{C}H_2$, 1C), 27.79 (s, $C(\underline{C}H_3)_3$, 3C), 21.63 (d, $^3J_{PC}$ = 3.6, $\underline{C}H_2C(H)P$, 1C), 16.45 (d, $^3J_{PC}$ = 5.1, $\underline{C}H_3CH_2OP$, 1C), 16.44 (d, $^3J_{PC}$ = 5.2, $\underline{C}H_3CH_2OP$, 1C), 14.20 (s, $CO_2CH_2\underline{C}H_3$, 1C). **^{31}P NMR** (101 MHz, $CDCl_3$) δ 23.30.

***Tert*-butyl 2-(diethoxyphosphoryl)-3-(6-(3-(dimethylamino)-3-oxopropyl)imidazo[1,2-*a*]pyridin-3-yl)propanoate (12f):** two-step reduction of carbon double bonds was carried out. The first stage involved the reduction of double bond *HC=CH*$CONMe_2$. This reaction was carried out in a single-neck flask equipped with two-way stopcock, which enabled degassing the system (vacuum– hydrogen, three times). In a singleneck flask compound **14f** (160 mg) and 10% Pd/C (10 mg) was placed in EtOH (10 mL). The system was degassed using a two-way stopcock. This suspension was stirred with H_2 overnigth at room temperature. The catalyst was then filtered off through a thin layer of Celite500, and the filtrate was evaporated to dryness. Thus obtained compound was used in the second step which involved reduction of doube bond *HC=C*-CO_2tBu using the procedure with $NaBH_4$ previously described for compound **11**. Compound **12f** was purified by flash chromatography using Gilson PLC 2250 purification system. Eluents A: DCM and B: Acetone, to both of them TEA was added, 1mL/(1L of eluent) (gradient 0→23 min. 0→30%B, retention time 45 min.). Yield: 62% (100 mg, purity 88% according to the ^{31}P NMR spectrum). **^{1}H NMR** (700 MHz, $CDCl_3$) δ 7.87 (s, $C\underline{H}_{Ar(5)}$, 1H), 7.51 (d, $^3J_{HH}$ = 9.3, $C\underline{H}_{Ar(8)}$, 1H), 7.35 (s, $C\underline{H}_{Ar(2)}$, 1H), 7.08 (dd, $^3J_{HH}$ = 9.2, $^4J_{HH}$ = 1.6, $C\underline{H}_{Ar(7)}$, 1H), 4.20 – 4.11 (m, $C\underline{H}_2OP$, 4H), 3.49 (ddd, $^2J_{HH}$ = 16.2, $^3J_{HH}$ = 11.8, $^3J_{PH}$ = 6.6, $C\underline{H}_2C(H)P$, 1H), 3.29 – 3.23 (m, $C\underline{H}_2C(\underline{H})P$, 2H), 2.95 (t, $^3J_{HH}$ = 7.5, $C_{Ar(5)}C\underline{H}_2$, 2H), 2.93 (s, $NC\underline{H}_3$, 3H), 2.90 (s, $NC\underline{H}_3$, 3H), 2.61 (t, $^3J_{HH}$ = 7.6, $C\underline{H}_2CO_2$, 2H), 1.33 (s, $C(C\underline{H}_3)_3$, 9H), 1.32 (t, $^3J_{HH}$ = 7.1, $C\underline{H}_3CH_2OP$, 6H). **^{13}C NMR** (176 MHz, $CDCl_3$ 77.16) δ 171.38 (s, $\underline{C}ON$, 1C), 167.25 (d, $^2J_{PC}$ = 5.1, $\underline{C}O_2t$Bu, 1C), 144.39 (s, $\underline{C}_{Ar(9)}$, 1C), 130.67 (s, $\underline{C}H_{Ar(2)}$, 1C), 126.55 (s, $\underline{C}_{Ar(6)}$, 1C), 126.18 (s, $\underline{CH}_{Ar(7)}$, 1C), 121.36 (d, $^3J_{PC}$ = 19.3, $\underline{C}_{Ar(3)}$, 1C), 121.34 (s, $\underline{C}H_{Ar(5)}$, 1C), 117.24 (s, $\underline{C}H_{Ar(8)}$, 1C), 82.60 (s, $CO_2\underline{C}Me_3$, 1C), 63.09 (d, $^2J_{PC}$ = 6.4, $\underline{C}H_2OP$, 1C), 62.97 (d, $^2J_{PC}$ = 6.7, $\underline{C}H_2OP$, 1C), 44.99 (d, $^2J_{PC}$ = 129.4, $CH_2\underline{C}(H)P$, 1C), 37.16 (s, $N\underline{C}H_3$, 1C), 35.51 (s, $N\underline{C}H_3$, 1C), 34.39 (s, $\underline{C}H_2CON$, 1C), 28.13 (s, $C_{Ar(6)}\underline{C}H_2$, 1C), 27.83 (s, $C(\underline{C}H_3)_3$, 3C), 21.66 (d, $^3J_{PC}$ = 3.6, $\underline{C}H_2C(H)P$, 1C), 16.46 (d, $^3J_{PC}$ = 3.7, $\underline{C}H_3CH_2OP$, 1C), 16.43 (d, $^3J_{PC}$ = 4.0, $\underline{C}H_3CH_2OP$, 1C). **^{31}P NMR** (101 MHz, $CDCl_3$) δ 21.69.

***Tert*-butyl 2-(diethoxyphosphoryl)-3-(6-(3-ethoxy-3-oxopropyl)imidazo[1,2-*a*]pyridin-3-yl)-2-fluoropropanoate (13d):** obtained according to the general procedure of fluorination. Scale: 85 mg

(0.18 mmol) of **12d**. Compound **13d** was purified by flash chromatography using Gilson PLC 2250 purification system. Eluents A:DCM, B:Acetone, to both of them TEA was added, 1mL/(1L of eluent) (gradient 5→30 min. 0→30% B, retention time 16 min.). Yield: 84%. ^{1}**H NMR** (700 MHz, $CDCl_3$) δ 7.93 (s, $C\underline{H}_{Ar(5)}$, 1H), 7.49 (d, $^{3}J_{HH}$ = 9.2, $C\underline{H}_{Ar(8)}$, 1H), 7.45 (s, $C\underline{H}_{Ar(2)}$, 1H), 7.03 (dd, $^{3}J_{HH}$ = 9.2, $^{4}J_{HH}$=1.6, $C\underline{H}_{Ar(7)}$, 1H), 4.29 – 4.19 (m, $C\underline{H}_2OP$, 4H), 4.10 (q, $^{3}J_{HH}$ = 7.1, $CO_2C\underline{H}_2$, 2H), 3.81 (ddd, $^{3}J_{FH}$ = 37.8, $^{2}J_{HH}$ = 16.1, $^{3}J_{PH}$ = 5.6, $C\underline{H}_2C(F)P$, 1H), 3.63 (ddd, $^{2}J_{HH}$ = 16.0, $^{3}J_{FH}$ = 11.9, $^{3}J_{PH}$ = 6.7, $C\underline{H}_2C(F)P$, 1H), 2.92 (d, $^{3}J_{HH}$ = 7.6, $C\underline{H}_2CH_2CO_2$, 2H), 2.61 (t, $^{3}J_{HH}$ = 7.6, $C\underline{H}_2CO_2$, 2H), 1.37 – 1.32 (m, $C\underline{H}_3CH_2OP$, $C(C\underline{H}_3)_3$, 15H), 1.20 (t, $^{3}J_{HH}$ = 7.2, CO_2 $CH_2C\underline{H}_3$, 3H). ^{13}**C NMR** (176 MHz, $CDCl_3$ 77.16) δ 172.4 (s, $\underline{C}O_2Et$, 1C), 165.1 (dd, $^{2}J_{FC}$ = 22.2, $^{2}J_{PC}$ = 3.8, $\underline{C}O_2t$Bu, 1C), 145.3 (s, $\underline{C}_{Ar(9)}$, 1C), 134.1 (s, $\underline{C}H_{Ar(2)}$, 1C), 126.0 (s, $\underline{C}H_{Ar(7)}$, 1C), 124.9 (s, $\underline{C}_{Ar(6)}$, 1C), 122.0 (d, J_{FC} = 5.0, $\underline{C}H_{Ar(5)}$, 1C), 117.5 (s, $\underline{C}H_{Ar(8)}$, 1C), 116.9 (d, $^{3}J_{PC}$ = 14.8, $\underline{C}_{Ar(3)}$, 1C), 95.9 (dd, $^{1}J_{FC}$ = 199.6, $^{1}J_{PC}$ = 159.6, $\underline{C}(F)P$, 1C), 84.5 (s, $CO_2\underline{C}Me_3$, 1C), 64.6 (d, $^{2}J_{PC}$ = 6.5, $\underline{C}H_2OP$, 1C), 64.4 (d, $^{2}J_{PC}$ = 7.1, $\underline{C}H_2OP$, 1C), 60.7 (s, $\underline{C}O_2Et$, 1C), 35.5 (s, $\underline{C}H_2CO_2$, 1C), 28.4 (dd, $^{2}J_{FC}$ = 20.8, $^{2}J_{PC}$ = 2.1, $\underline{C}H_2C(F)P$, 1C), 28.1 (s, $\underline{C}H_2CH_2CO_2$, 1C), 27.8 (s, $C(\underline{C}H_3)_3$, 3C), 16.52 (d, $^{3}J_{PC}$ = 5.5, $\underline{C}H_3CH_2OP$, 1C), 16.48 (d, $^{3}J_{PC}$ = 5.5, $\underline{C}H_3CH_2OP$, 1C), 14.3 (s, $CO_2CH_2\underline{C}H_3$, 1C). ^{31}**P NMR** (283 MHz, $CDCl_3$) δ 12.88 (d, $^{2}J_{PF}$ = 83.2).

***Tert*-butyl 2-(diethoxyphosphoryl)-3-(6-(3-(dimethylamino)-3-oxopropyl)imidazo[1,2-*a*]pyridin-3-yl)-2-fluoropropanoate (13f):** obtained according to the general procedure of fluorination. Scale: 100 mg (0.21 mmol) of **12f**. Compound **13f** was purified by flash chromatography using Gilson PLC 2250 purification system. Eluents A:DCM, B:Acetone, to both of them TEA was added, 1mL/(1L of eluent) (gradient 0→15 min. 0→30% B, retention time 43 min.). Yield: 63% (77 mg). ^{1}**H NMR** (700 MHz, $CDCl_3$) δ 7.93 (s, $C\underline{H}_{Ar(5)}$, 1H), 7.46 (d, $^{3}J_{HH}$ = 9.2, $C\underline{H}_{Ar}$, 1H), 7.42 (s, $C\underline{H}_{Ar(2)}$, 1H), 7.05 (dd, $^{3}J_{HH}$ = 9.2, $^{4}J_{HH}$=1.4, $C\underline{H}_{Ar}$, 1H), 4.26 – 4.17 (m, $C\underline{H}_2OP$, 4H), 3.79 (ddd, $^{3}J_{FH}$ = 38.0, $^{2}J_{HH}$ = 16.1, $^{3}J_{PH}$ = 5.3, $C\underline{H}_2C(F)P$, 1H), 3.62 (ddd, $^{2}J_{HH}$ = 16.3, $^{3}J_{FH}$ = 11.8, $^{3}J_{PH}$ = 6.7, $C\underline{H}_2C(F)P$, 1H), 2.92 (d, $^{3}J_{HH}$ = 7.4, $C\underline{H}_2CH_2C(O)N$, 2H), 2.91 (s, $C\underline{H}_3$, 3H), 2.89 (s, $C\underline{H}_3$, 3H), 2.58 (t, $^{3}J_{HH}$ = 7.6, $C\underline{H}_2C(O)N$, 2H), 1.34 – 1.31 (m, $C\underline{H}_3CH_2OP$, $C(C\underline{H}_3)_3$, 15H). ^{13}**C NMR** (176 MHz, $CDCl_3$) δ 171.5 (s, $\underline{C}ON$, 1C), 165.0 (dd, $^{2}J_{FC}$ = 22.6, $^{2}J_{PC}$ = 3.8, $\underline{C}O_2t$Bu, 1C), 145.0 (s, $\underline{C}_{Ar(9)}$, 1C), 133.6 (s, $\underline{C}H_{Ar(2)}$, 1C), 126.6 (s, $\underline{C}H_{Ar}$, 1C), 125.8 (s, $\underline{C}_{Ar(6)}$, 1C), 122.0 (d, J_{FC} = 4.5, $\underline{C}H_{Ar(5)}$, 1C), 117.2 (s, $\underline{C}H_{Ar}$, 1C), 116.8 (d, $^{3}J_{PC}$ = 15.0, $\underline{C}_{Ar(3)}$, 1C), 95.8 (dd, $^{1}J_{FC}$ = 199.3, $^{1}J_{PC}$ = 159.6, $\underline{C}(F)P$, 1C), 84.5 (s, $CO_2\underline{C}Me_3$, 1C), 64.6 (d, $^{2}J_{PC}$ = 7.1, $\underline{C}H_2OP$, 1C), 64.3 (d, $^{2}J_{PC}$ = 7.2, $\underline{C}H_2OP$, 1C), 37.3 – 37.0 (m, CH_3, 1C), 35.6 – 35.3 (m, CH_3, 1C), 34.5 (s, $\underline{C}H_2C(O)N$, 1C), 28.3 (d, $^{2}J_{FC}$ = 23.1, $\underline{C}H_2C(F)P$, 1C), 28.2 (s, $\underline{C}H_2CH_2C(O)N$, 1C), 27.8 (s, $C(\underline{C}H_3)_3$, 3C), 16.5 (d, $^{3}J_{PC}$ = 5.0, $\underline{C}H_3CH_2OP$, 1C), 16.4 (d, $^{3}J_{PC}$ = 4.6, $\underline{C}H_3CH_2OP$, 1C). ^{31}**P NMR** (283 MHz, $CDCl_3$) δ 12.86 (d, $^{2}J_{PF}$ = 83.3).

3-(6-(3-ethoxy-3-oxopropyl)imidazo[1,2-*a*]pyridin-3-yl)-2-fluoro-2-phosphonopropanoic acid (1d): obtained according to the general procedure of ester deprotection. Scale: 43 mg of **13d**. Product **1d** was purified by preparative HPLC (gradient 3→30 min. 0→30% B, retention time 12.1 min.) followed by lyophilization. Yield: 34% (12 mg). ^{1}**H NMR** (700 MHz, D_2O pH 2) δ 8.62 (s, $C\underline{H}_{Ar(5)}$, 1H), 7.91 (dd, $^{3}J_{HH}$ = 9.3, $^{4}J_{HH}$=1.4, $C\underline{H}_{Ar(7)}$, 1H), 7.86 (d, $^{3}J_{HH}$ = 9.3, $C\underline{H}_{Ar(8)}$, 1H), 7.83 (s, $C\underline{H}_{Ar(2)}$, 1H), 4.18 (q, $^{3}J_{HH}$ = 7.2, $CO_2C\underline{H}_2$, 2H), 4.05 (ddd, $^{3}J_{FH}$ = 38.8, $^{2}J_{HH}$ = 16.5, $^{3}J_{PH}$ = 2.2, $C\underline{H}_2C(F)P$, 1H), 3.87 (ddd, $^{2}J_{HH}$ = 15.8, $^{3}J_{FH}$ = 7.9, $^{3}J_{PH}$ = 7.9, $C\underline{H}_2C(F)P$, 1H), 3.16 (t, $^{3}J_{HH}$ = 7.4, $C\underline{H}_2CH_2CO$, 2H), 2.87 (t, $^{3}J_{HH}$ = 7.5, $C\underline{H}_2CO$, 2H), 1.23 (t, $^{3}J_{HH}$ = 7.2, $CO_2CH_2C\underline{H}_3$, 3H. ^{13}**C NMR** (176 MHz, D_2O pH 2) δ 175.1 (s, $\underline{C}O_2Et$, 1C), 172.3 (d, $^{2}J_{FC}$ = 24.3, $\underline{C}O_2H$, 1C), 139.1 (s, $\underline{C}_{Ar(9)}$, 1C), 135.1 (s, $\underline{C}H_{Ar(7)}$, 1C), 130.2 (s, $\underline{C}_{Ar(6)}$, 1C), 124.8 (d, J_{FC} = 4.4, $\underline{C}H_{Ar(5)}$, 1C), 121.5 (d, $^{3}J_{PC}$ = 14.0, $\underline{C}_{Ar(3)}$, 1C), 121.2 (s, $\underline{C}H_{Ar(2)}$, 1C), 111.7 (s, $\underline{C}H_{Ar(8)}$, 1C), 97 (m, $\underline{C}(F)P$, 1C, in HMBC spectrum), 61.9 (s, $CO_2\underline{C}H_2$, 1C), 34.7 (s, $\underline{C}H_2CO$, 1C), 27.7 (d, $^{2}J_{FC}$ = 20.7, $^{2}J_{PC}$ = 2.5, $\underline{C}H_2C(F)P$, 1C), 27.0 (s, $\underline{C}H_2CH_2CO$, 1C), 13.3 (s, $CO_2CH_2\underline{C}H_3$, 1C). ^{31}**P NMR** (101 MHz, D_2O pH 2) δ 6.99 (d, $^{2}J_{PF}$ = 71.6). HR-MS: m/z [$M+H^+$] calculated 389.0908, found 389.0907. Elemental analysis: $C_{15}H_{18}FN_2O_7P$ *$0.45H_2O$*$0.5CF_3CO_2H$: calculated C42.43; H4.33; N6.09; found C42.38; H4.31; N6.18; max. diff. 0.09.

3-(6-(3-(dimethylamino)-3-oxopropyl)imidazo[1,2-*a*]pyridin-3-yl)-2-fluoro-2-phosphonopropanoic acid (1f): obtained according to the general procedure of ester deprotection. Scale: 70 mg of **13f**. Product **1f** was purified by preparative HPLC (gradient 3→20 min. 0→30% B, retention time 9.2 min.) followed by lyophilization. Yield: 61% (33 mg). In the ^{13}C NMR spectrum of **1f** signals from $\underline{C}F_3\underline{C}O_2^-$ are present. **^{1}H NMR** (700 MHz, D_2O pH 3) δ 8.59 (s, C$\underline{H}_{Ar(5)}$, 1H), 7.88 (dd, $^3J_{HH}$ = 9.3, $^4J_{HH}$=1.1, C$\underline{H}_{Ar(7)}$, 1H), 7.84 (d, $^3J_{HH}$ = 8.9, C$\underline{H}_{Ar(8)}$, 1H), 7.83 (s, C$\underline{H}_{Ar(2)}$, 1H), 4.04 (ddd, $^3J_{FH}$ = 38.2, $^2J_{HH}$ = 16.5, $^3J_{PH}$ = 3.5, C$\underline{H}_2$C(F)P, 1H), 3.88 (ddd, $^2J_{HH}$ = 16.4, $^3J_{FH}$ = 9.5, $^3J_{PH}$ = 7.0, C$\underline{H}_2$C(F)P, 1H), 3.12 (t, $^3J_{HH}$ = 7.9, C$\underline{H}_2$CH$_2$CO, 2H), 3.07 (s, C$\underline{H}_3$, 3H), 2.93 (s, C$\underline{H}_3$, 3H), 2.89 (td, $^3J_{HH}$ = 7.4, J_{HH} =2.2, C$\underline{H}_2$CO, 2H). **^{13}C NMR** (176 MHz, D_2O pH 3) δ 174.2 (s, $\underline{C}$ON, 1C), 172.0 (dd, $^2J_{FC}$ = 22.9, $^2J_{PC}$ = 5.2, $\underline{C}O_2H$, 1C), 139.1 (s, $\underline{C}_{Ar(9)}$, 1C), 135.2 (s, $\underline{C}H_{Ar(7)}$, 1C), 130.6 (s, $\underline{C}_{Ar(6)}$, 1C), 124.6 (d, J_{FC} = 4.3, $\underline{C}H_{Ar(5)}$, 1C), 121.3 (d, $^3J_{PC}$ = 14.0, $\underline{C}_{Ar(3)}$, 1C), 121.2 (s, $\underline{C}H_{Ar(2)}$, 1C), 111.6 (s, $\underline{C}H_{Ar(8)}$, 1C), 97.1 (dd, $^1J_{FC}$ = 193.0, $^1J_{PC}$ = 142.4, $\underline{C}$(F)P, 1C), 37.5 (s, $\underline{C}H_3$, 1C), 35.4 (s, $\underline{C}H_3$, 1C), 33.3 (s, $\underline{C}H_2$CO, 1C), 27.6 (d, $^2J_{FC}$ = 20.2, $\underline{C}H_2$C(F)P, 1C), 27.5 (s, $\underline{C}H_2$CH$_2$CO, 1C). **^{31}P NMR** (283 MHz, D_2O pH 3) δ 9.02 (d, $^2J_{PF}$ = 73.1). HR-MS: m/z [M+H$^+$] calculated 388.1068, found 388.1069.

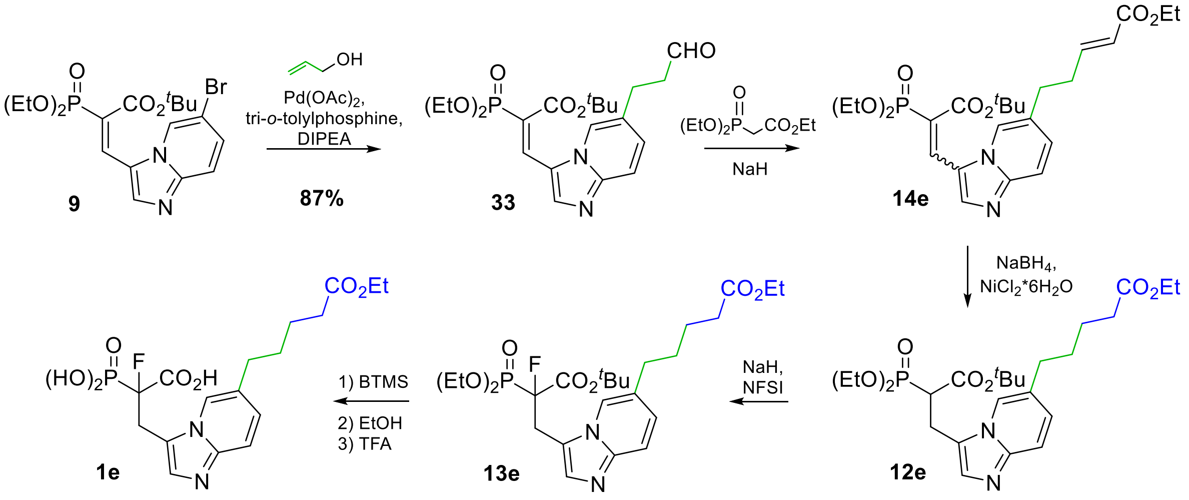

Scheme S5. Synthesis of compound **1e.**

***Tert*-butyl (*E*)-2-(diethoxyphosphoryl)-3-(6-(3-oxopropyl)imidazo[1,2-*a*]pyridin-3-yl)acrylate (33):** obtained according to the Mizoroki–Heck reaction procedure for compound **12a**, using allyl alcohole. Scale: 0.22 mmol of **9**. Compound **33** was purified by flash chromatography using Gilson PLC 2250 purification system. Eluents A: DCM and B: Acetone (gradient 0→45 min. 10→40%B, retention time 33 min.). Yield: 67% (92% purity according to ^{31}P NMR spectrum). **^{1}H NMR** (700 MHz, CDCl$_3$) δ 9.84 (t, $^3J_{HH}$ = 1.0, C$\underline{H}$O, 1H), 8.45 (s, C$\underline{H}_{Ar(2)}$, 1H), 8.19 (s, C$\underline{H}_{Ar(5)}$, 1H), 7.84 (d, $^3J_{PH}$ = 24.1, PCC$\underline{H}$, 1H), 7.63 (d, $^3J_{HH}$ = 9.2, C$\underline{H}_{Ar(8)}$, 1H), 7.24 (dd, $^3J_{HH}$ = 9.2, $^4J_{HH}$ = 1.7, C$\underline{H}_{Ar(7)}$, 1H), 4.23 – 4.12 (m, POC$\underline{H}_2$, 4H), 3.01 (t, $^3J_{HH}$ = 7.3, C$_{Ar(6)}$C$\underline{H}_2$, 2H), 2.87 (t, $^3J_{HH}$ = 7.3, C$\underline{H}_2$CHO, 2H), 1.58 (s, C(C$\underline{H}_3$)$_3$, 9H), 1.37 (t, $^3J_{HH}$ = 7.3, POCH$_2$C$\underline{H}_3$, 6H). **^{13}C NMR** (176 MHz, CDCl$_3$ 77.16) δ 200.17 (s, $\underline{C}$HO, 1C), 164.99 (d, $^2J_{PC}$ = 11.4, $\underline{C}O_2{}^t$Bu, 1C), 146.68 (s, $\underline{C}_{Ar(9)}$, 1C), 140.60 (s, $\underline{C}H_{Ar(2)}$, 1C), 132.38 (d, $^2J_{PC}$ = 10.3, PC$\underline{C}$H, 1C), 128.95 (s, $\underline{C}H_{Ar(7)}$, 1C), 127.07 (s, $\underline{C}_{Ar(6)}$, 1C), 122.04 (s, $\underline{C}H_{Ar(5)}$, 1C), 120.26 (d, $^3J_{PC}$ = 24.8, $\underline{C}_{Ar(3)}$, 1C), 118.05 (s, $\underline{C}_{Ar(8)}$, 1C), 117.59 (d, $^1J_{PC}$ = 182.6, P$\underline{C}$, 1C), 82.59 (s, CO$_2\underline{C}$Me$_3$, 1C), 62.43 (d, $^2J_{PC}$ = 5.3, $\underline{C}H_2$OP, 1C), 44.59 (s, C$_{Ar(6)}$CH$_2\underline{C}$H$_2$, 1C), 28.04 (s, C($\underline{C}$H$_3$)$_3$, 3C), 24.97 (s, C$_{Ar(6)}\underline{C}$H$_2$, 1C), 16.29 (d, $^3J_{PC}$ = 6.9, $\underline{C}$H$_3$CH$_2$OP, 2C), 14.20 (s, CO$_2$CH$_2\underline{C}$H$_3$, 1C). **^{31}P NMR** (101 MHz, CDCl$_3$) δ 16.87. HRMS ($C_{21}H_{29}N_2O_6P$ + H$^+$) m/z: calculated 437.1836, found 437.1840.

Ethyl (2*E*)-5-(3-(3-(*tert*-butoxy)-2-(diethoxyphosphoryl)-3-oxoprop-1-en-1-yl)imidazo[1,2-*a*]pyridin-6-yl)pent-2-enoate (14e): obtained according to the Horner–Wadsworth–Emmons reaction procedure for compound **12b**. Scale: 0.36 mmol (155 mg) of **33**. Compound **14e** was purified by flash chromatography using Gilson PLC 2250 purification system. Eluents A: DCM and B: Acetone, to both of them TEA was added, 1mL/(1L of eluent) (gradient 5→30 min. 0→30%B, retention time 12 min.). Yield: 53% (95 mg, 73% purity according to ^{31}P NMR spectrum). ^{1}**H NMR** (700 MHz, $CDCl_3$) δ 8.39 (s, $C\underline{H}_{Ar(2)}$, 1H), 8.11 (s, $C\underline{H}_{Ar(5)}$, 1H), 7.78 (d, $^{3}J_{PH}$ = 24.0, PCC$\underline{H}$, 1H), 7.56 (dd, $^{3}J_{HH}$ = 9.3, $C\underline{H}_{Ar(8)}$, 1H), 7.16 (dd, $^{3}J_{HH}$ = 9.1, $^{4}J_{HH}$ = 1.6, $C\underline{H}_{Ar(7)}$, 1H), 6.87 (dt, $^{3}J_{HH}$ = 15.6, $^{3}J_{HH}$ = 6.9, $C\underline{H}CHCO_2$, 1H), 5.78 (d, $^{3}J_{HH}$ = 15.6, $CHC\underline{H}CO_2$, 1H), 4.15 – 4.03 (m, $POC\underline{H}_2$, CO_2CH_2, 6H), 2.76 (t, $^{3}J_{HH}$ = 7.7, $C_{Ar(6)}C\underline{H}_2CH_2$, 2H), 2.53 – 2.47 (m, $C\underline{H}_2CO_2$, 2H), 1.50 (s, $C(C\underline{H}_3)_3$, 9H), 1.30 (t, $^{3}J_{HH}$ = 7.1, $POCH_2C\underline{H}_3$, 6H), 1.20 (t, $^{3}J_{HH}$ = 7.1, $CO_2CH_2C\underline{H}_3$, 4H). ^{13}**C NMR** (176 MHz, $CDCl_3$) δ 166.17 (s, $\underline{C}O_2Et$, 1C), 165.03 (d, $^{2}J_{PC}$ = 11.3, $\underline{C}O_2{}^{t}Bu$, 1C), 146.79 (s, $\underline{C}_{Ar(9)}$, 1C), 146.29 (s, $\underline{C}HCHCO_2$, 1C), 140.72 (s, $\underline{C}H_{Ar(2)}$, 1C), 132.39 (d, $^{2}J_{PC}$ = 10.2, PC$\underline{C}$H, 1C), 128.74 (s, $\underline{C}H_{Ar(7)}$, 1C), 127.14 (s, $\underline{C}_{Ar(6)}$, 1C), 122.75 (s, $\underline{C}HCO_2$, 1C), 121.77 (s, $\underline{C}H_{Ar(5)}$, 1C), 120.24 (d, $^{3}J_{PC}$ = 24.7, $\underline{C}_{Ar(3)}$, 1C), 118.07 (s, $\underline{C}_{Ar(8)}$, 1C), 117.42 (d, $^{1}J_{PC}$ = 182.6, P$\underline{C}$, 1C), 82.51 (s, $CO_2\underline{C}Me_3$, 1C), 62.35 (d, $^{2}J_{PC}$ = 4.8, $\underline{C}H_2OP$, 1C), 60.29 (s, $CO_2\underline{C}H_2$, 1C), 33.30 (s, $C_{Ar(6)}CH_2\underline{C}H_2$, 1C), 31.33 (s, $C_{Ar(6)}\underline{C}H_2$, 1C), 28.03 (s, $C(\underline{C}H_3)_3$, 3C), 16.29 (d, $^{3}J_{PC}$ = 6.9, $\underline{C}H_3CH_2OP$, 2C), 14.20 (s, $CO_2CH_2\underline{C}H_3$, 1C). ^{31}**P NMR** (101 MHz, $CDCl_3$) δ 17.31.

Ethyl 5-(3-(3-(*tert*-butoxy)-2-(diethoxyphosphoryl)-3-oxopropyl)imidazo[1,2-*a*]pyridin-6-yl)pentanoate (12e): obtained according to the reduction procedure for compound **11**. Scale: 170 mg of **14e**. Compound **12e** was purified by flash chromatography using Gilson PLC 2250 purification system. Eluents A: DCM and B: Acetone, to both of them TEA was added, 1mL/(1L of eluent) (gradient 0→45 min. 0→50%B, retention time 42 min.). Yield: 63% (107 mg). ^{1}**H NMR** (700 MHz, $CDCl_3$) δ 7.75 (s, $C\underline{H}_{Ar(5)}$, 1H), 7.46 (d, $^{3}J_{HH}$ = 9.2, $C\underline{H}_{Ar(8)}$, 1H), 7.35 (s, $C\underline{H}_{Ar(2)}$, 1H), 6.99 (dd, $^{3}J_{HH}$ = 9.2, $^{4}J_{HH}$ = 1.7, $C\underline{H}_{Ar(7)}$, 1H), 4.21 – 4.14 (m, $C\underline{H}_2OP$, 4H), 4.08 (t, $^{3}J_{HH}$ = 7.1, $CO_2C\underline{H}_2$, 2H), 3.50 (ddd, $^{2}J_{HH}$ = 16.2, $^{3}J_{HH}$ = 11.8, $^{3}J_{PH}$ = 6.6, $C\underline{H}_2C(H)P$, 1H), 3.26 (ddd, $^{2}J_{HH}$ = 15.8, $^{3}J_{PH}$ = 9.9, $^{3}J_{HH}$ = 2.6, $C\underline{H}_2C(H)P$, 1H), 3.23 (ddd, $^{2}J_{PH}$ = 22.6, $^{3}J_{HH}$ = 11.7, $^{3}J_{HH}$ = 2.9, $CH_2C(\underline{H})P$, 1H), 2.63 – 2.58 (m, $C_{Ar(6)}C\underline{H}_2$, 2H), 2.32 – 2.27 (m, $C\underline{H}_2CO_2$, 2H), 1.68 – 1.62 (m, $C\underline{H}_2C\underline{H}_2CH_2CO_2$, 4H), 1.34 (s, $C(C\underline{H}_3)_3$, 9H), 1.34 – 1.32 (m, $C\underline{H}_3CH_2OP$, 6H), 1.20 (t, $^{3}J_{HH}$ = 7.1, $CO_2CH_2C\underline{H}_3$, 3H). ^{13}**C NMR** (176 MHz, $CDCl_3$ 77.16) δ 173.40 (s, $\underline{C}O_2Et$, 1C), 167.35 (d, $^{2}J_{PC}$ = 5.1, $\underline{C}O_2t$Bu, 1C), 144.87 (s, $\underline{C}_{Ar(9)}$, 1C), 131.52 (s, $\underline{C}H_{Ar(2)}$, 1C), 126.20 (s, $\underline{C}_{Ar(6)}$, 1C), 125.81 (s, $\underline{CH}_{Ar(7)}$, 1C), 121.10 (d, $^{3}J_{PC}$ = 19.2, $\underline{C}_{Ar(3)}$, 1C), 120.66 (s, $\underline{C}H_{Ar(5)}$, 1C), 117.54 (s, $\underline{C}H_{Ar(8)}$, 1C), 82.54 (s, $CO_2\underline{C}Me_3$, 1C), 63.04 (d, $^{2}J_{PC}$ = 6.4, $\underline{C}H_2OP$, 1C), 62.94 (d, $^{2}J_{PC}$ = 6.8, $\underline{C}H_2OP$, 1C), 60.32 (s, $CO_2\underline{C}H_2$, 1C), 45.15 (d, $^{2}J_{PC}$ = 129.1, $CH_2\underline{C}(H)P$, 1C), 34.08 (s, $\underline{C}H_2CO_2Et$, 1C), 32.60 (s, $C_{Ar(6)}\underline{C}H_2$, 1C), 30.32 i 24.51 (s, $\underline{C}H_2\underline{C}H_2CH_2CO_2Et$, 2C), 27.85 (s, $C(\underline{C}H_3)_3$, 3C), 21.74 (d, $^{3}J_{PC}$ = 3.6, $\underline{C}H_2C(H)P$, 1C), 16.49 (d, $^{3}J_{PC}$ = 5.3, $\underline{C}H_3CH_2OP$, 1C), 16.46 (d, $^{3}J_{PC}$ = 5.5, $\underline{C}H_3CH_2OP$, 1C), 14.29 (s, $CO_2CH_2\underline{C}H_3$, 1C). ^{31}**P NMR** (101 MHz, $CDCl_3$) δ 22.34.

Ethyl 5-(3-(3-(*tert*-butoxy)-2-(diethoxyphosphoryl)-2-fluoro-3-oxopropyl)imidazo[1,2-*a*]pyridin-6-yl)pentanoate (13e): obtained according to the general procedure of fluorination. Scale: 107 mg (0.21 mmol) of **12e**. Compound **13e** was purified by flash chromatography using Gilson PLC 2250 purification system. Eluents A:DCM, B:Acetone, to both of them TEA was added, 1mL/(1L of eluent) (gradient 0→40 min. 10→40% B, retention time 27 min.). Yield: 69% (77 mg). ^{1}**H NMR** (250 MHz, $CDCl_3$) δ 7.83 (s, $C\underline{H}_{Ar(5)}$, 1H), 7.44 (d, $^{3}J_{HH}$ = 11.1, $C\underline{H}_{Ar(8)}$, 1H), 7.42 (s, $C\underline{H}_{Ar(2)}$, 1H), 6.98 (dd, $^{3}J_{HH}$ = 9.2, $^{4}J_{HH}$ = 1.6, $C\underline{H}_{Ar(7)}$, 1H), 4.29 – 4.13 (m, $POC\underline{H}_2$, 4H), 4.06 (q, $^{3}J_{HH}$ = 7.1, $CO_2C\underline{H}_2$, 2H), 3.94 – 3.51 (m, $C\underline{H}_2C(F)P$, 2H), 2.63 – 2.51 (m, $C_{Ar(6)}C\underline{H}_2CH_2$, 2H), 2.32 – 2.22 (m, $C\underline{H}_2CO_2$, 2H), 1.69 – 1.55 (m, $C\underline{H}_2C\underline{H}_2CH_2CO_2$, 4H), 1.38 – 1.27 (m, $POCH_2C\underline{H}_3$, 6H), 1.32 (s, $C(C\underline{H}_3)_3$, 9H), 1.19 (t, $^{3}J_{HH}$ = 7.1, $CO_2CH_2C\underline{H}_3$, 3H). ^{13}**C NMR** (63 MHz, $CDCl_3$) δ 173.4 (s, $\underline{C}O_2Et$, 1C), 165.0 (dd, $^{2}J_{FC}$ = 22.0,

$^2J_{PC}$ = 3.7, $\underline{C}O_2{}^tBu$, 1C), 145.2 (s, $\underline{C}_{Ar(9)}$, 1C), 134.0 (s, $\underline{C}H_{Ar(2)}$, 1C), 126.2 (s, $\underline{C}_{Ar(6)}$, 1C), 126.1 (s, $\underline{C}H_{Ar(7)}$, 1C), 121.5 (d, J_{FC} = 4.7, $\underline{C}H_{Ar(5)}$, 1C), 117.3 (s, $\underline{C}_{Ar(8)}$, 1C), 116.6 (d, $^3J_{PC}$=14.9, $\underline{C}_{Ar(3)}$, 1C), 96 (m, $\underline{C}$(F)P, 1C, in HMBC spectrum), 84.4 (s, $CO_2\underline{C}Me_3$, 1C), 64.6 (d, $^2J_{PC}$ = 6.7, $\underline{C}H_2OP$, 1C), 64.3 (d, $^2J_{PC}$ = 7.2, $\underline{C}H_2OP$, 1C), 60.3 (s, $CO_2\underline{C}H_2$, 1C), 34.0 (s, $CH_2\underline{C}H_2CO_2Et$, 1C), 32.5 (s, $C_{Ar(6)}\underline{C}H_2$, 1C), 30.3 and 24.4 (s, $\underline{C}H_2\underline{C}H_2CH_2$,$CO_2Et$, 2C), 28.3 (d, $^2J_{FC}$ = 20.6, $\underline{C}H_2C(F)P$, 1C), 27.8 (s, $C(\underline{C}H_3)_3$, 3C), 16.4 (d, $^3J_{PC}$ = 5.7, $\underline{C}H_3CH_2OP$, 1C), 14.2 (s, $CO_2CH_2\underline{C}H_3$, 1C). **^{31}P NMR** (101 MHz, $CDCl_3$) 13.15 (d, $^2J_{PF}$ = 83.7).

3-(6-(5-ethoxy-5-oxopentyl)imidazo[1,2-*a*]pyridin-3-yl)-2-fluoro-2-phosphonopropanoic acid (1e): obtained according to the general procedure of ester deprotection. Scale: 77 mg of **13e**. Product **1e** was purified by preparative HPLC (gradient 1→15 min. 0→30% B, retention time 13.6 min.) followed by lyophilization. Yield: 67% (33 mg). **^{1}H NMR** (700 MHz, D_2O pH 2) δ 8.56 (s, $C\underline{H}_{Ar(5)}$, 1H), 7.87 (dd, $^3J_{HH}$ = 9.3, $^4J_{HH}$ = 1.5, $C\underline{H}_{Ar(7)}$, 1H), 7.83 (s, $C\underline{H}_{Ar(2)}$, 1H), 7.82 (d, $^3J_{HH}$ = 9.2, $C\underline{H}_{Ar(7)}$, 1H), 4.17 (q, $^3J_{HH}$ = 7.2, $CO_2C\underline{H}_2$, 2H), 4.05 (ddd, $^3J_{FH}$ = 38.4, $^2J_{HH}$ = 16.5, $^3J_{PH}$ = 3.8, $C\underline{H}_2C(F)P$, 1H), 3.88 (ddd, $^2J_{HH}$ = 16.4, $^3J_{FH}$ = 9.4, $^3J_{PH}$ = 7.2, $C\underline{H}_2C(F)P$, 1H), 2.85 (t, $^3J_{HH}$ = 7.4, $C_{Ar(6)}C\underline{H}_2CH_2$, 2H), 2.46 (t, $^3J_{HH}$ = 7.3, $C\underline{H}_2CO_2$, 2H), 1.80 – 1.73 (m, $C\underline{H}_2CH_2CH_2CO_2$, 2H), 1.71 – 1.65 (m, $C\underline{H}_2CH_2CO_2$, 2H), 1.26 (t, $^3J_{HH}$ = 7.2, $CO_2CH_2C\underline{H}_3$, 3H). **^{13}C NMR** (176 MHz, D_2O pH 2) δ 176.9 (s, $\underline{C}O_2Et$, 1C), 171.8 (d, $^2J_{FC}$ = 23.6, $\underline{C}O_2H$, 1C), 139.0 (s, $\underline{C}_{Ar(9)}$, 1C), 135.4 (s, $\underline{C}H_{Ar(7)}$, 1C), 131.9 (s, $\underline{C}_{Ar(6)}$, 1C), 124.4 (s, $\underline{C}H_{Ar(5)}$, 1C), 121.1 (s, $\underline{C}H_{Ar(2)}$, 1C), 121.0 (d, $^3J_{PC}$=14.6, $\underline{C}_{Ar(3)}$, 1C), 111.5 (s, $\underline{C}_{Ar(8)}$, 1C), 97.0 (dd, $^1J_{FC}$ = 193.2, $^1J_{PC}$ = 143.8, $\underline{C}$(F)P, 1C), 61.6 (s, $CO_2\underline{C}H_2$, 1C), 33.7 (s, $CH_2\underline{C}H_2CO_2Et$, 1C), 31.3 (s, $C_{Ar(6)}\underline{C}H_2CH_2$, 1C), 29.1 (s, $C_{Ar(6)}CH_2\underline{C}H_2$, 1C), 27.6 (d, $^2J_{FC}$ = 20.0, $\underline{C}H_2C(F)P$, 1C), 23.5 (s, $\underline{C}H_2CH_2$,CO_2Et, 1C), 13.3 (s, $CO_2CH_2\underline{C}H_3$, 1C). **^{31}P NMR** (101 MHz, D_2O pH 2) δ 7.85 (d, $^2J_{PF}$ = 73.3). HR-MS: m/z $[M+H^+]$ calculated 417.1221, found 417.1222. Elemental analysis: $C_{17}H_{22}FN_2O_7P$*0.25H_2O*0.5CF_3CO_2H: calculated C45.24; H4.85; N5.86; found C45.25; H4.85; N5.86; max. diff. 0.01.

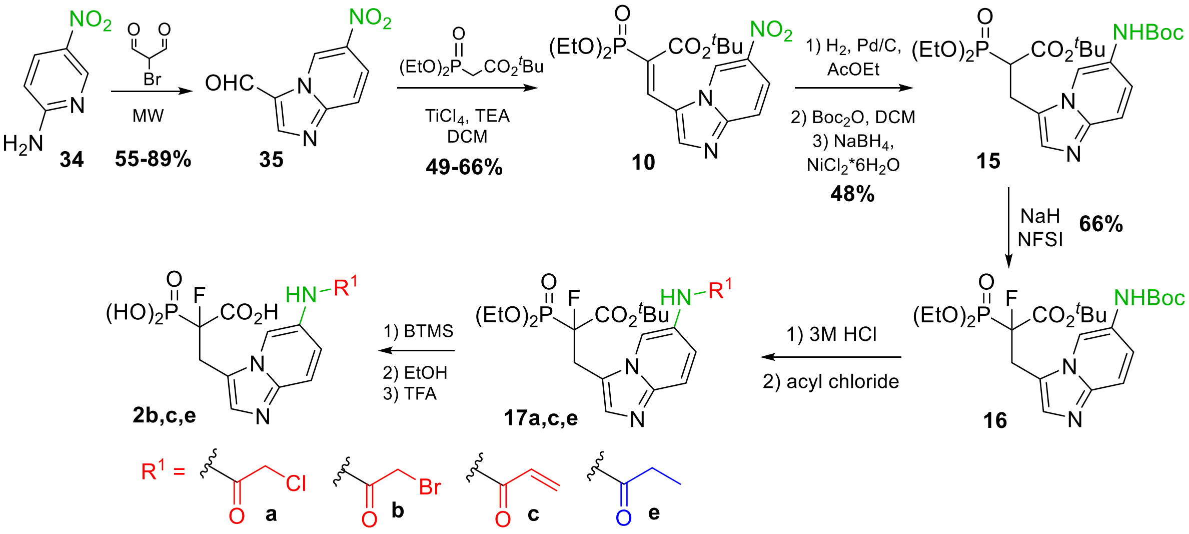

Scheme S6. Synthesis of compounds **2b,c,e.**

6-Nitroimidazo[1,2-*a*]pyridine-3-carbaldehyde (35): obtained according to the procedure for compound **31**, using 2-amino-5-nitropyridine **34**. Scale: 2.4 mmol (333 mg) of **34**. Crude product **35** was subjected to the Knovenagel condensation without futher purification. Yield: 89%. **^{1}H NMR** (700 MHz, $CDCl_3$) δ 7.85 (dd, $^3J_{HH}$ = 9.8, $^5J_{HH}$ = 0.9, $C\underline{H}_{Ar(8)}$, 1H), 8.27 (dd, $^3J_{HH}$ = 9.8, $^4J_{HH}$ = 2.3, $C\underline{H}_{Ar(7)}$,

1H), 8.45 (s, $C\underline{H}_{Ar(2)}$, 1H), 10.02 (s, $\underline{H}CO$, 1H), 10.42 (dd, $^{4}J_{HH}$ = 2.3, $^{5}J_{HH}$ = 0.9, $C\underline{H}_{Ar(5)}$, 1H). **^{13}C NMR** (176 MHz, $CDCl_3$) δ: 117.69 (s, $\underline{C}H_{Ar(8)}$, 1C), 123.89 (s, $\underline{C}H_{Ar(7)}$, 1C), 126.16 (s, $\underline{C}_{Ar(3)}$, 1C), 128.05 (s, $\underline{C}H_{Ar(5)}$, 1C), 139.15 (s, $\underline{C}_{Ar(6)}$, 1C), 148.53 (s, $\underline{C}H_{Ar(2)}$, 1C), 149.11 (s, $\underline{C}H_{Ar(9)}$, 1C), 178.56 (s, $\underline{C}HO$, 1C).

***Tert*-butyl (*E*)-2-(diethoxyphosphoryl)-3-(6-nitroimidazo[1,2-*a*]pyridin-3-yl)acrylate (10):** obtained according to the Knovenagel condensation procedure for compound **9**. Scale: 8.5 mmol of **35**. Compound **10** was purified by flash chromatography using Gilson PLC 2250 purification system. Eluents A:DCM and B:Acetone (gradient 15→30 min. 0→60%B, retention time 32 min.). Yield: 49%. **^{1}H NMR** (700 MHz, $CDCl_3$) δ 1.33 (t, $^{3}J_{HH}$ = 7.2, $C\underline{H}_3CH_2OP$, 6H), 1.52 (s, $C(C\underline{H}_3)_3$, 9H), 4.09 – 4.20 (m, $C\underline{H}_2OP$, 4H), 7.73 (dd, $^{3}J_{HH}$ = 9.8, $^{5}J_{HH}$ = 0.8, $C\underline{H}_{Ar(8)}$, 1H), 7.76 (d, $^{3}J_{PH}$ = 23.6, C=C$\underline{H}$, 1H), 8.05 (dd, $^{3}J_{HH}$ = 9.8, $^{4}J_{HH}$ = 2.1, $C\underline{H}_{Ar(7)}$, 1H), 8.45 (s, $C\underline{H}_{Ar(2)}$, 1H), 9.35 (dd, $^{4}J_{HH}$ = 2.1, $C\underline{H}_{Ar(5)}$, 1H); **^{13}C NMR** (176 MHz, $CDCl_3$) δ: 16.33 (d, $^{3}J_{PC}$ = 6.4, $\underline{C}H_3CH_2OP$, 2C), 28.01 (s, $C(\underline{C}H_3)_3$, 3C), 62.76 (d, $^{2}J_{PC}$ = 5.1, $\underline{C}H_2OP$, 2C), 83.37 (s, $\underline{C}(CH_3)_3$, 1C), 118.16 (s, $\underline{C}H_{Ar(8)}$, 1C), 120.49 (s, $\underline{C}H_{Ar(7)}$, 1C), 122.55 (d, $^{3}J_{PC}$ = 24.7, $\underline{C}H_{Ar(3)}$, 1C), 123.35 (d, $^{1}J_{PC}$ = 180.0, P$\underline{C}$, 1C), 123.90 (s, $\underline{C}H_{Ar(5)}$, 1C), 130.01 (d, $^{2}J_{PC}$ = 10.1, PC=$\underline{C}$H, 1C), 138.33 (s, $\underline{C}_{Ar(6)}$, 1C), 142.17 (s, $\underline{C}H_{Ar(2)}$, 1C), 147.19 (s, $\underline{C}H_{Ar(9)}$, 1C), 164.61 (d, $^{2}J_{PC}$ = 10.5, $\underline{C}O_2$, 1C). **^{31}P NMR** (283 MHz, $CDCl_3$) δ 16.41.

***Tert*-butyl 3-(6-((tert-butoxycarbonyl)amino)imidazo[1,2-*a*]pyridin-3-yl)-2-(diethoxyphosphoryl)propanoate (15):** obtained in three-step synthesis. The first step involved the reduction of nitro group. This reaction was carried out in a single-neck flask equipped with two-way stopcock, which enabled degassing the system (vacuum–hydrogen, three times). In a singleneck flask compound **10** (520 mg, 1.23 mmol) and 10% Pd/C (60 mg) was placed in AcOEt (60 mL). The system was degassed using a two-way stopcock. This suspension was stirred for 48h at room temperature. The catalyst was then filtered off through a thin layer of Celite500, and the filtrate was evaporated to dryness. Thus obtained amine derivative was used in the second step which involved introduction of *tert*-butyloxycarbonyl protecting group (Boc). Amine derivative, obtained in the reduction step, was dissolved in DCM (30 mL). Then, Boc_2O (2 eq., 2.82 mmol, 616 mg) was added and the mixture was stirred for 24h at room temperature. Next, the solvent was evaporated and obtained oil subjected to the third step, reduction of carbon double bond *HC*=*C*-CO_2*t*Bu using the procedure with $NaBH_4$ previously described for compound **11**. Compound **15** was purified by flash chromatography using Gilson PLC 2250 purification system. Eluents A: DCM and B: Acetone, to both of them TEA was added, 1mL/(1L of eluent) (gradient 0→30 min. 0→70%B, retention time 24 min.). Yield: 48% (350 mg). **^{1}H NMR** (700 MHz, $CDCl_3$) δ 8.52 (bs, $C\underline{H}_{Ar(5)}$, 1H), 7.54 (d, $^{3}J_{HH}$ = 9.2, $C\underline{H}_{Ar}$, 1H), 7.41 (s, $C\underline{H}_{Ar(2)}$, 1H), 6.96 (dd, $^{3}J_{HH}$ = 9,5, $^{4}J_{HH}$ = 2.0, $C\underline{H}_{Ar}$, 1H), 4.29 – 4.14 (m, $C\underline{H}_2OP$, 4H), 3.63 – 3.46 (m, $C\underline{H}_2C\underline{H}P$, 1H), 3.37 – 3.19 (m, $C\underline{H}_2C\underline{H}P$, 2H), 1.53 (s, $(C\underline{H}_3)_3COC(O)N$, 18H), 1.39, (s, $(C\underline{H}_3)_3OCC$, 9H), 1.38 (t, $^{3}J_{HH}$ = 7.2, $C\underline{H}_3CH_2OP$, 6H). **^{31}P NMR** (101 MHz, $CDCl_3$) δ 21.84.

***Tert*-butyl 3-(6-((*tert*-butoxycarbonyl)amino)imidazo[1,2-*a*]pyridin-3-yl)-2-(diethoxyphosphoryl)-2-fluoropropanoate (16):** obtained according to the *general procedure of fluorination*. Scale: 190 mg (0.38 mmol) of **15**. Compound **16** was purified by flash chromatography using Gilson PLC 2250 purification system. Eluents A:DCM, B:Acetone, to both of them TEA was added, 1mL/(1L of eluent) (gradient 0→35 min. 0→60% B, retention time 17 min.). Yield: 66% (130 mg). **^{1}H NMR** (700 MHz, $CDCl_3$) δ 8.60 (bs, $C\underline{H}_{Ar}$ lub N$\underline{H}$, 1H), 7.53 (d, $^{3}J_{HH}$ = 10.4, $C\underline{H}_{Ar}$, 1H), 7.51 (s, $C\underline{H}_{Ar}$, 1H), 6.98 (dd, $^{3}J_{HH}$ = 9.6, $^{5}J_{HH}$ = 2.0, $C\underline{H}_{Ar}$, 1H), 6.40 (bs, $C\underline{H}_{Ar}$ lub N$\underline{H}$, 1H), 4.39 – 4.21 (m, $C\underline{H}_2OP$, 4H), 3.87 (ddd, $^{3}J_{FH}$ = 37.5, $^{2}J_{HH}$ = 16.1, $^{3}J_{PH}$= 5.1, $C\underline{H}_2CFP$, 1H), 3.67 (ddd, $^{2}J_{HH}$ = 16.0, $^{3}J_{FH}$ = 11.4, $^{3}J_{PH}$= 7.0, $C\underline{H}_2CFP$, 1H), 1.52 (s, $(C\underline{H}_3)_3COC(O)N$, 9H), 1.41 (s, $CO_2(C\underline{H}_3)_3$, 9H), 1.40 (t, $^{3}J_{HH}$ = 6.8, $C\underline{H}_3CH_2OP$, 6H). **^{31}P NMR** (700 MHz, $CDCl_3$) δ 12.32 (d, $^{2}J_{PF}$ = 83.5).

***Tert*-butyl 3-(6-(2-chloroacetamido)imidazo[1,2-*a*]pyridin-3-yl)-2-(diethoxyphosphoryl)-2-fluoropropanoate (17a):** obtained according to the *general procedure of coupling reaction* for synthesis of compounds **17** using chloroacetic chloride. Scale: 105 mg (0.20 mmol) of **16**. Compound **17a** was purified by flash chromatography using Gilson PLC 2250 purification system. Eluents A:DCM, B:Acetone, to both of them TEA was added, 1mL/(1L of eluent) (gradient 0→5 min. 0→50% B, retention time 17 min.). Yield: 29% (29 mg). **^{1}H NMR** (700 MHz, $CDCl_3$) δ 9.04 (s, N$\underline{H}$, 1H), 8.96 (s, $CH_{Ar(5)}$, 1H), 7.51 (s, C$\underline{H}_{Ar(2)}$, 1H), 7.48 (d, $^3J_{HH}$ = 9.5, C$\underline{H}_{Ar(8)}$, 1H, 1H), 7.09 (dd, $^3J_{HH}$ = 9.6, $^4J_{HH}$=2.0, C$\underline{H}_{Ar(7)}$, 1H), 4.31 – 4.23 (m, C$\underline{H}_2$OP, 4H), 4.18 (s, CH_2Cl, 1H), 3.83 (ddd, $^3J_{FH}$ = 37.4, $^2J_{HH}$ = 16.2, $^3J_{PH}$ = 5.8, C$\underline{H}_2$C(F)P, 1H), 3.67 (ddd, $^2J_{HH}$ = 16.1, $^3J_{FH}$ = 12.3, $^3J_{PH}$ = 6.9, C$\underline{H}_2$C(F)P, 1H), 1.38 (s, C(C$\underline{H}_3$)$_3$, 9H), 1.374 i 1.369 (2t, $^3J_{HH}$ = 7.1, C$\underline{H}_3$CH$_2$OP, 6H). **^{13}C NMR** (176 MHz, $CDCl_3$) δ 165.1 (dd, $^2J_{FC}$ = 22.3, $^2J_{PC}$ = 3.8, $\underline{C}$O$_2$*t*Bu, 1C), 164.8 (s, $\underline{C}$ONH, 1C), 143.7 (s, $\underline{C}_{Ar(9)}$, 1C), 134.14 (s, $\underline{C}H_{Ar(2)}$, 1C), 125.3 (s, $\underline{C}_{Ar(6)}$, 1C), 120.5 (s, $\underline{C}H_{Ar(7)}$, 1C), 118.3 (d, $^3J_{PC}$=14.3, $\underline{C}_{Ar(3)}$, 1C), 117.5 (s, $\underline{C}H_{Ar(8)}$, 1C), 116.0 (d, J_{FC} = 5.1, $\underline{C}H_{Ar(5)}$, 1C), 95.8 (dd, $^1J_{FC}$ = 198.7, $^1J_{PC}$ = 160.1, $\underline{C}$(F)P, 1C), 84.8 (s, CO$_2\underline{C}$Me$_3$, 1C), 64.8 (d, $^2J_{PC}$ = 6.5, $\underline{C}$H$_2$OP, 1C), 64.7 (d, $^2J_{PC}$ = 7.2, $\underline{C}$H$_2$OP, 1C), 42.9 (s, $\underline{C}$H$_2$Cl, 1C), 28.5 (d, $^2J_{FC}$ = 20.5, $\underline{C}$H$_2$C(F)P, 1C), 27.9 (s, C($\underline{C}$H$_3$)$_3$, 3C), 16.5 (d, $^3J_{PC}$ = 6.2, $\underline{C}$H$_3$CH$_2$OP, 2C). **^{31}P NMR** (101 MHz, $CDCl_3$) δ 12.13 (d, $^2J_{PF}$ = 84.0).

***Tert*-butyl 3-(6-acrylamidoimidazo[1,2-*a*]pyridin-3-yl)-2-(diethoxyphosphoryl)-2-fluoropropanoate (17c):** obtained according to the *general procedure of coupling reaction* for synthesis of compounds **17** using acrylic chloride. Scale: 100 mg (0.16 mmol) of **16**. Compound **17c** was purified by flash chromatography using Gilson PLC 2250 purification system. Eluents A:DCM, B:Acetone, to both of them TEA was added, 1mL/(1L of eluent) (gradient 0→10 min. 0→50% B, retention time 21 min.). Yield: 26% (20 mg). **^{1}H NMR** (700 MHz, $CDCl_3$) δ 9.21 (s, N$\underline{H}$, 1H), 9.15 (s, C$\underline{H}_{Ar(5)}$, 1H), 7.50 (s, C$\underline{H}_{Ar(2)}$, 1H), 7.40 (dd, $^3J_{HH}$ = 9.5, C$\underline{H}_{Ar(7)}$, 1H), 7.09 (dd, $^3J_{HH}$ = 9.6, $^4J_{HH}$ = 1.9, C$\underline{H}_{Ar(8)}$, 1H), 6.41 (dd, $^3J_{HH(trans)}$ = 16.9, $^2J_{HH}$ = 1.7, OCCHC$\underline{H}_2$, 1H), 6.35 (dd, $^3J_{HH(trans)}$ = 16.9, $^3J_{HH(cis)}$ = 9.9, OCC$\underline{H}$CH$_2$, 1H), 5.69 (dd, $^3J_{HH(cis)}$ = 9.9, $^3J_{HH}$ = 1.7, OCCHC$\underline{H}_2$, 1H), 4.33 – 4.25 (m, C$\underline{H}_2$OP, 4H), 3.85 (ddd, $^3J_{FH}$ = 37.6, $^2J_{HH}$ = 16.2, $^3J_{PH}$ = 5.3, C$\underline{H}_2$C(F)P, 1H), 3.69 (ddd, $^2J_{HH}$ = 16.0, $^3J_{FH}$ = 11.4, $^3J_{PH}$ = 6.9, C$\underline{H}_2$C(F)P, 1H), 1.40 – 1.37 (m, C$\underline{H}_3$CH$_2$OP, 5H), 1.39 (s, C(C$\underline{H}_3$)$_3$, 9H). **^{13}C NMR** (176 MHz, $CDCl_3$) δ 165.2 (d, $^2J_{FC}$ = 22.6, $^2J_{PC}$ = 3.6, $\underline{C}$O$_2$*t*Bu, 1C), 164.3 (s, $\underline{C}$ONH, 1C), 143.7 (s, $\underline{C}_{Ar(9)}$, 1C), 133.9 (s, $\underline{C}H_{Ar(2)}$, 1C), 130.8 (s, OC$\underline{C}$H=CH$_2$, 1C), 127.9 (s, OCCH=$\underline{C}$H$_2$, 1C), 126.4 (s, $\underline{C}_{Ar(6)}$, 1C), 120.5 (s, $\underline{C}H_{Ar(8)}$, 1C), 118.1 (d, $^3J_{PC}$ = 15.1, $\underline{C}_{Ar(3)}$, 1C), 117.2 (s, $\underline{CH}_{Ar(7)}$, 1C), 115.5 (d, J_{FC} = 4.8, $\underline{C}H_{Ar(5)}$, 1C), 95.9 (dd, $^1J_{FC}$ = 198.6, $^1J_{PC}$ = 160.1, $\underline{C}$(F)P, 1C), 84.7 (s, CO$_2\underline{C}$Me$_3$, 1C), 64.8 (d, $^2J_{PC}$ = 7.2, $\underline{C}$H$_2$OP, 1C), 64.7 (d, $^2J_{PC}$ = 7.4, $\underline{C}$H$_2$OP, 1C), 28.5 (d, $^2J_{FC}$ = 21.0, $\underline{C}$H$_2$C(F)P, 1C), 27.9 (s, C($\underline{C}$H$_3$)$_3$, 3C), 16.6 (d, $^3J_{PC}$ = 5.1, $\underline{C}$H$_3$CH$_2$OP, 2C). **^{31}P NMR** (284 MHz, $CDCl_3$) δ 11.84 (d, $^2J_{PF}$ = 83.3).

***Tert*-butyl 2-(diethoxyphosphoryl)-2-fluoro-3-(6-propionamidoimidazo[1,2-*a*]pyridin-3-yl)propanoate (17e):** obtained according to the *general procedure of coupling reaction* for synthesis of compounds **17** using acetic chloride. Scale: 175 mg (0.35 mmol) of **16**. Compound **17e** was purified by flash chromatography using Gilson PLC 2250 purification system. Eluents A:DCM, B:Acetone, to both of them TEA was added, 1mL/(1L of eluent). Yield: 36% (60 mg). **^{1}H NMR** (700 MHz, $CDCl_3$) δ 8.89 (s, C$\underline{H}_{Ar(5)}$, 1H), 7.66 (s, N$\underline{H}$, 1H), 7.50 (s, C$\underline{H}_{Ar(2)}$, 1H), 7.44 (d, $^3J_{HH}$ = 9.5, C$\underline{H}_{Ar}$, 1H), 7.09 (dd, $^3J_{HH}$ = 9.5, $^4J_{HH}$ = 2.0, C$\underline{H}_{Ar}$, 1H), 4.33 – 4.23 (m, C$\underline{H}_2$OP, 4H), 3.84 (ddd, $^3J_{FH}$ = 37.6, $^2J_{HH}$ = 16.2, $^3J_{PH}$ = 5.6, C$\underline{H}_2$C(F)P, 1H), 3.68 (ddd, $^2J_{HH}$ = 16.1, $^3J_{FH}$ = 11.6, $^3J_{PH}$ = 6.9, C$\underline{H}_2$C(F)P, 1H), 2.41 (q, $^3J_{HH}$ = 7.6, OCC$\underline{H}_2$CH$_3$, 2H), 1.41 (s, C(C$\underline{H}_3$)$_3$, 9H), 1.40 (t, $^3J_{HH}$ = 7.0, C$\underline{H}_3$CH$_2$OP, 3H), 1.39 (t, $^3J_{HH}$ = 7.0, C$\underline{H}_3$CH$_2$OP, 3H), 1.24 (t, $^3J_{HH}$ = 7.6, OCCH$_2$C$\underline{H}_3$, 3H). **^{13}C NMR** (176 MHz, $CDCl_3$) δ 173.1 (s, $\underline{C}$ONH, 1C), 165.2 (dd, $^2J_{FC}$ = 22.6, $^2J_{PC}$ = 3.5, $\underline{C}$O$_2$*t*Bu, 1C), 143.6 (s, $\underline{C}_{Ar(9)}$, 1C), 133.7 (s, $\underline{C}H_{Ar(2)}$, 1C), 126.6 (s, $\underline{C}_{Ar(6)}$, 1C), 120.5 (s, $\underline{C}H_{Ar}$, 1C), 117.9 (d, $^3J_{PC}$ = 15.0, $\underline{C}_{Ar(3)}$, 1C), 117.0 (s, $\underline{C}H_{Ar}$, 1C), 115.0 (d, J_{FC} = 3.2, $\underline{C}H_{Ar(5)}$, 1C), 95.8 (dd, $^1J_{FC}$ = 198.4, $^1J_{PC}$ = 160.2, $\underline{C}$(F)P, 1C), 84.7 (s, CO$_2\underline{C}$Me$_3$,

1C), 64.8 (d, $^{2}J_{PC}$ = 7.0, $\underline{C}H_2OP$, 1C), 64.7 (d, $^{2}J_{PC}$ = 6.6, $\underline{C}H_2OP$, 1C), 30.1 (s, $OC\underline{C}H_2CH_3$, 1C), 28.4 (d, $^{2}J_{FC}$ = 20.6, $\underline{C}H_2C(F)P$, 1C), 27.8 (s, $C(\underline{C}H_3)_3$, 3C), 16.5 (d, $^{3}J_{PC}$ = 5.4, $\underline{C}H_3CH_2OP$, 2C), 9.7 (s, $OCCH_2\underline{C}H_3$, 1C). **^{31}P NMR** (284 MHz, $CDCl_3$) δ 12.52 (d, $^{2}J_{PF}$ = 83.5).

3-(6-(2-bromoacetamido)imidazo[1,2-*a*]pyridin-3-yl)-2-fluoro-2-phosphonopropanoic acid (2b): obtained according to the general procedure of ester deprotection. Scale: 29 mg of **17a**. Product **2b** was purified by preparative HPLC (gradient 5→20 min. 0→40% B, retention time 8.8 min.) followed by lyophilization. Yield: 58% (13 mg). **^{1}H NMR** (700 MHz, D_2O pH 6) δ 9.16 (s, $CH_{Ar(5)}$, 1H), 7.97 (dd, $^{3}J_{HH}$ = 9.7, $^{4}J_{HH}$=1.8, $C\underline{H}_{Ar(7)}$, 1H), 7.94 (dd, $^{3}J_{HH}$ = 9.7, $^{4}J_{HH}$=0.9, $C\underline{H}_{Ar(8)}$, 1H), 7.91 (s, $C\underline{H}_{Ar(2)}$, 1H), 4.20 (s, CH_2Br, 1H), 4.07 (ddd, $^{3}J_{FH}$ = 37.7, $^{2}J_{HH}$ = 16.5, $^{3}J_{PH}$ = 4.3, $C\underline{H}_2C(F)P$, 1H), 3.91 (ddd, $^{2}J_{HH}$ = 16.7, $^{3}J_{FH}$ = 10.4, $^{3}J_{PH}$ = 6.6, $C\underline{H}_2C(F)P$, 1H). **^{13}C NMR** (176 MHz, D_2O pH 6) δ 169.0 (s, $\underline{C}ONH$, 1C), 171 (m, $\underline{C}O_2$, 1C, in HMBC spectrum), 137.9 (s, $\underline{C}_{Ar(9)}$, 1C), 129.2 (s, $\underline{C}H_{Ar(7)}$, 1C), 128.7 (s, $\underline{C}_{Ar(6)}$, 1C), 122.1 (s, $\underline{C}H_{Ar(2)}$, 1C), 122.0 (d, $^{3}J_{PC}$=12.2, $\underline{C}_{Ar(3)}$, 1C), 118.9 (d, J_{FC} = 4.8, $\underline{C}H_{Ar(5)}$, 1C), 112.3 (s, $\underline{C}H_{Ar(8)}$, 1C), 97 (m, $\underline{C}(F)P$, 1C, in HMBC spectrum), 28.3 (s, $\underline{C}H_2Br$, 1C), 27.7 (d, $^{2}J_{FC}$ = 21.0, $\underline{C}H_2C(F)P$, 1C). **^{31}P NMR** (284 MHz, D_2O, pH 2) δ 7.37 (d, $^{2}J_{PF}$ = 71.4). HR-MS: m/z [M+H$^+$] calculated 423.9704, found 423.9692. Elemental analysis: $C_{12}H_{12}BrFN_3O_6P$*1.3H_2O: calculated C32.21; H3.29; N9.39; found C31.91; H2.99; N9.43; max. diff. 0.3;

3-(6-acrylamidoimidazo[1,2-*a*]pyridin-3-yl)-2-fluoro-2-phosphonopropanoic acid (2c): obtained according to the general procedure of ester deprotection. Reaction with BTMS carried out in presence of TEA (10 eq). Scale: 20 mg of **17c**. Product **2c** was purified by preparative HPLC (gradient 5→20 min. 0→40% B, retention time 7.5 min.) followed by lyophilization. Yield: 51% (8 mg). In the ^{13}C NMR spectrum of **2c** signals from $\underline{C}F_3\underline{C}O_2^-$ are present. **^{1}H NMR** (700 MHz, D_2O pH 7) δ 8.99 (s, $C\underline{H}_{Ar(5)}$, 1H), 7.75 (bs, $C\underline{H}_{Ar(7)}$, $C\underline{H}_{Ar(8)}$, 2H), 7.67 (s, $C\underline{H}_{Ar(2)}$, 1H), 6.49 (dd, $^{3}J_{HH}$ = 17.0, 10.1, $C\underline{H}=CH_2$, 1H), 6.43 (dd, $^{3}J_{HH}$ = 17.0, $^{2}J_{HH}$ = 1.3, $CH=C\underline{H}_2$, 1H), 5.97 (dd, $^{3}J_{HH}$ = 10.0, $^{2}J_{HH}$ = 1.2, $CH=C\underline{H}_2$, 1H), 3.96 (dd, $^{3}J_{FH}$ = 39.7, $^{2}J_{HH}$ = 16.2, $C\underline{H}_2C(F)P$, 1H), 3.80 (ddd, $^{2}J_{HH}$ = 15.6, $^{3}J_{FH/PH}$ = 7.5, $C\underline{H}_2C(F)P$, 1H). **^{13}C NMR** (176 MHz, D_2O pH 7) δ 174.4 (d, $^{2}J_{FC}$ = 20.3, $\underline{C}O_2H$, 1C), 167.3 (s, $\underline{C}ONH$, 1C), 139.6 (s, $\underline{C}_{Ar(9)}$, 1C), 129.7 (s, $C\underline{H}=CH_2$, 1C), 129.3 (s, $CH=\underline{C}H_2$, 1C), 127.3 (s, $\underline{C}_{Ar(6)}$, 1C), 126.7 (s, $\underline{C}H_{Ar(7)}$, 1C), 125.1 (s, $\underline{C}H_{Ar(2)}$, 1C), 122.8 (d, $^{3}J_{PC}$ = 15.2, $\underline{C}_{Ar(3)}$, 1C), 118.6 (d, J_{FC} = 5.2, $\underline{C}H_{Ar(5)}$, 1C), 113.3 (s, $\underline{C}H_{Ar(8)}$, 1C), 98.9 (dd, $^{1}J_{FC}$ = 192.4, $^{1}J_{PC}$ = 145.8, $\underline{C}(F)P$, 1C), 28.6 (dd, $^{2}J_{FC}$ = 21.3, $^{2}J_{PC}$ = 3.2, $\underline{C}H_2C(F)P$, 1C). **^{31}P NMR** (284 MHz, D_2O pH 7) δ 10.62 (d, $^{2}J_{PF}$ = 75.8). HR-MS: m/z [M+H$^+$] calculated 358.0599, found 358.0589.

2-fluoro-2-phosphono-3-(6-propionamidoimidazo[1,2-*a*]pyridin-3-yl)propanoic acid (2e): obtained according to the general procedure of ester deprotection. Scale: 60 mg of **17e**. Product **2e** was purified by preparative HPLC (gradient 5→20 min. 0→40% B, retention time 8.1 min.) followed by lyophilization. Yield: 83% (38 mg). **^{1}H NMR** (700 MHz, D_2O pH 2) δ 9.14 (s, $CH_{Ar(5)}$, 1H), 7.91 (dd, $^{3}J_{HH}$ = 9.7, $^{4}J_{HH}$=1.7, $C\underline{H}_{Ar}$, 1H), 7.87 (dd, $^{3}J_{HH}$ = 9.6, $^{4}J_{HH}$=0.8, $C\underline{H}_{Ar}$, 1H), 7.89 (s, $C\underline{H}_{Ar(2)}$, 1H), 4.05 (ddd, $^{3}J_{FH}$ = 37.9, $^{2}J_{HH}$ = 16.6, $^{3}J_{PH}$ = 4.4, $C\underline{H}_2C(F)P$, 1H), 3.9 (ddd, $^{2}J_{HH}$ = 16.8, $^{3}J_{FH}$ = 10.5, $^{3}J_{PH}$ = 6.6, $C\underline{H}_2C(F)P$, 1H),2.55 (q, $^{3}J_{HH}$ = 7.6, $OCC\underline{H}_2CH_3$, 2H), 1.24 (t, $^{3}J_{HH}$ = 7.6, $OCCH_2C\underline{H}_3$, 3H). **^{13}C NMR** (176 MHz, D_2O pH 2) δ 177.2 (s, $\underline{C}ONH$, 1C), 171.7 (dd, $^{2}J_{FC}$ = 23.1, $^{2}J_{PC}$ = 2.1, $\underline{C}O_2$, 1C), 137.5 (s, $\underline{C}_{Ar(9)}$, 1C), 129.1 (s, $\underline{C}H_{Ar}$, 1C), 128.8 (s, $\underline{C}_{Ar(6)}$, 1C), 123.1 (d, $^{3}J_{PC}$ = 14.3, $\underline{C}_{Ar(3)}$, 1C), 121.5 (s, $\underline{C}H_{Ar(2)}$, 1C), 118.6 (d, J_{FC} = 5.0, $\underline{C}H_{Ar(5)}$, 1C), 112.0 (s, $\underline{C}H_{Ar}$, 1C), 98.3 (dd, $^{1}J_{FC}$ = 192.8, $^{1}J_{PC}$ = 143.7, $\underline{C}(F)P$, 1C), 29.6 (s, $OC\underline{C}H_2CH_3$, 1C), 28.4 (dd, $^{2}J_{FC}$ = 20.4, $^{2}J_{PC}$ = 3.0, $\underline{C}H_2C(F)P$, 1C), 9.0 (s, $OCCH_2\underline{C}H_3$, 1C). **^{31}P NMR** (284 MHz, D_2O pH 2) 7.30 (d, $^{2}J_{PF}$ = 71.2). HR-MS: m/z [$C_{13}H_{15}FN_3O_6P$+H$^+$] calculated 360.0755, found 360.0754. Elemental analysis: $C_{13}H_{15}FN_3O_6P$*0.75H_2O*HCl: calculated C38.67; H4.33; N10.41; found C38.55; H4.32; N10.53; max. diff. 0.12;

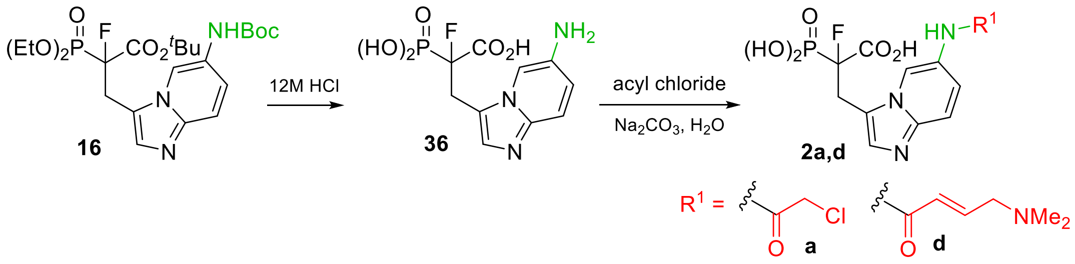

Scheme S7. Synthesis of compounds **2a,d** from compound **16**.

3-(6-aminoimidazo[1,2-a]pyridin-3-yl)-2-fluoro-2-phosphonopropanoic acid (36): to the compound **16** (60 mg) 12 M HCl (4 mL) was added. The mixture was held at reflux for 5 h. Excess HCl was evaporated. Compound **36** was purified by preparative HPLC (eluent A, isocratic, retention time 2.8 min.) as eluent followed by lyophilization from 0.1M HCl (repeated three times). Yield: 63% (26 mg). ^{1}**H NMR** (700 MHz, D_2O, pH 2) δ 8.64 (s, $CH_{Ar(5)}$, 1H), 7.96 (d, $^{3}J_{HH}$ = 9.6, $C\underline{H}_{Ar(7)}$, 1H), 7.92 (s, $C\underline{H}_{Ar(2)}$, 1H), 7.85 (d, $^{3}J_{HH}$ = 9.6, $C\underline{H}_{Ar(8)}$, 1H), 4.04 (ddd, $^{3}J_{FH}$ = 37.4, $^{2}J_{HH}$ = 16.6, $^{3}J_{PH}$ = 4.8, $C\underline{H}_2C(F)P$, 1H), 3.89 (ddd, $^{2}J_{HH}$ = 17.1, $^{3}J_{FH}$ = 11.1, $^{3}J_{PH}$ = 6.6, $C\underline{H}_2C(F)P$, 1H). ^{13}**C NMR** (176 MHz, D_2O, pH 2) δ 171.6 (d, $^{2}J_{FC}$ = 23.9, $\underline{C}O_2$, 1C), 138.0 (s, $\underline{C}_{Ar(9)}$, 1C), 128.4 (s, $\underline{C}H_{Ar(8)}$, 1C), 128.4 (s, $\underline{C}_{Ar(6)}$, 1C), 122.4 (s, $\underline{C}H_{Ar(2)}$, 1C), 121.9 (d, $^{3}J_{PC}$ = 12.8, $\underline{C}_{Ar(3)}$, 1C), 117.9 (s, $\underline{C}H_{Ar(5)}$, 1C) 113.2 (s, $\underline{C}H_{Ar(7)}$, 1C), 96.9 (dd, $^{1}J_{FC}$ = 193.2, $^{1}J_{PC}$ = 144.1, $\underline{C}(F)P$, 1C), 27.6 (dd, $^{2}J_{FC}$ = 20.3, $^{2}J_{PC}$ = 2.9, $\underline{C}H_2C(F)P$, 1C). ^{31}**P NMR** (284 MHz, D_2O pH 2) δ 6.79 (d, $^{2}J_{PF}$ = 67.1). HR-MS: m/z [$M+H^+$] calculated 304.0493, found 304.0485.

3-(6-(2-chloroacetamido)imidazo[1,2-*a*]pyridin-3-yl)-2-fluoro-2-phosphonopropanoic acid (2a): Compound **36** (42 mg) was dissolved in water (3 mL) in a single neck flask. The solution was cooled to 0 °C and 1M NaOH (approx. 1 mL) was added to reach pH 9. Then, chloroacetic acid chloride (5 equiv, 50 µL) was added forming a separate liquid phase on the water surface. This phase disappeared after 30 minutes of rapid stirring. Then, the pH of the reaction mixture (pH 2) was checked and re-adjusted to pH 9 using 1M NaOH. Another portion of chloroacetic acid chloride (5 eq) was added and continued mixing for 30 minutes. The solvent was evaporated. In order to remove the NaCl salt formed in the reaction, the crude product was purified using an Amberlite IR120 ion exchange column (H_2O as eluent). Product **2a** was further purified by preparative HPLC using A: H_2O:ACN:TFA 95:5:0,1; B: H_2O:ACN:TFA 5:95:0,1 (gradient 5→20 min. 0→40% B, retention time 8.6 min.) as eluent followed by lyophilization. Yield: 23% (11 mg). ^{1}**H NMR** (700 MHz, D_2O, pH 2) δ 9.02 (s, $CH_{Ar(5)}$, 1H), 7.93 (dd, $^{3}J_{HH}$ = 9.7, $^{4}J_{HH}$=1.7, $C\underline{H}_{Ar}$, 1H), 7.87 (d, $^{3}J_{HH}$ = 9.6, $C\underline{H}_{Ar}$, 1H), 7.78 (s, $C\underline{H}_{Ar(2)}$, 1H), 4.42 (s, CH_2Cl, 1H), 3.99 (ddd, $^{3}J_{FH}$ = 38.7, $^{2}J_{HH}$ = 16.1, $^{3}J_{PH}$ = 3.3, $C\underline{H}_2C(F)P$, 1H), 3.76 (ddd, $^{2}J_{HH}$ = 15.8, $^{3}J_{FH}$ = 7.8, $^{3}J_{PH}$ = 7.8, $C\underline{H}_2C(F)P$, 1H). ^{13}**C NMR** (176 MHz, D_2O, pH 2) δ 168.9 (s, $\underline{C}ONH$, 1C), 173 ($\underline{C}O_2$, 1C), 138.3 (s, $\underline{C}_{Ar(9)}$, 1C), 128.8 (s, $\underline{C}H_{Ar}$, 1C), 127.8 (s, $\underline{C}_{Ar(6)}$, 1C), 123.1 (d, $^{3}J_{PC}$=14.3, $\underline{C}_{Ar(3)}$, 1C), 122.5 (s, $\underline{C}H_{Ar(2)}$, 1C), 119.6 (d, J_{FC} = 4.9, $\underline{C}H_{Ar(5)}$, 1C), 112.5 (s, $\underline{C}H_{Ar}$, 1C), 98 (m, $\underline{C}(F)P$, 1C, in HMBC spectrum), 42.7 (s, $\underline{C}H_2Cl$, 1C), 28.4 (dd, $^{2}J_{FC}$ = 21.4, $^{2}J_{PC}$ = 3.9, $\underline{C}H_2C(F)P$, 1C). ^{31}**P NMR** (284 MHz, D_2O pH 2) δ 10.05 (d, $^{2}J_{PF}$ = 70.3). HR-MS: m/z [$M+H^+$] calculated 380.0209, found 380.0205.

(*E*)-3-(6-(4-(dimethylamino)but-2-enamido)imidazo[1,2-*a*]pyridin-3-yl)-2-fluoro-2-phosphonopropanoic acid (2d): obtained according to the procedure for analog **2a**. Scale: 33 mg of **36**. (*E*)-4-(dimethylamine)but-2-enoic chloride used during this step was prepared according to the literature procedure.[4] Product **2d** was purified by preparative HPLC (gradient 5→20 min. 0→40% B, retention time 5.5 min.) followed by lyophilization from 0.1M HCl (repeated three times). Yield: 23% (9 mg). ^{1}**H NMR** (700 MHz, D_2O pH 2) δ 9.28 (s, $CH_{Ar(5)}$, 1H), 7.94 (dd, $^{3}J_{HH}$ = 9.7, $^{4}J_{HH}$=1.8, $C\underline{H}_{Ar}$, 1H), 7.91 (d, $^{3}J_{HH}$ = 9.6, $C\underline{H}_{Ar}$, 1H), 7.91 (s, $C\underline{H}_{Ar(2)}$, 1H), 6.96 (dt, $^{3}J_{HH}$ = 15.3, $^{3}J_{HH}$ = 7.2,

C$\underline{H}$CHC(O)NH, 1H), 6.65 (d, $^3J_{HH}$ = 15.3, CHC$\underline{H}$C(O)NH, 1H), 4.07 (dd, $^3J_{HH}$ = 7.2, J_{HH} = 1.3, C$\underline{H}_2$CHCHC(O)NH, 2H), 4.06 (ddd, $^3J_{FH}$ = 38.0, $^2J_{HH}$ = 16.5, $^3J_{PH}$ = 4.3, C$\underline{H}_2$C(F)P, 1H), 3.90 (ddd, $^2J_{HH}$ = 16.7, $^3J_{FH}$ = 10.3, $^3J_{PH}$ = 6.6, C$\underline{H}_2$C(F)P, 1H), 2.99 (s, N(CH$_3$)$_2$, 6H). **^{13}C NMR** (176 MHz, D_2O pH 2) δ 171.9 (d, $^2J_{FC}$ = 22.5, $\underline{C}O_2$, 1C), 165.1 (s, $\underline{C}$ONH, 1C), 137.7 (s, $\underline{C}_{Ar(9)}$, 1C), 132.6 (s, $\underline{C}$HCHC(O)NH, 1C), 131.5 (s, CH$\underline{C}$HC(O)NH, 1C), 128.9 (s, $\underline{C}_{Ar(6)}$, 1C), 128.9 (s, $\underline{C}H_{Ar}$, 1C), 122.1 (s, $\underline{C}H_{Ar(2)}$, 1C), 122.05 (d, $^3J_{PC}$ = 12.1, $\underline{C}_{Ar(3)}$, 1C), 118.4 (d, J_{FC} = 5.2, $\underline{C}H_{Ar(5)}$, 1C), 112.3 (s, $\underline{C}H_{Ar}$, 1C), 97.1 (dd, $^1J_{FC}$ = 192.9, $^1J_{PC}$ = 144.2, $\underline{C}$(F)P, 1C), 57.5 (s, $\underline{C}H_2$CHCHC(O)NH, 1C), 42.6 (s, N($\underline{C}H_3$)$_2$, 2C), 27.8 (dd, $^2J_{FC}$ = 20.4, $^2J_{PC}$ = 2.7, $\underline{C}H_2$C(F)P, 1C). **^{31}P NMR** (284 MHz, D_2O, pH 2) 6.79 (d, $^2J_{PF}$ = 72.3); HR-MS: m/z [$C_{16}H_{20}FN_4O_6P+H^+$] calculated 415.1177, found 415.1176.

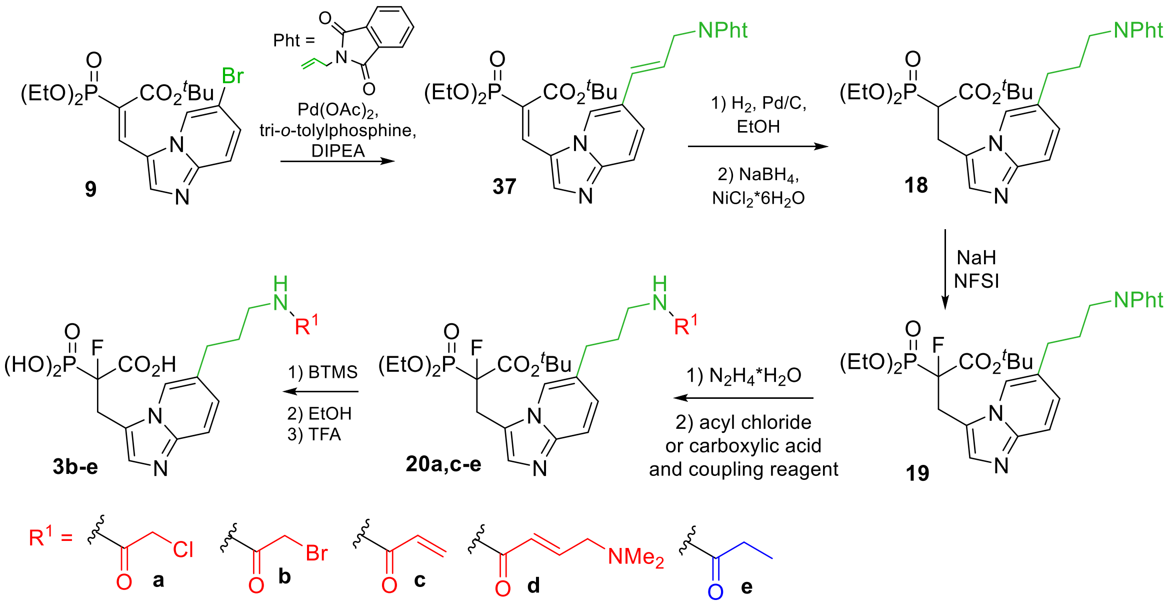

Scheme S8. Synthesis of compounds **3b-e.**

***Tert*-butyl 2-(diethoxyphosphoryl)-3-(6-((*E*)-3-(1,3-dioxoisoindolin-2-yl)prop-1-en-1-yl)imidazo[1,2-*a*]pyridin-3-yl)acrylate (37):** obtained according to the Mizoroki–Heck reaction procedure for compound **12a**, using 2-allylisoindoline-1,3-dione. Scale: 0.66 mmol (300 mg) of **9**. Compound **37** was purified by flash chromatography using Gilson PLC 2250 purification system. Eluent A: DCM and B: Acetone, to both of them TEA was added, 1mL/(1L of eluent) (gradient 15→45 min. 0→40%B, retention time 37 min.). Yield: 65% (84% purity according to ^{31}P NMR spectrum). **^{1}H NMR** (700 MHz, $CDCl_3$) δ 8.40 (s, C$\underline{H}_{Ar(2)}$, 1H), 8.18 (s, C$\underline{H}_{Ar(5)}$, 1H), 7.88 – 7.85 (m, C$\underline{H}_{Pht}$, 2H), 7.76 (d, $^3J_{PH}$ = 23.9, PCC$\underline{H}$, 1H), 7.74 – 7.71 (m, C$\underline{H}_{Pht}$, 2H), 7.59 (dd, $^3J_{HH}$ = 9.3, C$\underline{H}_{Ar(7)}$, 1H), 7.46 (dd, $^3J_{HH}$ = 9.4, $^4J_{HH}$=1.5, C$\underline{H}_{Ar(8)}$, 1H), 6.55 (d, $^3J_{HH}$ = 15.9, C$\underline{H}$CHCH$_2$NPht, 1H), 6.31 (dt, $^3J_{HH}$ = 15.8, $^3J_{HH}$ = 5.9, C$\underline{H}$CH$_2$NPht, 1H), 4.47 (dd, $^3J_{HH}$ = 5.9, $^4J_{HH}$ = 1.3, C$\underline{H}_2$NPht, 1H), 4.17 – 4.09 (m, C$\underline{H}_2$OP, 4H), 1.54 (s, C(C$\underline{H}_3$)$_3$, 9H), 1.34 (t, $^3J_{HH}$ = 7.1, C$\underline{H}_3$CH$_2$OP, 6H). **^{13}C NMR** (176 MHz, $CDCl_3$) δ 167.94 (s, $\underline{C}$ON, 2C), 165.09 (d, $^2J_{PC}$ = 11.1, $\underline{C}O_2t$Bu, 1C), 147.05 (s, $\underline{C}_{Ar(9)}$, 1C), 140.79 (s, $\underline{C}H_{Ar(2)}$, 1C), 134.24 (s, $\underline{C}H_{Pht}$, 2C), 132.16 (s, $\underline{C}CH_{Pht}$, 2C), 132.07 (d, $^2J_{PC}$ = 10.1, PC$\underline{C}$H, 1C), 128.08 (s, $C_{Ar(6)}\underline{C}H$, 1C), 125.34 (s, $\underline{C}$HCH$_2$NPht, 1C), 124.83 (s, $\underline{C}H_{Ar(8)}$, 1C), 124.18 (s, $\underline{C}_{Ar(6)}$, 1C), 123.59 (s, $\underline{C}H_{Pht}$, 2C), 122.14 (s, $\underline{C}H_{Ar(5)}$, 1C), 120.74 (d, $^3J_{PC}$ = 24.8, $\underline{C}_{Ar(3)}$, 1C), 118.43 (d, $^1J_{PC}$ = 182.0, P$\underline{C}$, 1C), 118.23 (s, $\underline{C}H_{Ar(7)}$, 1C), 82.72 (s, CO$_2\underline{C}$Me$_3$, 1C), 62.48 (d, $^2J_{PC}$ = 4.9, $\underline{C}H_2$OP, 2C), 39.28 (s, $\underline{C}H_2$NPht, 1C), 28.13 (s, C($\underline{C}H_3$)$_3$, 3C), 16.39 (d, $^3J_{PC}$ = 6.9, $\underline{C}H_3$CH$_2$OP, 2C). **^{31}P NMR** (101 MHz, $CDCl_3$) δ 16.69. HRMS ($C_{29}H_{32}N_3O_7P + H^+$) m/z: calculated 566.2051, found 566.2061.

***Tert*-butyl 2-(diethoxyphosphoryl)-3-(6-(3-(1,3-dioxoisoindolin-2-yl)propyl)imidazo[1,2-*a*] pyridin-3-yl)propanoate (18):** obtained according to the two-step reduction procedure for compound **12f**. Scale: 0.35 mmol (200 mg) of **37**. Compound **18** was purified by flash chromatography using Gilson PLC 2250 purification system. Eluent A: DCM and B: Acetone, to both of them TEA was added, 1mL/(1L of eluent) (gradient 0→30 min. 0→50%B, retention time 45 min.). Yield (two-step reduction): 83% (167 mg). **^{1}H NMR** (700 MHz, $CDCl_3$) δ 7.74 (s, C$\underline{H}_{Ar(5)}$, 1H), 7.68 – 7.65 (m, C$\underline{H}_{Pht}$, 2H), 7.57 – 7.54 (m, C$\underline{H}_{Pht}$, 2H), 7.34 (d, $^3J_{HH}$ = 9.2, C$\underline{H}_{Ar(8)}$, 1H), 7.26 (s, C$\underline{H}_{Ar(2)}$, 1H), 6.94 (dd, $^3J_{HH}$ = 9.3, $^4J_{HH}$=1.5, C$\underline{H}_{Ar(7)}$, 1H), 4.16 – 4.11 (m, C$\underline{H}_2$OP, 4H), 3.66 (t, $^3J_{HH}$ = 6.89, C$\underline{H}_2$NPht, 2H), 3.45 (ddd, $^2J_{HH}$ = 15.5, $^3J_{HH}$ = 11.9, $^3J_{PH}$ = 6.7, C$\underline{H}_2$C(H)P, 1H), 3.25 (ddd, $^2J_{PH}$ = 23.5, $^3J_{HH}$ = 12.0, $^3J_{HH}$ = 3.1, CH_2C($\underline{H}$)P, 1H), 3.22 (ddd, $^2J_{HH}$ = 15.3, $^3J_{PH}$ = 9.9, $^3J_{HH}$ = 3.0, C$\underline{H}_2$C(H)P, 1H), 2.63 – 2.57 (m, C$\underline{H}_2CH_2CH_2$NPht, 2H), 2.01 – 1.95 (m, C$\underline{H}_2CH_2$NPht, 2H), 1.29 (s, C(C$\underline{H}_3)_3$, 9H), 1.28 (t, $^3J_{HH}$ = 7.0, C$\underline{H}_3CH_2$OP, 6H). **^{13}C NMR** (176 MHz, $CDCl_3$ 77.16) δ 168.09 (s, $\underline{C}$ON, 2C), 167.06 (d, $^2J_{PC}$ = 5.1, $\underline{C}O_2$*t*Bu, 1C), 144.38 (s, $\underline{C}_{Ar(9)}$, 1C), 133.74 (s, $\underline{C}H_{Pht}$, 2C), 131.62 (s, $\underline{C}CH_{Pht}$, 2C), 131.20 (s, $\underline{C}H_{Ar(2)}$, 1C), 125.18 (s, $\underline{CH}_{Ar(7)}$, 1C), 124.76 (s, $\underline{C}_{Ar(6)}$, 1C), 122.81 (s, $\underline{C}H_{Pht}$, 2C), 120.90 (d, $^3J_{PC}$ = 19.4, $\underline{C}_{Ar(3)}$, 1C), 120.76 (s, $\underline{C}H_{Ar(5)}$, 1C), 117.25 (s, $\underline{C}H_{Ar(8)}$, 1C), 82.18 (s, $CO_2\underline{C}Me_3$, 1C), 62.86 – 62.58 (m, $\underline{C}H_2$OP, 2C), 44.75 (d, $^2J_{PC}$ = 128.9, $CH_2\underline{C}$(H)P, 1C), 37.10 (s, $\underline{C}H_2$NPht, 1C), 29.90 (s, $\underline{C}H_2CH_2CH_2$NPht, 1C), 28.75 (s, $\underline{C}H_2CH_2$NPht, 1C), 27.57 (s, C($\underline{C}H_3)_3$, 3C), 21.41 (d, $^3J_{PC}$ = 2.7, $\underline{C}H_2$C(H)P, 1C), 16.22 (d, $^3J_{PC}$ = 5.9, $\underline{C}H_3CH_2$OP, 1C), 16.20 (d, $^3J_{PC}$ = 6.1, $\underline{C}H_3CH_2$OP, 1C). **^{31}P NMR** (101 MHz, $CDCl_3$) δ 22.10.

***Tert*-butyl 2-(diethoxyphosphoryl)-3-(6-(3-(1,3-dioxoisoindolin-2-yl)propyl)imidazo[1,2-*a*]pyridin-3-yl)-2-fluoropropanoate (19):** obtained according to the *general procedure of fluorination*. Scale: 125 mg (0.22 mmol) of **18**. Compound **19** was purified by flash chromatography using Gilson PLC 2250 purification system. Eluents A:DCM, B:Acetone, to both of them TEA was added, 1mL/(1L of eluent) (gradient 0→20 min. 0→45% B, retention time 23 min.). Yield: 74% (95 mg). **^{1}H NMR** (700 MHz, $CDCl_3$) δ 7.92 (s, C$\underline{H}_{Ar(5)}$, 1H), 7.79 – 7.76 (m, C$\underline{H}_{Pht}$, 2H), 7.68 – 7.64 (m, C$\underline{H}_{Pht}$, 2H), 7.48 (d, $^3J_{HH}$ = 9.2, C$\underline{H}_{Ar(8)}$, 1H), 7.45 (s, C$\underline{H}_{Ar(2)}$, 1H), 7.05 (dd, $^3J_{HH}$ = 9.2, $^4J_{HH}$=1.6, C$\underline{H}_{Ar(7)}$, 1H), 4.29 – 4.20 (m, C$\underline{H}_2$OP, 4H), 3.82 (ddd, $^3J_{FH}$ = 37.7, $^2J_{HH}$ = 16.1, $^3J_{PH}$ = 5.8, C$\underline{H}_2$C(F)P, 1H), 3.74 (t, $^3J_{HH}$ = 6.9, C$\underline{H}_2$NPht, 2H), 3.66 (ddd, $^2J_{HH}$ = 16.1, $^3J_{FH}$ = 12.3, $^3J_{PH}$ = 6.5, C$\underline{H}_2$C(F)P, 1H), 2.70 – 2.62 (m, C$\underline{H}_2CH_2CH_2$NPht, 2H), 2.06 – 1.99 (m, C$\underline{H}_2CH_2$NPht, 2H), 1.36 – 1.32 (m, C$\underline{H}_3CH_2$OP, 6H), 1.35 (s, C(C$\underline{H}_3)_3$, 9H). **^{13}C NMR** (176 MHz, $CDCl_3$) δ 168.4 (s, $\underline{C}$ON, 2C), 165.0 (dd, $^2J_{FC}$ = 22.1, $^2J_{PC}$ = 3.8, $\underline{C}O_2$*t*Bu, 1C), 144.9 (s, $\underline{C}_{Ar(9)}$, 1C), 134.0 (s, $\underline{C}H_{Pht}$, 2C), 133.6 (s, $\underline{C}H_{Ar(2)}$, 1C), 132.0 (s, $\underline{C}CH_{Pht}$, 2C), 126.2 (s, $\underline{CH}_{Ar(7)}$, 1C), 125.3 (s, $\underline{C}_{Ar(6)}$, 1C), 123.2 (s, $\underline{C}H_{Pht}$, 2C), 121.8 (d, J_{FC} = 4.7, $\underline{C}H_{Ar(5)}$, 1C), 117.3 (s, $\underline{C}H_{Ar(8)}$, 1C), 116.8 (d, $^3J_{PC}$ = 15.0, $\underline{C}_{Ar(3)}$, 1C), 95.7 (dd, $^1J_{FC}$ = 199.6, $^1J_{PC}$ = 159.6, $\underline{C}$(F)P, 1C), 84.5 (s, $CO_2\underline{C}Me_3$, 1C), 64.6 (d, $^2J_{PC}$ = 6.4, $\underline{C}H_2$OP, 1C), 64.3 (d, $^2J_{PC}$ = 7.0, $\underline{C}H_2$OP, 1C), 37.4 (s, $\underline{C}H_2$NPht, 1C), 30.2 (s, $\underline{C}_{Ar(6)}\underline{C}H_2$, 1C), 29.3 (s, $\underline{C}H_2CH_2$NPht, 1C), 28.3 (d, $^2J_{FC}$ = 21.1, $\underline{C}H_2$C(F)P, 1C), 27.8 (s, C($\underline{C}H_3)_3$, 3C), 16.4 (d, $^3J_{PC}$ = 5.6, $\underline{C}H_3CH_2$OP, 1C). **^{31}P NMR** (101 MHz, $CDCl_3$) δ 12.58 (d, $^2J_{PF}$ = 83.1).

***Tert*-butyl 3-(6-(3-(2-chloroacetamido)propyl)imidazo[1,2-*a*]pyridin-3-yl)-2-(diethoxyphosphoryl)-2-fluoropropanoate (20a):** obtained according to the *general procedure of coupling reaction* for synthesis of compounds **20** using chloroacetic chloride. Scale: 95 mg (0.16 mmol) of **19**. Due to decomposition of **20a** during its purification using column chromatography, crude product **20a** was used in the next step of ester deprotection. Yield of crude product **20a**: 99% (113 mg). **^{1}H NMR** (700 MHz, $CDCl_3$) δ 7.96 (s, C$\underline{H}_{Ar(5)}$, 1H), 7.55 (d, $^3J_{HH}$ = 9.2, C$\underline{H}_{Ar(8)}$, 1H), 7.47 (s, C$\underline{H}_{Ar(2)}$, 1H), 7.09 (dd, $^3J_{HH}$ = 9.2, $^4J_{HH}$=1.6, C$\underline{H}_{Ar(7)}$, 1H), 6.98 (bt, $^3J_{HH}$ = 6.0, CH_2N$\underline{H}$, 1H), 4.28 – 4.15 (m, C$\underline{H}_2$OP, 4H), 4.00 (s, OCC$\underline{H}_2$Cl, 1H), 3.82 (ddd, $^3J_{FH}$ = 36.4, $^2J_{HH}$ = 16.2, $^3J_{PH}$ = 6.7, C$\underline{H}_2$C(F)P, 1H), 3.67 (ddd, $^2J_{HH}$ = 16.1, $^3J_{FH}$ = 12.9, $^3J_{PH}$ = 6.6, C$\underline{H}_2$C(F)P, 1H), 3.37 – 3.25 (m, C$\underline{H}_2$NH, 2H), 2.65 (t, $^3J_{HH}$ = 7.5, $C_{Ar(6)}$C$\underline{H}_2$, 1H), 1.93 – 1.83 (m, $C_{Ar(6)}CH_2$C$\underline{H}_2$, 2H), 1.35 (s, C(C$\underline{H}_3)_3$,

9H), 1.34 (t, $^3J_{HH}$ = 6.0, $C\underline{H}_3CH_2OP$, 3H), 1.30 (t, $^3J_{HH}$ = 7.1, $C\underline{H}_3CH_2OP$, 3H). **^{13}C NMR** (176 MHz, $CDCl_3$) δ 166.3 (s, $\underline{C}ONH$, 1C), 165.0 (dd, $^2J_{FC}$ = 22.2, $^2J_{PC}$ = 3.8, $\underline{C}O_2t$Bu, 1C), 144.4 (s, $\underline{C}_{Ar(9)}$, 1C), 132.5 (s, $\underline{C}H_{Ar(2)}$, 1C), 127.2 (s, $\underline{C}H_{Ar(7)}$, 1C), 126.0 (s, $\underline{C}_{Ar(6)}$, 1C), 122.1 (d, J_{FC} = 4.9, $\underline{C}H_{Ar(5)}$, 1C), 117.1 (d, $^3J_{PC}$ = 14.6, $\underline{C}_{Ar(3)}$, 1C), 116.9 (s, $\underline{C}H_{Ar(8)}$, 1C), 95.6 (dd, $^1J_{FC}$ = 199.4, $^1J_{PC}$ = 160.1, $\underline{C}$(F)P, 1C), 84.7 (s, $CO_2\underline{C}Me_3$, 1C), 64.7 (d, $^2J_{PC}$ = 6.8, $\underline{C}H_2OP$, 1C), 64.4 (d, $^2J_{PC}$ = 7.0, $\underline{C}H_2OP$, 1C), 42.7 (s, $\underline{C}H_2Cl$, 1C), 39.0 (s, $\underline{C}H_2NH$, 1C), 30.1 (s, $\underline{C}H_2CH_2NH$, 1C), 30.0 (s, $C_{Ar(6)}\underline{C}H_2$, 1C), 28.3 (d, $^2J_{FC}$ = 20.6, $\underline{C}H_2C(F)P$, 1C), 27.8 (s, $C(\underline{C}H_3)_3$, 3C), 16.5 (d, $^3J_{PC}$ = 5.6, $\underline{C}H_3CH_2OP$, 1C), 16.4 (d, $^3J_{PC}$ = 5.7, $\underline{C}H_3CH_2OP$, 1C). **^{31}P NMR** (101 MHz, $CDCl_3$) δ 12.50 (d, $^2J_{PF}$ = 83.5).

***Tert*-butyl 3-(6-(3-acrylamidopropyl)imidazo[1,2-*a*]pyridin-3-yl)-2-(diethoxyphosphoryl)-2-fluoropropanoate (20c):** obtained according to the *general procedure of coupling reaction* for synthesis of compounds **20** using acrylic chloride. Scale: 100 mg (0.17 mmol) of **19**. Compound **20c** was purified by flash chromatography using Gilson PLC 2250 purification system. Eluents A:DCM, B:Acetone, to both of them TEA was added, 1mL/(1L of eluent) (gradient 0→15 min. 0→50% B, retention time 35 min.). Yield: 80% (70 mg). **^{1}H NMR** (700 MHz, $CDCl_3$) δ 8.02 (s, $C\underline{H}_{Ar(5)}$, 1H), 7.55 (d, $^3J_{HH}$ = 9.2, $C\underline{H}_{Ar(8)}$, 1H), 7.53 (s, $C\underline{H}_{Ar(2)}$, 1H), 7.09 (dd, $^3J_{HH}$ = 9.2, $^4J_{HH}$=1.7, $C\underline{H}_{Ar(7)}$, 1H), 6.50 (bt, $^3J_{HH}$ = 5.3, $CH_2N\underline{H}$, 1H), 6.32 (dd, $^3J_{HH(trans)}$ = 17.0, $^2J_{HH}$ = 1.5, $OCCHC\underline{H}_2$, 1H), 6.20 (dd, $^3J_{HH(trans)}$ = 17.0, $^3J_{HH(cis)}$ = 10.3, $OCC\underline{H}CH_2$, 1H), 5.64 (dd, $^3J_{HH(cis)}$ = 10.2, $^3J_{HH}$ = 1.5, $OCCHC\underline{H}_2$, 1H), 4.35 – 4.15 (m, $C\underline{H}_2OP$, 4H), 3.88 (ddd, $^3J_{FH}$ = 34.9, $^2J_{HH}$ = 16.2, $^3J_{PH}$ = 8.2, $C\underline{H}_2C(F)P$, 1H), 3.75 (ddd, $^2J_{HH}$ = 16.0, $^3J_{FH}$ = 15.5, $^3J_{PH}$ = 6.5, $C\underline{H}_2C(F)P$, 1H), 3.43 (ddt, $^2J_{HH}$ = 13.0, $^3J_{HH}$ = 6.4, $C\underline{H}_2NH$, 1H), 3.31 (ddt, $^2J_{HH}$ = 13.4, $^3J_{HH}$ =6.9, $C\underline{H}_2NH$, 1H), 2.71 (t, $^3J_{HH}$ = 7.5, $C_{Ar(6)}C\underline{H}_2$, 1H), 1.99 (dtt, $^2J_{HH}$ = 14.1, $^3J_{HH}$ = 6.9, $C_{Ar(6)}CH_2C\underline{H}_2$, 1H), 1.93 (dtt, $^2J_{HH}$ = 14.1, $^3J_{HH}$ = 7.2, $C_{Ar(6)}CH_2C\underline{H}_2$, 1H), 1.44 (s, $C(C\underline{H}_3)_3$, 9H), 1.41 (t, $^3J_{HH}$ = 7.1, $C\underline{H}_3CH_2OP$, 3H), 1.34 (t, $^3J_{HH}$ = 7.1, $C\underline{H}_3CH_2OP$, 1H). **^{13}C NMR** (176 MHz, $CDCl_3$) δ 166.0 (s, $\underline{C}ONH$, 1C), 165.3 (dd, $^2J_{FC}$ = 22.2, $^2J_{PC}$ = 3.9, $\underline{C}O_2t$Bu, 1C), 145.2 (s, $\underline{C}_{Ar(9)}$, 1C), 134.0 (s, $\underline{C}H_{Ar(2)}$, 1C), 131.1 (s, $OC\underline{C}H{=}CH_2$, 1C), 126.3 (s, $\underline{C}H_{Ar(7)}$, 1C), 126.2 (s, $OCCH{=}\underline{C}H_2$, 1C), 125.4 (s, $\underline{C}_{Ar(6)}$, 1C), 122.1 (d, J_{FC} = 4.6, $\underline{C}H_{Ar(5)}$, 1C), 117.4 (s, $\underline{C}H_{Ar(8)}$, 1C), 116.6 (d, $^3J_{PC}$ = 13.6, $\underline{C}_{Ar(3)}$, 1C), 95.6 (dd, $^1J_{FC}$ = 199.5, $^1J_{PC}$ = 160.6, $\underline{C}$(F)P, 1C), 84.7 (s, $CO_2\underline{C}Me_3$, 1C), 64.8 (d, $^2J_{PC}$ = 6.9, $\underline{C}H_2OP$, 1C), 64.4 (d, $^2J_{PC}$ = 7.4, $\underline{C}H_2OP$, 1C), 38.5 (s, $\underline{C}H_2NH$, 1C), 30.1 (s, $\underline{C}H_2CH_2NH$, 1C), 29.9 (s, $\underline{C}H_2CH_2CH_2NH$, 1C), 28.4 (d, $^2J_{FC}$ = 21.2, $\underline{C}H_2C(F)P$, 1C), 27.9 (s, $C(\underline{C}H_3)_3$, 3C), 16.5 (d, $^3J_{PC}$ = 5.7, $\underline{C}H_3CH_2OP$, 1C), 16.5 (d, $^3J_{PC}$ = 5.7, $\underline{C}H_3CH_2OP$, 1C). **^{31}P NMR** (101 MHz, $CDCl_3$) δ 13.00 (d, $^2J_{PF}$ = 83.4).

***Tert*-butyl (*E*)-2-(diethoxyphosphoryl)-3-(6-(3-(4-(dimethylamino)but-2-enamido)propyl)imidazo[1,2-*a*]pyridin-3-yl)-2-fluoropropanoate (20d):** obtained accourding to the modified procedure from Zhao et al.[5] To the cooled solution of HATU (*N*-[(dimethylamino)-1*H*-1,2,3-triazolo-[4,5-*b*]pyridin-1-ylmethylene]-*N*-methylmethanaminium hexafluorophosphate *N*-oxide, 1.1 eq. 0.28 mmol, 107 mg) in DMF (1 mL), (*E*)-4-(dimethylamino)but-2-enoic acid (1.1 eq. 47 mg) and DIPEA (1.1 eq., 47 μL) were added. After 5 minutes of stiring a mixture of DIPEA (2 eq., 89 μL) and amine derivative (obtained from compound **19** (150 mg, 0.26 mmol) in reaction with hydrazine hydrate) in DMF (3 mL) was added. Reaction was mixed at rt overnight. Then, the reaction was quenched by addition of H_2O (3 mL). The product **20d** was extracted with $CHCl_3$ (3x5 mL, pH 9). Combined organic phases were dried over $MgSO_4$ and concentrated. Yield: 76% (110 mg). **^{1}H NMR** (250 MHz, $CDCl_3$) δ 7.88 (s, $C\underline{H}_{Ar(5)}$, 1H), 7.38 (d, $^3J_{HH}$ = 9.2, $C\underline{H}_{Ar(8)}$, 1H), 7.37 (s, $C\underline{H}_{Ar(2)}$, 1H), 7.05 (bt, $^3J_{HH}$ = 5.9, $CH_2N\underline{H}$, 1H), 6.96 (dd, $^3J_{HH}$ = 9.2, $^4J_{HH}$=1.6, $C\underline{H}_{Ar(7)}$, 1H), 6.71 (dt, $^3J_{HH}$ = 15.4, $^4J_{HH}$ = 6.4, $OCCH{=}C\underline{H}$, 1H), 6.05 (d, $^3J_{HH}$ = 15.4, $OCC\underline{H}{=}CH$, 1H), 4.23 – 4.08 (m, $C\underline{H}_2OP$, 4H), 3.75 (ddd, $^3J_{FH}$ = 36.4, $^2J_{HH}$ = 16.2, $^3J_{PH}$ = 6.9, $C\underline{H}_2C(F)P$, 1H), 3.61 (ddd, $^2J_{HH}$ = 16.0, $^3J_{FH}$ = 13.2, $^3J_{PH}$ = 6.7, $C\underline{H}_2C(F)P$, 1H), 3.31 – 3.17 (m, $C\underline{H}_2NH$, 2H), 3.07 (dd, $^3J_{HH}$ = 6.4, $^4J_{HH}$ = 1.5, $CH{=}CHC\underline{H}_2$, 2H), 2.57 (t, $^3J_{HH}$ = 7.6, $C_{Ar(6)}C\underline{H}_2$, 1H), 2.23 (s, $N(CH_3)_2$, 6H), 1.86 – 1.72 (m, $C_{Ar(6)}CH_2C\underline{H}_2$, 2H), 1.30 (s, $C(C\underline{H}_3)_3$, 9H), 1.29 (t, $^3J_{HH}$ = 7.0, $C\underline{H}_3CH_2OP$, 3H), 1.24 (t, $^3J_{HH}$ = 7.1, $C\underline{H}_3CH_2OP$, 1H). **^{13}C NMR**

(176 MHz, $CDCl_3$) δ 165.65 (s, $\underline{C}$ONH, 1C), 165.0 (dd, $^2J_{FC}$ = 22.7, $^2J_{PC}$ = 3.6, $\underline{C}O_2t$Bu, 1C), 145.1 (s, $\underline{C}_{Ar(9)}$, 1C), 138.2 (s, OCCH=C$\underline{H}$, 1C), 133.8 (s, $\underline{C}H_{Ar(2)}$, 1C), 127.2 (s, OCC$\underline{H}$=CH, 1C), 126.2 (s, $\underline{C}H_{Ar(7)}$, 1C), 125.5 (s, $\underline{C}_{Ar(6)}$, 1C), 121.8 (d, J_{FC} = 5.0, $\underline{C}H_{Ar(5)}$, 1C), 117.1 (s, $\underline{C}H_{Ar(8)}$, 1C), 116.5 (d, $^3J_{PC}$=14.5, $\underline{C}_{Ar(3)}$, 1C), 95.6 (dd, $^1J_{FC}$ = 198.6, $^1J_{PC}$ = 160.2, $\underline{C}$(F)P, 1C), 84.5 (s, $CO_2\underline{C}Me_3$, 1C), 64.6 (d, $^2J_{PC}$ = 6.7, $\underline{C}H_2OP$, 1C), 64.3 (d, $^2J_{PC}$ = 7.4, $\underline{C}H_2OP$, 1C), 59.8 (s, CH=CHC$\underline{H}_2$N, 1C), 44.8 (s, $N(CH_3)_2$, 1C), 38.5 (s, $\underline{C}H_2NH$, 1C), 30.2 (s, $\underline{C}H_2CH_2NH$, 1C), 29.9 (s, $C_{Ar(6)}\underline{C}H_2$, 1C), 28.2 (d, $^2J_{FC}$ = 21.3, $\underline{C}H_2C(F)P$, 1C), 27.7 (s, $C(\underline{C}H_3)_3$, 3C), 16.4 (d, $^3J_{PC}$ = 6.2, $\underline{C}H_3CH_2OP$, 1C), 16.3 (d, $^3J_{PC}$ = 5.3, $\underline{C}H_3CH_2OP$, 1C). **^{31}P NMR** (101 MHz, $CDCl_3$) δ 12.60 (d, $^2J_{PF}$ = 83.2).

***Tert*-butyl 2-(diethoxyphosphoryl)-2-fluoro-3-(6-(3-propionamidopropyl)imidazo[1,2-*a*]pyridin-3-yl)propanoate (20e):** obtained according to the *general procedure of coupling reaction* for synthesis of compounds **20** using propionyl chloride. Scale: 110 mg (0.19 mmol) of **19**. Compound **20e** was purified by flash chromatography using Gilson PLC 2250 purification system. Eluents A:DCM, B:Acetone, to both of them TEA was added, 1mL/(1L of eluent) (gradient 0→15 min. 0→50% B, retention time 33 min.). Yield: 63% (60 mg). **^{1}H NMR** (250 MHz, $CDCl_3$) δ 7.89 (s, C$\underline{H}_{Ar(5)}$, 1H), 7.43 (d, $^3J_{HH}$ = 8.5, C$\underline{H}_{Ar(8)}$, 2H), 7.41 (s, C$\underline{H}_{Ar(2)}$, 1H), 6.99 (dd, $^3J_{HH}$ = 9.3, $^4J_{HH}$=1.5, C$\underline{H}_{Ar(7)}$, 1H), 6.32 (bd, $^3J_{HH}$ = 7.8, $CH_2N\underline{H}$, 1H), 4.29 – 4.09 (m, C$\underline{H}_2$OP, 4H), 3.95 – 3.51 (m, C$\underline{H}_2$C(F)P, 2H), 3.35 – 3.11 (m, C$\underline{H}_2$NH, 2H), 2.59 (t, $^3J_{HH}$ = 7.5, $C_{Ar(6)}C\underline{H}_2$, 2H), 2.15 (q, $^3J_{HH}$ = 7.6, C$\underline{H}_2CH_3$, 2H), 1.90 – 1.70 (m, $C_{Ar(6)}CH_2C\underline{H}_2$, 2H), 1.33 (s, $C(C\underline{H}_3)_3$, 9H), 1.37 – 1.20 (m, $C\underline{H}_3CH_2OP$, 6H), 1.08 (t, $^3J_{HH}$ = 7.6, $CH_2C\underline{H}_3$, 3H). **^{13}C NMR** (63 MHz, $CDCl_3$) δ 174.2 (s, $\underline{C}$ONH, 1C), 165.0 (dd, $^2J_{FC}$ = 22.5, $^2J_{PC}$ = 4.0, $\underline{C}O_2t$Bu, 1C), 145.0 (s, $\underline{C}_{Ar(9)}$, 1C), 133.7 (s, $\underline{C}H_{Ar(2)}$, 1C), 126.3 (s, $\underline{C}H_{Ar(7)}$, 1C), 125.6 (s, $\underline{C}_{Ar(6)}$, 1C), 121.8 (d, J_{FC} = 4.9, $\underline{C}H_{Ar(5)}$, 1C), 117.2 (s, $\underline{C}H_{Ar(8)}$, 1C), 116.6 (d, $^3J_{PC}$ = 14.5, $\underline{C}_{Ar(3)}$, 1C), 95.6 (dd, $^1J_{FC}$ = 198.7, $^1J_{PC}$ = 159.8, $\underline{C}$(F)P, 1C), 84.5 (s, $CO_2\underline{C}Me_3$, 1C), 64.6 (d, $^2J_{PC}$ = 6.2, $\underline{C}H_2OP$, 1C), 64.3 (d, $^2J_{PC}$ = 7.4, $\underline{C}H_2OP$, 1C), 38.5 (s, $\underline{C}H_2NH$, 1C), 30.4 (s, $\underline{C}H_2CH_2NH$, 1C), 30.0 (s, $C_{Ar(6)}\underline{C}H_2$, 1C), 29.6 (s, $\underline{C}H_2CH_3$, 1C), 28.3 (dd, $^2J_{FC}$ = 20.9, $^2J_{PC}$ = 2.0, $\underline{C}H_2C(F)P$, 1C), 27.7 (s, $C(\underline{C}H_3)_3$, 3C), 16.7 – 16.2 (m, $\underline{C}H_3CH_2OP$, 2C), 10.0 (s, $CH_2\underline{C}H_3$, 1C). **^{31}P NMR** (101 MHz, $CDCl_3$) δ 12.99 (d, $^2J_{PF}$ = 83.6).

3-(6-(3-(2-bromoacetamido)propyl)imidazo[1,2-*a*]pyridin-3-yl)-2-fluoro-2-phosphonopropanoic acid (3b): obtained according to the general procedure of ester deprotection. Scale: 113 mg of **20a**. Product **3b** was purified by preparative HPLC (gradient 5→20 min. 0→40% B, retention time 9.3 min.) followed by lyophilization. Yield: 36% (27 mg). In the ^{13}C NMR spectrum of **3b** signals from $\underline{C}F_3\underline{C}O_2^-$ are present. **^{1}H NMR** (700 MHz, D_2O pH 2) δ 8.57 (s, C$\underline{H}_{Ar(5)}$, 1H), 7.81 – 7.77 (m, C$\underline{H}_{Ar(7)}$, C$\underline{H}_{Ar(8)}$, 2H), 7.71 (s, C$\underline{H}_{Ar(2)}$, 1H), 3.98 (ddd, $^3J_{FH}$ = 39.0, $^2J_{HH}$ = 16.2, $^3J_{PH}$ = 3.0, C$\underline{H}_2$C(F)P, 1H), 3.89 (s, OCC$\underline{H}_2$Br, 1H), 3.74 (ddd, $^2J_{HH}$ = 15.6, $^3J_{FH}$ = 7.5, $^3J_{PH}$ = 7.5, C$\underline{H}_2$C(F)P, 1H), 3.33 (t, $^3J_{HH}$ = 6.7, C$\underline{H}_2$NH, 2H), 2.88 (t, $^3J_{HH}$ = 7.5, $C_{Ar(6)}C\underline{H}_2$, 1H), 2.02 (tt, $^3J_{HH}$ = 7.0 $C_{Ar(6)}CH_2C\underline{H}_2$, 2H). **^{13}C NMR** (176 MHz, D_2O pH 2) δ 174.3 (d, $^2J_{FC}$ = 19.8, $\underline{C}O_2H$, 1C), 169.8 (s, $\underline{C}$ONH, 1C), 139.3 (s, $\underline{C}_{Ar(9)}$, 1C), 134.3 (s, $\underline{C}H_{Ar}$, 1C), 130.5 (s, $\underline{C}H_{Ar(6)}$, 1C), 124.5 (d, J_{FC} = 4.0, $\underline{C}H_{Ar(5)}$, 1C), 122.3 (d, $^3J_{PC}$ = 14.6, $\underline{C}_{Ar(3)}$, 1C), 121.4 (s, $\underline{C}H_{Ar(2)}$, 1C), 111.8 (s, $\underline{C}H_{Ar(8)}$, 1C), 98 (m, $\underline{C}$(F)P, 1C, in HMBC spectrum), 39.2 (s, $\underline{C}H_2NH$, 1C), 28.7 (s, $\underline{C}H_2CH_2NH$, 1C), 29.2 (s, $C_{Ar(6)}\underline{C}H_2$, 1C), 28.4 (d, $^2J_{FC}$ = 21.0, $\underline{C}H_2C(F)P$, 1C), 28.1 (s, $\underline{C}H_2Br$, 1C). **^{31}P NMR** (101 MHz, D_2O pH 2) δ 6.79 (d, $^2J_{PF}$ = 71.3). HR-MS: m/z [M+H$^+$] calculated 466.0173 and 468.0153, found 466.0169 and 468.0145.

3-(6-(3-acrylamidopropyl)imidazo[1,2-*a*]pyridin-3-yl)-2-fluoro-2-phosphonopropanoic acid (3c): obtained according to the general procedure of ester deprotection. Reaction with BTMS carried out in presence of TEA (10 eq). Scale: 29 mg of **20c**. Product **3c** was purified by preparative HPLC (gradient 5→20 min. 0→40% B, retention time 9.0 min.) followed by lyophilization. Yield: 42% (9 mg). In the ^{13}C NMR spectrum of **3c** signals from $\underline{C}F_3\underline{C}O_2^-$ are present. **^{1}H NMR** (700 MHz, D_2O pH 3) δ 8.56 (s, C$\underline{H}_{Ar(5)}$, 1H), 7.84 (dd, $^3J_{HH}$ = 9.3, $^3J_{HH}$ =1.4, C$\underline{H}_{Ar(7)}$, 1H), 7.79 (d, $^3J_{HH}$ = 9.2, C$\underline{H}_{Ar(8)}$, 1H), 7.77 (s,

$CH_{Ar(2)}$, 1H), 6.20 (dd, $^3J_{HH}$ = 17.1, 10.3, $CH{=}CH_2$, 1H), 6.12 (dd, $^3J_{HH}$ = 17.2, $^2J_{HH}$ = 1.3, $CH{=}CH_2$, 1H), 5.71 (dd, $^3J_{HH}$ = 10.3, $^2J_{HH}$ = 1.3, $CH{=}CH_2$, 1H), 4.01 (ddd, $^3J_{FH}$ = 38.3, $^2J_{HH}$ = 16.3, $^3J_{PH}$ = 3.5, $CH_2C(F)P$, 1H), 3.80 (ddd, $^2J_{HH}$ = 16.1, $^3J_{FH/PH}$ = 8.0, $CH_2C(F)P$, 1H), 3.40 – 3.31 (m, CH_2NH, 2H), 2.87 (t, $^3J_{HH}$ = 7.5, $C_{Ar(6)}CH_2$, 2H), 2.00 (tt, $^3J_{HH}$ = 7.0, CH_2CH_2NH, 2H). **^{13}C NMR** (176 MHz, D_2O pH 3) δ 173.0 (d, $^2J_{FC}$ = 21.4, CO_2H, 1C), 168.3 (s, CONH, 1C), 138.8 (s, $C_{Ar(9)}$, 1C), 135.1 (s, $CH_{Ar(7)}$, 1C), 131.1 (s, $C_{Ar(6)}$, 1C), 129.9 (s, $CH{=}CH_2$, 1C), 127.0 (s, $CH{=}CH_2$, 1C), 124.4 (d, J_{FC} = 4.0, $CH_{Ar(5)}$, 1C), 121.7 (d, $^3J_{PC}$ = 14.9, $C_{Ar(3)}$, 1C), 120.9 (s, $CH_{Ar(2)}$, 1C), 111.5 (s, $CH_{Ar(8)}$, 1C), 97.6 (dd, $^1J_{FC}$ = 193.4, $^1J_{PC}$ = 147.8, C(F)P, 1C), 38.6 (s, CH_2NH, 1C), 29.2 (s, $C_{Ar(6)}CH_2$, 1C), 28.9 (s, CH_2CH_2NH, 1C), 27.9 (dd, $^2J_{FC}$ = 20.9, $^2J_{PC}$ = 3.3, $CH_2C(F)P$, 1C). **^{31}P NMR** (283 MHz, D_2O, pH 3) δ 8.82 (d, $^2J_{PF}$ = 75.4). HR-MS: m/z [M+H$^+$] calculated 400.1068, found 400.1068.

(*E*)-3-(6-(3-(4-(dimethylamino)but-2-enamido)propyl)imidazo[1,2-*a*]pyridin-3-yl)-2-fluoro-2-phosphonopropanoic acid (3d): obtained according to the general procedure of ester deprotection. Reaction with BTMS carried out in presence of TEA (10 eq). Scale: 29 mg of **20d**. Product **3d** was purified by preparative HPLC (gradient 5→20 min. 0→40% B, retention time 9.0 min.) followed by lyophilization. Yield: 42% (9 mg). In the ^{13}C NMR spectrum of **3d** signals from $CF_3CO_2^-$ are present. **^{1}H NMR** (700 MHz, D_2O pH 2) δ 8.55 (s, $CH_{Ar(5)}$, 1H), 7.89 (dd, $^3J_{HH}$ = 9.3, $^4J_{HH}$ = 1.4, $CH_{Ar(7)}$, 1H), 7.84 (d, $^3J_{HH}$ = 9.8, $CH_{Ar(8)}$, 1H), 7.83 (s, $CH_{Ar(2)}$, 1H), 6.70 (dt, $^3J_{HH}$ = 15.5, $^4J_{HH}$ = 7.3, OCCH=CH, 1H), 6.40 (d, $^3J_{HH}$ = 15.4, OCCH=CH, 1H), 4.04 (ddd, $^3J_{FH}$ = 38.2, $^2J_{HH}$ = 16.4, $^3J_{PH}$ = 3.7, $CH_2C(F)P$, 1H), 3.95 (dd, $^3J_{HH}$ = 7.4, $^4J_{HH}$ = 1.3, $CH{=}CHCH_2$, 2H), 3.87 (ddd, $^2J_{HH}$ = 16.4, $^3J_{FH}$ = 9.5, $^3J_{PH}$ = 6.9, $CH_2C(F)P$, 1H), 3.35 (t, $^3J_{HH}$ = 6.6, CH_2NH, 2H), 2.93 (s, $N(CH_3)_2$, 6H), 2.89 (t, $^3J_{HH}$ = 7.4, $C_{Ar(6)}CH_2$, 1H), 2.03 (tt, $^3J_{HH}$ = 7.1, $C_{Ar(6)}CH_2CH_2$, 2H). **^{13}C NMR** (176 MHz, D_2O pH 2**)** δ 172.1 (d, $^2J_{FC}$ = 23.3, CO_2H, 1C), 166.4 (s, CONH, 1C), 139.0 (s, $C_{Ar(9)}$, 1C), 135.2 (s, $CH_{Ar(7)}$, 1C), 132.0 (s, OCCH=CH, 1C), 131.0 (s, $C_{Ar(6)}$, 1C), 130.2 (s, OCCH=CH, 1C), 124.6 (d, J_{FC} = 4.3, $CH_{Ar(5)}$, 1C), 121.2 (d, $^3J_{PC}$=14.1, $C_{Ar(3)}$, 1C), 121.1 (s, $CH_{Ar(2)}$, 1C), 111.6 (s, $CH_{Ar(8)}$, 1C), 97.1 (dd, $^1J_{FC}$ = 192.7, $^1J_{PC}$ = 144.5, C(F)P, 1C), 57.5 (s, $CH{=}CHCH_2N$, 1C), 42.5 (s, $N(CH_3)_2$, 1C), 38.43 (s, CH_2NH, 1C), 28.9 (s, $C_{Ar(6)}CH_2$, 1C), 28.6 (s, CH_2CH_2NH, 1C), 27.7 (dd, $^2J_{FC}$ = 20.7, $^2J_{FC}$ = 3.3, $CH_2C(F)P$, 1C). **^{31}P NMR** (284 MHz, D_2O, pH 2) δ 8.16 (d, $^2J_{PF}$ = 71.2); HR-MS: m/z [M+H$^+$] calculated 457.1647, found 457.1644.

2-fluoro-2-phosphono-3-(6-(3-propionamidopropyl)imidazo[1,2-a]pyridin-3-yl)propanoic acid (3e): obtained according to the general procedure of ester deprotection. Scale: 77 mg of **20e**. Product **3e** was purified by preparative HPLC (gradient 5→20 min. 0→40% B, retention time 9.3 min.) followed by lyophilization. Yield: 78% (47 mg). In the ^{13}C NMR spectrum of **3e** signals from $CF_3CO_2^-$ are present. **^{1}H NMR** (700 MHz, D_2O pH 3) δ 8.57 (s, $CH_{Ar(5)}$, 1H), 7.88 (d, $^3J_{HH}$ = 9.0, $CH_{Ar(7)}$, 1H), 7.85 (s, $CH_{Ar(2)}$, 1H), 7.84 (d, $^3J_{HH}$ = 8.8, $CH_{Ar(8)}$, 1H), 4.06 (ddd, $^3J_{FH}$ = 38.1, $^2J_{HH}$ = 16.5, $^3J_{PH}$ = 4.1, $CH_2C(F)P$, 1H), 3.90 (ddd, $^2J_{HH}$ = 16.7, $^3J_{FH}$ = 10.1, $^3J_{PH}$ = 6.8, $CH_2C(F)P$, 1H), 3.26 (t, $^3J_{HH}$ = 6.8, CH_2NH, 2H), 2.87 (t, $^3J_{HH}$ = 7.6, $C_{Ar(6)}CH_2$, 2H), 2.24 (q, $^3J_{HH}$ = 7.7, CH_2CH_3, 2H), 1.96 (tt, $^3J_{HH}$ = 7.1, CH_2CH_2NH, 2H), 1.10 (t, $^3J_{HH}$ = 7.7, CH_2CH_3, 2H). **^{13}C NMR** (176 MHz, D_2O pH 3) δ 178.0 (s, CONH, 1C), 171.7 (d, $^2J_{FC}$ = 23.4, CO_2H, 1C), 139.1 (s, $C_{Ar(9)}$, 1C), 135.4 (s, $CH_{Ar(7)}$, 1C), 131.3 (s, $C_{Ar(6)}$, 1C), 124.4 (d, J_{FC} = 3.6, $CH_{Ar(5)}$, 1C), 121.2 (s, $CH_{Ar(2)}$, 1C), 121.0 (d, $^3J_{PC}$=13.5, $C_{Ar(3)}$, 1C), 111.6 (s, $CH_{Ar(8)}$, 1C), 96.9 (dd, $^1J_{FC}$ = 193, $^1J_{PC}$ = 144, C(F)P, 1C), 38.4 (s, CH_2NH, 1C), 29.16 and 29.11 and 29.07 (3s, $CH_2CH_2CH_2NH$, CH_2CH_3, 3C), 27.5 (dd, $^2J_{FC}$ = 20.3, $^2J_{PC}$ = 2.8, $CH_2C(F)P$, 1C), 9.6 (s, CH_2CH_3,1C). **^{31}P NMR** (101 MHz, D_2O, pH 2) δ 7.65 (d, $^2J_{PF}$ = 71.7); HR-MS: m/z [M+H$^+$] calculated 402.1225, found 402.1226.

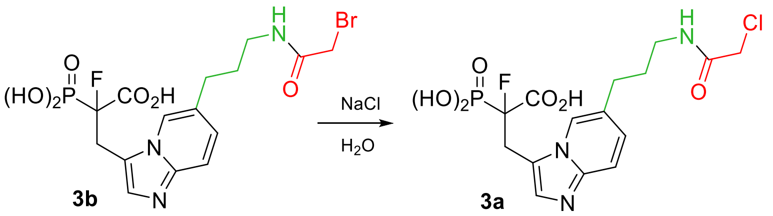

Scheme S9. Synthesis of compounds **3a.**

3-(6-(3-(2-chloroacetamido)propyl)imidazo[1,2-*a*]pyridin-3-yl)-2-fluoro-2-phosphonopropanoic acid (3a): to compound **3b** dissolved in mixture of H_2O:EtOH v:v 1:1 (4 mL) sodium chloride (20 eq.) was added. After 3 days of mixing at rt, product **3a** was separated using an Amberlite IR120 ion exchange column (H_2O as eluent). Product **3a** was further purified by preparative HPLC (gradient 5→20 min. 0→40% B, retention time 10.4 min.) followed by lyophilization. Yield: 42% (65 mg). In the ^{13}C NMR spectrum of **3a** signals from $\underline{C}F_3\underline{C}O_2^-$ are present. **^{1}H NMR** (700 MHz, D_2O pH 2) δ 8.58 (s, $C\underline{H}_{Ar(5)}$, 1H), 7.90 (d, $^3J_{HH}$ = 9.3, $C\underline{H}_{Ar(7)}$, 1H), 7.85 (d, $^3J_{HH}$ = 9.7, $C\underline{H}_{Ar(8)}$, 1H), 7.85 (s, $C\underline{H}_{Ar(2)}$, 1H), 4.10 (s, $OCC\underline{H}_2Cl$, 1H), 4.06 (ddd, $^3J_{FH}$ = 38.2, $^2J_{HH}$ = 16.3, $^3J_{PH}$ = 4.3, $C\underline{H}_2C(F)P$, 1H), 3.67 (ddd, $^2J_{HH}$ = 16.6, $^3J_{FH}$ = 9.9, $^3J_{PH}$ = 6.7, $C\underline{H}_2C(F)P$, 1H), 3.35 (t, $^3J_{HH}$ = 6.7, $C\underline{H}_2NH$, 2H), 2.90 (t, $^3J_{HH}$ = 7.5, $C_{Ar(6)}C\underline{H}_2$, 1H), 2.02 (tt, $^3J_{HH}$ = 7.1 $C_{Ar(6)}CH_2C\underline{H}_2$, 2H). **^{13}C NMR** (176 MHz, D_2O pH 2) δ 172 ($\underline{C}O_2H$, 1C), 169.5 (s, $\underline{C}ONH$, 1C), 139.1 (s, $\underline{C}_{Ar(9)}$, 1C), 135.3 (s, $\underline{C}H_{Ar(7)}$, 1C), 131.3 (s, $\underline{C}_{Ar(6)}$, 1C), 124.4 (d, J_{FC} = 3.4, $\underline{C}H_{Ar(5)}$, 1C), 121.2 (s, $\underline{C}H_{Ar(2)}$, 1C), 121.1 (d, $^3J_{PC}$ = 12.9, $\underline{C}_{Ar(3)}$, 1C), 111.60 (s, $\underline{C}H_{Ar(8)}$, 1C), 97 (m, $\underline{C}(F)P$, 1C, in HMBC spectrum), 42.2 (s, $\underline{C}H_2Cl$, 1C), 39.0 (s, $\underline{C}H_2NH$, 1C), 29.1 (s, $\underline{C}H_2CH_2CH_2NH$, 1C), 28.8 (s, $\underline{C}H_2CH_2NH$, 1C), 27.5 (d, $^2J_{FC}$ = 19.9, $\underline{C}H_2C(F)P$, 1C). **^{31}P NMR** (284 MHz, D_2O pH 2) δ 6.88 (d, $^2J_{PF}$ = 68.1). HR-MS: m/z [M+H$^+$] calculated 422.0679, found 422.0673.

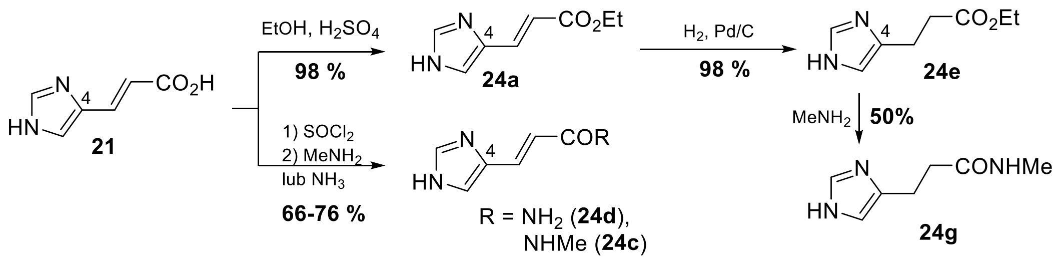

Scheme S10. Synthesis of compounds **24a,c,d,e,g.**

Ethyl (*E*)-3-(1*H*-imidazol-4-yl)acrylate (24a): urocanic acid **21** (400 mg) was dissolved in EtOH (25 mL). Then, concentrated H_2SO_4 (0.3 mL) was added. The reaction mixture was stirred for 5h at 75 °C and overnight at 60 °C. Obtained mixture was alkalized with concentrated $Na_2CO_{3(aq)}$ up to pH 8. The aqueous phase was extracted with DCM (4x40 mL). The combined organic phases were dried over $MgSO_4$ and concentrated. Yield: 98%. **^{1}H NMR** (250 MHz, D_2O pH 7) δ 8.72 (s, CH_{im}, 2H), 7.69 (s, CH_{im}, 2H), 7.51 (d, $^2J_{HH}$= 16.3, $C\underline{H}$=CH, 1H), 6.45 (d, $^2J_{HH}$= 16.3, $C\underline{H}$=CH, 1H), 4.19 (q, $^3J_{HH}$= 7.2, $OC\underline{H}_2CH_3$, 2H), 1.22 (t, $^3J_{HH}$= 7.0, OCH_2CH_3, 3H).

(*E*)-3-(1*H*-imidazol-4-yl)-*N*-methylacrylamide (24c): urocanic acid **21** (500 mg, 3.6 mmol) was suspended in $SOCl_2$ (12 mL). The suspension was refluxed for 3h. Then, exess $SOCl_2$ was evaporated providing anhydrous conditions. Thus obtained acyl chloride was added in 4 portions to the cooled (0 °C) solution of $MeNH_2$*HCl (4 eq., 14.5 mmol, 978 mg) and TEA (18.1 mmol, 2.5 mL) in DMF (5 mL). The reaction was continued for 2h at 0 °C. Next, the solvent was evaporated. Compound **24c** was

initially purified by flash chromatography using Gilson PLC 2250 purification system. Eluents A:$CHCl_3$ and B:MeOH. Product **24c** was further purified by crystallization from $CHCl_3$ (10 mL). Yield: 66%. **^{1}H NMR** (250 MHz, D_2O) δ 7.89 (s, C$\underline{H}_{Im(2)}$, 1H), 7.40 (s, C$\underline{H}_{Im(5)}$, 1H), 7.33 (d, $^3J_{HH}$ = 15.8, C$\underline{H}$=CH, 1H), 6.43 (d, $^3J_{HH}$ = 15.7, C$\underline{H}$=CH, 1H), 2.78 (s, NH$\underline{Me}$, 3H).

(*E*)-3-(1*H*-imidazol-4-yl)acrylamide (24d): obtained according to the procedure for compound **24c**, using 25% $NH_{3(aq)}$. Scale: 500 mg of **21**. Compound **24d** was initially purified by flash chromatography using Gilson PLC 2250 purification system. Eluents mixture of A:$CHCl_3$ and B:MeOH. Product **24d** was further purified by crystallization of impurities from DMF (3 mL). The filtrate containing product **24d** was concentrated under reduced pressure. Yield: 76%. **^{1}H NMR** (250 MHz, D_2O pH 8) δ 7.80 (s, C$\underline{H}_{Im(2)}$, 1H), 7.29 (s, C$\underline{H}_{Im(5)}$, 1H), 7.19 (d, $^3J_{HH}$ = 16.1, C$\underline{H}$=CH, 1H), 6.30 (d, $^3J_{HH}$ = 15.4, C$\underline{H}$=CH, 1H).

Ethyl 3-(1*H*-imidazol-4-yl)propanoate (24e): the reaction was carried out in a single-neck flask equipped with twoway stopcock, which enabled degassing the system (vacuum– hydrogen –vacuum, three times). In a single-neck flask compound **24a** (4.0 g) and 10% Pd/C (480 mg) was placed in EtOH (250 mL). The system was degassed using a two-way stopcock. This suspension was stirred for 24h at room temperature. The catalyst was then filtered off through a thin layer of Celite500, and the filtrate was evaporated to dryness. Yield: 98% (3.95 g). **^{1}H NMR** (250 MHz, $CDCl_3$) δ 1.24 (t, $^3J_{HH}$= 7.1, $OCH_2C\underline{H}_3$, 3H), 2.65 (t, $^3J_{HH}$= 7.0, $C_{Im}CH_2C\underline{H}_2$, 2H), 2.92 (t, $^3J_{HH}$= 7.0, $C_{Im}C\underline{H}_2CH_2$, 2H) 4.14 (q, $^3J_{HH}$= 7.1, $OC\underline{H}_2CH_3$, 2H), 6.80 (s, C$\underline{H}_{im(5)}$, 1H), 7.55 (s, C$\underline{H}_{im(2)}$, 1H).

3-(1*H*-imidazol-4-yl)-*N*-methylpropanamide (24g): to compound **24e** (250 mg) dissolved in H_2O (2 mL), NaOH (0,5 eq., 560 mg) was added. The mixture was cooled to 0 °C and then $MeNH_2$*HCl (2.9 g) was added. The reaction mixture was stirred for 7 days at room temperature. The solvent was evaporated and compound **24g** was purified by flash chromatography using Gilson PLC 2250 purification system. Eluents A:$CHCl_3$ and B:MeOH. Yield: 50% (115 mg). **^{1}H NMR** (700 MHz, D_2O pH 5) δ 7.74 (d, $^4J_{HH}$ = 1.3, C$\underline{H}_{Im(2)}$, 1H), 6.92 (d, $^4J_{HH}$ = 1.1, C$\underline{H}_{Im(5)}$, 1H), 2.92 (td, $^3J_{HH}$ = 7.3, 0.9, C$\underline{H}_2$, 2H), 2.70 (s, C$\underline{H}_3$, 3H), 2.58 (t, $^3J_{HH}$ = 7.3, C$\underline{H}_2$, 2H).

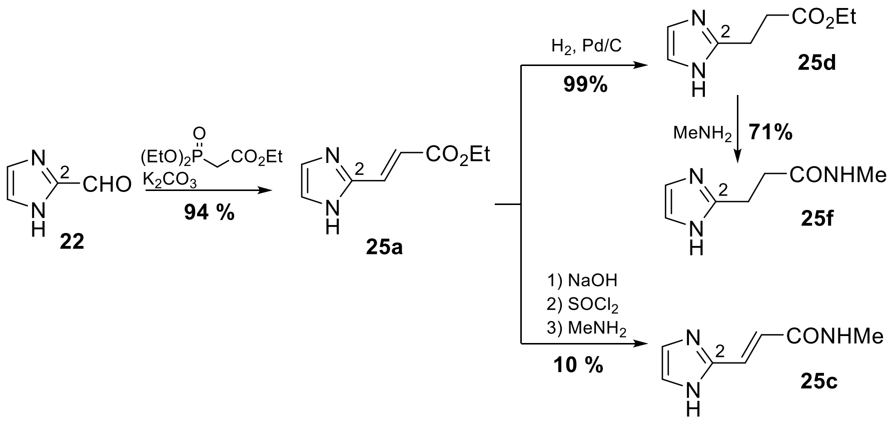

Scheme S11. Synthesis of compounds **25a,c,d,f.**

Ethyl (*E*)-3-(1*H*-imidazol-2-yl)acrylate (25a): In a double-neck flask equipped with thermometer and reflux condenser compound **22** (1.5 g, 1.0 eq.), triethyl phosphonoacetate (1.2 eq.) and anhydrous Na_2CO_3 were suspended in EtOH (60 mL). The reaction mixture was stirred at 70 °C for 1h. Then, the exess Na_2CO_3 was filtered and disposed. The filtrate was concentrated and redissolved in DCM (30 mL) and water (60 mL). The aqueous phase was extracted with DCM (3x30 mL). The combined organic phases were dried over $MgSO_4$ and concentrated. Yield: 71%. **^{1}H NMR** (250 MHz, CD_3OD) δ

1.31 (t, $^3J_{HH}$= 7.1, OCH_2CH_3, 3H), 4.24 (q, $^3J_{HH}$= 7.1, $OC\underline{H}_2CH_3$, 2H), 6.56 (d, $^2J_{HH}$= 16.0, $CH=C\underline{H}CO_2$, 1H), 7.19 (s, CH_{Im}, 2H), 7.47 (d, $^2J_{HH}$= 16.0, $C\underline{H}=CHCO_2$, 1H).

(*E*)-3-(1*H*-imidazol-2-yl)-*N*-methylacrylamide (25c): obtained in three step synthesis. The first step involved hydrolysis of ethyl ester **25a**. To the compound **25a** (800 mg) 2.5 M NaOH in EtOH (10 mL) was added. The reaction was continued for 24h at room temperature. Obtained precipitate was filtered and used in the next step. This involved aminolysis according to the procedure for compound **25c**. Compound **20c** was initially purified by flash chromatography using Gilson PLC 2250 purification system. Eluents A:$CHCl_3$ and B:MeOH. Product **25c** was further purified by crystallization from $CHCl_3$ (10 mL). Yield: 10% (65 mg). **^{1}H NMR** (700 MHz, MeOD) δ 7.31 (d, $^3J_{HH}$ = 15.7, $C\underline{H}=CH$, 1H), 7.13 (s, $C\underline{H}_{im}C\underline{H}_{im}$, 2H), 6.64 (d, $^3J_{HH}$ = 15.7, $C\underline{H}=CH$, 1H), 2.81 (s, NH$\underline{Me}$, 3H).

Ethyl 3-(1*H*-imidazol-2-yl)propanoate (25d): obtained according to the procedure for compound **24e**. Scale: 450 mg (2.7 mmol) of **25a**. Yield: 99%. **^{1}H NMR** (250 MHz, $CDCl_3$) δ 1.26 (t, $^3J_{HH}$ = 7.1, $OCH_2C\underline{H}_3$, 3H), 2.74 (t, $^3J_{HH}$= 7.1, $C_{im}CH_2C\underline{H}_2$, 2H), 3.04 (t, $^3J_{HH}$= 7.1, $C_{im}C\underline{H}_2CH_2$, 2H) 4.16 (q, $^3J_{HH}$= 7.1, $OC\underline{H}_2CH_3$, 2H), 6.94 (s, $C\underline{H}_{im}$, 2H)

3-(1*H*-imidazol-2-yl)-*N*-methylpropanamide (25f): obtained according to the procedure for compound **24g**. Scale: 160 mg (0.96 mmol) of **25d**. Compound **25f** was purified by flash chromatography using Gilson PLC 2250 purification system. Eluents A:$CHCl_3$ and B:MeOH. Yield: 71% (104 mg). **^{1}H NMR** (700 MHz, D_2O pH 5) δ 7.03 (s, $C\underline{H}_{im}C\underline{H}_{im}$, 2H), 3.03 (t, $^3J_{HH}$ = 7.5, $C\underline{H}_2$, 2H), 2.72 (s, NH$\underline{Me}$, 3H), 2.66 (t, $^3J_{HH}$ = 7.5, $C\underline{H}_2$, 2H).

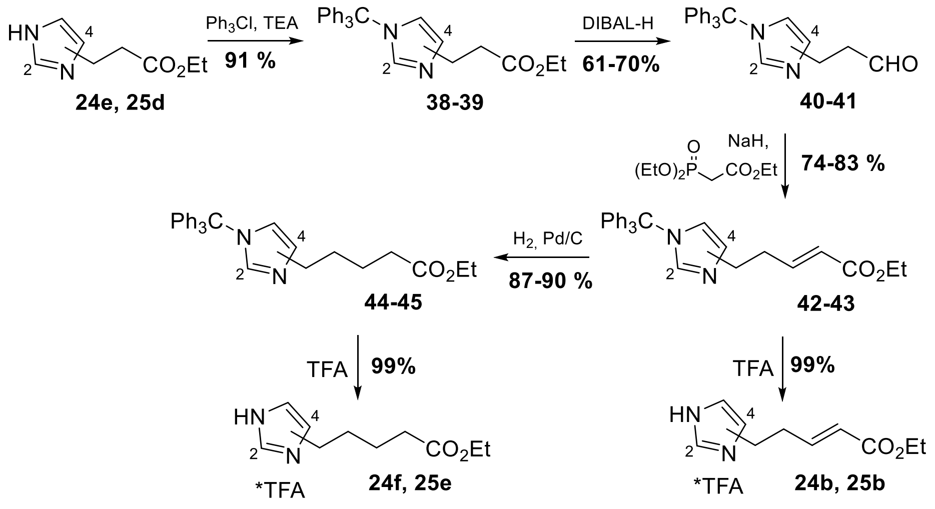

Scheme S12. Synthesis of compounds **24,b,f and 25b,f.**

Ethyl 3-(1-trityl-1*H*-imidazol-4-yl)propanoate (38): in a dry single-neck flask compound **24e** (3.5 g, 21.0 mmol, 1.0 eq.) was dissolved in $CHCl_3$ (40 mL) and cooled to -5 °C. Then, TEA (3.5 mL, 25.2 mmol, 1.2 eq.) and trityl chloride (7.0 g, 1.2 eq.) were added via syringe. The mixture was stirred overnight at room temperature. The reaction was quenched by addition of water (30 mL). The aqueous phase was extracted with DCM (3x40 mL). The combined organic phases were dried over $MgSO_4$ and concentrated. The solvent was evaporated and compound **38** was purified by flash chromatography using Gilson PLC 2250 purification system. Eluents A:$CHCl_3$ and B:MeOH (gradient 0%→1%B). Yield: 91%. **^{1}H NMR** (250 MHz, $CDCl_3$) δ 1.19 (t, $^3J_{HH}$ = 7.1, $OCH_2C\underline{H}_3$, 3H), 2.63 (t, $^3J_{HH}$ = 7.5, $C_{im}CH_2C\underline{H}_2$, 2H), 2.87 (t, $^3J_{HH}$ = 7.5, $C_{im}C\underline{H}_2$, 2H), 4.07 (q, $^3J_{HH}$= 7.1, $OC\underline{H}_2CH_3$, 2H), 6.54 (d, $^3J_{HH}$= 0.7, $C\underline{H}_{im}$, 1H), 7.08-7.16 (m, $C\underline{H}_{Ph}$, 6H), 7.29-7.37 (m, $C\underline{H}_{im}$, $C\underline{H}_{Ph}$, 10H).

Ethyl 3-(1-trityl-1*H*-imidazol-2-yl)propanoate (39): obtained according to the procedure for compound **38**. Scale: 100 mg of **25d**. Yield: 91%. **^{1}H NMR** (250 MHz, $CDCl_3$) δ 1.16 (t, $^3J_{HH}$ = 7.1, $OCH_2C\underline{H}_3$, 3H), 2.12-2.30 (m, $C\underline{H}_2C\underline{H}_2$, 4H), 4.01 (q, $^3J_{HH}$ = 7.1, $OC\underline{H}_2CH_3$, 2H), 6.73 (d, $^3J_{HH}$= 1.5, $C\underline{H}_{im(4)}$, 1H), 6.94 (d, $^3J_{HH}$= 1.5, $C\underline{H}_{im(5)}$, 1H), 7.12-7.16 (m, $C\underline{H}_{Ph}$, 6H), 7.30-7.36 (m, $C\underline{H}_{Ph}$, 9H).

3-(1-trityl-1*H*-imidazol-4-yl)propanal (40): in a dry single-neck flask compound **38** (630 mg, 1.0 eq) was dissolved in anhydrous DCM (10 mL) and cooled to -75°C. Then, 1.2M DIBAL-H in toluene (2.56 mL, 2.0 eq.) was added dropwise for 15 minutes. The reaction mixture was stirred for 1h at -75°C. The reaction was quenched by addition of water (10 mL) and after 30 min. of stirring at room temperature, obtained precipitate was removed by filtration. The filtrate was extracted with $CHCl_3$ (4x15 mL). The combined organic phases were dried over $MgSO_4$ and concentrated. The solvent was evaporated and compound **40** was purified by flash chromatography using Gilson PLC 2250 purification system. Eluents A:$CHCl_3$ and B:MeOH (*v:v* 30:1). Yield: 70% (395 mg). **^{1}H NMR** (250 MHz, $CDCl_3$) δ: 2.78 (t, $^3J_{HH}$ = 7.4, $C\underline{H}_2CHO$, 2H), 2.88 (t, $^3J_{HH}$ = 7.4, $C\underline{H}_2CH_2CHO$, 2H), 6.56 (s, $C\underline{H}_{im(5)}$, 1H), 7.11-7.13 (m, $C\underline{H}_{Ph}$, 6H), 7.32-7.34 (m, $C\underline{H}_{Ph}$, 9H), 7.35 (s, $C\underline{H}_{im(2)}$, 1H), 9.81 (t, $^3J_{HH}$ = 1.5, $C\underline{H}O$, 1H).

3-(1-trityl-1*H*-imidazol-2-yl)propanal (41): obtained according to the procedure for compound **40**. Scale: 1.1 mmol of **39**. Compound **41** was purified by flash chromatography using an eluent mixture of DCM:AcOEt:MeOH. Yield: 74% (600 mg). **^{1}H NMR** (250 MHz, $CDCl_3$) δ 2.19 (t, $^3J_{HH}$ = 7.2, $C\underline{H}_2CHO$, 2H), 2.37 (t, $^3J_{HH}$= 7.2, $C\underline{H}_2CH_2CHO$, 2H), 6.74 (d, $^3J_{HH}$ = 1.4, $C\underline{H}_{im(4)}$, 1H), 6.93 (d, $^3J_{HH}$ = 1.4, $C\underline{H}_{im(5)}$, 1H), 7.13-7.15 (m, $C\underline{H}_{Ph}$, 6H), 7.31-7.34 (m, $C\underline{H}_{Ph}$, 9H), 9.54 (s, $C\underline{H}O$, 1H).

Ethyl (*E*)-5-(1-trityl-1*H*-imidazol-4-yl)pent-2-enoate (42): obtained according to the Horner–Wadsworth–Emmons reaction procedure for compound **12b** (scheme S2). Scale: 150 mg of **40**. Compound **42** was purified by flash chromatography using an eluent mixture of EtOAc:toluene (v:v 1:1). Yield: 83%. **^{1}H NMR** (250 MHz, CD_3OD) δ 1.28 (t, $^3J_{HH}$= 7.1, $OCH_2C\underline{H}_3$, 3H), 2.50-2.58 (m, $C\underline{H}_2CH{=}CH$, 2H), 2.70 (t, $^3J_{HH}$ = 7.1, $C\underline{H}_2CH_2CH{=}CH$, 2H), 4.18 (q, $^3J_{HH}$ = 7.1, $OC\underline{H}_2CH_3$, 2H), 5.79 (d, $^3J_{HH}$ = 15.7, $CH{=}C\underline{H}CO_2$, 1H), 6.53 (s, $C\underline{H}_{Im(5)}$, 1H), 6.95 (dt, $^3J_{HH}$ = 15.6, $^3J_{HH}$ = 6.9, $C\underline{H}{=}CHCO_2$, 1H), 7.10-7.15 (m, $C\underline{H}_{Ph}$, 6H), 7.30-7.33 (m, $C\underline{H}_{Ph}$, 9H), 7.34 (s, $C\underline{H}_{Im(2)}$, 1H).

Ethyl (*E*)-5-(1-trityl-1*H*-imidazol-2-yl)pent-2-enoate (43): obtained according to the Horner–Wadsworth–Emmons reaction procedure for compound **12b** (scheme S2). Scale: 150 mg of **41**. Compound **43** was purified by flash chromatography using an eluent mixture of EtOAc:toluene (v:v 1:1). Yield: 66%. **^{1}H NMR** (250 MHz, $CDCl_3$) δ 1.25 (t, $^3J_{HH}$ = 7.2, $OCH_2C\underline{H}_3$, 3H), 2.07-2.15 (m, $C\underline{H}_2C\underline{H}_2$, 4H), 4.12 (q, $^3J_{HH}$ = 7.2, $OC\underline{H}_2CH_3$, 2H), 5.49 (bd, $^3J_{HH}$ = 15.6, $CH{=}C\underline{H}CO_2$, 1H), 6.60 (dt, $^3J_{HH}$ = 16.0, $^3J_{HH}$ = 6.1, $C\underline{H}{=}CHCO_2$, 1H), 6.71 (d, $^3J_{HH}$ = 1.5, $C\underline{H}_{Im(4)}$, 1H), 6.96 (d, $^3J_{HH}$ = 1.5, $C\underline{H}_{Im(5)}$, 1H), 7.10-7.15 (m, $C\underline{H}_{Ph}$, 6H), 7.30-7.35 (m, $C\underline{H}_{Ph}$, 9H).

Ethyl 5-(1-trityl-1*H*-imidazol-4-yl)pentanoate (44): obtained according to the procedure for compound **24e**. Scale: 100 mg of **42**. Yield: 90%. **^{1}H NMR** (250 MHz, $CDCl_3$) δ 1.23 (t, $^3J_{HH}$ = 7.1, $OCH_2C\underline{H}_3$, 3H), 1.60-1.70 (m, $C\underline{H}_2C\underline{H}_2$, 4H), 2.30 (t, $^3J_{HH}$= 7.1, $C\underline{H}_2CO_2$, 2H), 2.59 (t, $^3J_{HH}$ = 7.1, $C_{im}C\underline{H}_2$, 2H), 4.10 (q, $^3J_{HH}$ = 7.1, $OC\underline{H}_2CH_3$, 2H), 6.55 (s, $C\underline{H}_{im(5)}$, 1H), 7.09-7.15 (m, $C\underline{H}_{Ph}$, 6H), 7.29-7.38 (m, $C\underline{H}_{Ph}$, 9H), 7.47 (s, $C\underline{H}_{im(2)}$, 1H).

Ethyl 5-(1-trityl-1*H*-imidazol-2-yl)pentanoate (45): obtained according to the procedure for compound **24e**. Scale: 400 mg of **43**. Yield: 87%. **^{1}H NMR** (250 MHz, $CDCl_3$) δ 1.23 (t, $^3J_{HH}$ = 7.0, $OCH_2C\underline{H}_3$, 3H), 1.97-2.02 (m, $C\underline{H}_2C\underline{H}_2$, 4H), 2.38 (t, $^3J_{HH}$ = 7.0, $C\underline{H}_2CO_2$, 2H), 3.08 (t, $^3J_{HH}$ = 7.0, $C_{im}C\underline{H}_2$, 2H), 4.08 (q, $^3J_{HH}$ = 7.0, $OC\underline{H}_2CH_3$, 2H), 6.73 (s, $C\underline{H}_{im(4)}$, 1H), 7.01 (s, $C\underline{H}_{im(5)}$, 1H), 7.09-7.15 (m, $C\underline{H}_{Ph}$, 6H), 7.30-7.36 (m, $C\underline{H}_{Ph}$, 9H).

Ethyl (*E*)-5-(1*H*-imidazol-4-yl)pent-2-enoate (24b): to compound **42** (315 mg, 0.72 mmol) dissolved in DCM (6 mL) neat trifluoroacetic acid (2 mL, final concentration 25%) was added. After 4h of stirring at RT, solvents were evaporated under reduced pressure. Next. $CHCl_3$ (2 mL) and H_2O (2 mL) was added. The aqueos phase was seperated and the organic phase was additionally extracted with water (2x2 mL). Combined aqueos phases were concentrated under reduced pressure. Product **24b** was obtained as a trifluoroacetic salt with quantitive yield (240 mg). **^{1}H NMR** (250 MHz, $CDCl_3$) δ 8.49 (s, C$\underline{H}_{im(2)}$, 1H), 7.04 (s, C$\underline{H}_{im(5)}$, 1H), 6.89 (dt, $^3J_{HH}$ = 15.7, 6.8, C$\underline{H}$=CHCO$_2$, 1H), 5.85 (d, $^3J_{HH}$ = 15.7, CH=C$\underline{H}$CO$_2$, 1H), 4.17 (q, $^3J_{HH}$ = 7.1, OC$\underline{H}_2$CH$_3$, 2H), 2.91 (t, $^3J_{HH}$ = 7.4, C$\underline{H}_2$CH$_2$CH=CH, 2H), 2.66 – 2.52 (m, C$\underline{H}_2$CH=CH, 2H), 1.27 (t, $^3J_{HH}$= 7.1, OCH$_2$C$\underline{H}_3$, 3H).

Ethyl 5-(1*H*-imidazol-4-yl)pentanoate (24f): obtained according to the procedure for compound **24b**. Scale: 315 mg of **44**. Yield: quantitive.

Ethyl (*E*)-5-(1*H*-imidazol-4-yl)pent-2-enoate (25b): obtained according to the procedure for compound **24b**. Scale: 336 mg of **43**. Yield: quantitive. **^{1}H NMR** (250 MHz, $CDCl_3$) δ 7.19 (s, C$\underline{H}_{Im(4)}$C$\underline{H}_{Im(5)}$, 2H), 6.84 (dt, $^3J_{HH}$ = 15.5, $^3J_{HH}$ = 6.8, C$\underline{H}$=CHCO$_2$, 1H), 5.83 (d, $^3J_{HH}$ = 15.8, CH=C$\underline{H}$CO$_2$, 1H), 4.14 (q, $^3J_{HH}$ = 7.1, OC$\underline{H}_2$CH$_3$, 2H), 3.19 (t, $^3J_{HH}$ = 7.5, C$_{im}$C$\underline{H}_2$, 2H), 2.70 (dt, $^3J_{HH}$ = 7.3, CH$_2$C$\underline{H}_2$CH, 2H), 1.25 (t, $^3J_{HH}$ = 7.1, OCH$_2$C$\underline{H}_3$, 3H).

Ethyl 5-(1*H*-imidazol-4-yl)pentanoate (25e): obtained according to the procedure for compound **24b**. Scale: 214 mg of **45**. Yield: quantitive. **^{1}H NMR** (250 MHz, $CDCl_3$) δ 7.23 (s, C$\underline{H}_{im(4)}$C$\underline{H}_{im(5)}$, 1H), 4.13 (q, $^3J_{HH}$ = 7.1, OC$\underline{H}_2$CH$_3$, 2H), 2.93 (t, $^3J_{HH}$ = 7.3, C$_{im}$C$\underline{H}_2$, 2H), 2.39 (t, $^3J_{HH}$ = 7.2, C$\underline{H}_2$CO$_2$, 2H), 1.86 – 1.51 (m, C$\underline{H}_2$C$\underline{H}_2$, 4H), 1.22 (t, $^3J_{HH}$ = 7.2, OCH$_2$C$\underline{H}_3$, 3H).

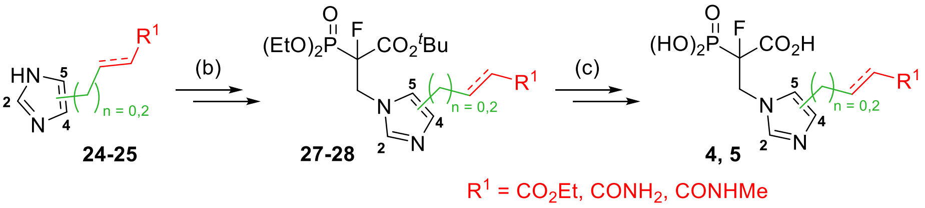

Scheme S13. Synthesis of compounds **4-5.**

Ethyl (*E*)-3-(1-(3-(*tert*-butoxy)-2-(diethoxyphosphoryl)-2-fluoro-3-oxopropyl)-1*H*-imidazol-4-yl)acrylate (27a): obtained according to the *general procedure of Michael addition and fluorination.* Scale: 127 mg (0.65 mmol) of **24a**. Compound **27a** was purified by flash chromatography using Gilson PLC 2250 purification system. Eluents A:$CHCl_3$, B:Acetone, to both of them TEA was added, 1mL/(1L of eluent) (gradient 5→30 min. 0→10% B, retention time 16 min.). Yield: 62% (180 mg). According to ^{1}H NMR spectrum of the main fraction, single regioisomer C4 was obtained with no traces of regioisomer C5. **^{1}H NMR** (700 MHz, $CDCl_3$) δ 7.53 (s, C$\underline{H}_{Im(2)}$, 1H), 7.52 (d, $^3J_{HH}$ = 15.4, C$_{im}$C$\underline{H}$=, 1H), 7.18 (s, C$\underline{H}_{Im(5)}$, 1H), 6.53 (d, $^3J_{HH}$ = 15.7, C$_{im}$CH=C$\underline{H}$, 1H), 4.69 (ddd, $^3J_{FH}$ = 31.3, $^2J_{HH}$ = 15.2, $^3J_{PH}$ = 5.8, C$\underline{H}_2$C(F)P, 1H), 4.51 (ddd, $^2J_{HH}$ = 15.1, $^3J_{FH}$ = 15.1, $^3J_{PH}$ = 3.9, C$\underline{H}_2$C(F)P, 1H), 4.31 – 4.20 (m, C$\underline{H}_2$OP, 4H), 4.23 (q, $^3J_{HH}$ = 7.1, CO$_2$C$\underline{H}_2$, 2H), 1.44 (s, C(C$\underline{H}_3$)$_3$, 9H), 1.39 i 1.40 (2t, $^3J_{HH}$ = 7.1, C$\underline{H}_3$CH$_2$OP 6H), 1.31 (t, $^3J_{HH}$ = 7.1, CO$_2$CH$_2$C$\underline{H}_3$, 3H). **^{13}C NMR** (176 MHz, $CDCl_3$) δ 167.2 (s, $\underline{C}$O$_2$Et, 1C), 163.3 (dd, $^2J_{FC}$ = 22.0, $^2J_{PC}$ = 2.0, $\underline{C}$O$_2$*t*Bu, 1C), 139.3 (s, $\underline{C}$H$_{Im(2)}$, 1C), 138.1 (s, $\underline{C}_{Im(4)}$, 1C), 135.4 (s, C$_{Im(4)}$$\underline{C}$H=, 1C), 122.2 (s, $\underline{C}$H$_{Im(5)}$, 1C), 116.4 (s, C$_{im}$CH=$\underline{C}$H, 1C), 93.9 (dd, $^1J_{FC}$ = 203.8, $^1J_{PC}$ = 158.0, $\underline{C}$(F)P, 1C), 85.2 (s, CO$_2$$\underline{C}Me_3$, 1C), 64.7 (d, $^2J_{PC}$ = 6.5, $\underline{C}$H$_2$OP, 1C), 64.5 (d, $^2J_{PC}$ = 6.9, $\underline{C}$H$_2$OP, 1C), 59.9 (s, CO$_2$$\underline{C}H_2$, 1C), 49.2 (dd, $^2J_{FC}$ = 20.0, $^2J_{PC}$ = 5.9, $\underline{C}$H$_2$C(F)P, 1C), 27.4 (s, C($\underline{C}$H$_3$)$_3$,

3C), 16.1 (d, $^3J_{PC}$ = 5.7, $\underline{C}H_3CH_2OP$, 2C), 14.1 (s, $CO_2CH_2\underline{C}H_3$, 1C). **^{31}P NMR** (101 MHz, $CDCl_3$) δ 10.06 (d, $^2J_{PF}$ = 78.8).

Ethyl (*E*)-5-(1-(3-(*tert*-butoxy)-2-(diethoxyphosphoryl)-2-fluoro-3-oxopropyl)-1*H*-imidazol-4/5-yl)pent-2-enoate (27b): obtained according to the *general procedure of Michael addition and fluorination*. Scale: 0.72 mmol of **24b**. In fluorination step 1.4 eq. of NaH was used. Compound **27b** was purified by flash chromatography using Gilson PLC 2250 purification system. Eluents A:$CHCl_3$, B:AcOEt (gradient 10→25 min. 0→5% B, retention time 26 min.). Yield: 54% (187 mg, purity 66% acourding to ^{31}P NMR spectrum). According to ^{1}H NMR spectrum of the main fraction, the mixture of regioisomer C4 and C5 was obtained in ratio 77:23. Spectroscopic data of regioisomer C4: **^{1}H NMR** (250 MHz, $CDCl_3$) δ 7.39 (d, $^4J_{HH}$= 1.2, $C\underline{H}_{Im(2)}$, 1H), 6.98 (dt, $^3J_{HH}$ = 15.7, 6.8, $C\underline{H}$=$CHCO_2$, 1H), 6.69 (d, $^4J_{HH}$= 1.5, $C\underline{H}_{Im(5)}$, 1H), 5.84 (dt, $^3J_{HH}$ = 15.6, $^4J_{HH}$ = 1.6, CH=$C\underline{H}CO_2$, 1H), 4.64 (ddd, $^3J_{FH}$ = 32.6, $^2J_{HH}$ = 15.2, $^3J_{PH}$ = 5.3, $C\underline{H}_2C(F)P$, 1H), 4.45 (ddd, $^2J_{HH}$ = 15.0, $^3J_{FH}$ = 15.0, $^3J_{PH}$ = 3.8, $C\underline{H}_2C(F)P$, 1H), 4.29 – 4.19 (m, $C\underline{H}_2OP$, 4H), 4.16 (q, $^3J_{HH}$ = 7.1, $CO_2C\underline{H}_2$, 2H), 2.68 (t, $^3J_{HH}$ = 7.7, $C_{im}C\underline{H}_2CH_2$, 2H), 2.57 – 2.49 (m, C_{im} $CH_2C\underline{H}_2$, 2H), 1.41 (s, $C(C\underline{H}_3)_3$, 9H), 1.37 (t, $^3J_{HH}$ = 7.1, $C\underline{H}_3CH_2OP$, 6H), 1.27 (t, $^3J_{HH}$ = 7.1, $CO_2CH_2C\underline{H}_3$, 3H). **^{13}C NMR** (176 MHz, $CDCl_3$) δ 166.8 (s, $\underline{C}O_2Et$, 1C), 163.9 (d, $^2J_{FC}$ = 22.2, $^2J_{PC}$ =2.0, $\underline{C}O_2t$Bu, 1C), 148.5 (s, $C\underline{H}$=$CHCO_2$, 1C), 141.7 (s, $\underline{C}_{\underline{im(4)}}$, 1C), 137.9 (s, $\underline{C}H_{Im(2)}$, 1C), 121.8 (s, CH=$C\underline{H}CO_2$, 1C), 116.6 (s, $\underline{C}H_{Im(5)}$, 1C), 94.6 (dd, $^1J_{FC}$ = 203.2, $^1J_{PC}$ = 157.8, $\underline{C}(F)P$, 1C), 85.3 (s, $CO_2\underline{C}Me_3$, 1C), 65.0 (d, $^2J_{PC}$ = 6.6, $\underline{C}H_2OP$, 1C), 64.8 (d, $^2J_{PC}$ = 7.0, $\underline{C}H_2OP$, 1C), 60.3 (s, $CO_2\underline{C}H_2$, 1C), 49.4 (dd, $^2J_{FC}$ = 20.1, $^2J_{PC}$ = 6.0, $\underline{C}H_2C(F)P$, 1C), 32.0 (s, $C_{im}CH_2\underline{C}H_2$, 1C), 27.9 (s, $C(\underline{C}H_3)_3$, 3C), 26.9 (s, $C_{im}\underline{C}H_2CH_2$, 1C), 16.5 (d, $^3J_{PC}$ = 5.8, $\underline{C}H_3CH_2OP$, 2C), 14.4 (s, $CO_2CH_2\underline{C}H_3$, 1C). **^{31}P NMR** (283 MHz, $CDCl_3$) δ 10.13 (d, $^2J_{PF}$ = 79.9).

***Tert*-butyl (*E*)-2-(diethoxyphosphoryl)-2-fluoro-3-(4-(3-(methylamino)-3-oxoprop-1-en-1-yl)-1*H*-imidazol-1-yl)propanoate (27c)**: obtained according to the *general procedure of Michael addition and fluorination*. Scale: 153 mg of **24c**. In Michael addition DMF was used as a solvent. In fluorination step 1.7 eq. of NaH was used. Compound **27c** was purified by flash chromatography using Gilson PLC 2250 purification system. Eluents A:$CHCl_3$, B:AcOEt, to both of them TEA was added, 1mL/(1L of eluent) (gradient 14→30 min. 0→20% B, retention time 27 min.). Yield: 92% (404 mg, purity 66% acourding to ^{31}P NMR spectrum). According to ^{1}H NMR spectrum of the main fraction, single regioisomer C4 was obtained with no traces of regioisomer C5. **^{1}H NMR** (250 MHz, $CDCl_3$) δ 7.44 (s, $C\underline{H}_{Im(2)}$, 1H), 7.42 (d, $^3J_{HH}$ = 15.1, $C_{im(3)}C\underline{H}$=, 1H), 7.04 (s, $C\underline{H}_{Im(5)}$, 1H), 6.54 (d, $^3J_{HH}$ = 15.1, $C_{im}CH$=$C\underline{H}$, 1H), 5.884 i 5.877 (2bs, $N\underline{H}$, 1H), 4.65 (ddd, $^3J_{FH}$ = 31.9, $^2J_{HH}$ = 15.3, $^3J_{PH}$ = 5.8, $C\underline{H}_2C(F)P$, 1H), 4.48 (ddd, $^2J_{HH}$ = 15.0, $^3J_{FH}$ = 15.0, $^3J_{PH}$ = 3.8, $C\underline{H}_2C(F)P$, 1H), 4.28 – 4.10 (m, $C\underline{H}_2OP$, 4H), 2.87 i 2.86 (2s, $NHC\underline{H}_3$, 3H), 1.38 (s, $CO_2C(CH_3)_3$, 9H), 1.34 (t, $^3J_{HH}$ = 7.1, $C\underline{H}_3CH_2OP$, 3H), 1.33 (t, $^3J_{HH}$ = 7.1, $C\underline{H}_3CH_2OP$, 3H); **^{13}C NMR** (176 MHz, $CDCl_3$) δ 167.0 (s, $\underline{C}$ONHMe, 1C), 164.8 (dd, $^2J_{FC}$ = 21.6, $^2J_{PC}$ = 1.6, $\underline{C}O_2{}^t$Bu, 1C), 139.2 (s, $\underline{C}H_{Im(5)}$, 1C), 138.6 (s, $\underline{C}_{\underline{im(4)}}$, 1C), 131.5 (s, $C_{im(4)}\underline{C}H$=, 1C), 121.8 (s, $\underline{C}H_{Im(2)}$, 1C), 119.1 (s, $C_{im(4)}CH$=$\underline{C}H$, 1C), 94.1 (dd, $^1J_{FC}$ = 204.1, $^1J_{PC}$ = 158.1, $\underline{C}(F)P$, 1C), 85.4 (s, $CO_2\underline{C}Me_3$, 1C), 64.9 (d, $^2J_{PC}$ = 6.8, $\underline{C}H_2OP$, 1C), 64.7 (d, $^2J_{PC}$ = 6.9, $\underline{C}H_2OP$, 1C), 49.3 (dd, $^2J_{FC}$ = 20.1, $^2J_{PC}$ = 5.8, $\underline{C}H_2C(F)P$, 1C), 27.6 (s, $C(\underline{C}H_3)_3$, 3C), 26.33 and 26.31 (2s, $NH\underline{C}H_3$, 1C), 16.3 (d, $^3J_{PC}$ = 5.7, $\underline{C}H_3CH_2OP$, 2C). **^{31}P NMR** (283 MHz, $CDCl_3$) δ 10.42 (d, $^2J_{PF}$ = 79.6).

***Tert*-butyl (*E*)-3-(4-(3-amino-3-oxoprop-1-en-1-yl)-1*H*-imidazol-1-yl)-2-(diethoxyphosphoryl)-2-fluoropropanoate (27d)**: obtained according to the *general procedure of Michael addition and fluorination*. Scale: 150 mg (1.1 mmol) of **24d**. In Michael addition DMF was used as a solvent. In fluorination step 1.7 eq. of NaH was used. Compound **27d** was purified by flash chromatography using Gilson PLC 2250 purification system. Eluents A:$CHCl_3$, B:Acetone and C: MeOH, to all of them TEA was added, 1mL/(1L of eluent) (gradient 10→50 min. 0→40%B, 35→50 min. 2→2%C,

retention time 45 min.). Yield: 57% (220 mg). According to ^{1}H NMR spectrum of the main fraction, single regioisomer C4 was obtained with no traces of regioisomer C5. **^{1}H NMR** (700 MHz, $CDCl_3$) δ 7.47 (d, $^{4}J_{HH}$ = 0.9, $C\underline{H}_{im}$, 1H), 7.45 (d, $^{3}J_{HH}$ = 15.2, $C\underline{H}$=CH, 1H), 7.08 (d, $^{4}J_{HH}$ = 1.3, $C\underline{H}_{im}$, 1H), 6.62 (d, $^{3}J_{HH}$ = 15.2, $C\underline{H}$=CH, 1H), 4.67 (ddd, $^{3}J_{FH}$ = 31.9, $^{2}J_{HH}$ = 15.3, $^{3}J_{PH}$ = 5.8, $C\underline{H}_2C(F)P$, 1H), 4.50 (ddd, $^{2}J_{HH}$ = 15.0, $^{3}J_{FH}$ = 15.0, $^{3}J_{PH}$ = 3.8, $C\underline{H}_2C(F)P$, 1H), 4.28 – 4.18 (m, $C\underline{H}_2OP$, 4H), 1.39 (s, $CO_2C(CH_3)_3$, 9H), 1.35 i 1.34 (2t, $^{3}J_{HH}$ = 7.1, $C\underline{H}_3CH_2OP$, 6H); **^{13}C NMR** (176 MHz, $CDCl_3$) δ 168.3 (s, $\underline{C}ONH_2$, 1C), 163.5 (dd, $^{2}J_{FC}$ = 21.7, $^{2}J_{PC}$ = 1.7, $CF\underline{C}O_2Et$, 1C), 139.4 (s, $\underline{C}H_{im}$, 1C) , 138.4 (s, $\underline{C}_{im(4)}$, 1C), 133.1 (s, $C_{im(4)}\underline{C}H$=CH, 1C), 122.3 (s, $\underline{C}H_{im}$, 1C), 118.2 (s, $C_{im(4)}CH=\underline{C}H$, 1C), 94.1 (dd, $^{1}J_{FC}$ = 204.0, $^{1}J_{PC}$ = 157.8, $\underline{C}(F)P$, 1C), 85.4 (s, $CO_2\underline{C}Me_3$, 1C), 64.94 (d, $^{2}J_{PC}$ = 6.6, $\underline{C}H_2OP$, 1C), 64.7 (d, $^{2}J_{PC}$ = 7.0, $\underline{C}H_2OP$, 1C), 49.4 (dd, $^{2}J_{FC}$ = 19.8, $^{2}J_{PC}$ = 6.2, $\underline{C}H_2C(F)P$, 1C), 27.7 (s, $C(\underline{C}H_3)_3$, 3C), 16.3 (d, $^{3}J_{PC}$ = 5.5, $\underline{C}H_3CH_2OP$, 2C). **^{31}P NMR** (283 MHz, $CDCl_3$) δ 10.38 (d, $^{2}J_{PF}$ = 79.5).

***Tert*-butyl 2-(diethoxyphosphoryl)-3-(4/5-(3-ethoxy-3-oxopropyl)-1*H*-imidazol-1-yl)-2-fluoropropanoate (27e):** obtained according to the *general procedure of Michael addition and fluorination.* Scale: 1.21 mmol (204 mg) of **24e**. Compound **27e** was purified by flash chromatography using Gilson PLC 2250 purification system. Eluents A:$CHCl_3$, B:Acetone (gradient 10→15 min. 0→5% B, retention time 17 min.). Yield: 86% (435 mg). According to ^{1}H NMR spectrum of the main fraction, the mixture of regioisomer C4 and C5 was obtained in ratio 90:10. Spectroscopic data of regioisomer C4: **^{1}H NMR** (250 MHz, $CDCl_3$) δ 7.38 (s, $C\underline{H}_{Im(2)}$, 1H), 6.71 (s, $C\underline{H}_{Im(5)}$, 1H), 4.63 (ddd, $^{3}J_{FH}$ = 32.6, $^{2}J_{HH}$ = 15.2, $^{3}J_{PH}$ = 5.3, $C\underline{H}_2C(F)P$, 1H), 4.44 (ddd, $^{2}J_{HH}$ = 15.0, $^{3}J_{FH}$ = 15.0, $^{3}J_{PH}$ = 3.8, $C\underline{H}_2C(F)P$, 1H), 4.33 – 4.14 (m, $C\underline{H}_2OP$, 4H), 4.12 (q, $^{3}J_{HH}$ = 7.1, $CO_2C\underline{H}_2$, 2H), 2.86 (t, $^{3}J_{HH}$ = 7.6, $C\underline{H}_2CH_2CO_2$, 2H), 2.63 (t, $^{3}J_{HH}$ = 7.9, $C\underline{H}_2CO_2$, 2H), 1.41 (s, $C(C\underline{H}_3)_3$, 9H), 1.37 (t, $^{3}J_{HH}$ = 7.1, $C\underline{H}_3CH_2OP$, 6H), 1.24 (t, $^{3}J_{HH}$ = 7.1, $CO_2CH_2C\underline{H}_3$, 3H). **^{13}C NMR** (176 MHz, $CDCl_3$) δ 173.1 (s, $\underline{C}O_2Et$, 1C), 163.7 (d, $^{2}J_{FC}$ = 22.1, $\underline{C}O_2tBu$, 1C), 141.3 (s, $\underline{C}_{im(4)}$, 1C), 137.7 (s, $\underline{C}H_{Im(2)}$, 1C), 116.5 (s, $\underline{C}H_{Im(5)}$, 1C), 94.4 (dd, $^{1}J_{FC}$ = 203.5, $^{1}J_{PC}$ = 157.7, $\underline{C}(F)P$, 1C), 85.1 (s, $CO_2\underline{C}Me_3$, 1C), 64.9 (d, $^{2}J_{PC}$ = 6.9, $\underline{C}H_2OP$, 1C), 64.7 (d, $^{2}J_{PC}$ = 7.1, $\underline{C}H_2OP$, 1C), 60.3 (s, $CO_2\underline{C}H_2$, 1C), 49.2 (dd, $^{2}J_{FC}$ = 20.4, $^{2}J_{PC}$ = 6.2, $\underline{C}H_2C(F)P$, 1C), 33.9 (s, $\underline{C}H_2CO_2Et$, 1C), 27.8 (s, $C(\underline{C}H_3)_3$, 3C), 23.6 (s, $\underline{C}H_2CH_2CO_2Et$, 1C), 16.4 (d, $^{3}J_{PC}$ = 5.7, $\underline{C}H_3CH_2OP$, 2C), 14.2 (s, $CO_2CH_2\underline{C}H_3$, 1C). **^{31}P NMR** (101 MHz, $CDCl_3$) δ 10.47 (d, $^{2}J_{PF}$ = 80.0).

Ethyl 5-(1-(3-(*tert*-butoxy)-2-(diethoxyphosphoryl)-2-fluoro-3-oxopropyl)-1*H*-imidazol-4/5-yl)pentanoate (27f): obtained according to the *general procedure of Michael addition and fluorination.* Scale: 130 mg (0.42 mmol) of **24f**. In fluorination step 2.2 eq. of NaH was used. Compound **27f** was purified by flash chromatography using Gilson PLC 2250 purification system. Eluents A:$CHCl_3$, B:Acetone (gradient 0→20 min. 0→5% B, retention time 16 min.). Yield: 50% (100 mg). According to ^{1}H NMR spectrum of the main fraction, the mixture of regioisomer C4 and C5 was obtained in ratio 90:10. Spectroscopic data of regioisomer C4: **^{1}H NMR** (700 MHz, $CDCl_3$) δ 7.38 (s, $C\underline{H}_{Im(2)}$, 1H), 6.68 (s, $C\underline{H}_{Im(5)}$, 1H), 4.63 (ddd, $^{3}J_{FH}$ = 33.1, $^{2}J_{HH}$ = 15.2, $^{3}J_{PH}$ = 5.3, $C\underline{H}_2C(F)P$, 1H), 4.45 (ddd, $^{2}J_{HH}$ = 15.0, $^{3}J_{FH}$ = 14.8, $^{3}J_{PH}$ = 3.7, $C\underline{H}_2C(F)P$, 1H), 4.32 – 4.21 (m, $C\underline{H}_2OP$, 4H), 4.11 (q, $^{3}J_{HH}$ = 7.1, $CO_2C\underline{H}_2$, 2H), 2.54 (t, $^{3}J_{HH}$ = 7.1, $C_{im}C\underline{H}_2$, 2H), 2.31 (dd, $^{3}J_{HH}$ = 7.2, $C\underline{H}_2CO_2$, 2H), 1.69 – 1.62 (m, $C\underline{H}_2C\underline{H}_2CH_2CO_2$, 2H), 1.42 (s, $C(C\underline{H}_3)_3$, 9H), 1.38 (t, $^{3}J_{HH}$ = 7.1, $C\underline{H}_3CH_2OP$, 6H), 1.24 (t, $^{3}J_{HH}$ = 7.1, $CO_2CH_2C\underline{H}_3$, 3H). **^{13}C NMR** (63 MHz, $CDCl_3$) δ 173.7 (s, $\underline{C}O_2Et$, 1C), 163.7 (d, $^{2}J_{FC}$ = 22.1, $\underline{C}O_2tBu$, 1C), 142.7 (s, $\underline{C}_{im(4)}$, 1C), 137.6 (s, $\underline{C}H_{Im(2)}$, 1C), 116.2 (s, $\underline{C}H_{Im(5)}$, 1C), 94.4 (dd, $^{1}J_{FC}$ = 203.0, $^{1}J_{PC}$ = 158.0, $\underline{C}(F)P$, 1C), 85.1 (s, $CO_2\underline{C}Me_3$, 1C), 64.8 (d, $^{2}J_{PC}$ = 6.5, $\underline{C}H_2OP$, 1C), 64.6 (d, $^{2}J_{PC}$ = 7.2, $\underline{C}H_2OP$, 1C), 60.1 (s, $CO_2\underline{C}H_2$, 1C), 49.1 (dd, $^{2}J_{FC}$ = 19.6, $^{2}J_{PC}$ = 5.2, $\underline{C}H_2C(F)P$, 1C), 34.2 (s, $\underline{C}H_2CO_2$, 1C), 28.8 (s, $C_{im(2)}CH_2\underline{C}H_2$, 1C), 27.9 (s, $C_{im(2)}\underline{C}H_2$, 1C), 27.7 (s, $C(\underline{C}H_3)_3$, 3C), 24.6 (s, $\underline{C}H_2CH_2CO_2$, 1C), 16.4 (d, $^{3}J_{PC}$ = 5.8, $\underline{C}H_3CH_2OP$, 2C), 14.2 (s, $CO_2CH_2\underline{C}H_3$, 1C). **^{31}P NMR** (101 MHz, $CDCl_3$) δ 10.99 (d, $^{2}J_{PF}$ = 80.0).

***Tert*-butyl 2-(diethoxyphosphoryl)-2-fluoro-3-(4/5-(3-(methylamino)-3-oxopropyl)-1*H*-imidazol-1-yl)propanoate (27g)**: obtained according to the *general procedure of Michael addition and fluorination*. Scale: 115 mg (0.77 mmol) of **24g**. In Michael addition DMF was used as a solvent. In fluorination step 1.5 eq. of NaH was used. Compound **27g** was purified by flash chromatography using Gilson PLC 2250 purification system. Eluents A:$CHCl_3$, B:Acetone (gradient 4→10 min. 0→20% B, retention time 23 min.). Yield: 36% (120 mg). According to ^{1}H NMR spectrum of the main fraction, the mixture of regioisomer C4 and C5 was obtained in ratio 90:10. Spectroscopic data of regioisomer C4: **^{1}H NMR** (700 MHz, $CDCl_3$) δ 7.23 (s, $C\underline{H}_{Im(2)}$, 1H), 6.84 i 6.83 (2bs, $N\underline{H}$, 1H), 6.57 (s, $C\underline{H}_{Im(5)}$, 1H), 4.48 (ddd, $^3J_{FH}$ = 32.9, $^3J_{HH}$ = 15.3, $^3J_{PH}$ = 5.3, $CH_2C(F)P$, 1H), 4.30 (ddd, $^2J_{HH}$ = 15.0, $^3J_{FH}$ = 15.0, $^3J_{PH}$ = 3.8, $CH_2C(F)P$, 1H), 4.14 – 4.04 (m, $C\underline{H_2}OP$, 4H), 2.67 (t, $^3J_{HH}$ = 7.3, $C_{im(4)}C\underline{H_2}$, 3H), 2.57 (2s, $NHC\underline{H_3}$, 3H), 2.35 (dt, $^3J_{HH}$ = 7.3, $^4J_{HH}$ = 1.1, $C\underline{H_2}CONH$, 2H), 1.26 (s, $CO_2C(CH_3)_3$, 9H), 1.22 (t, $^3J_{HH}$ = 7.1, $C\underline{H_3}CH_2OP$, 6H). **^{13}C NMR** (176 MHz, $CDCl_3$) δ 173.2 (s, $\underline{C}ONHMe$, 1C), 163.4 (dd, $^2J_{FC}$ = 22.4, $^2J_{PC}$ = 2.0, $\underline{C}O_2{}^t$Bu, 1C), 141.3 (s, $\underline{C_{im(4)}}$, 1C), 137.3 (s, $C\underline{H}_{Im(2)}$, 1C), 116.6 (s, $C\underline{H}_{Im(5)}$, 1C), 94.1 (dd, $^1J_{FC}$ = 202.5, $^1J_{PC}$ = 158.1, $\underline{C}(F)P$, 1C), 85.0 (s, $CO_2\underline{C}Me_3$, 1C), 64.7 (d, $^2J_{PC}$ = 6.6, $\underline{C}H_2OP$, 1C), 64.5 (d, $^2J_{PC}$ = 7.0, $\underline{C}H_2OP$, 1C), 49.0 (dd, $^2J_{FC}$ = 20.0, $^2J_{PC}$ = 6.0, $\underline{C}H_2C(F)P$, 1C), 35.9 (s, $\underline{C}H_2CONH$, 1C), 27.5 (s, $C(\underline{C}H_3)_3$, 3C), 25.9 (s, $NH\underline{C}H_3$, 1C), 23.9 (s, $C_{im(4)}\underline{C}H_2$, 1C), 16.2 (d, $^3J_{PC}$ = 5.8, $\underline{C}H_3CH_2OP$, 2C). **^{31}P NMR** (101 MHz, $CDCl_3$) δ 10.21 (d, $^2J_{PF}$ = 80.3).

Ethyl (*E*)-3-(1-(3-(*tert*-butoxy)-2-(diethoxyphosphoryl)-2-fluoro-3-oxopropyl)-1*H*-imidazol-2-yl)acrylate (28a): obtained according to the *general procedure of Michael addition and fluorination*. Scale: 93 mg (0.56 mmol) of **25a**. In fluorination step 1.4 eq. of NaH was used. Compound **28a** was purified by flash chromatography using Gilson PLC 2250 purification system. Eluents A:$CHCl_3$, B:Acetone (gradient 10→18 min. 0→10% B, retention time 12 min.). Yield: 68% (180 mg). **^{1}H NMR** (700 MHz, $CDCl_3$) δ 7.47 (d, $^3J_{HH}$ = 15.3, $C_{im(2)}CH{=}C\underline{H}CO_2$, 1H), 7.10 (s, $C\underline{H}_{Im(4)}$, 1H), 7.06 (s, $C\underline{H}_{Im(5)}$, 1H), 6.82 (d, $^3J_{HH}$ = 15.2, $C_{im}CH{=}C\underline{H}CO_2$, 1H), 4.80 (ddd, $^3J_{FH}$ = 32.0, $^3J_{HH}$ = 15.5, $^3J_{PH}$ = 4.7, $C\underline{H_2}C(F)P$, 1H), 4.54 (ddd, $^3J_{HH}$ = 14.4, $^3J_{FH}$ = 14.4, $^3J_{PH}$ = 3.7, $C\underline{H_2}C(F)P$, 1H), 4.30 – 4.17 (m, $C\underline{H_2}OP$, $CO_2C\underline{H_2}$, 6H), 1.38 – 1.344 (m, $C(C\underline{H_3})_3$, $C\underline{H_3}CH_2OP$, 15H), 1.27 (t, $^3J_{HH}$ = 7.2, $CO_2CH_2C\underline{H_3}$, 3H). **^{13}C NMR** (176 MHz, $CDCl_3$) δ 166.5 (s, $\underline{C}O_2Et$, 1C), 163.3 (d, $^2J_{FC}$ = 22.2, $\underline{C}O_2t$Bu, 1C), 143.5 (s, $\underline{C_{im(2)}}$, 1C), 130.6 (s, $\underline{C}H_{Im(4)}$, 1C), 128.2 (s, $C_{im(2)}CH{=}\underline{C}HCO_2$, 1C), 122.9 (s, $\underline{C}H_{Im(5)}$, 1C), 121.6 (s, $C_{im(2)}C\underline{H}{=}CHCO_2$, 1C), 94.2 (dd, $^1J_{FC}$ = 203.4, $^1J_{PC}$ = 158.7, $\underline{C}(F)P$, 1C), 85.5 (s, $CO_2\underline{C}Me_3$, 1C), 64.9 (d, $^2J_{PC}$ = 6.6, $\underline{C}H_2OP$, 1C), 64.8 (d, $^2J_{PC}$ = 6.7, $\underline{C}H_2OP$, 1C), 60.5 (s, $CO_2\underline{C}H_2$, 1C), 47.6 (dd, $^2J_{FC}$ = 20.2, $^2J_{PC}$ = 6.1, $\underline{C}H_2C(F)P$, 1C), 27.5 (s, $C(\underline{C}H_3)_3$, 3C), 16.3 (d, $^3J_{PC}$ = 5.3, $\underline{C}H_3CH_2OP$, 2C), 14.2 (s, $CO_2CH_2\underline{C}H_3$, 1C). **^{31}P NMR** (283 MHz, $CDCl_3$) δ 10.37 (d, $^2J_{PF}$ = 79.9).

Ethyl (*E*)-5-(1-(3-(*tert*-butoxy)-2-(diethoxyphosphoryl)-2-fluoro-3-oxopropyl)-1*H*-imidazol-2-yl)pent-2-enoate (28b): obtained according to the *general procedure of Michael addition and fluorination*. Scale: 149 mg (0.77 mmol) of **25b**. In fluorination step 1.4 eq. of NaH was used. Compound **28b** was purified by flash chromatography using Gilson PLC 2250 purification system. Eluents A:$CHCl_3$, B:AcOEt (gradient 10→25 min. 0→5% B, retention time 22 min.). Yield: 48% (175 mg). **^{1}H NMR** (700 MHz, $CDCl_3$) δ 7.04 (dt, $^3J_{HH}$ = 15.6, 6.7, $C\underline{H}{=}CHCO_2$, 1H), 6.94 (s, $C\underline{H}_{Im(4)}$, 1H), 6.91 (s, $C\underline{H}_{Im(5)}$, 1H), 5.89 (d, $^3J_{HH}$ = 15.7, $CH{=}C\underline{H}CO_2$, 1H), 4.66 (ddd, $^3J_{FH}$ = 32.5, $^2J_{HH}$ = 15.5, $^3J_{PH}$ = 5.0, $C\underline{H_2}C(F)P$, 1H), 4.43 (ddd, $^2J_{HH}$ = 15.4, $^3J_{FH}$ = 13.2, $^3J_{PH}$ = 3.9, $C\underline{H_2}C(F)P$, 1H), 4.31 – 4.22 (m, $C\underline{H_2}OP$, 4H), 4.18 (q, $^3J_{HH}$ = 7.2, $CO_2C\underline{H_2}$, 2H), 2.85 – 2.80 (m, $C_{im(2)}C\underline{H_2}CH_2$, 2H), 2.77 – 2.70 (m, $C_{im}CH_2C\underline{H_2}$, 2H), 1.41 (s, $C(C\underline{H_3})_3$, 9H), 1.39 (t, $^3J_{HH}$ = 7.1, $C\underline{H_3}CH_2OP$, 6H), 1.28 (t, $^3J_{HH}$ = 7.1, $CO_2CH_2C\underline{H_3}$, 3H); **^{13}C NMR** (63 MHz, $CDCl_3$) δ 166.3 (s, $\underline{C}O_2Et$, 1C), 163.9 (d, $^2J_{FC}$ = 21.6, $\underline{C}O_2t$Bu, 1C), 147.5 (s, $\underline{C_{im(2)}}$, 1C), 147.3 (s, $C\underline{H}{=}CHCO_2$, 1C), 127.6 (s, $\underline{C}H_{Im(4)}$, 1C), 122.0 (s, $CH{=}C\underline{H}CO_2$, 1C), 120.1 (s, $\underline{C}H_{Im(5)}$, 1C), 94.6 (dd, $^1J_{FC}$ = 205.0, $^1J_{PC}$ = 158.2, $\underline{C}(F)P$, 1C), 85.1 (s, $CO_2\underline{C}Me_3$, 1C), 64.8 (d, $^2J_{PC}$ = 6.9, $\underline{C}H_2OP$, 1C), 64.7 (d, $^2J_{PC}$ = 7.2, $\underline{C}H_2OP$, 1C), 60.1 (s, $CO_2\underline{C}H_2$, 1C), 47.5 (dd, $^2J_{FC}$ = 19.9, $^2J_{PC}$ = 6.2, $\underline{C}H_2C(F)P$, 1C), 29.7 (s, $C_{im(2)}CH_2\underline{C}H_2$, 1C), 27.6 (s, $C(\underline{C}H_3)_3$, 3C), 24.9 (s,

$C_{im(2)}\underline{C}H_2CH_2$, 1C), 16.3 (d, $^3J_{PC}$ = 5.8, $\underline{C}H_3CH_2OP$, 2C), 14.2 (s, $CO_2CH_2\underline{C}H_3$, 1C). **^{31}P NMR** (101 MHz, $CDCl_3$) δ 10.46 (d, $^2J_{PF}$ = 79.8).

***Tert*-butyl (*E*)-2-(diethoxyphosphoryl)-2-fluoro-3-(2-(3-(methylamino)-3-oxoprop-1-en-1-yl)-1*H*-imidazol-1-yl)propanoate (28c)**: obtained according to the *general procedure of Michael addition and fluorination*. Scale: 65 mg (0.43 mmol) of **25c**. In Michael addition DMF was used as a solvent. In fluorination step 1.5 eq. of NaH was used. Compound **28c** was purified by flash chromatography using Gilson PLC 2250 purification system. Eluents A: $CHCl_3$, B: Acetone (gradient 5→10 min. 0→20% B, retention time 17 min.). Yield: 48% (90 mg). **^{1}H NMR** (250 MHz, $CDCl_3$) δ 7.45 (d, $^3J_{HH}$ = 14.9, $C_{im(2)}CH{=}C\underline{H}$, 1H), 7.04 – 7.02 (m, $C\underline{H}_{im}{=}C\underline{H}_{im}$, 2H), 6.91 (d, $^3J_{HH}$ = 15.0, $C_{im(2)}C\underline{H}{=}$, 1H), 6.67 i 6.66 (2bs, N$\underline{H}$, 1H), 4.81 (ddd, $^3J_{FH}$ = 32.1, $^3J_{HH}$ = 15.5, $^3J_{PH}$ = 4.9, $C\underline{H_2}C(F)P$, 1H), 4.54 (ddd, $^3J_{HH}$ = 15.5, $^3J_{FH}$ =13.2, $^3J_{PH}$ = 4.0, 1H), 4.28 – 4.19 (m, $C\underline{H_2}OP$, 4H), 2.834 i 2.827 (2s, $NHC\underline{H_3}$, 3H), 1.36 (s, $CO_2C(CH_3)_3$, 9H), 1.34 (t, $^3J_{HH}$ = 7.1, $C\underline{H_3}CH_2OP$, 6H). **^{13}C NMR** (176 MHz, $CDCl_3$) δ 165.9 (s, $\underline{C}ONHMe$, 1C), 163.4 (d, $^2J_{FC}$ = 22.6, $\underline{C}O_2{}^tBu$, 1C), 144.3 (s, $\underline{C_{im(2)}}$, 1C), 129.9 (s, $\underline{C}H_{im}$, 1C), 125.0 (s, $C_{im}\underline{C}H{=}$, 1C), 124.5 (s, $C_{im}CH{=}\underline{C}H$, 1C), 122.6 (s, $\underline{CH_{im}}$, 1C), 94.3 (dd, $^1J_{FC}$ = 202.0, $^1J_{PC}$ = 158.6, $\underline{C}(F)P$, 1C), 85.6 (s, $CO_2\underline{C}Me_3$, 1C), 65.1 (d, $^2J_{PC}$ = 7.0, $\underline{C}H_2OP$, 1C), 65.0 (d, $^2J_{PC}$ = 7.4, $\underline{C}H_2OP$, 1C), 47.7 (dd, $^2J_{FC}$ = 19.4, $^2J_{PC}$ = 6.1, $\underline{C}H_2C(F)P$, 1C), 27.7 (s, $C(\underline{C}H_3)_3$, 3C), 26.51 and 26.50 (2s, $NH\underline{C}H_3$, 1C), 16.5 (d, $^3J_{PC}$ = 5.2, $\underline{C}H_3CH_2OP$, 2C). **^{31}P NMR** (283 MHz, $CDCl_3$) δ 9.84 (d, $^2J_{PF}$ = 81.6).

***Tert*-butyl 2-(diethoxyphosphoryl)-3-(2-(3-ethoxy-3-oxopropyl)-1*H*-imidazol-1-yl)-2-fluoropropanoate (28d):** obtained according to the *general procedure of Michael addition and fluorination*. Scale: 100 mg (0.60 mmol) of **25d**. Compound **28d** was purified by flash chromatography using Gilson PLC 2250 purification system. As eluent mixture of A: $CHCl_3$ and B: Acetone was used (gradient 10→18 min. 0→5% B, retention time 19 min.). Yield: 55% (146 mg). **^{1}H NMR** (250 MHz, $CDCl_3$) δ 6.96 – 6.85 (m, $C\underline{H_{im}}$, 2H), 4.72 (ddd, $^3J_{FH}$ = 32.2, $^2J_{HH}$ = 15.5, $^3J_{PH}$ = 5.1, $C\underline{H_2}C(F)P$, 1H), 4.51 (ddd, $^2J_{HH}$ = 15.5, $^3J_{FH}$ = 13.8, $^3J_{PH}$ = 4.0, $C\underline{H_2}C(F)P$, 1H), 4.36 – 4.19 (m, $C\underline{H_2}OP$, 4H), 4.12 (q, $^3J_{HH}$ = 7.1, $CO_2C\underline{H_2}$, 2H), 3.11 – 2.91 (m, $\underline{C}H_2CH_2CO_2Et$, 2H), 2.89 – 2.80 (m, $\underline{C}H_2CO_2Et$, 2H), 1.42 (s, $C(C\underline{H_3})_3$, 9H), 1.39 (t, $^3J_{HH}$ = 7.1, $C\underline{H_3}CH_2OP$, 6H), 1.24 (t, $^3J_{HH}$ = 7.1, $CO_2CH_2C\underline{H_3}$, 3H). **^{13}C NMR** (176 MHz, $CDCl_3$) δ 172.8 (s, $\underline{C}O_2Et$, 1C), 164.1 (d, $^2J_{FC}$ = 21.4, $\underline{C}O_2tBu$, 1C), 147.8 (s, $\underline{C_{im}}$, 1C), 127.8 (s, $\underline{C}H_{Im(4)}$, 1C), 120.2 (s, $\underline{C}H_{Im(5)}$, 1C), 94.8 (dd, $^1J_{FC}$ = 205.1, $^1J_{PC}$ = 156.7, $\underline{C}(F)P$, 1C), 85.2 (s, $CO_2\underline{C}Me_3$, 1C), 64.9 (d, $^2J_{PC}$ = 6.4, $\underline{C}H_2OP$, 1C), 64.8 (d, $^2J_{PC}$ = 6.6, $\underline{C}H_2OP$, 1C), 60.6 (s, $CO_2\underline{C}H_2$, 1C), 47.7 (dd, $^2J_{FC}$ = 19.5, $^2J_{PC}$ = 4.9, $\underline{C}H_2C(F)P$, 1C), 32.0 (s, $\underline{C}H_2CO_2Et$, 1C), 27.8 (s, $C(\underline{C}H_3)_3$, 3C), 21.6 (s, $\underline{C}H_2CH_2CO_2Et$, 1C), 16.5 (d, $^3J_{PC}$ = 5.3, $\underline{C}H_3CH_2OP$, 2C), 14.2 (s, $CO_2CH_2\underline{C}H_3$, 1C). **^{31}P NMR** (283 MHz, $CDCl_3$) δ 10.92 (d, $^2J_{PF}$ = 78.9).

Ethyl 5-(1-(3-(*tert*-butoxy)-2-(diethoxyphosphoryl)-2-fluoro-3-oxopropyl)-1*H*-imidazol-2-yl)pentanoate (28e): obtained according to the *general procedure of Michael addition and fluorination*. Scale: 0.27 mmol of **25d**. In fluorination step 1.4 eq. of NaH was used. Compound **28d** was purified by flash chromatography using Gilson PLC 2250 purification system. Eluents A: $CHCl_3$, B: Acetone (gradient 5→15 min. 0→10% B, retention time 9 min.). Yield: 47% (60 mg). **^{1}H NMR** (250 MHz, $CDCl_3$) δ 6.91 (d, $^3J_{HH}$ = 1.4, $C\underline{H}_{Im(4)}$, 1H), 6.89 (t, $^3J_{HH}$ = 1.5, $C\underline{H}_{Im(5)}$, 1H), 4.66 (ddd, $^3J_{FH}$ = 32.6, $^2J_{HH}$ = 15.4, $^3J_{PH}$ = 4.8, $C\underline{H_2}C(F)P$, 1H), 4.42 (ddd, $^2J_{HH}$ = 15.4, $^3J_{FH}$ = 13.4, $^3J_{PH}$ = 4.0, $C\underline{H_2}C(F)P$, 1H), 4.35 – 4.19 (m, $C\underline{H_2}OP$, 4H), 4.11 (q, $^3J_{HH}$ = 7.1, $CO_2C\underline{H_2}$, 2H), 2.76 – 2.64 (m, $C_{im(2)}C\underline{H_2}$, 2H), 2.35 (t, $^3J_{HH}$ = 7.2, $C\underline{H_2}CO_2$, 2H), 1.92 – 1.62 ($C\underline{H_2}C\underline{H_2}CH_2CO_2$, 4H), 1.41 (s, $C(C\underline{H_3})_3$, 9H), 1.39 (t, $^3J_{HH}$ = 7.1, $C\underline{H_3}CH_2OP$, 6H), 1.24 (t, $^3J_{HH}$ = 7.1, $CO_2CH_2C\underline{H_3}$, 3H). **^{13}C NMR** (63 MHz, $CDCl_3$) δ 173.4 (s, $\underline{C}O_2Et$, 1C), 164.0 (d, $^2J_{FC}$ = 21.6, $\underline{C}O_2tBu$, 1C), 148.8 (s, $\underline{C_{im(2)}}$, 1C), 127.6 (s, $\underline{C}H_{Im(4)}$, 1C), 119.8 (s, $\underline{C}H_{Im(5)}$, 1C), 94.7 (dd, $^1J_{FC}$ = 205.1, $^1J_{PC}$ = 158.2, $\underline{C}(F)P$, 1C), 85.1 (s, $CO_2\underline{C}Me_3$, 1C), 64.9 (d, $^2J_{PC}$ = 6.9, $\underline{C}H_2OP$, 1C), 64.7 (d, $^2J_{PC}$ = 7.3, $\underline{C}H_2OP$, 1C), 60.2 (s, $CO_2\underline{C}H_2$, 1C), 47.5 (dd, $^2J_{FC}$ = 20.0, $^2J_{PC}$ = 6.3, $\underline{C}H_2C(F)P$, 1C), 34.0 (s, $\underline{C}H_2CO_2$, 1C), 27.6 (s, $C(\underline{C}H_3)_3$, 3C),

27.1 (s, $C_{im(2)}CH_2\underline{C}H_2$, 1C), 26.1 (s, $C_{im(2)}\underline{C}H_2$, 1C), 24.7 (s, $\underline{C}H_2CH_2CO_2$, 1C), 16.4 (d, $^3J_{PC}$ = 5.7, $\underline{C}H_3CH_2OP$, 2C), 14.2 (s, $CO_2CH_2\underline{C}H_3$, 1C). **^{31}P NMR** (101 MHz, $CDCl_3$) δ 10.59 (d, $^2J_{PF}$ = 80.2).

***Tert*-butyl 2-(diethoxyphosphoryl)-2-fluoro-3-(2-(3-(methylamino)-3-oxopropyl)-1*H*-imidazol-1-yl)propanoate (28f)**: obtained according to the *general procedure of Michael addition and fluorination*. Scale: 0.68 mmol of **25f**. In Michael addition DMF was used as a solvent. In fluorination step 1.7 eq. of NaH was used. Compound **28f** was purified by flash chromatography using Gilson PLC 2250 purification system. Eluents A: $CHCl_3$, B: Acetone (gradient 5→10 min. 0→20% B, retention time 24 min.). Yield: 32% (95 mg). **^{1}H NMR** (700 MHz, $CDCl_3$) δ 6.90 – 6.85 (m, $C\underline{H}_{im}=C\underline{H}_{im}$, N$\underline{H}$, 3H), 4.68 (ddd, $^3J_{FH}$ = 32.2, $^3J_{HH}$ =15.6, $^3J_{PH}$ =5.3, $C\underline{H}_2C(F)P$, 1H), 4.50 (ddd, $^3J_{HH}$ = 15.6, $^3J_{FH}$ = 13.6, $^3J_{PH}$ = 4.0, $C\underline{H}_2C(F)P$, 1H), 4.29 – 4.18 (m, $C\underline{H}_2OP$, 4H), 2.97 (t, $^3J_{HH}$ = 6.5, $C\underline{H}_2CON$, 2H), 2.73 – 2.70 (m, $C_{im(2)}C\underline{H}_2$, 2H), 2.691 and 2.684 (2s, $NHC\underline{H}_3$, 3H), 1.38 (s, $CO_2C(CH_3)_3$, 9H), 1.35 (t, $^3J_{HH}$ = 7.1, $C\underline{H}_3CH_2OP$, 6H). **^{13}C NMR** (176 MHz, $CDCl_3$) δ 172.8 (s, $\underline{C}$ONHMe, 1C), 164.0 (d, $^2J_{FC}$ = 22.0, $\underline{C}O_2{}^tBu$, 1C), 148.4 (s, $\underline{C}_{im(2)}$, 1C), 126.8 (s, CH_{im}, 1C), 120.4 (s, $\underline{C}H_{im}$, 1C), 94.7 (dd, $^1J_{FC}$ = 204.0, $^1J_{PC}$ = 158.6, $\underline{C}(F)P$, 1C), 85.3 (s, $CO_2\underline{C}Me_3$, 1C), 65.0 (d, $^2J_{PC}$ = 7.2, $\underline{C}H_2OP$, 1C), 64.9 (d, $^2J_{PC}$ = 6.5, $\underline{C}H_2OP$, 1C), 47.8 (dd, $^2J_{FC}$ = 20.2, $^2J_{PC}$ = 5.9, $\underline{C}H_2C(F)P$, 1C), 33.6 (s, $C\underline{H}_2CON$, 1C), 27.8 (s, $C(\underline{C}H_3)_3$, 3C), 26.27 and 26.25 (2s, $NH\underline{C}H_3$, 1C), 22.2 (s, $C_{im(2)}C\underline{H}_2$, 1C), 16.5 (d, $^3J_{PC}$ = 5.2, $\underline{C}H_3CH_2OP$, 2C). **^{31}P NMR** (283 MHz, $CDCl_3$) δ 10.05 (d, $^2J_{PF}$ = 81.4).

Sodium (*E*)-3-(4-(3-ethoxy-3-oxoprop-1-en-1-yl)-1*H*-imidazol-1-yl)-2-fluoro-2-phosphonopropanoate (4a): obtained according to the general procedure of ester deprotection. Reaction with BTMS carried out in presence of TEA (10 eq). Scale: 80 mg of **27a**. Product **4a** was obtained as a sodium salt using crystallization from mixture of H_2O (pH 7 adjusted by 1M NaOH) and EtOH. Yield: 88% (63 mg). According to signals in ^{1}H NMR spectrum single regioisomer C4 was obtained with no traces of regioisomer C5. **^{1}H NMR** (700 MHz, D_2O pH 7) δ 7.96 (s, $C\underline{H}_{Im(2)}$, 1H), 7.65 (d, $^3J_{HH}$ = 16.0, $C_{im}C\underline{H}$=, 1H), 7.57 (s, $C\underline{H}_{Im(5)}$, 1H), 6.46 (d, $^3J_{HH}$ = 15.9, $C_{im(4)}CH=C\underline{H}$, 1H), 4.91 (bdd, $^3J_{FH}$ = 33.3, $^2J_{HH}$ = 14.8, $C\underline{H}_2C(F)P$, 1H), 4.58 (bdd, J = 11.7, $C\underline{H}_2C(F)P$, 1H), 4.30 (q, $^3J_{HH}$ = 7.2, $CO_2C\underline{H}_2$, 2H), 1.35 (t, $^3J_{HH}$ = 7.3, $CO_2CH_2C\underline{H}_3$, 3H). **^{13}C NMR** (176 MHz, D_2O pH 7) δ 172.6 (s, $\underline{C}O_2H$, 1C), 169.5 (s, $\underline{C}O_2Et$, 1C), 140.3 (s, $\underline{C}H_{Im(2)}$, 1C), 135.4 (s, $\underline{C}H$=CH, 1C), 134.9 (s, $\underline{C}_{im}$, 1C), 124.7 (s, $\underline{C}H_{Im(5)}$, 1C), 115.9 (s, $\underline{C}H$=CH, 1C), 95 (m, $\underline{C}(F)P$, 1C, in HMBC spectrum), 61.7 (s, $CO_2\underline{C}H_2$, 1C), 51.2 (bd, $^2J_{FC}$ = 20.0, $\underline{C}H_2C(F)P$, 1C), 13.4 (s, $CO_2CH_2\underline{C}H_3$, 1C). **^{31}P NMR** (283 MHz, D_2O, pH 7) δ 7.96 (d, $^2J_{PF}$ = 64.7). Elemental analysis: $C_{11}H_{11}FN_2Na_3O_7P$*0.2C_2H_6O*3.2H_2O: calculated C29.19; H4.00; N5.97; found C29.19; H3.60; N5.85; max. diff. 0.40.

Sodium (*E*)-3-(4-(5-ethoxy-5-oxopent-3-en-1-yl)-1*H*-imidazol-1-yl)-2-fluoro-2-phosphonopropanoate (4b): obtained according to the general procedure of ester deprotection. Reaction with BTMS carried out in presence of TEA (10 eq). Scale: 60 mg of **27b** (mixture of regioizomers C4 and C5). Product **4b** was initially purified by crystallization as a sodium salt from mixture of H_2O (pH 7 adjusted by 1M NaOH) and EtOH. Futher purification was carried out by preparative HPLC (gradient 2→20 min. 5→20% B, retention time 14.4 min.) followed by lyophilization. In order to prevent potential spontaneous hydrolysis of the ethyl ester group, product **4b** was converted to sodium salt by using crystallization from mixture of H_2O (pH 7 adjusted by 1M NaOH) and EtOH. Yield: 28% (12 mg). According to ^{1}H NMR spectrum of main HPLC fraction single regioisomer C4 was obtained with no traces of regioisomer C5. **^{1}H NMR** (700 MHz, D_2O pH 2) δ 8.75 (s, $C\underline{H}_{Im(2)}$, 1H), 7.34 (s, $C\underline{H}_{Im(5)}$, 1H), 7.00 (dt, $^3J_{HH}$ = 15.7, 6.9, $C\underline{H}=CHCO_2$, 1H), 5.92 (dt, $^3J_{HH}$ = 15.7, $^4J_{HH}$ = 1.5, $CH=C\underline{H}CO_2$, 1H), 5.03 (ddd, $^3J_{FH}$ = 32.5, $^2J_{HH}$ = 15.3, $^3J_{PH}$ = 3.9, $C\underline{H}HC(F)P$, 1H), 4.85 – 4.81 (m, $CH\underline{H}C(F)P$, 1H), 4.23 (q, $^3J_{HH}$ = 7.1, $CO_2C\underline{H}_2$, 2H), 2.94 (t, $^3J_{HH}$ = 7.0, $C_{im(4)}C\underline{H}_2$, 2H), 2.67 – 2.61 (m, $C_{im(4)}CH_2C\underline{H}_2$, 2H), 1.30 (t, $^3J_{HH}$ = 7.1, $CO_2CH_2C\underline{H}_3$, 3H). **^{13}C NMR** (176 MHz, D_2O pH 3) δ 169.8 (d, $^2J_{FC}$ = 23.7, $\underline{C}O_2H$, 1C), 168.8 (s, $\underline{C}O_2Et$, 1C), 148.0 (s, $C\underline{H}=CHCO_2$, 1C), 135.3 (d,

J = 5.1, C̲H$_{Im(2)}$, 1C), 133.5 (s, C̲$_{im(4)}$, 1C), 122.1 (s, CH=C̲HCO$_2$, 1C), 119.6 (s, C̲H$_{Im(5)}$, 1C), 95.2 (dd, $^1J_{FC}$ = 196.0, $^1J_{PC}$ = 139.5, C̲(F)P, 1C), 61.7 (s, CO$_2$C̲H$_2$, 1C), 51.6 (dd, $^2J_{FC}$ = 20.0, $^2J_{PC}$ = 6.0, C̲H$_2$C(F)P, 1C), 30.1 (s, C$_{im(4)}$CH$_2$C̲H$_2$, 1C), 22.4 (s, C$_{im(4)}$C̲H$_2$, 1C), 13.3 (s, CO$_2$CH$_2$C̲H$_3$, 1C). **^{31}P NMR** (283 MHz, D$_2$O, pH 2) δ 4.26 (d, $^2J_{PF}$ = 67.6). **(*E*)-3-(5-(5-ethoxy-5-oxopent-3-en-1-yl)-1*H*-imidazol-1-yl)-2-fluoro-2-phosphonopropanoic acid:** The regioisomer C5 was obtained as the minor HPLC fraction: **^{1}H NMR** (700 MHz, D$_2$O pH 2) δ 8.74 (bs, CH̲$_{Im(2)}$, 1H), 7.31 (d, $^3J_{HH}$ = 1.4, CH̲$_{Im(4)}$, 1H), 7.07 (dt, $^3J_{HH}$ = 15.8, 6.7, CH̲=CHCO$_2$, 1H), 6.01 (dt, $^3J_{HH}$ = 15.8, $^4J_{HH}$ = 1.6, CH=CH̲CO$_2$, 1H), 5.06 (ddd, $^3J_{FH}$ = 31.9, $^2J_{HH}$ = 15.8, $^3J_{PH}$ = 4.6, CH̲HC(F)P, 1H), 4.85 – 4.81 (m, CHH̲C(F)P, 1H), 4.24 (q, $^3J_{HH}$ = 7.2, CO$_2$CH̲$_2$, 2H), 3.01-2.98 (m, C$_{im(5)}$CH̲$_2$, 2H), 2.73 – 2.68 (m, C$_{im(5)}$CH$_2$CH̲$_2$, 2H), 1.37 (t, $^3J_{HH}$ = 7.2, CO$_2$CH$_2$CH̲$_3$, 3H).

(*E*)-2-fluoro-3-(4-(3-(methylamino)-3-oxoprop-1-en-1-yl)-1*H*-imidazol-1-yl)-2-phosphonopropanoic acid (4c): obtained according to the general procedure of ester deprotection. Scale: 80 mg of **27c**. Product **4c** was purified by crystallization from mixture of H$_2$O (pH 2) and EtOH followed by additional lyophilization from H$_2$O. Yield: 74% (44 mg). According to ^{1}H NMR spectrum single regioisomer C4 was obtained with no traces of regioisomer C5. **^{1}H NMR** (700 MHz, D$_2$O pH 7) δ 7.74 (s, CH̲$_{Im(2)}$, 1H), 7.45 (s, CH̲$_{Im(5)}$, 1H), 7.40 (d, $^3J_{HH}$ = 15.6, CH̲=, 1H), 6.53 (d, $^3J_{HH}$ = 15.6, CH̲=, 1H), 6.57 (ddd, $^3J_{FH}$ = 36.1, $^2J_{HH}$ = 15.0, $^3J_{PH}$ = 1.1, CH̲$_2$C(F)P, 1H), 4.48 (ddd, $^2J_{HH}$ = 14.9, $^3J_{FH}$ = 8.9, $^3J_{PH}$ = 4.5, CH̲$_2$C(F)P, 1H), 2.86 (s, NHM̲e̲, 3H). **^{13}C NMR** (176 MHz, D$_2$O) δ 174.7 (dd, $^2J_{FC}$ = 21.3, $^2J_{PC}$ = 1.4, CFC̲O$_2$, 1C), 169.7 (s, C̲ONH, 1C), 140.6 (s, C̲H$_{Im(2)}$, 1C), 136.1 (s, C̲$_{im(4)}$, 1C), 132.1 (s, CH=C̲H, 1C), 124.0 (s, C̲H$_{Im(5)}$, 1C), 117.3 (s, CH=C̲H, 1C), 98.9 (dd, $^1J_{FC}$ = 193.0, $^1J_{PC}$ = 133.2, C̲(F)P, 1C), 52.1 – 51.2 (m, C̲H$_2$C(F)P, 1C), 26.0 (s, NHC̲H$_3$, 1C). **^{31}P NMR** (283 MHz, D$_2$O, pH 7) δ 8.08 (d, $^2J_{PF}$ = 72.6). Elemental analysis: $C_{10}H_{13}FN_3O_6P$*0.5H_2O: calculated C36.37; H4.27; N12.73; found C36.19; H4.05; N12.52; max. diff. 0.22.

(*E*)-3-(4-(3-amino-3-oxoprop-1-en-1-yl)-1*H*-imidazol-1-yl)-2-fluoro-2-phosphonopropanoic acid (4d): obtained according to the general procedure of ester deprotection. Scale: 70 mg of **27d**. Product **4d** was purified by crystallization from mixture of H$_2$O (pH 2) and EtOH followed by additional lyophilization from H$_2$O. Yield: 57% (29 mg). According to ^{1}H NMR spectrum single regioisomer C4 was obtained with no traces of regioisomer C5. **^{1}H NMR** (700 MHz, D$_2$O pH 7) δ 8.89 (s, CH̲$_{Im(52)}$, 1H), 7.83 (s, CH̲$_{Im(5)}$, 1H), 7.42 (d, $^3J_{HH}$ = 16.1, CH̲CONH$_2$, 1H), 6.73 (d, $^3J_{HH}$ = 16.1, C$_{im(4)}$CH̲, 1H), 5.06 (ddd, $^3J_{FH}$ = 31.9, $^2J_{HH}$ = 15.1, $^3J_{PH}$ = 4.3, CH̲$_2$C(F)P, 1H), 4.85 – 4.80 (m, CH̲$_2$C(F)P, 1H). **^{13}C NMR** (176 MHz, D$_2$O pH 7) δ 173.0 (d, $^2J_{FC}$ = 20.4, CFC̲O$_2$, 1C), 171.4 (s, C̲ONH$_2$, 1C), 140.5 (s, C̲H$_{Im(2)}$, 1C), 135.9 (s, C̲$_{Im(4)}$, 1C), 133.3 (s, C̲HCONH$_2$, 1C), 124.2 (s, C̲H$_{Im(5)}$, 1C), 116.9 (s, C$_{Im(4)}$C̲H, 1C), 97.4 (dd, $^1J_{FC}$ = 194.5, $^1J_{PC}$ = 141.7, C̲(F)P, 1C), 51.1 (dd, $^2J_{FC}$ = 20.5, $^2J_{PC}$ = 8.0, C̲H$_2$C(F)P, 1C). **^{31}P NMR** (283 MHz, D$_2$O pH 7) δ 5.85 (d, $^2J_{PF}$ = 70.9).

Sodium 3-(4-(3-ethoxy-3-oxopropyl)-1*H*-imidazol-1-yl)-2-fluoro-2-phosphonopropanoate (4e): obtained according to the general procedure of ester deprotection. Scale: 100 mg of **27e**. Product **4e** was obtained as a sodium salt by crystallization from mixture of H$_2$O (pH 7 adjusted by 1M NaOH) and EtOH. Yield: 79% (70 mg). According to ^{1}H NMR spectrum single regioisomer C4 was obtained with no traces of regioisomer C5. **^{1}H NMR** (700 MHz, D$_2$O pH 7) δ 8.66 (s, CH̲$_{Im(2)}$, 1H), 7.30 (s, CH̲$_{Im(5)}$, 1H), 4.98 (ddd, $^3J_{FH}$ = 32.7, $^2J_{HH}$ = 14.8, $^3J_{PH}$ = 3.1, CH̲$_2$C(F)P, 1H), 4.74 – 4.67 (m, CH̲$_2$C(F)P, 1H), 4.18 (q, $^3J_{HH}$ = 7.2, CO$_2$CH̲$_2$, 2H), 3.03 (t, $^3J_{HH}$ = 7.1, CH̲$_2$CH$_2$CO$_2$, 2H), 2.80 (t, $^3J_{HH}$ = 7.2, CH̲$_2$CO$_2$, 2H), 1.25 (t, $^3J_{HH}$ = 7.2, CO$_2$CH$_2$CH̲$_3$, 3H). **^{13}C NMR** (176 MHz, D$_2$O pH 7) δ 174.7 (s, C̲O$_2$Et, 1C), 172.0 (d, $^2J_{FC}$ = 21.6, C̲O$_2$H, 1C), 135.1 (s, C̲H$_{Im(2)}$, 1C), 133.1 (s, C̲$_{im}$, 1C), 119.5 (s, C̲H$_{Im(5)}$, 1C), 96.7 (dd, $^1J_{FC}$ = 196.6, $^1J_{PC}$ = 142.6, C̲(F)P, 1C), 62.0 (s, CO$_2$C̲H$_2$, 1C), 52.7 (dd, $^2J_{FC}$ = 20.5, $^2J_{PC}$ = 8.1, C̲H$_2$C(F)P, 1C), 32.6 (s, C$_{im(4)}$CH$_2$C̲H$_2$, 1C), 19.6 (s, C$_{im}$C̲H$_2$, 1C), 13.3 (s, CO$_2$CH$_2$C̲H$_3$, 1C). **^{31}P NMR NMR** (283 MHz, D$_2$O, pH 7) δ 6.40 (d, $^2J_{PF}$ = 69.9).

Sodium 3-(4-(5-ethoxy-5-oxopentyl)-1*H*-imidazol-1-yl)-2-fluoro-2-phosphonopropanoate (4f): obtained according to the general procedure of ester deprotection. Scale: 95 mg of **27f**. Product **4f** was purified by preparative HPLC (gradient 2→20 min. 5→20% B, retention time 13.5 min.) followed by lyophilization. In order to prevent potential spontaneous hydrolysis of the ethyl ester group, product **4f** was converted to sodium salt by using crystallization from mixture of H_2O (pH 7 adjusted by 1M NaOH) and EtOH. Yield: 40% (49 mg). According to ^{1}H NMR spectrum of main HPLC fraction single regioisomer C4 was obtained with no traces of regioisomer C5. **^{1}H NMR** (700 MHz, D_2O pH 3) δ 8.71 (s, $C\underline{H}_{im}$, 1H), 7.31 (s, $C\underline{H}_{im}$, 1H), 5.02 (ddd, $^3J_{FH}$ = 33.0, $^2J_{HH}$ = 15.4, $^3J_{PH}$ = 3.9, $C\underline{H}_2C(F)P$, 1H), 4.79 (m, $C\underline{H}_2C(F)P$, 1H), 4.19 (q, $^3J_{HH}$ = 7.2, $CO_2C\underline{H}_2$, 2H), 2.76 (t, $^3J_{HH}$ = 7.0, $C_{im}C\underline{H}_2$, 2H), 2.44 (t, $^3J_{HH}$ = 7.2, $C\underline{H}_2CO_2$, 2H), 1.74 – 1.68 (m, $C\underline{H}_2CH_2$, 2H), 1.68 – 1.62 (m, $C\underline{H}_2CH_2$, 2H), 1.28 (t, $^3J_{HH}$ = 7.2, $CO_2CH_2C\underline{H}_3$, 3H). **^{13}C NMR** (176 MHz, D_2O pH 3) δ 176.8 (s, $\underline{C}O_2Et$, 1C), 172.5 (d, $^2J_{FC}$ = 21.9, $\underline{C}O_2H$, 1C), 134.7 (s, $\underline{C}H_{im}$, 1C), 134.3 (s, $\underline{C}_{im(4)}$, 1C), 119.1 (s, $\underline{C}H_{im}$, 1C), 97.1 (dd, $^1J_{FC}$ = 195.9, $^1J_{PC}$ = 140.8, $\underline{C}(F)P$, 1C), 61.7 (s, $CO_2\underline{C}H_2$, 1C), 52.9 (dd, $^2J_{FC}$ = 20.9, $^2J_{PC}$ = 7.1, $\underline{C}H_2C(F)P$, 1C), 33.5 (s, $\underline{C}H_2CO_2$, 1C), 26.9 (s, $C_{im(4)}\underline{C}H_2$, 1C), 23.6 (s, $CH_2\underline{C}H_2$, 1C), 23.5 (s, $CH_2\underline{C}H_2$, 1C), 13.3 (s, $CO_2CH_2\underline{C}H_3$, 1C). **^{31}P NMR** (283 MHz, D_2O, pH 3) δ 5.13 (d, $^2J_{PF}$ = 69.2). Elemental analysis: $C_{13}H_{17}FN_2Na_3O_7P$*$0.35H_2O$: calculated C35.61; H4.07; N6.39; found C35.60; H4.09; N6.43; max. diff. 0.04.

2-fluoro-3-(4-(3-(methylamino)-3-oxopropyl)-1*H*-imidazol-1-yl)-2-phosphonopropanoic acid (4g): obtained according to the general procedure of ester deprotection. Scale: 80 mg of **27g**. Product **4g** was initially purified by crystallization from mixture of H_2O (pH 2) and EtOH followed by additional lyophilization from H_2O. Futher purification was carried out by preparative HPLC (eluent A: H_2O:ACN 95:5, isocratic, retention time 4.3 min.) followed by lyophilization. Yield: 64% (33 mg). According to ^{1}H NMR spectrum single regioisomer C4 was obtained with no traces of regioisomer C5. **^{1}H NMR** (700 MHz, D_2O pH 7) δ 8.73 (s, $C\underline{H}_{Im(2)}$, 1H), 7.31 (s, $C\underline{H}_{Im(5)}$, 1H), 5.02 (ddd, $^3J_{FH}$ = 32.6, $^2J_{HH}$ = 15.2, $^3J_{PH}$ = 3.7, $C\underline{H}_2C(F)P$, 1H), 4.79 – 4.75 (m, $C\underline{H}_2C(F)P$, 1H), 3.03 (t, $^3J_{HH}$ = 7.2, $C_{im(4)}C\underline{H}_2$, 2H), 2.73 (s, NH$\underline{Me}$, 3H), 2.64 (t, $^3J_{HH}$ = 7.3, $C\underline{H}_2CONH$, 2H). **^{13}C NMR** (176 MHz, D_2O pH 7) δ 174.5 (s, $\underline{C}ONH$, 1C), 170.0 (d, $^2J_{FC}$ = 23.1, $CF\underline{C}O_2$, 1C), 135.4 (s, $\underline{C}H_{Im(2)}$, 1C), 133.0 (s, $\underline{C}_{im(4)}$, 1C), 119.5 (s, $\underline{C}H_{Im(5)}$, 1C), 95.3 (dd, $^1J_{FC}$ = 196.1, $^1J_{PC}$ = 139.6, $\underline{C}(F)P$, 1C), 52.0 – 51.5 (m, $\underline{C}H_2C(F)P$, 1C), 34.0 (s, CH_2CONH, 1C), 25.8 (s, $NH\underline{C}H_3$, 1C), 20.3 (s, $C_{im(4)}\underline{C}H_2$, 1C). **^{31}P NMR** (283 MHz, D_2O, pH 7) δ 5.45 (d, $^2J_{PF}$ = 68.5). Elemental analysis: $C_{10}H_{15}FN_3O_6P$*$1.4H_2O$: calculated C34.47; H5.15; N12.06; found C34.54; H5.12; N11.,95; max. diff. 0.11.

Sodium (*E*)-3-(2-(3-ethoxy-3-oxoprop-1-en-1-yl)-1*H*-imidazol-1-yl)-2-fluoro-2-phosphonopropanoate (5a): obtained according to the general procedure of ester deprotection. Reaction with BTMS carried out in presence of TEA (10 eq). Scale: 50 mg of **28a**. Product **5a** was obtained as sodium salt by crystallization from mixture of H_2O (pH 7 adjusted by 1M NaOH) and EtOH. Yield: 69% (30 mg). **^{1}H NMR** (700 MHz, D_2O pH 7) δ 7.75 (dd, $^3J_{HH}$ = 15.4, J_{HH} = 1.0, $C_{im(2)}C\underline{H}$=, 1H), 7.51 (s, $C\underline{H}_{im}$, 1H), 7.44 (s, $C\underline{H}_{im}$, 1H), 6.83 (d, $^3J_{HH}$ = 15.9, $C_{im(2)}CH=C\underline{H}$, 1H), 5.07 (ddd, $^3J_{FH}$ = 18.1, $^2J_{HH}$ = 15.0, $^3J_{PH}$ = 2.6, $C\underline{H}_2C(F)P$, 1H), 4.79 (m, $C\underline{H}_2C(F)P$, 1H), 4.37 (q, $^3J_{HH}$ = 7.2, $CO_2C\underline{H}_2$, 2H), 1.38 (t, $^3J_{HH}$ = 7.2, $CO_2CH_2C\underline{H}_3$, 3H). **^{13}C NMR** (176 MHz, D_2O pH 7) δ 171.9 (d, $^2J_{FC}$ = 20.8, $\underline{C}O_2H$, 1C), 167.4 (s, $\underline{C}O_2Et$, 1C), 141.9 (s, $\underline{C}_{im(2)}$, 1C), 125.7 (s, $\underline{C}HCO_2Et$, 1C), 125.3 (s, $\underline{C}HCHCO_2Et$, 1C), 125.0 (s, $\underline{C}H_{Im(5)}$, 1C), 123.9 (s, $\underline{C}H_{Im(4)}$, 1C), 96.6 (dd, $^1J_{FC}$ = 197.2, $^1J_{PC}$ = 141.4, $\underline{C}(F)P$, 1C), 62.5 (s, $CO_2\underline{C}H_2$, 1C), 50.5 (dd, $^2J_{FC}$ = 19.2, $^2J_{PC}$ = 8.6, $\underline{C}H_2C(F)P$, 1C), 13.3 (s, $CO_2CH_2\underline{C}H_3$, 1C). **^{31}P NMR** (283 MHz, D_2O pH 7) δ 7.55 (d, $^2J_{PF}$ = 73.9).

Sodium (*E*)-3-(2-(5-ethoxy-5-oxopent-3-en-1-yl)-1*H*-imidazol-1-yl)-2-fluoro-2-phosphonopropanoate (5b): obtained according to the general procedure of ester deprotection. Reaction with BTMS carried out in presence of TEA (10 eq). Scale: 70 mg of **28b**. Product **5b** was

obtained as sodium salt by crystallization from mixture of H_2O (pH 7 adjusted by 1M NaOH) and EtOH. Yield: 63% (40 mg). **^{1}H NMR** (700 MHz, D_2O pH 7) δ 7.39 (dd, $^{3}J_{HH}$ = 2.1, J = 1.0, $C\underline{H}_{im}$, 1H), 7.36 (d, $^{3}J_{HH}$ = 2.1, $C\underline{H}_{im}$, 1H), 7.05 (dt, $^{3}J_{HH}$ = 15.7, 6.9, $C\underline{H}$=$CHCO_2$, 1H), 6.00 (dt, $^{3}J_{HH}$ = 15.7, J = 1.5, CH=$C\underline{H}CO_2$, 1H), 4.98 (ddd, $^{3}J_{FH}$ = 32.8, $^{2}J_{HH}$ = 15.2, $^{3}J_{PH}$ = 2.7, $C\underline{H_2}C(F)P$, 1H), 4.68 (ddd, $^{2}J_{HH}$ = 14.9, $^{3}J_{FH}$ = 9.5, $^{3}J_{PH}$ = 4.7, $C\underline{H_2}C(F)P$, 1H), 4.25 (q, $^{3}J_{HH}$ = 7.2, $CO_2C\underline{H_2}$, 2H), 3.35 – 3.23 (m, $C_{im(2)}C\underline{H_2}$, 2H), 2.80 – 2.76 (m, $C_{im(2)}CH_2C\underline{H_2}$, 2H), 1.31 (t, $^{3}J_{HH}$ = 7.2, $CO_2CH_2C\underline{H_3}$, 3H). **^{13}C NMR** (176 MHz, D_2O pH 7) δ 172.1 (d, $^{2}J_{FC}$ = 20.7, $\underline{C}O_2H$, 1C), 168.6 (s, $\underline{C}O_2Et$, 1C), 147.1 (s, $\underline{C_{im(2)}}$, 1C), 146.6 (s, $C\underline{H}$=$CHCO_2$, 1C), 122.7 (s, $\underline{C}H_{im}$, 1C), 122.6 (s, CH=$C\underline{H}CO_2$, 1C), 118.4 (s, $\underline{C}H_{im}$, 1C), 96.8 (dd, $^{1}J_{FC}$ = 196.3, $^{1}J_{PC}$ = 142.3, $\underline{C}$(F)P, 1C), 61.7 (s, $CO_2\underline{C}H_2$, 1C), 50.7 (dd, $^{2}J_{FC}$ = 19.5, $^{2}J_{PC}$ = 7.8, $\underline{C}H_2C(F)P$, 1C), 28.6 (s, $C_{im(2)}CH_2\underline{C}H_2$, 1C), 22.9 (s, $C_{im(2)}\underline{C}H_2$, 1C), 13.3 (s, $CO_2CH_2\underline{C}H_3$, 1C). **^{31}P NMR** (283 MHz, D_2O, pH 7) δ 7.38 (d, $^{2}J_{PF}$ = 71.7). Elemental analysis: $C_{13}H_{15}FN_2Na_3O_7P$*$0.67C_2H_6O$*$1.8H_2O$: calculated C34.90; H4.62; N5.68; found C34.69; H4.35; N5.80; max. diff. 0.27.

(*E*)-2-fluoro-3-(2-(3-(methylamino)-3-oxoprop-1-en-1-yl)-1*H*-imidazol-1-yl)-2-phosphonopropanoic acid (5c): obtained according to the general procedure of ester deprotection. Scale: 74 mg of **28c**. Product **5c** was purified by crystallization from EtOH. Yield: 74% (22 mg).. **^{1}H NMR** (700 MHz, D_2O pH 7) δ 7.61 (bs, $C\underline{H}_{im}$, 1H), 7.58 (d, $^{4}J_{HH}$ = 2.0, $C\underline{H}_{im}$, 1H), 7.54 (dd, $^{3}J_{HH}$ = 15.9, $^{3}J_{HH}$ = 1.1 $C\underline{H}$=, 1H), 7.02 (d, $^{3}J_{HH}$ = 16.0, $C\underline{H}$=, 1H), 5.14 (ddd, $^{3}J_{FH}$ = 31.4, $^{2}J_{HH}$ = 15.6, $^{3}J_{PH}$ = 3.8, $C\underline{H_2}C(F)P$, 1H), 4.90 (ddd, $^{2}J_{HH}$ = 15.6, $^{3}J_{FH}$ = 11.4, $^{3}J_{PH}$ = 4.4, $C\underline{H_2}C(F)P$, 1H), 2.91 (s, NH$\underline{Me}$, 3H). **^{13}C NMR** (176 MHz, D_2O pH 7) δ 170.4 (d, $^{2}J_{FC}$ = 22.5, $\underline{C}O_2$, 1C), 166.1 (s, $\underline{C}$ONH, 1C), 141.5 (s, $\underline{C}_{im(2)}$, 1C), 131.6 (s, CH=$\underline{C}$H, 1C), 124.7 (d, J = 2.0, $\underline{C}H_{im}$, 1C), 120.4 (s, $\underline{C}H_{im}$, 1C), 119.1 (d, J = 3.1, CH=$\underline{C}$H, 1C), 95.7 (dd, $^{1}J_{FC}$ = 196.7, $^{1}J_{PC}$ = 141.4, $\underline{C}$(F)P, 1C), 50.6 (dd, $^{2}J_{FC}$ = 19.5, $^{2}J_{PC}$ = 7.3, $\underline{C}H_2C(F)P$, 1C), 26.3 (s, $NH\underline{C}H_3$, 1C). **^{31}P NMR** (283 MHz, D_2O, pH 7) δ 5.83 (d, $^{2}J_{PF}$ = 71.3).

Sodium 3-(2-(3-ethoxy-3-oxopropyl)-1H-imidazol-1-yl)-2-fluoro-2-phosphonopropanoate (5d): obtained according to the general procedure of ester deprotection. Scale: 63 mg of **28d**. Product **5d** was obtained as sodium salt by crystallization from mixture of H_2O (pH 7 adjusted by 1M NaOH) and acetone. Yield: 71% (40 mg). **^{1}H NMR** (700 MHz, D_2O pH 7) δ 7.40 – 7.38 (m, $C\underline{H_{im}}$, 2H), 5.01 (ddd, $^{3}J_{FH}$ = 32.6, $^{2}J_{HH}$ = 15.3, $^{3}J_{PH}$ = 2.9, $C\underline{H_2}C(F)P$, 1H), 4.73 (ddd, $^{2}J_{HH}$ = 14.9, $^{3}J_{FH}$ = 9.9, $^{3}J_{PH}$ = 4.7, $C\underline{H_2}C(F)P$, 1H), 4.20 (q, $^{3}J_{HH}$ = 7.2, $CO_2C\underline{H_2}$, 2H), 3.39 (t, $^{3}J_{HH}$ = 7.2, $\underline{C}H_2CH_2CO_2Et$, 2H), 2.98 (t, $^{3}J_{HH}$ = 7.0, $\underline{C}H_2CO_2Et$, 2H), 1.26 (t, $^{3}J_{HH}$ = 7.2, $CO_2CH_2C\underline{H_3}$, 3H). **^{13}C NMR** (176 MHz, D_2O pH 7) δ 173.5 (s, $\underline{C}O_2Et$, 1C), 171.8 (d, $^{2}J_{FC}$ = 21.0, $\underline{C}O_2H$, 1C), 145.0 (s, $\underline{C_{im}}$, 1C), 122.7 (s, $\underline{C}H_{Im(5)}$, 1C), 118.4 (s, $\underline{C}H_{Im(4)}$, 1C), 96.6 (dd, $^{1}J_{FC}$ = 196.5, $^{1}J_{PC}$ = 144.2, $\underline{C}$(F)P, 1C), 62.2 (s, $CO_2\underline{C}H_2$, 1C), 50.7 (dd, $^{2}J_{FC}$ = 19.5, $^{2}J_{PC}$ = 8.6, $\underline{C}H_2C(F)P$, 1C), 30.9 (s, $\underline{C}H_2CO_2$, 1C), 19.7 (s, $C_{im(2)}\underline{C}H_2$, 1C), 13.2 (s, $CO_2CH_2\underline{C}H_3$, 1C). **^{31}P NMR** (283 MHz, D_2O, pH 7) δ 7.36 (d, $^{2}J_{PF}$ = 72.6). Elemental analysis: $C_{11}H_{13}FN_2Na_3O_7P$*$0.15C_2H_6O$ *$1.8H_2O$: calculated C30.60; H3.98; N6.32; found C30.48; H3.85; N6.31; max. diff. 0.13.

Sodium 3-(2-(5-ethoxy-5-oxopentyl)-1*H*-imidazol-1-yl)-2-fluoro-2-phosphonopropanoate (5e): obtained according to the general procedure of ester deprotection. Scale: 49 mg of **28e**. Product **5e** was as sodium salt by crystallization from mixture of H_2O (pH 7 adjusted by 1M NaOH) and EtOH. Yield: 88% (39 mg). **^{1}H NMR** (700 MHz, D_2O pH 7) δ 7.37 – 7.36 (m, $C\underline{H}_{im}$, 1H), 7.34 (d, $^{3}J_{HH}$ = 2.1, $C\underline{H}_{im}$, 1H), 4.97 (ddd, $^{3}J_{FH}$ = 33.4, $^{2}J_{HH}$ = 15.1, $^{3}J_{PH}$ = 1.8, $C\underline{H_2}C(F)P$, 1H), 4.64 (ddd, $^{2}J_{HH}$ = 14.9, $^{3}J_{FH}$ = 8.9, $^{3}J_{PH}$ = 4.6, $C\underline{H_2}C(F)P$, 1H), 4.20 (q, $^{3}J_{HH}$ = 7.2, $CO_2C\underline{H_2}$, 2H), 3.15 – 3.06 (m, $C_{im(2)}C\underline{H_2}$, 2H), 2.48 (t, $^{3}J_{HH}$ = 7.4, $C\underline{H_2}CO_2$, 2H), 1.88 – 1.80 (m, $C_{im(2)}CH_2C\underline{H_2}$, 2H), 1.76 – 1.71 (m, $C\underline{H_2}CH_2CO_2$, 2H), 1.28 (t, $^{3}J_{HH}$ = 7.2, $CO_2CH_2C\underline{H_3}$, 3H). **^{13}C NMR** (176 MHz, D_2O pH 7) δ 176.5 (s, $\underline{C}O_2Et$, 1C), 172.8 (d, $^{2}J_{FC}$ = 20.9, $\underline{C}O_2H$, 1C), 148.3 (s, $\underline{C_{im(2)}}$, 1C), 122.5 (d, ^{4}J = 2.3, $\underline{C}H_{im}$, 1C), 118.1 (s, $\underline{C}H_{im}$, 1C), 97.3 (dd, $^{1}J_{FC}$ = 195.9, $^{1}J_{PC}$ = 139.3, $\underline{C}$(F)P, 1C), 61.7 (s, $CO_2\underline{C}H_2$, 1C), 50.9 (dd, $^{2}J_{FC}$ = 19.7, $^{2}J_{PC}$ = 8.3,

$\underline{C}H_2C(F)P$, 1C), 33.4 (s, $CH_2\underline{C}H_2CO_2$, 1C), 25.5 (s, $C_{im(2)}CH_2\underline{C}H_2$, 1C), 24.0 (s, $C_{im(2)}\underline{C}H_2$, 1C), 23.6 (s, $CH_2\underline{C}H_2CO_2$, 1C), 13.3 (s, $CO_2CH_2\underline{C}H_3$, 1C). **^{31}P NMR** (283 MHz, D_2O pH 7) δ 7.43 (d, $^2J_{PF}$ = 69.6).

2-fluoro-3-(2-(3-(methylamino)-3-oxopropyl)-1*H*-imidazol-1-yl)-2-phosphonopropanoic acid (2f): obtained according to the general procedure of ester deprotection. Scale: 70 mg of **28f**. Product **4f** was initially purified by crystallization from EtOH. Futher purification was carried out by preparative HPLC (eluent A: H_2O:ACN 99:5, isocratic, retention time 3.7 min.) followed by lyophilization. Yield: 64% (28 mg). **^{1}H NMR** (700 MHz, D_2O pH 7) δ 7.45 – 7.40 (m, $C\underline{H}_{im}C\underline{H}_{im}$, 2H), 5.03 (ddd, $^3J_{FH}$ = 31.9, $^2J_{HH}$ = 15.5, $^3J_{PH}$ = 3.8, $C\underline{H}_2C(F)P$, 1H), 4.85 – 4.80 (m, $C\underline{H}_2C(F)P$, 1H), 3.36 (t, $^3J_{HH}$ = 7.3, $CH_2C\underline{H}_2$, 2H), 2.80 (dt, $^3J_{HH}$ = 7.4, 2.4, $CH_2C\underline{H}_2$, 2H), 2.74 (s, NH$\underline{Me}$, 3H). **^{13}C NMR** (176 MHz, D_2O pH 7) δ 173.3 (s, $\underline{C}$ONH, 1C), 170.3 (d, $^2J_{FC}$ = 23.5, CF$\underline{CO}_2$, 1C), 147.4 (s, $\underline{C}_{im(2)}$, 1C), 122.7 (s, $\underline{C}H_{im}$, 1C), 118.6 (s, $\underline{C}H_{im}$, 1C), 95.6 (dd, $^1J_{FC}$ = 195.9, $^1J_{PC}$ = 140.6, $\underline{C}$(F)P, 1C), 50.5 – 49.5 (m, $\underline{C}H_2C(F)P$, 1C), 32.1 (s, $CH_2\underline{C}H_2$, 1C), 25.9 (s, $NH\underline{C}H_3$, 1C), 20.4 (s, $CH_2\underline{C}H_2$, 1C). **^{31}P NMR** (283 MHz, D_2O, pH 7) δ 5.02 (d, $^2J_{PF}$ = 69.3).

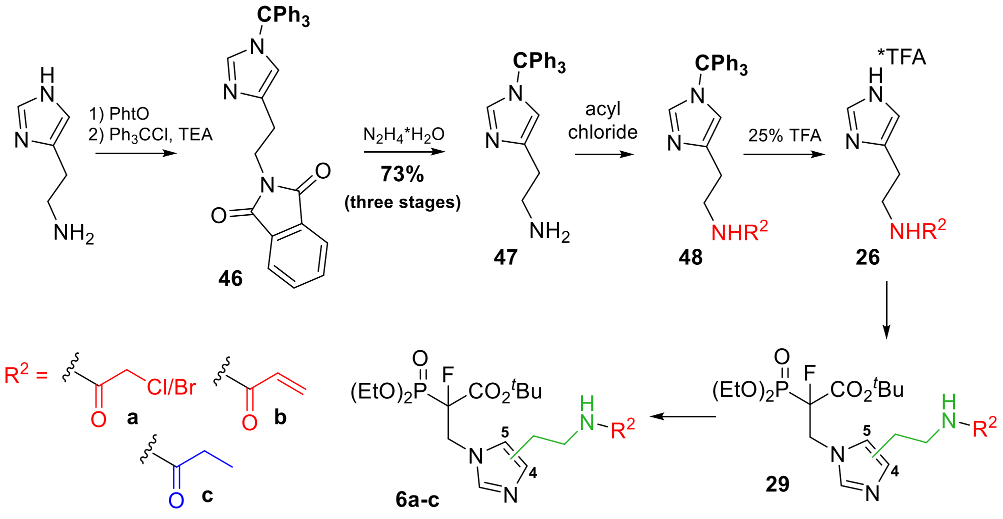

Scheme S14. Synthesis of compounds **6.**

2-(1-trityl-1*H*-imidazol-4-yl)ethan-1-amine (47): required three steps of synthesis. The first step included incorporation of phthalimide. A mixture of 2-(1*H*-imidazol-4-yl)ethan-1-amine dihydrochloride (1.3 g), isobenzofuran-1,3-dione (PhtO, 1.1 eq., 1.1 g) in acetic acid (11 mL) was refluxed for 5 h. After cooling to ambient temperature, the mixture was evaporated under reduced pressure, the residue was diluted with water (50 mL) and neutralized with sodium carbonate (pH 9). The mixture was extracted with $CHCl_3$ (3x100 mL). The combined organic phases were dried over $MgSO_4$ and concentrated. Thus obtained compound was used in the second step, introduction of trityl group. This step was carried out according to the procedure used for compound **38**, with trityl chloride (1.2 eq.). After extraction and column chromatography (eluent: $CHCl_3$), thus obtained compound **46** was subjected to deprotection of amine group. Compound **46** was dissolved in MeOH (25 mL), then N_2H_4*H_2O (15 eq.) was added and the resulting mixture was stirred for 24h at RT. Obtained precipitate was removed by filtration and the filtrate was concentrated under reduced pressure giving pure product **47**. Yield (three steps): 73%. **^{1}H NMR** (700 MHz, $CDCl_3$) δ 7.35 (d, J_{HH} = 1.4, $C\underline{H}_{Im(2)}$, 1H), 7.34 – 7.32 (m, $C\underline{H}_{Ph}$, 9H), 7.15 – 7.11 (m, $C\underline{H}_{Ph}$, 6H), 6.59 (d, J_{HH} = 1.5, $C\underline{H}_{Im(5)}$, 1H), 3.02 (dt, $^3J_{HH}$ = 6.5, 1.5, $C\underline{H}_2NH_2$, 2H), 2.70 (t, $^3J_{HH}$ = 6.5, $C_{im}C\underline{H}_2$, 2H).

2-Chloro-*N*-(2-(1-trityl-1*H*-imidazol-4-yl)ethyl)acetamide (48a): compound **47** (350 mg) was dissolved in DCM (10 mL) and cooled to 0 °C. Then, TEA (3 eq., 3.0 mmol, 413 μL) was added

followed by addition of chloroacetyl chloride (2 eq., 160 μL). After 2h of stirring at 0 °C, the reaction was quenched by addition of H_2O (3 mL). The organic and aqueous phases were separated. The aqueous phase (pH 9) was additionally extracted with $CHCl_3$ (3x10 mL). Combined organic phases were dried over $MgSO_4$ and concentrated. Compound **48a** was purified by flash chromatography using Gilson PLC 2250 purification system. Eluents A: $CHCl_3$ and B: MeOH (gradient 5→10 min. 0→20%B, retention time 10 min.). Yield: 94% (400 mg). **^{1}H NMR** (700 MHz, $CDCl_3$) δ 7.47 (s, N$\underline{H}$, 1H), 7.41 (d, $^4J_{HH}$ = 1.5, C$\underline{H}_{Im(2)}$, 1H), 7.35 – 7.32 (m, C$\underline{H}_{Ph}$, 9H), 7.14 – 7.12 (m, C$\underline{H}_{Ph}$, 6H), 6.61 (dd, $^4J_{HH}$ = 1.4, 0.8, C$\underline{H}_{Im(5)}$, 1H), 4.00 (s, C$\underline{H}_2$Cl, 2H), 3.63 – 3.57 (m, C$\underline{H}_2$NH, 2H), 2.76 (t, $^3J_{HH}$ = 6.2, C_{im}C$\underline{H}_2$, 2H).

***N*-(2-(1-trityl-1*H*-imidazol-4-yl)ethyl)acrylamide (48b):** obtained according to the procedure for compound **48a**, using acrylic chloride. Scale: 335 mg (0.95 mmol) of **47**. Compound **48b** was purified by flash chromatography using Gilson PLC 2250 purification system. Eluent A: $CHCl_3$ (retention time 22 min.). Yield: 47% (180 mg). **^{1}H NMR** (250 MHz, $CDCl_3$) δ 7.37 (d, $^4J_{HH}$ = 1.4, C$\underline{H}_{Im(2)}$, 1H), 7.36 – 7.30 (m, C$\underline{H}_{Ph}$, 9H), 7.15 – 7.09 (m, C$\underline{H}_{Ph}$, 6H), 6.82 (bs, N$\underline{H}$, 1H), 6.60 (d, $^4J_{HH}$ = 1.3, C$\underline{H}_{Im(5)}$, 1H), 6.23 (dd, $^3J_{HH}$ = 17.0, $^2J_{HH}$ = 1.8, CH=C$\underline{H}_2$, 1H), 6.07 (dd, $^2J_{HH}$ = 17.0, $^3J_{HH}$ = 10.0, C$\underline{H}$=CH_2, 1H), 5.59 (dd, $^3J_{HH}$ = 10.0, $^2J_{HH}$ = 1.8, CH=C$\underline{H}_2$, 1H), 3.60 (dt, $^3J_{HH}$ = 5.9, C$\underline{H}_2$NH, 2H), 2.74 (t, $^3J_{HH}$ = 6.3, C_{im}C$\underline{H}_2$, 2H).

***N*-(2-(1-trityl-1*H*-imidazol-4-yl)ethyl)propionamide (48c):** obtained according to the procedure for compound **48a**, using propionyl chloride. Scale: 150 mg (0.42 mmol) of **47**. Compound **48c** was purified by flash chromatography using Gilson PLC 2250 purification system. As eluent A: $CHCl_3$ was used (retention time 27 min.). Yield: 87% (150 mg). **^{1}H NMR** (250 MHz, $CDCl_3$) δ 7.37 (d, $^4J_{HH}$ = 1.4, C$\underline{H}_{Im(2)}$, 1H), 7.36 – 7.31 (m, C$\underline{H}_{Ph}$, 9H), 7.16 – 7.09 (m, C$\underline{H}_{Ph}$, 6H), 6.59 (bs, C$\underline{H}_{Im(5)}$, 1H), 6.52 (bs, N$\underline{H}$, 1H), 3.51 (dt, $^3J_{HH}$ = 6.0, C$\underline{H}_2$NH, 2H), 2.70 (t, $^3J_{HH}$ = 6.3, C_{im}C$\underline{H}_2$, 2H), 2.17 (q, $^3J_{HH}$ = 7.6, C$\underline{H}_2$$CH_3$, 2H), 1.12 (t, $^3J_{HH}$ = 7.6, C$\underline{H}_3$,

***N*-(2-(1*H*-imidazol-4-yl)ethyl)-2-chloroacetamide (26a):** obtained according to the procedure for compound **24b**. Scale: 336 mg of **48a**. Yield: quantitive. **^{1}H NMR** (700 MHz, D_2O pH 2) δ 7.83 (d, $^4J_{HH}$ = 1.2, C$\underline{H}_{Im(2)}$, 1H), 7.06 (d, $^4J_{HH}$ = 1.2, C$\underline{H}_{Im(5)}$, 1H), 4.20 (s, C$\underline{H}_2$Cl, 2H), 3.59 (t, $^3J_{HH}$ = 6.8, $C_{im}CH_2$C$\underline{H}_2$, 2H), 2.93 (t, $^3J_{HH}$ = 6.8, C_{im}C$\underline{H}_2$, 2H).

***N*-(2-(1*H*-imidazol-4-yl)ethyl)acrylamide (26b):** obtained according to the procedure for compound **24b**. Scale: 180 mg of **48b**. Yield: quantitive. **^{1}H NMR** (250 MHz, D_2O pH 2) δ 8.57 (d, $^4J_{HH}$ = 1.5, C$\underline{H}_{Im(2)}$, 1H), 7.24 (d, $^4J_{HH}$ = 1.3, C$\underline{H}_{Im(5)}$, 1H), 6.21 (dd, $^3J_{HH}$ = 17.1, $^3J_{HH}$ = 9.1, C$\underline{H}$=CH_2, 1H), 6.11 (dd, $^3J_{HH}$ = 17.1, $^2J_{HH}$ = 2.5, CH=C$\underline{H}_2$, 1H), 5.73 (dd, $^3J_{HH}$ = 9.1, $^2J_{HH}$ = 2.5, CH=C$\underline{H}_2$, 1H), 3.56 (dt, $^3J_{HH}$ = 6.6, C$\underline{H}_2$NH, 2H), 2.96 (t, $^3J_{HH}$ = 6.5, C_{im}C$\underline{H}_2$, 2H).

***N*-(2-(1*H*-imidazol-4-ylo)ethyl)propionamide (26c):** obtained according to the procedure for compound **24b**. Scale: 150 mg of **48c**. Yield: quantitive.

***Tert*-butyl 3-(4/5-(2-(2-chloroacetamido)ethyl)-1*H*-imidazol-1-yl)-2-(diethoxyphosphoryl)-2-fluoropropanoate (29a):** obtained according to the *general procedure of Michael addition and fluorination.* Scale: 0.75 mmol of **26a**. In fluorination step 2.0 eq. of NaH was used. Compound **29a** was purified by flash chromatography using Gilson PLC 2250 purification system. Eluents A: $CHCl_3$, B: Acetone (gradient 5→10 min. 0→20% B, retention time 33 min.). Yield: 24% (84 mg). According to ^{1}H NMR spectrum of the main fraction, the mixture of regioisomer C4 and C5 was obtained in ratio 90:10. Spectroscopic data of regioisomer C4: **^{1}H NMR** (250 MHz, $CDCl_3$) δ 7.47 (bs, NH, 1H), 7.35 (s, C$\underline{H}_{Im(2)}$, 1H), 6.70 (s, C$\underline{H}_{Im(5)}$, 1H), 4.59 (ddd, $^3J_{FH}$ = 32.2, $^2J_{HH}$ = 15.3, $^3J_{PH}$ = 5.5, C$\underline{H}_2$C(F)P, 1H), 4.40 (ddd, $^2J_{HH}$ = 15.1, $^3J_{FH}$ = 15.1, $^3J_{PH}$ = 3.8, C$\underline{H}_2$C(F)P, 1H), 4.27 – 4.09 (m, C$\underline{H}_2$OP, 4H), 3.94 (s,

$OCC\underline{H}_2Cl$, 2H), 3.48 (dd, $^3J_{HH}$ = 6.3, 5.9, $C_{im}CH_2C\underline{H}_2NH$, 2H), 2.68 (t, $^3J_{HH}$ = 6.4, $C_{im}C\underline{H}_2CH_2NH$, 2H), 1.34 (s, $C(C\underline{H}_3)_3$, 9H), 1.29 (dd, J_{HH} = 7.1, $^4J_{PH}$ = 0.7, $C\underline{H}_3CH_2OP$, 6H). **^{13}C NMR** (63 MHz, $CDCl_3$) δ 166.0 (s, $CH_2\underline{C}ONH$, 1C), 163.6 (d, $^2J_{FC}$ = 22.2, $\underline{C}O_2{}^tBu$, 1C), 139.9 (s, $\underline{C_{im}}$, 1C), 138.0 (s, $\underline{C}H_{Im(2)}$, 1C), 117.2 (s, $\underline{C}H_{Im(5)}$, 1C), 94.3 (dd, $^1J_{FC}$ = 202.9, $^1J_{PC}$ = 158.2, $\underline{C}(F)P$, 1C), 85.2 (s, $CO_2\underline{C}Me_3$, 1C), 64.9 (d, $^2J_{PC}$ = 6.7, $\underline{C}H_2OP$, 1C), 64.7 (d, $^2J_{PC}$ = 7.1, $\underline{C}H_2OP$, 1C), 49.2 (dd, $^2J_{FC}$ = 19.2, $^2J_{PC}$ = 5.9, $FC\underline{C}H_2$, 1C), 42.6 (s, $OC\underline{C}H_2Cl$, 1C), 39.5 (s, $\underline{C}H_2NH$, 1C), 27.7 (s, $C(\underline{C}H_3)_3$, 3C), 27.1 (s, $C_{im(4)}\underline{C}H_2$, 1C), 16.4 (d, $^3J_{PC}$ = 5.7, $\underline{C}H_3CH_2OP$, 2C). **^{31}P NMR** (101 MHz, $CDCl_3$) δ 10.61 (d, $^2J_{PF}$ = 80.0).

***Tert*-butyl 3-(4/5-(2-acrylamidoethyl)-1*H*-imidazol-1-yl)-2-(diethoxyphosphoryl)-2-fluoropropanoate (29b):** obtained according to the general procedure of Michael addition and fluorination. Scale: 0.50 mmol of **26b**. In fluorination step 2.4 eq. of NaH was used. Compound **29b** was purified by flash chromatography using Gilson PLC 2250 purification system. Eluents A: $CHCl_3$, B: Acetone and C:MeOH (10→20 min. 0→20%B, 25→40 min. 1→1%C, retention time 27 min.). Yield: 48% (107 mg). According to ^{1}H NMR spectrum of the main fraction, the mixture of regioisomer C4 and C5 was obtained in ratio 90:10. Spectroscopic data of regioisomer C4: **^{1}H NMR** (700 MHz, $CDCl_3$) δ 7.34 (s, $C\underline{H}_{Im(2)}$, 1H), 6.88 (s, NH, 1H), 6.69 (s, $C\underline{H}_{Im(5)}$, 1H), 6.16 (dd, $^3J_{HH}$ = 17.0, $^2J_{HH}$ = 1.6, $OCCH{=}C\underline{H}_2$, 1H), 6.06 (dd, $^3J_{HH}$ = 17.1, 10.3, $OCC\underline{H}{=}CH_2$, 1H), 5.50 (dd, $^3J_{HH}$ = 10.3, $^2J_{HH}$ = 1.6, $OCCH{=}C\underline{H}_2$, 1H), 4.57 (ddd, $^3J_{FH}$ = 32.4, $^2J_{HH}$ = 15.3, $^3J_{PH}$ =5.9, $C\underline{H}_2C(F)P$, 1H), 4.41 (ddd, $^2J_{HH}$ = 15.2, $^3J_{FH}$ = 15.2, $^3J_{PH}$ = 3.6, $C\underline{H}_2C(F)P$, 1H), 4.23 – 4.13 (m, $C\underline{H}_2OP$, 4H), 3.54 – 3.44 (m, $C_{im}CH_2C\underline{H}_2NH$, 2H), 2.67 (t, $^3J_{HH}$ = 6.5, $C_{im}C\underline{H}_2CH_2NH$, 2H), 1.34 (s, $C(C\underline{H}_3)_3$, 9H), 1.30 (t, $^3J_{HH}$ = 7.0, $C\underline{H}_3CH_2OP$, 6H). **^{13}C NMR** (176 MHz, $CDCl_3$) δ 165.6 (s, $\underline{C}ONH$, 1C), 163.6 (dd, $^2J_{FC}$ = 22.2, $^2J_{PC}$ = 2.5, $\underline{C}O_2tBu$, 1C), 140.3 (s, $\underline{C_{im(4)}}$, 1C), 137.9 (s, $\underline{C}H_{Im(2)}$, 1C), 131.4 (s, $OC\underline{C}H{=}CH_2$, 1C), 125.6 (s, $OCCH{=}\underline{C}H_2$, 1C), 117.2 (s, $\underline{C}H_{Im(5)}$, 1C), 94.3 (dd, $^1J_{FC}$ = 202.7, $^1J_{PC}$ = 158.5, $\underline{C}(F)P$, 1C), 85.2 (s, $CO_2\underline{C}Me_3$, 1C), 64.9 (dd, J = 6.2, 5.2, $\underline{C}H_2OP$, 1C), 64.7 (dd, J = 6.9, 5.1, $\underline{C}H_2OP$, 1C), 49.3 (dd, $^2J_{FC}$ = 20.2, $^2J_{PC}$ = 5.8, $FC\underline{CH}_2$, 1C), 39.3 (s, $\underline{C}H_2NH$, 1C), 27.7 (s, $C(\underline{C}H_3)_3$, 3C), 27.3 (s, $C_{im(4)}\underline{C}H_2$, 1C), 16.4 (d, $^3J_{PC}$ = 5.7, $\underline{C}H_3CH_2OP$, 2C). **^{31}P NMR** (283 MHz, $CDCl_3$) δ 10.61 (d, $^2J_{PF}$ = 80.1).

Tert*-butyl 2-(diethoxyphosphoryl)-2-fluoro-3-(4/5-(2-propionamidoethyl)-1*H*-imidazol-1-yl)propanoate (29c):** obtained according to the general procedure of Michael addition and fluorination. Scale: 0.37 mmol of **26c**. In fluorination step 2.4 eq. of NaH was used. Compound **29c** was purified by flash chromatography using Gilson PLC 2250 purification system. Eluents A: $CHCl_3$, B: Acetone and C:MeOH (gradient 10→20 min. 0→20% B, 40→55 min. 1→1%C, retention time 37 min.). Yield: 65% (107 mg). According to ^{1}H NMR spectrum of the main fraction, the mixture of regioisomer C4 and C5 was obtained in ratio 70:30. Spectroscopic data of regioisomers ***C4 and ***C5***: **^{1}H NMR** (700 MHz, $CDCl_3$) δ [7.42(***5***) (s, $C\underline{H}_{Im(2)}$, 1H), 7.33(***4***) (s, $C\underline{H}_{Im(2)}$, 1H)], [6.72(***5***) (s, $C\underline{H}_{Im(4)}$, 1H), 6.68(***4***) (s, $C\underline{H}_{Im(5)}$, 1H)], [6.55(***5***) (s, NH, 1H), 6.23(***4***) (s, NH, 1H)], [4.57(***4***) (ddd, $^3J_{FH}$ = 32.6, $^2J_{HH}$ = 15.3, $^3J_{PH}$ = 5.7, $C\underline{H}_2C(F)P$, 1H), 4.66-4.52(***5***) (m, $C\underline{H}_2C(F)P$, 1H)], [4.32-4.37(***5***) (m, $C\underline{H}_2C(F)P$, 1H), 4.40 (ddd, $^2J_{HH}$ = 15.0, $^3J_{FH}$ = 15.0, $^3J_{PH}$ = 3.6, $C\underline{H}_2C(F)P$, 2H)], 4.22 – 4.13 (m, $C\underline{H}_2OP$, 4H), 3.45 – 3.35 (m, $C\underline{H}_2NH$, 2H), [2.74(***5***) (t, $^3J_{HH}$ = 6.6, $C_{im(5)}C\underline{H}_2$, 2H), 2.63(***4***) (t, $^3J_{HH}$ = 6.4, $C_{im(4)}C\underline{H}_2$, 2H)], [2.11(***4***) (q, $^3J_{HH}$ = 7.6, $OCC\underline{H}_2CH_3$, 2H), 2.09(***5***) (q, $^3J_{HH}$ = 7.6, $OCC\underline{H}_2CH_3$, 2H)], [1.348 (s, $C(C\underline{H}_3)_3$, 9H), 1.345 (s, $C(C\underline{H}_3)_3$, 9H)], [1.31(***5***) (t, $^3J_{HH}$ = 7.0, $C\underline{H}_3CH_2OP$, 6H), 1.30(***4***) (t, $^3J_{HH}$ = 7.0, $C\underline{H}_3CH_2OP$, 6H)], [1.05(***4***) (t, $^3J_{HH}$ = 7.6, $OCCH_2C\underline{H}_3$, 3H), 1.04(***5***) (t, $^3J_{HH}$ = 7.6, $OCCH_2C\underline{H}_3$, 3H)]. **^{13}C NMR** (176 MHz, $CDCl_3$) δ [174.1(***5***) (s, $\underline{C}ONH$, 1C), 173.9(***4***) (s, $\underline{C}ONH$, 1C)], [163.9(***5***) (dd, $^2J_{FC}$ = 22.0, $^2J_{PC}$ = 1.6, $\underline{C}O_2tBu$, 1C), 163.6(***4***) (dd, $^2J_{FC}$ = 22.3, $^2J_{PC}$ = 2.2, $\underline{C}O_2tBu$, 1C)], [140.4(***4***) (s, $\underline{C_{im(4)}}$, 1C), 129.8(***5***) (s, $\underline{C_{im(5)}}$, 1C)], [138.2(***5***) (s, $\underline{C}H_{Im(2)}$, 1C), 137.8(***4***) (s, $\underline{C}H_{Im(2)}$, 1C)], [126.5(***5***) (s, $\underline{C}H_{Im(4)}$, 1C), 117.1(***4***) (s, $\underline{C}H_{Im(5)}$, 1C)], [94.8(***5***) (dd, $^1J_{FC}$ = 204.3, $^1J_{PC}$ = 158.6, $\underline{C}(F)P$, 1C), 94.3(***4***) (dd, $^1J_{FC}$ = 202.8, $^1J_{PC}$ = 158.3, $\underline{C}(F)P$, 1C)], [85.4(***5***) (s, $CO_2\underline{C}Me_3$, 1C),

85.2(***4***) (s, $CO_2\underline{C}Me_3$, 1C)], 65.1 – 64.6 (m, $\underline{C}H_2OP$, 1C), [49.2(***4***) (dd, $^2J_{FC}$ = 20.0, $^2J_{PC}$ = 5.9, $FCC\underline{H}_2$, 1C), 46.5(***5***) (dd, $^2J_{FC}$ = 20.1, $^2J_{PC}$ = 6.0, $FCC\underline{H}_2$, 1C)], [39.2(***4***) (s, $\underline{C}H_2NH$, 1C), 38.0(***5***) (s, $\underline{C}H_2NH$, 1C)], [29.7(***4***) (s, $OC\underline{C}H_2CH_3$, 1C), 29.5(***5***) (s, $OC\underline{C}H_2CH_3$, 1C)], [27.7(***4***) (s, $C(\underline{C}H_3)_3$, 3C), 27.7(***5***) (s, $C(\underline{C}H_3)_3$, 3C)], [27.4(***4***) (s, $C_{im(4)}\underline{C}H_2$, 1C), 23.9(***5***) (s, $C_{im(5)}\underline{C}H_2$, 1C)], [16.4(***5***) (d, $^3J_{PC}$ = 5.3, $\underline{C}H_3CH_2OP$, 2C), 16.35(***4***) (d, $^3J_{PC}$ = 5.7, $\underline{C}H_3CH_2OP$, 2C)], 9.9(***4***) (s, $OCCH_2\underline{C}H_3$, 1C), 9.8(***5***) (s, $OCCH_2\underline{C}H_3$, 1C). **^{31}P NMR** (283 MHz, $CDCl_3$) δ 10.63(***4***) (d, $^2J_{PF}$ = 80.3), 10.71(***5***) (d, $^2J_{PF}$ = 80.2).

3-(4-(2-(2-bromoacetamido)ethyl)-1*H*-imidazol-1-yl)-2-fluoro-2-phosphonopropanoic acid (6a): obtained according to the general procedure of ester deprotection. Scale: 40 mg of **29a**. Product **6a** was purified by preparative HPLC (isocratic, A 100%, retention time 5.9 min.) followed by lyophilization. Yield: 53% (18 mg). According to ^{1}H NMR spectrum of main HPLC fraction single regioisomer C4 was obtained with no traces of regioisomer C5. **^{1}H NMR** (700 MHz, D_2O pH 3) δ 8.75 (s, $C\underline{H}_{Im(2)}$, 1H), 7.38 (s, $C\underline{H}_{Im(5)}$, 1H), 5.03 (ddd, $^3J_{FH}$ = 32.4, $^2J_{HH}$ = 15.2, $^3J_{PH}$ = 3.8, $C\underline{H}_2C(F)P$, 1H), 4.84 – 4.80 (m, $C\underline{H}_2C(F)P$, 1H), 3.90 (s, $OCC\underline{H}_2Br$, 2H), 3.56 (t, $^3J_{HH}$ = 6.5, $C\underline{H}_2NH$, 2H), 2.98 (t, $^3J_{HH}$ = 6.5, $C_{im(4)}C\underline{H}_2$, 2H). **^{13}C NMR** (176 MHz, D_2O pH 3) δ 170.4 (d, $^2J_{FC}$ = 22.0, $\underline{C}O_2$, 1C), 170.1 (s, $\underline{C}ONH$, 1C), 135.5 (s, $\underline{C_{im(4)}}$, 1C), 131.3 (s, $\underline{C}H_{Im(2)}$, 1C), 120.4 (s, $\underline{C}H_{Im(5)}$, 1C), 96 (m, $\underline{C}(F)P$, 1C, in HMBC spectrum), 51.9 (dd, $^2J_{FC}$ = 19.7, $^2J_{PC}$ = 6.8, $FC\underline{C}H_2$, 1C), 38.2 (s, $\underline{C}H_2NH$, 1C), 27.9 (s, $\underline{C}H_2Br$, 1C), 23.9 (s, $C_{im(4)}\underline{C}H_2$, 1C). **^{31}P NMR** (283 MHz, D_2O, pH 3) δ 4.23 (d, $^2J_{PF}$ = 67.8).

3-(4-(2-acrylamidoethyl)-1*H*-imidazol-1-yl)-2-fluoro-2-phosphonopropanoic acid (6b): obtained according to the general procedure of ester deprotection. Reaction with BTMS carried out in presence of TEA (10 eq). Scale: 70 mg of **29b**. Product **6b** was initially purified by crystallization from chloroform. Futher purification was carried out by preparative HPLC (isocratic, A 100%, retention time 4.3 min.) followed by lyophilization. Yield: 33% (17 mg). According to ^{1}H NMR spectrum of main HPLC fraction single regioisomer C4 was obtained with no traces of regioisomer C5. **^{1}H NMR** (700 MHz, D_2O pH 7) δ 8.74 (s, $C\underline{H}_{Im(2)}$, 1H), 7.36 (s, $C\underline{H}_{Im(5)}$, 1H), 6.26 (dd, $^3J_{HH}$ = 17.1, 10.2, $NHC(O)C\underline{H}{=}CHH$, 1H), 6.19 (dd, $^3J_{HH}$ = 17.1, $^2J_{HH}$ =1.3, $NHC(O)CH{=}C\underline{H}H$, 1H), 5.79 (dd, $^3J_{HH}$ = 10.2, $^2J_{HH}$ = 1.3, $NHC(O)CH{=}CH\underline{H}$, 1H), 5.03 (ddd, $^3J_{FH}$ = 32.4, $^2J_{HH}$ =15.2, $^3J_{PH}$ = 3.8, $CFC\underline{H}H$, 1H), 4.79 (m, $CFC\underline{H}H$, 1H), 3.60 (t, $^3J_{HH}$ = 6.6, $C\underline{H}_2NH$), 2H), 2.99 (t, $^3J_{HH}$ = 6.6, $C\underline{H}_2CH_2NH$, 2H). **^{13}C NMR** (176 MHz, D_2O) δ 170.4 (d, $^2J_{FC}$ = 22.7, $\underline{C}O_2H$, 1C), 168.7 (s, $\underline{C}ONH$, 1C), 135.5 (s, $\underline{C}H_{Im(2)}$, 1C), 131.6 (s, $\underline{C_{im(4)}}$, 1C), 129.8 (s, $NHC(O)\underline{C}H{=}CH_2$, 1C), 127.6 (s, $NHC(O)CH\underline{C}H_2$, 1C), 120.2 (s, $\underline{C}H_{Im(5)}$, 1C), 95 (m, $\underline{C}(F)P$, 1C, in HMBC spectrum) 51.9 (dd, $^2J_{FC}$ = 20.1, $^2J_{PC}$ = 7.0, $\underline{C}H_2C(F)P$, 1C), 37.9 (s, $\underline{C}H_2NHC(O)$, 1C), 24.1 (s, $\underline{C}H_2CH_2NHC(O)$, 1C). **^{31}P NMR** (283 MHz, D_2O, pH 7) δ 5.26 (d, $^2J_{PF}$ = 64.7).

2-fluoro-2-phosphono-3-(4-(2-propionamidoethyl)-1*H*-imidazol-1-yl)propanoic acid (6c): obtained according to the general procedure of ester deprotection. Scale: 107 mg of **29c** (mixture of regioisomers C4 and C5). Product **6c** was purified by preparative HPLC (isocratic, A 100%, retention time 6.4 min.) followed by lyophilization. Yield: 66% (43 mg). According to ^{1}H NMR spectrum of main HPLC fraction single regioisomer C4 was obtained with no traces of regioisomer C5. **^{1}H NMR** (700 MHz, 700 MHz, D_2O pH 3) δ 8.75 (s, $C\underline{H}_{Im(2)}$, 1H), 7.36 (s, $C\underline{H}_{Im(5)}$, 1H), 5.04 (ddd, $^3J_{FH}$ = 32.4, $^2J_{HH}$ = 15.2, $^3J_{PH}$ = 3.8, $C\underline{H}_2C(F)P$, 1H), 4.84 – 4.79 (m, $C\underline{H}_2C(F)P$, 1H), 3.51 (t, $^3J_{HH}$ = 6.6, $C\underline{H}_2NH$, 2H), 2.95 (t, $^3J_{HH}$ = 6.5, $C_{im}C\underline{H}_2$, 2H), 2.24 (q, $^3J_{HH}$ = 7.7, $OCC\underline{H}_2CH_3$, 2H), 1.09 (t, $^3J_{HH}$ = 7.7, $OCCH_2C\underline{H}_3$, 3H). **^{13}C NMR** (176 MHz, D_2O pH 3) δ 178.2 (s, $\underline{C}ONH$, 1C), 170.3 (d, $^2J_{FC}$ = 23.4, $\underline{C}O_2H$, 1C), 135.5 (s, $\underline{C}H_{Im(2)}$, 1C), 131.7 (s, $\underline{C_{im(4)}}$, 1C), 120.2 (s, $\underline{C}H_{Im(5)}$, 1C), 95.5 (dd, $^1J_{FC}$ = 196.4, $^1J_{PC}$ = 141.8, $\underline{C}(F)P$, 1C), 51.9 (dd, $^2J_{FC}$ = 19.5, $^2J_{PC}$ = 7.0, $FCC\underline{H}_2$, 1C), 37.7 (s, $\underline{C}H_2NH$, 1C), 29.1 (s, $OC\underline{C}H_2CH_3$, 1C), 24.1 (s, $C_{im(4)}\underline{C}H_2$, 1C), 9.5 (s, $OCCH_2\underline{C}H_3$, 1C). **^{31}P NMR** (283 MHz, D_2O, pH 3) 5.05 (d, $^2J_{PF}$ = 69.7). **2-fluoro-2-phosphono-3-(5-(2-propionamidoethyl)-1*H*-imidazol-1-yl)propanoic acid:** the regioisomer C5 was obtained as the minor HPLC fraction (retention time 5.0

min): **^{1}H NMR** (700 MHz, D_2O pH 3) δ 8.75 (s, C<u>H</u>$_{Im(2)}$, 1H), 7.34 (s, <u>C</u>H$_{Im(4)}$, 1H), 5.06 (ddd, $^{3}J_{FH}$ = 31.4, $^{2}J_{HH}$ = 15.6, $^{3}J_{PH}$ = 4.5, C<u>H</u>$_2$C(F)P, 1H), 4.87 – 4.79 (m, C<u>H</u>$_2$C(F)P, 1H), 3.55 (t, $^{3}J_{HH}$ = 6.7, C<u>H</u>$_2$NH, 2H), 3.08-2.98 (m, C$_{im(5)}$C<u>H</u>$_2$, 2H), 2.25 (q, $^{3}J_{HH}$ = 7.7, OCC<u>H</u>$_2$CH$_3$, 2H), 1.08 (t, $^{3}J_{HH}$ = 7.7, OCCH$_2$C<u>H</u>$_3$, 3H). **^{13}C NMR** (176 MHz, D_2O pH 3) δ 178.2 (s, <u>C</u>ONH, 1C), 170.3 (d, $^{2}J_{FC}$ = 21.6, <u>C</u>O$_2$H, 1C), 135.8 (s, <u>C</u>H$_{Im(2)}$, 1C), 133.1 (s, <u>C$_{im(5)}$</u>, 1C), 116.9 (s, <u>C</u>H$_{Im(4)}$, 1C), 96 (<u>C</u>(F)P, 1C), 48.9 (dd, $^{2}J_{FC}$ = 19.4, $^{2}J_{PC}$ = 6.0, FCC<u>H</u>$_2$, 1C), 37.0 (s, <u>C</u>H$_2$NH, 1C), 29.1 (s, OC<u>C</u>H$_2$CH$_3$, 1C), 22.9 (s, C$_{im(5)}$<u>C</u>H$_2$, 1C), 9.5 (s, OCCH$_2$<u>C</u>H$_3$, 1C). **^{31}P NMR** (283 MHz, D_2O, pH 3) δ 5.78 (d, $^{2}J_{PF}$ = 70.9).

NMR spectra of selected compounds

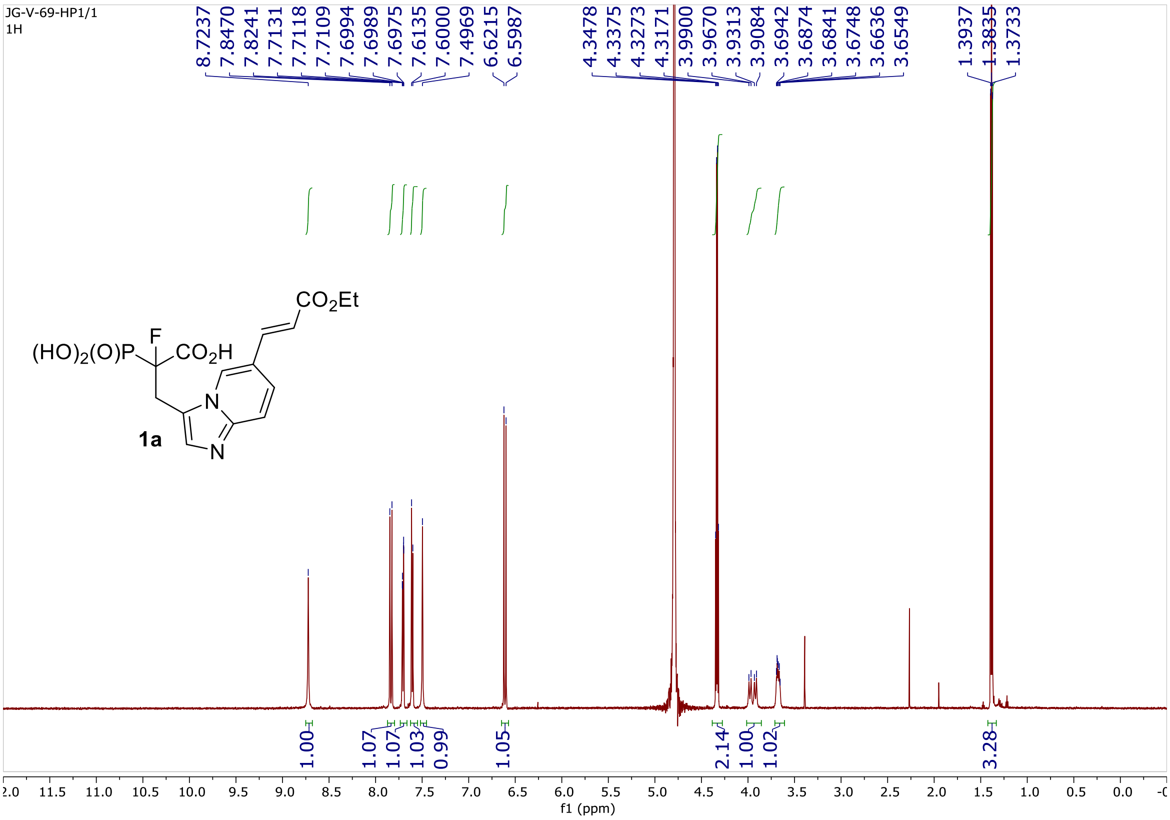

Figure S5. ^{1}H NMR (700 MHz, D_2O, pH 3) of compound **1a**.

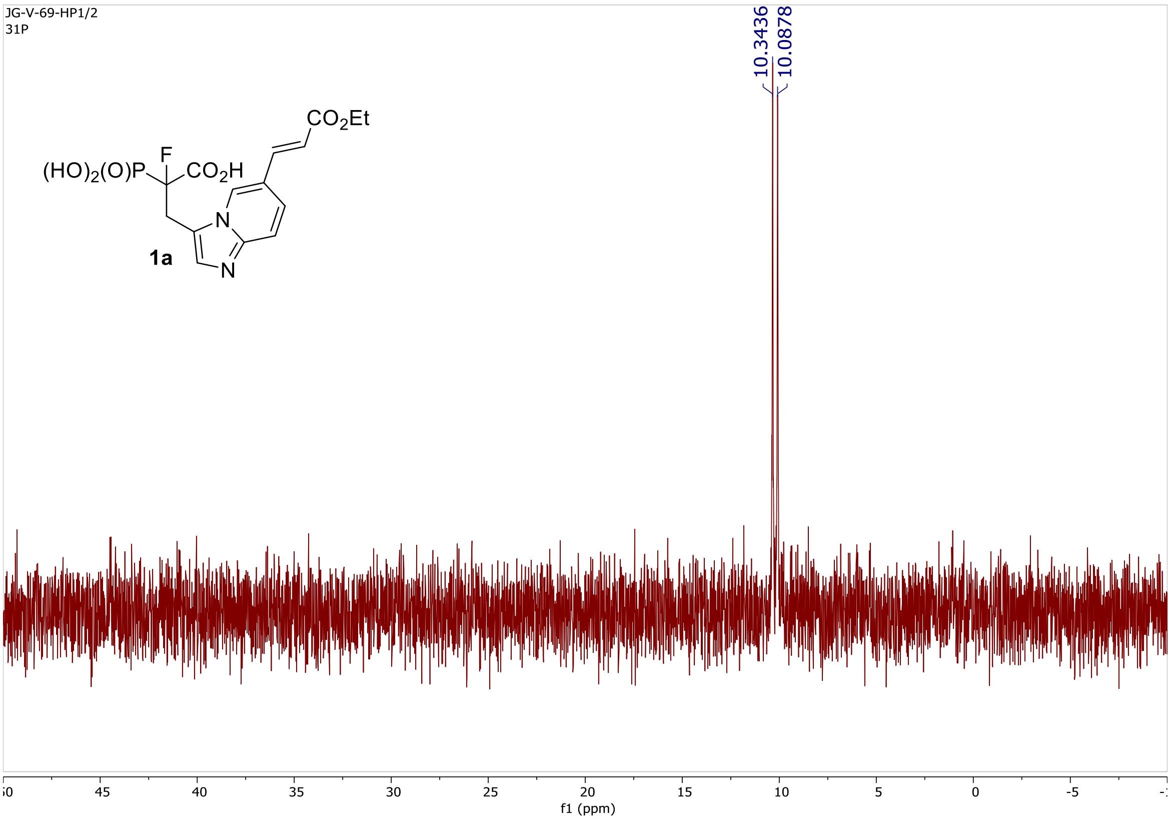

Figure S6. ^{31}P NMR (283 MHz, D_2O, pH 3) of compound **1a**.

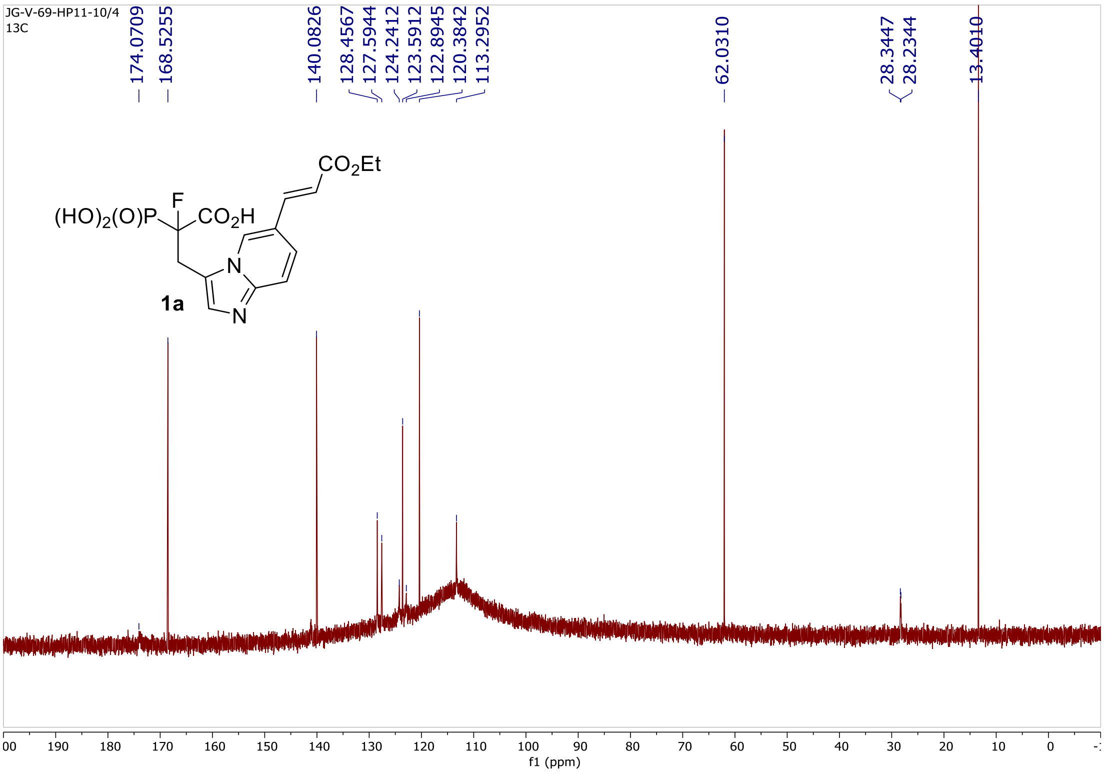

Figure S7. ^{13}C NMR (176 MHz, D_2O, pH 3) of compound **1a.**

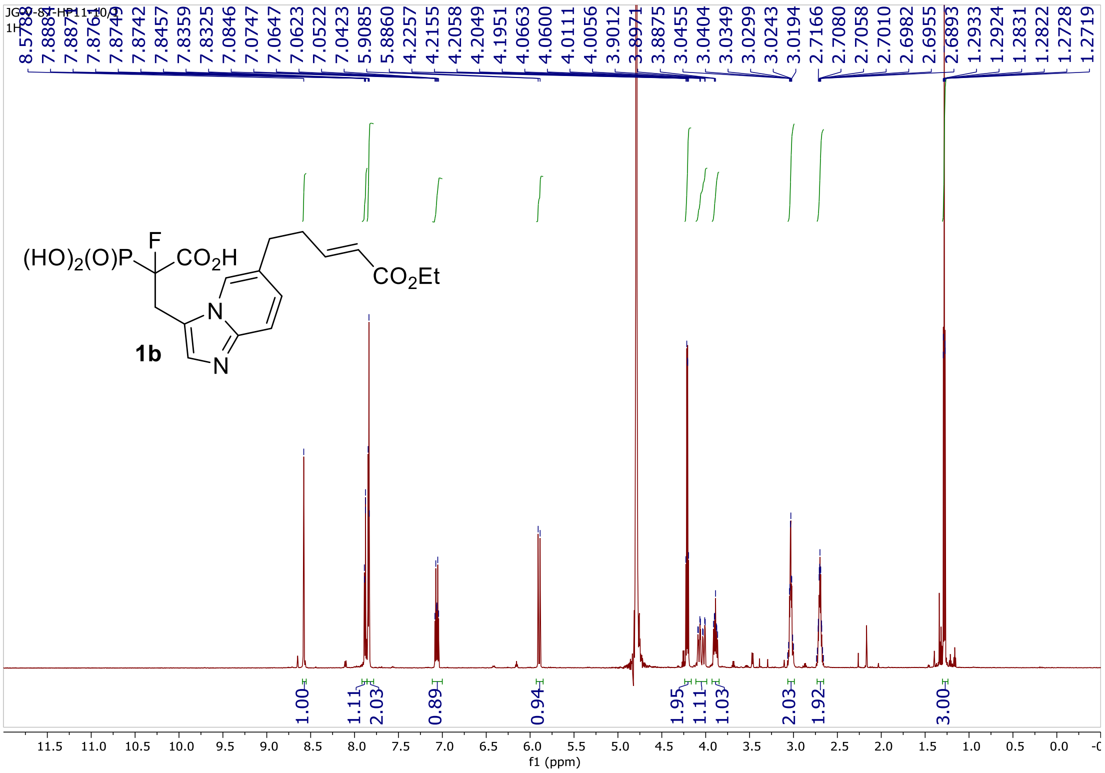

Figure S8. ^{1}H NMR (700 MHz, D_2O, pH 2) of compound **1b**.

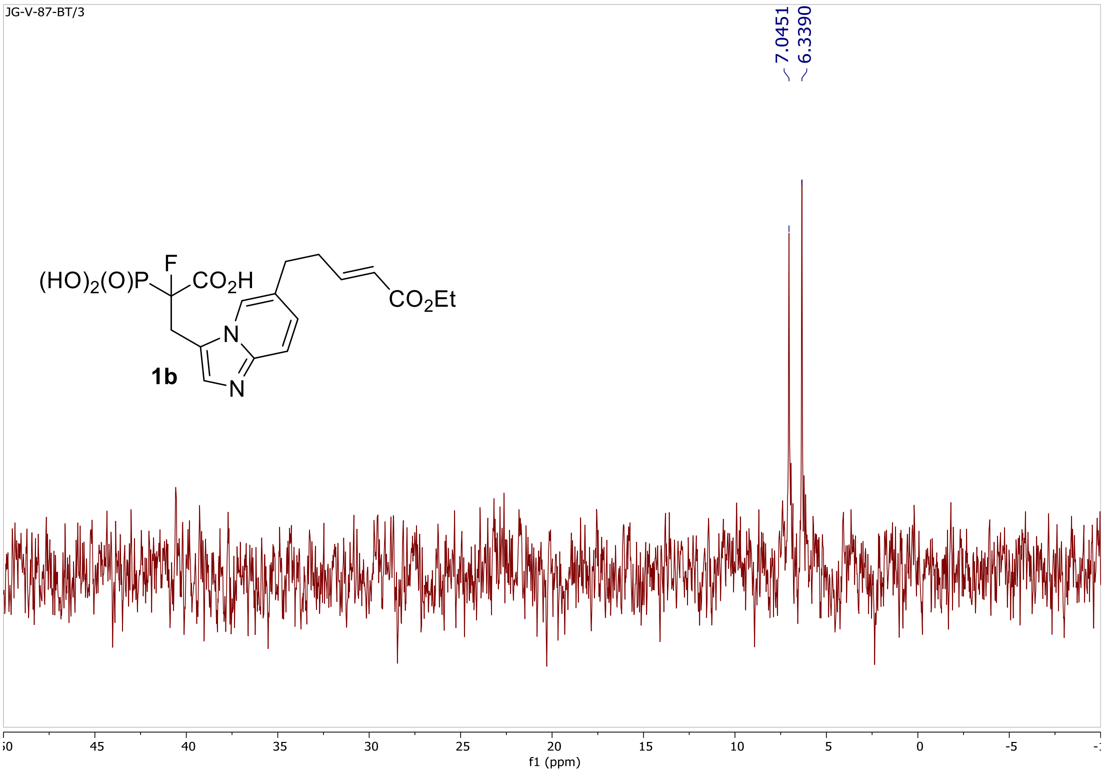

Figure S9. ^{31}P NMR (101 MHz, D_2O, pH 2) of compound **1b**.

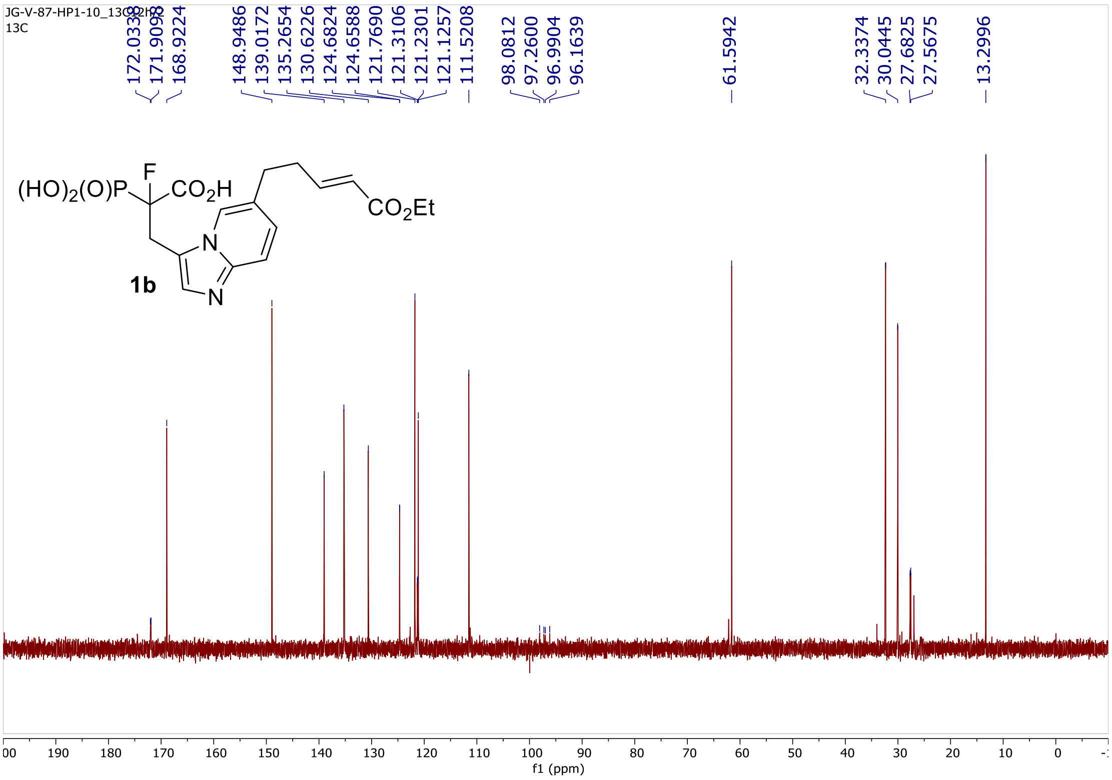

Figure S10. ^{13}C NMR (176 MHz, D_2O, pH 2) of compound **1b**.

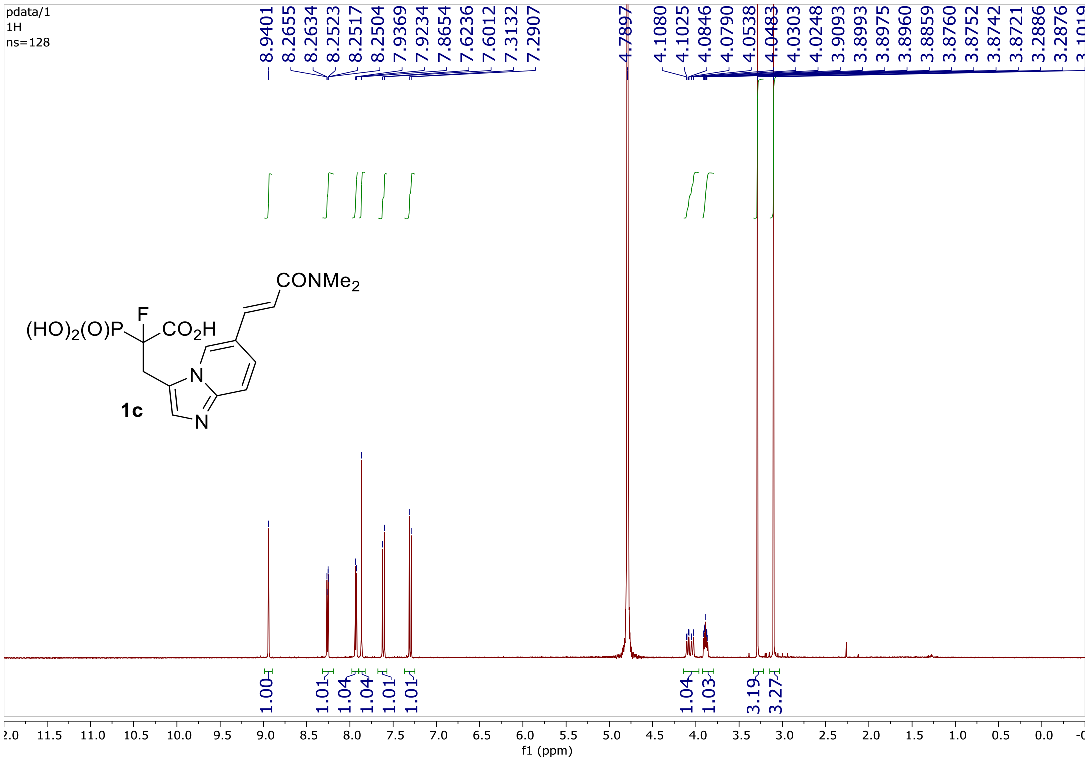

Figure S11. ¹H NMR (700 MHz, D_2O, pH 4) of compound **1c**.

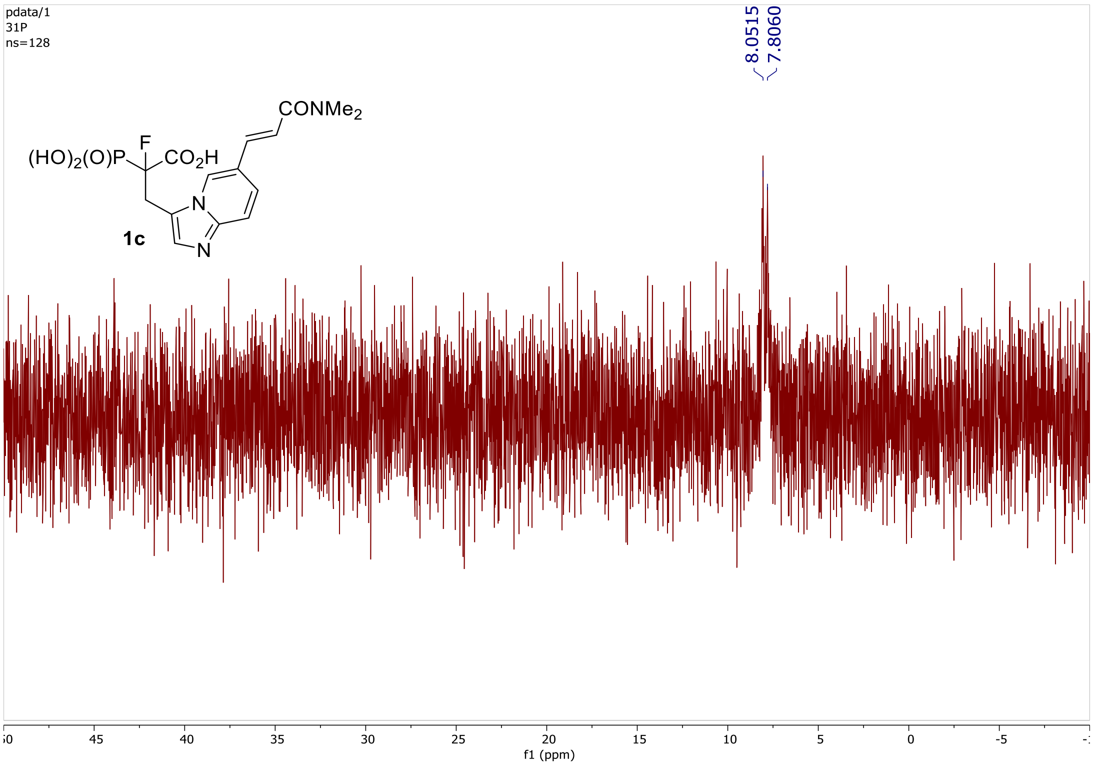

Figure S12. ³¹P NMR (283 MHz, D_2O, pH 4) of compound **1c.**

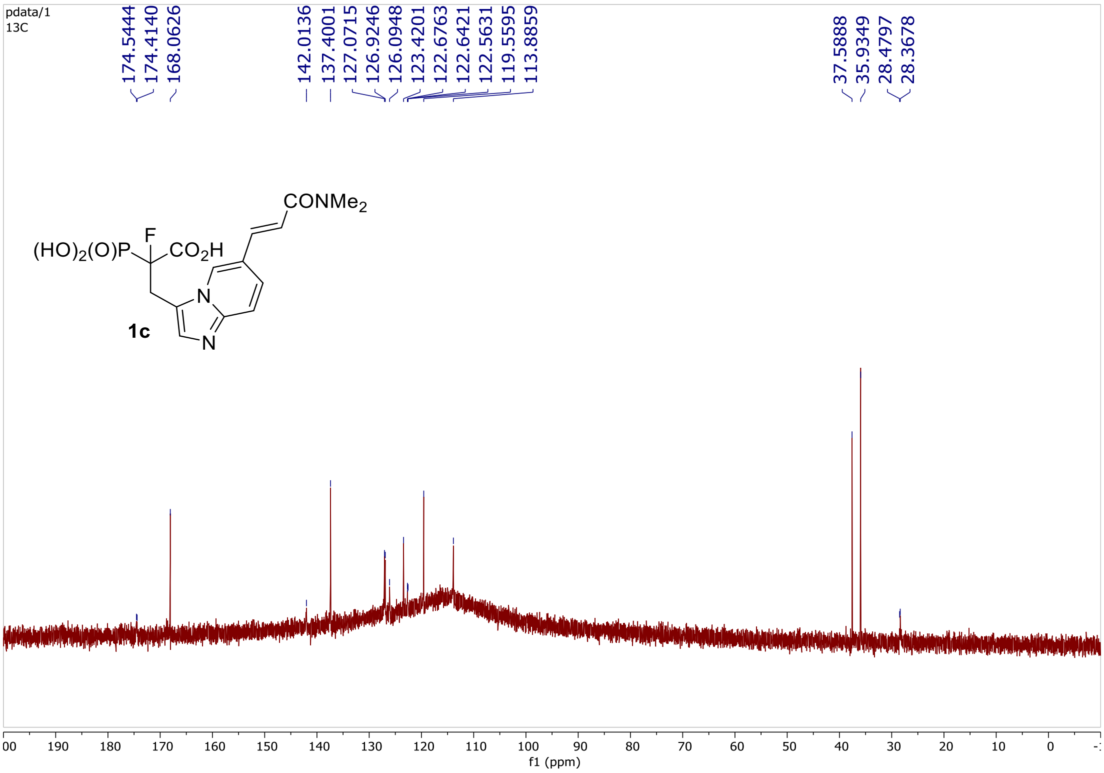

Figure S13. ^{13}C NMR (176 MHz, D_2O, pH 4) of compound **1c**.

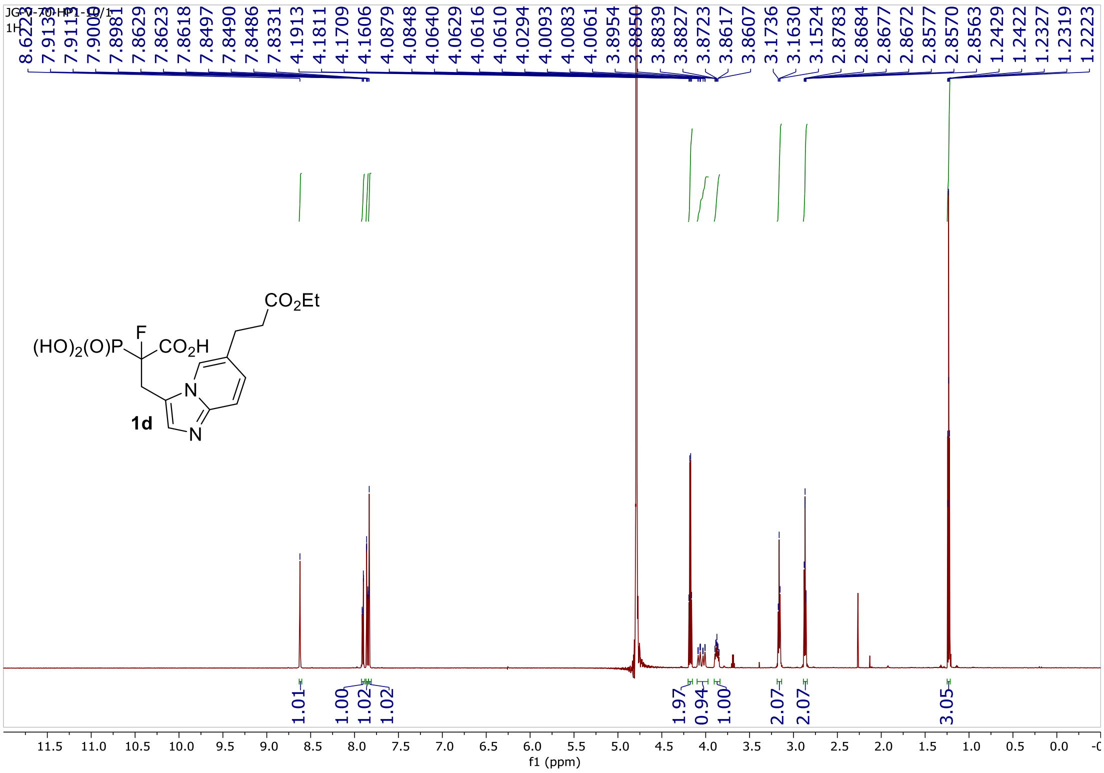

Figure S14. ^{1}H NMR (700 MHz, D_2O, pH 2) of compound **1d**.

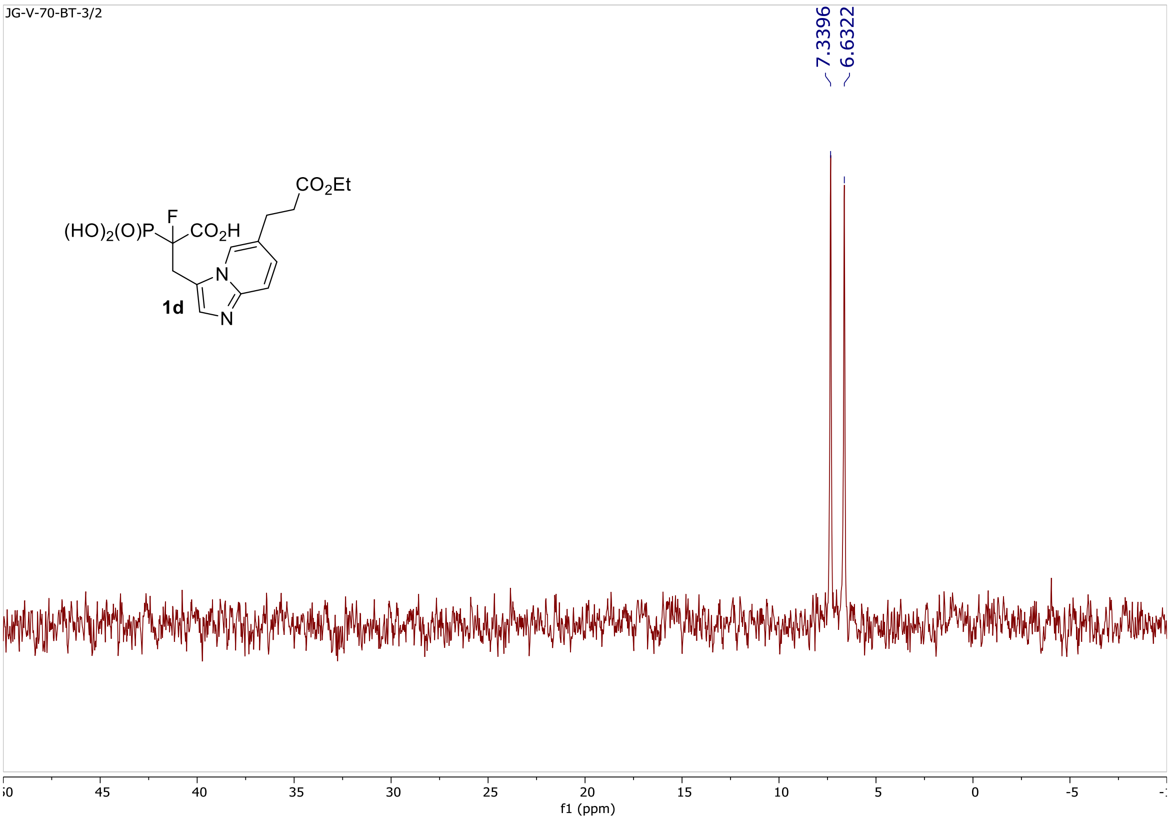

Figure S15. ^{31}P NMR (101 MHz, D_2O, pH 2) of compound **1d**.

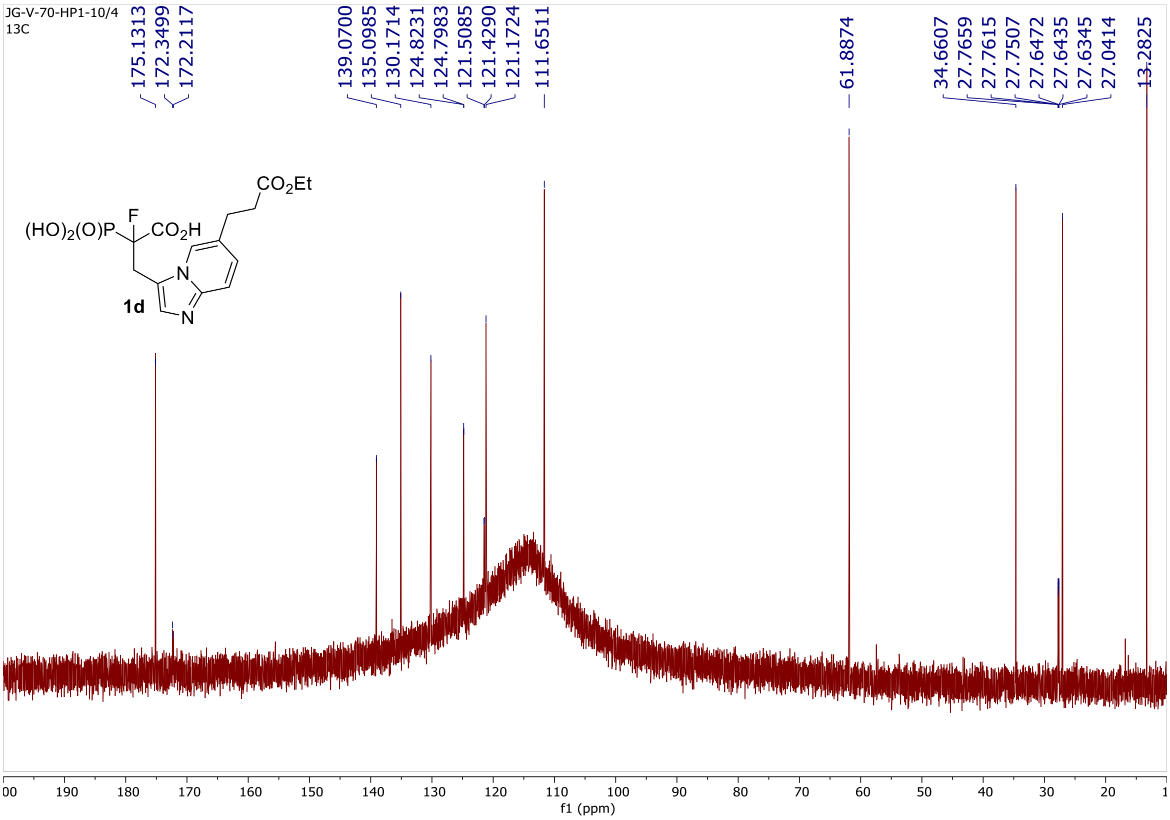

Figure S16. ^{13}C NMR (176 MHz, D_2O, pH 2) of compound **1d**.

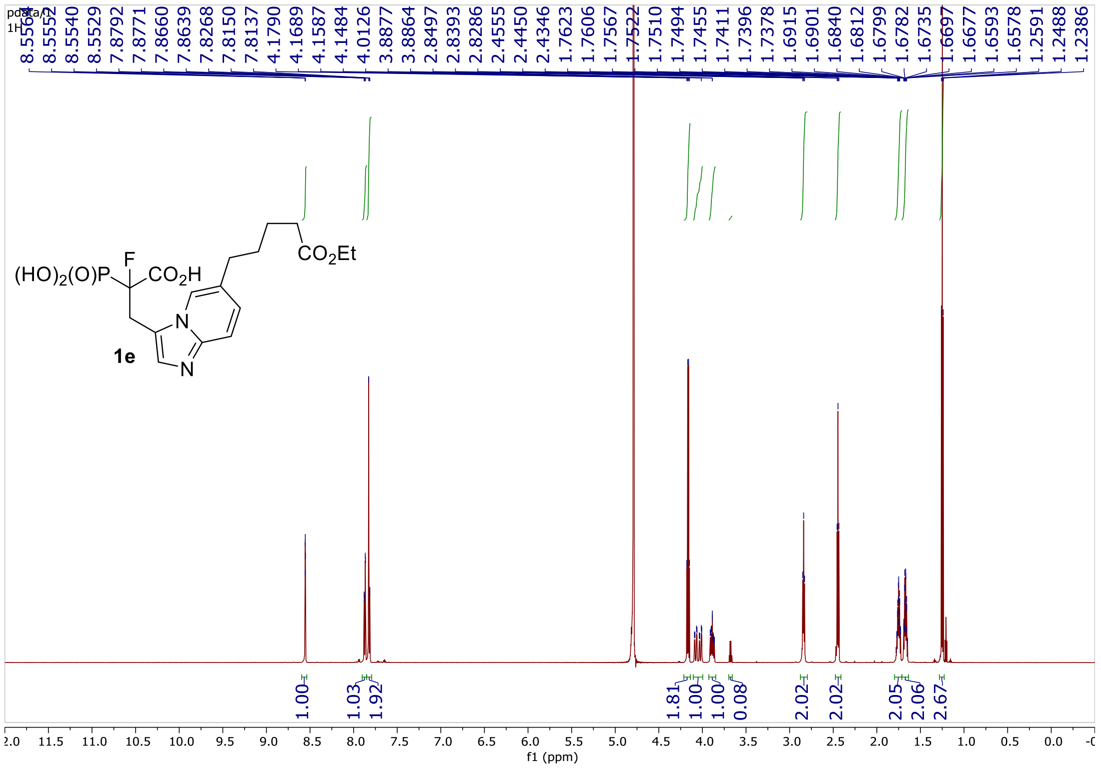

Figure S17. ^{1}H NMR (700 MHz, D_2O, pH 2) of compound **1e.**

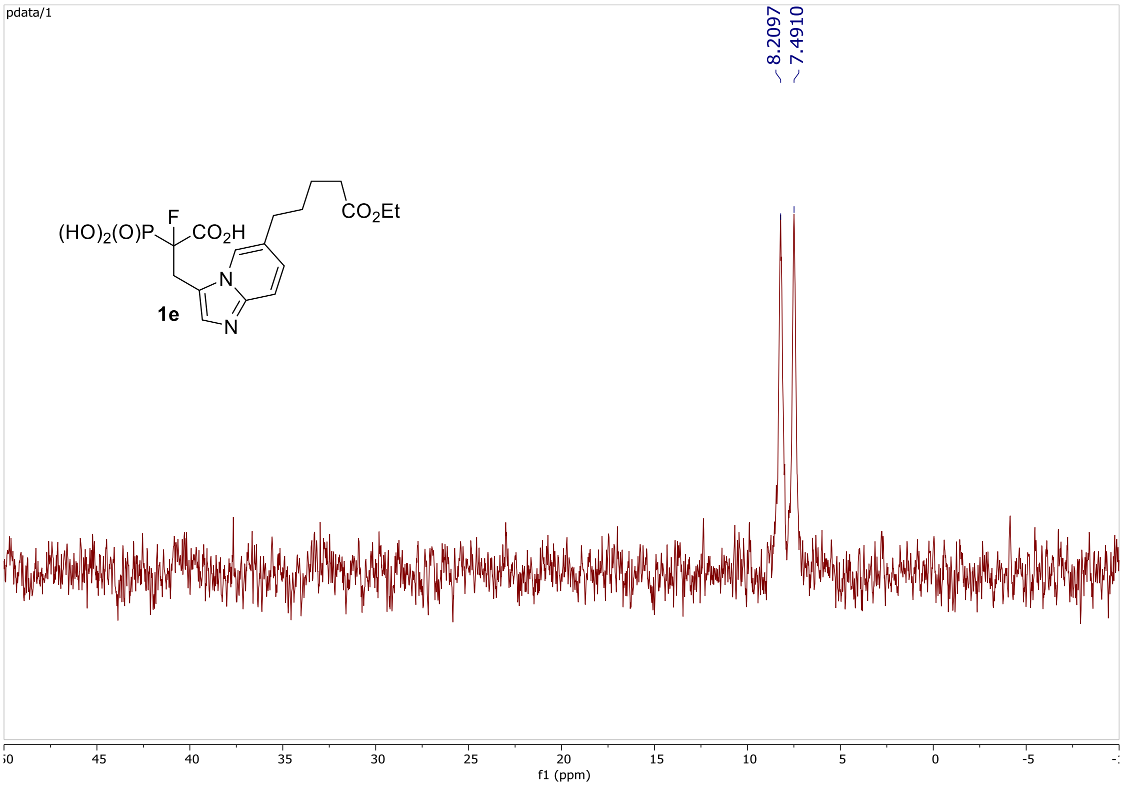

Figure S18. ^{31}P NMR (101 MHz, D_2O, pH 2) of compound **1e**.

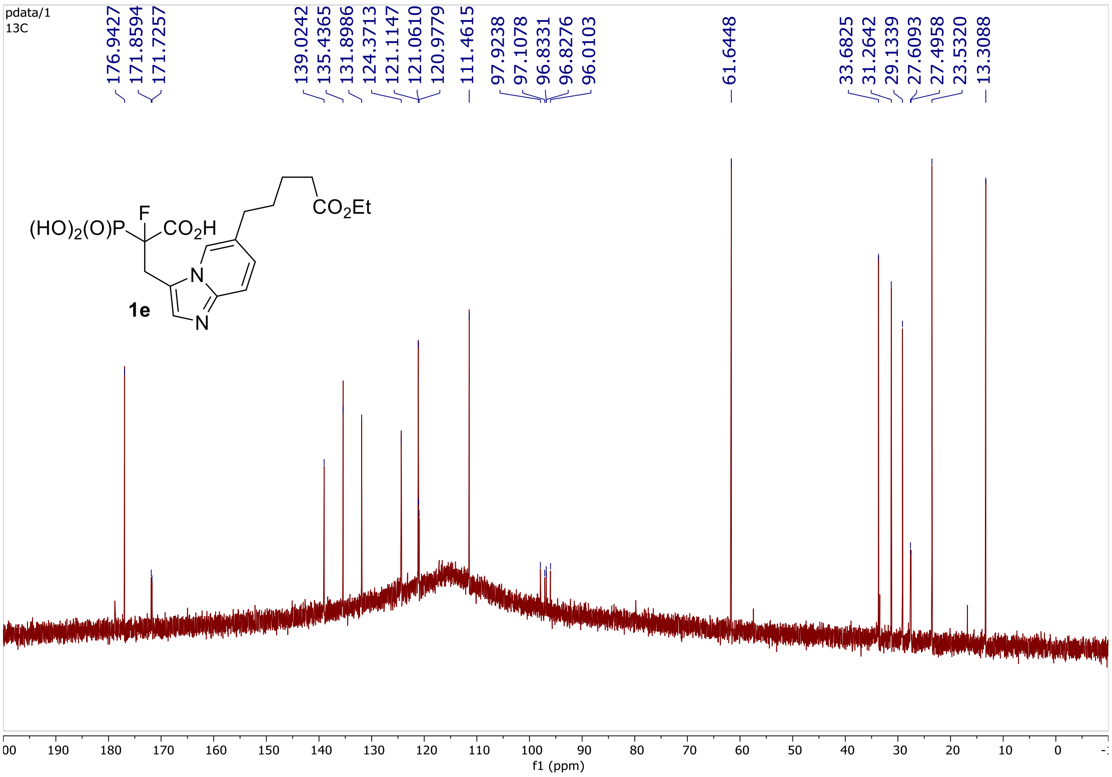

Figure S19. ^{13}C NMR (176 MHz, D_2O, pH 2) of compound **1e**.

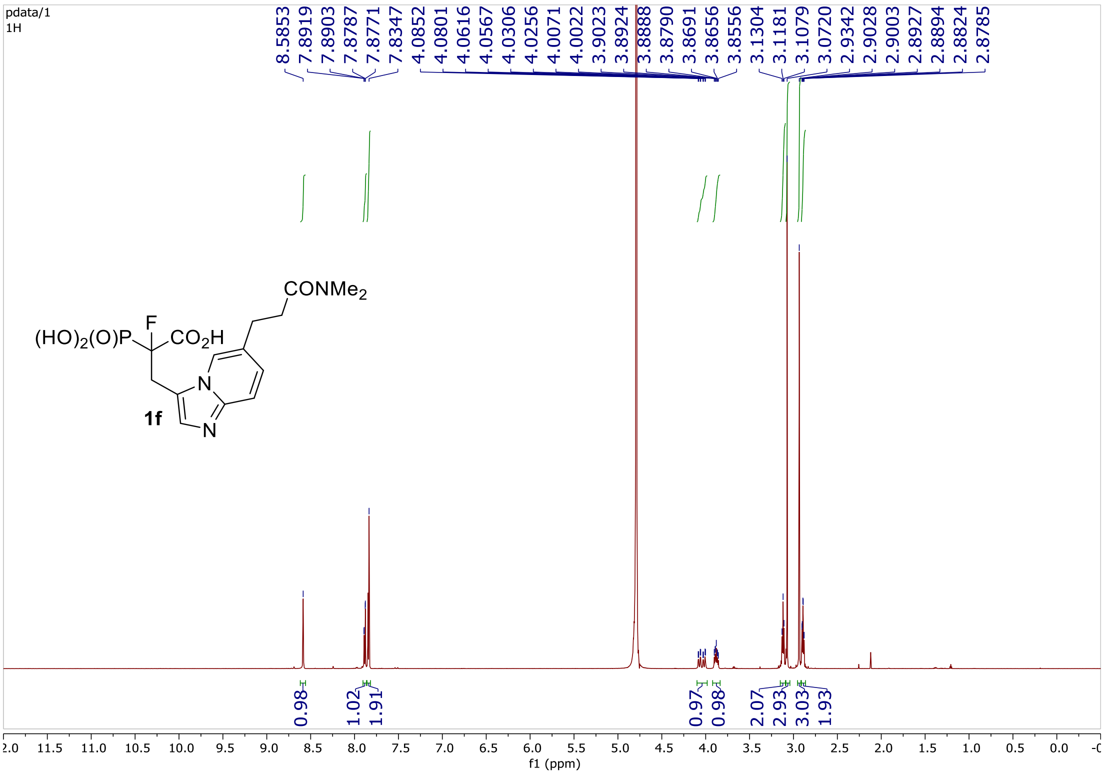

Figure S20. ^{1}H NMR (700 MHz, D_2O, pH 2) of compound **1f**.

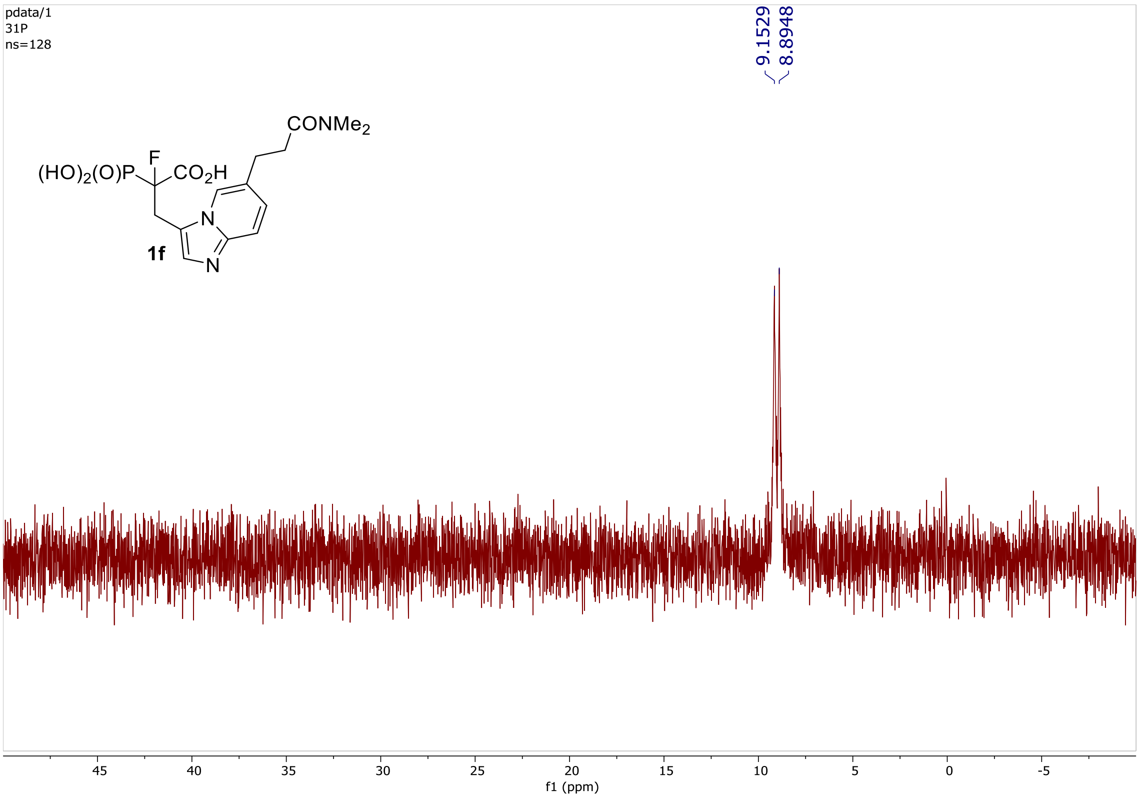

Figure S21. ^{31}P NMR (283 MHz, D_2O, pH 3) of compound **1f**.

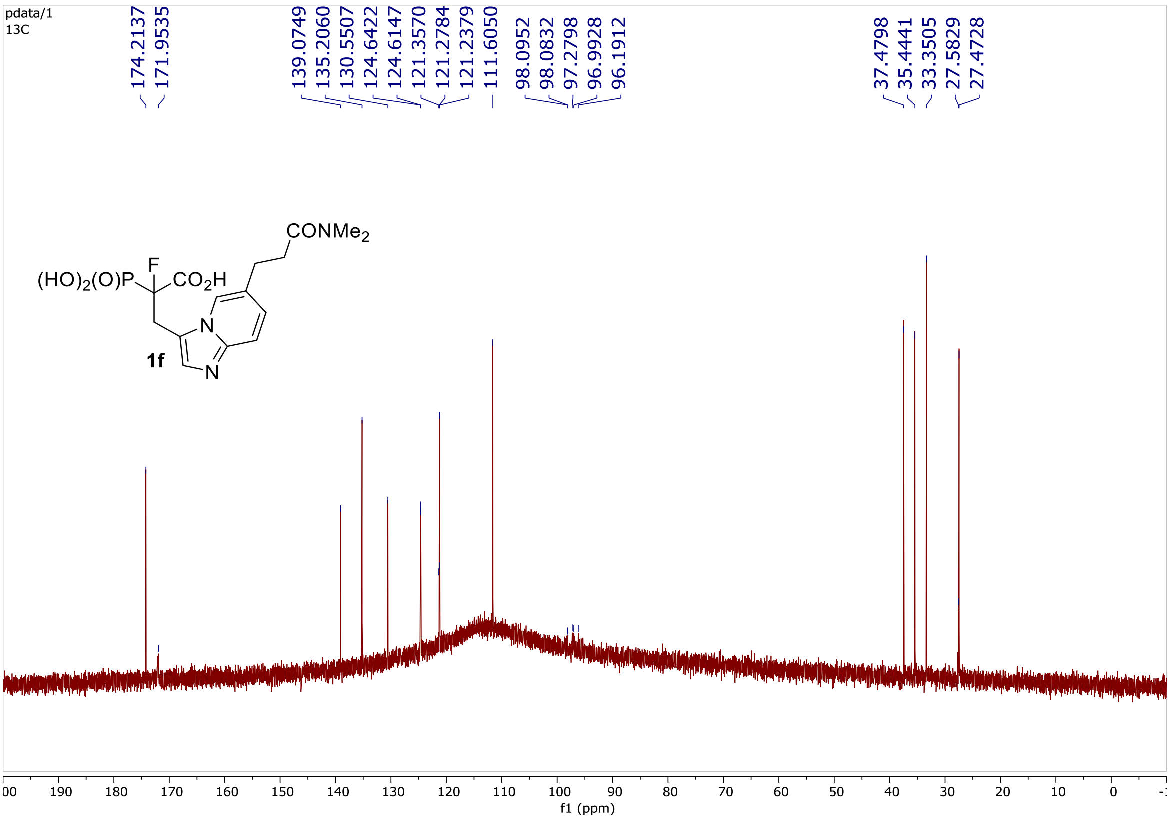

Figure S22. ^{13}C NMR (176 MHz, D_2O, pH 2) of compound **1f**.

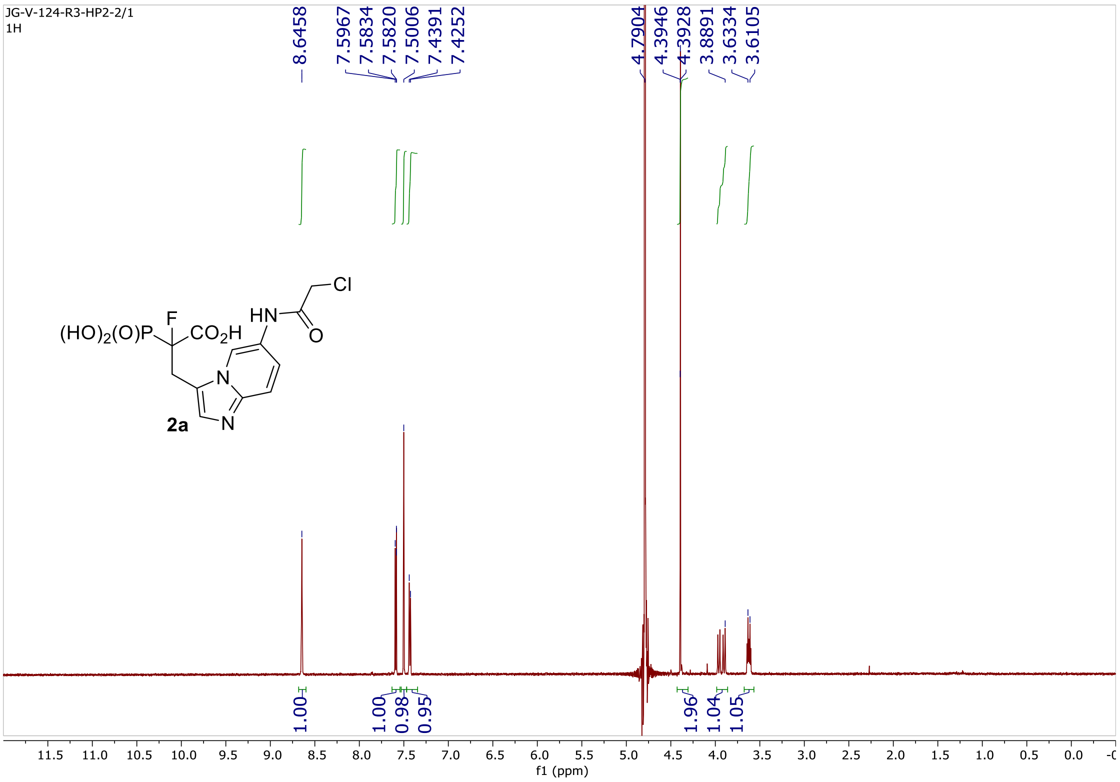

Figure S23. ^{1}H NMR (700 MHz, D_2O, pH 2) of compound **2a**.

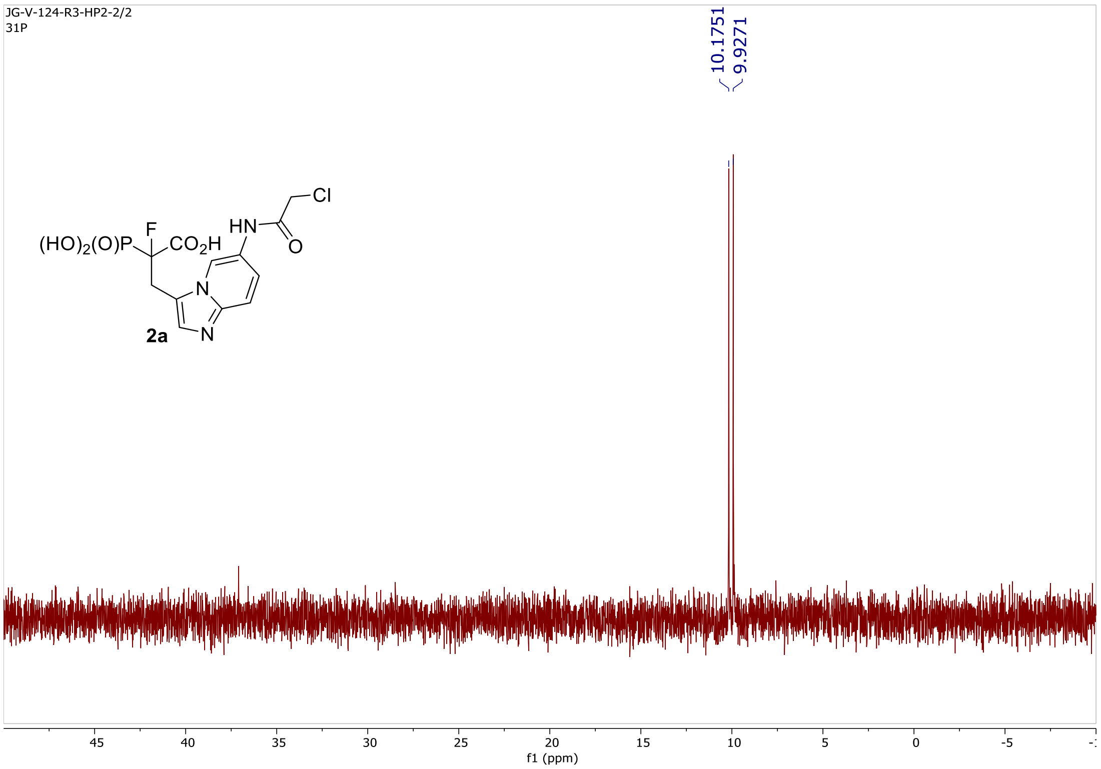

Figure S24. ^{31}P NMR (283 MHz, D_2O, pH 2) of compound **2a**.

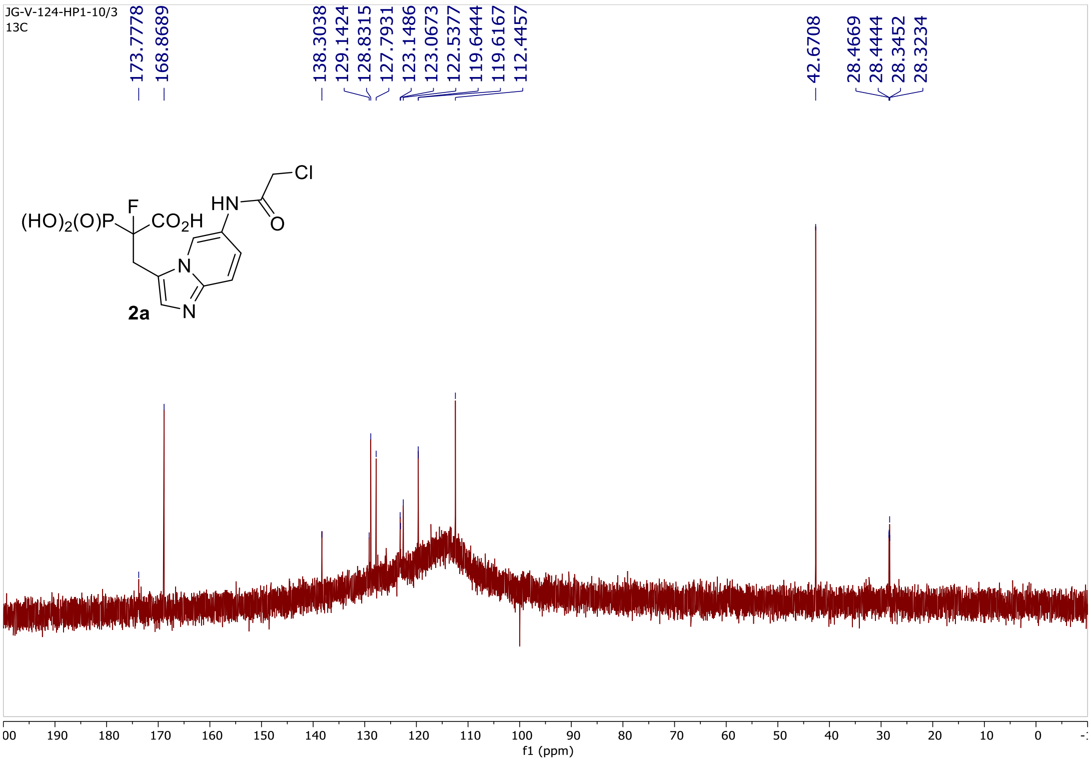

Figure S25. ^{13}C NMR (176 MHz, D_2O, pH 2) of compound **2a**.

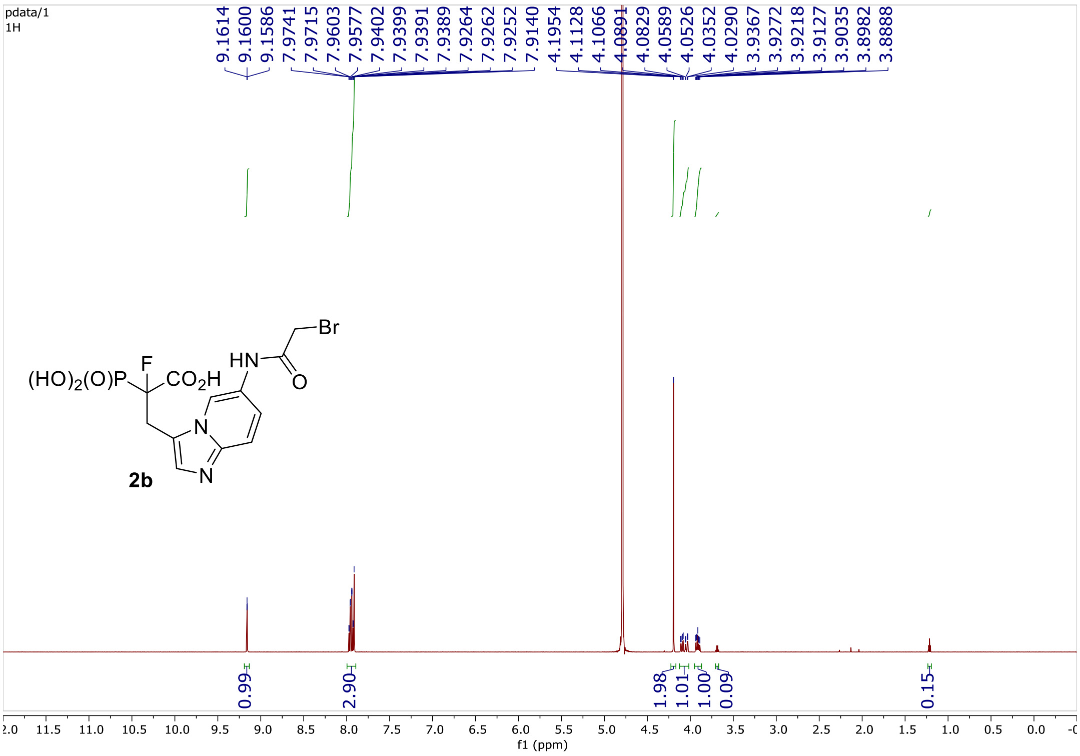

Figure S26. ^{1}H NMR (700 MHz, D_2O, pH 2) of compound **2b**.

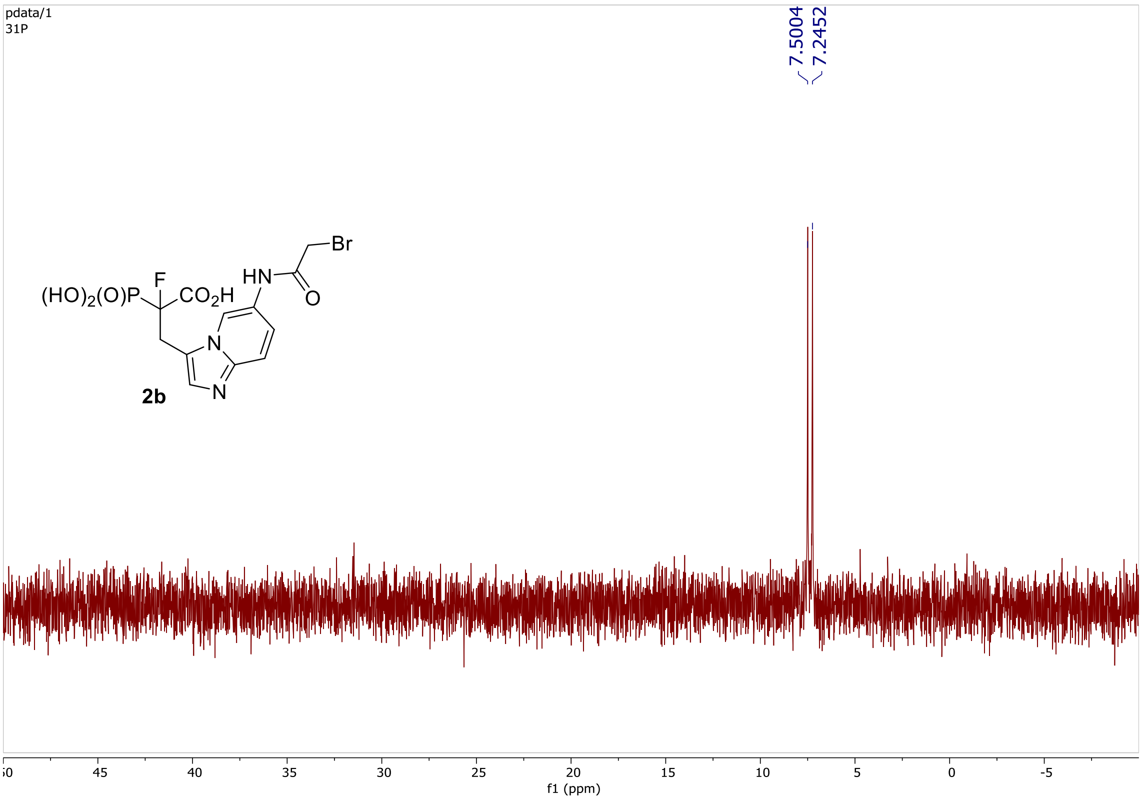

Figure S27. ^{31}P NMR (283 MHz, D_2O, pH 2) of compound **2b**.

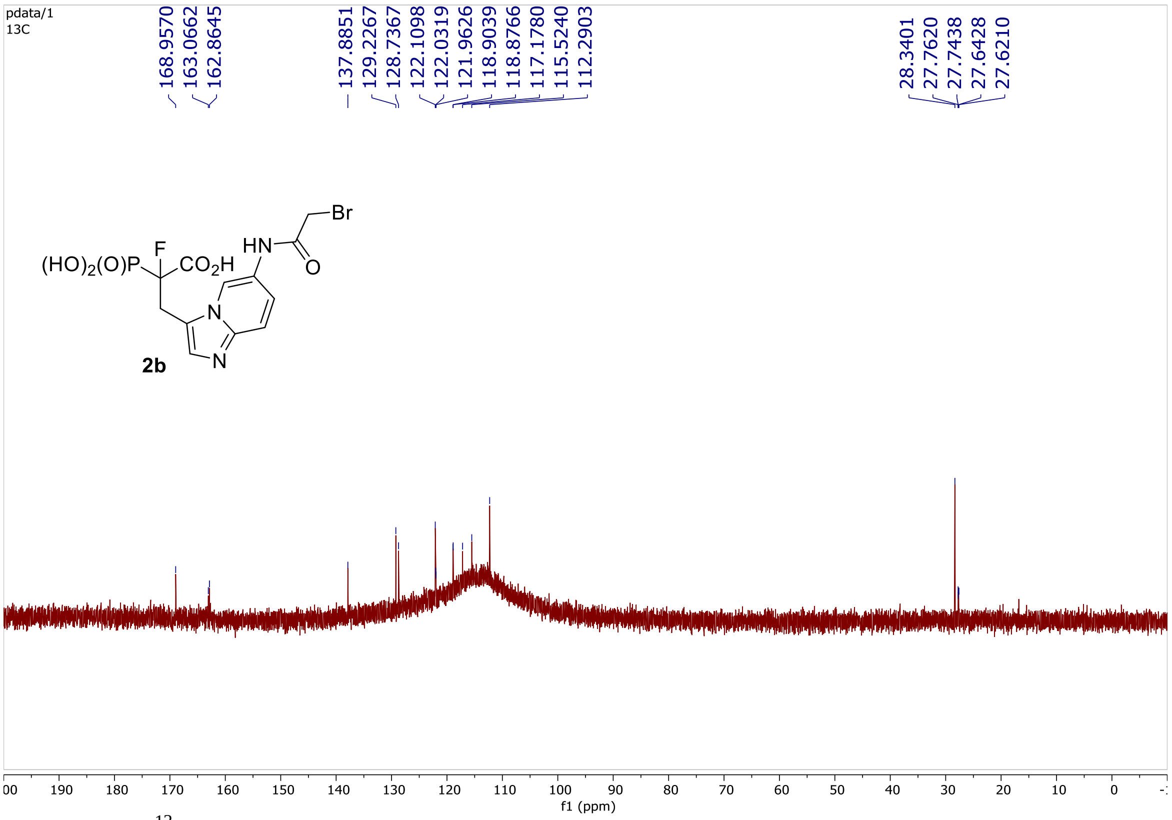

Figure S28. ^{13}C NMR (176 MHz, D_2O, pH 2) of compound **2b.**

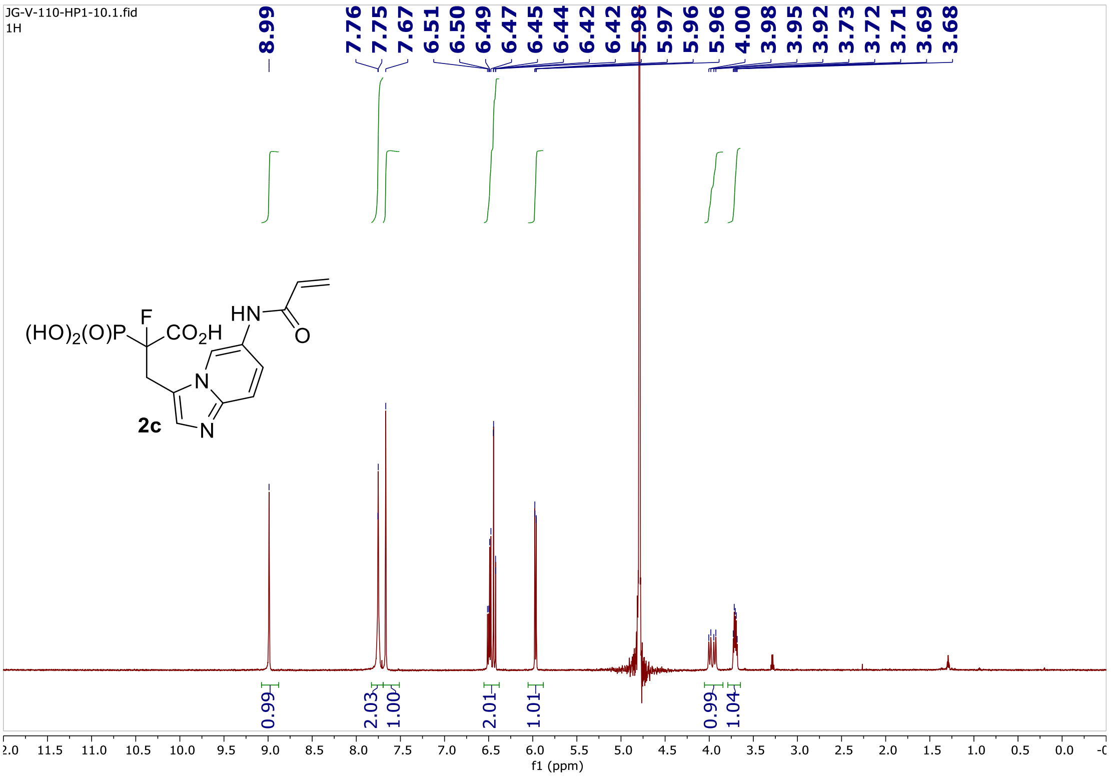

Figure S29. ^{1}H NMR (700 MHz, D_2O, pH 7) of compound **2c**.

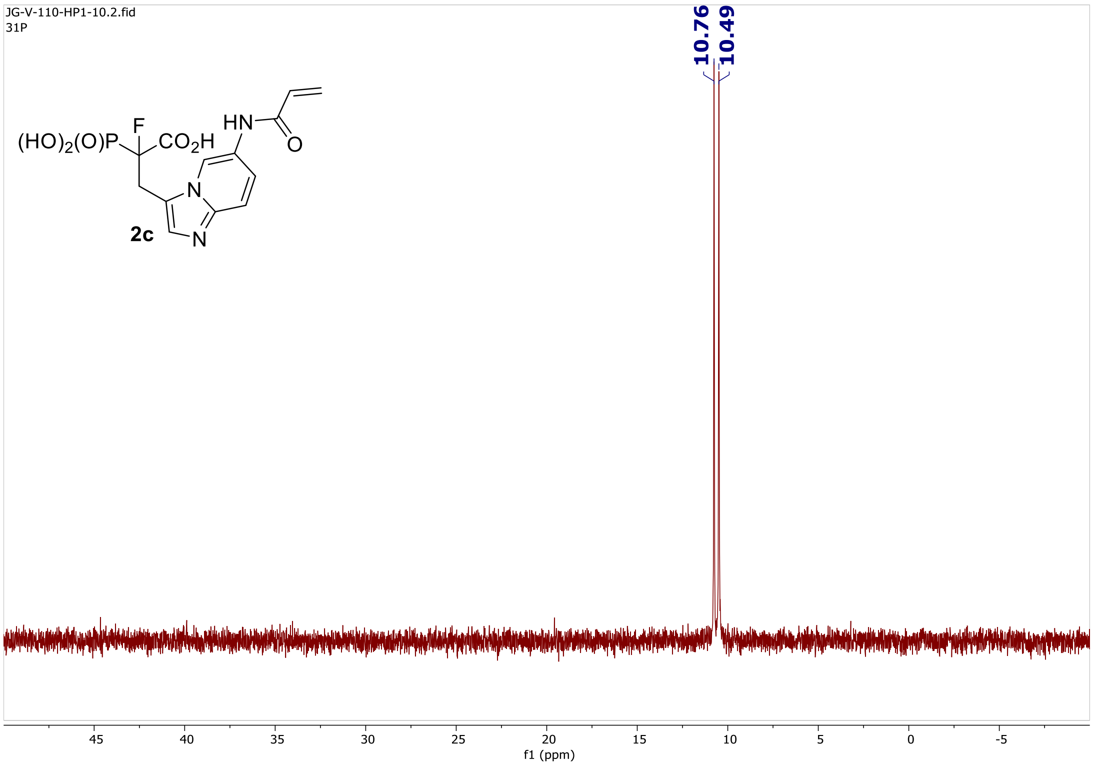

Figure S30. ^{31}P NMR (283 MHz, D_2O, pH 2) of compound **2c**.

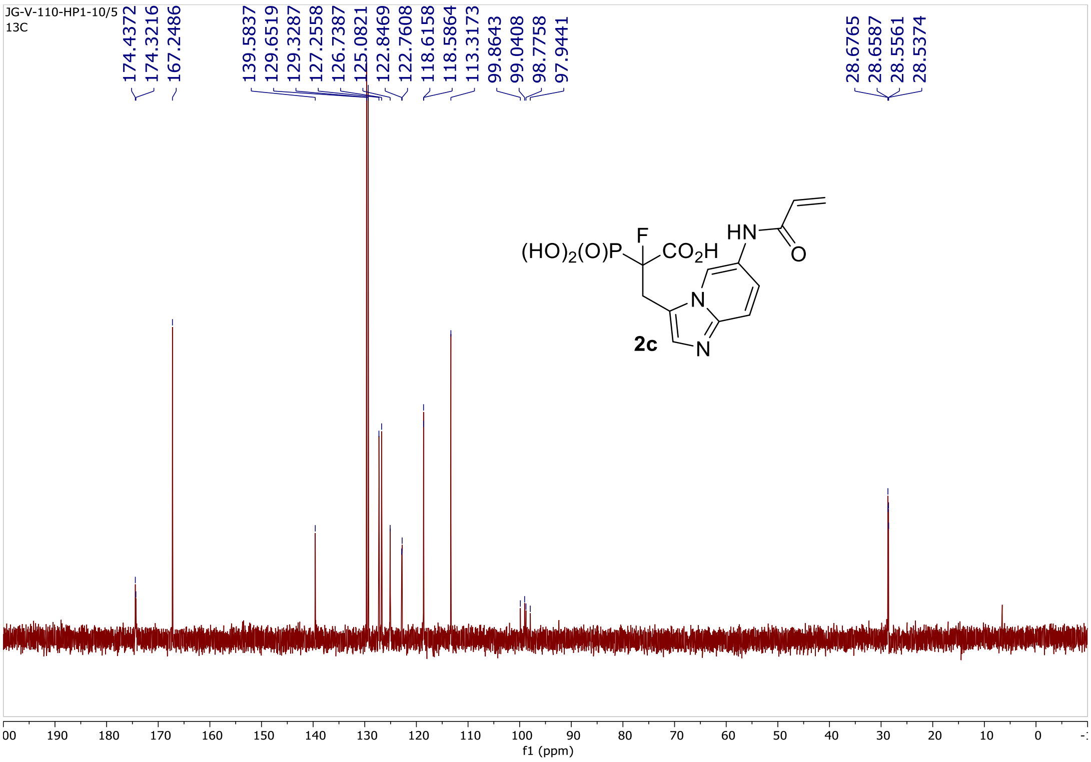

Figure S31. ^{13}C NMR (176 MHz, D_2O, pH 2) of compound **2c**.

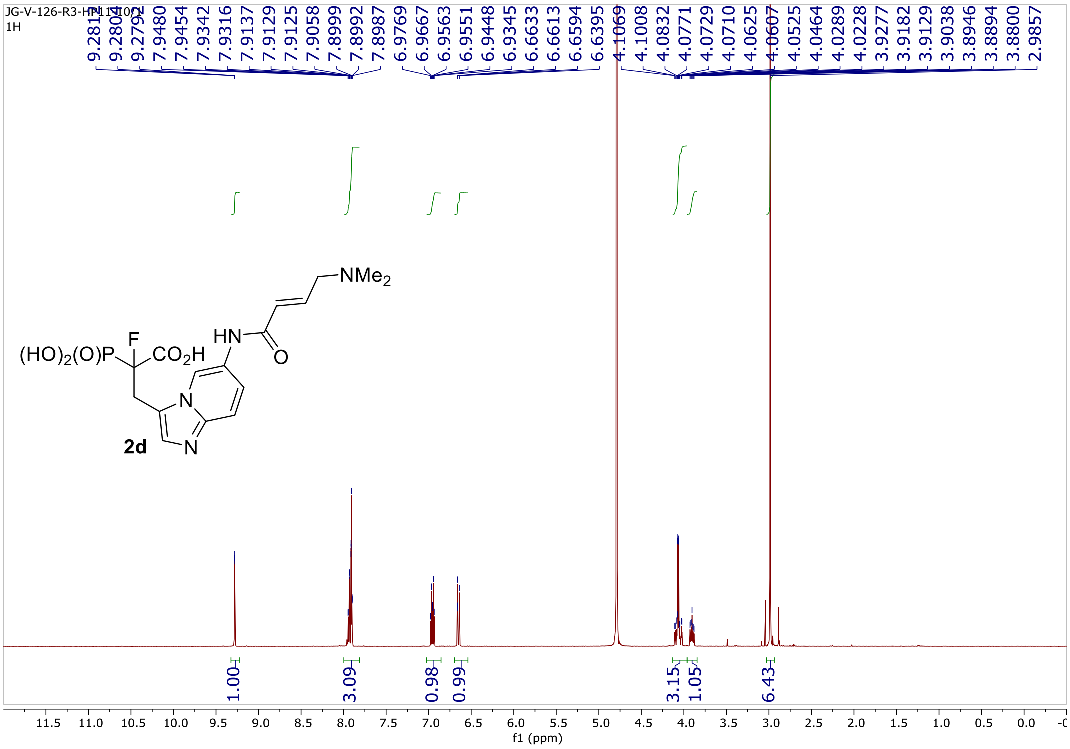

Figure S32. ^{1}H NMR (700 MHz, D_2O, pH 2) of compound **2d**.

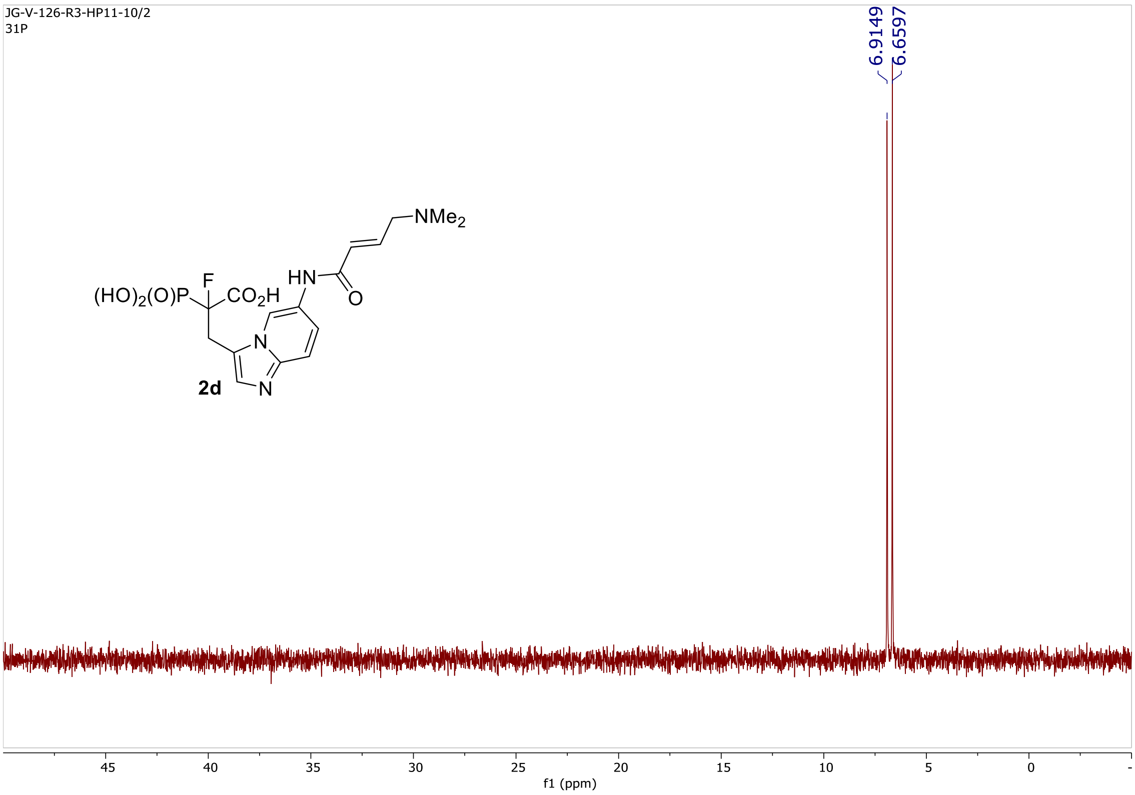

Figure S33. ^{31}P NMR (283 MHz, D_2O, pH 2) of compound **2d.**

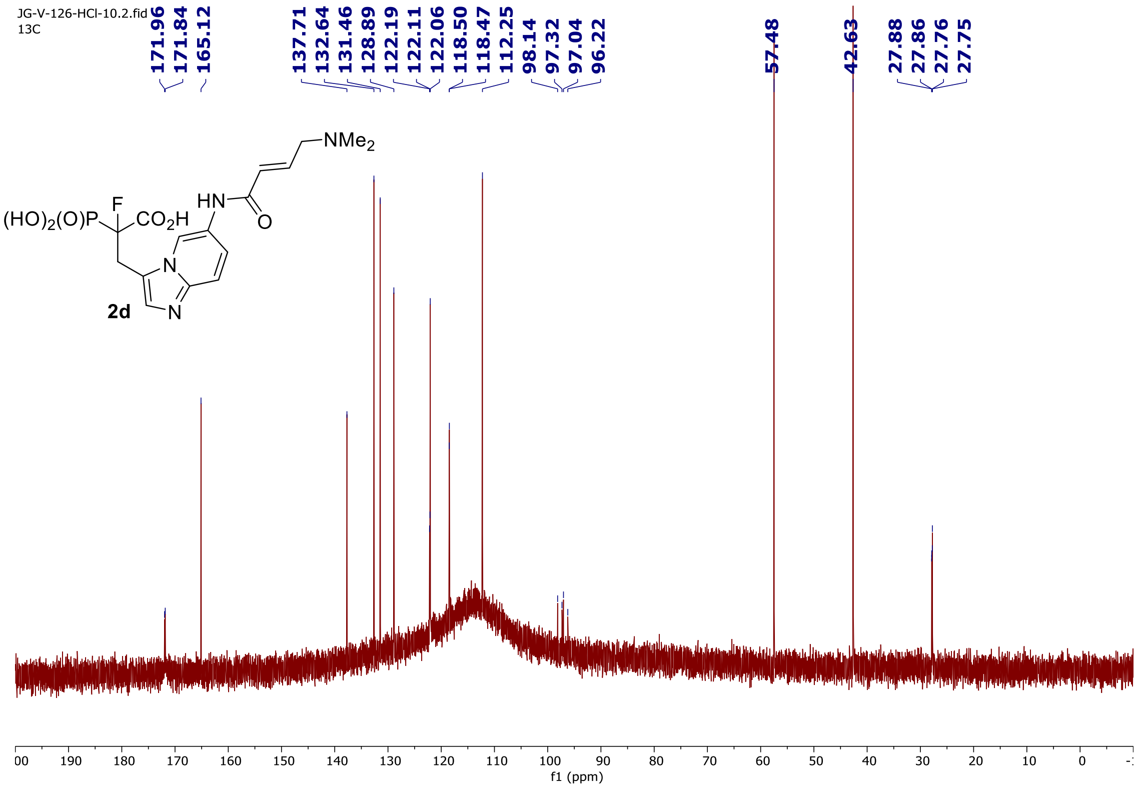

Figure S34. ^{13}C NMR (176 MHz, D_2O, pH 2) of compound **2d**.

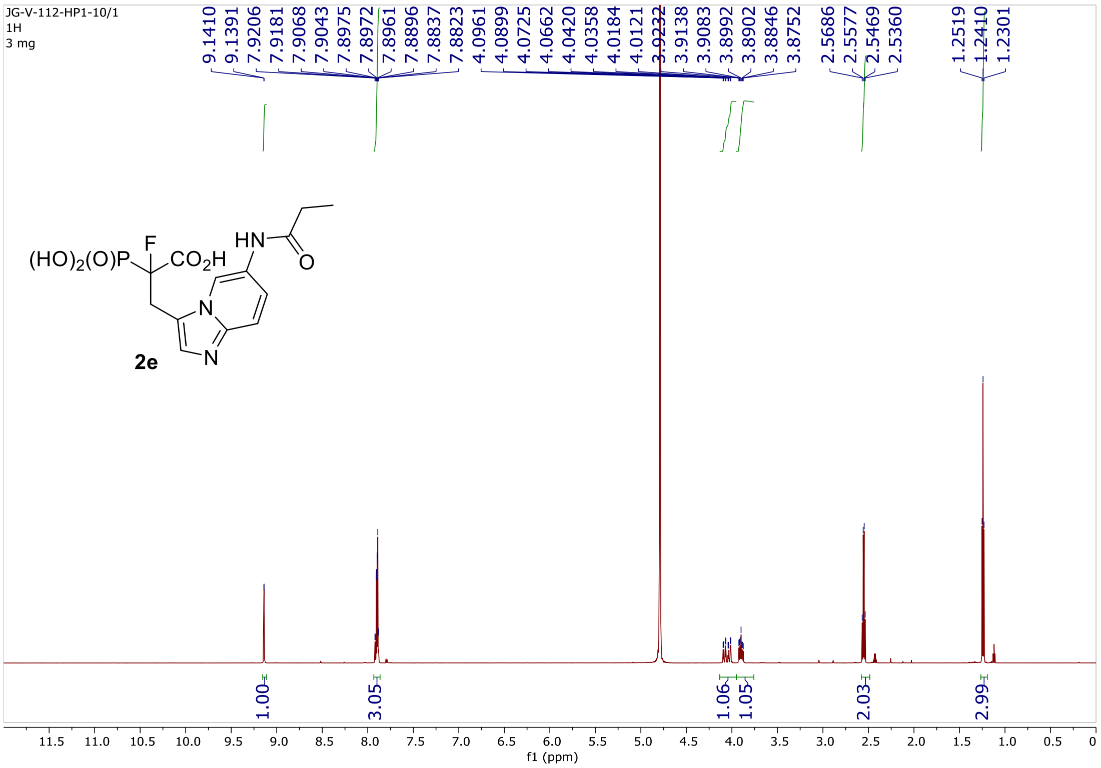

Figure S35. ^{1}H NMR (700 MHz, D_2O, pH 2) of compound **2e**.

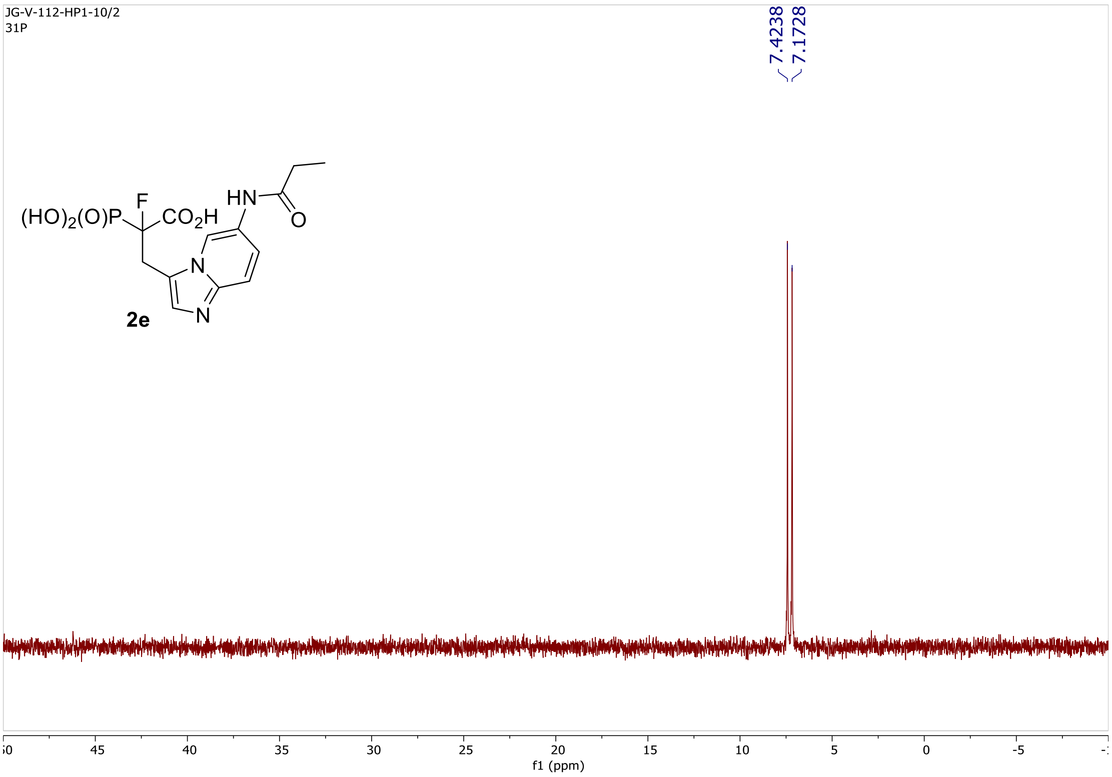

Figure S36. ^{31}P NMR (283 MHz, D_2O, pH 2) of compound **2e**.

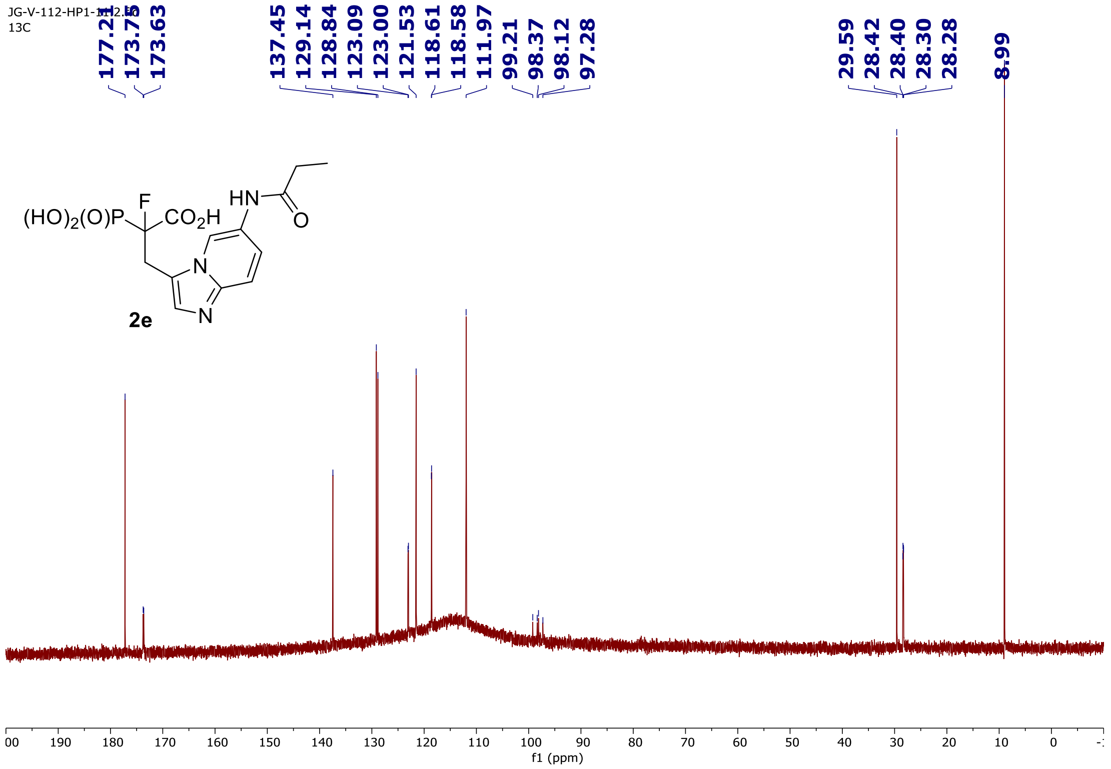

Figure S37. ^{13}C NMR (176 MHz, D_2O, pH 2) of compound **2e**.

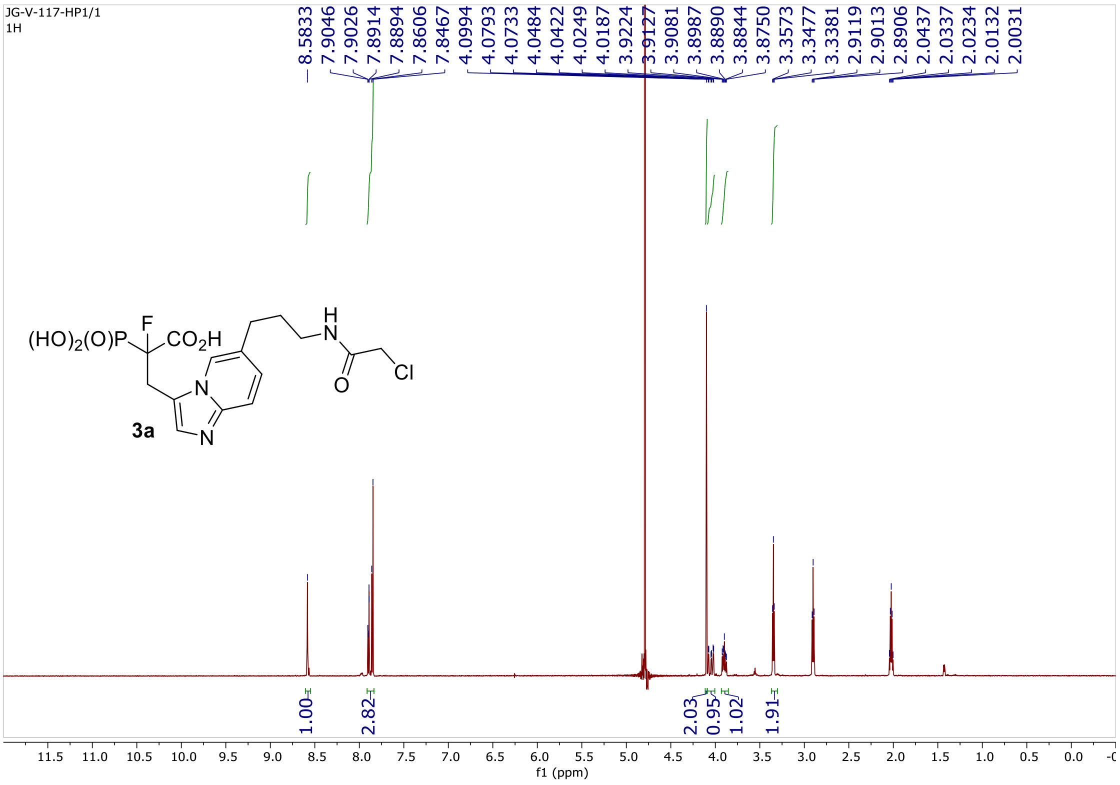

Figure S38. ^{1}H NMR (700 MHz, D_2O, pH 2) of compound **3a**.

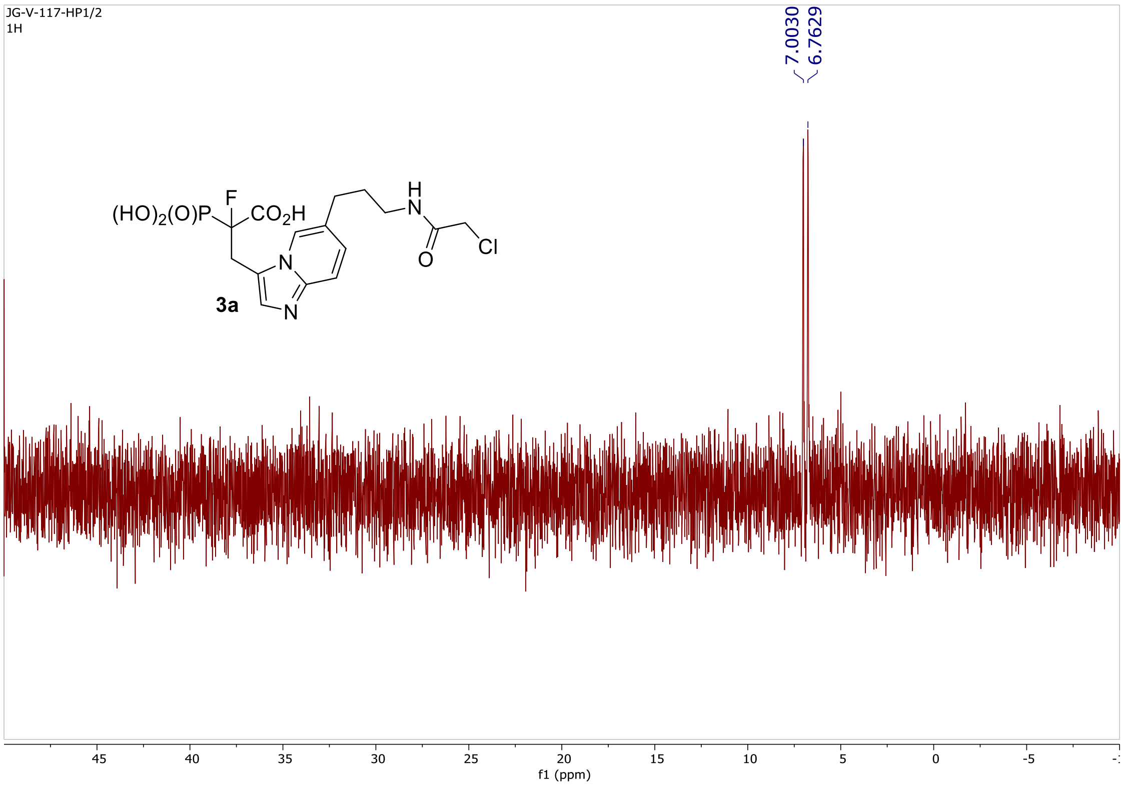

Figure S39. ^{31}P NMR (284 MHz, D_2O, pH 2) of compound **3a**.

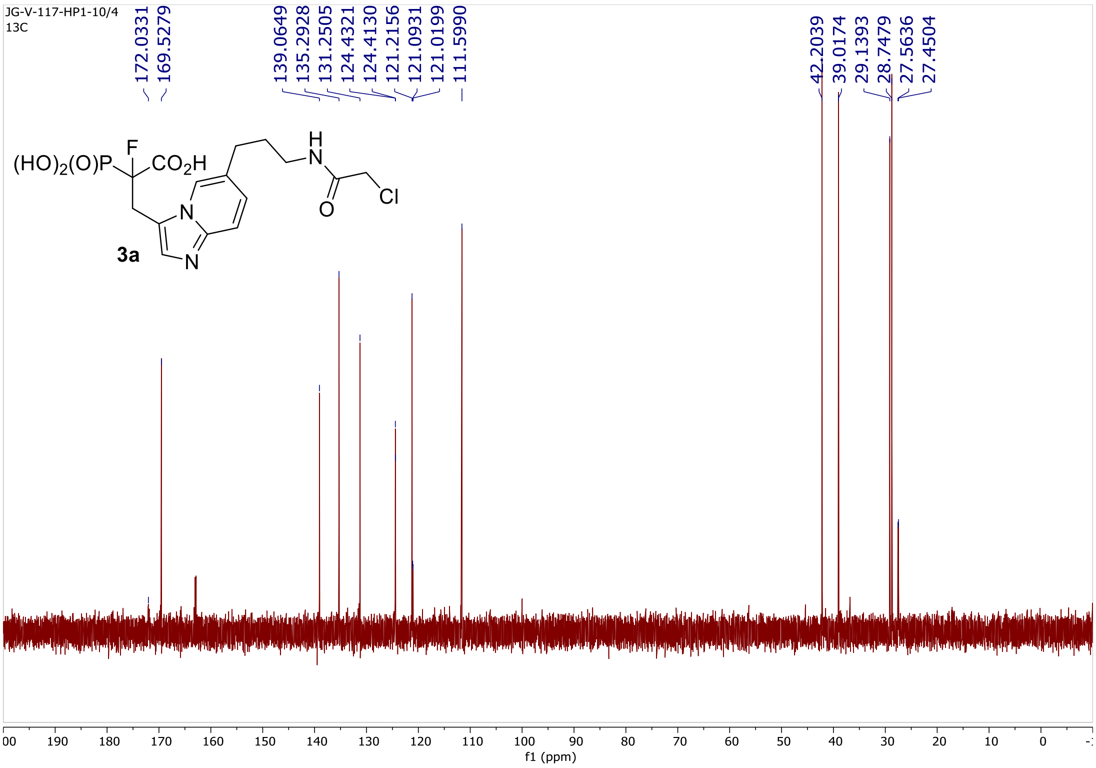

Figure S40. ^{13}C NMR (176 MHz, D_2O, pH 2) of compound **3a**.

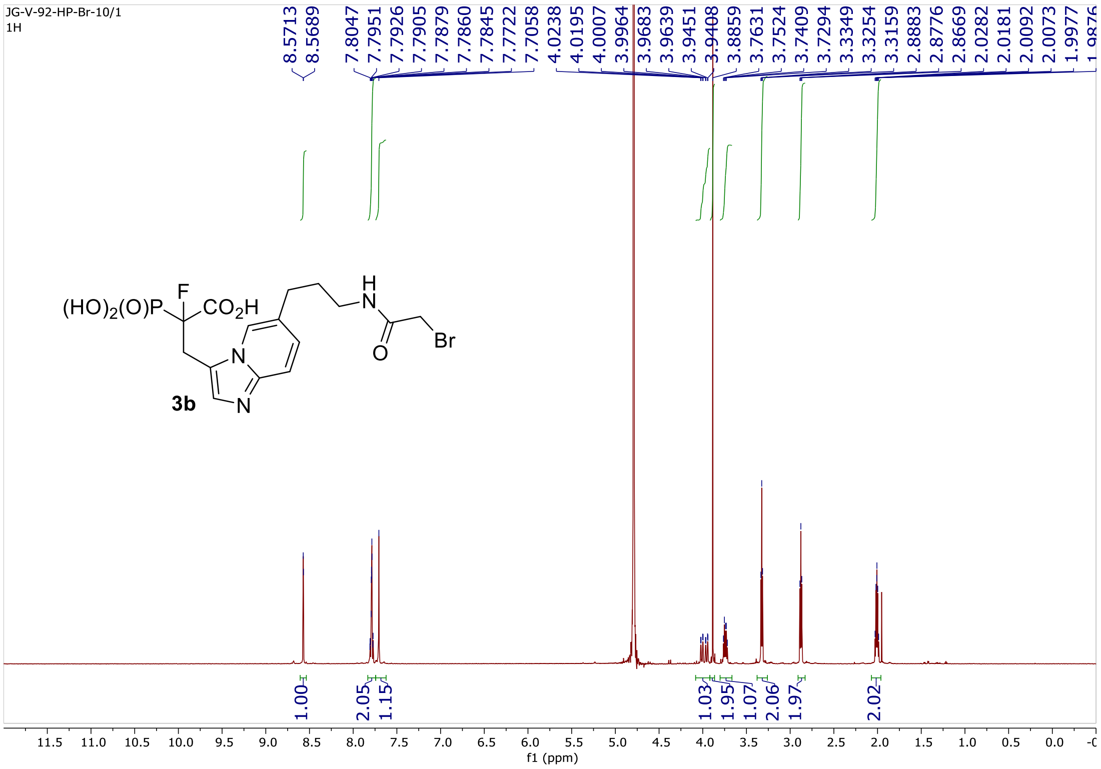

Figure S41. ^{1}H NMR (700 MHz, D_2O, pH 2) of compound **3b**.

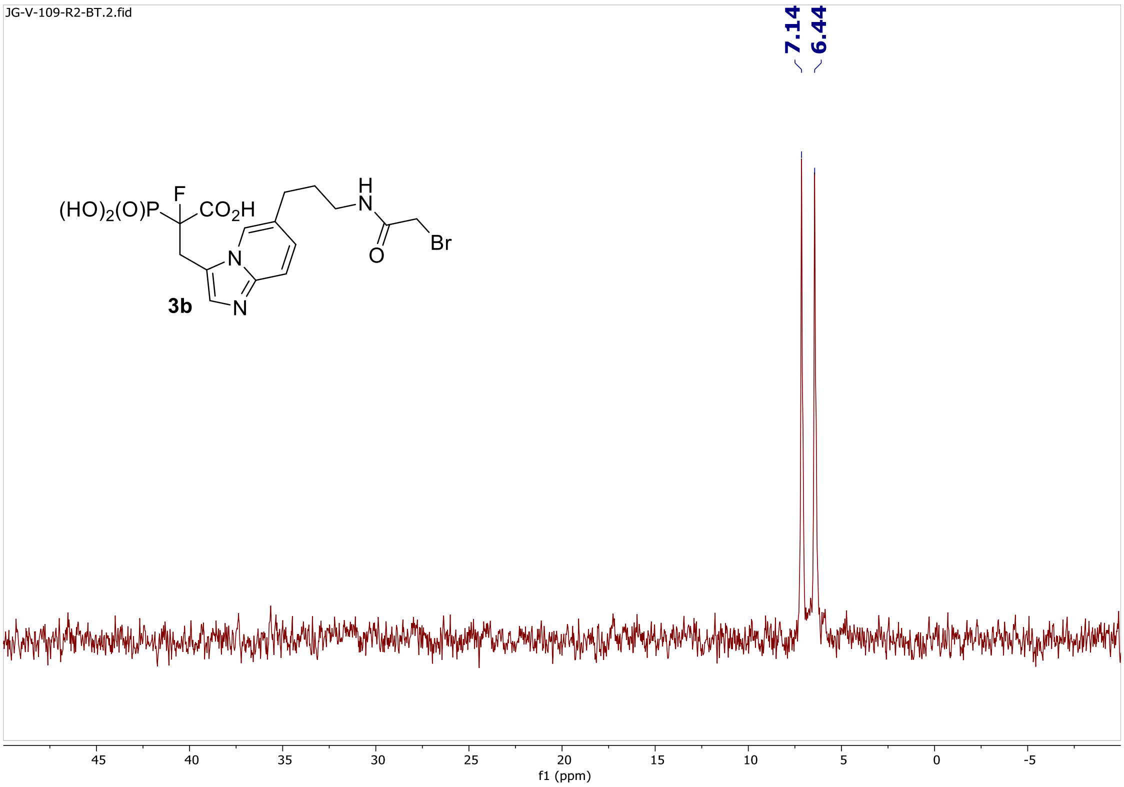

Figure S42. ^{31}P NMR (283 MHz, D_2O, pH 2) of compound **3b**.

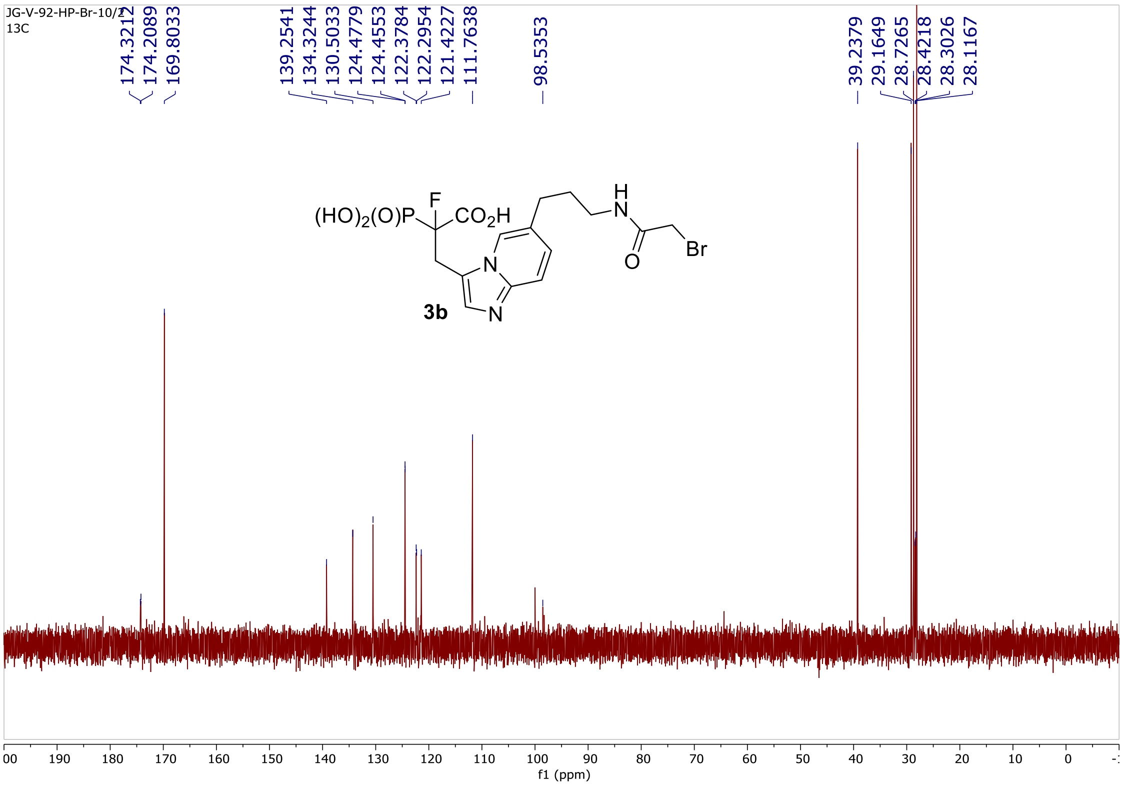

Figure S43. ^{13}C NMR (176 MHz, D_2O, pH 2) of compound **3b**.

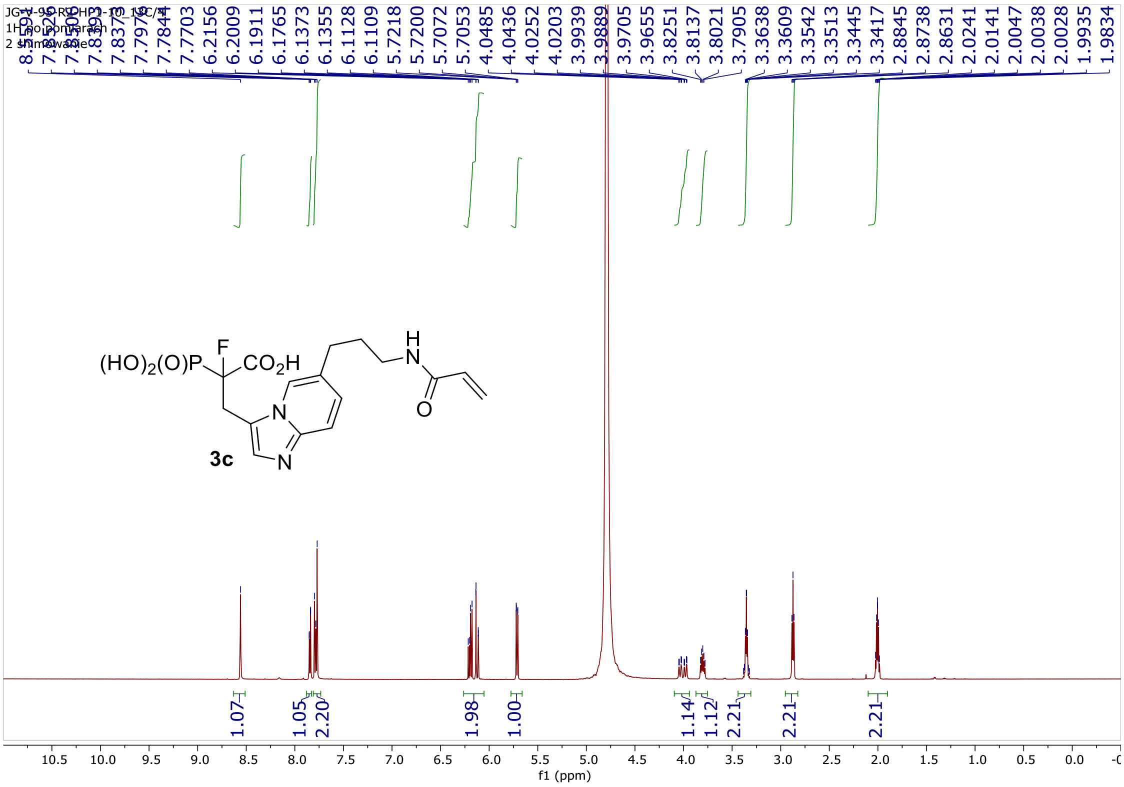

Figure S44. ^{1}H NMR (700 MHz, D_2O, pH 3) of compound **3c**.

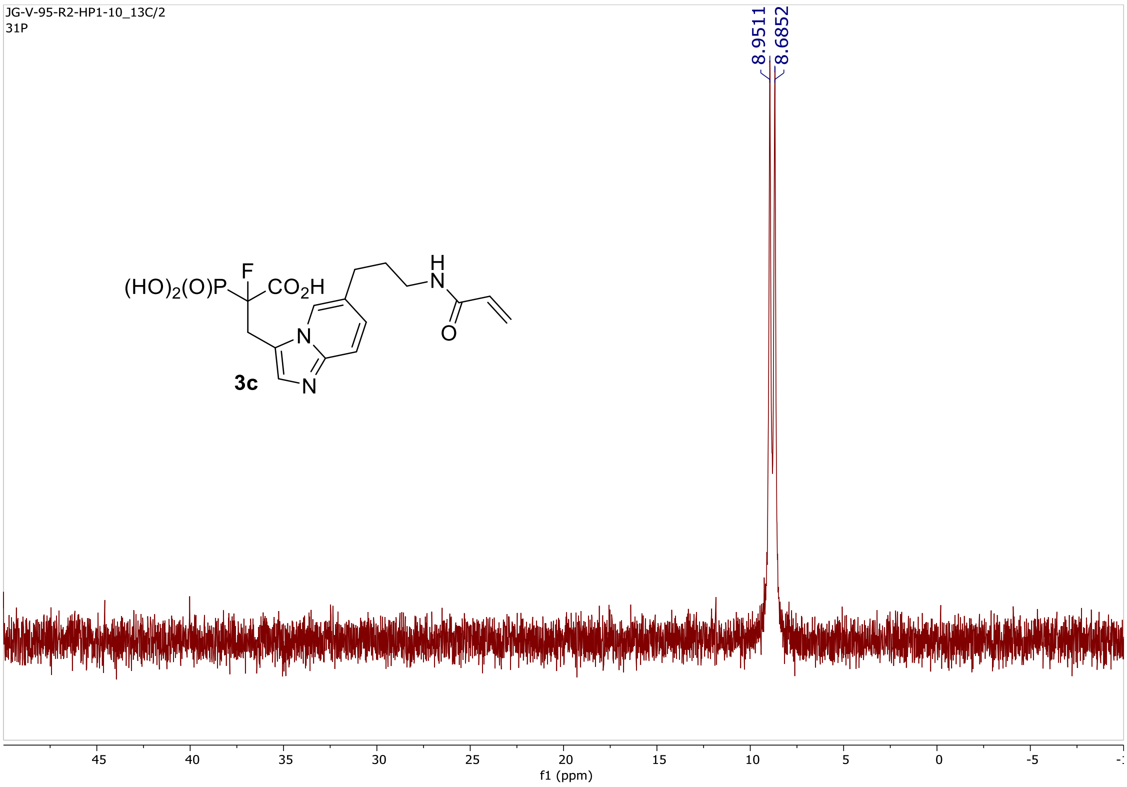

Figure S45. ^{31}P NMR (283 MHz, D_2O, pH 3) of compound **3c**.

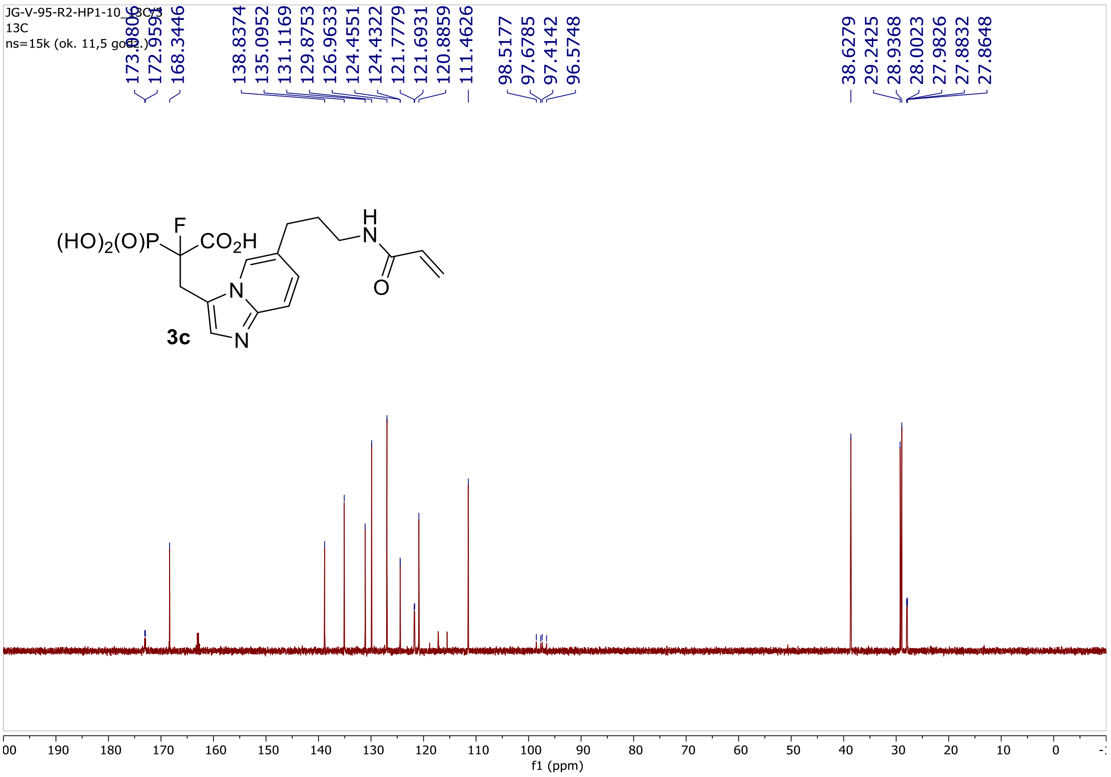

Figure S46. ^{13}C NMR (176 MHz, D_2O, pH 3) of compound **3c**.

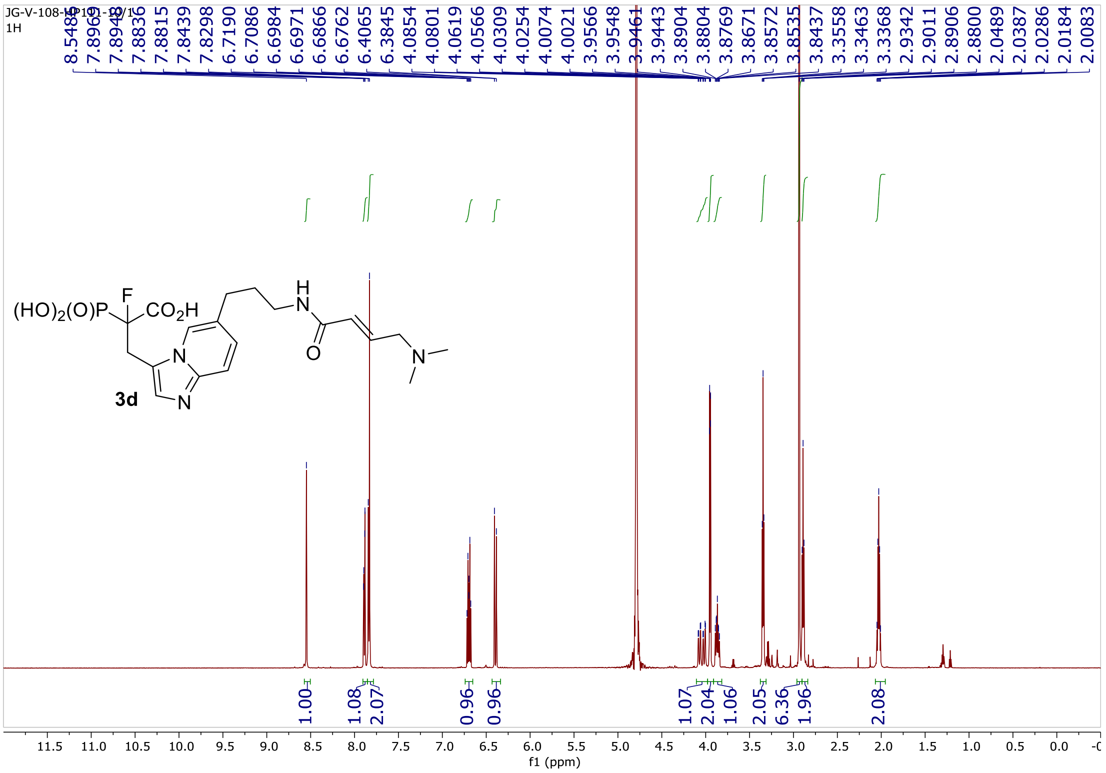

Figure S47. ^{1}H NMR (700 MHz, D_2O, pH 2) of compound **3d**.

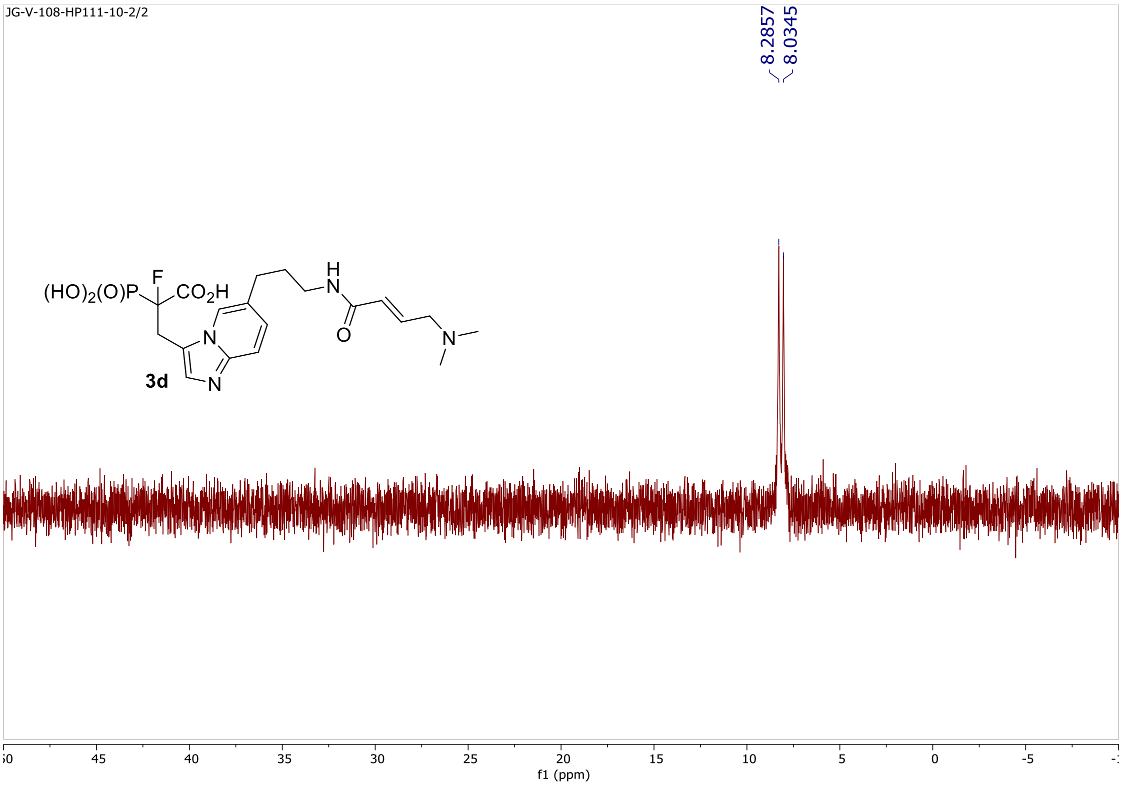

Figure S48. ^{31}P NMR (283 MHz, D_2O, pH 2) of compound **3d**.

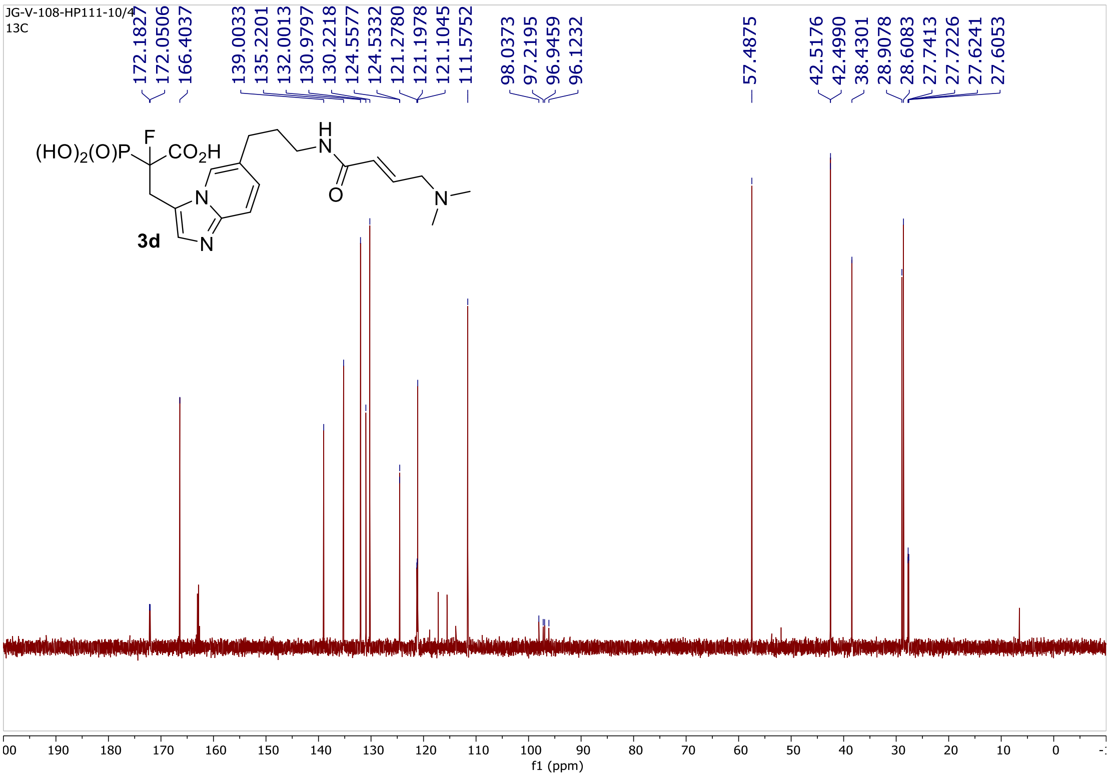

Figure S49. ^{13}C NMR (176 MHz, D_2O, pH 2) of compound **3d**.

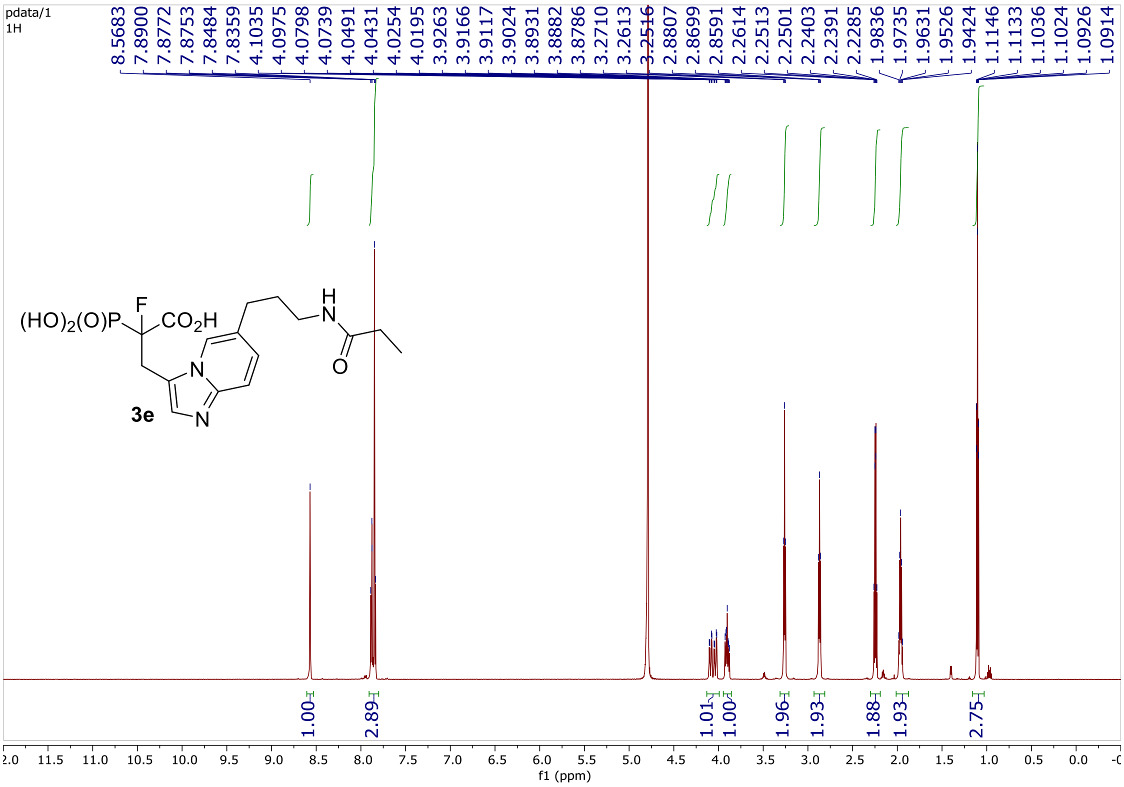

Figure S50. ^{1}H NMR (700 MHz, D_2O, pH 2) of compound **3e**.

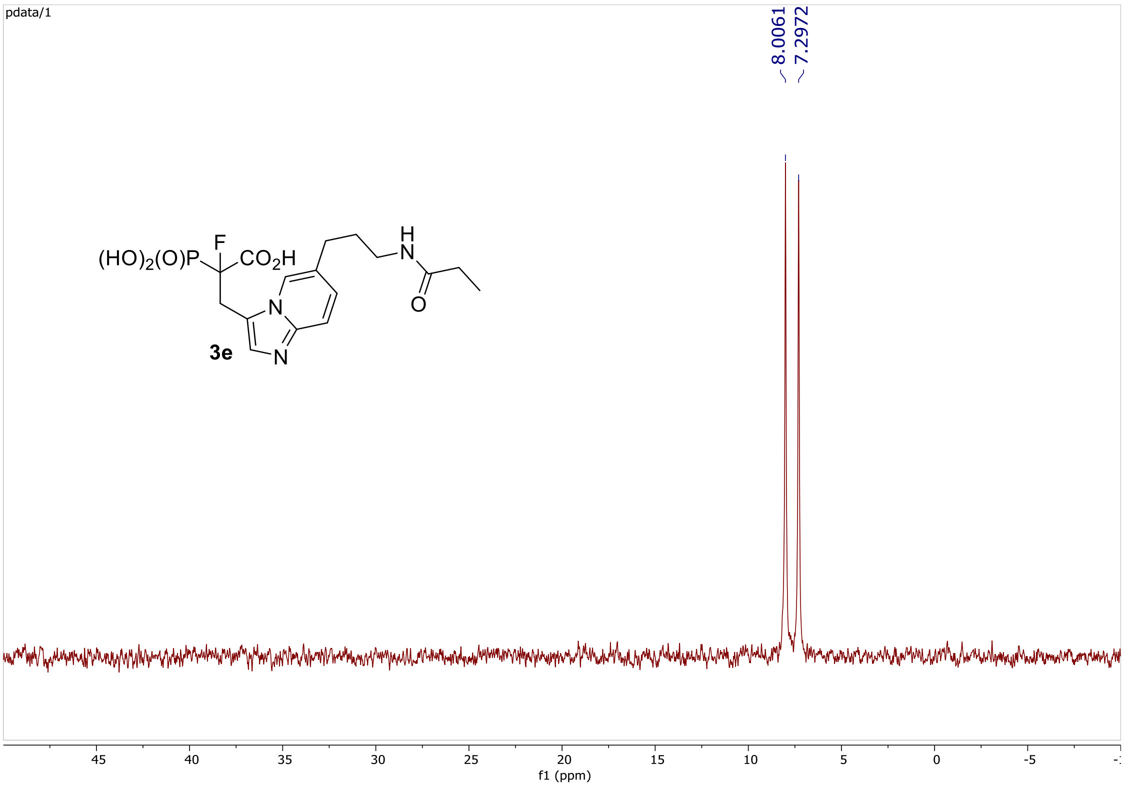

Figure S51. ^{31}P NMR (101 MHz, D_2O, pH 2) of compound **3e**.

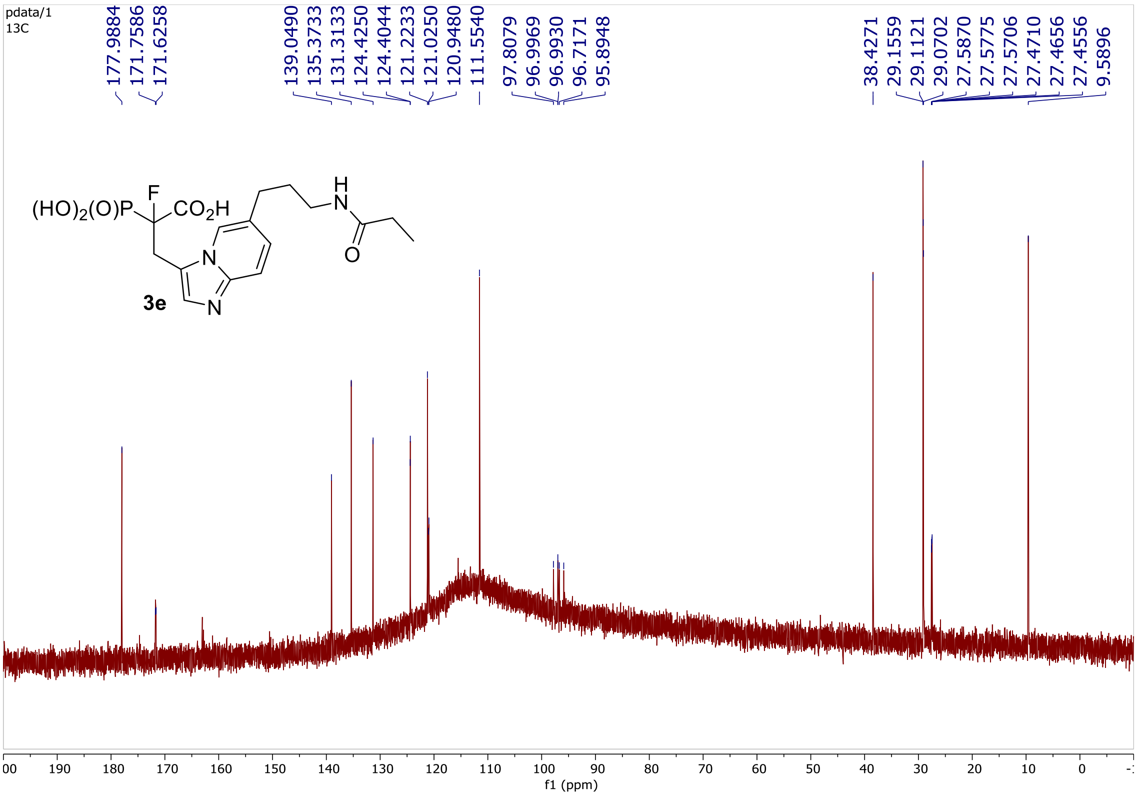

Figure S52. ^{13}C NMR (176 MHz, D_2O, pH 2) of compound **3e**.

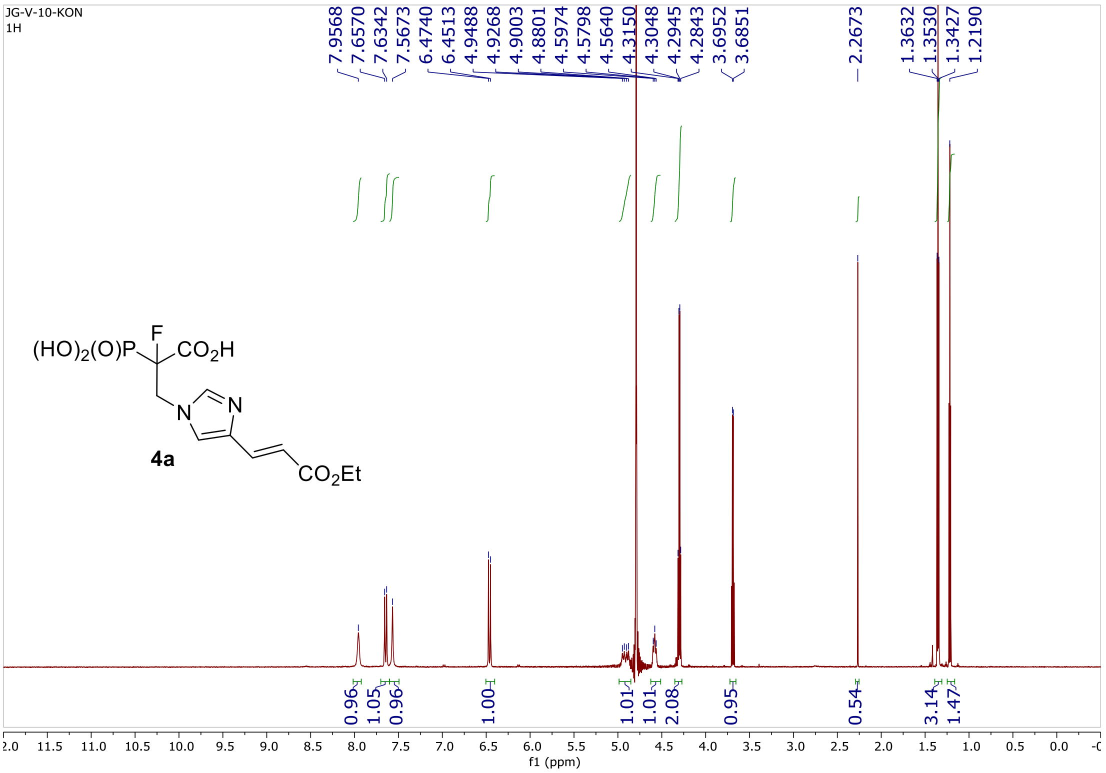

Figure S53. ^{1}H NMR (700 MHz, D_2O, pH 7) of compound **4a**.

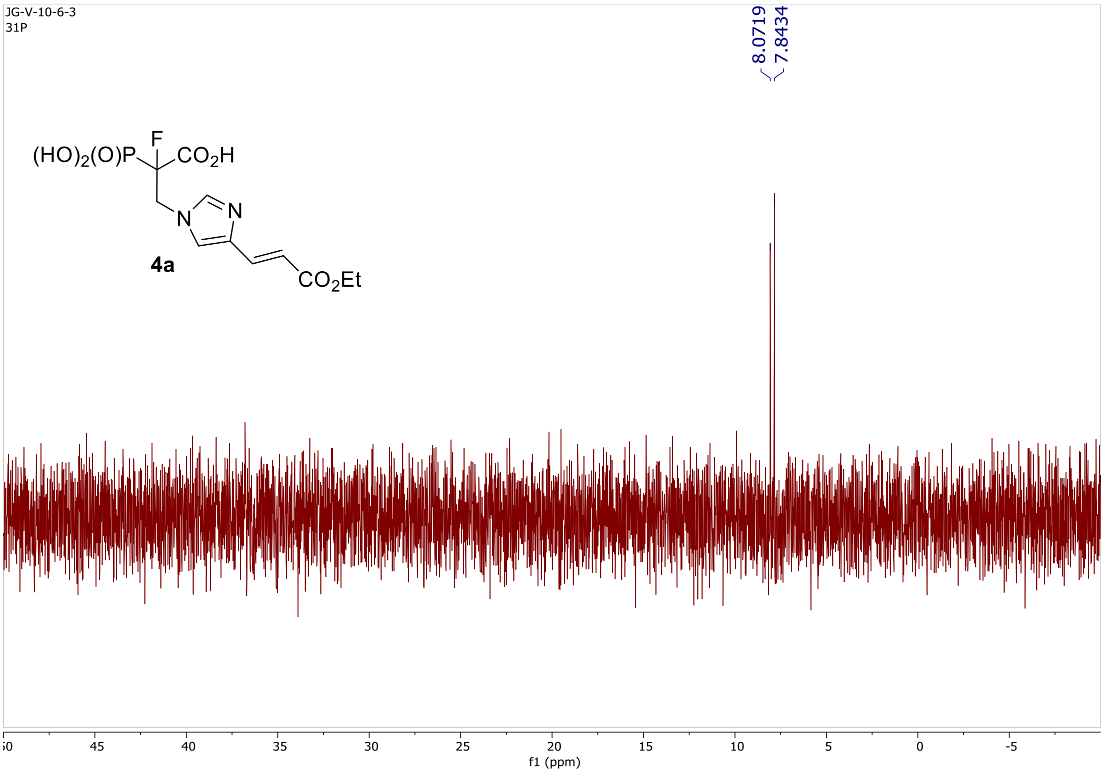

Figure S54. ^{31}P NMR (283 MHz, D_2O, pH 7) of compound **4a**.

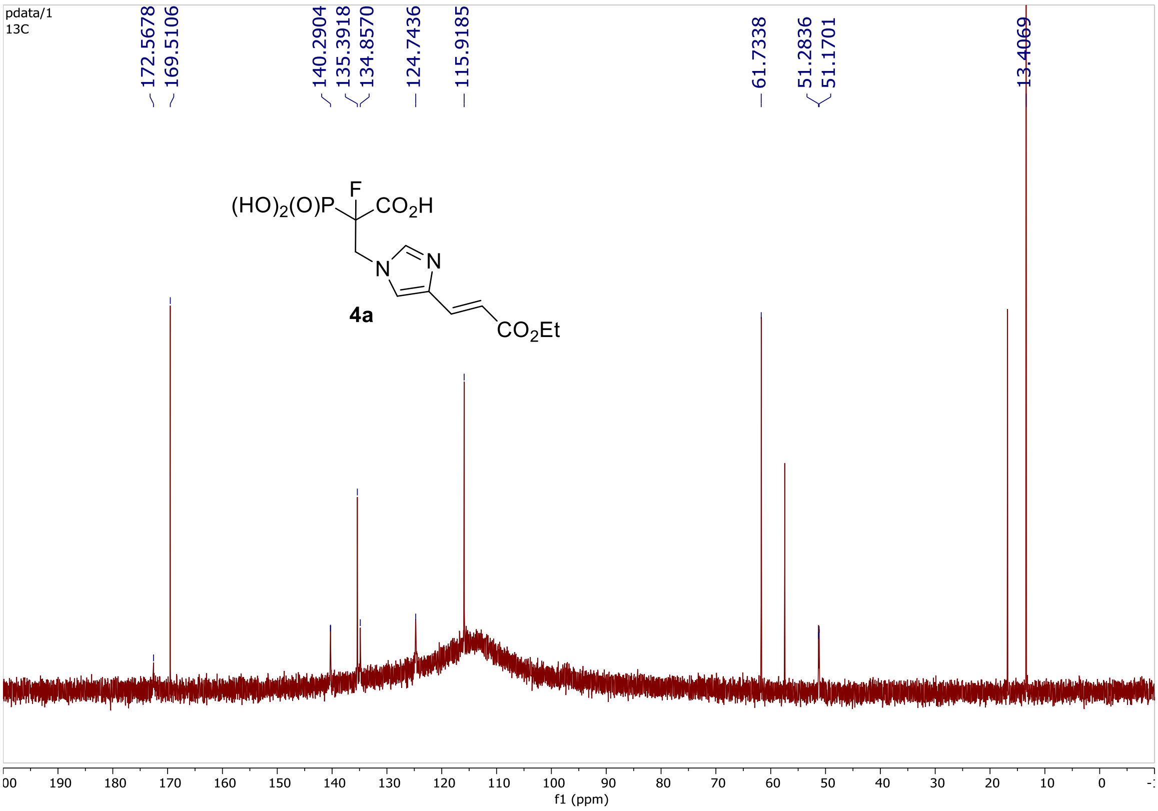

Figure S55. ^{13}C NMR (176 MHz, D_2O, pH 7) of compound **4a**.

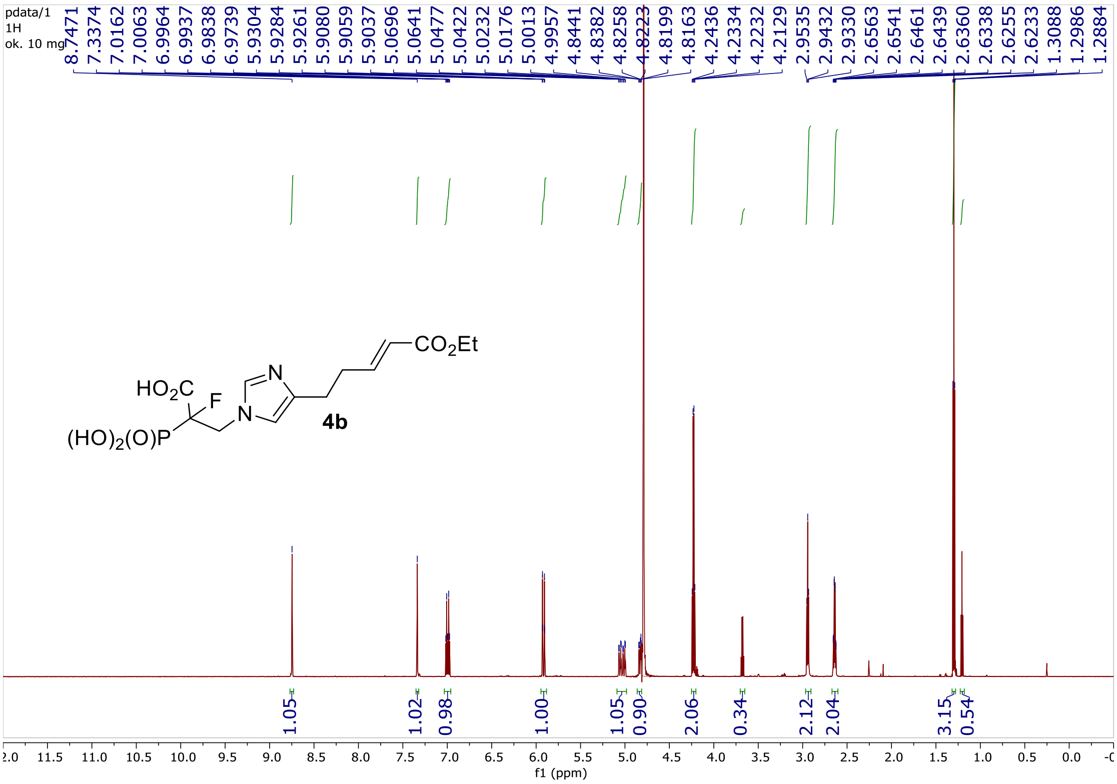

Figure S56. ^{1}H NMR (700 MHz, D_2O, pH 2) of compound **4b**.

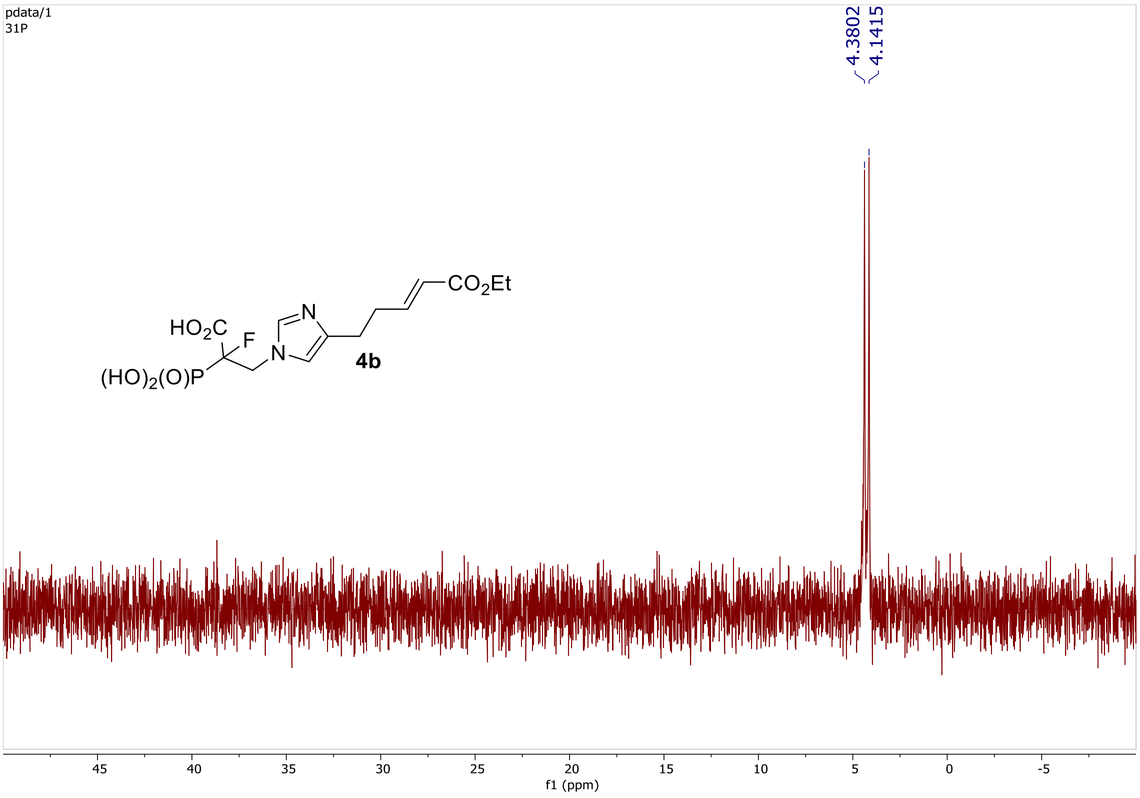

Figure S57. ^{31}P NMR (283 MHz, D_2O, pH 2) of compound **4b**.

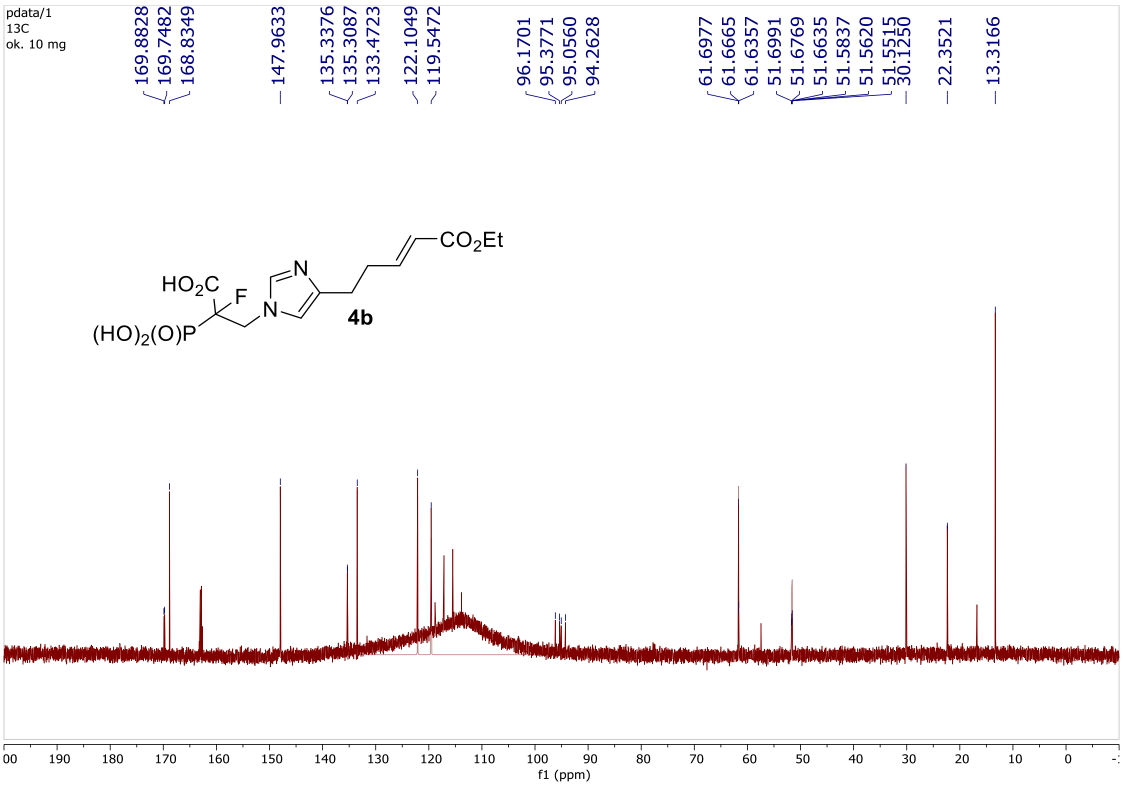

Figure S58. ^{13}C NMR (176 MHz, D_2O, pH 2) of compound **4b**.

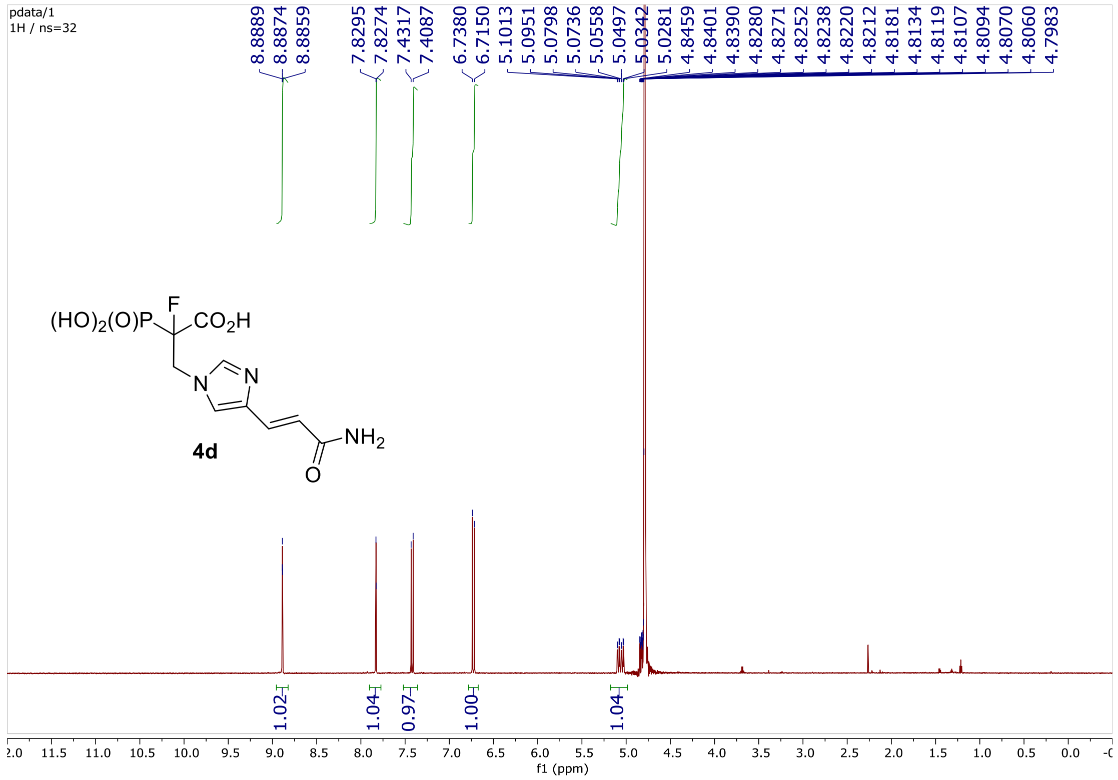

Figure S59. ^{1}H NMR (700 MHz, D_2O, pH 7) of compound **4d**.

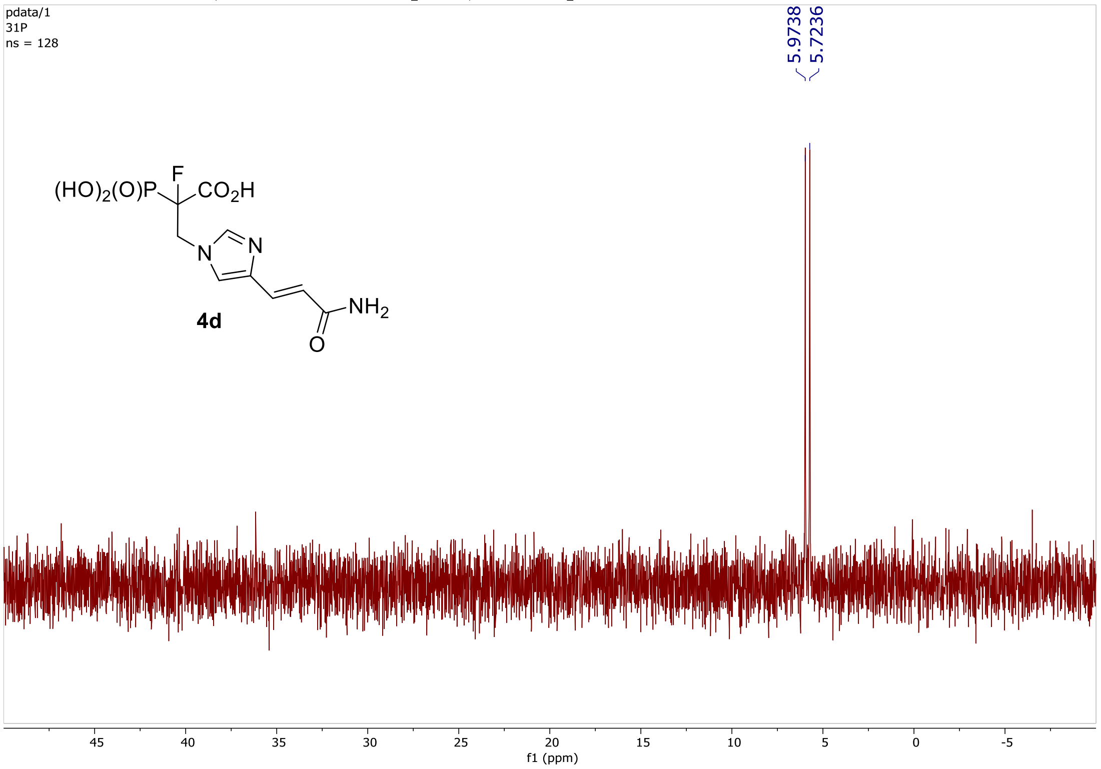

Figure S60. ^{31}P NMR (283 MHz, D_2O, pH 7) of compound **4d**.

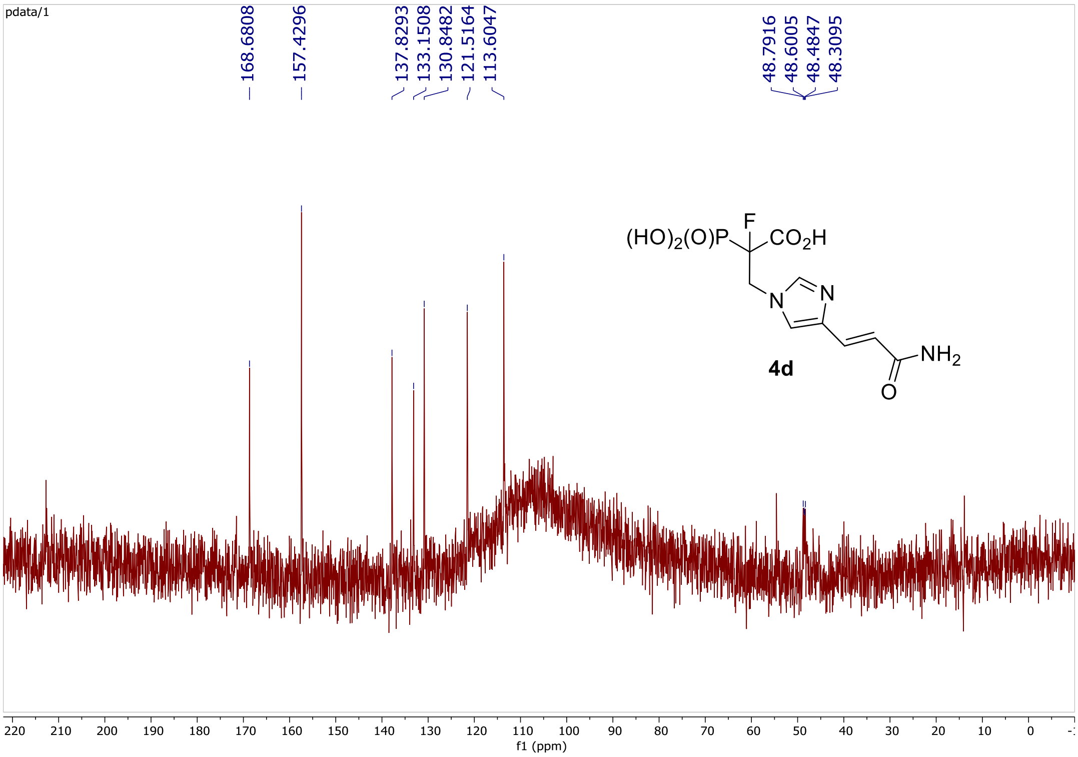

Figure S61. ^{13}C NMR (176 MHz, D_2O, pH 7) of compound **4d**.

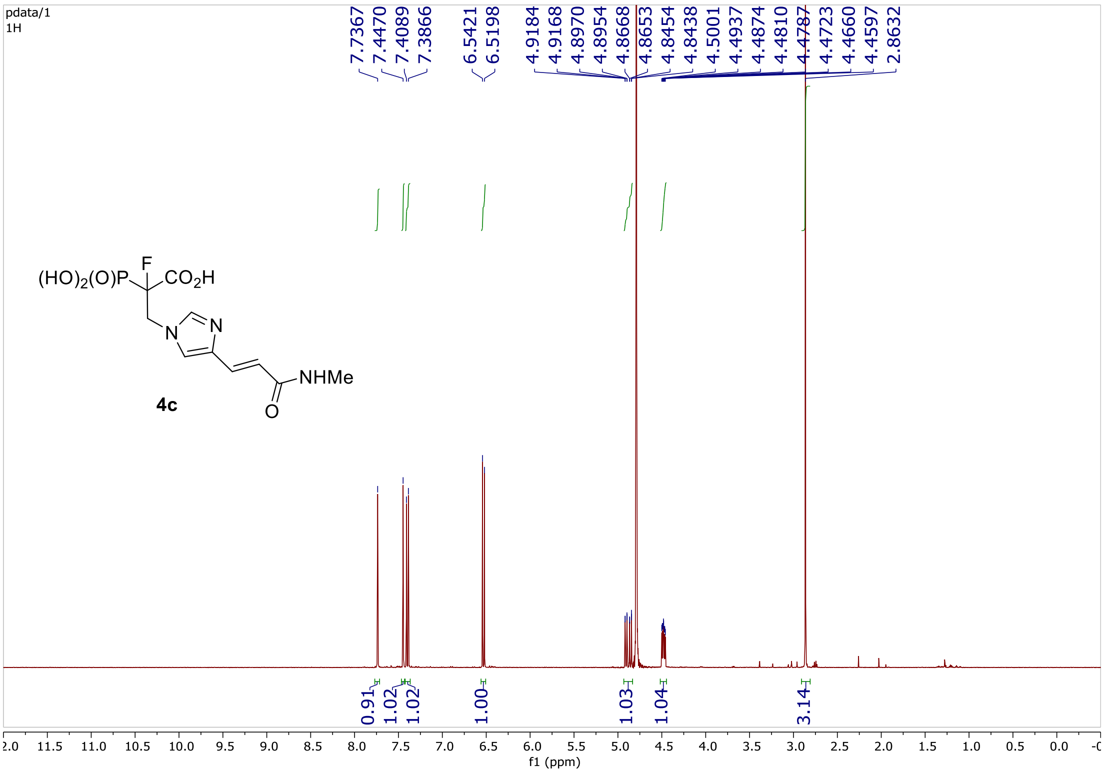

Figure S62. ^{1}H NMR (700 MHz, D_2O, pH 7) of compound **4c**.

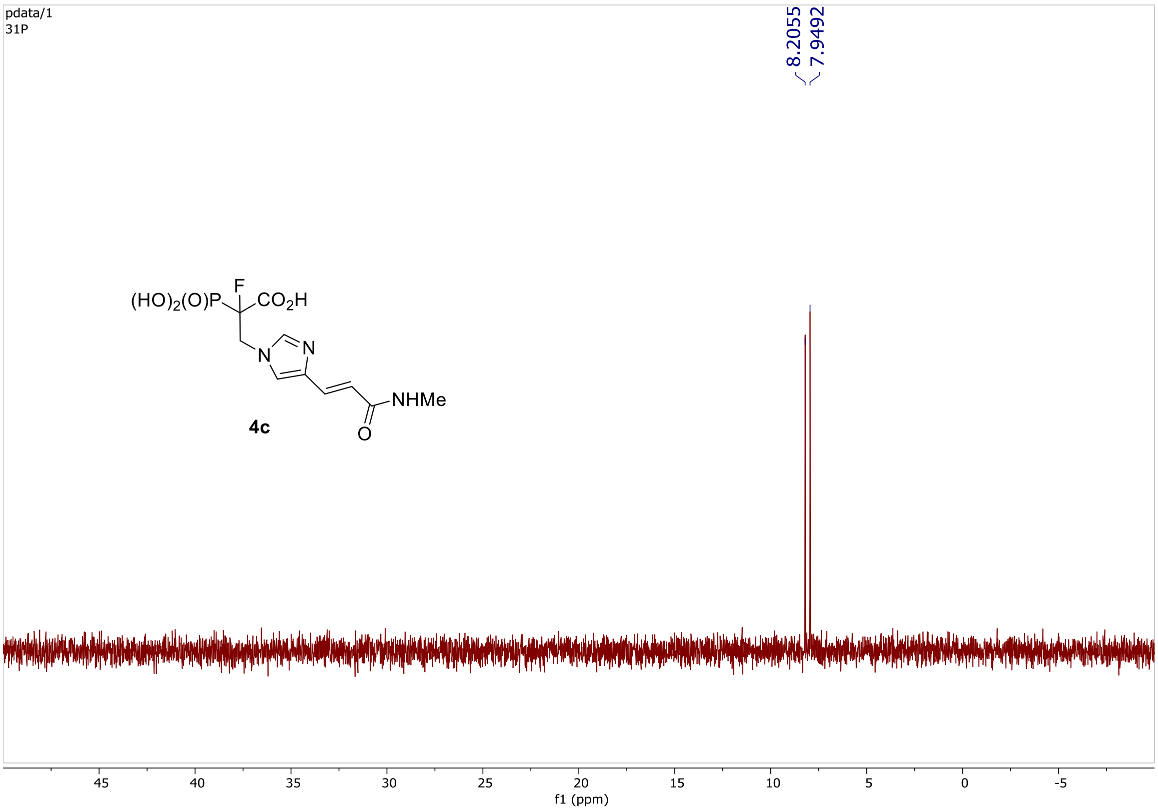

Figure S63. ^{31}P NMR (283 MHz, D_2O, pH 7) of compound **4c**.

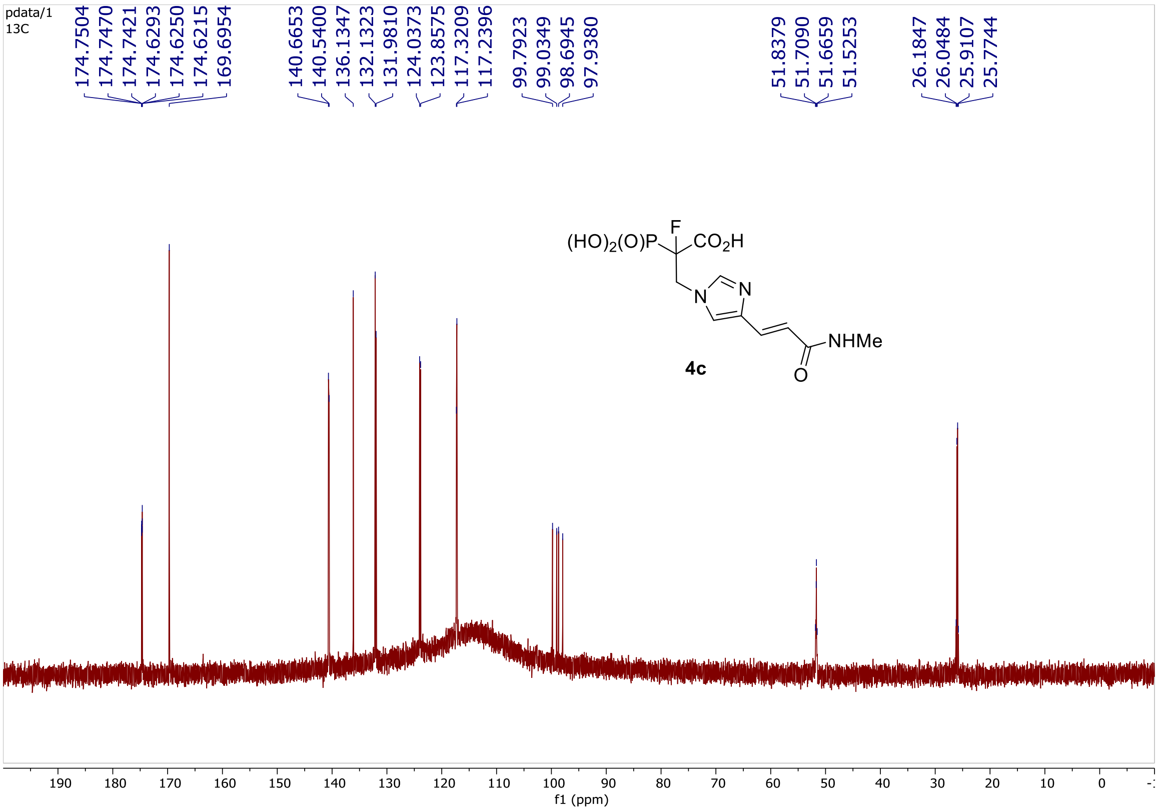

Figure S64. ^{13}C NMR (176 MHz, D_2O, pH 7) of compound **4c**.

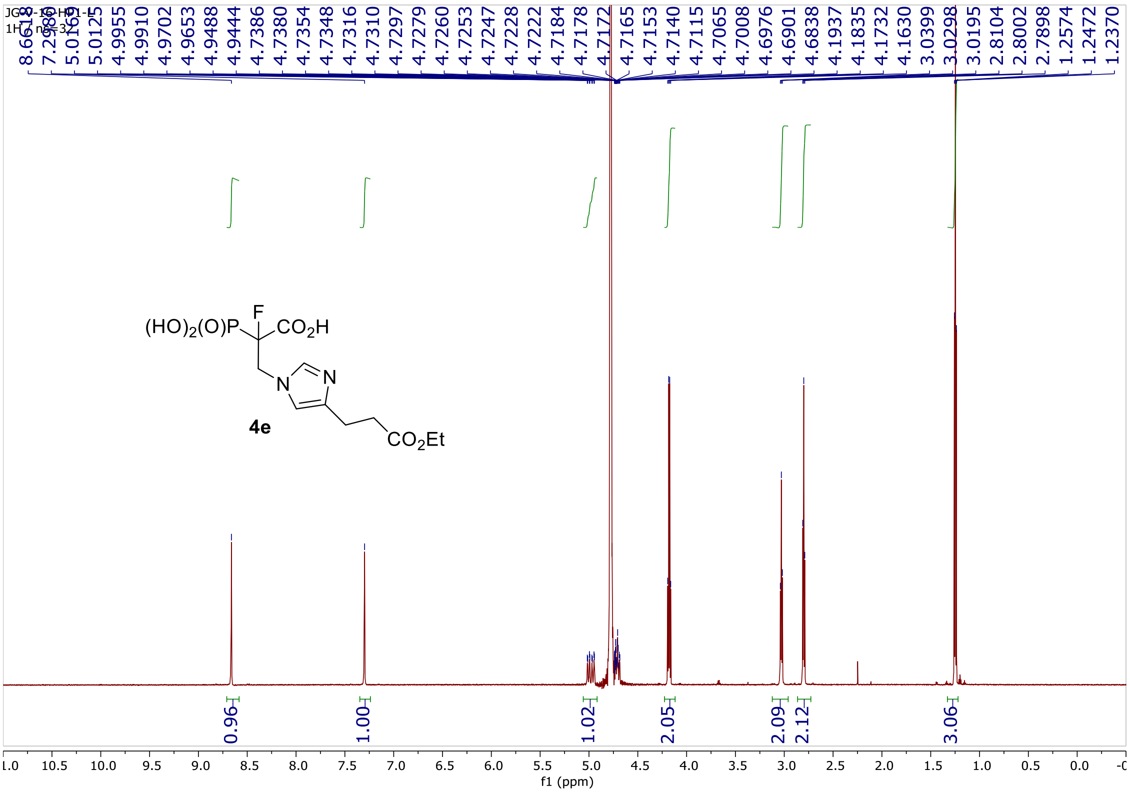

Figure S65. ^{1}H NMR (700 MHz, D_2O, pH 7) of compound **4e**.

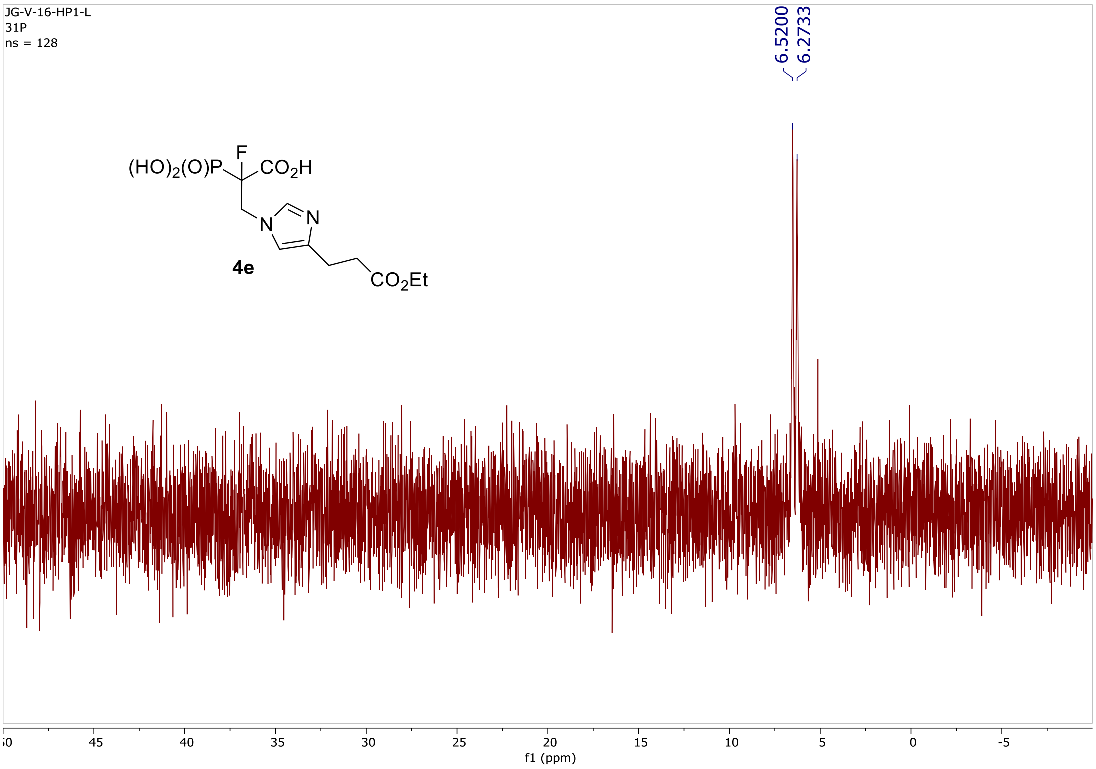

Figure S66. ^{31}P NMR (283 MHz, D_2O, pH 7) of compound **4e**.

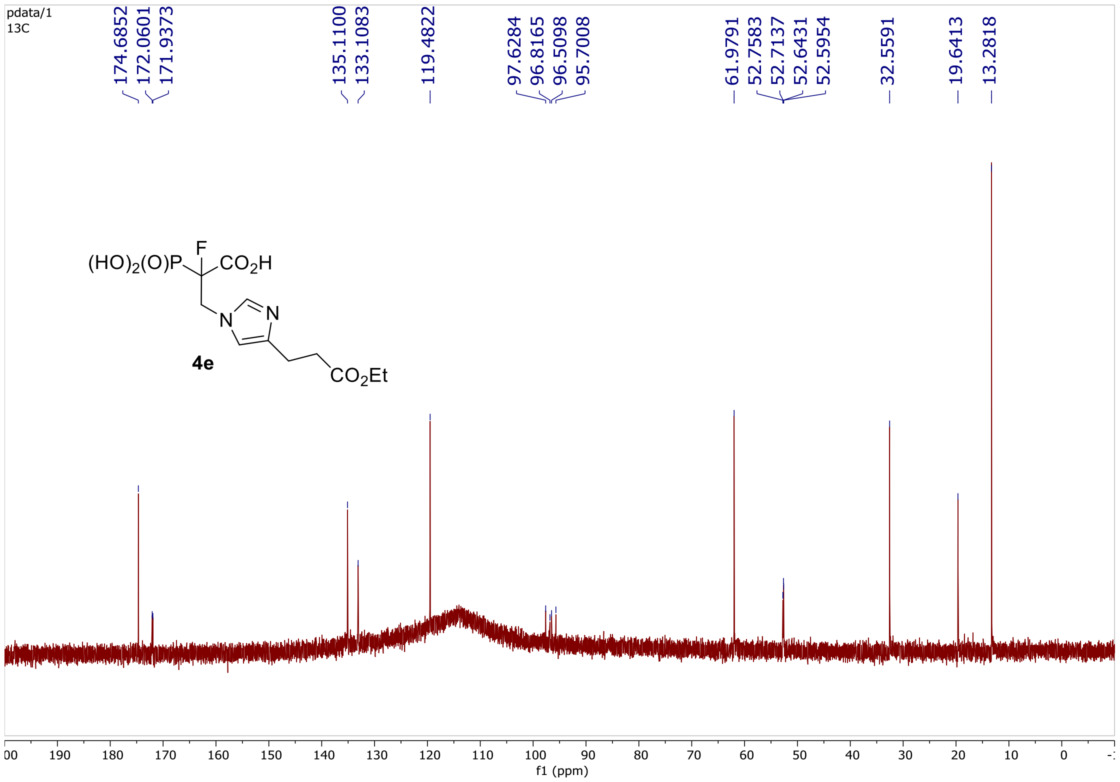

Figure S67. ^{13}C NMR (176 MHz, D_2O, pH 7) of compound **4e.**

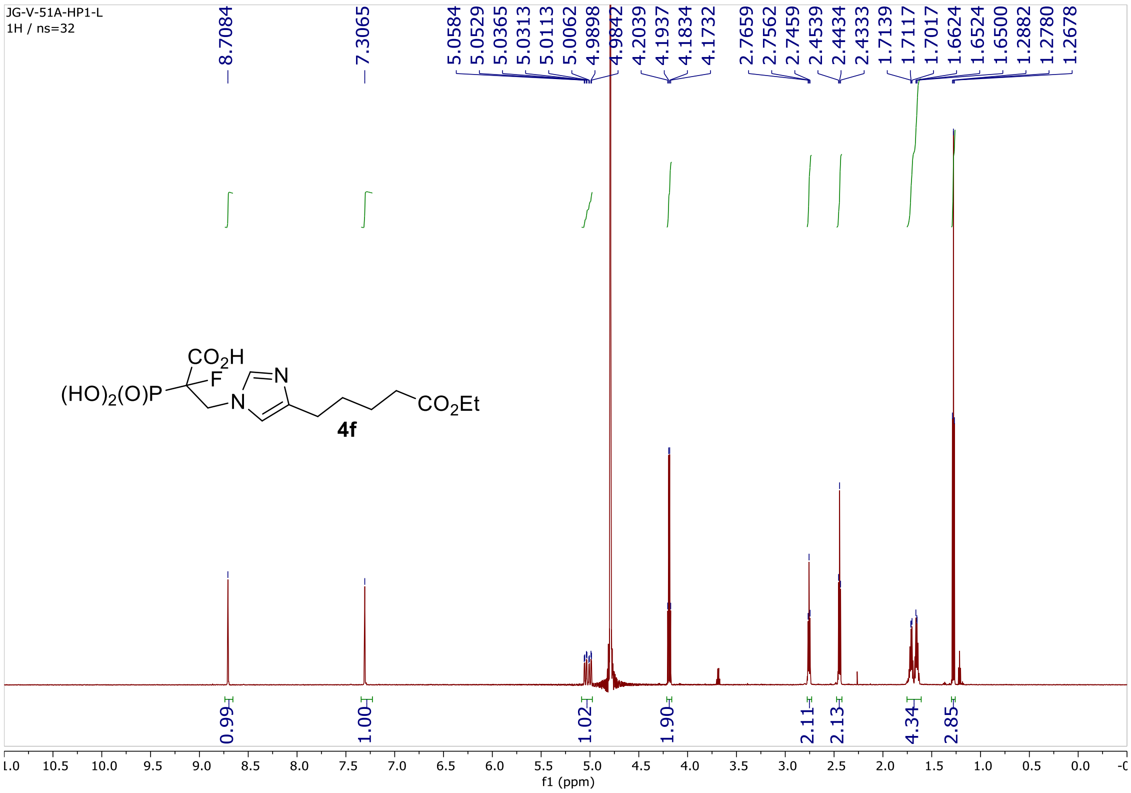

Figure S68. ^{1}H NMR (700 MHz, D_2O, pH 3) of compound **4f**.

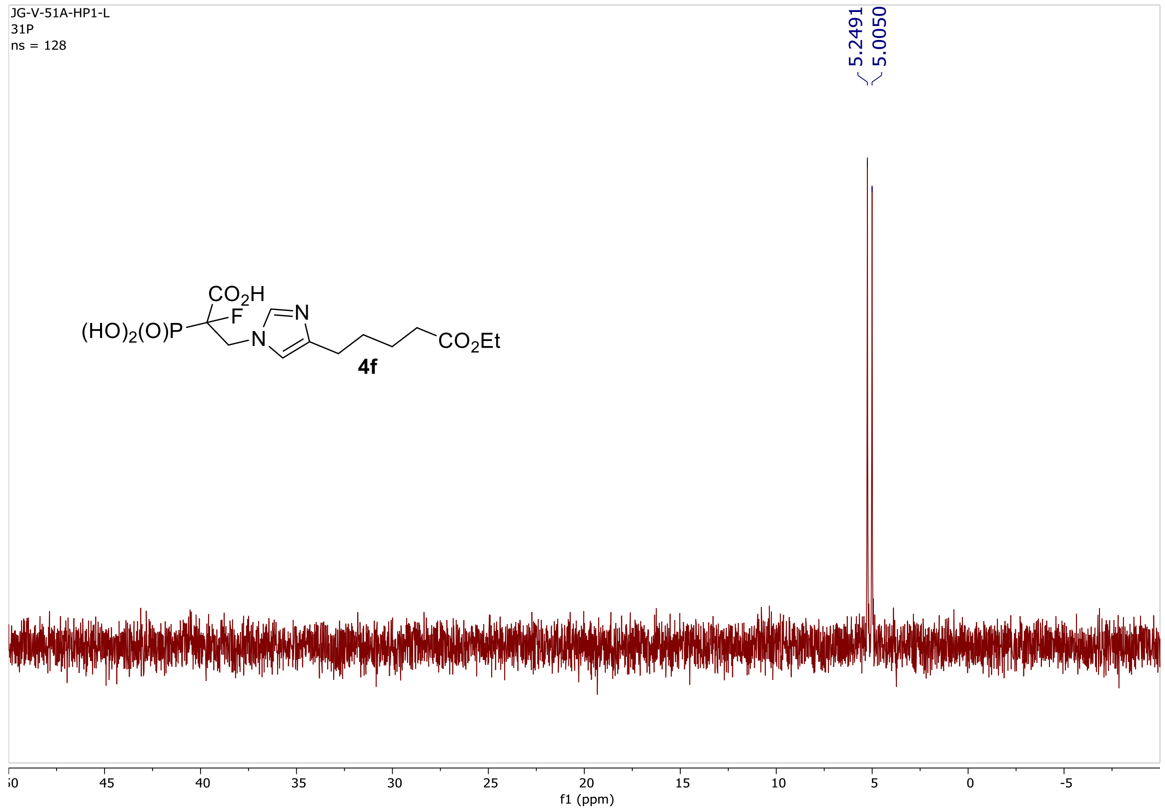

Figure S69. ^{31}P NMR (283 MHz, D_2O, pH 3) of compound **4f**.

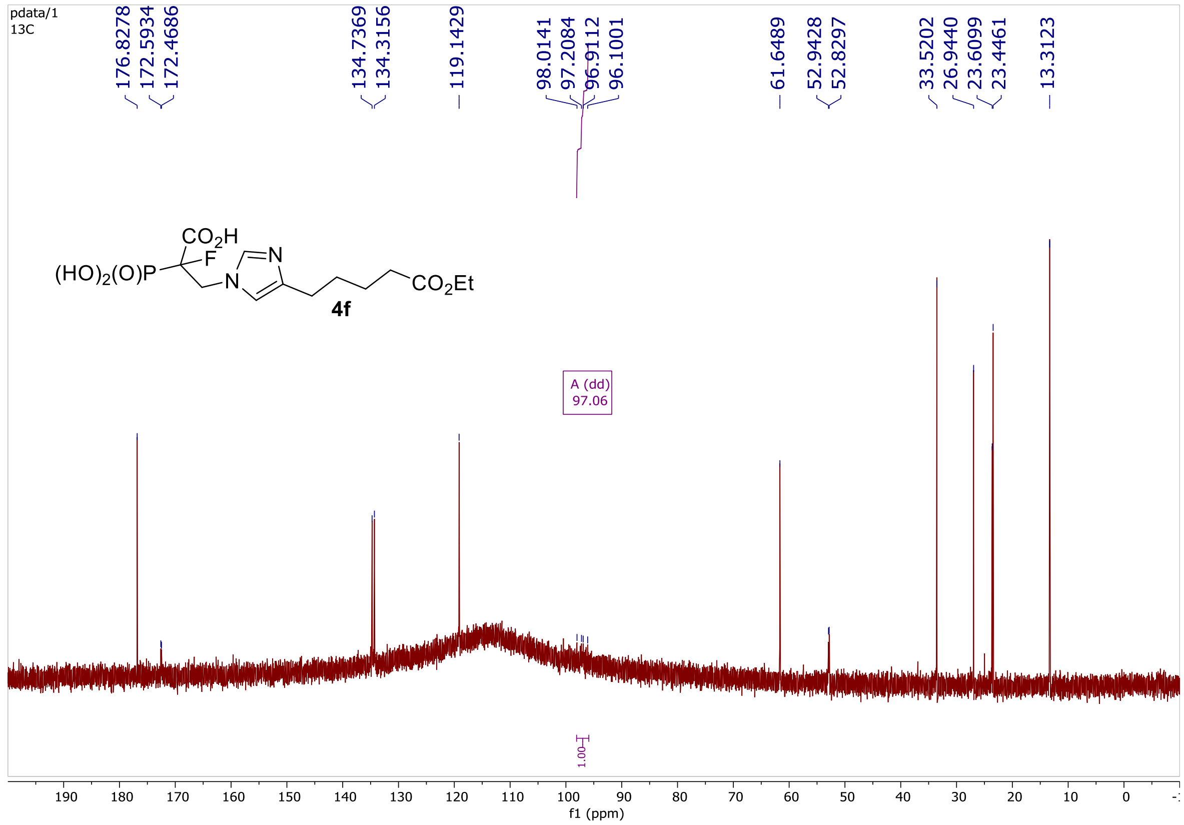

Figure S70. ^{13}C NMR (176 MHz, D_2O, pH 3) of compound **4f**.

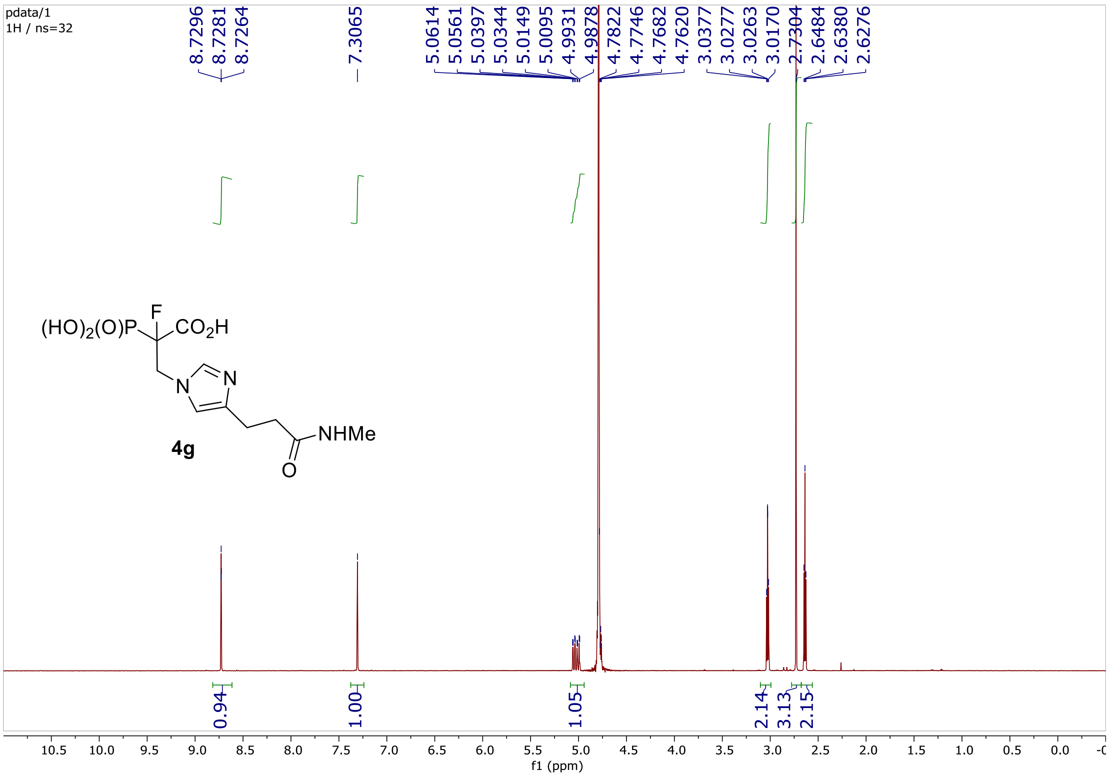

Figure S71. ^{1}H NMR (700 MHz, D_2O, pH 7) of compound **4g.**

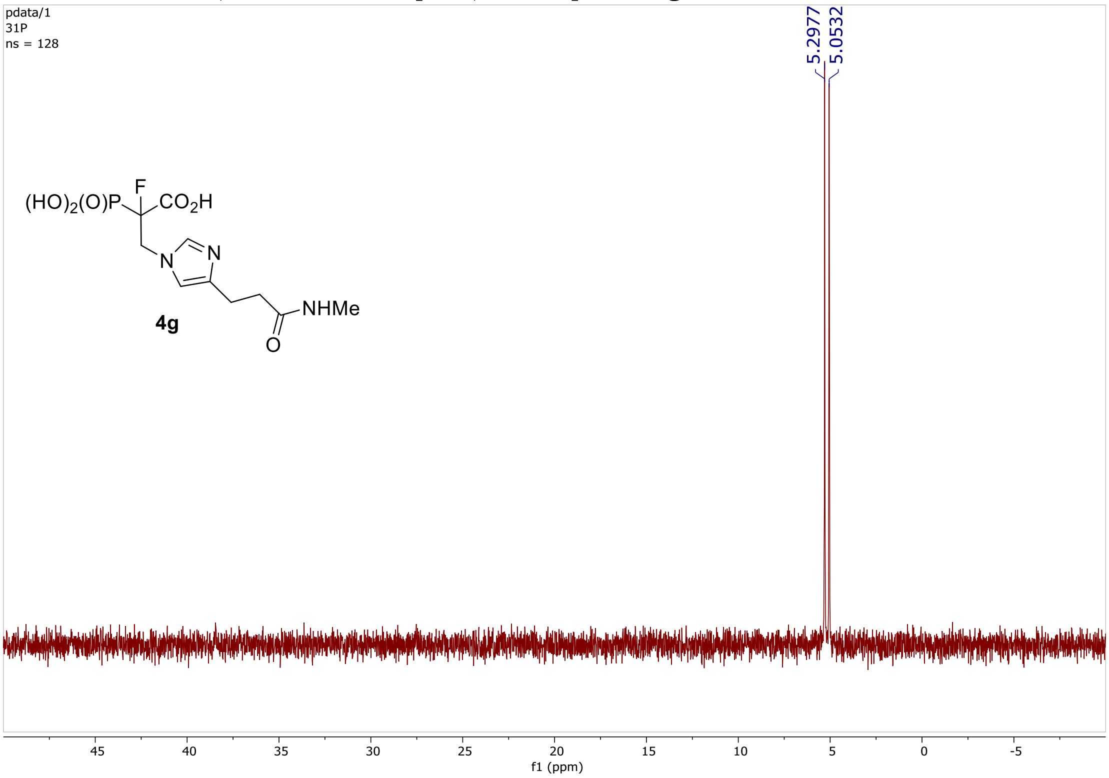

Figure S72. ^{31}P NMR (283 MHz, D_2O, pH 7) of compound **4g**.

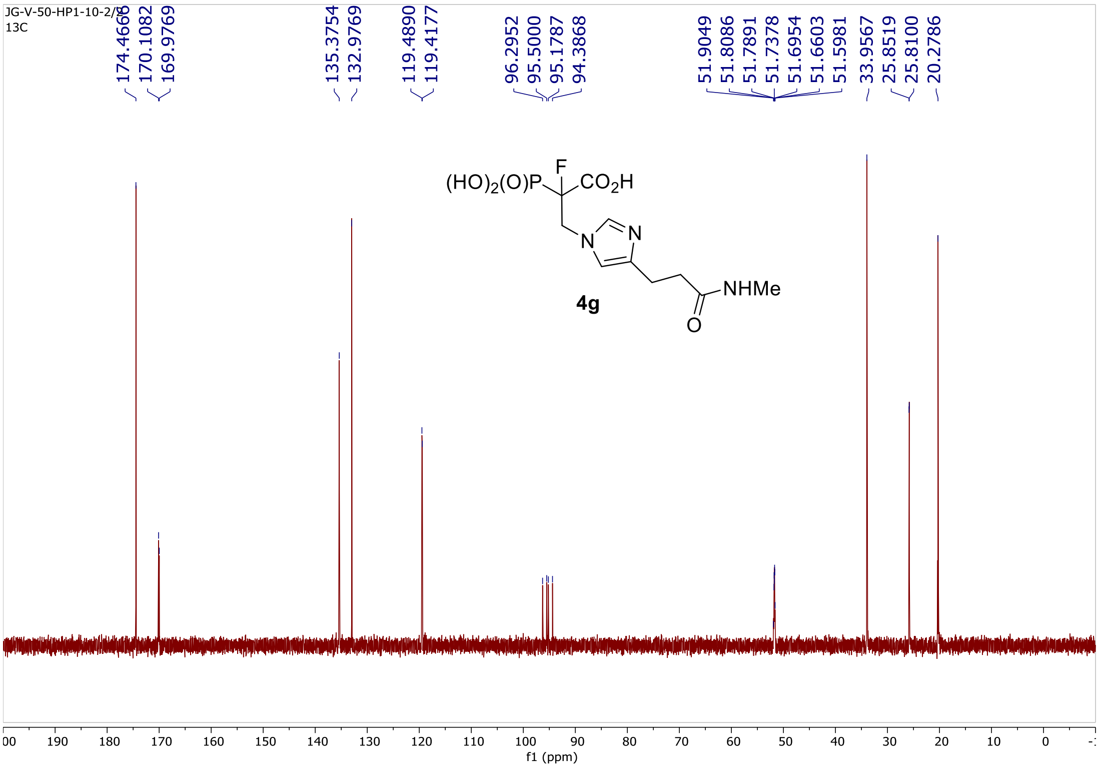

Figure S73. ^{13}C NMR (176 MHz, D_2O, pH 7) of compound **4g**.

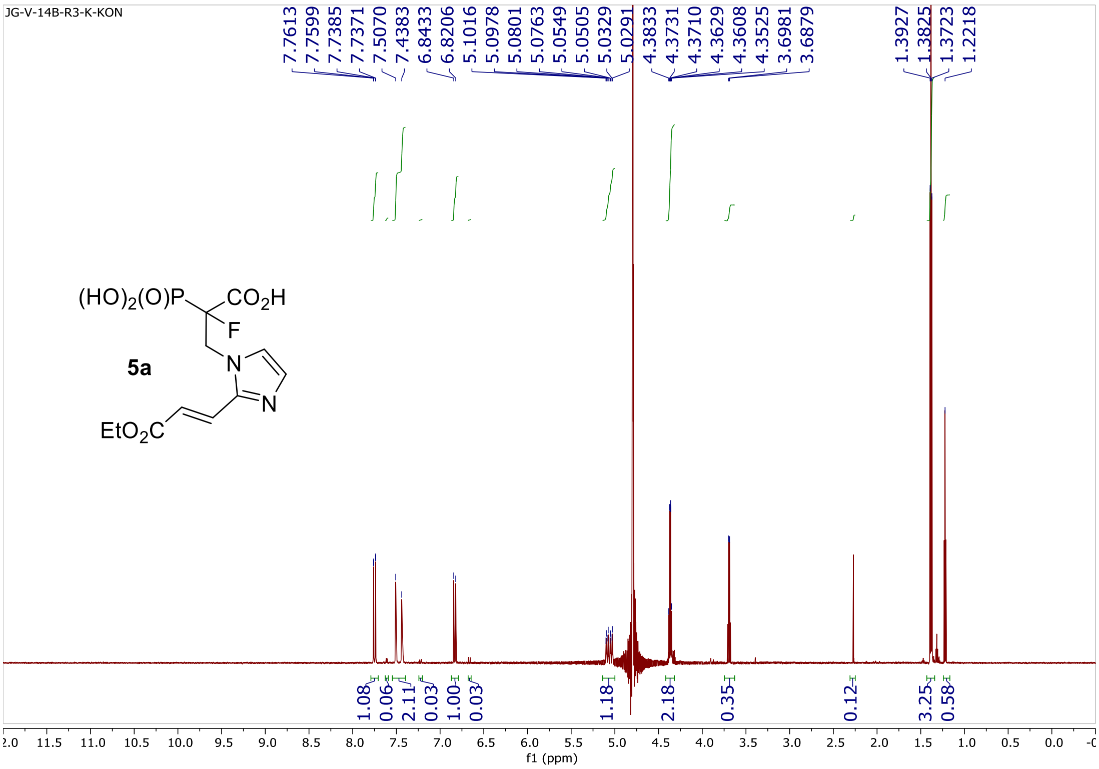

Figure S74. ^{1}H NMR (700 MHz, D_2O, pH 7) of compound **5a**.

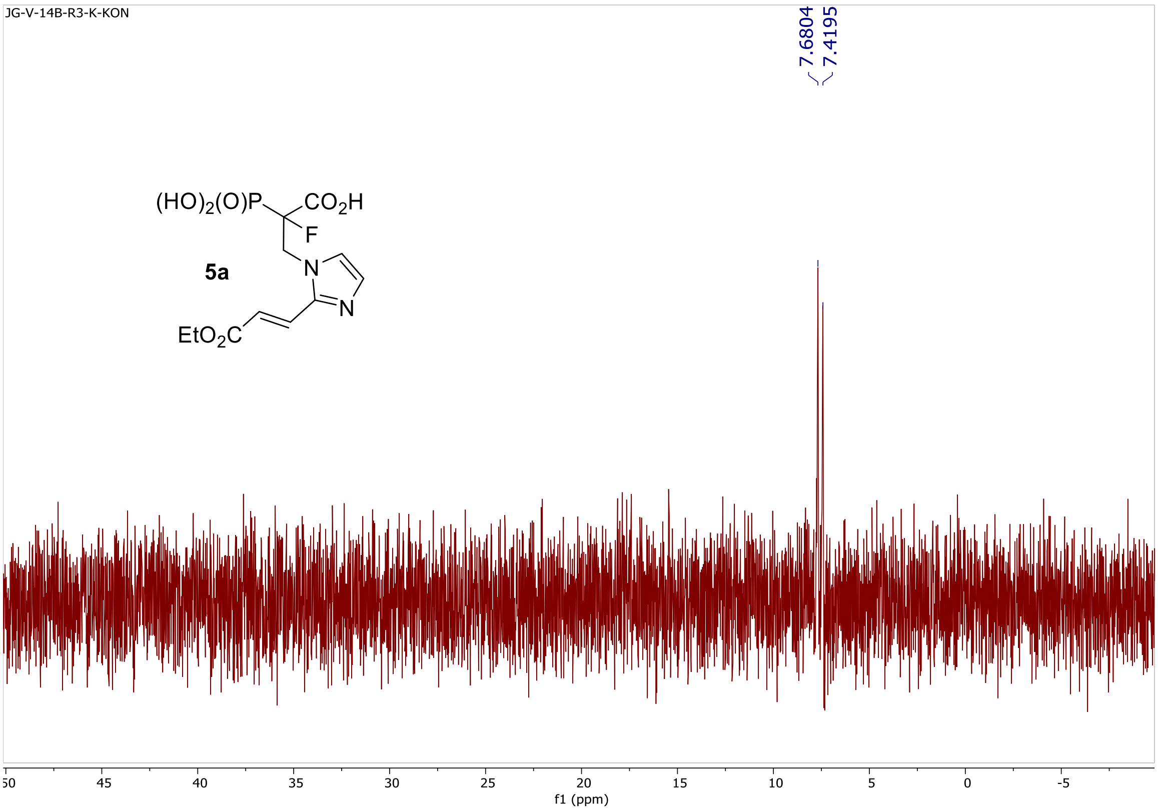

Figure S75. ^{31}P NMR (283 MHz, D_2O, pH 7) of compound **5a**.

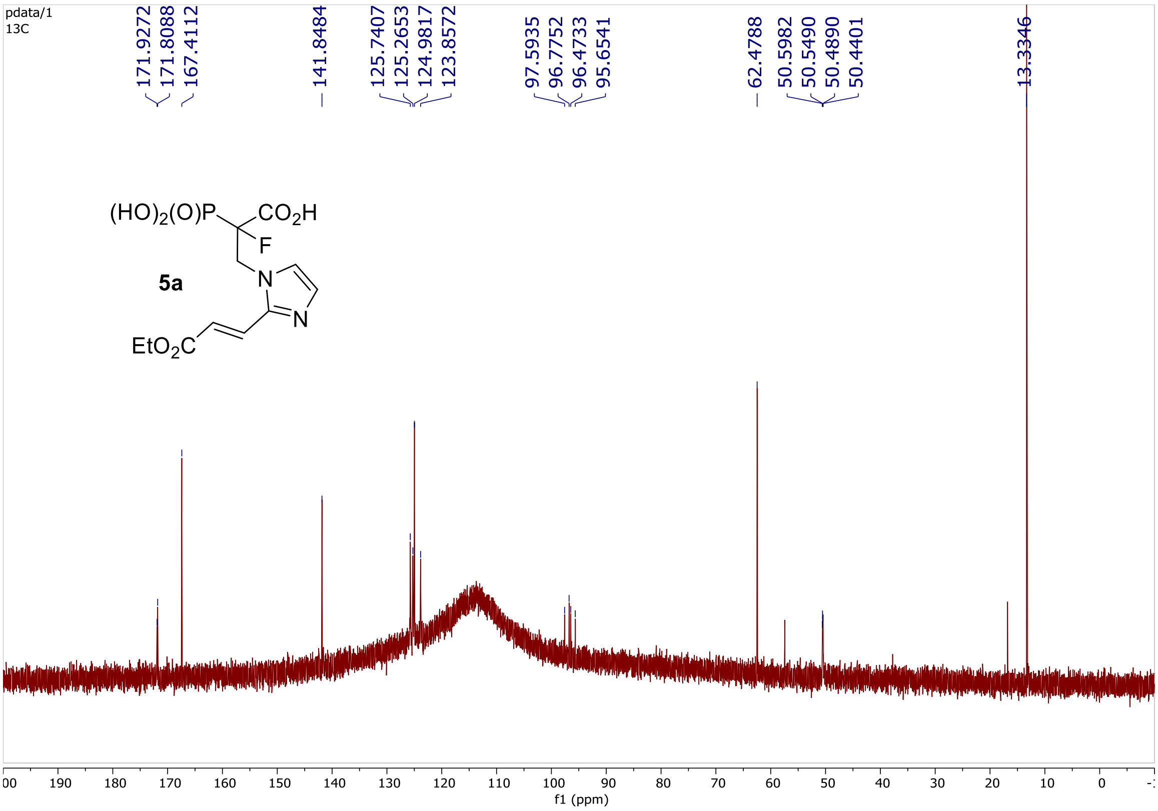

Figure S76. ^{13}C NMR (176 MHz, D_2O, pH 7) of compound **5a**.

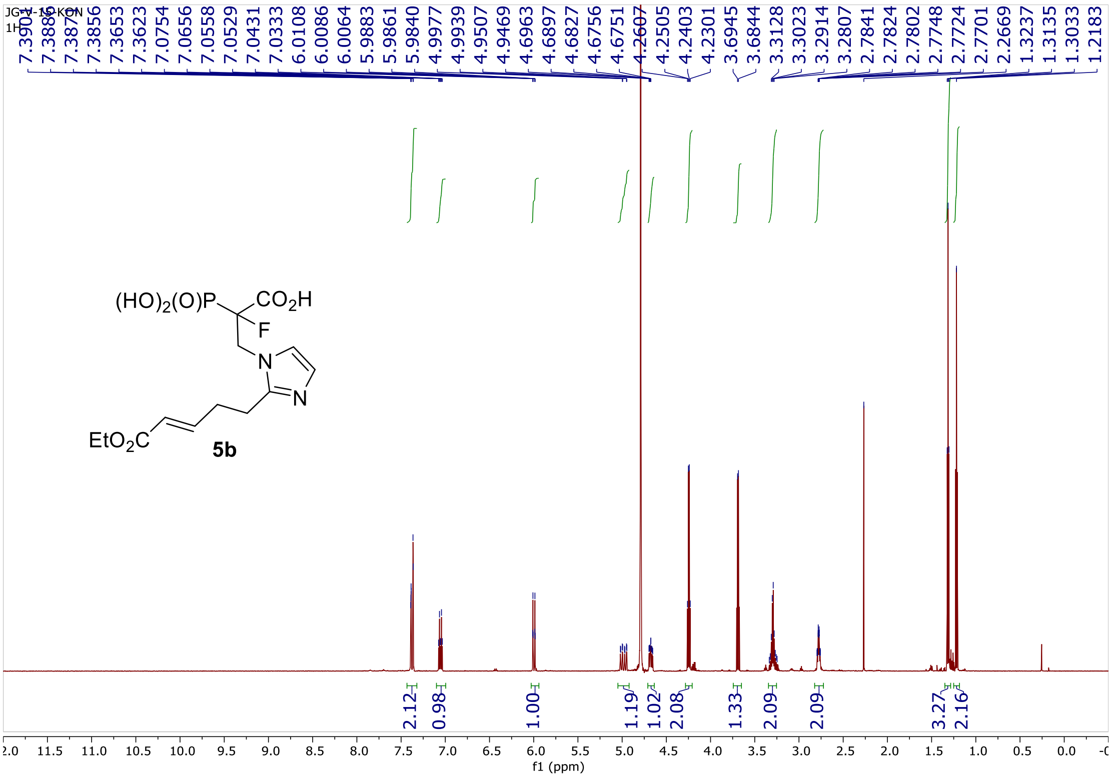

Figure S77. ^{1}H NMR (700 MHz, D_2O, pH 7) of compound **5b**.

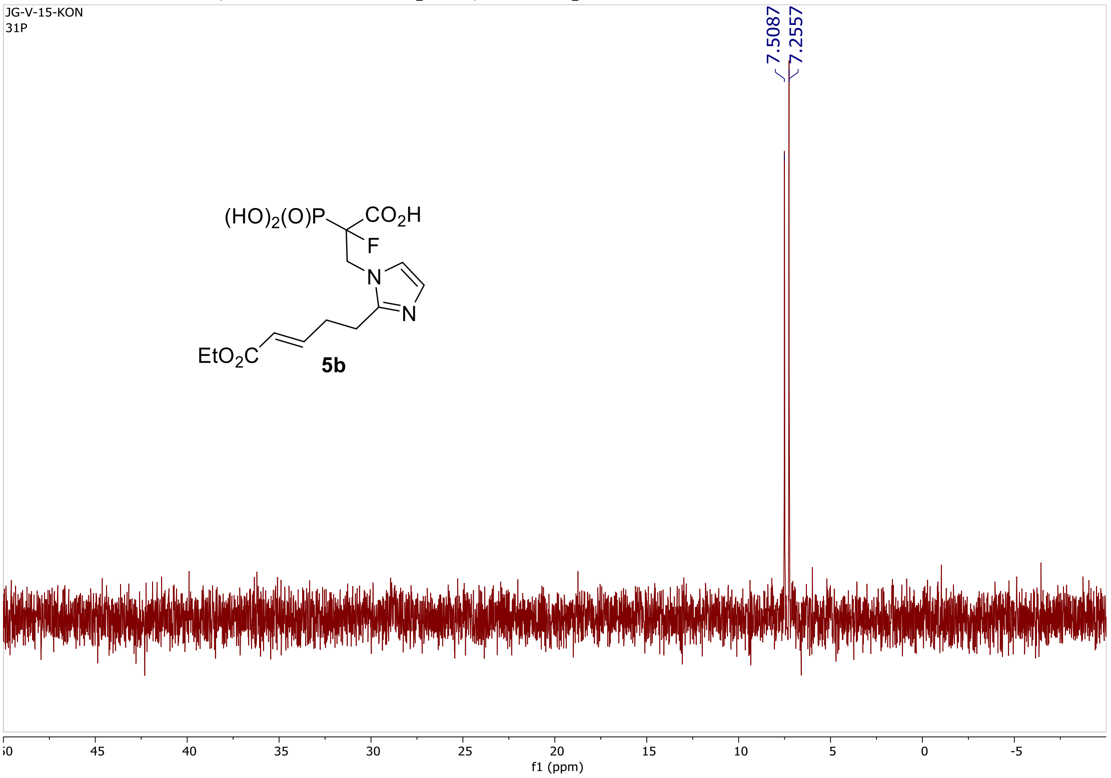

Figure S78. ^{31}P NMR (283 MHz, D_2O, pH 7) of compound **5b**.

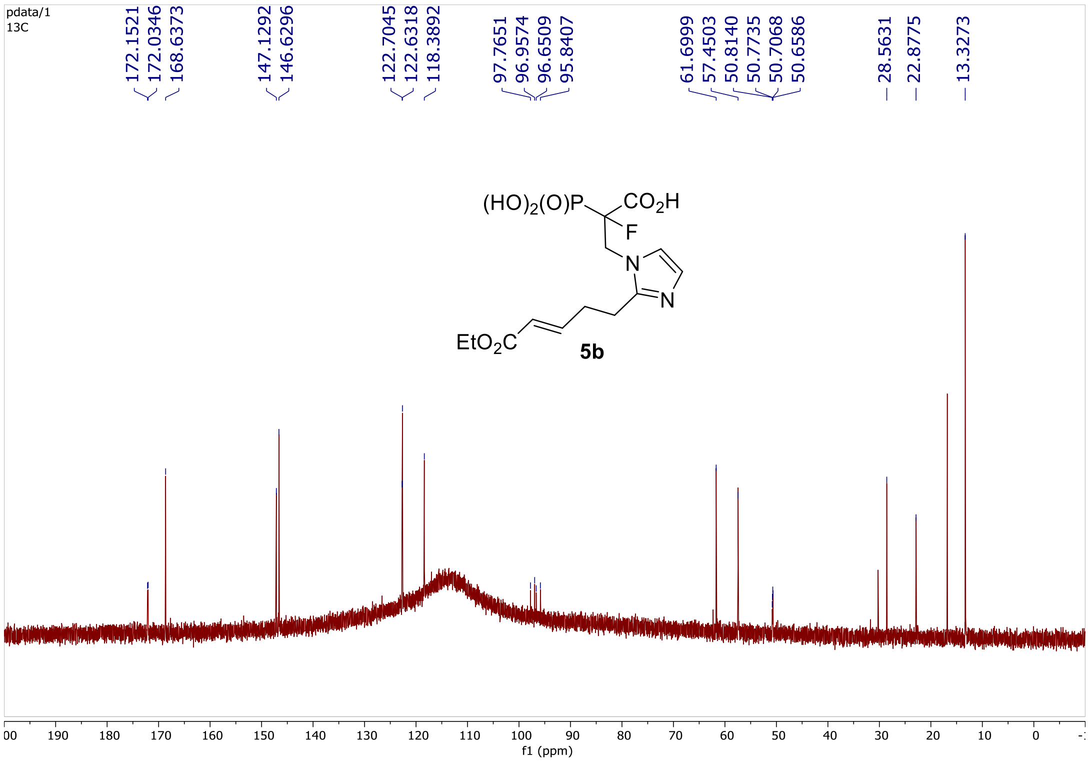

Figure S79. ^{13}C NMR (176 MHz, D_2O, pH 7) of compound **5b**.

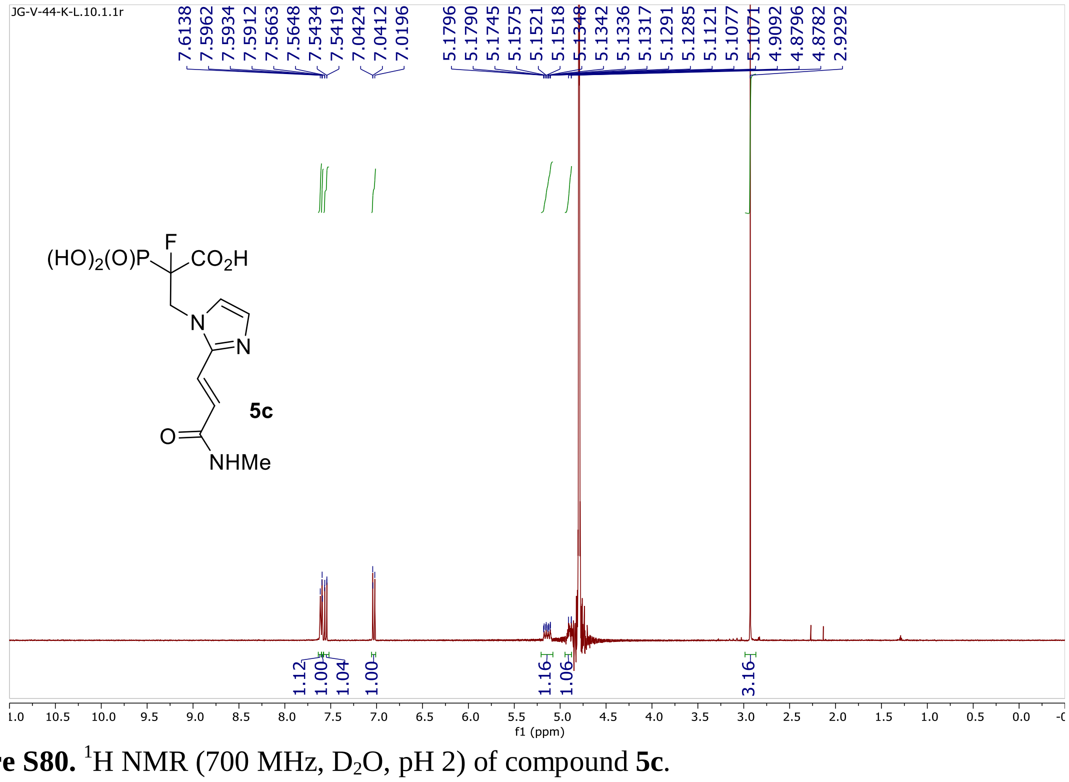

Figure S80. ^{1}H NMR (700 MHz, D_2O, pH 2) of compound **5c**.

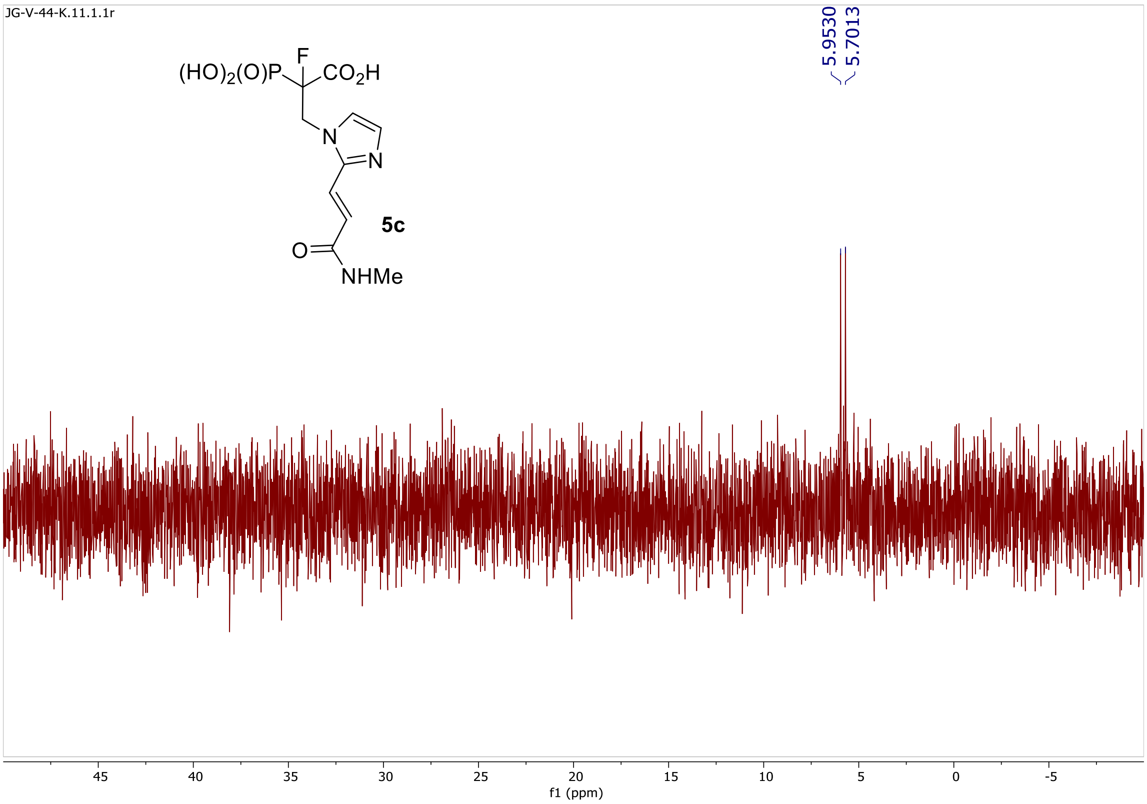

Figure S81. ^{31}P NMR (283 MHz, D_2O, pH 7) of compound **5c.**

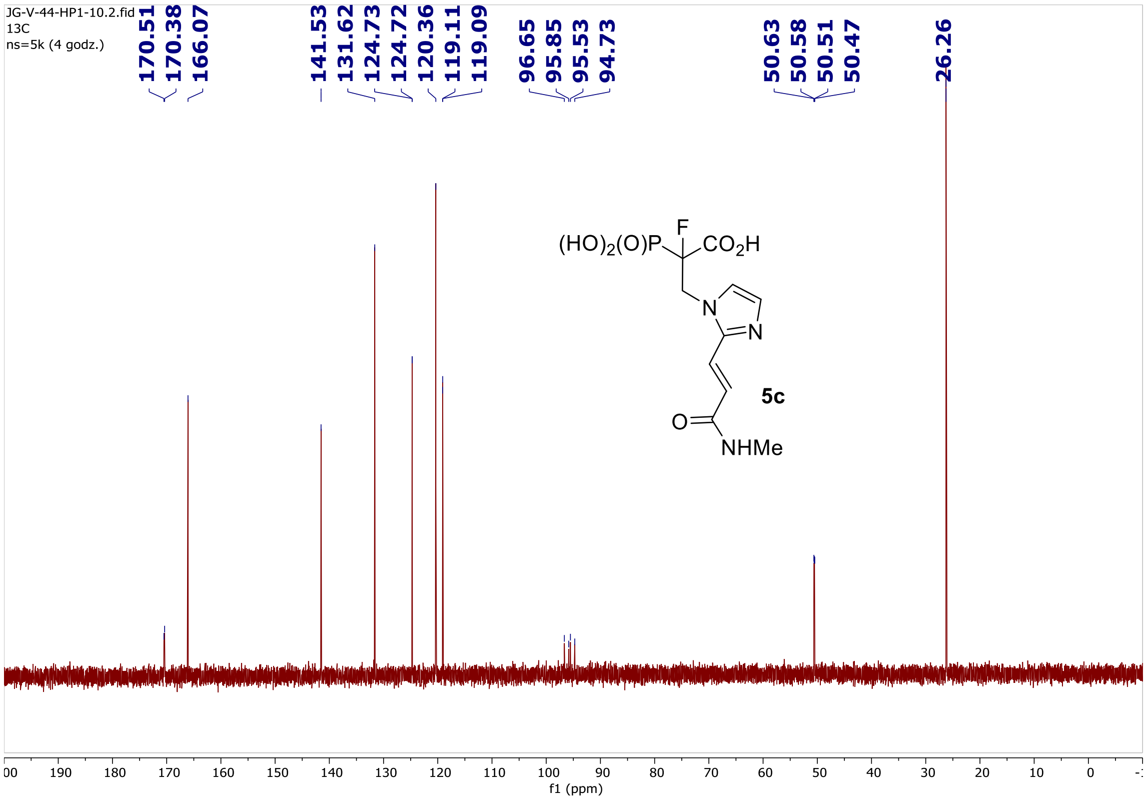

Figure S82. ^{13}C NMR (176 MHz, D_2O, pH 7) of compound **5c**.

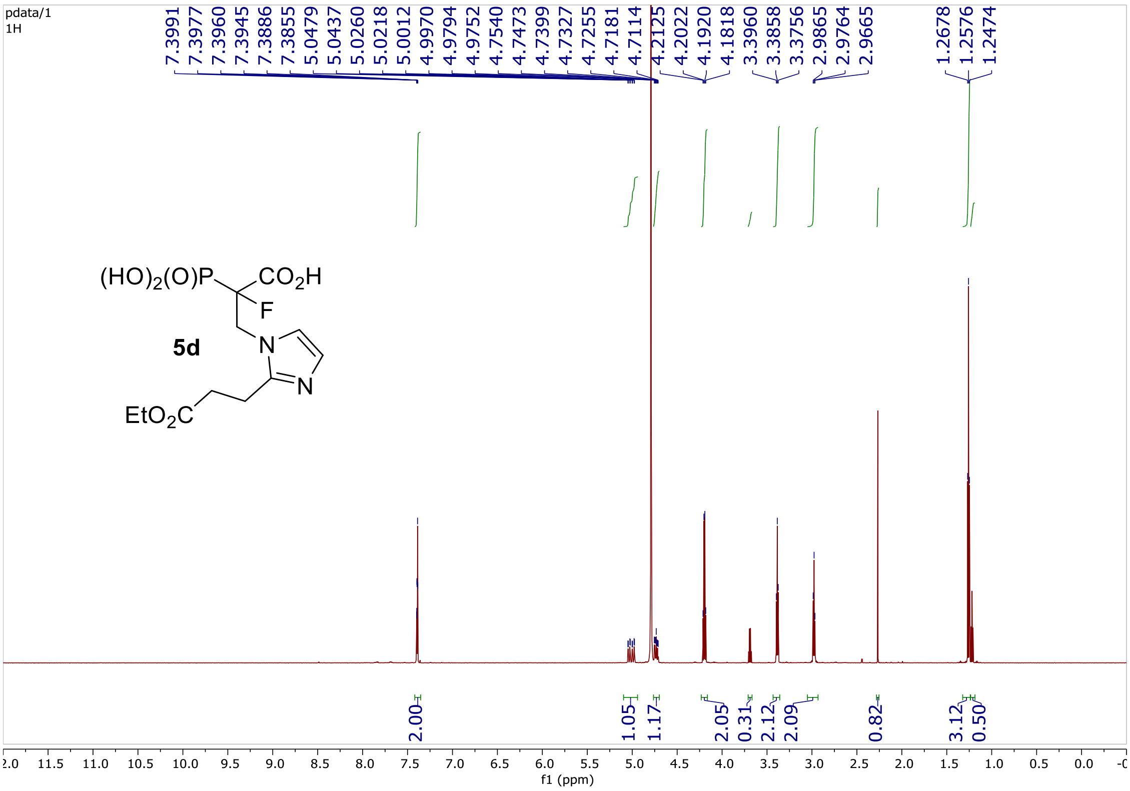

Figure S83. ^{1}H NMR (700 MHz, D_2O, pH 7) of compound **5d**.

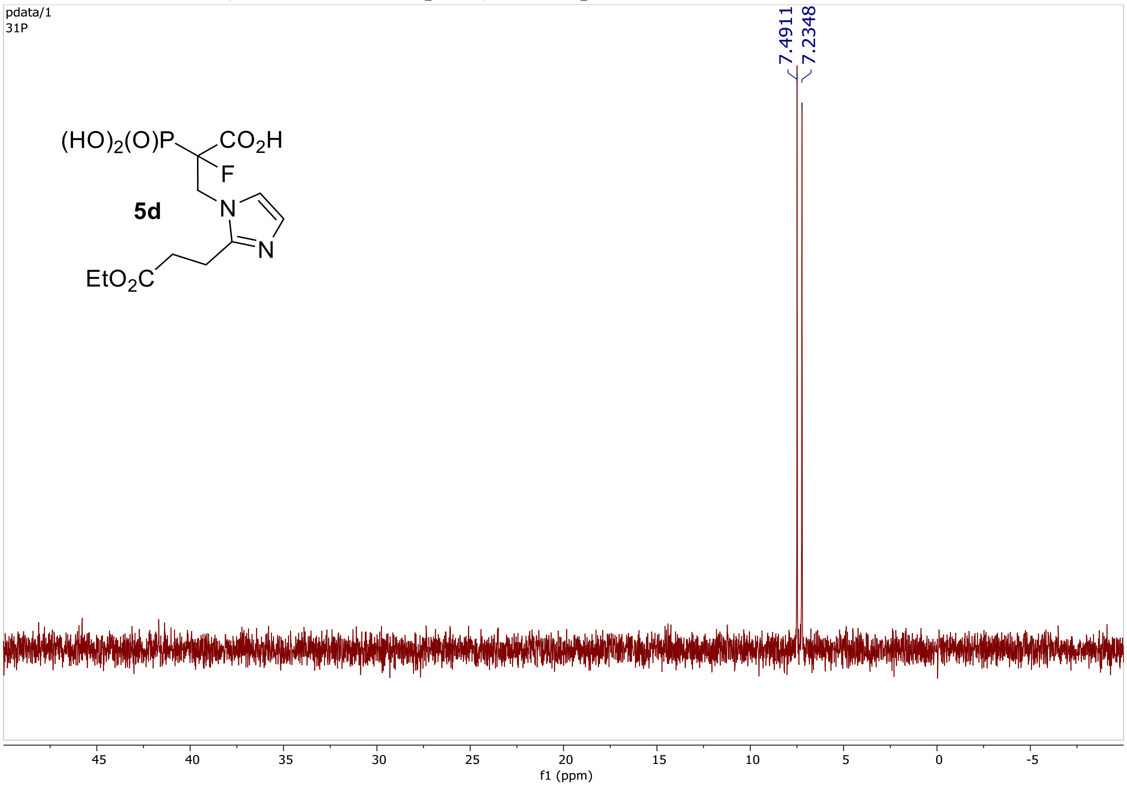

Figure S84. ^{31}P NMR (283 MHz, D_2O, pH 7) of compound **5d**.

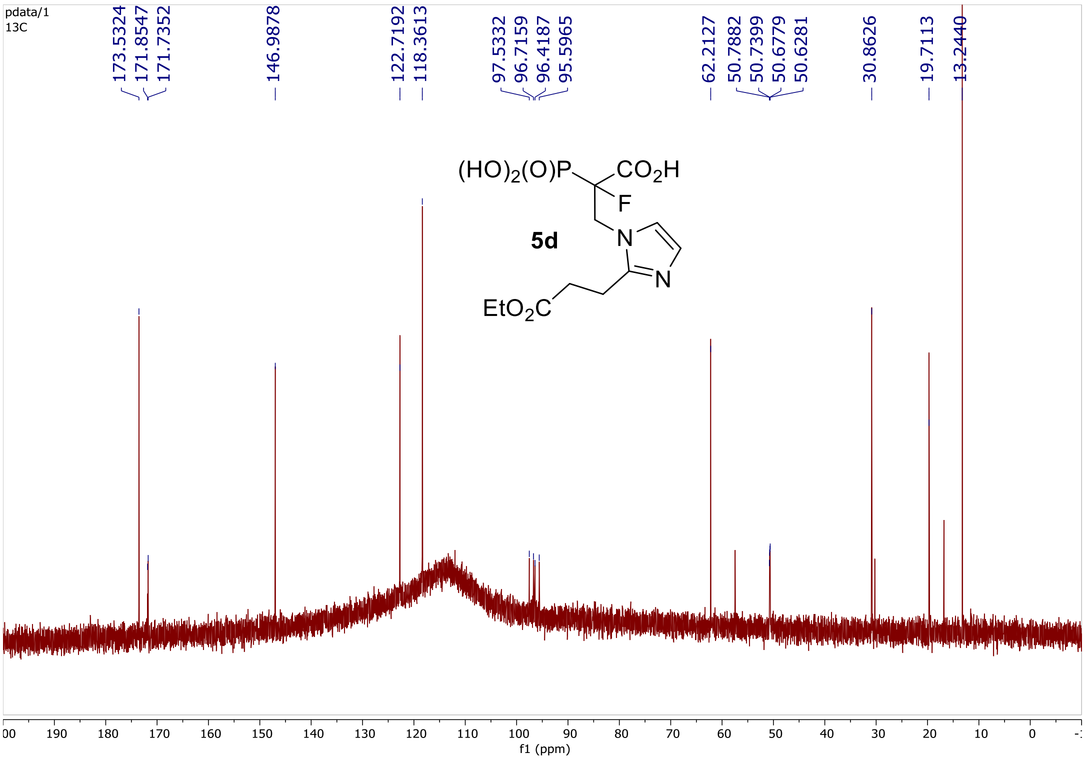

Figure S85. ^{13}C NMR (176 MHz, D_2O, pH 7) of compound **5d**.

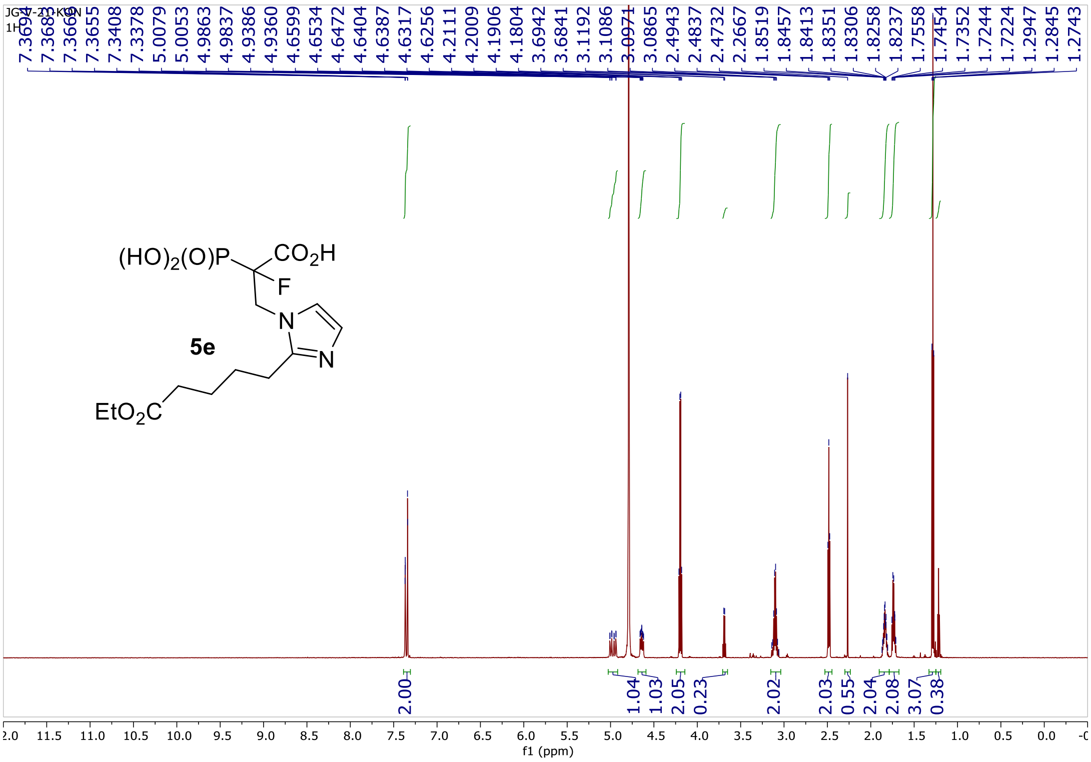

Figure S86. ^{1}H NMR (700 MHz, D_2O, pH 7) of compound **5e**.

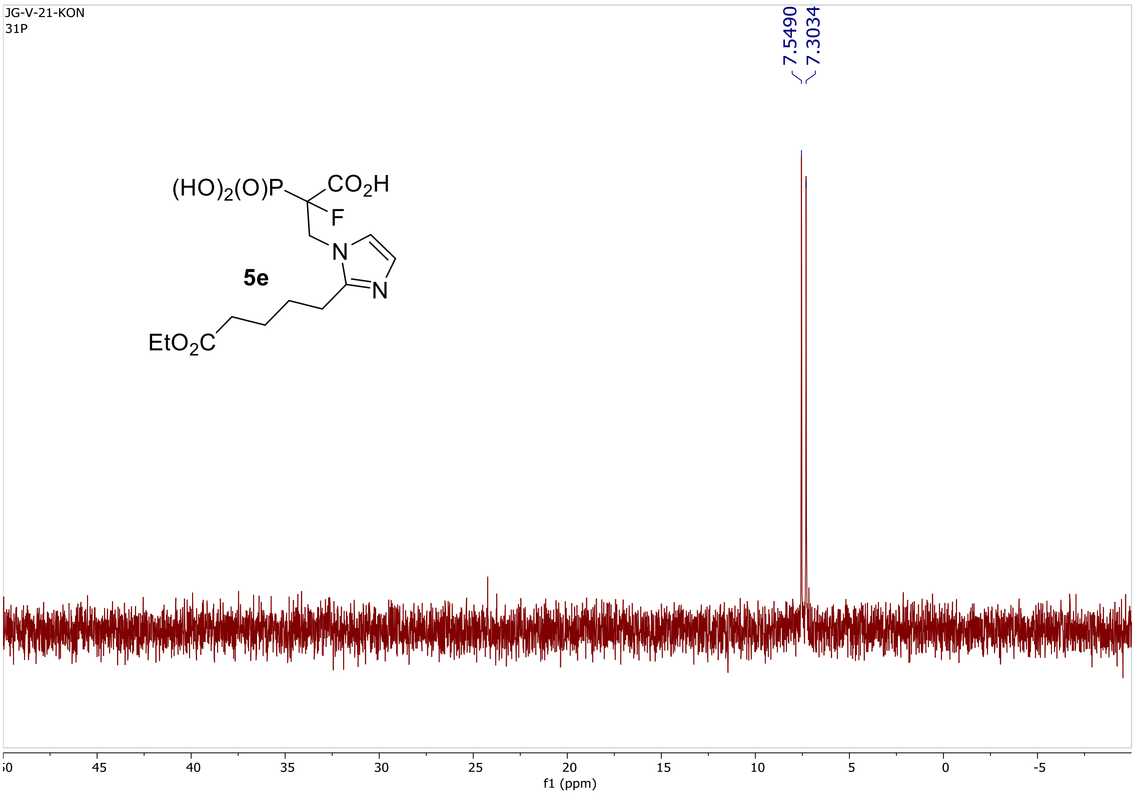

Figure S87. ^{31}P NMR (283 MHz, D_2O, pH 7) of compound **5e**.

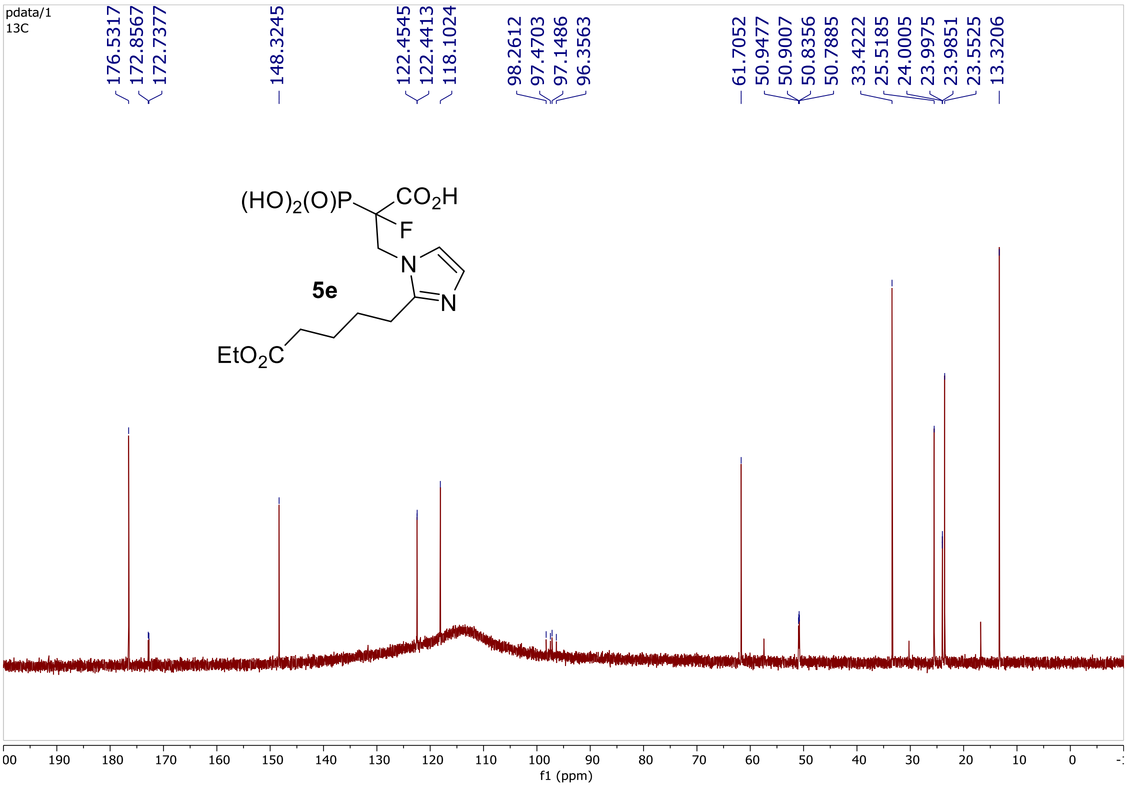

Figure S88. ^{13}C NMR (176 MHz, D_2O, pH 7) of compound **5e**.

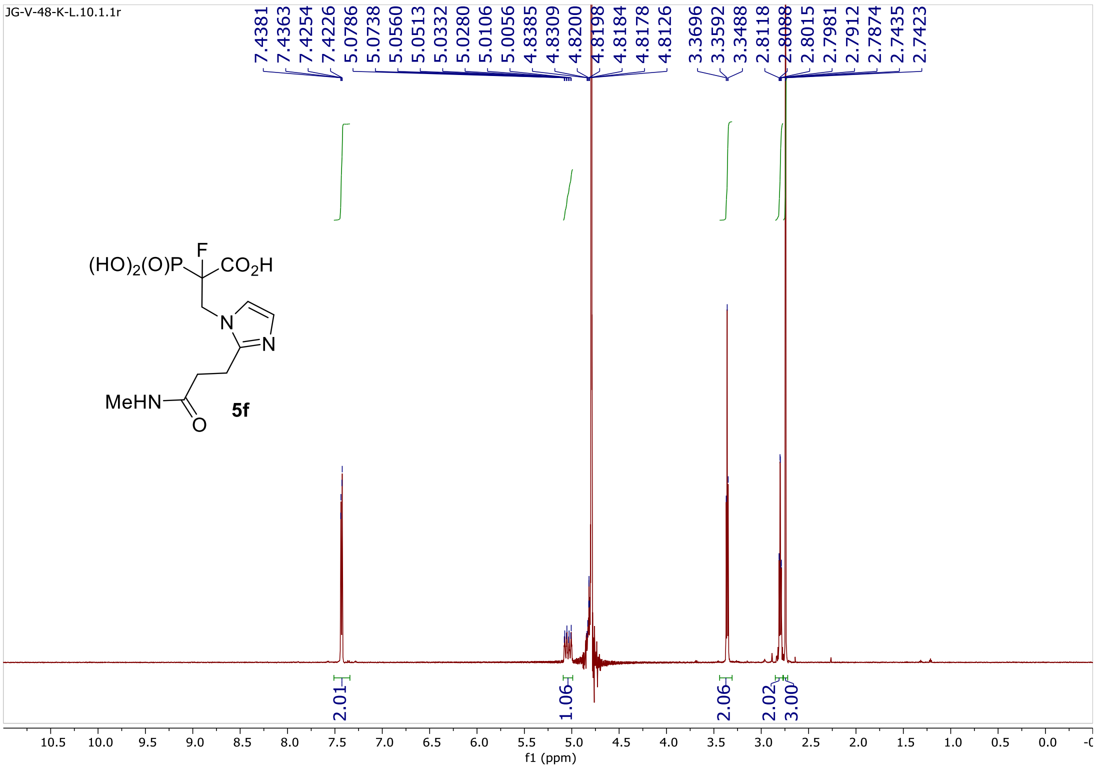

Figure S89. ^{1}H NMR (700 MHz, D_2O, pH 2) of compound **5f**.

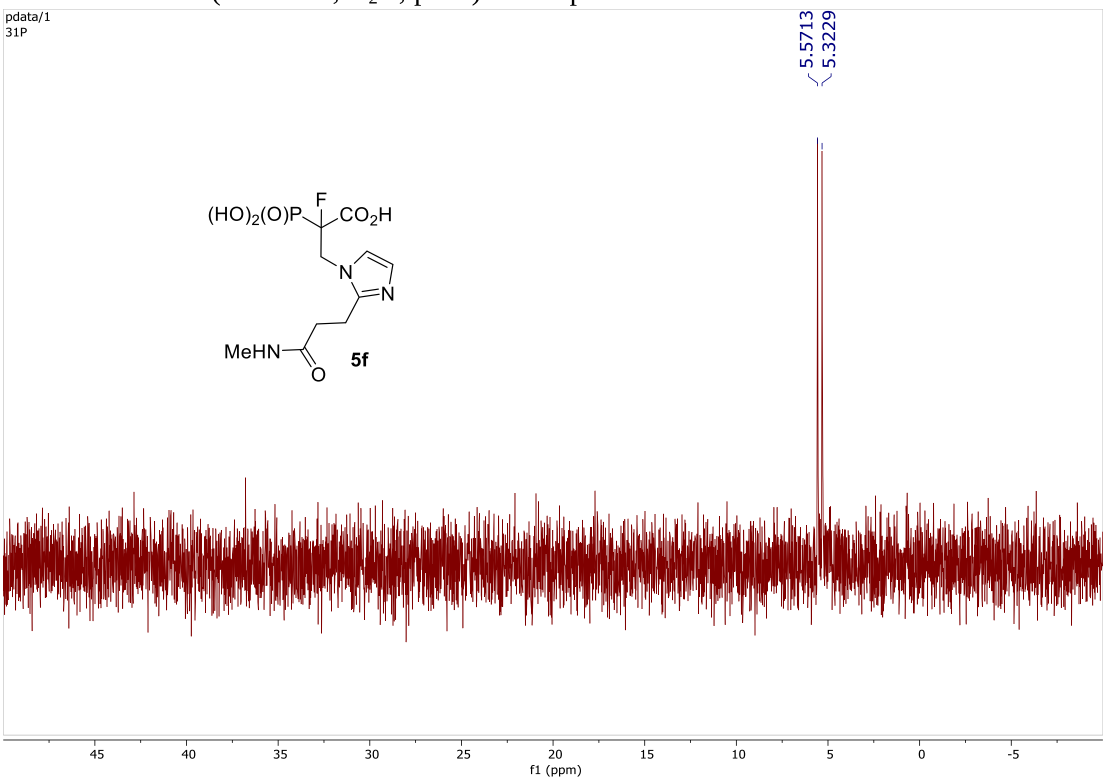

Figure S90. ^{31}P NMR (283 MHz, D_2O, pH 2) of compound **5f**.

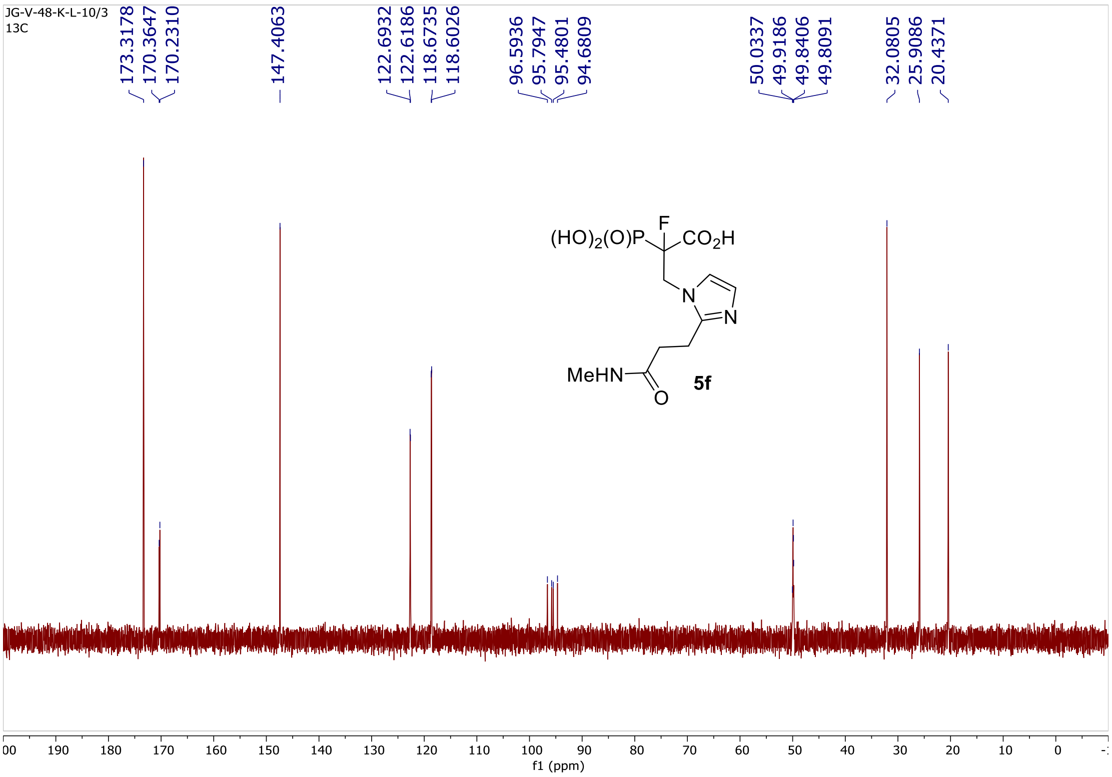

Figure S91. ^{13}C NMR (176 MHz, D_2O, pH 2) of compound **5f**.

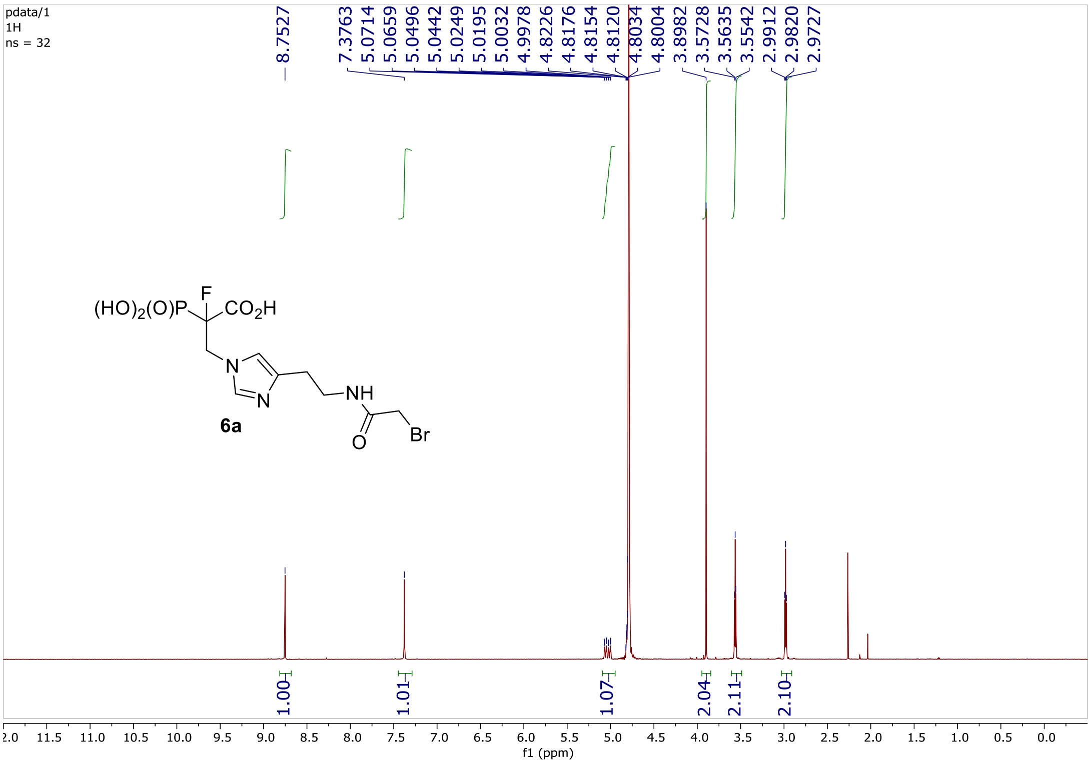

Figure S92. ^{1}H NMR (700 MHz, D_2O, pH 3) of compound **6a.**

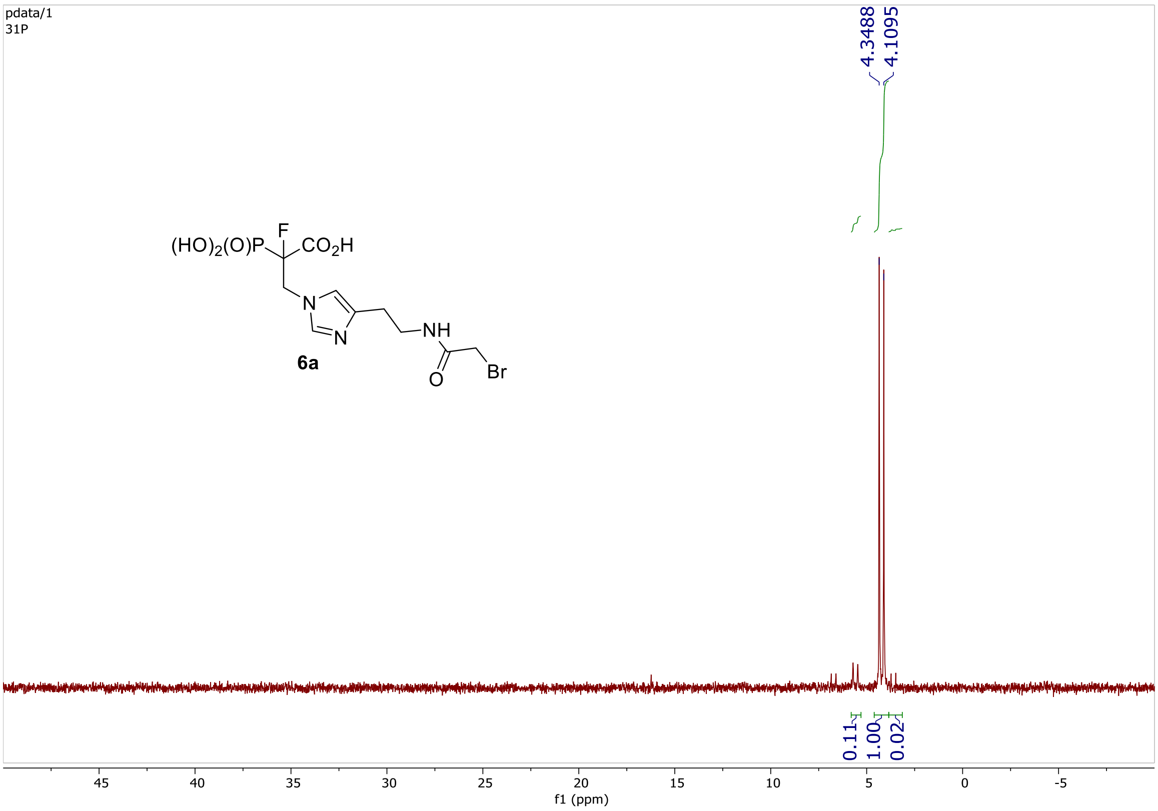

Figure S93. ^{31}P NMR (283 MHz, D_2O, pH 3) of compound **6a**.

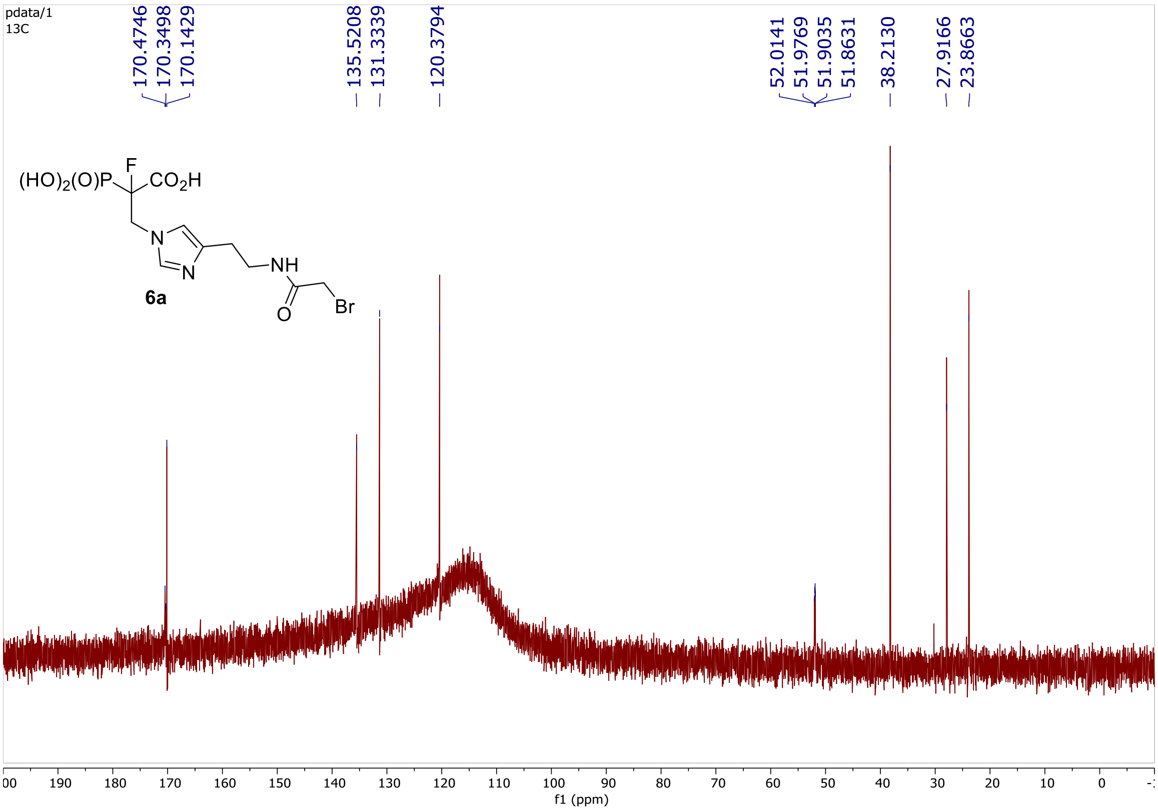

Figure S94. ^{13}C NMR (176 MHz, D_2O, pH 3) of compound **6a**.

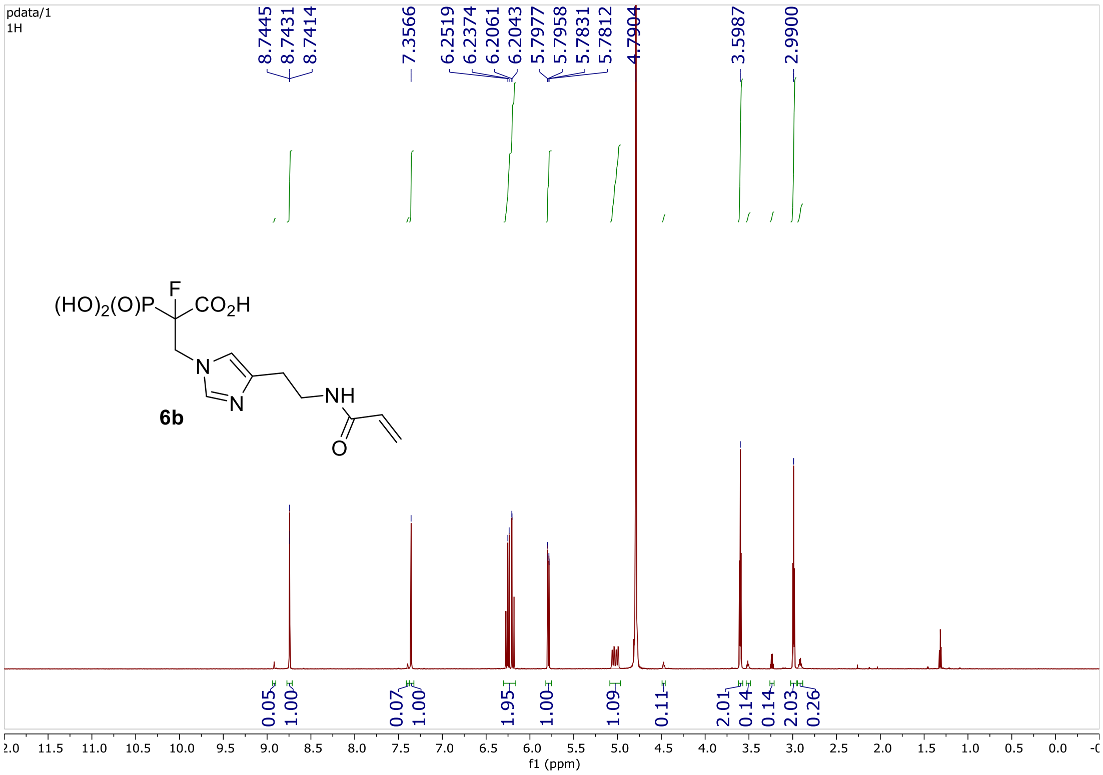

Figure S95. ^{1}H NMR (700 MHz, D_2O, pH 7) of compound **6b**.

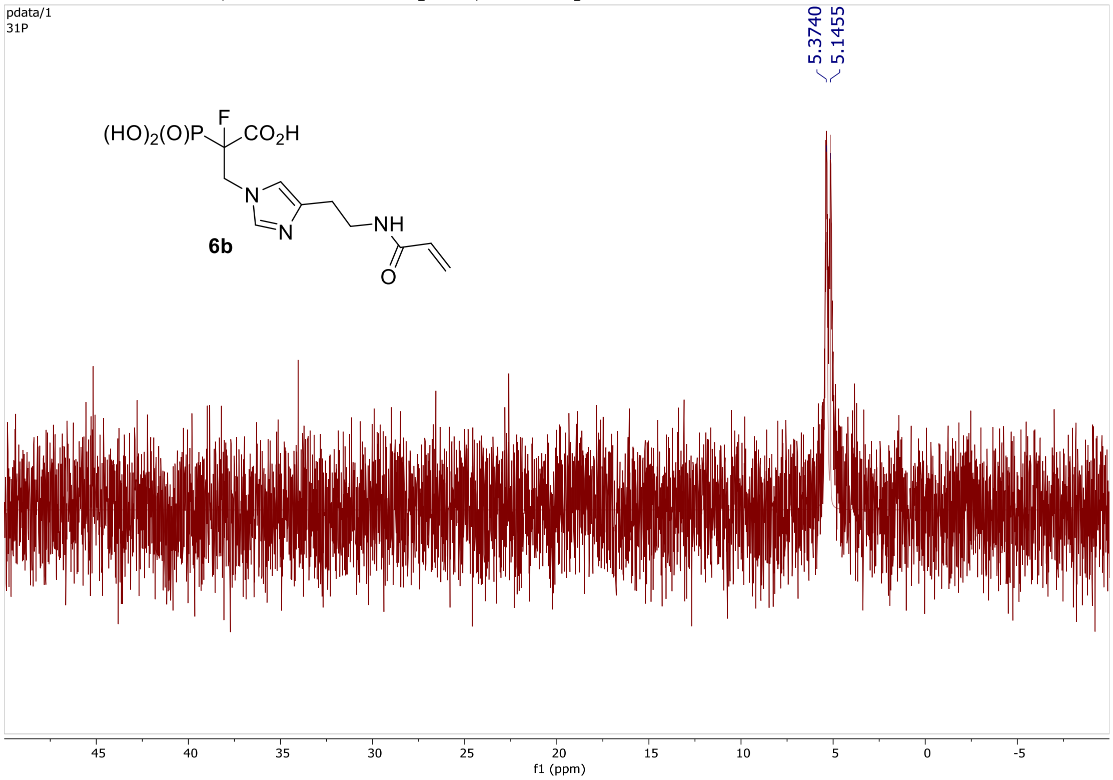

Figure S96. ^{31}P NMR (283 MHz, D_2O, pH 7) of compound **6b**.

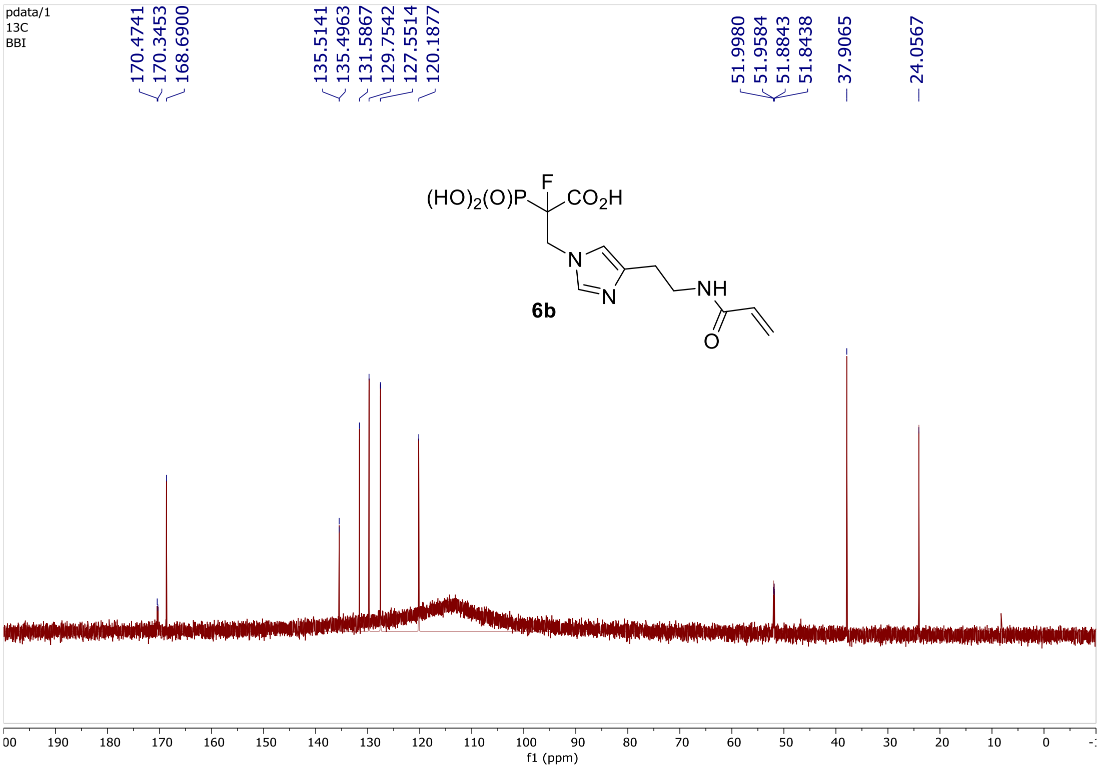

Figure S97. ^{13}C NMR (176 MHz, D_2O, pH 7) of compound **6b**.

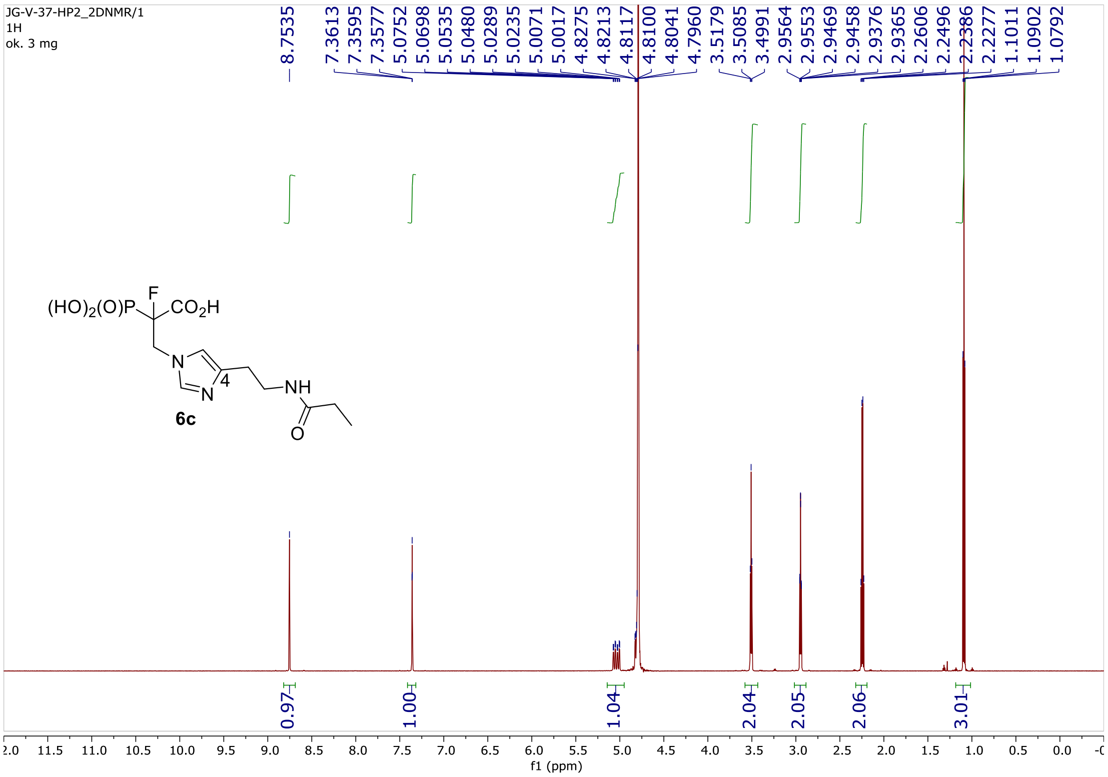

Figure S98. ^{1}H NMR (700 MHz, D_2O, pH 3) of compound **6c** (pure regioisomer C4).

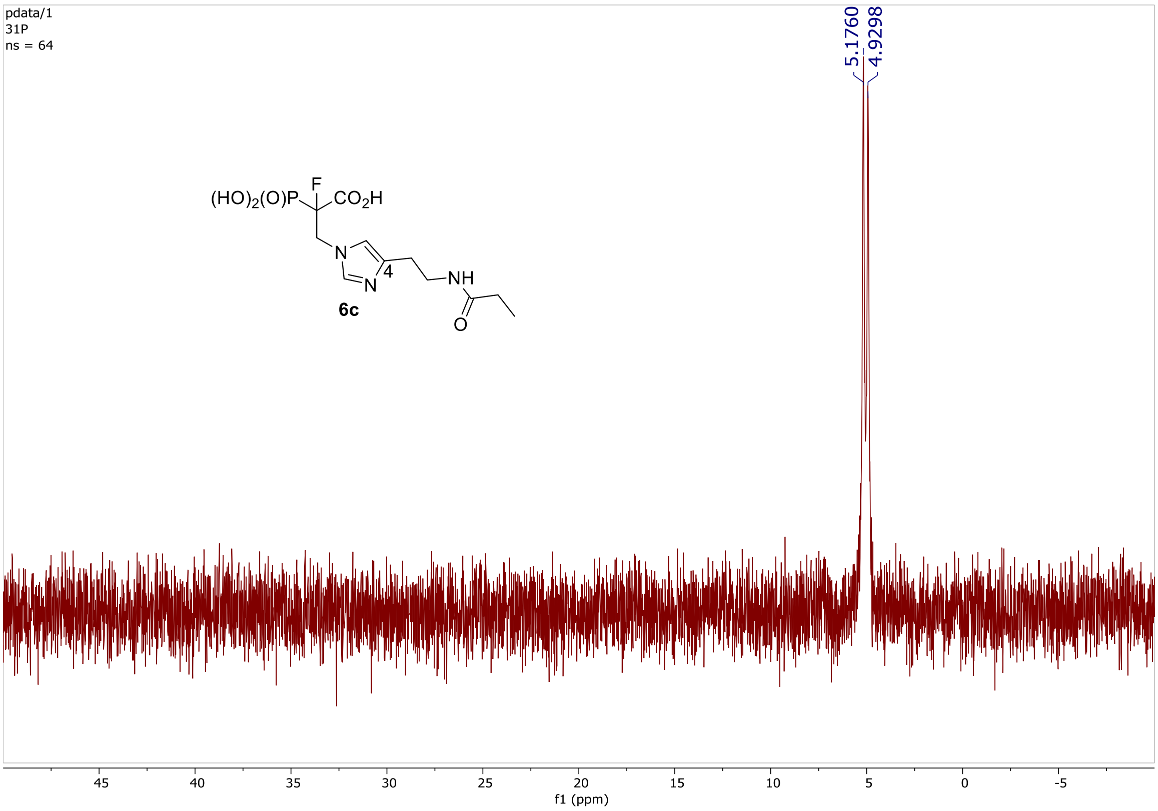

Figure S99. ^{31}P NMR (283 MHz, D_2O, pH 3) of compound **6c** (pure regioisomer C4).

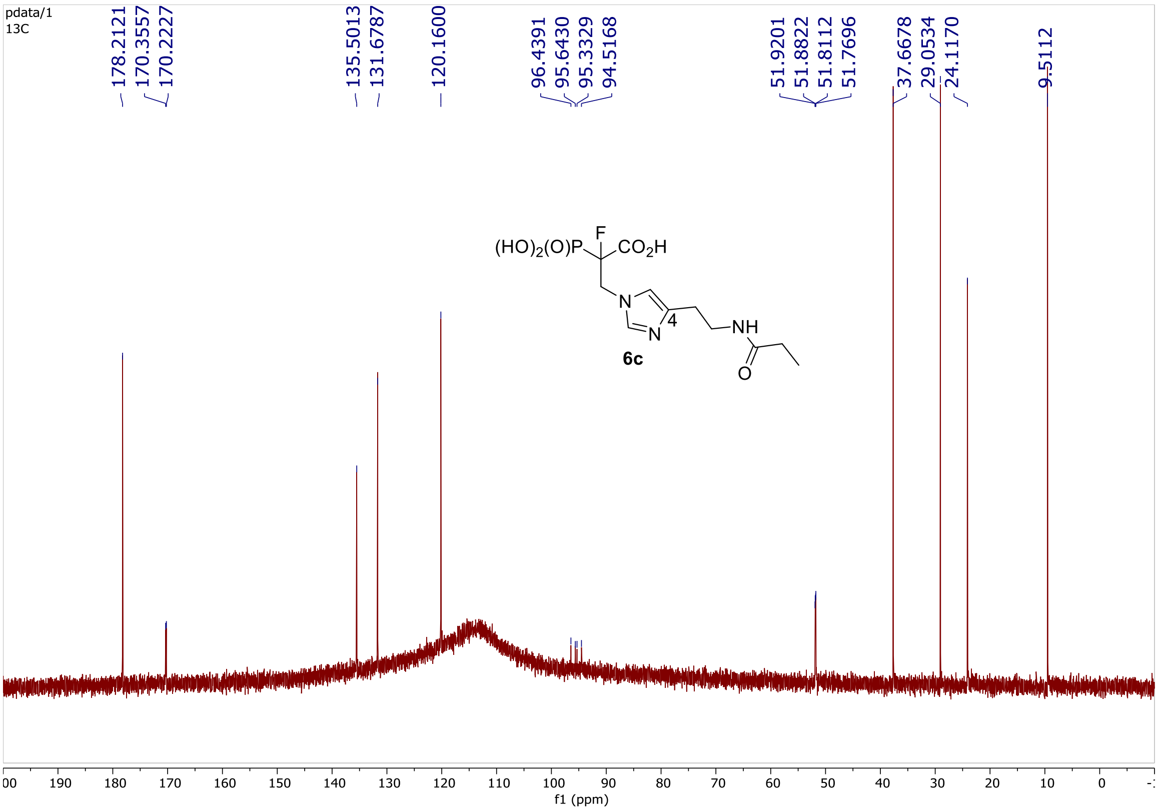

Figure S100. ^{13}C NMR (176 MHz, D_2O, pH 3) of compound **6c** (pure regioisomer C4).

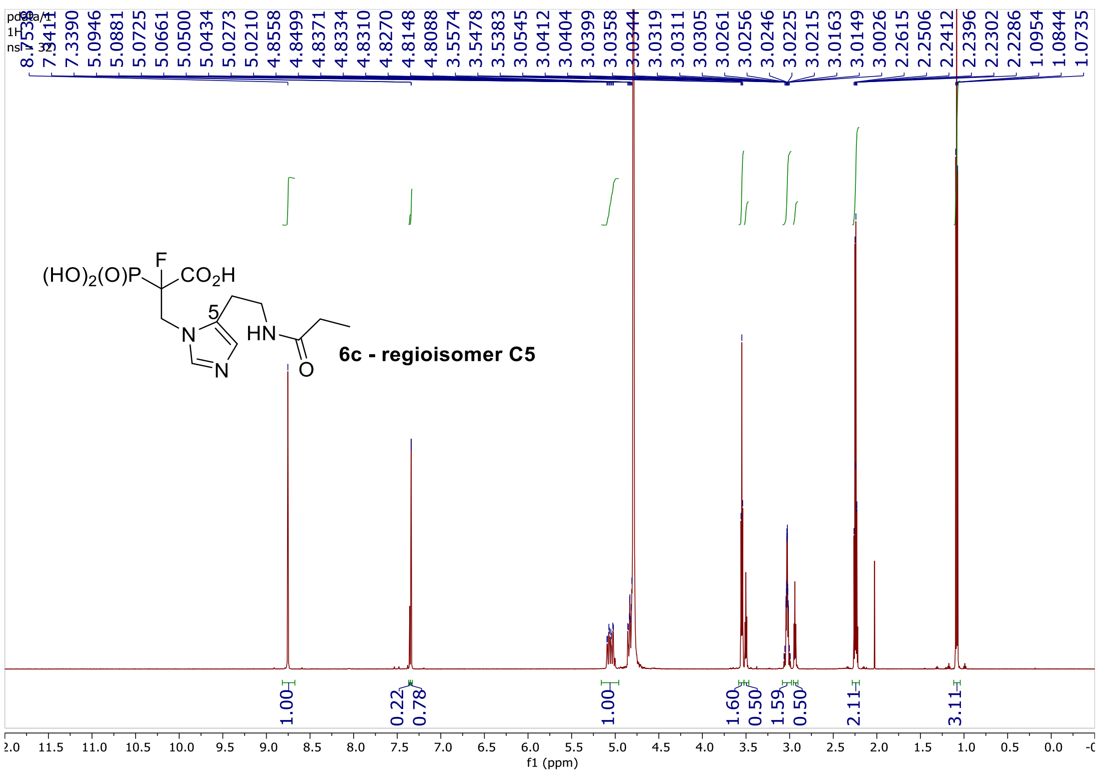

Figure S101. ^{1}H NMR (700 MHz, D_2O, pH 3) of compound **6c** (78% of regioisomer C5 and 22% of regioisomer C4).

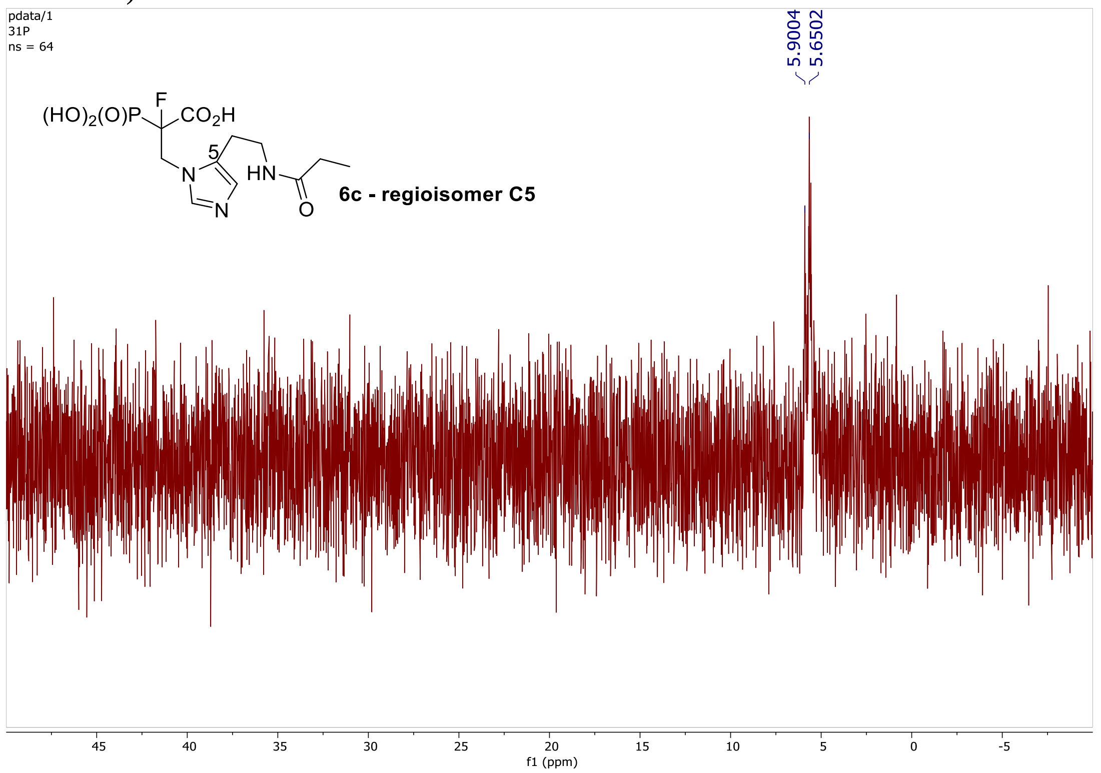

Figure S102. ^{31}P NMR (283 MHz, D_2O, pH 3) of compound **3h** (78% of regioisomer C5 and 22% of regioisomer C4).

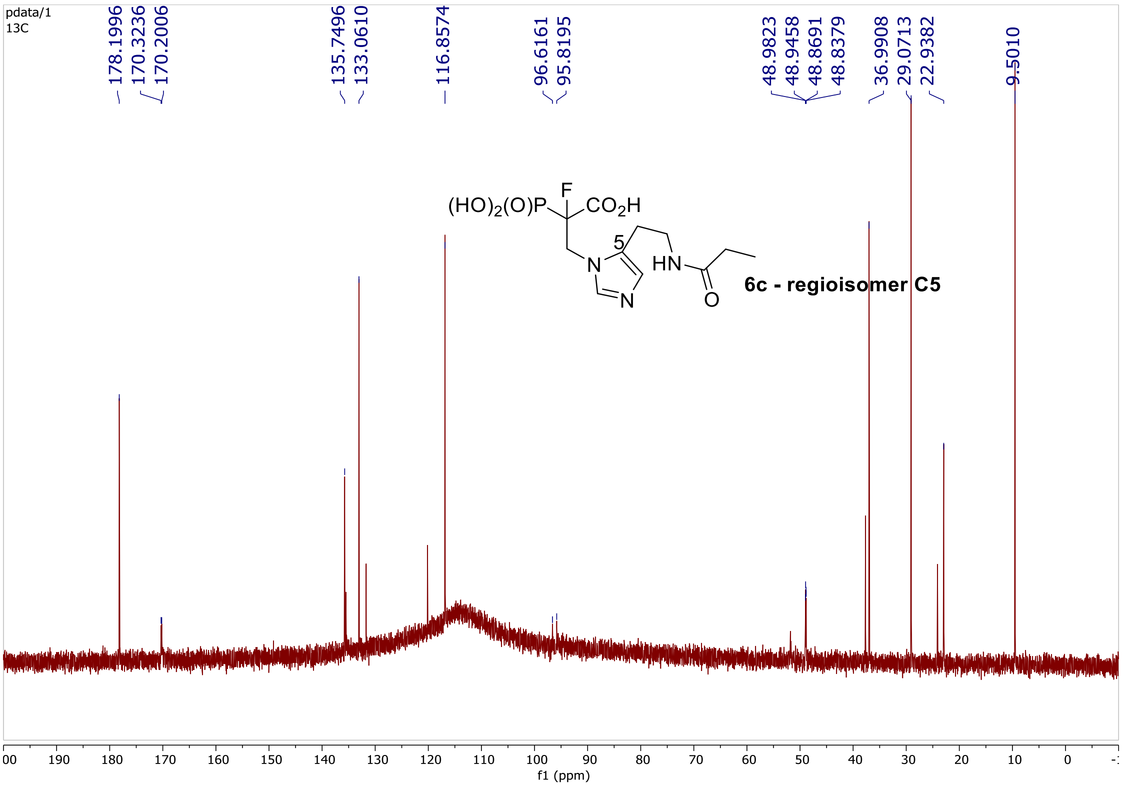

Figure S103. ^{13}C NMR (176 MHz, D_2O, pH 3) of compound **3h** (78% of regioisomer C5 and 22% of regioisomer C4).

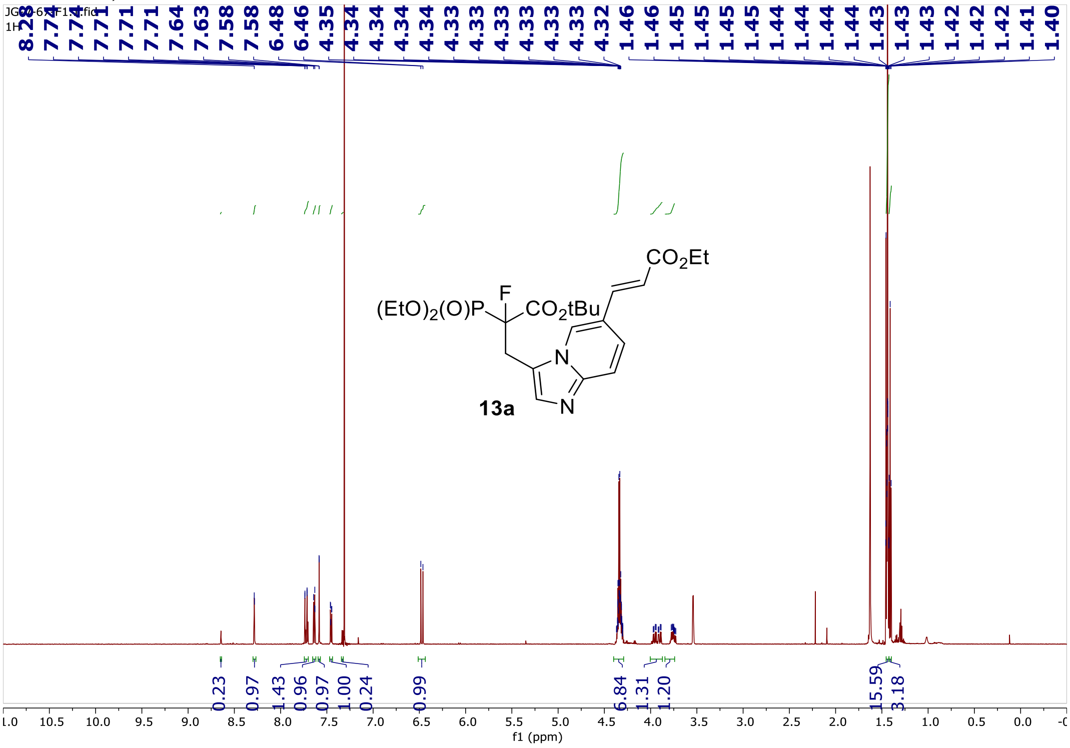

Figure S104. ^{1}H NMR (700 MHz, $CDCl_3$) of compound **13a**.

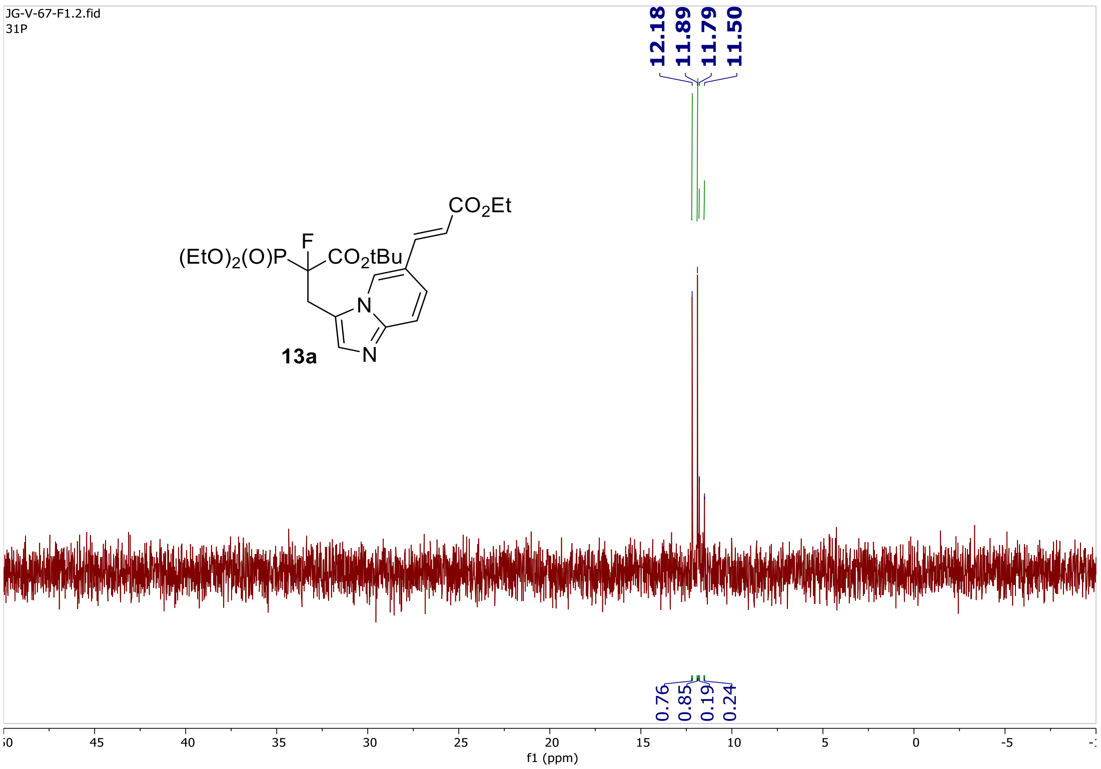

Figure S105. ^{31}P NMR (283 MHz, $CDCl_3$) of compound **13a**.

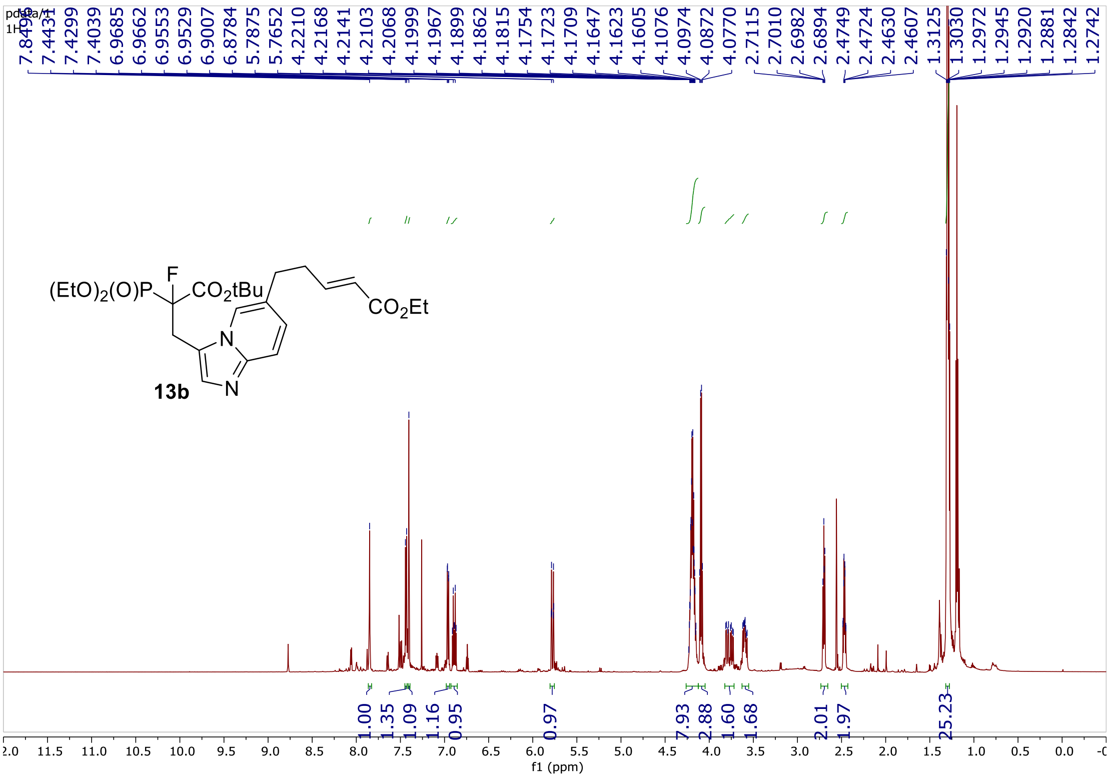

Figure S106. ^{1}H NMR (700 MHz, $CDCl_3$) of compound **13b**.

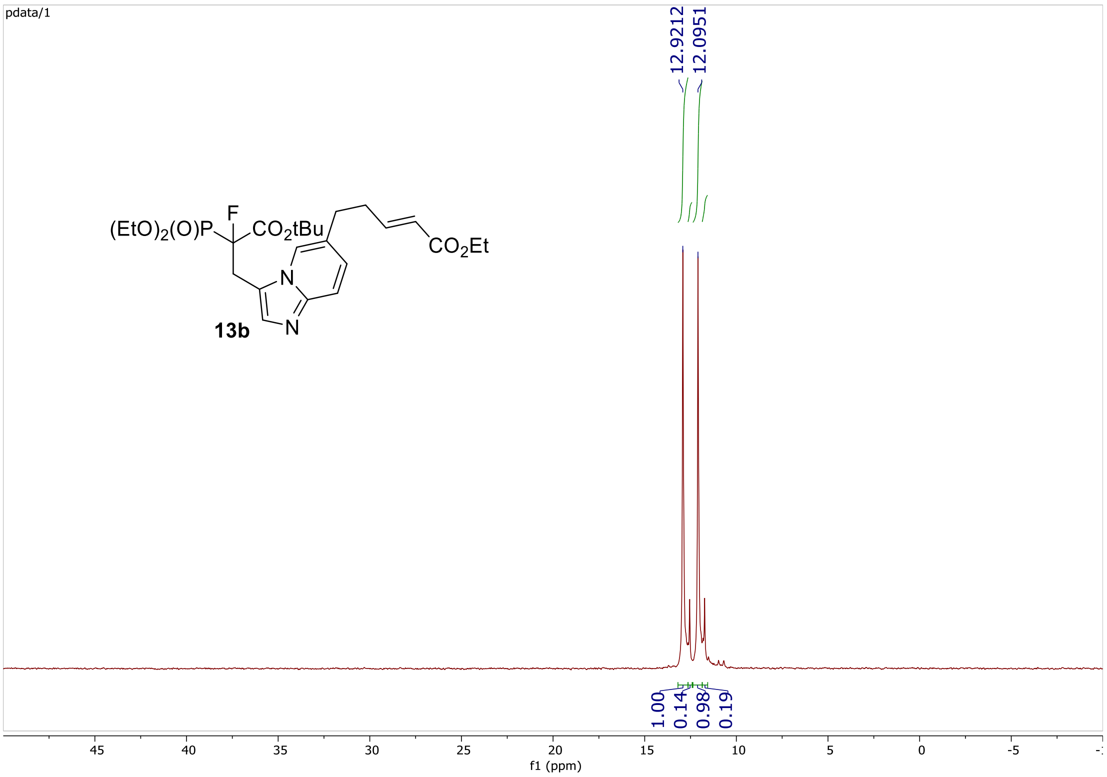

Figure S107. ^{31}P NMR (101 MHz, $CDCl_3$) of compound **13b**.

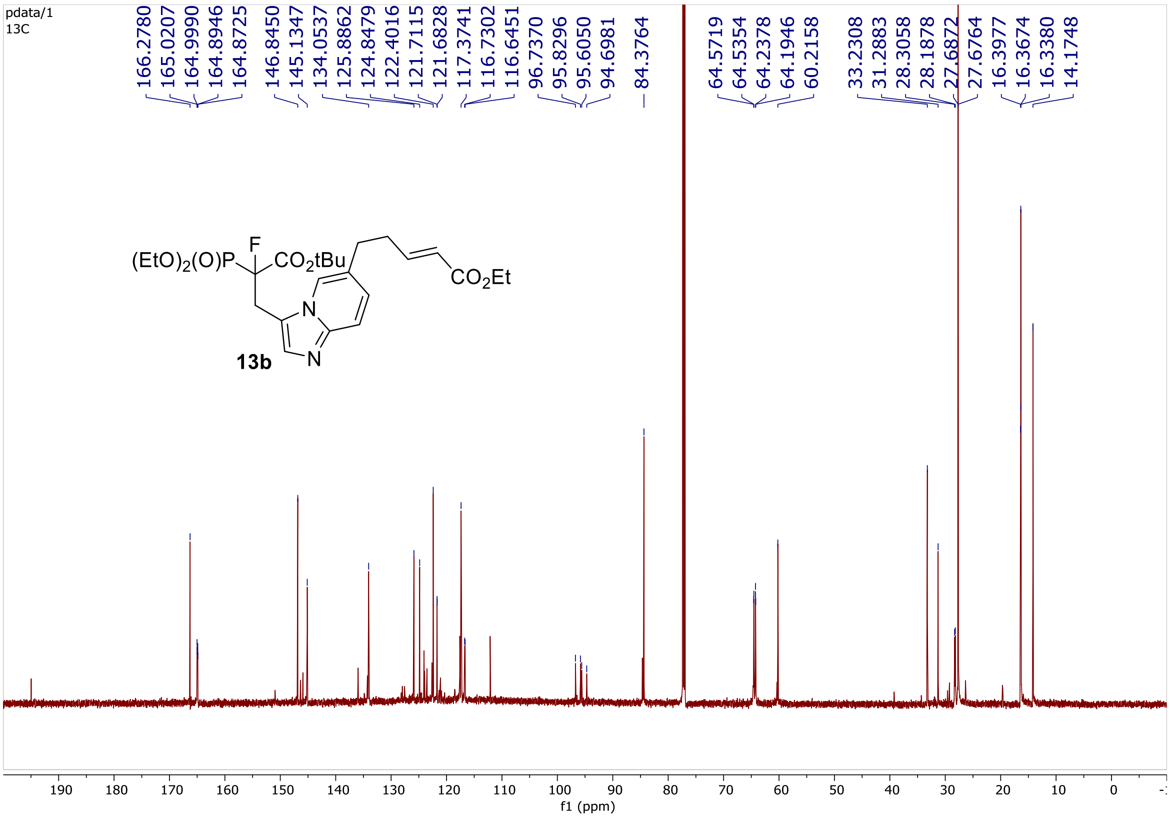

Figure S108. ^{13}C NMR (176 MHz, $CDCl_3$) of compound **13b**.

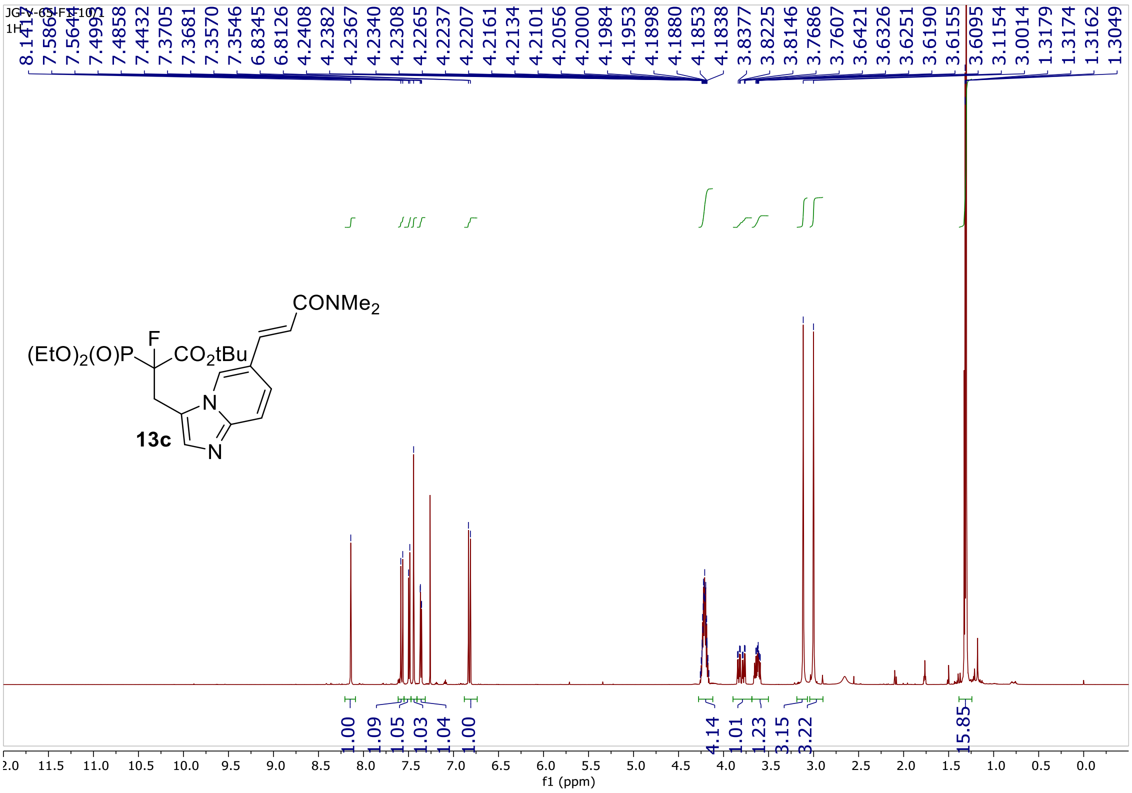

Figure S109. ^{1}H NMR (700 MHz, $CDCl_3$) of compound **13c**.

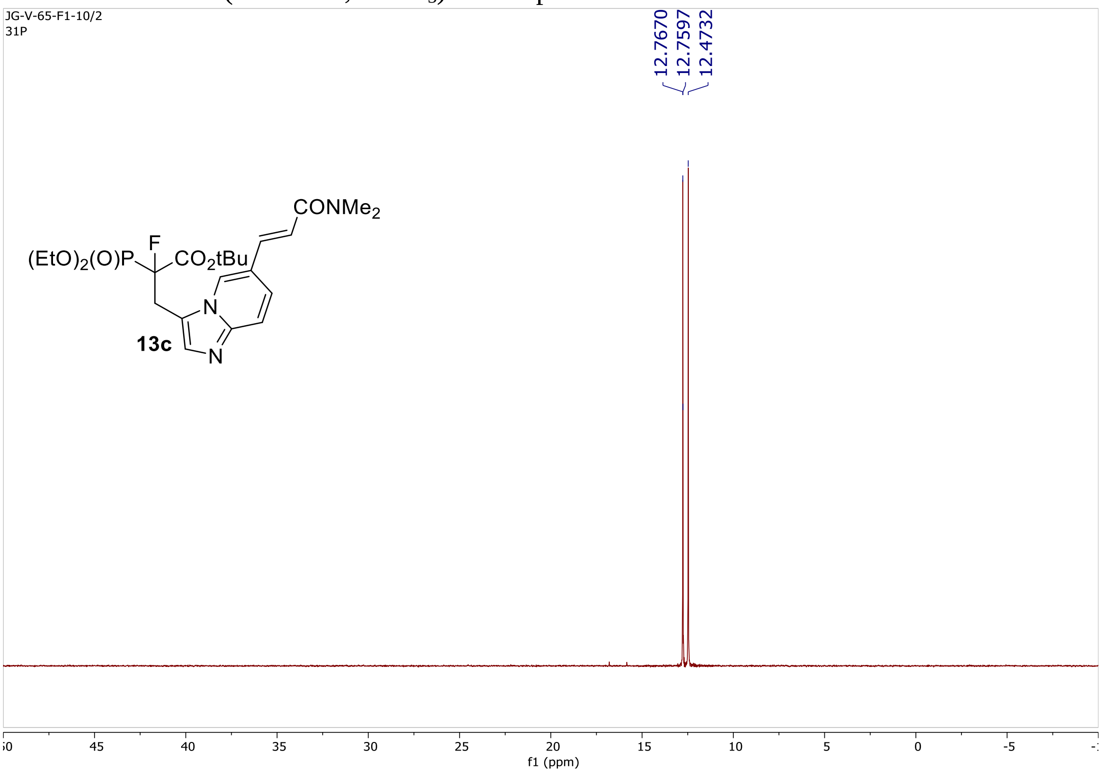

Figure S110. ^{31}P NMR (283 MHz, $CDCl_3$) of compound **13c**.

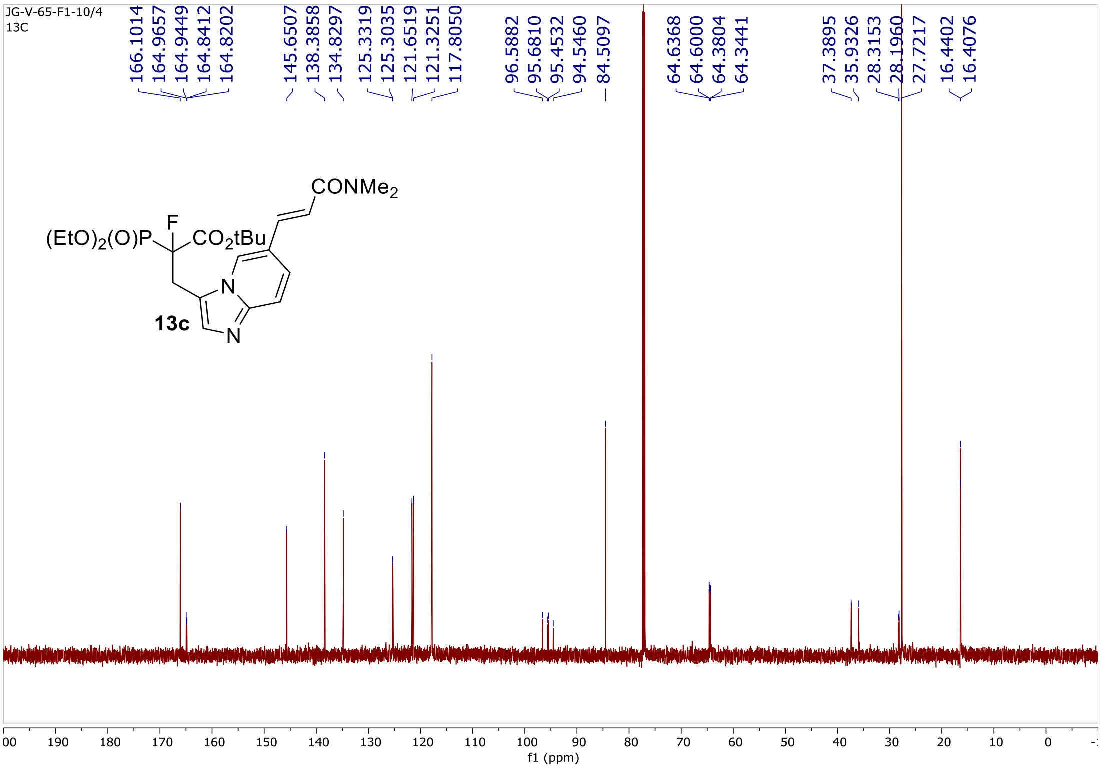

Figure S111. ^{13}C NMR (176 MHz, $CDCl_3$) of compound **13c**.

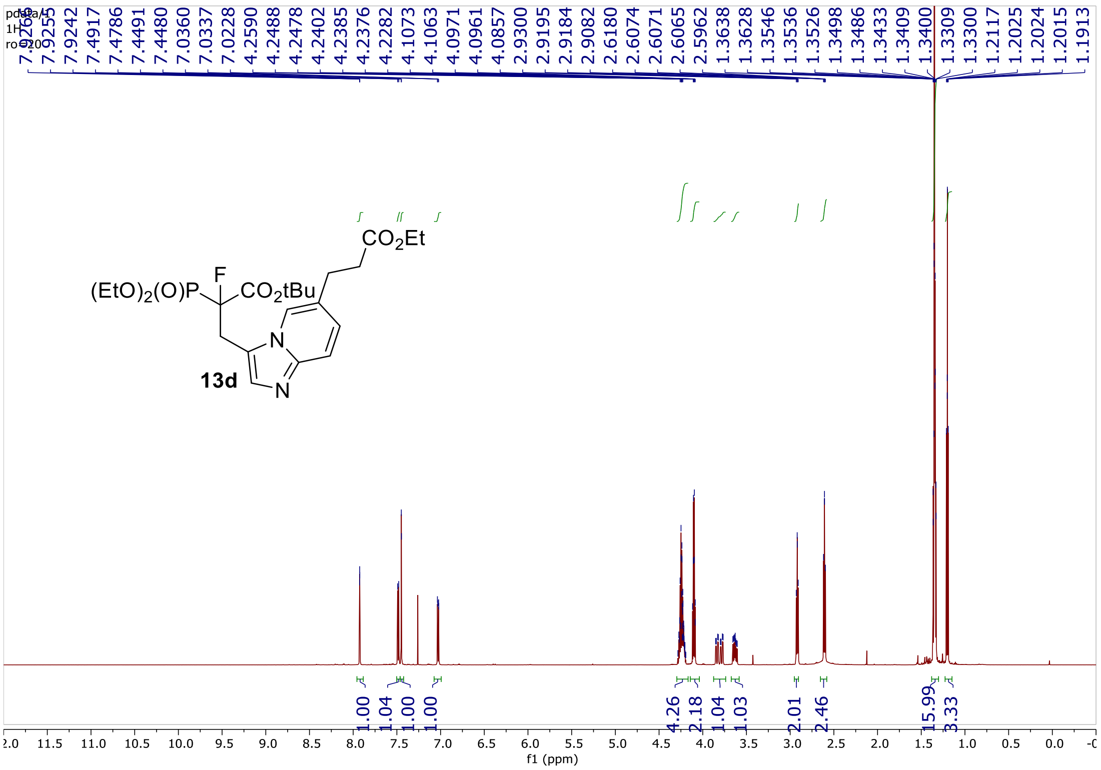

Figure S112. ^{1}H NMR (700 MHz, $CDCl_3$) of compound **13d**.

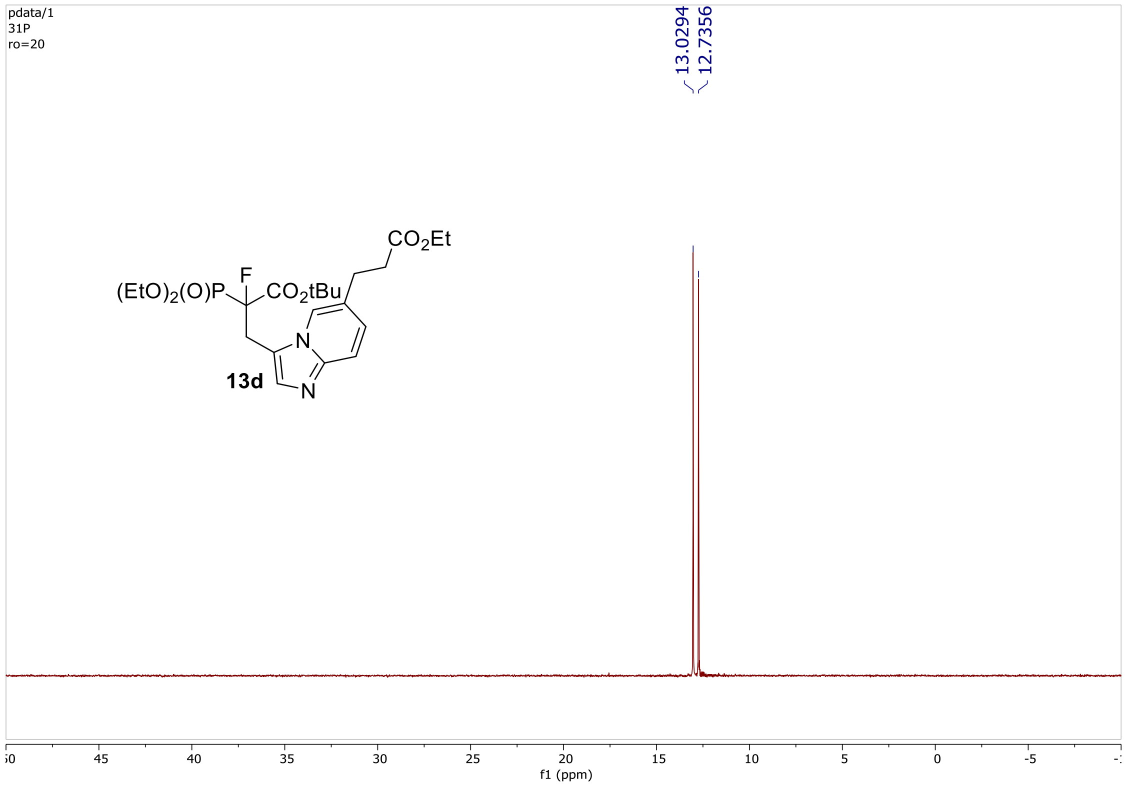

Figure S113. ^{31}P NMR (283 MHz, $CDCl_3$) of compound **13d**.

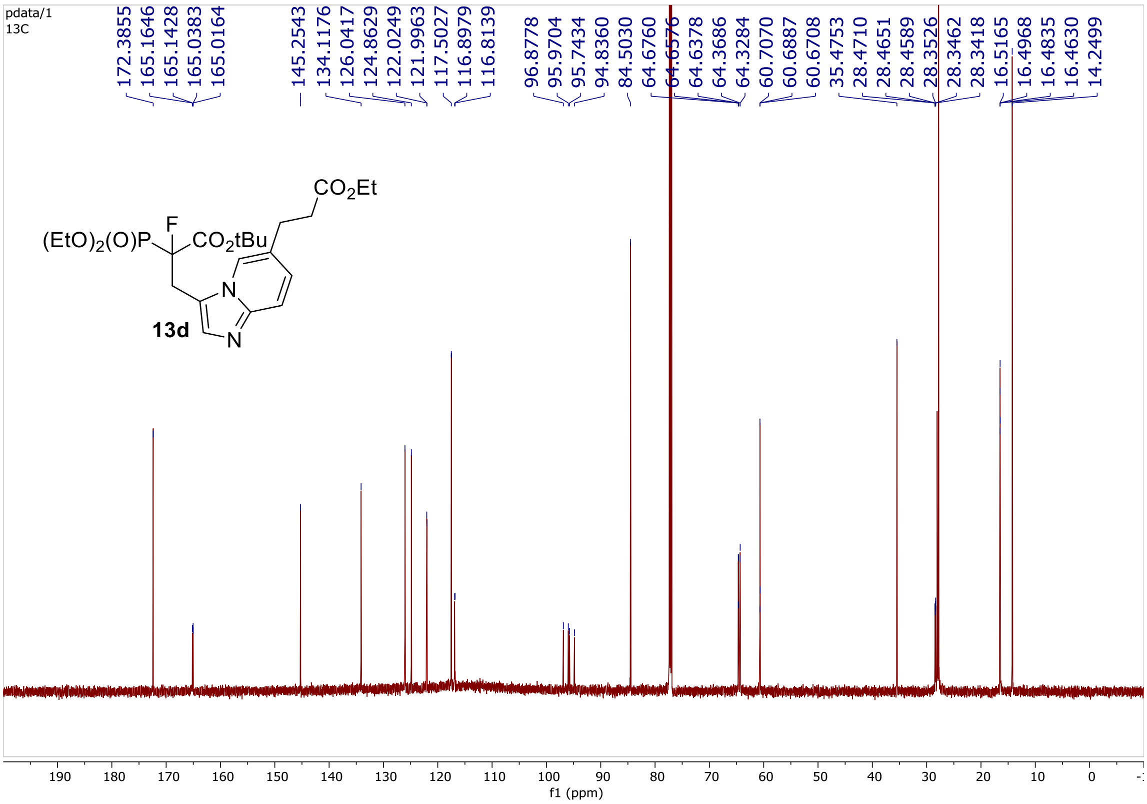

Figure S114. ^{13}C NMR (176 MHz, $CDCl_3$) of compound **13d**.

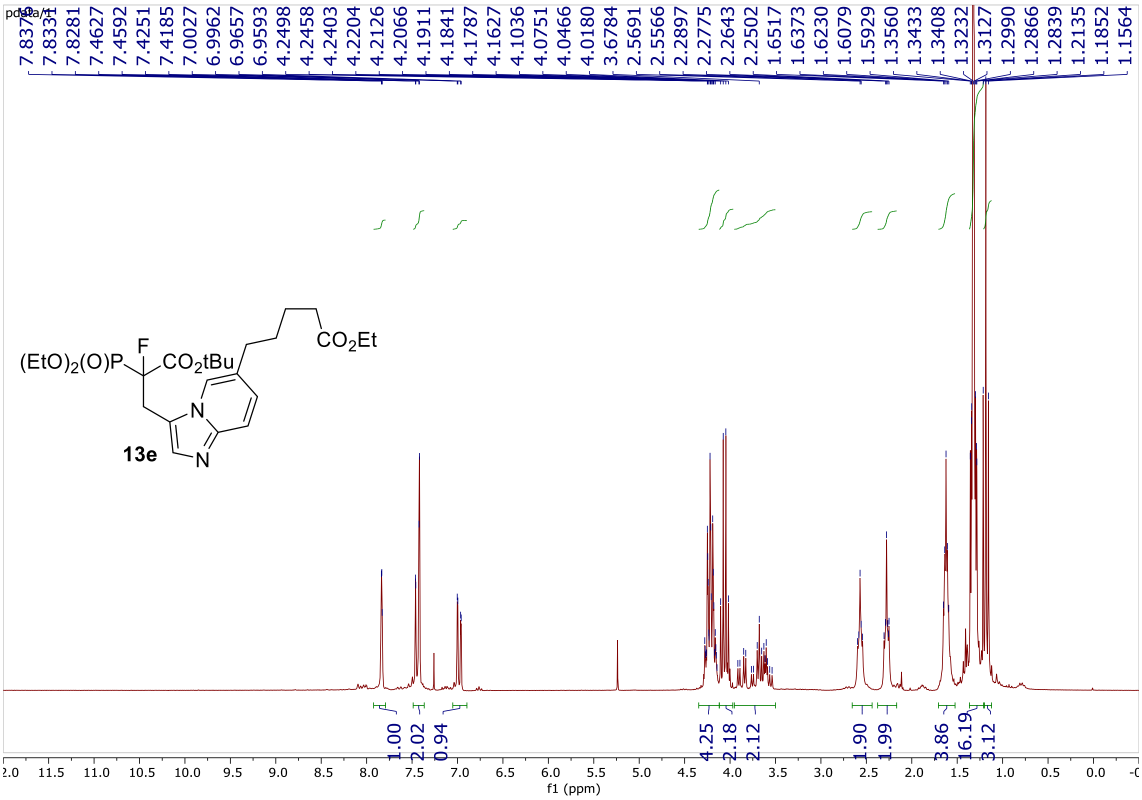

Figure S115. ^{1}H NMR (250 MHz, $CDCl_3$) of compound **13e**.

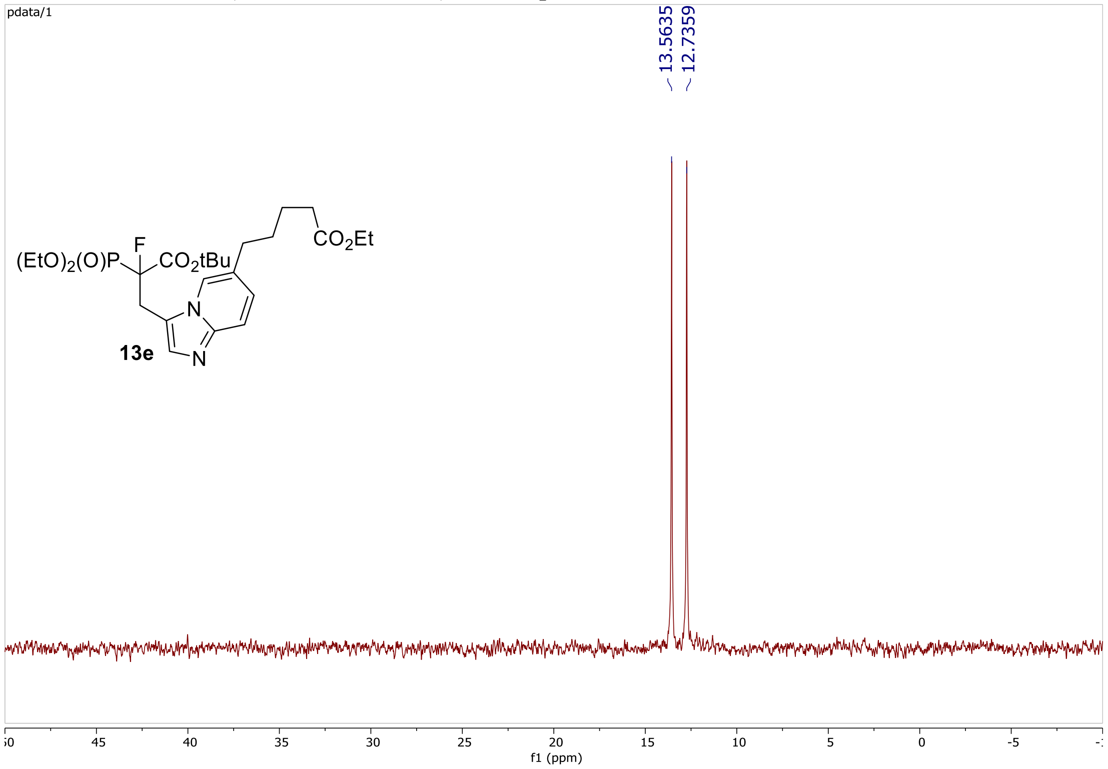

Figure S116. ^{31}P NMR (101 MHz, $CDCl_3$) of compound **13e**.

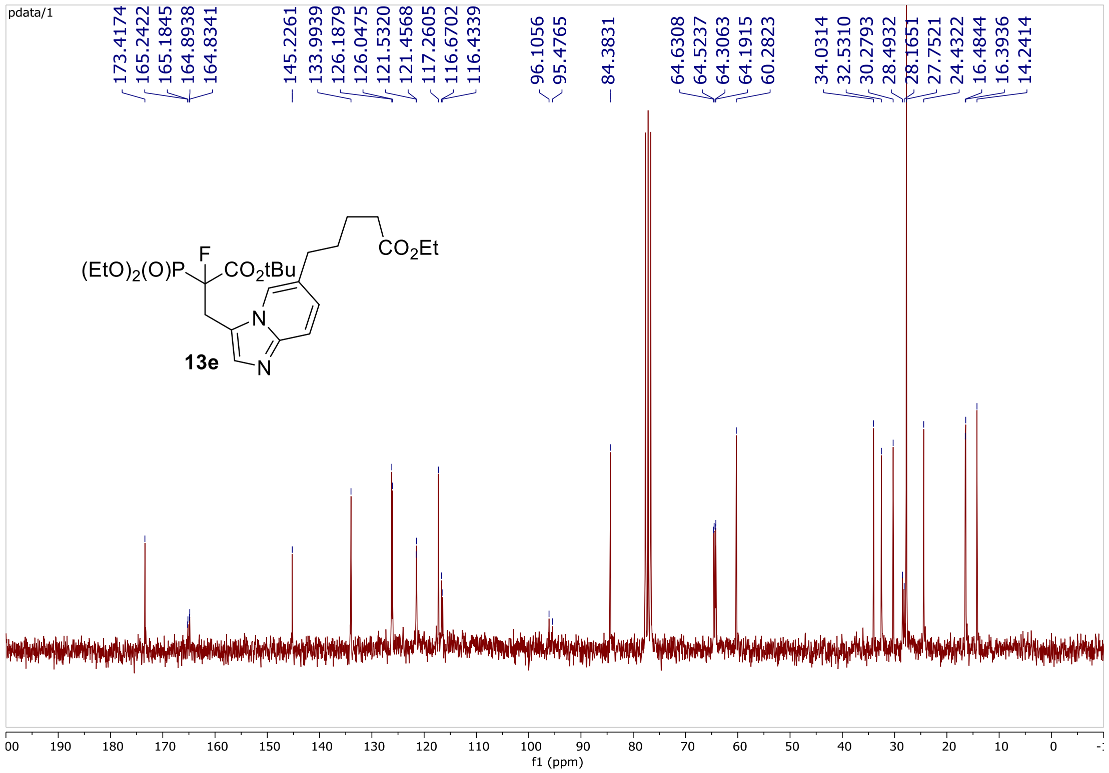

Figure S117. ^{13}C NMR (63 MHz, $CDCl_3$) of compound **13e**.

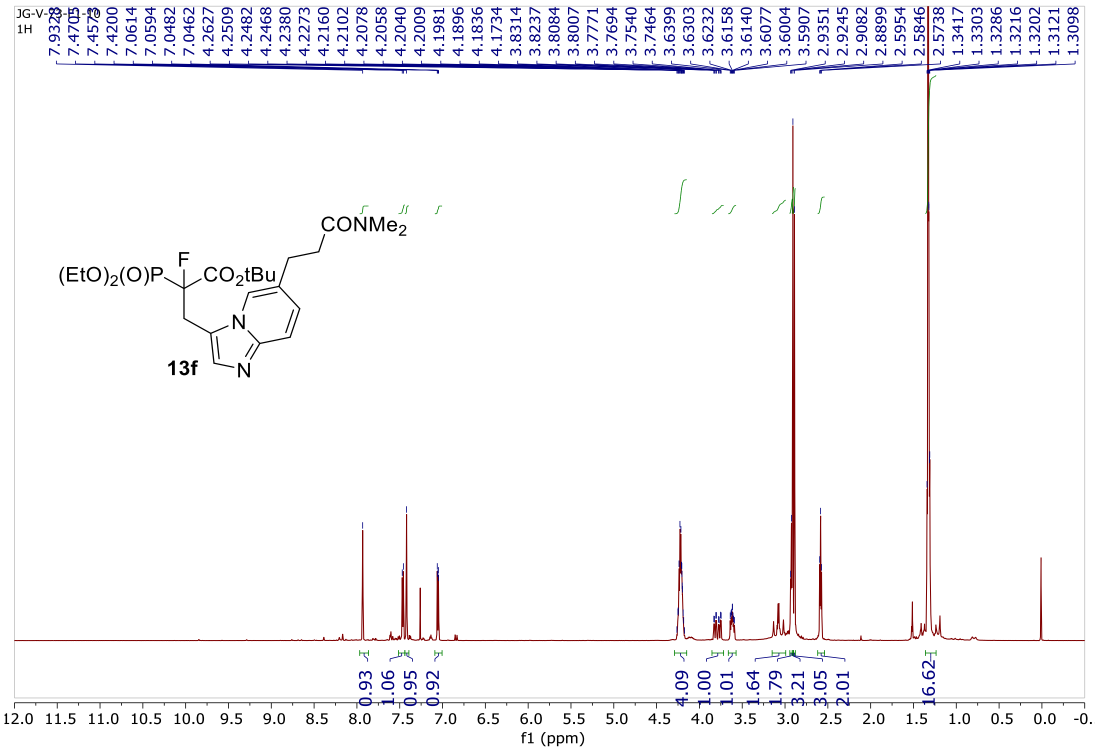

Figure S118. ^{1}H NMR (700 MHz, $CDCl_3$) of compound **13f**.

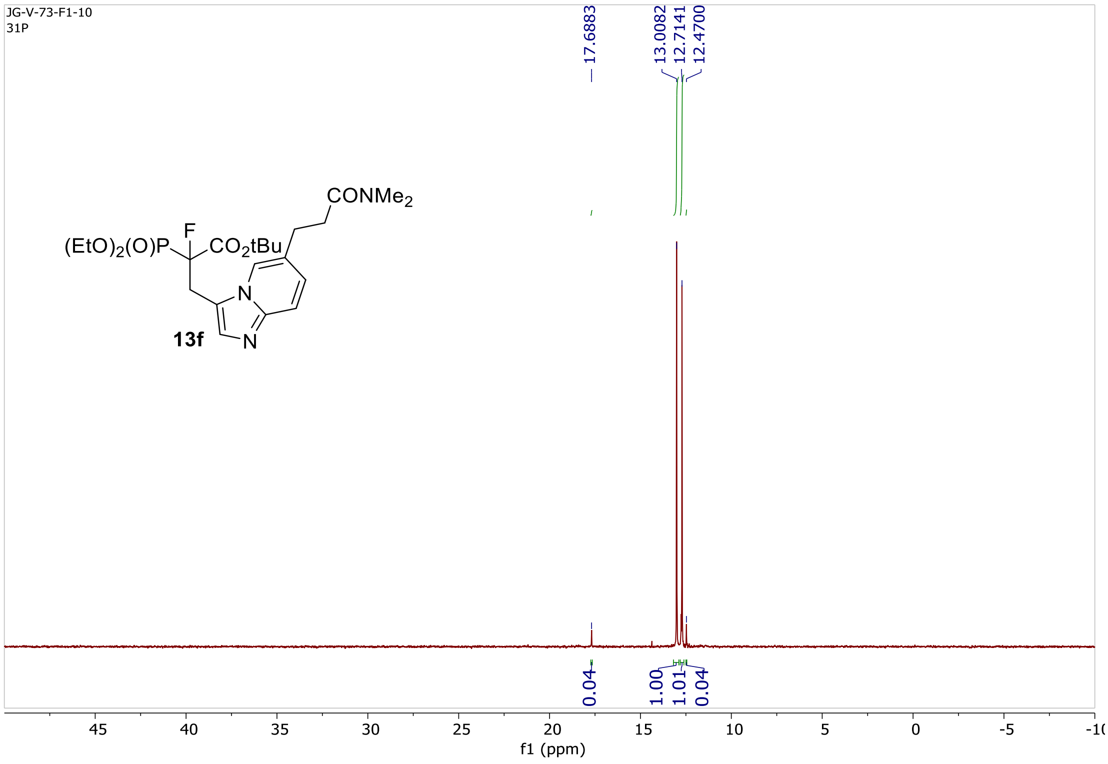

Figure S119. ^{31}P NMR (283 MHz, $CDCl_3$) of compound **13f**.

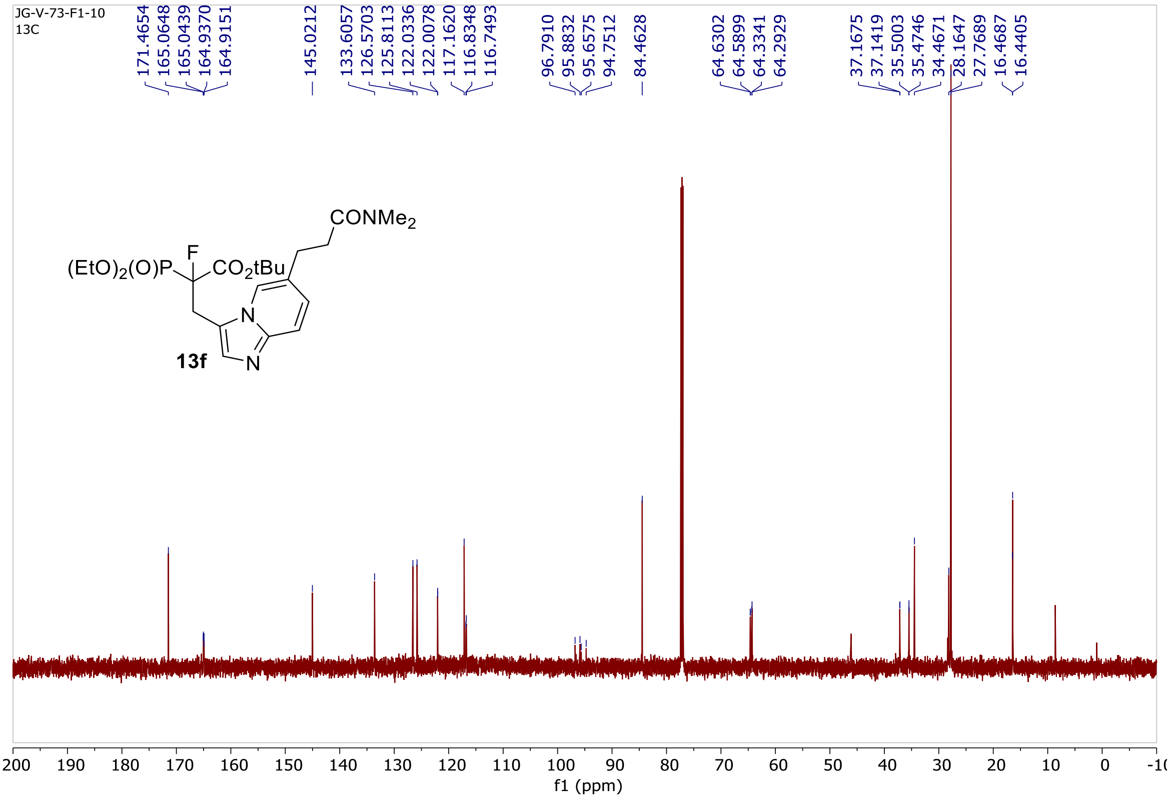

Figure S120. ^{13}C NMR (176 MHz, $CDCl_3$) of compound **13f**.

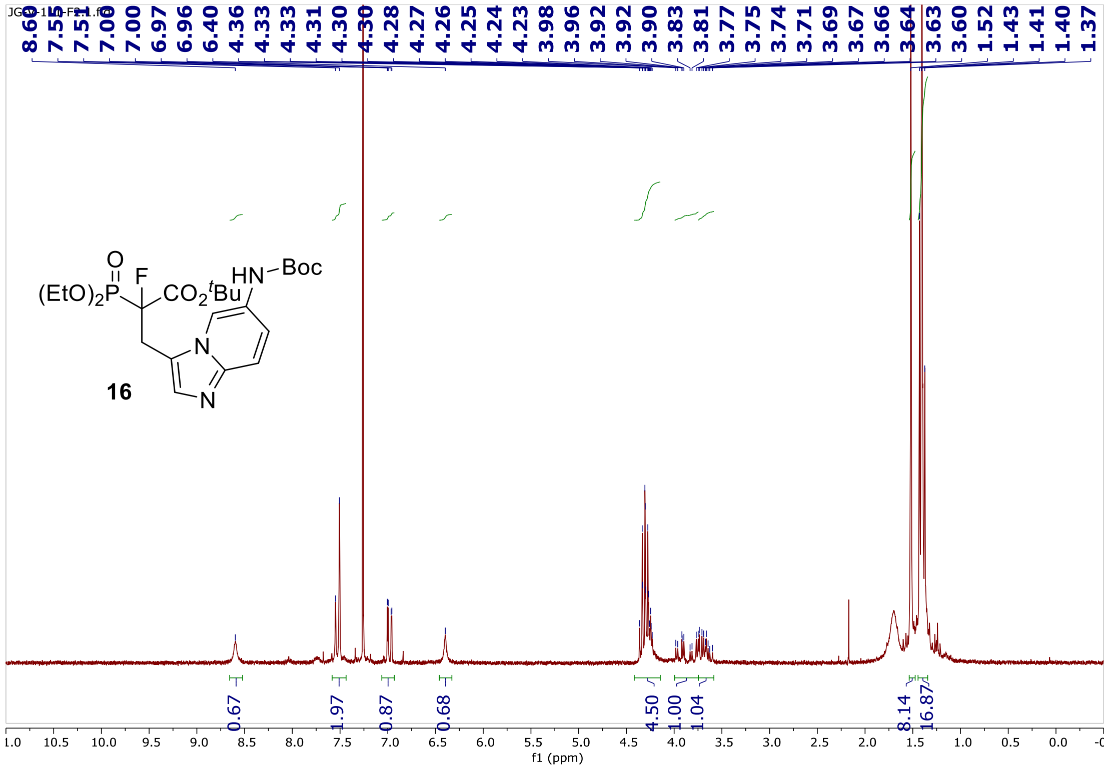

Figure S121. ^{1}H NMR (250 MHz, $CDCl_3$) of compound **16**.

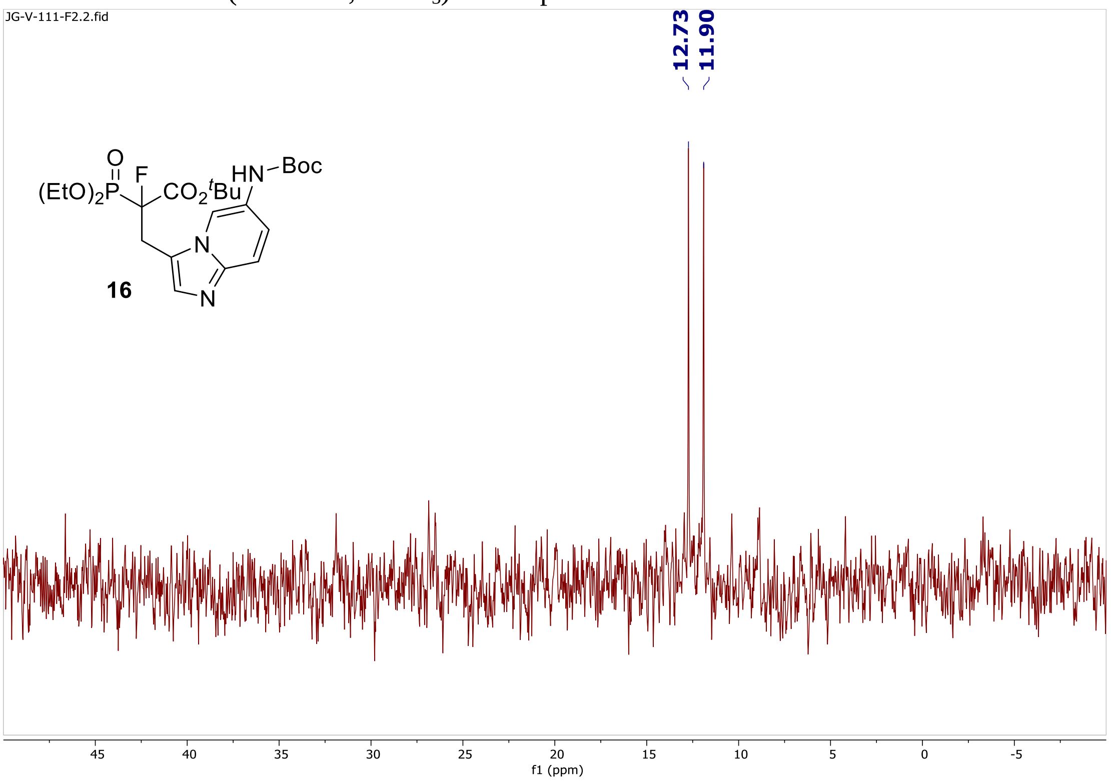

Figure S122. ^{31}P NMR (101 MHz, $CDCl_3$) of compound **16.**

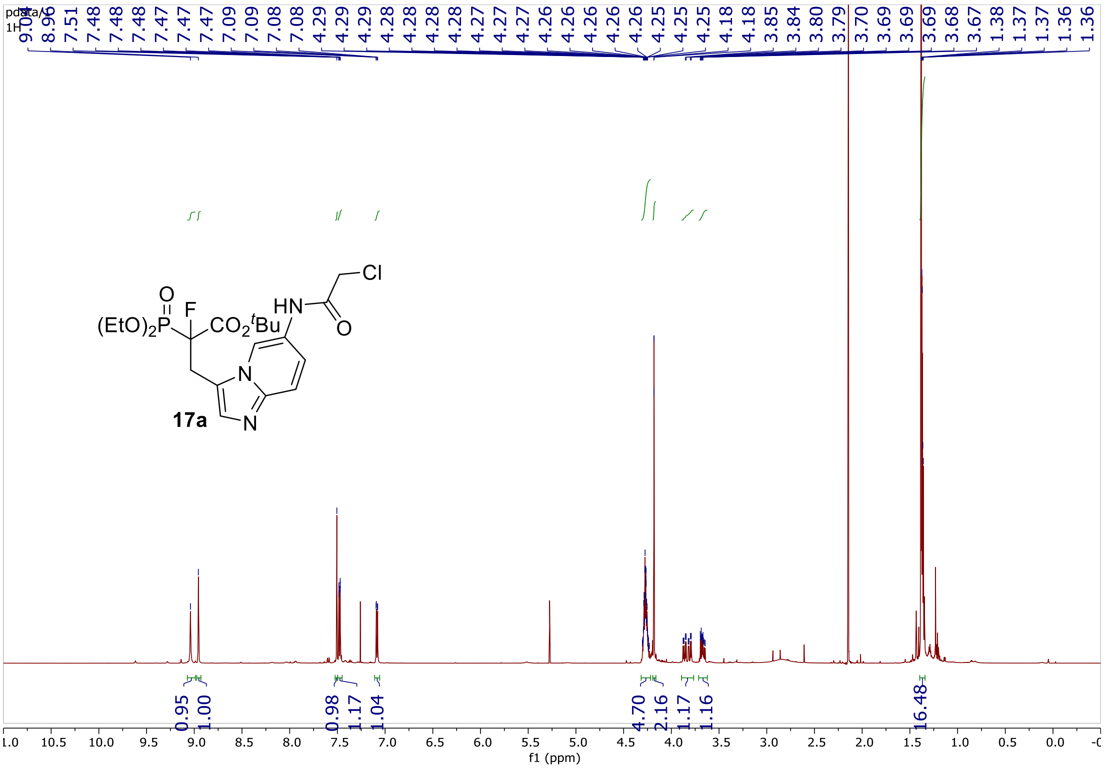

Figure S123. ^{1}H NMR (700 MHz, $CDCl_3$) of compound **17a**.

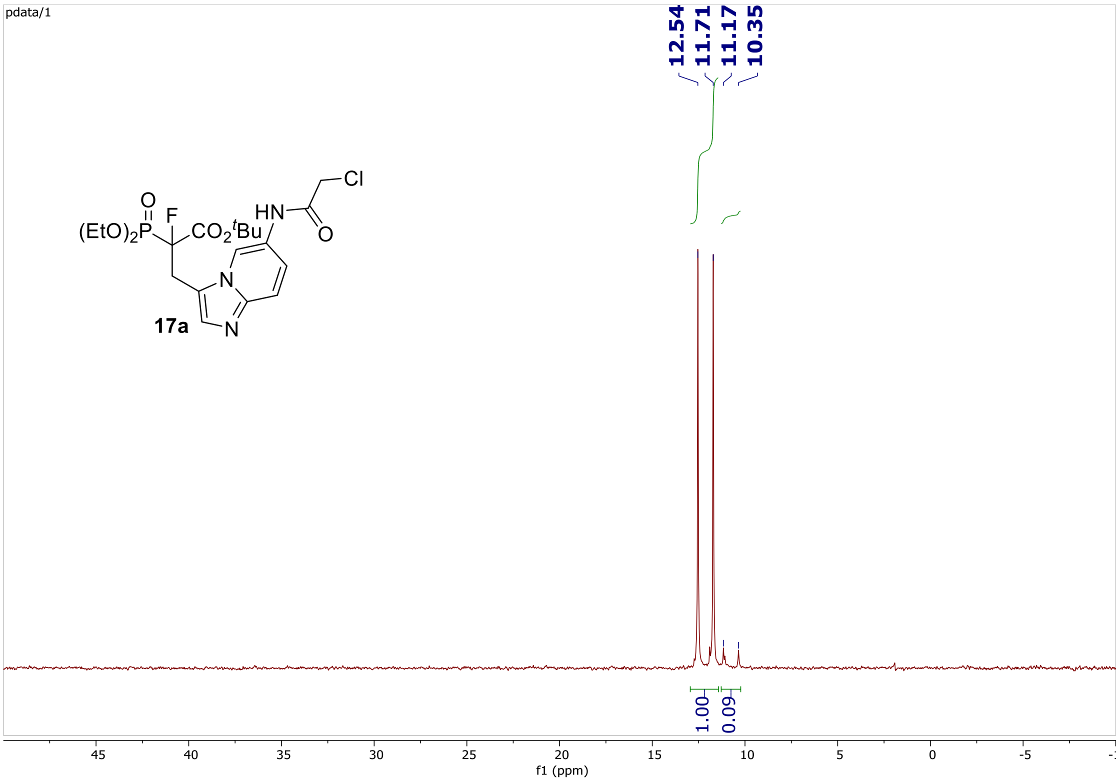

Figure S124. ^{31}P NMR (101 MHz, $CDCl_3$) of compound **17a**.

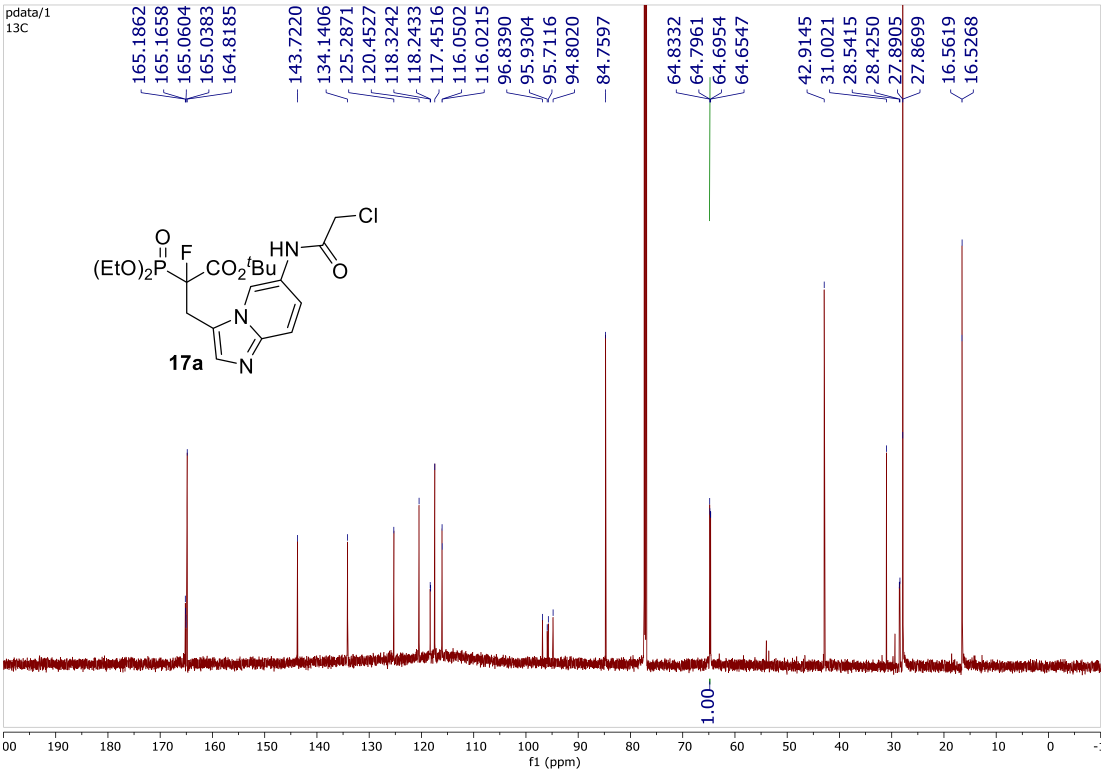

Figure S125. ^{13}C NMR (176 MHz, $CDCl_3$) of compound **17a.**

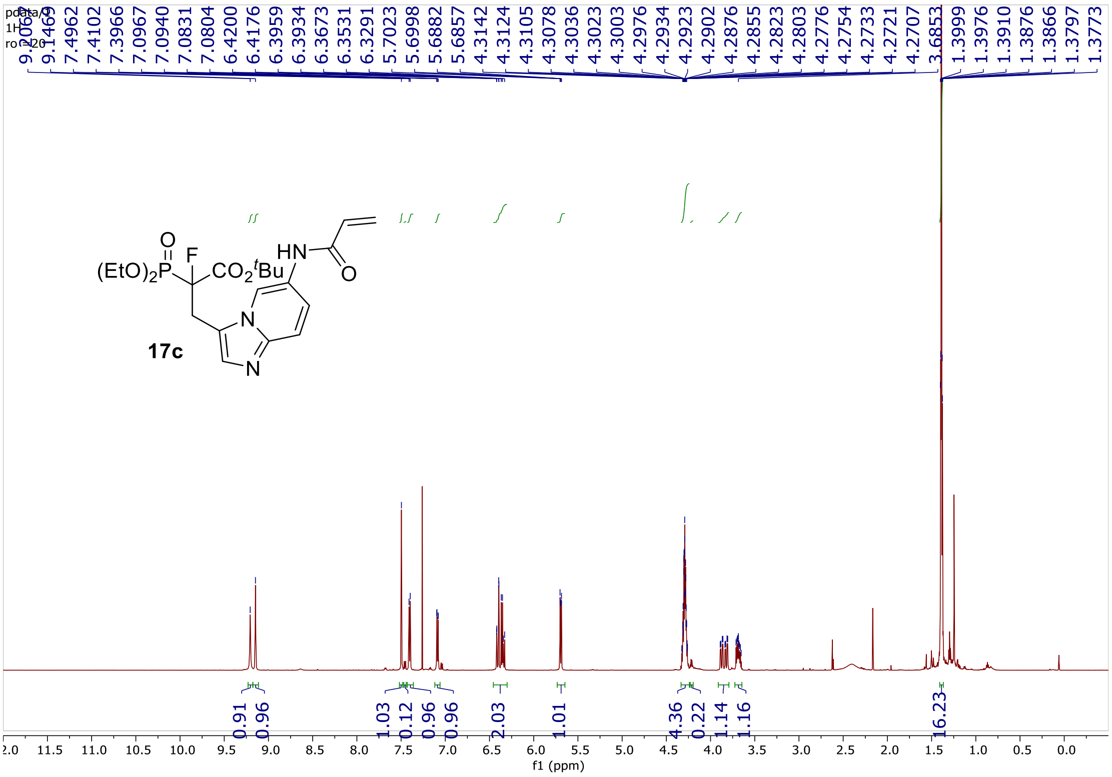

Figure S126. ^{1}H NMR (700 MHz, $CDCl_3$) of compound **17c**.

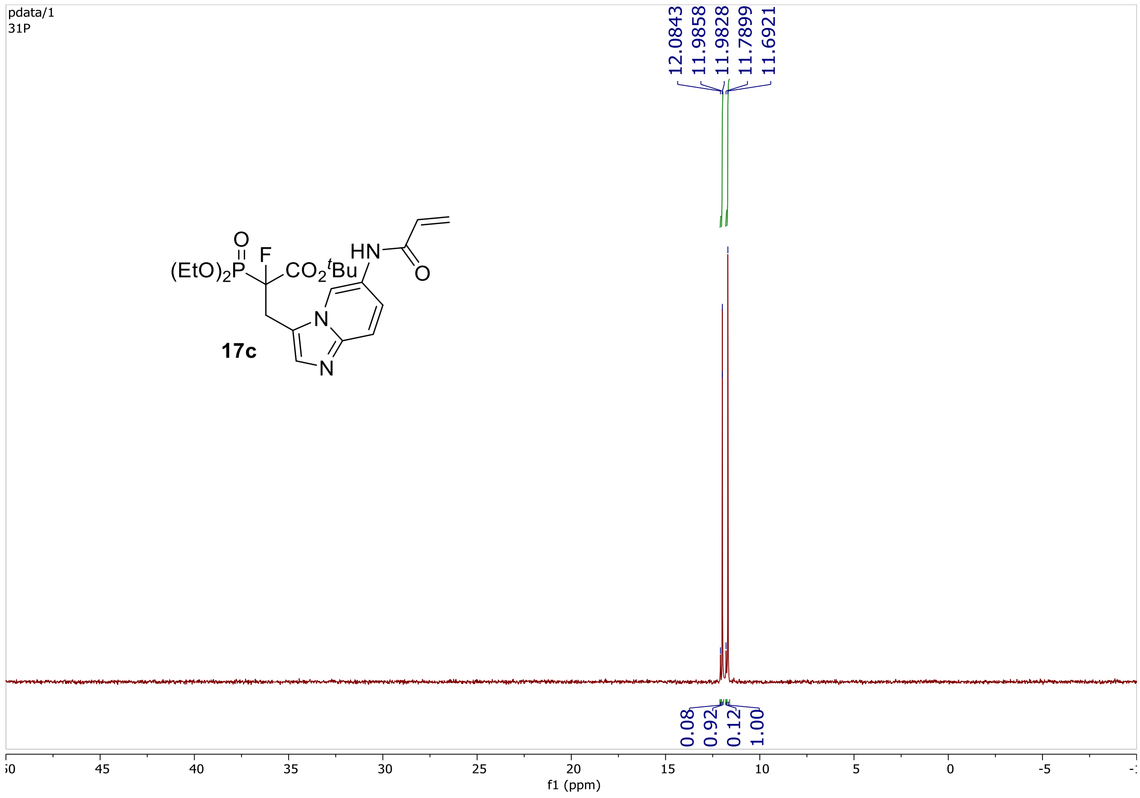

Figure S127. ^{31}P NMR (283 MHz, $CDCl_3$) of compound **17c**.

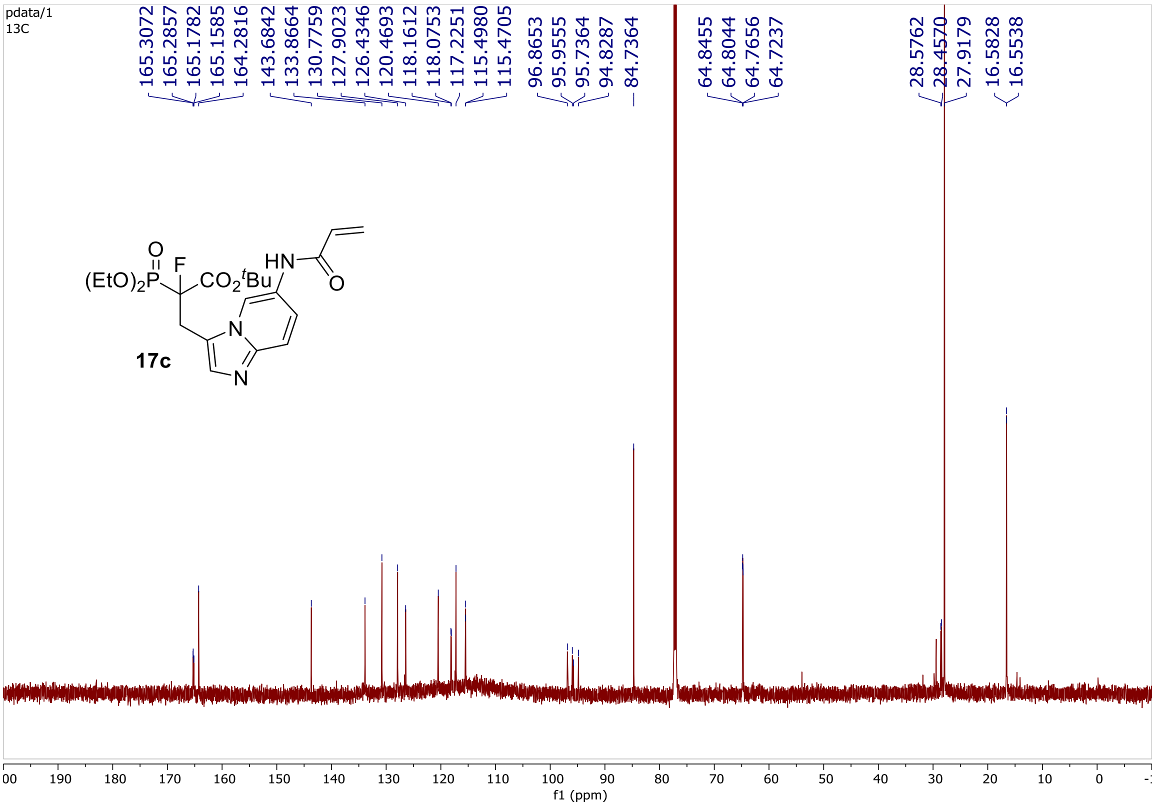

Figure S128. ^{13}C NMR (176 MHz, $CDCl_3$) of compound **17c**.

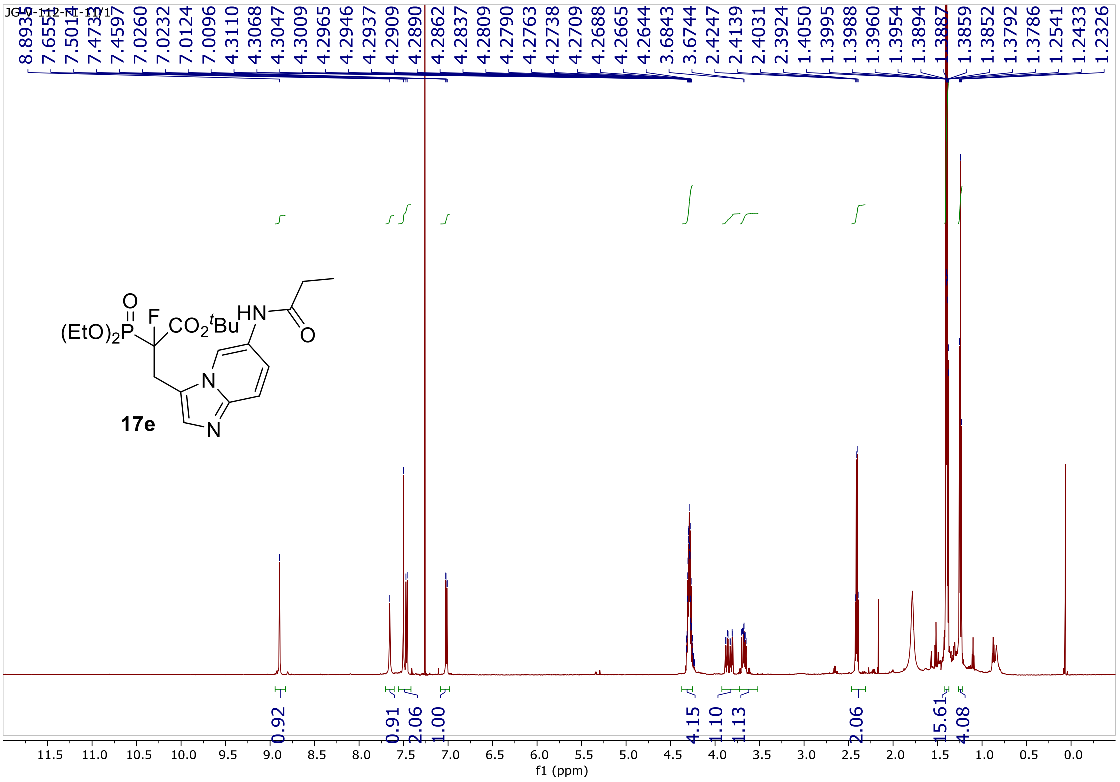

Figure S129. ^{1}H NMR (700 MHz, $CDCl_3$) of compound **17e**.

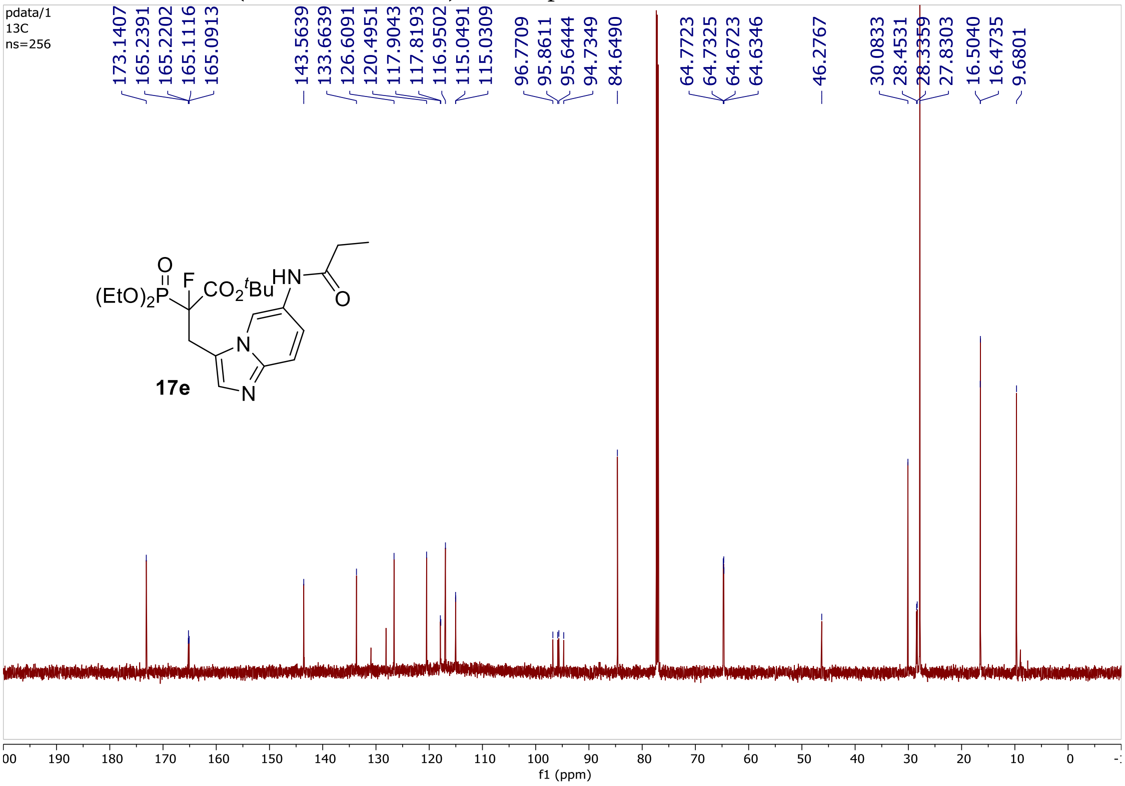

Figure S130. ^{13}C NMR (176 MHz, $CDCl_3$) of compound **17e**.

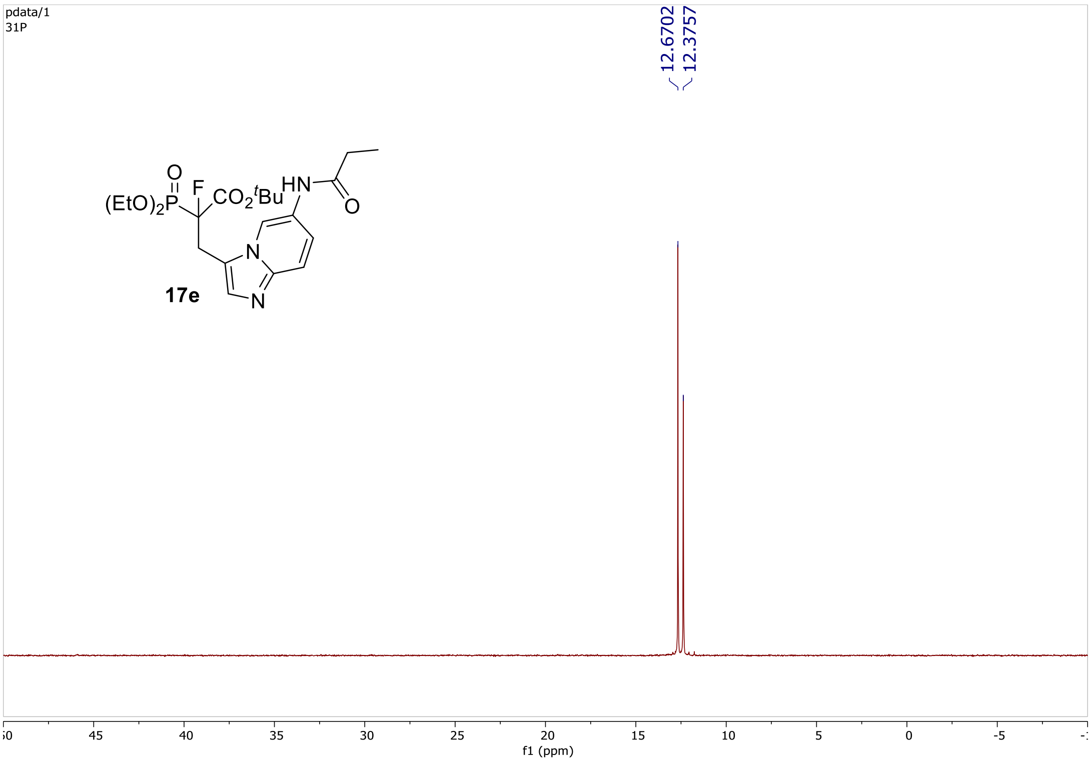

Figure S131. ^{31}P NMR (283 MHz, $CDCl_3$) of compound **17e**.

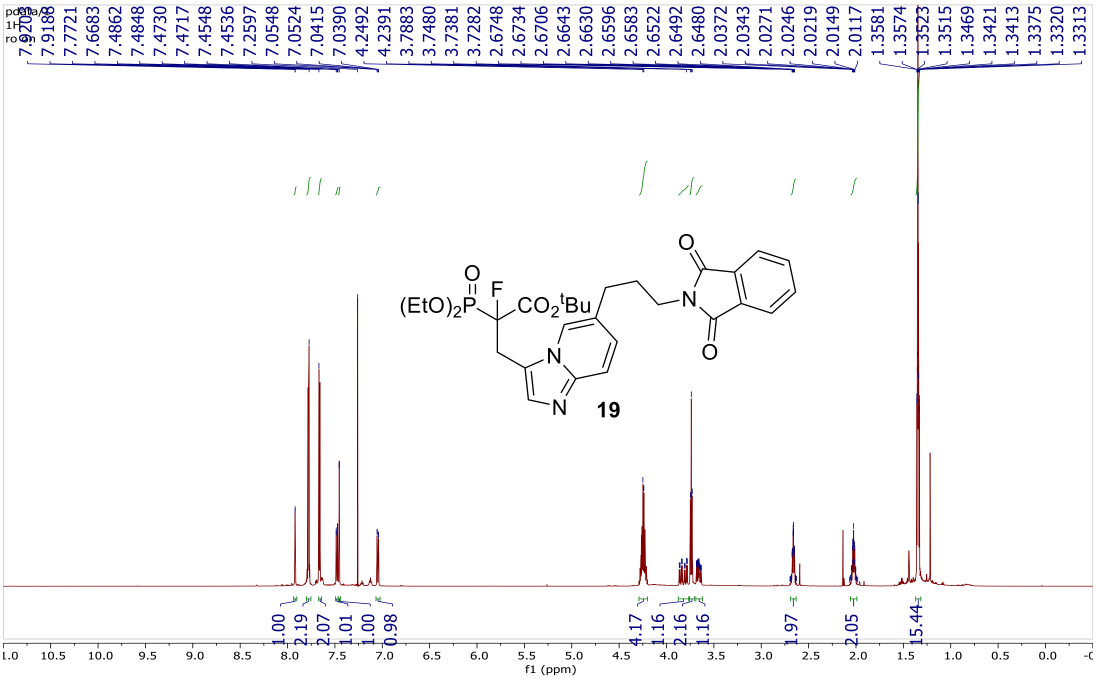

Figure S132. ^{1}H NMR (700 MHz, $CDCl_3$) of compound **19**.

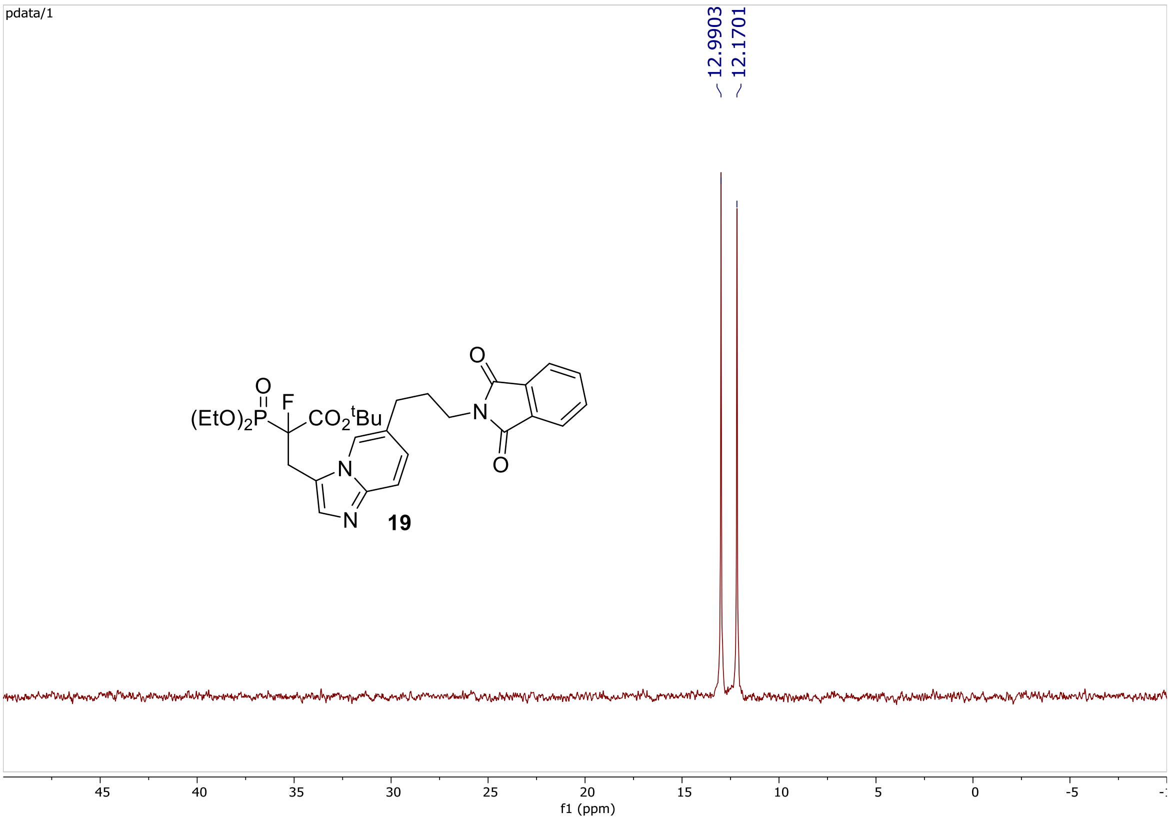

Figure S133. ^{31}P NMR (283 MHz, $CDCl_3$) of compound **19**.

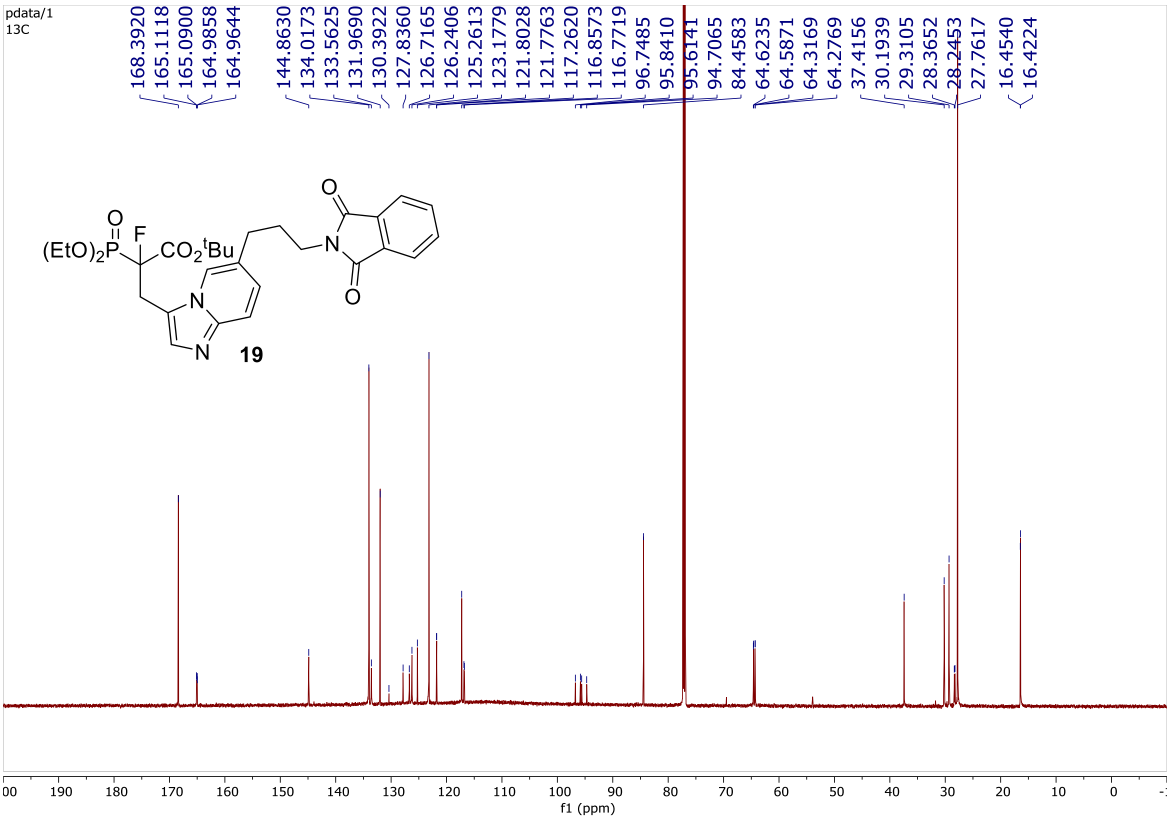

Figure S134. ^{13}C NMR (176 MHz, $CDCl_3$) of compound **19**.

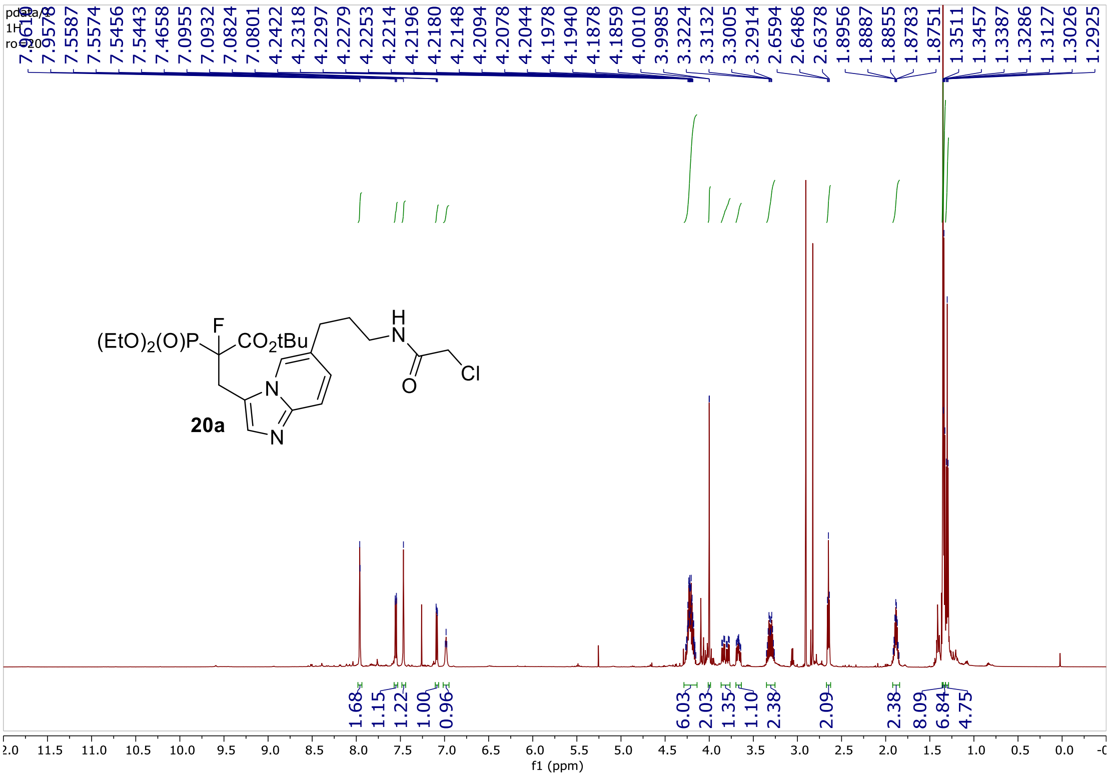

Figure S135. ^{1}H NMR (700 MHz, $CDCl_3$) of compound **20a**.

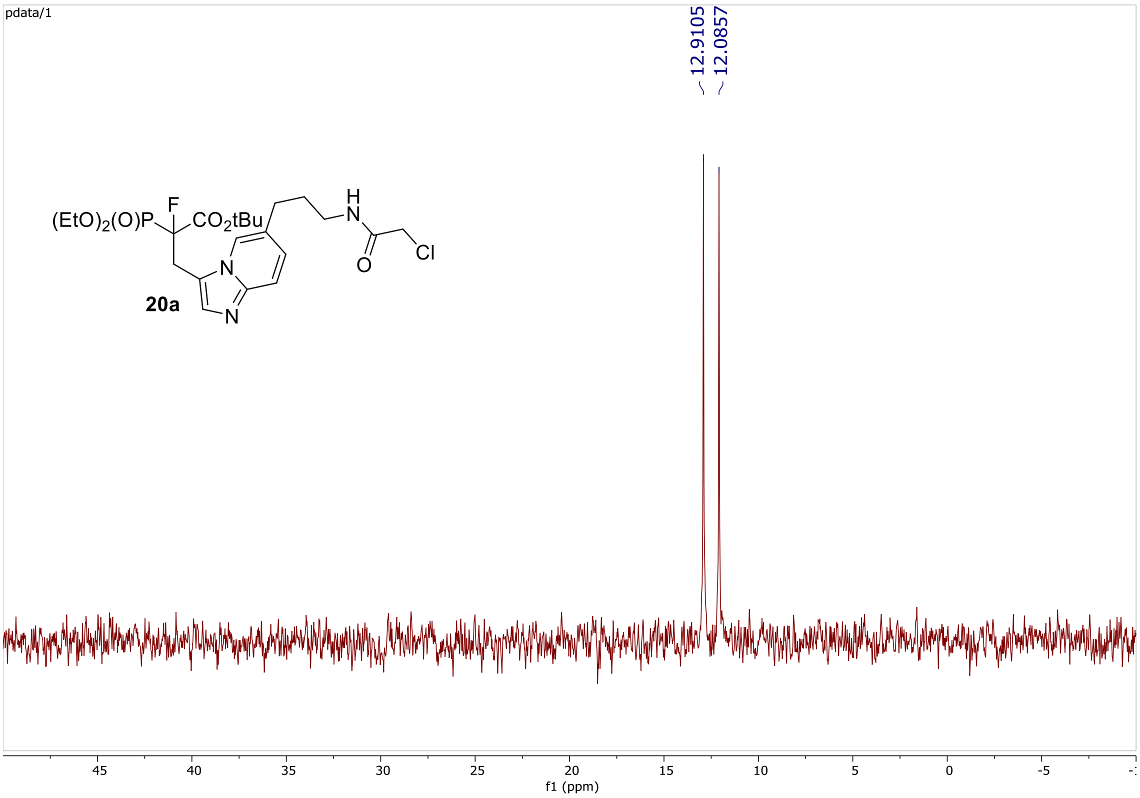

Figure S136. ^{31}P NMR (101 MHz, $CDCl_3$) of compound **20a**.

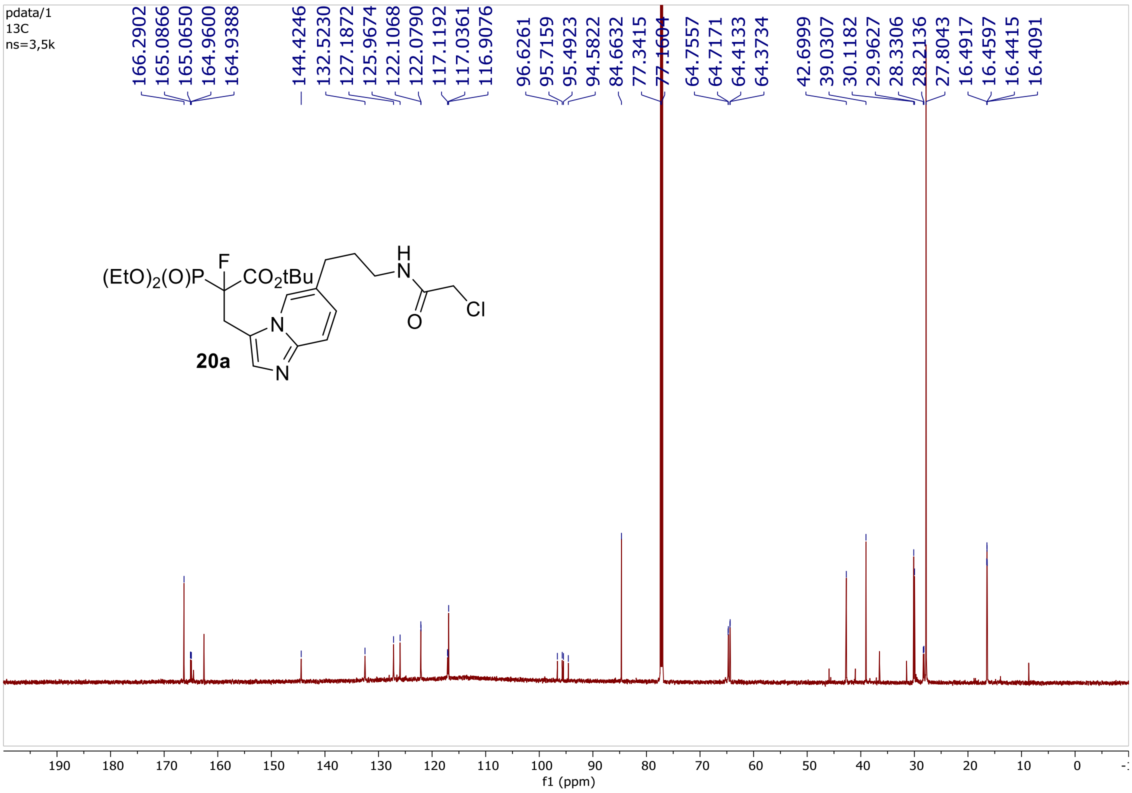

Figure S137. ^{13}C NMR (176 MHz, $CDCl_3$) of compound **20a**.

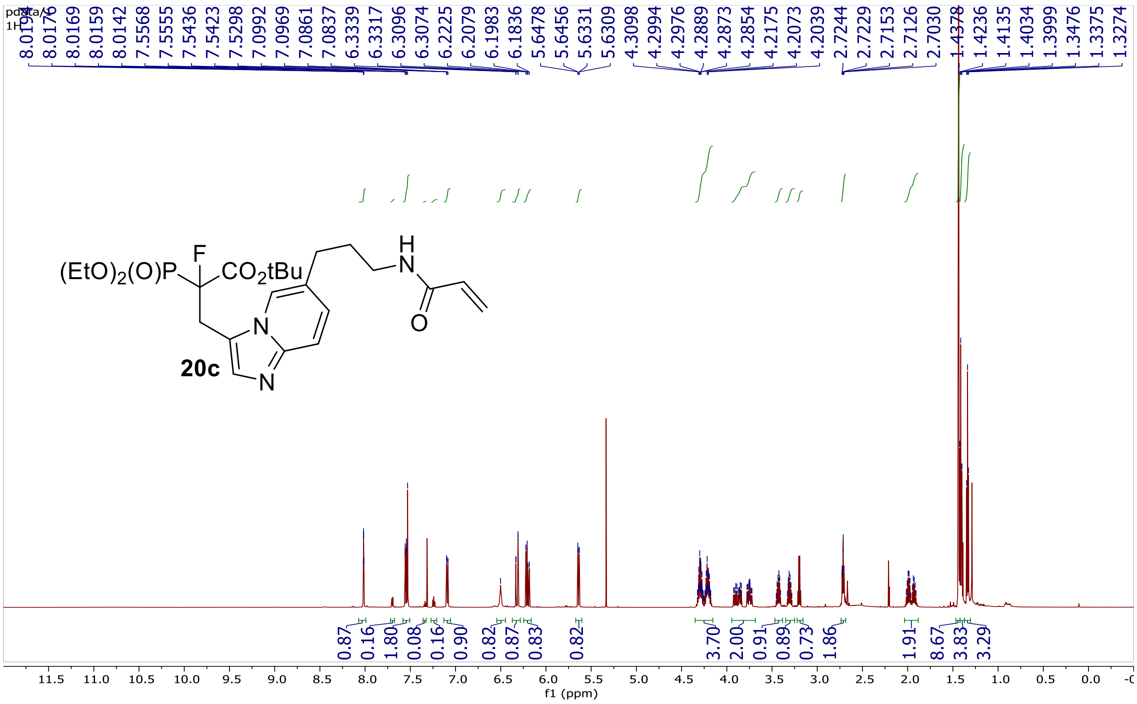

Figure S138. ^{1}H NMR (700 MHz, $CDCl_3$) of compound **20c**.

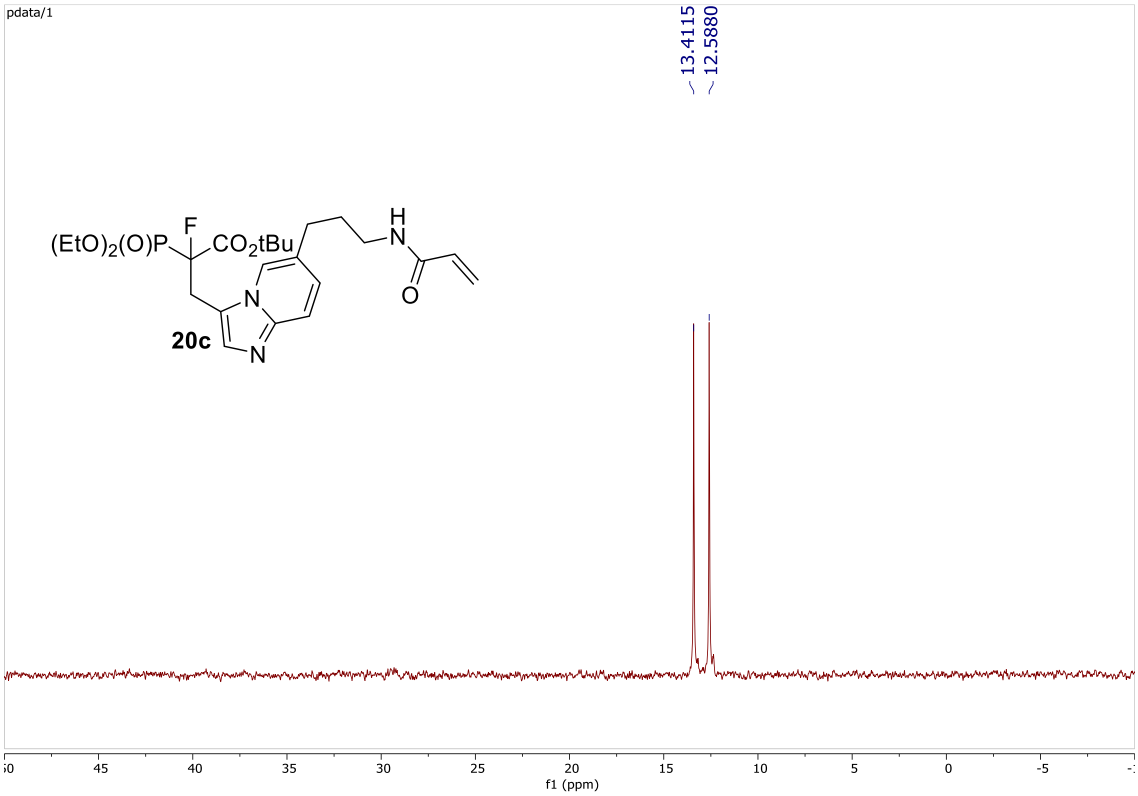

Figure S139. ^{31}P NMR (101 MHz, $CDCl_3$) of compound **20c**.

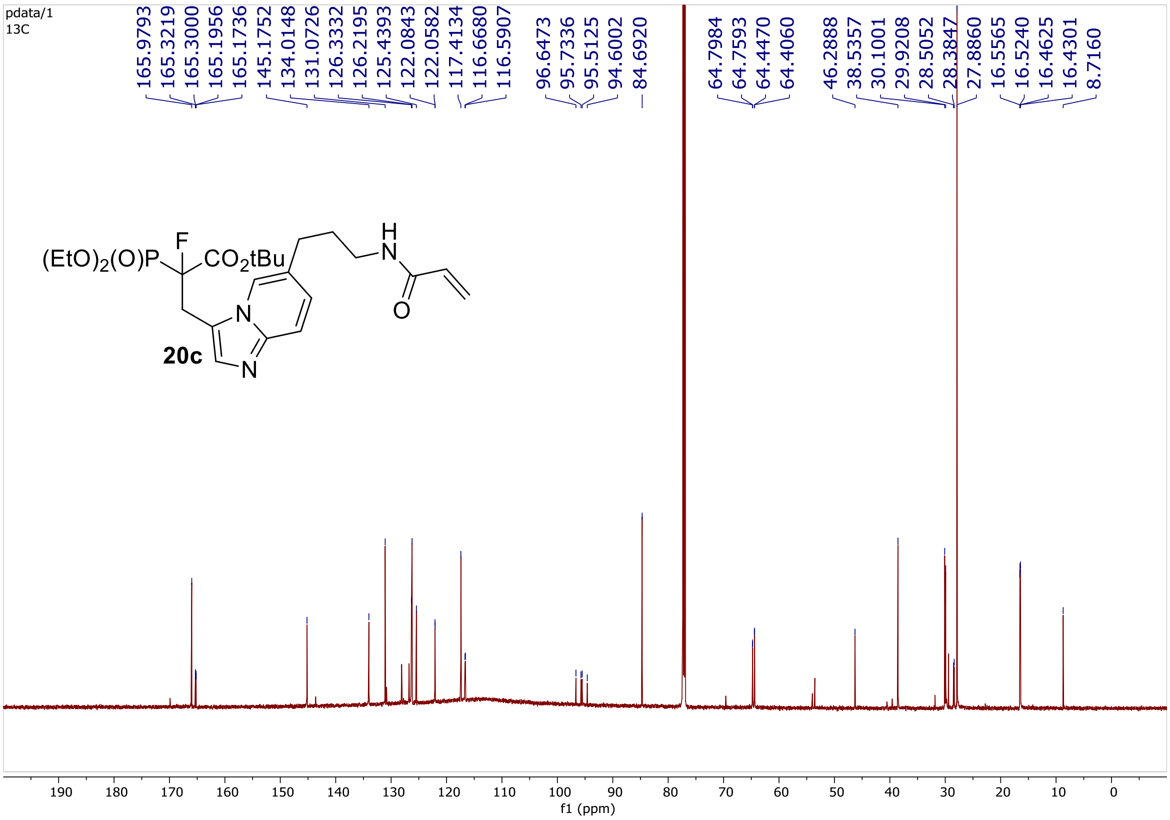

Figure S140. ^{13}C NMR (176 MHz, $CDCl_3$) of compound **20c**.

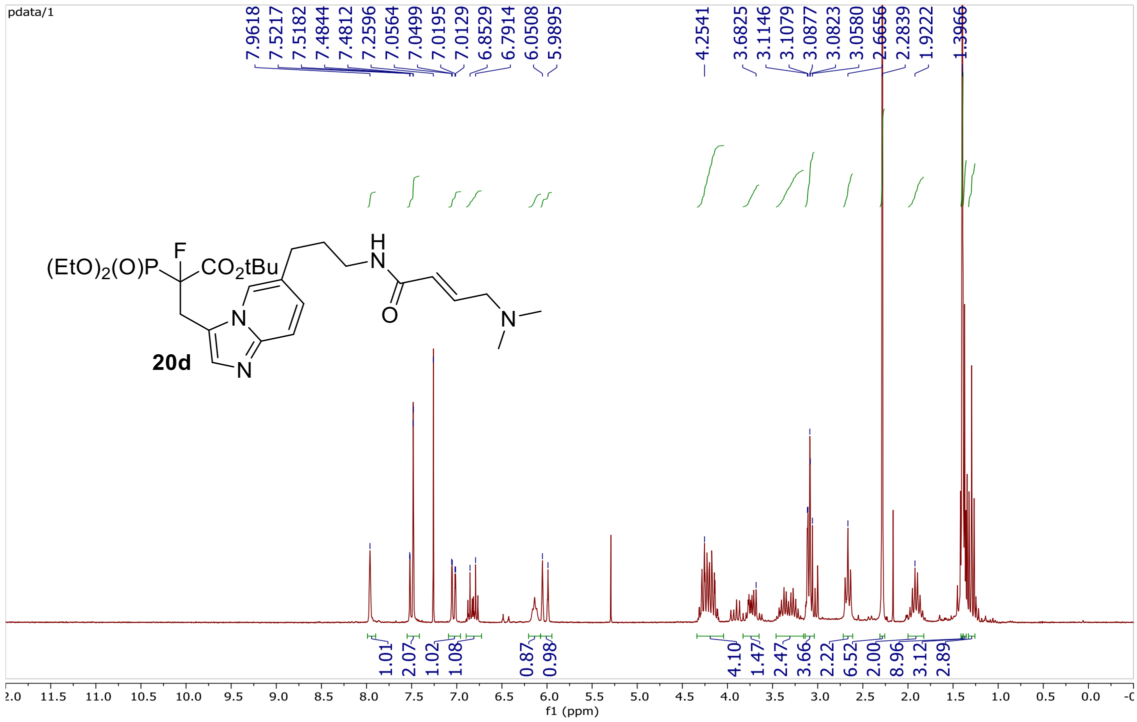

Figure S141. ^{1}H NMR (250 MHz, $CDCl_3$) of compound **20d**.

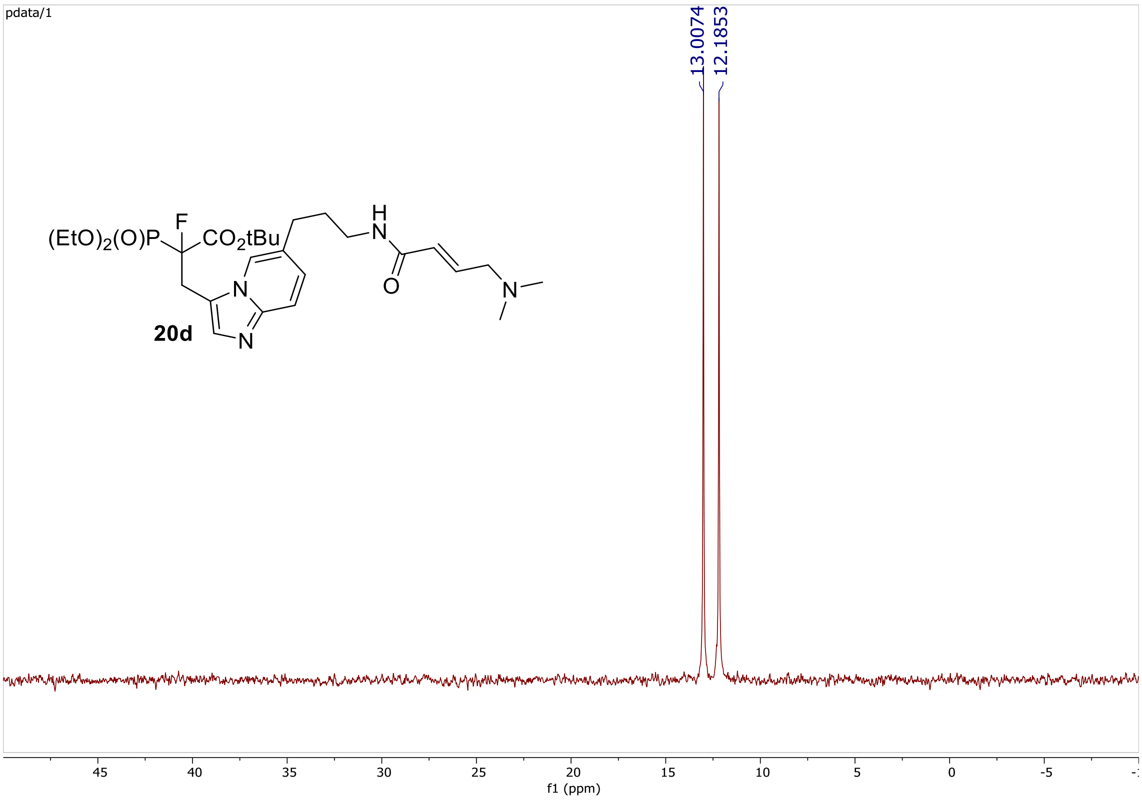

Figure S142. ^{31}P NMR (101 MHz, $CDCl_3$) of compound **20d**.

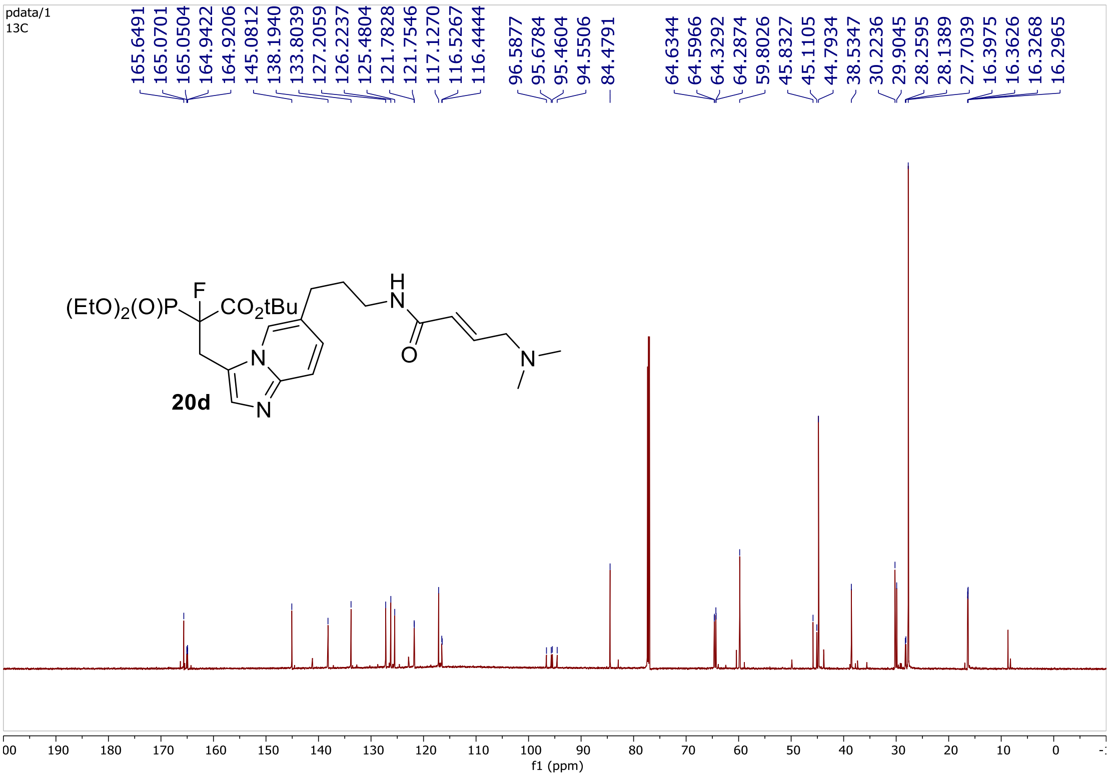

Figure S143. ^{13}C NMR (176 MHz, $CDCl_3$) of compound **20d**.

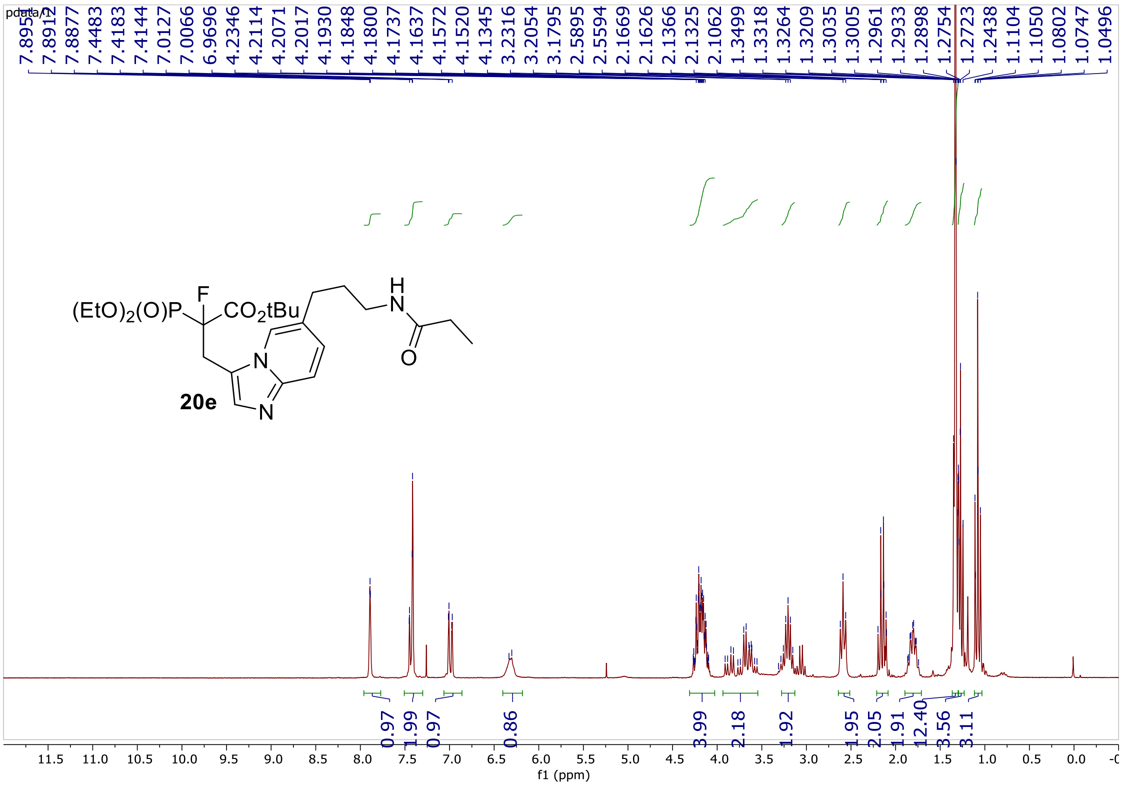

Figure S144. ^{1}H NMR (250 MHz, $CDCl_3$) of compound **20e**.

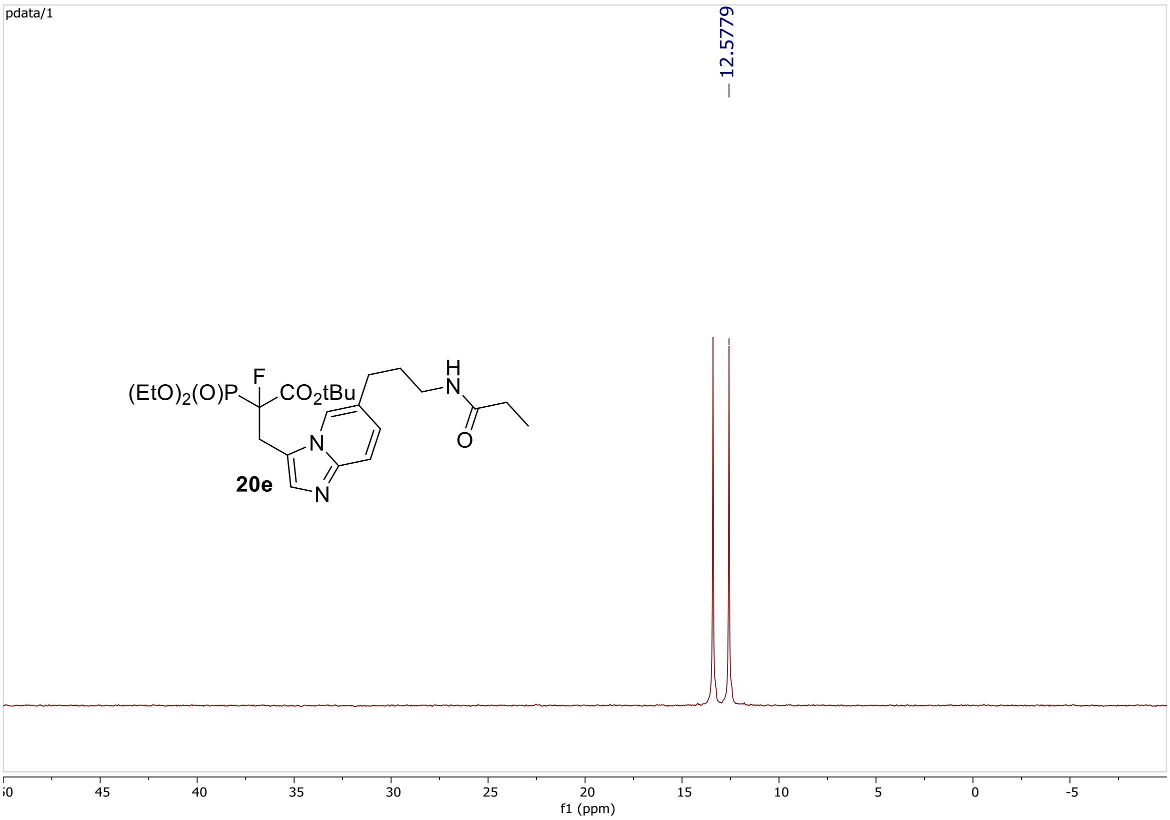

Figure S145. ^{31}P NMR (101 MHz, $CDCl_3$) of compound **20e**.

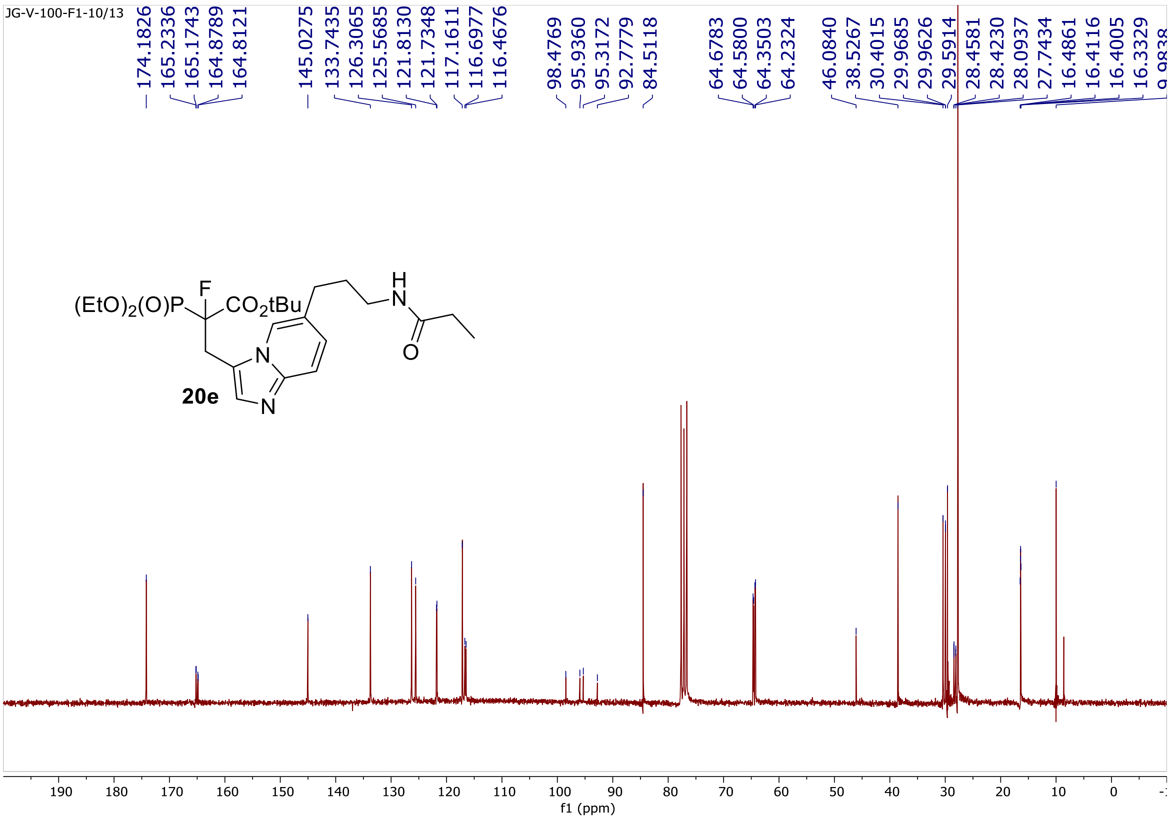

Figure S146. ^{13}C NMR (63 MHz, $CDCl_3$) of compound **20e**.

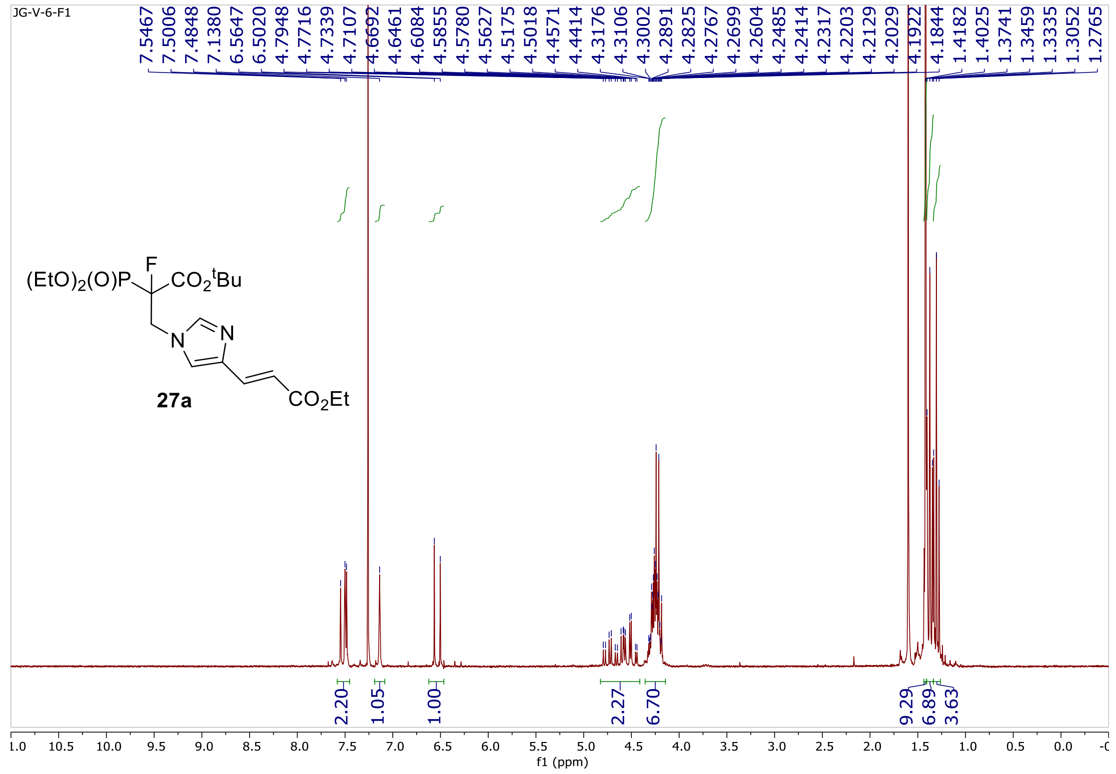

Figure S147. ^{1}H NMR (700 MHz, $CDCl_3$) of compound **27a**.

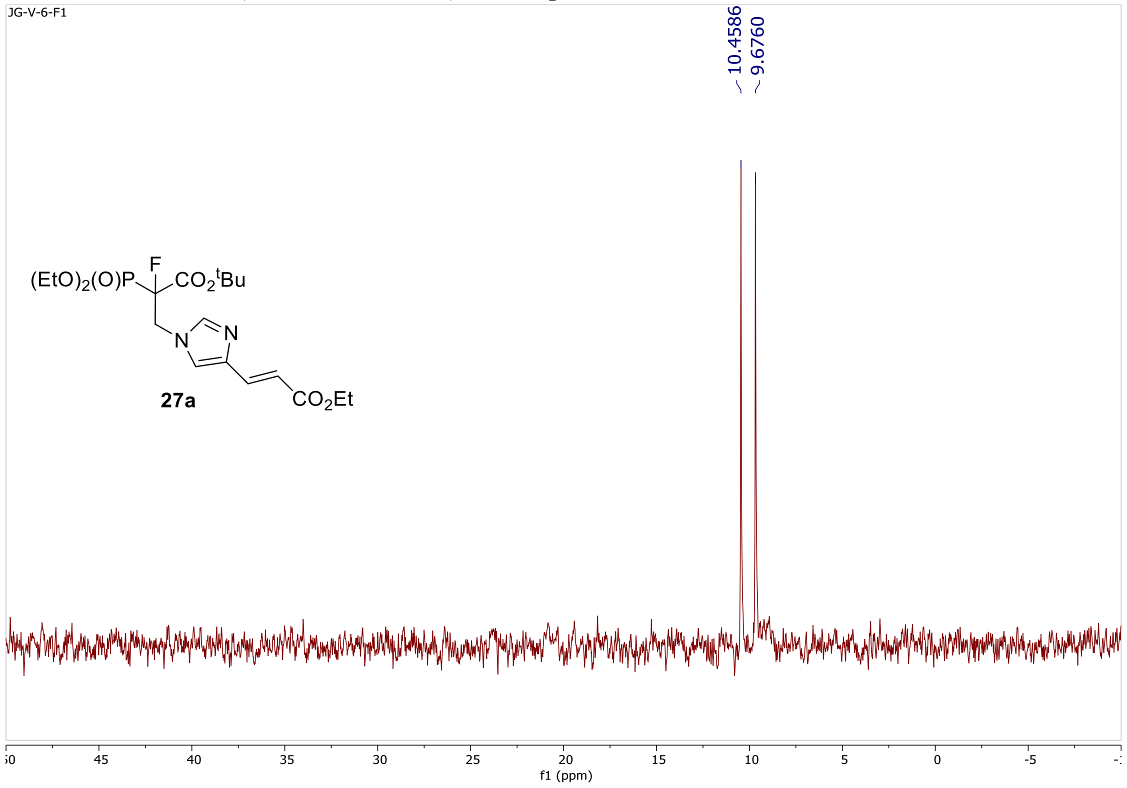

Figure S148. ^{31}P NMR (101 MHz, $CDCl_3$) of compound **27a**.

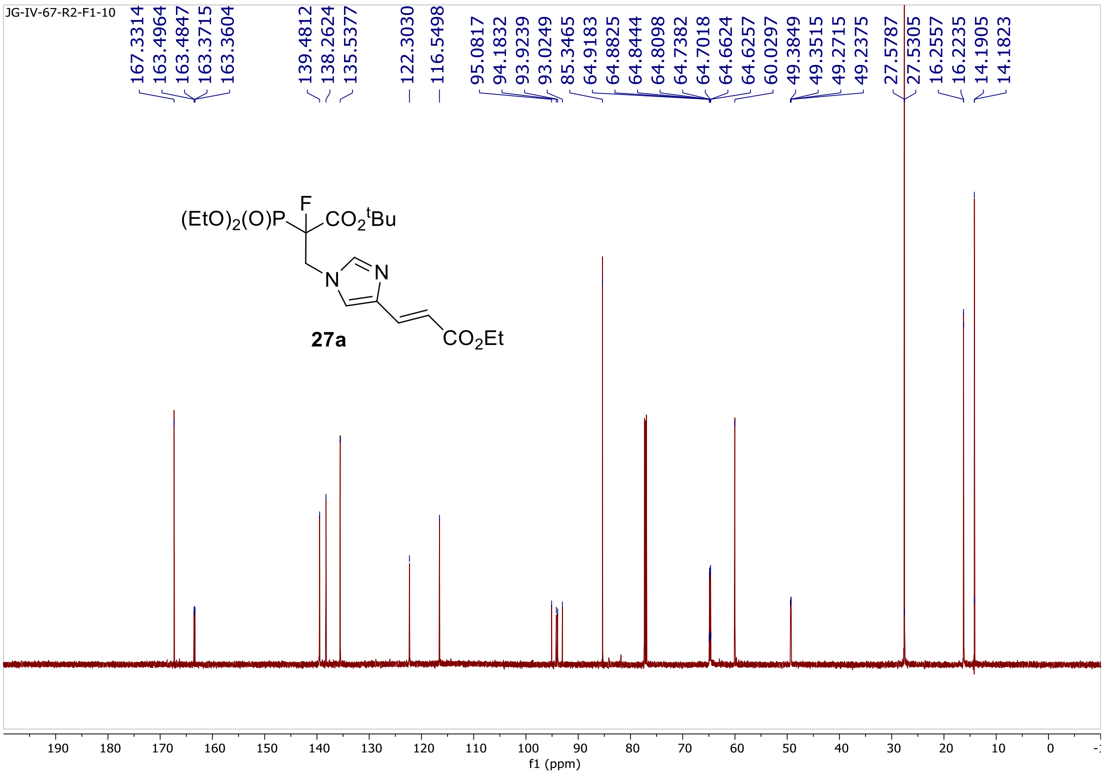

Figure S149. ^{13}C NMR (176 MHz, $CDCl_3$) of compound **27a**.

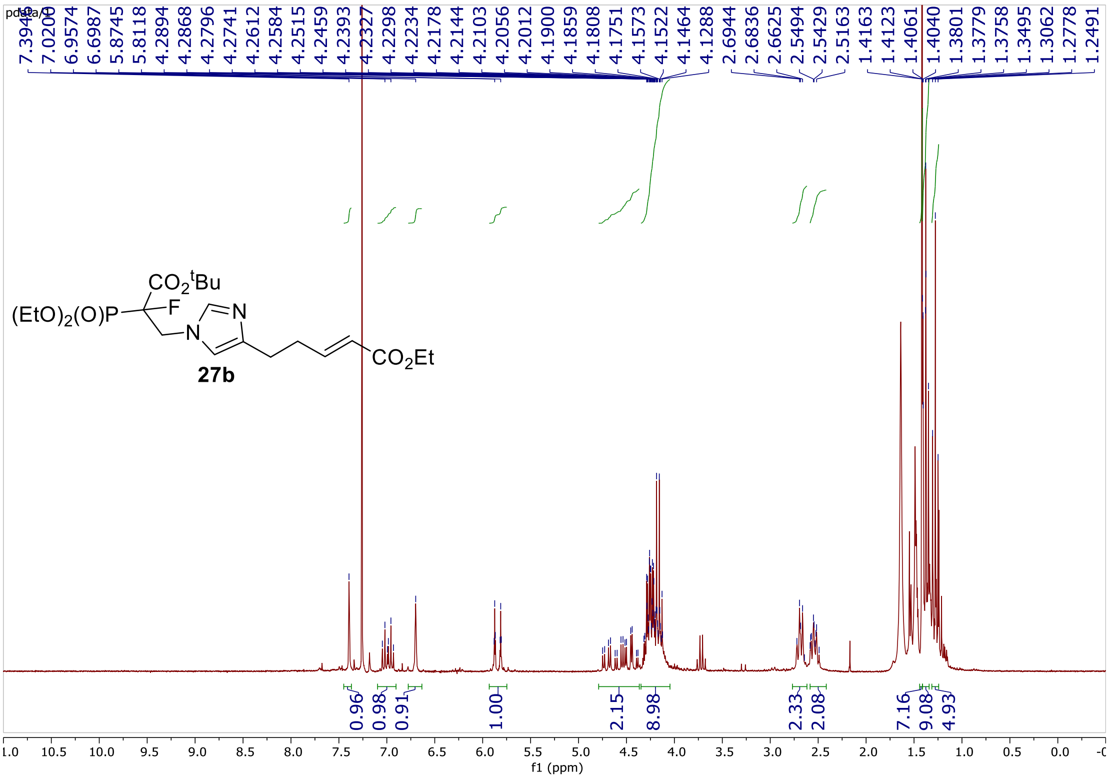

Figure S150. ^{1}H NMR (700 MHz, $CDCl_3$) of compound **27b** (mixture of regioisomers C4 and C5).

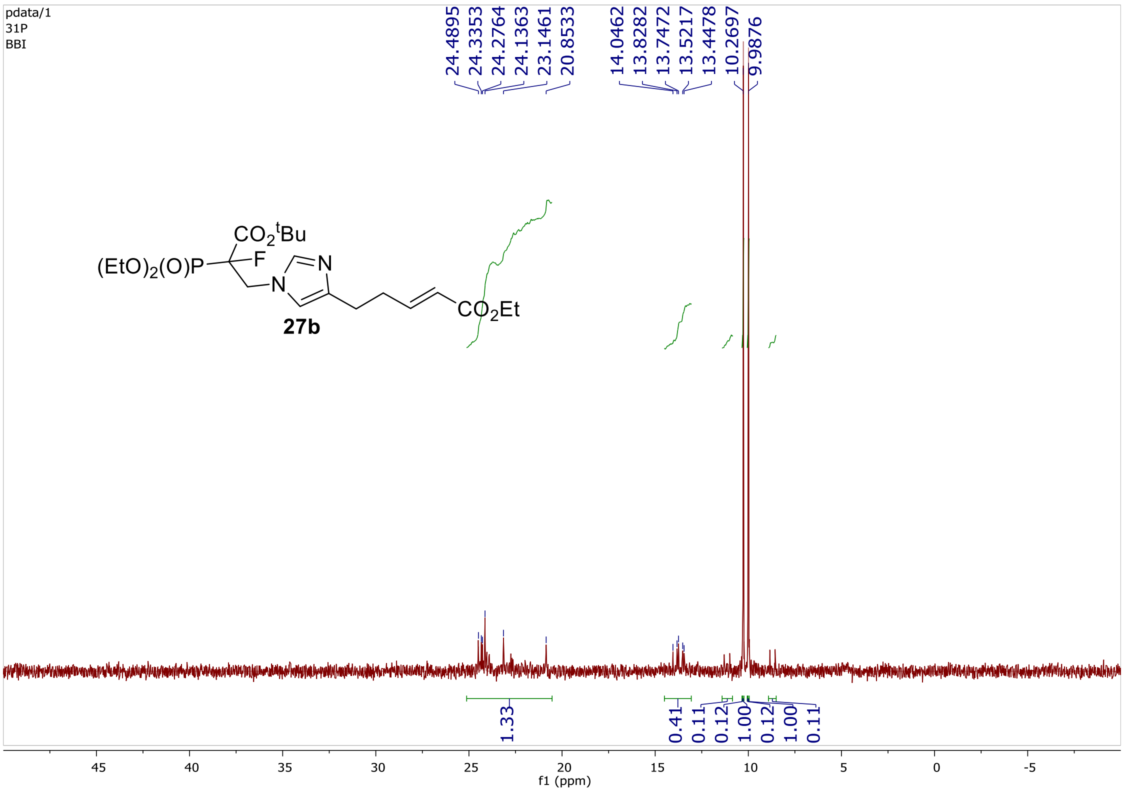

Figure S151. ^{31}P NMR (283 MHz, $CDCl_3$) of compound **27b** (mixture of regioisomers C4 and C5).

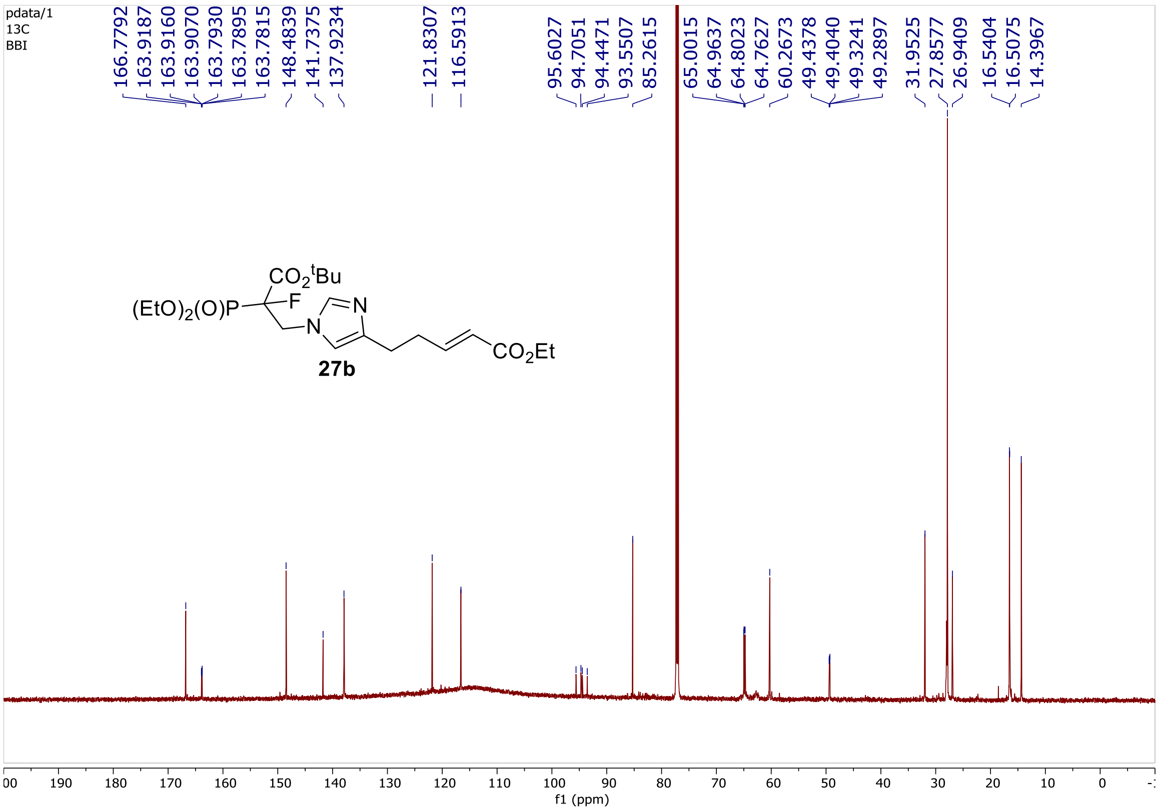

Figure S152. ^{13}C NMR (176 MHz, $CDCl_3$) of compound **27b** (mixture of regioisomers C4 and C5).

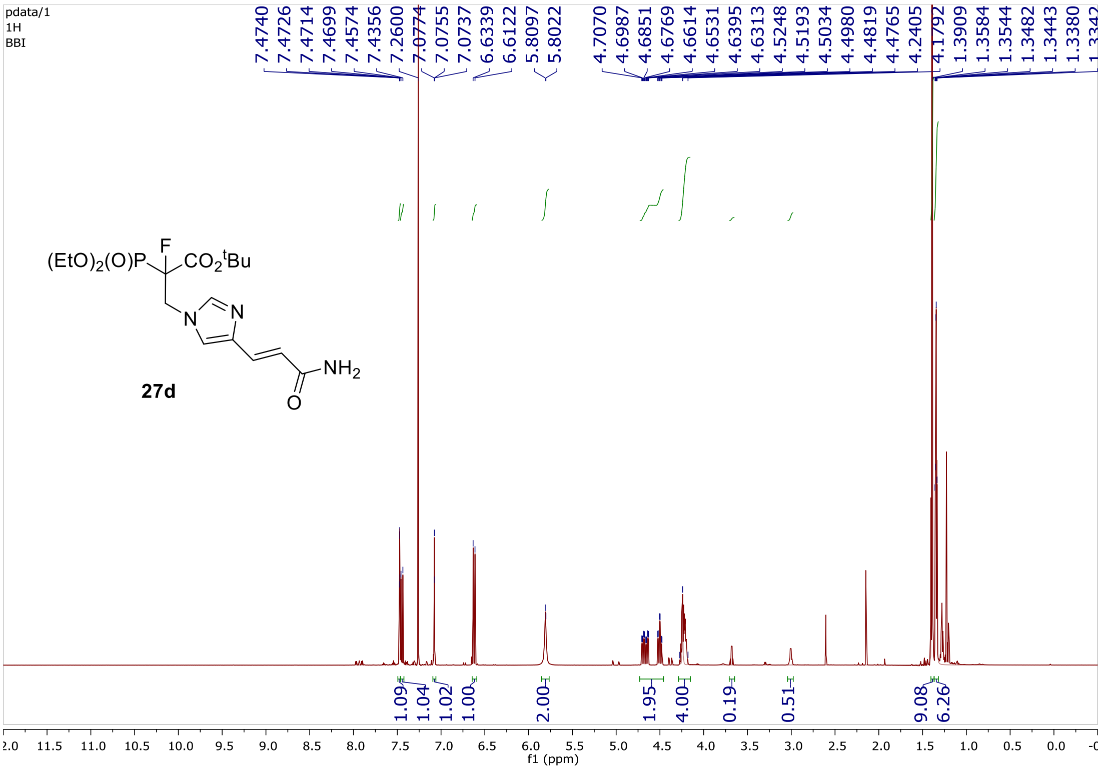

Figure S153. ^{1}H NMR (700 MHz, $CDCl_3$) of compound **27d**.

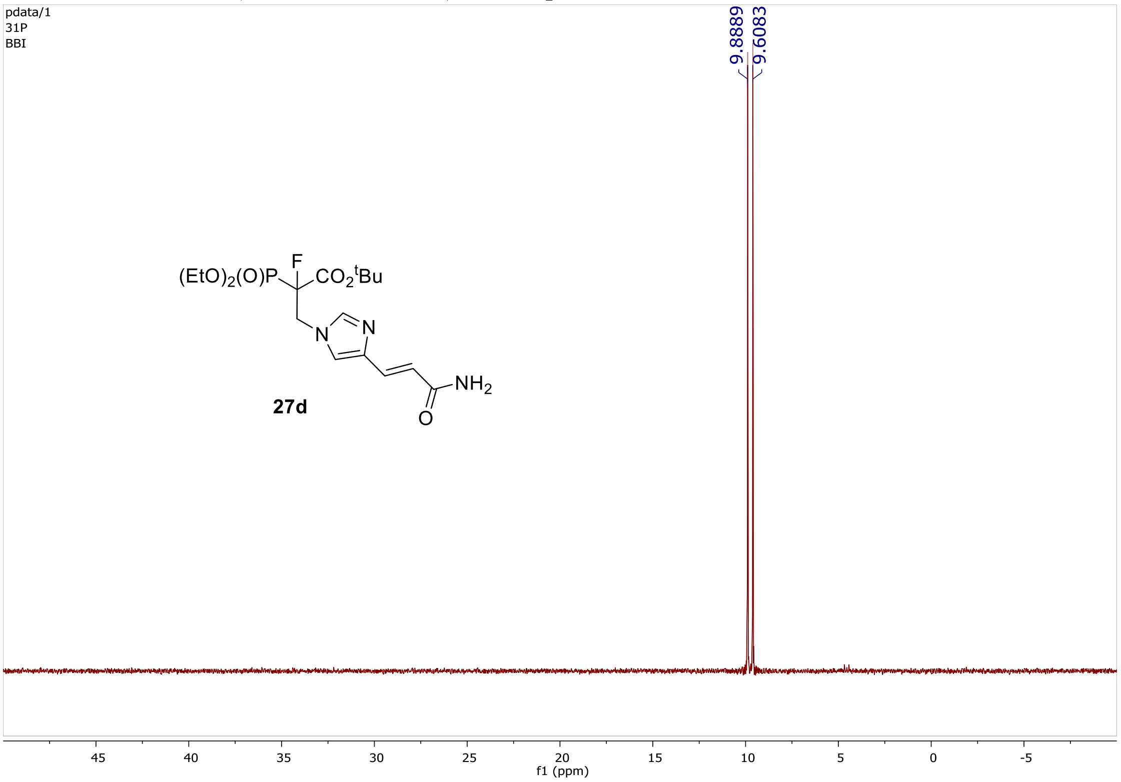

Figure S154. ^{31}P NMR (283 MHz, $CDCl_3$) of compound **27d**.

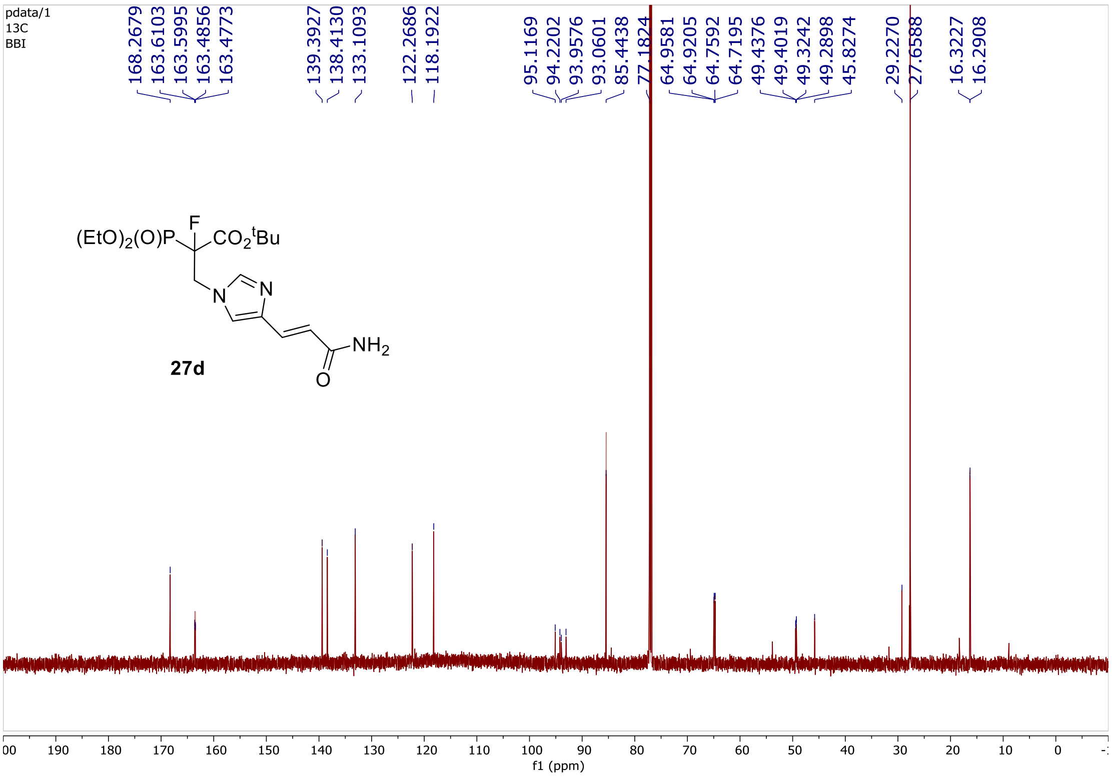

Figure S155. ^{13}C NMR (176 MHz, $CDCl_3$) of compound **27d**.

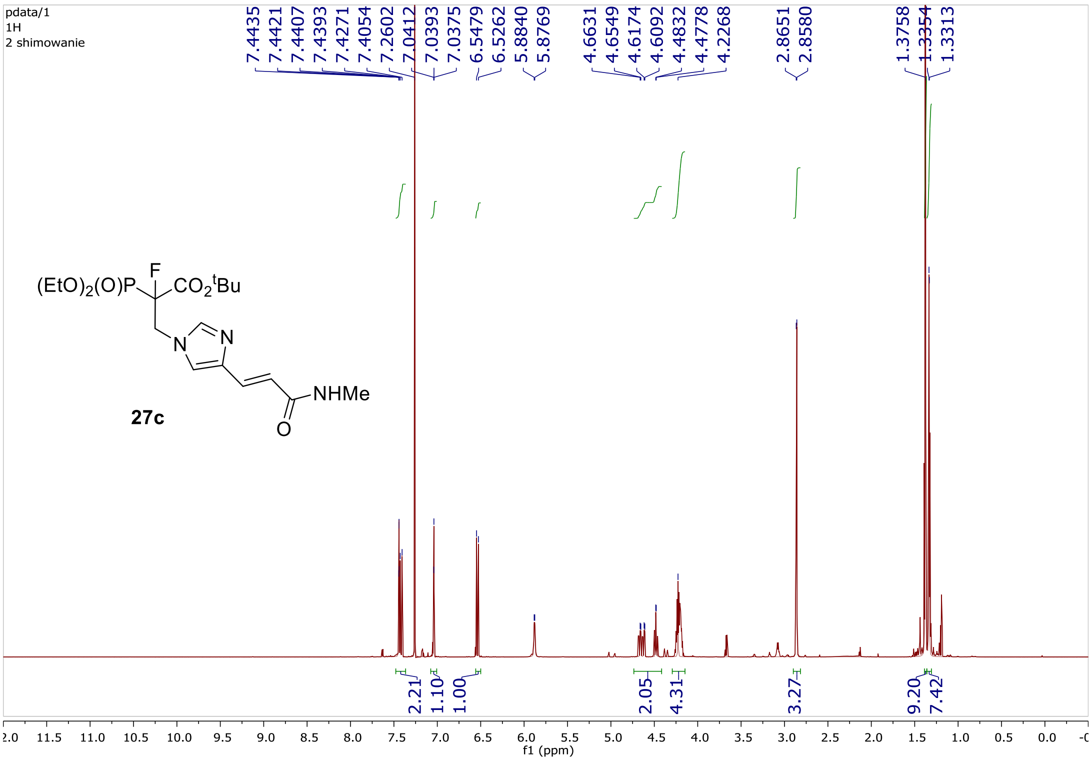

Figure S156. ^{1}H NMR (700 MHz, $CDCl_3$) of compound **27c**.

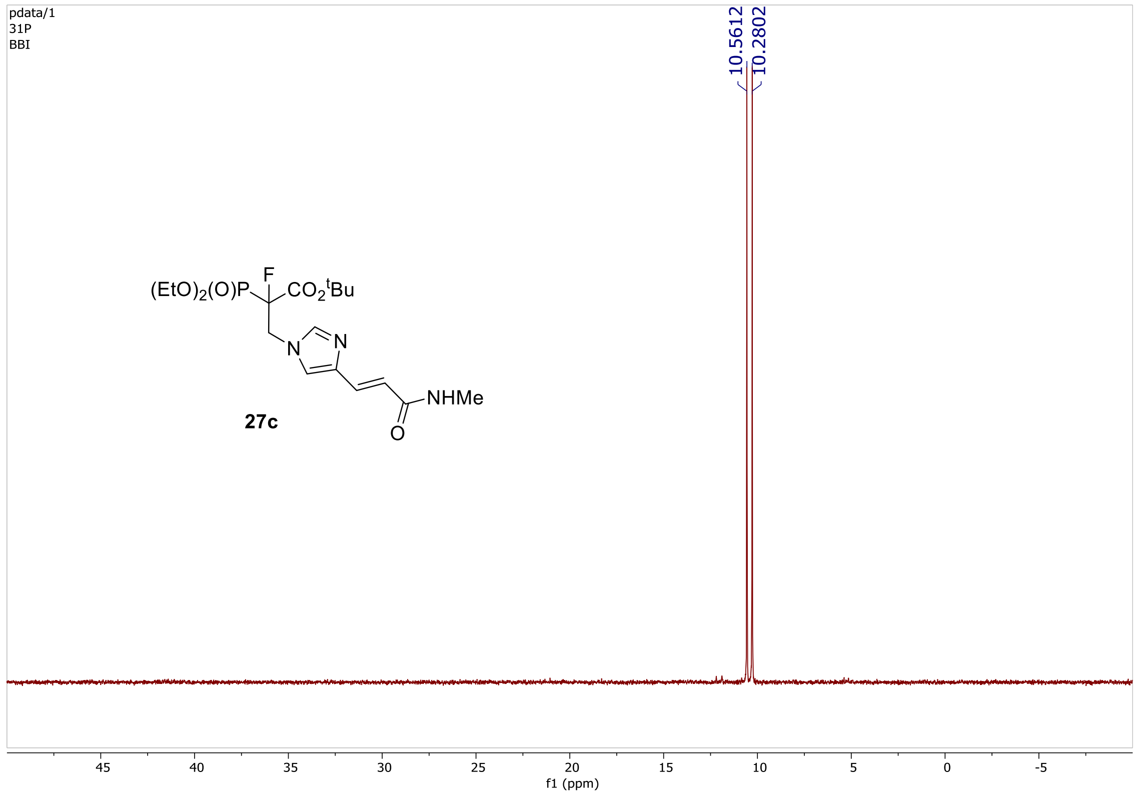

Figure S157. ^{31}P NMR (283 MHz, $CDCl_3$) of compound **27c**.

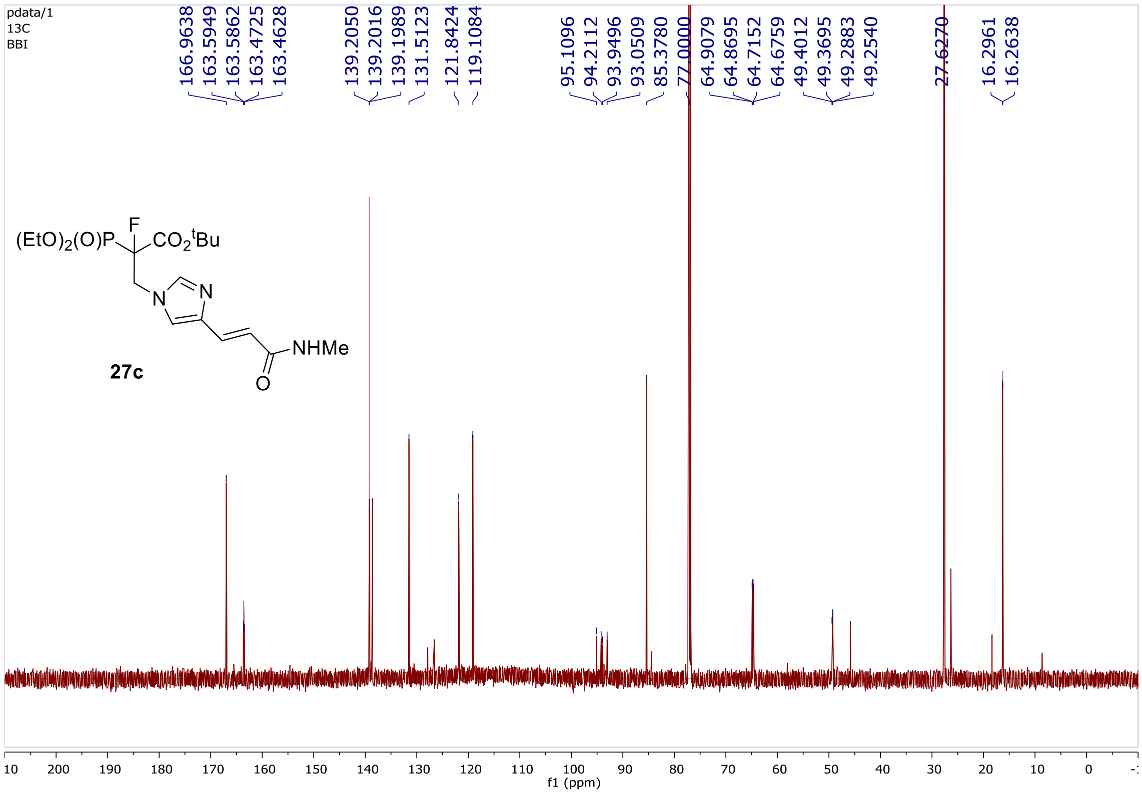

Figure S158. ^{13}C NMR (176 MHz, $CDCl_3$) of compound **27c**.

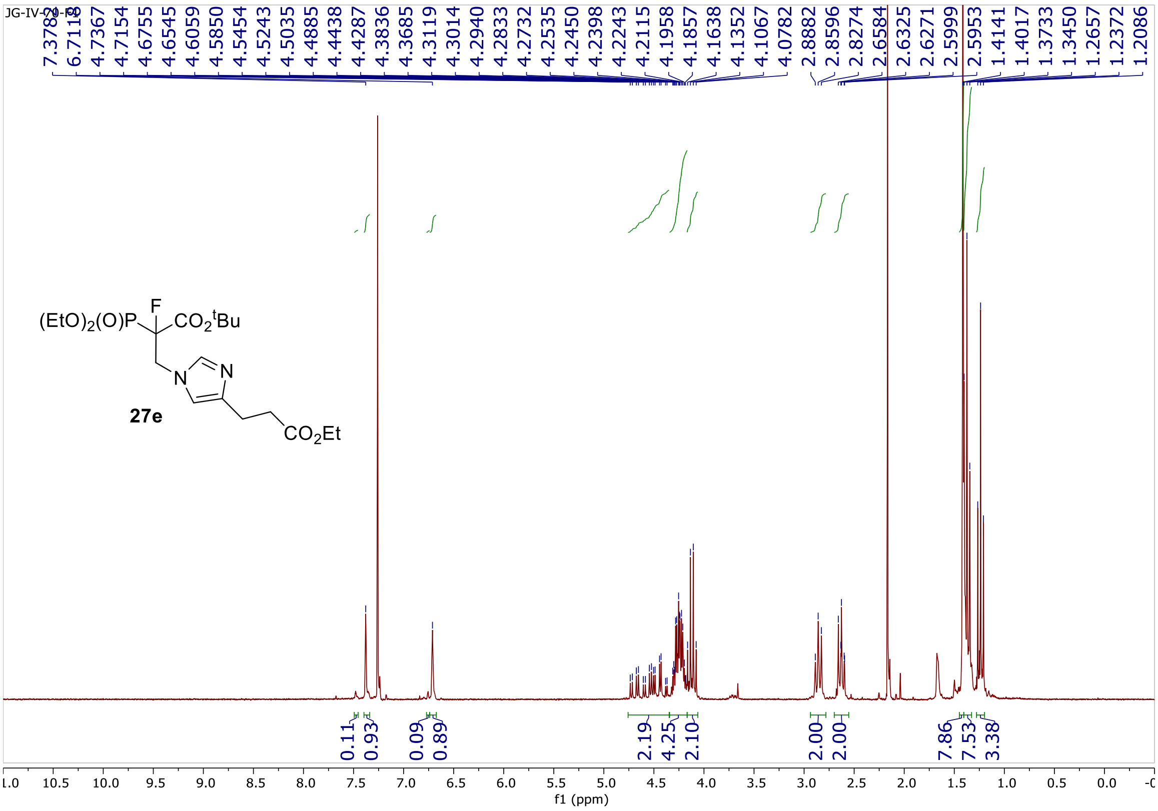

Figure S159. ^{1}H NMR (700 MHz, $CDCl_3$) of compound **27e**.

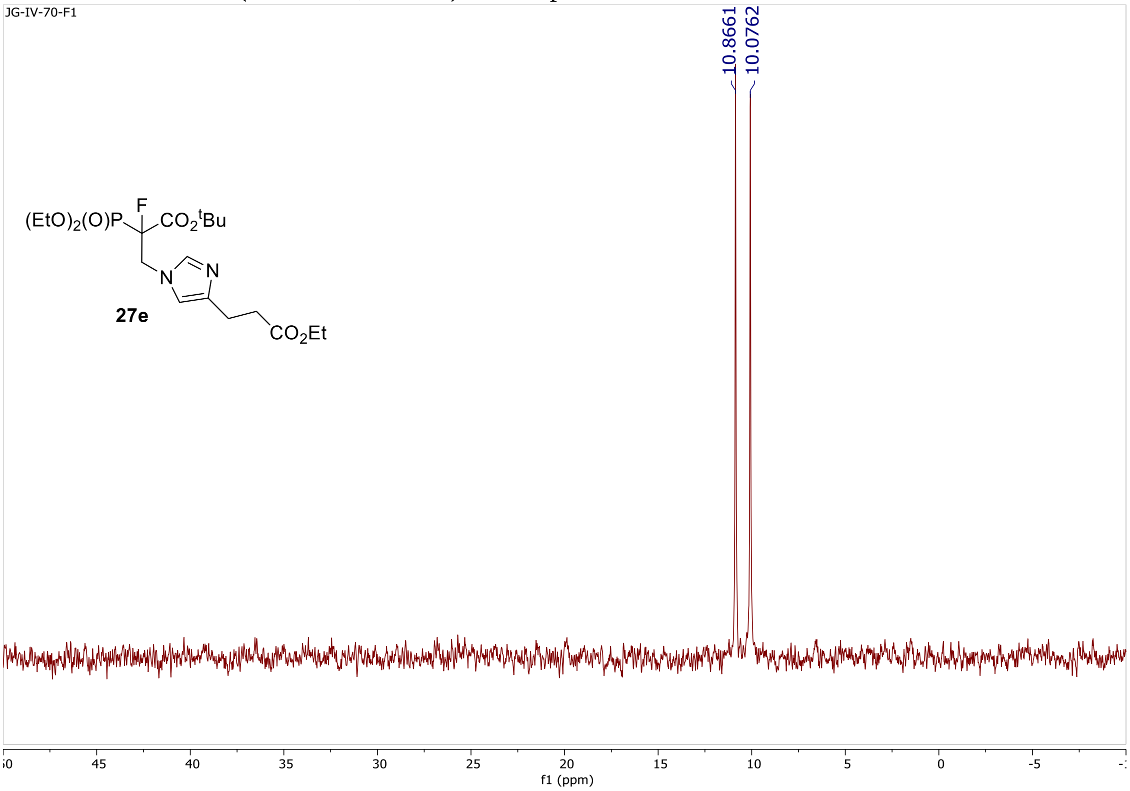

Figure S160. ^{31}P NMR (101 MHz, $CDCl_3$) of compound **27e**.

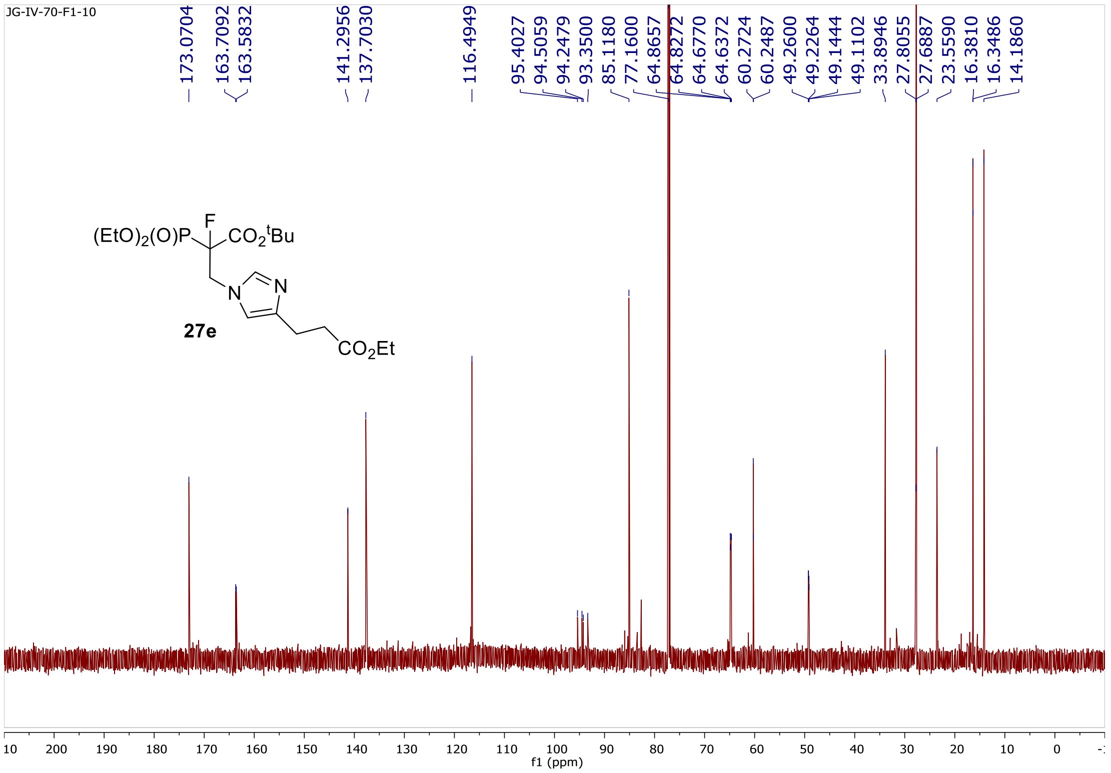

Figure S161. ^{13}C NMR (176 MHz, $CDCl_3$) of compound **27e**.

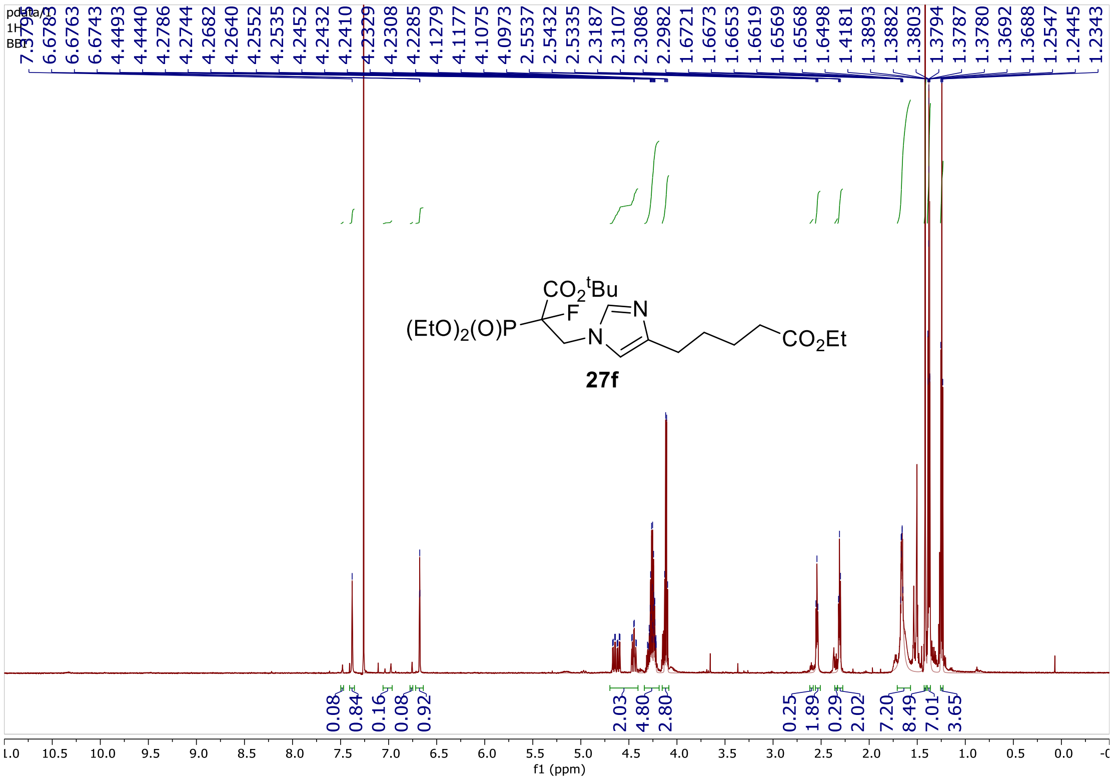

Figure S162. ^{1}H NMR (700 MHz, $CDCl_3$) of compound **27f** (mixture of regioisomers C4 and C5).

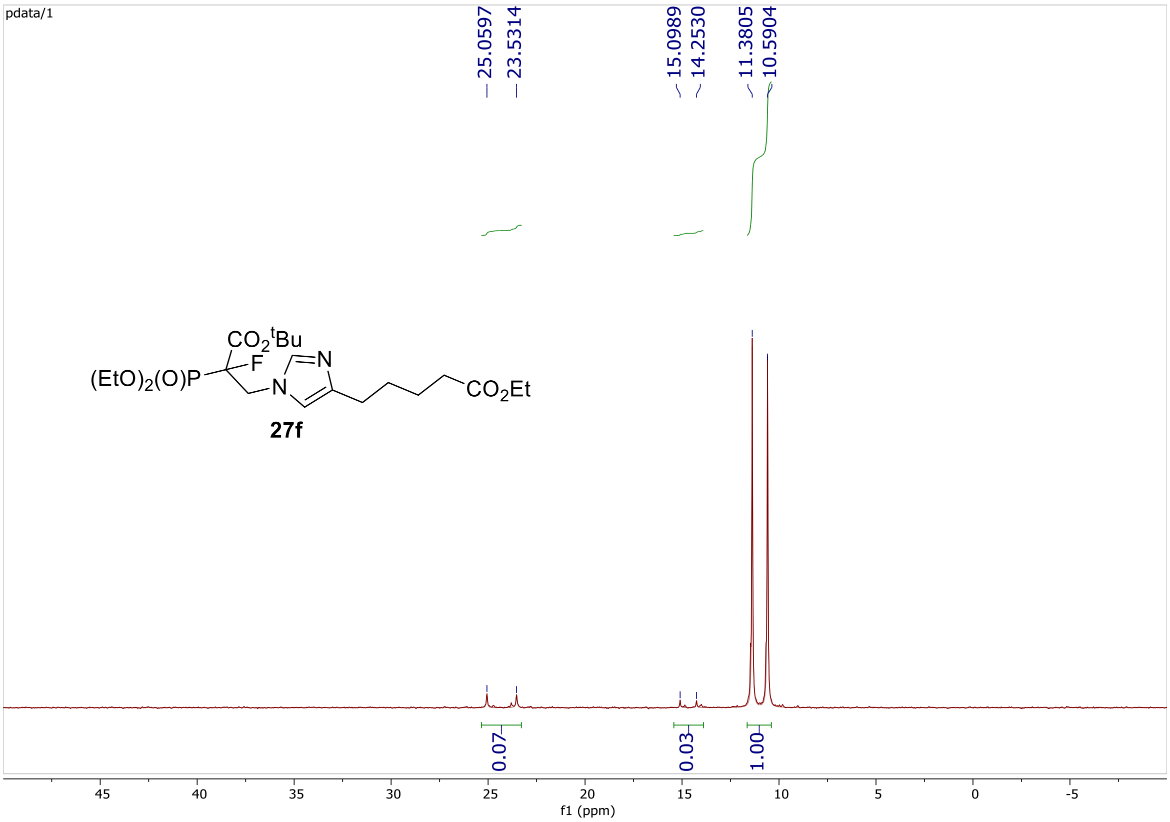

Figure S163. ^{31}P NMR (101 MHz, $CDCl_3$) of compound **27f** (mixture of regioisomers C4 and C5).

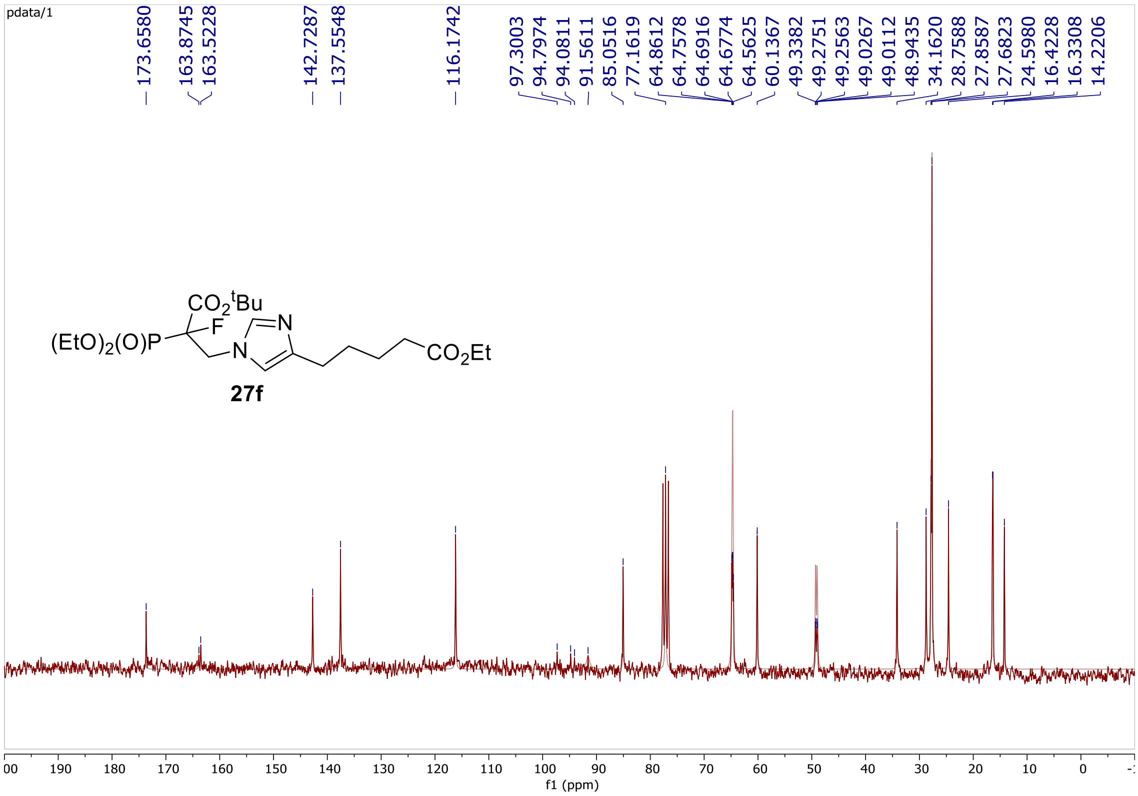

Figure S164. ^{13}C NMR (63 MHz, $CDCl_3$) of compound **27f** (mixture of regioisomers C4 and C5).

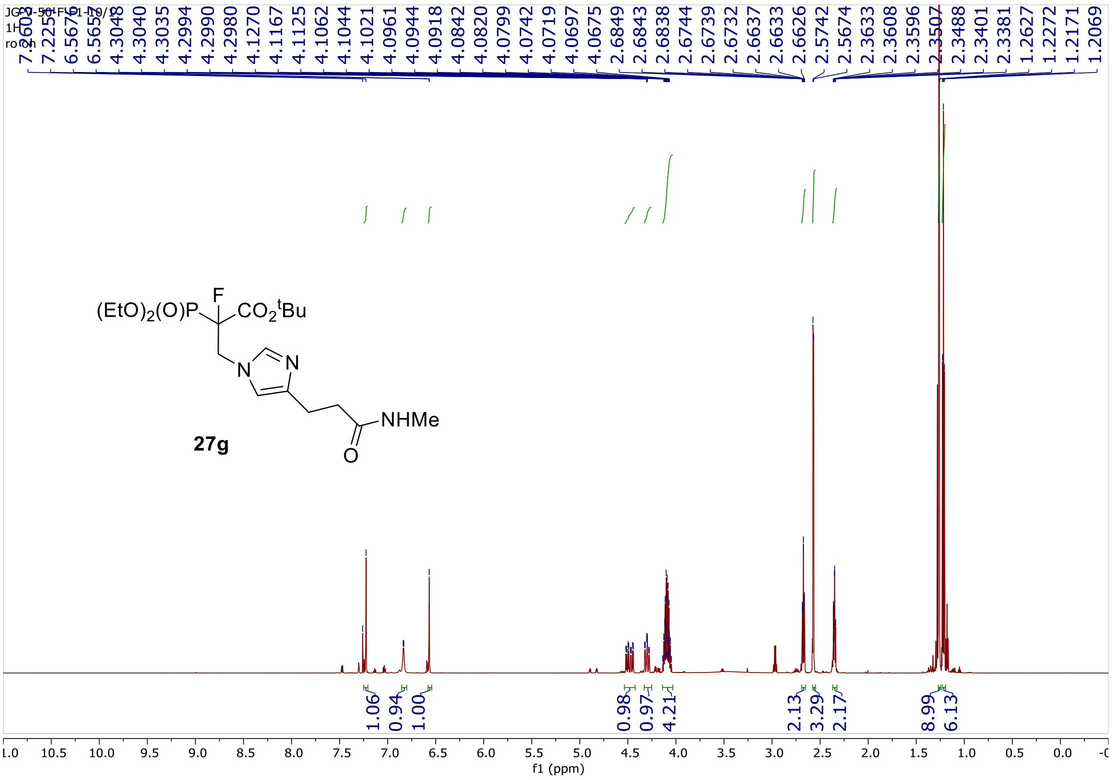

Figure S165. ^{1}H NMR (700 MHz, $CDCl_3$) of compound **27g**.

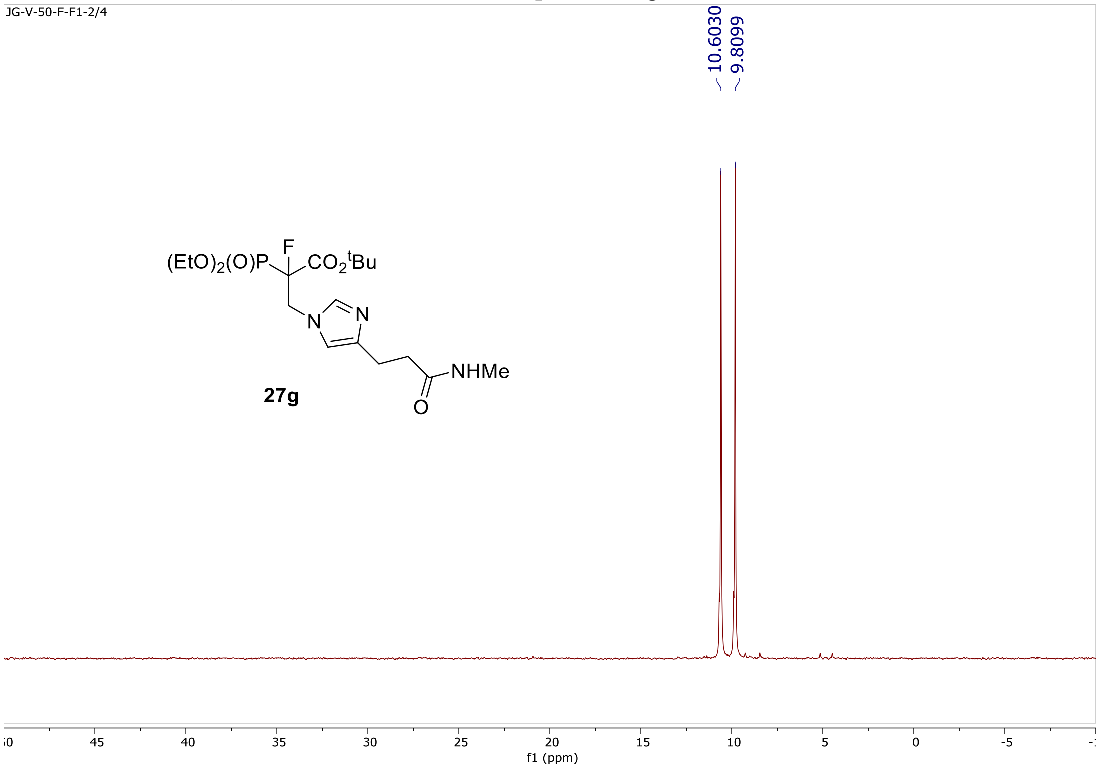

Figure S166. ^{31}P NMR (101 MHz, $CDCl_3$) of compound **27g**.

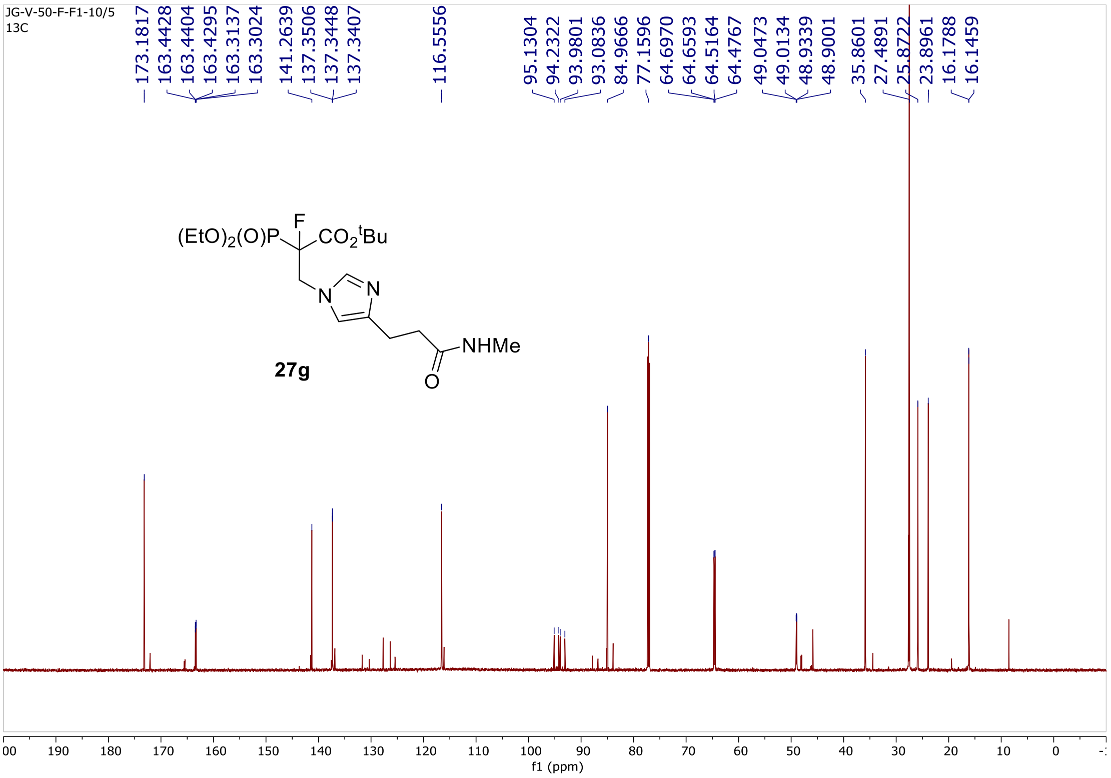

Figure S167. ^{13}C NMR (176 MHz, $CDCl_3$) of compound **27g**.

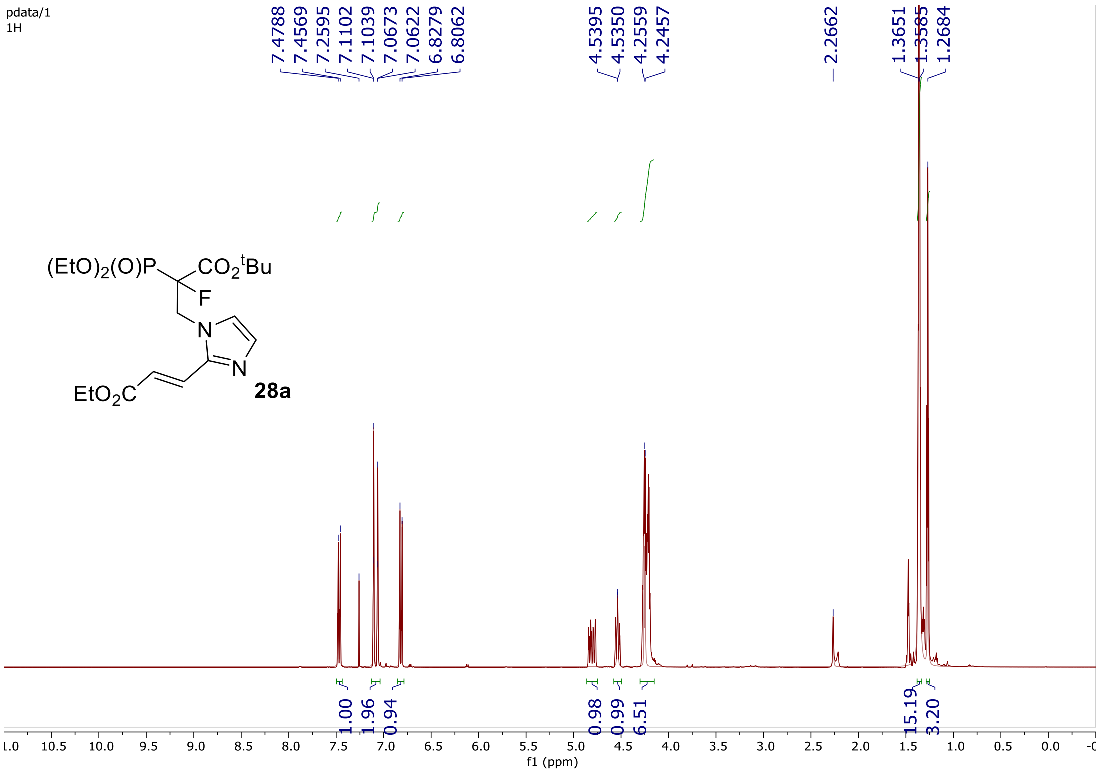

Figure S168. ^{1}H NMR (700 MHz, $CDCl_3$) of compound **28a**.

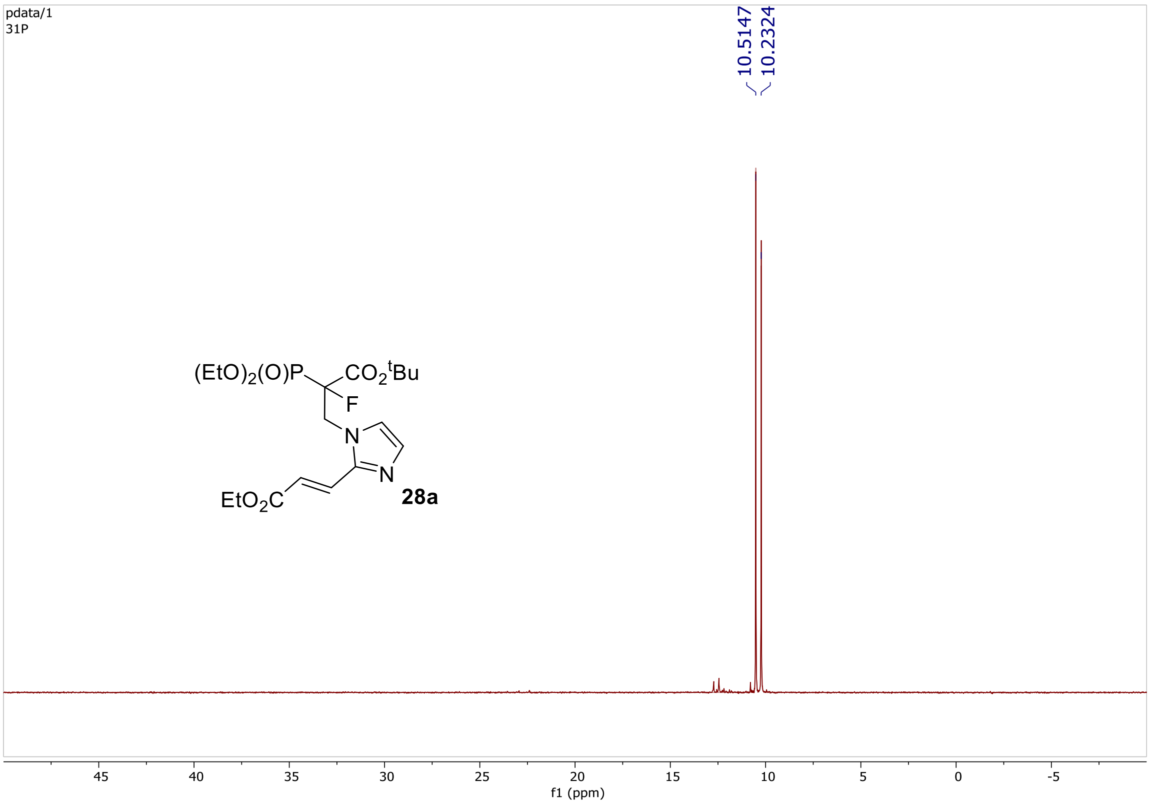

Figure S169. ^{31}P NMR (283 MHz, $CDCl_3$) of compound **28a**.

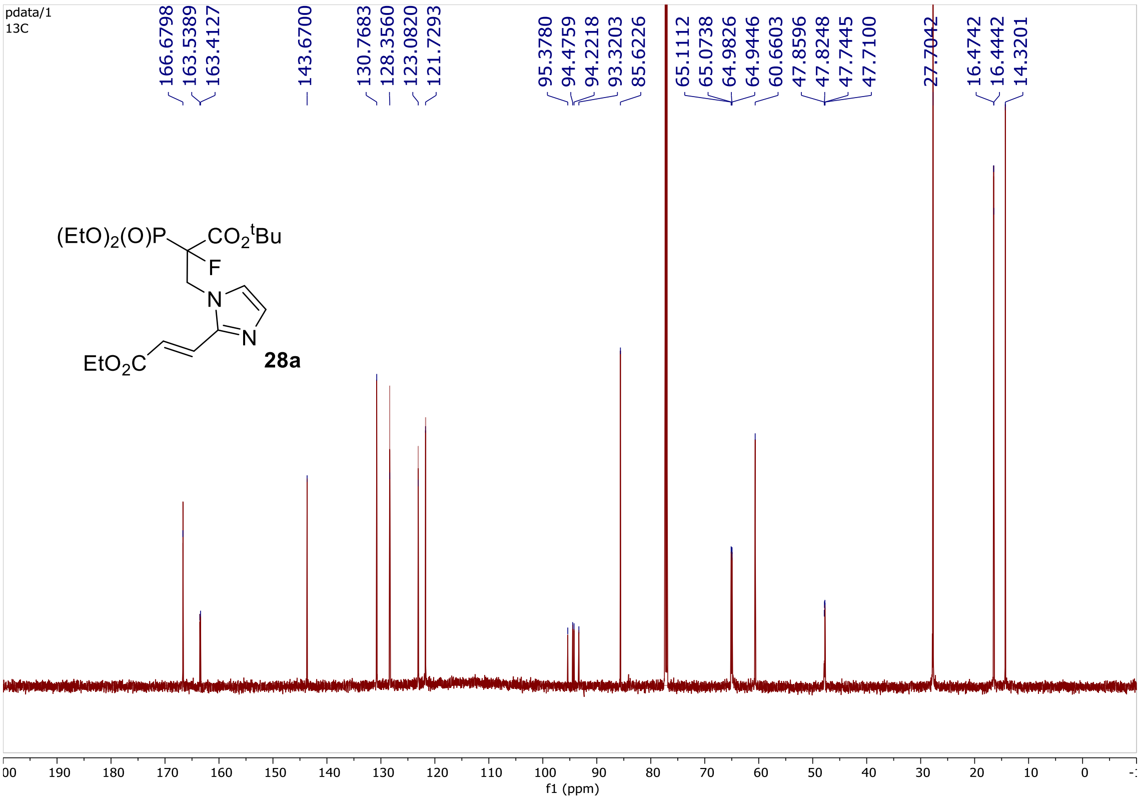

Figure S170. ^{13}C NMR (176 MHz, $CDCl_3$) of compound **28a**.

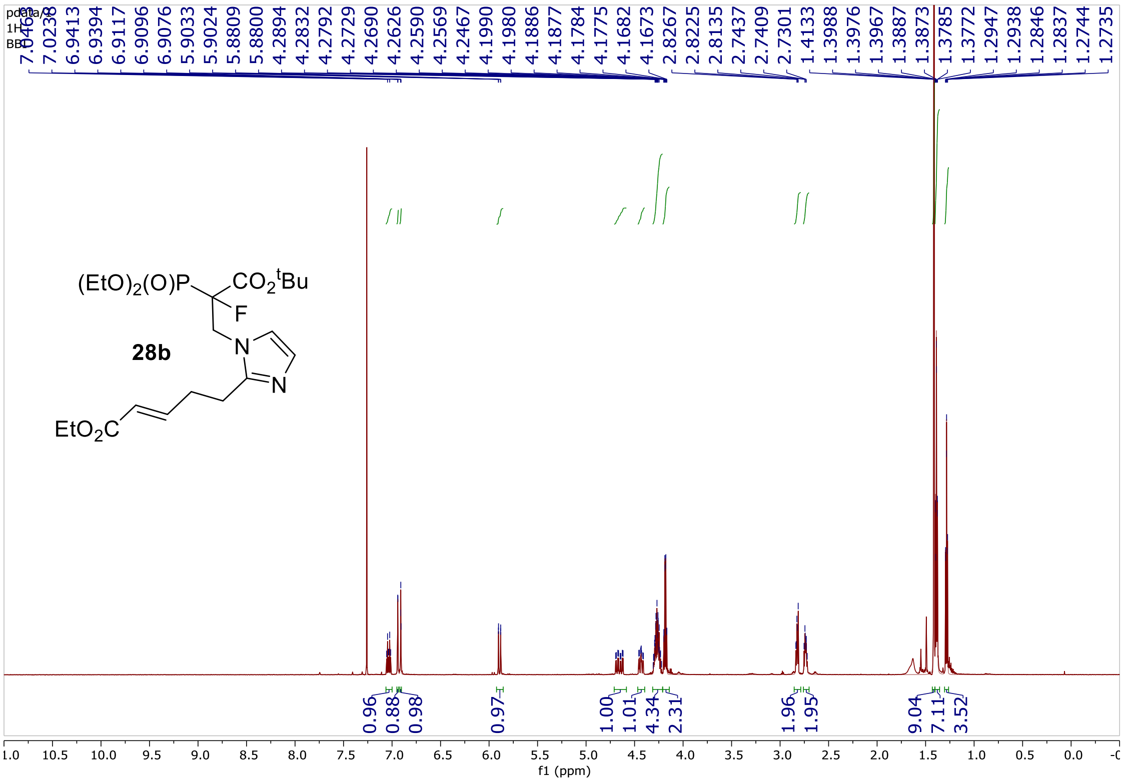

Figure S171. ^{1}H NMR (700 MHz, $CDCl_3$) of compound **28b**.

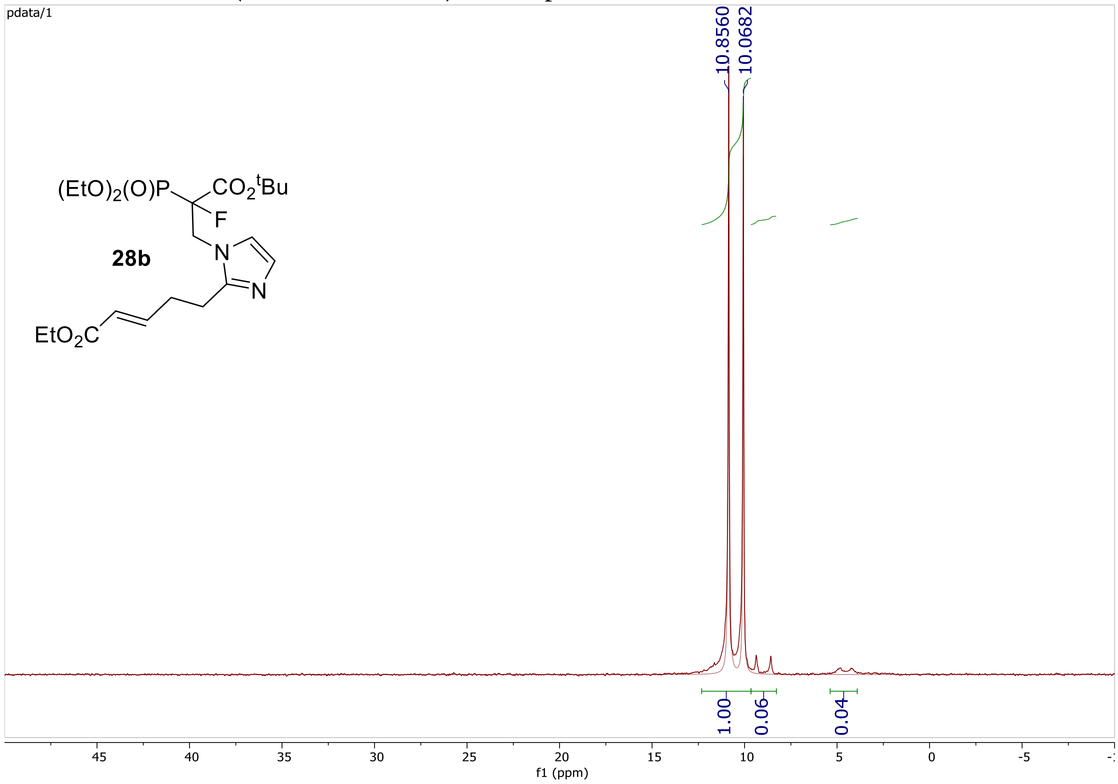

Figure S172. ^{31}P NMR (101 MHz, $CDCl_3$) of compound **28b**.

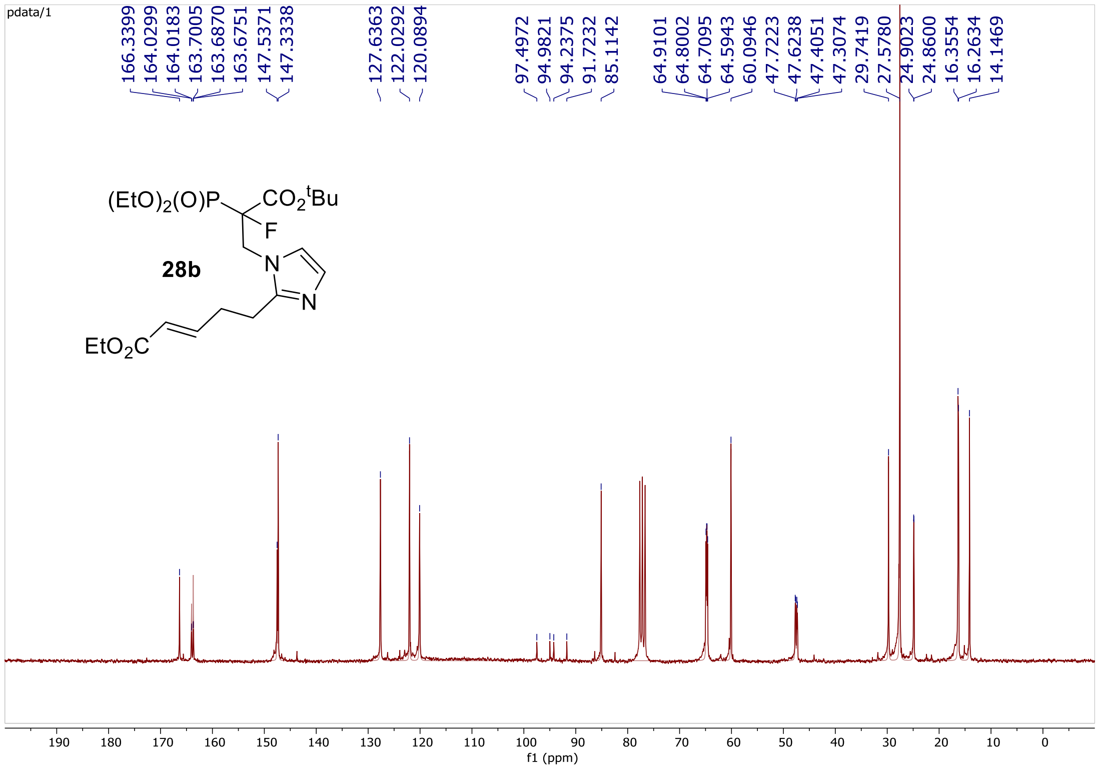

Figure S173. ^{13}C NMR (63 MHz, $CDCl_3$) of compound **28b**.

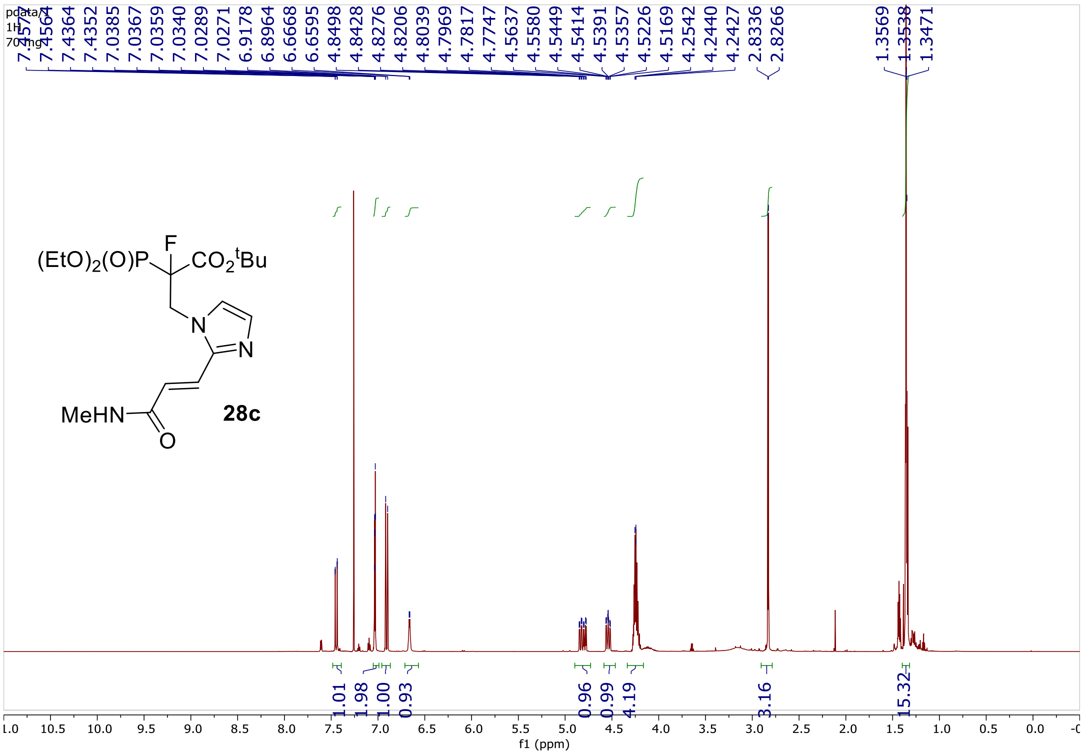

Figure S174. ^{1}H NMR (700 MHz, $CDCl_3$) of compound **28c**.

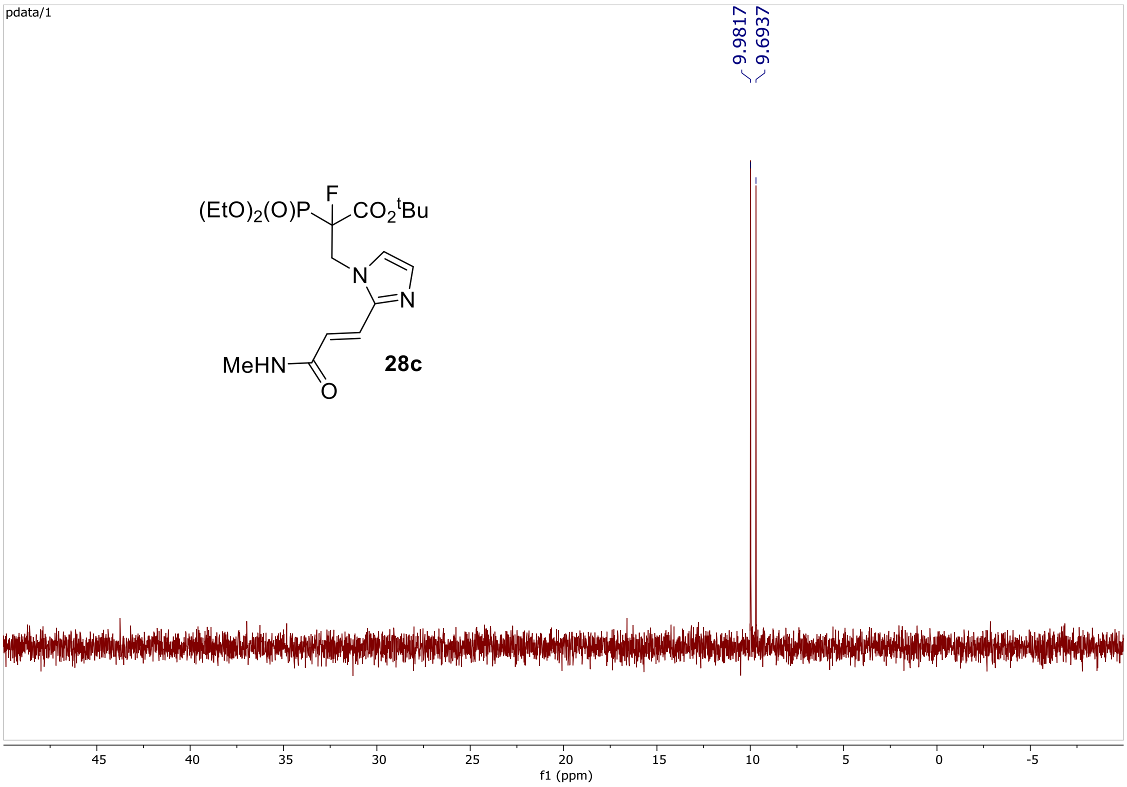

Figure S175. ^{31}P NMR (283 MHz, $CDCl_3$) of compound **28c**.

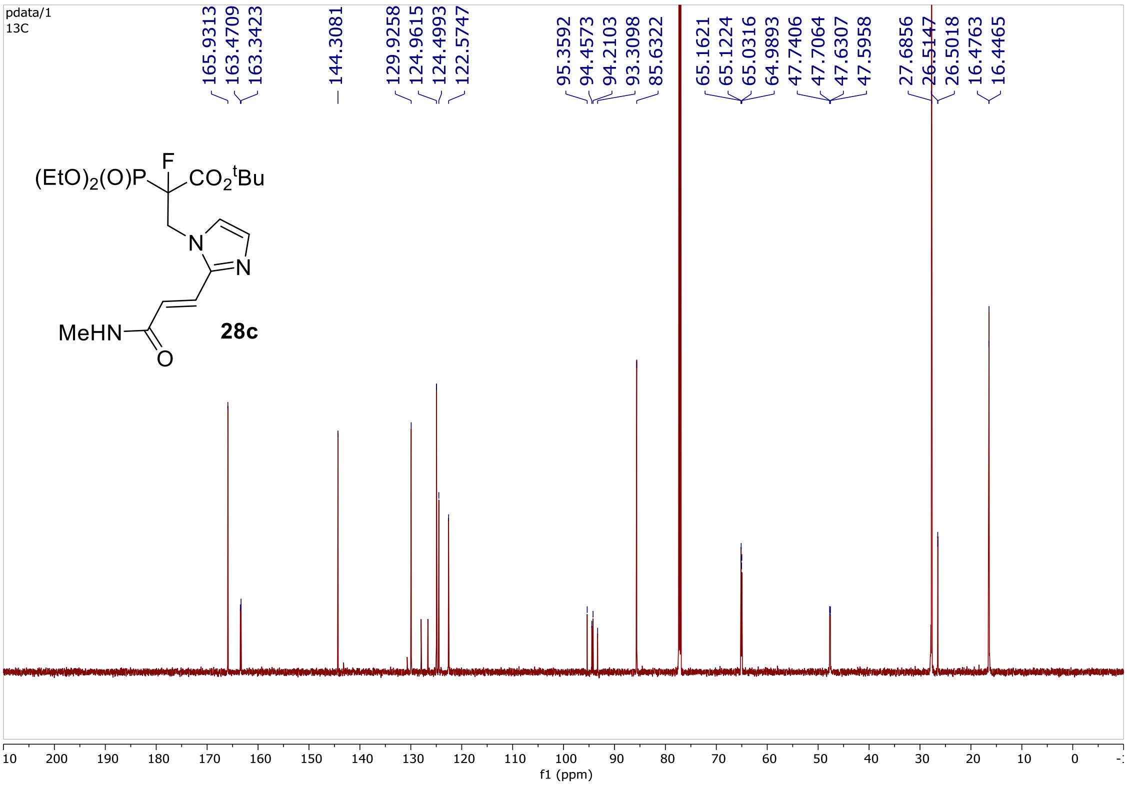

Figure S176. ^{13}C NMR (176 MHz, $CDCl_3$) of compound **28c**.

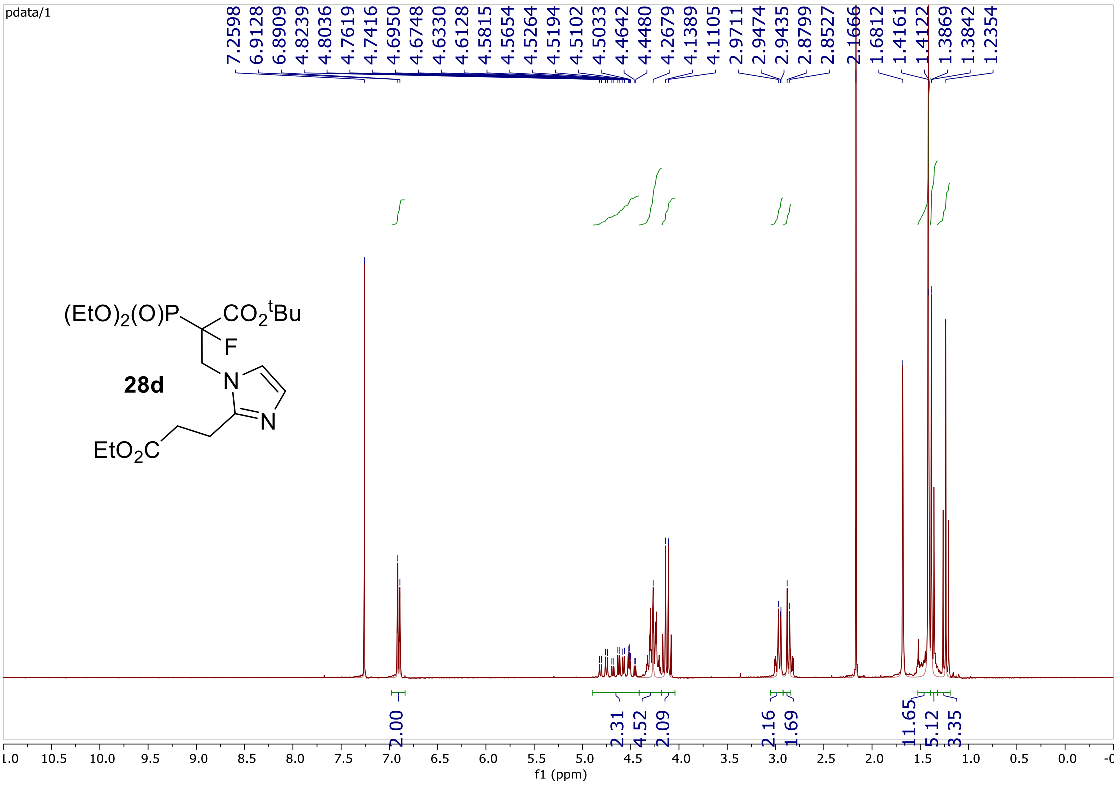

Figure S177. ^{1}H NMR (250 MHz, $CDCl_3$) of compound **28d**.

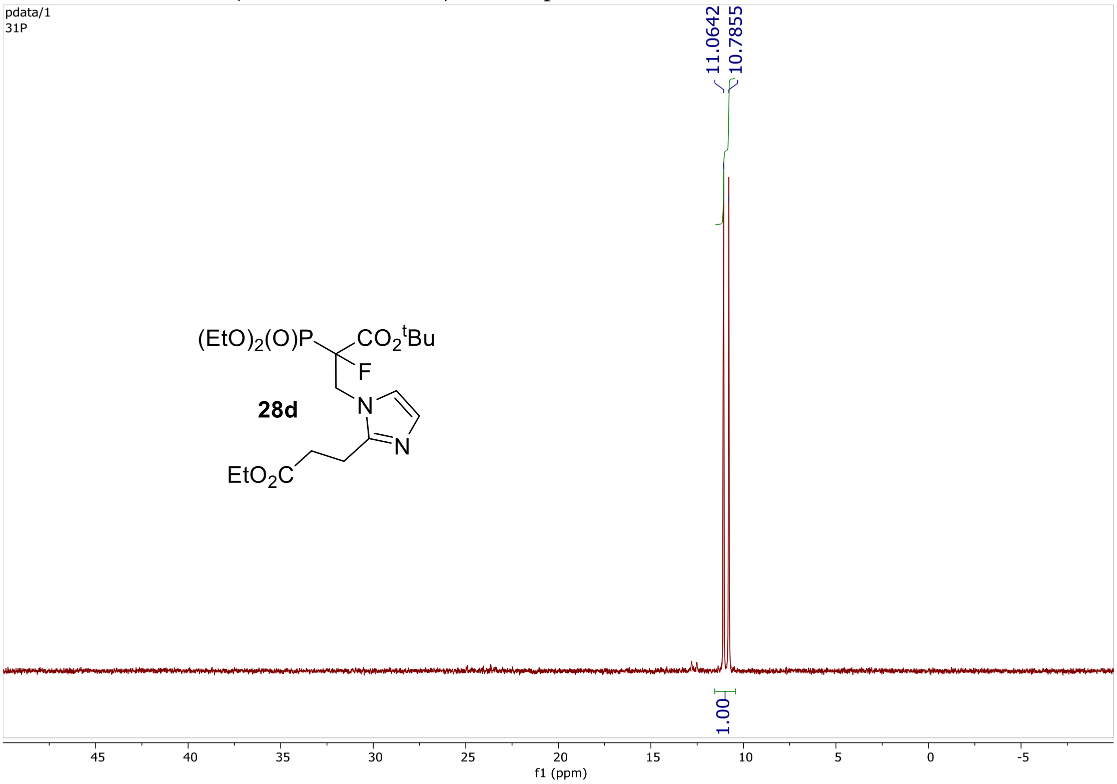

Figure S178. ^{31}P NMR (283 MHz, $CDCl_3$) of compound **28d**.

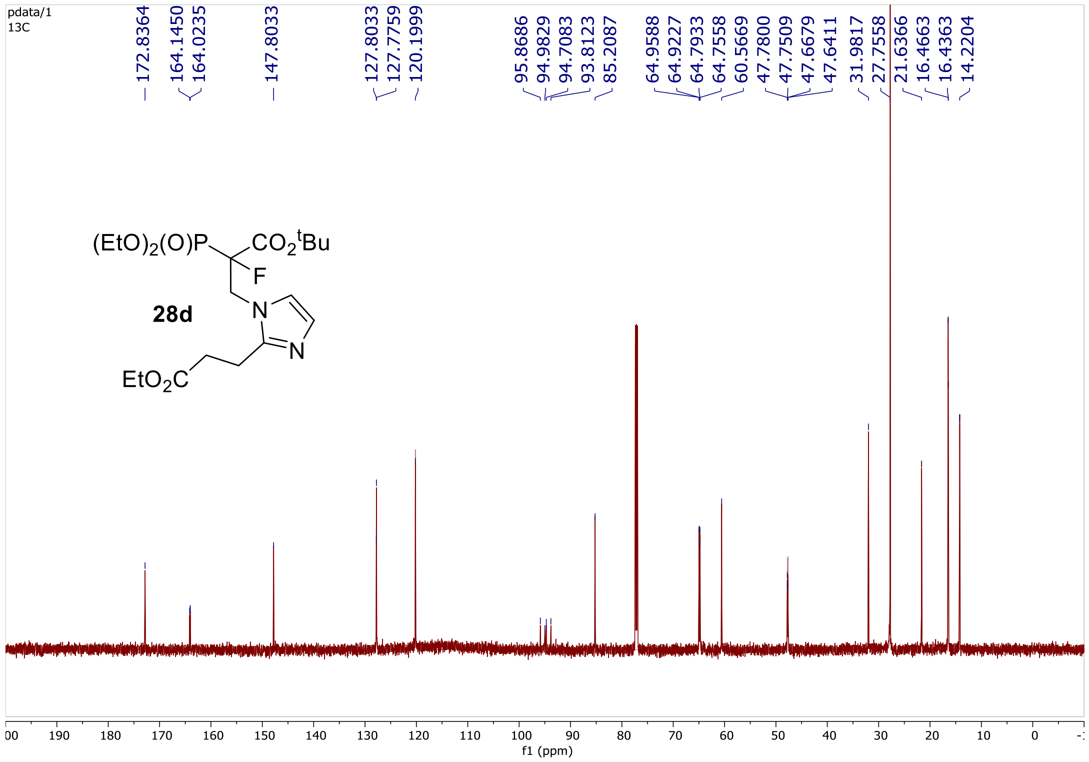

Figure S179. ^{13}C NMR (176 MHz, $CDCl_3$) of compound **28d**.

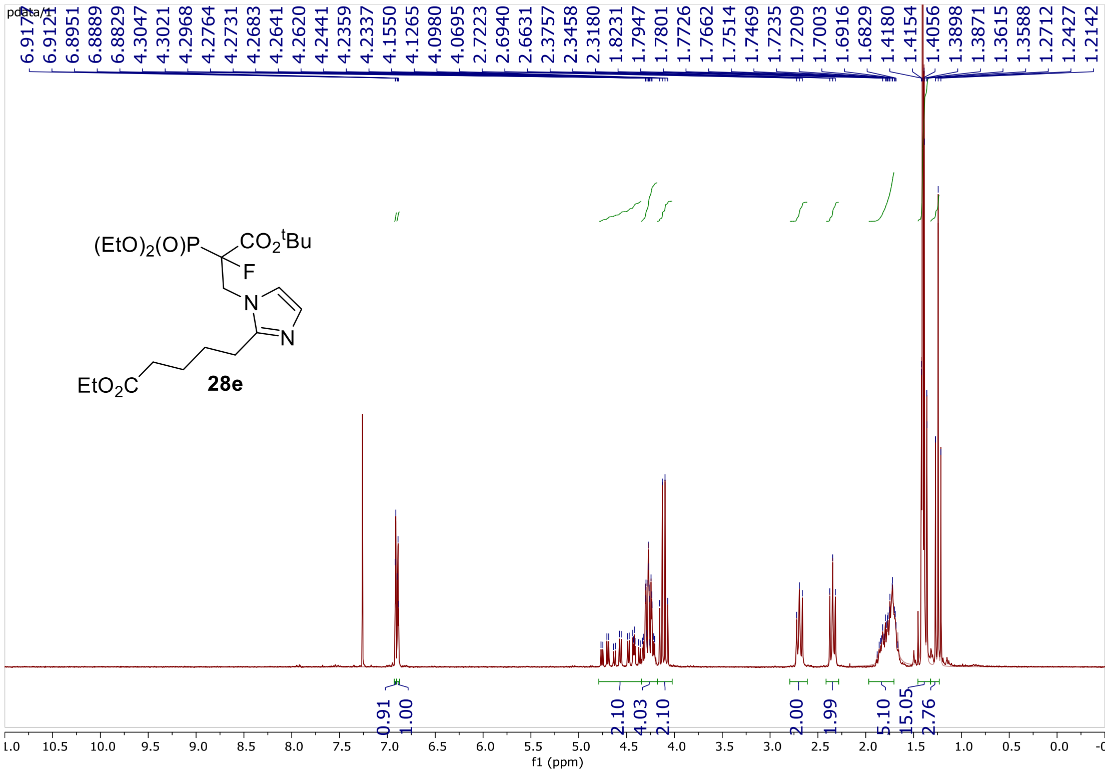

Figure S180. ^{1}H NMR (250 MHz, $CDCl_3$) of compound **28e**.

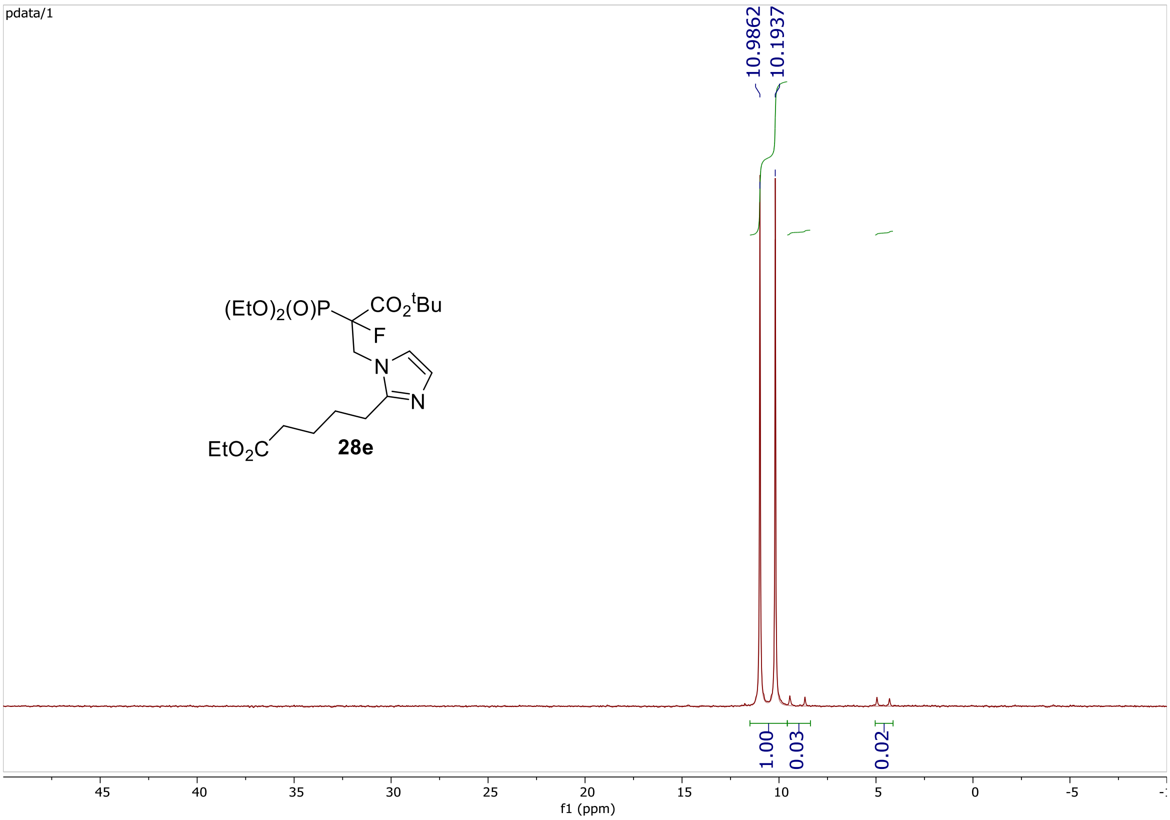

Figure S181. ^{31}P NMR (101 MHz, $CDCl_3$) of compound **28e**.

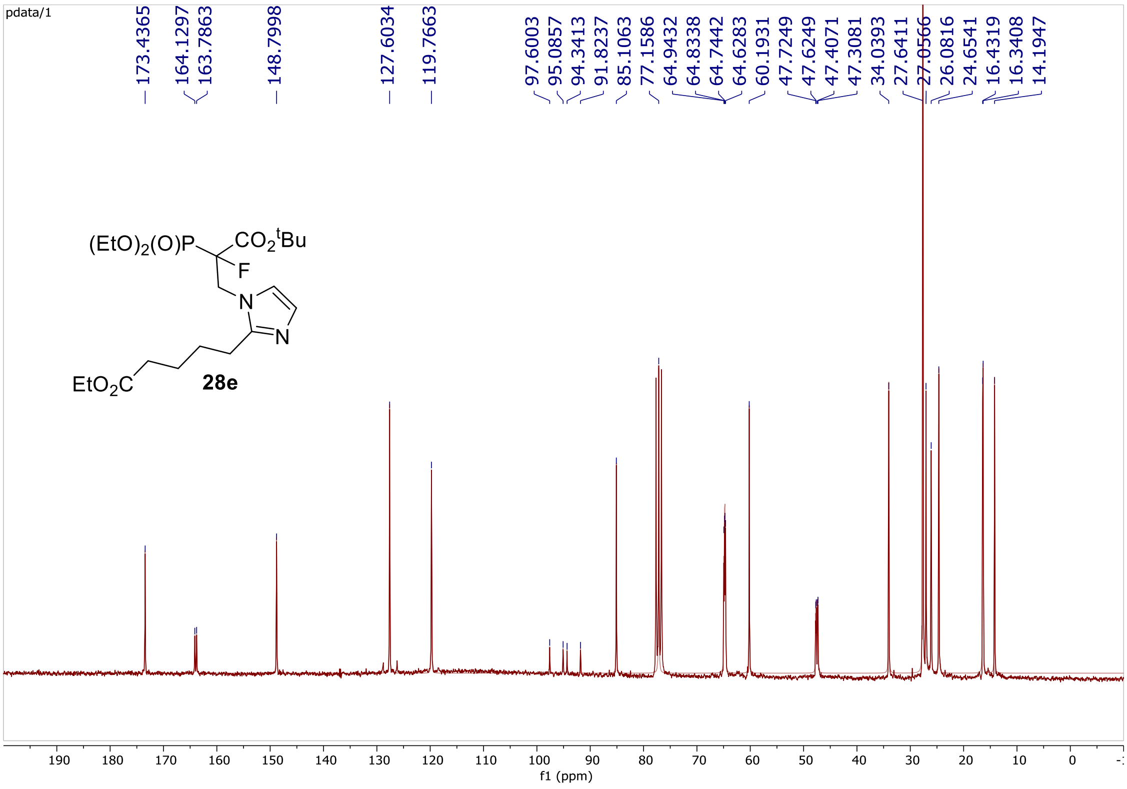

Figure S182. ^{13}C NMR (63 MHz, $CDCl_3$) of compound **28e**.

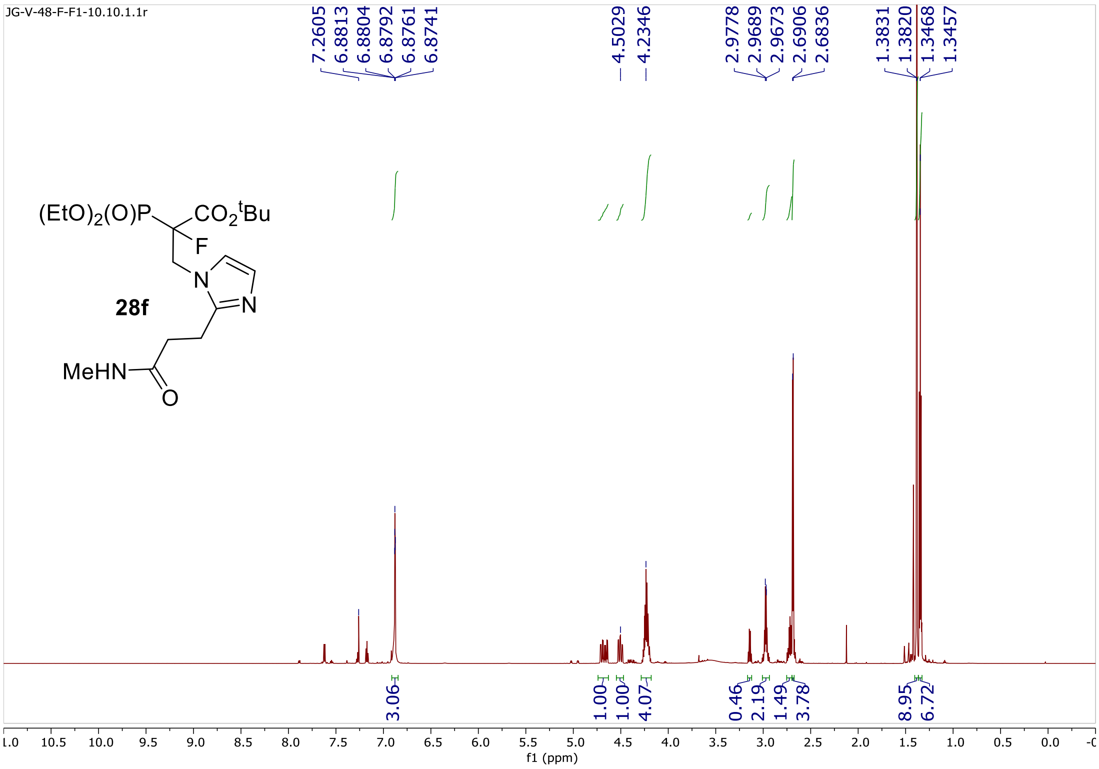

Figure S183. ^{1}H NMR (700 MHz, $CDCl_3$) of compound **28f**.

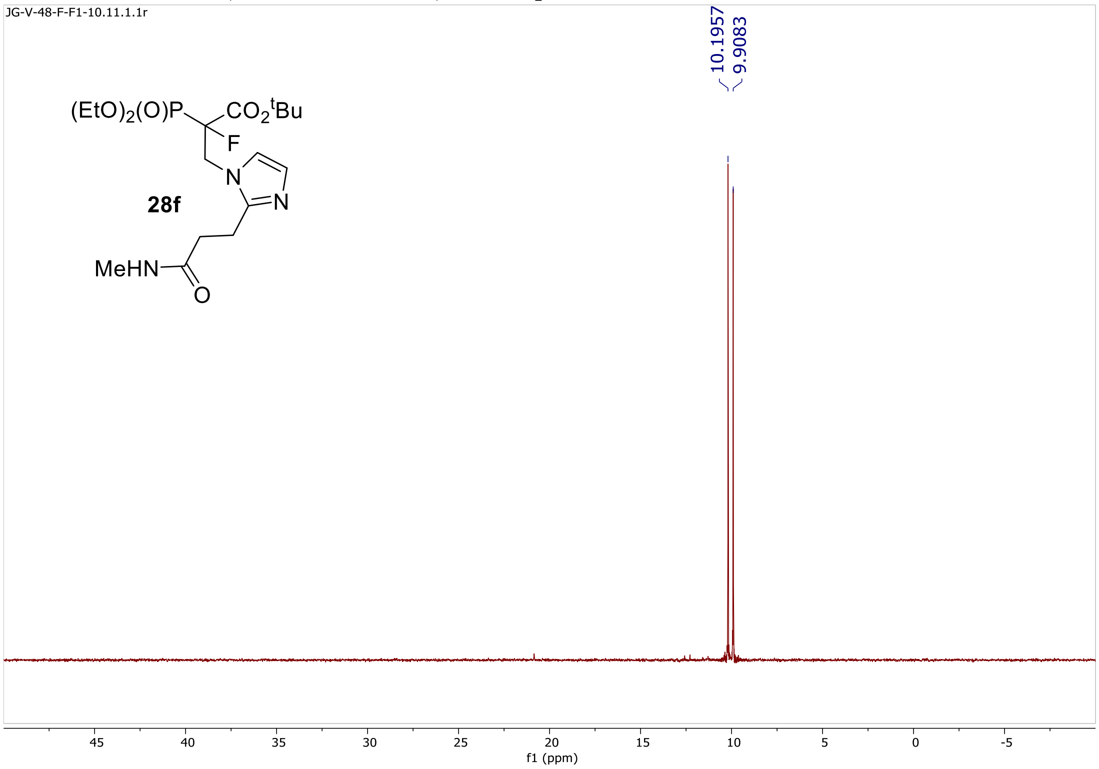

Figure S184. ^{31}P NMR (283 MHz, $CDCl_3$) of compound **28f**.

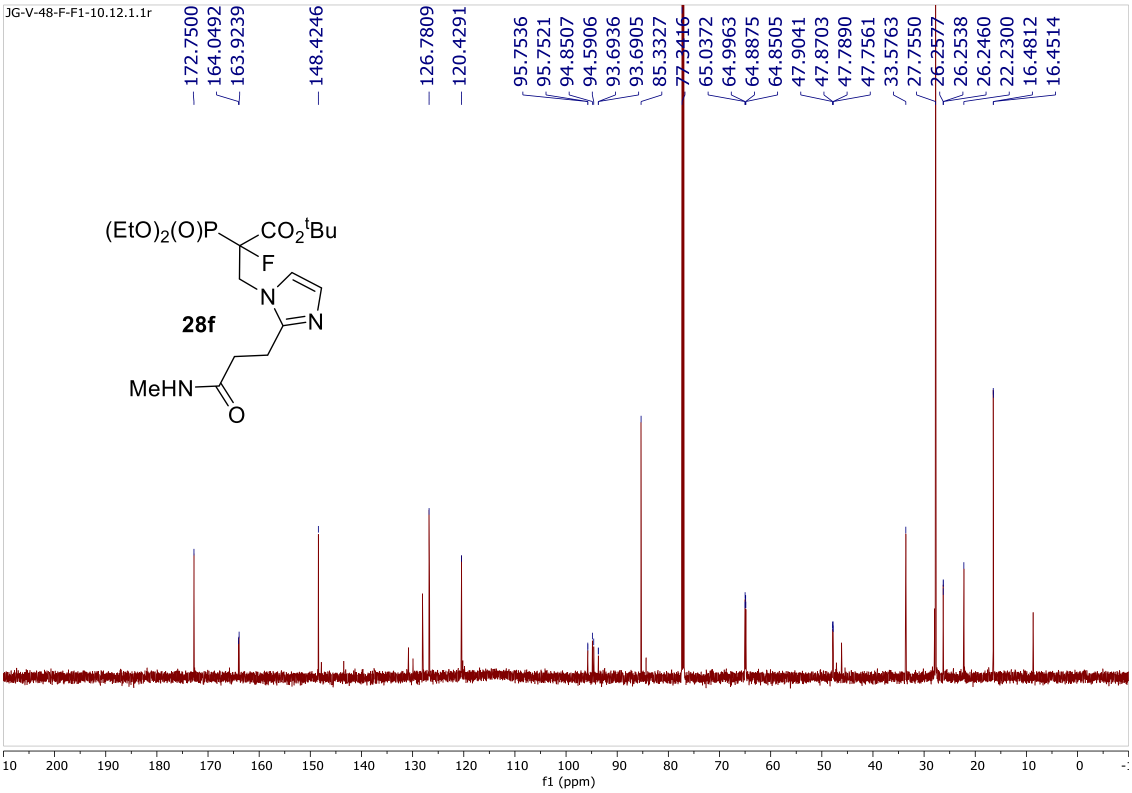

Figure S185. ^{13}C NMR (176 MHz, $CDCl_3$) of compound **28f**.

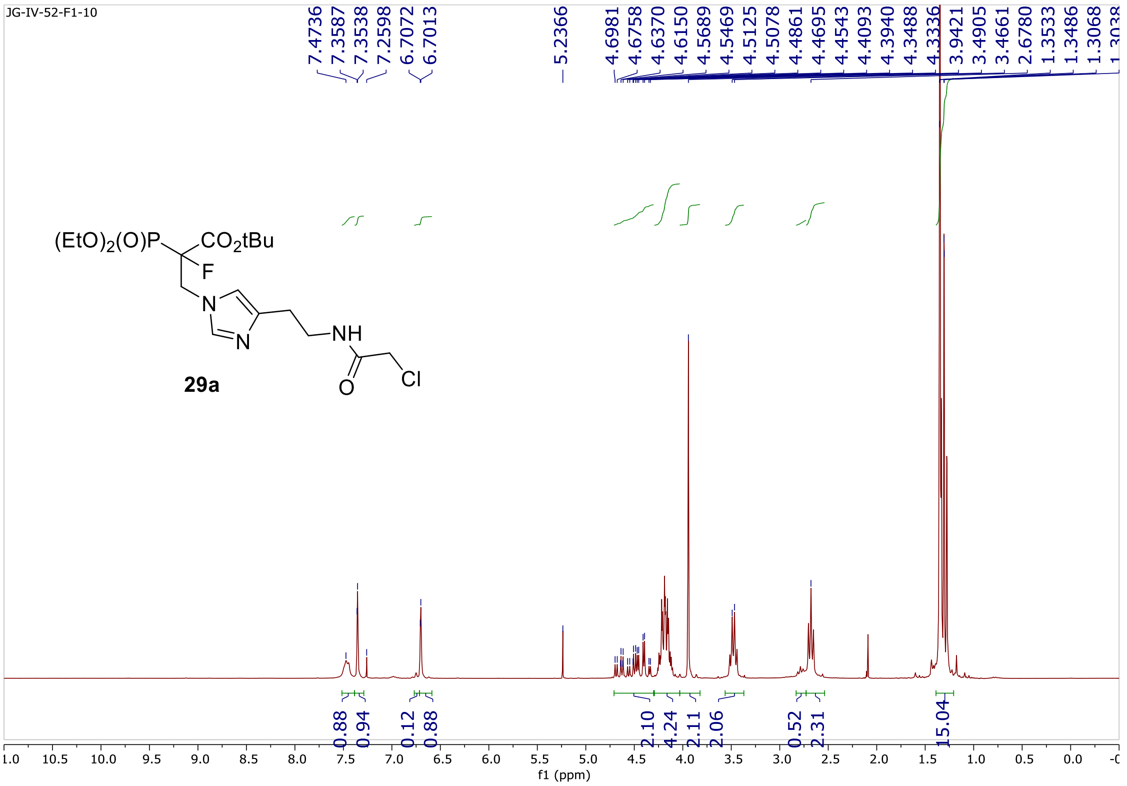

Figure S186. ^{1}H NMR (700 MHz, $CDCl_3$) of compound **29a**.

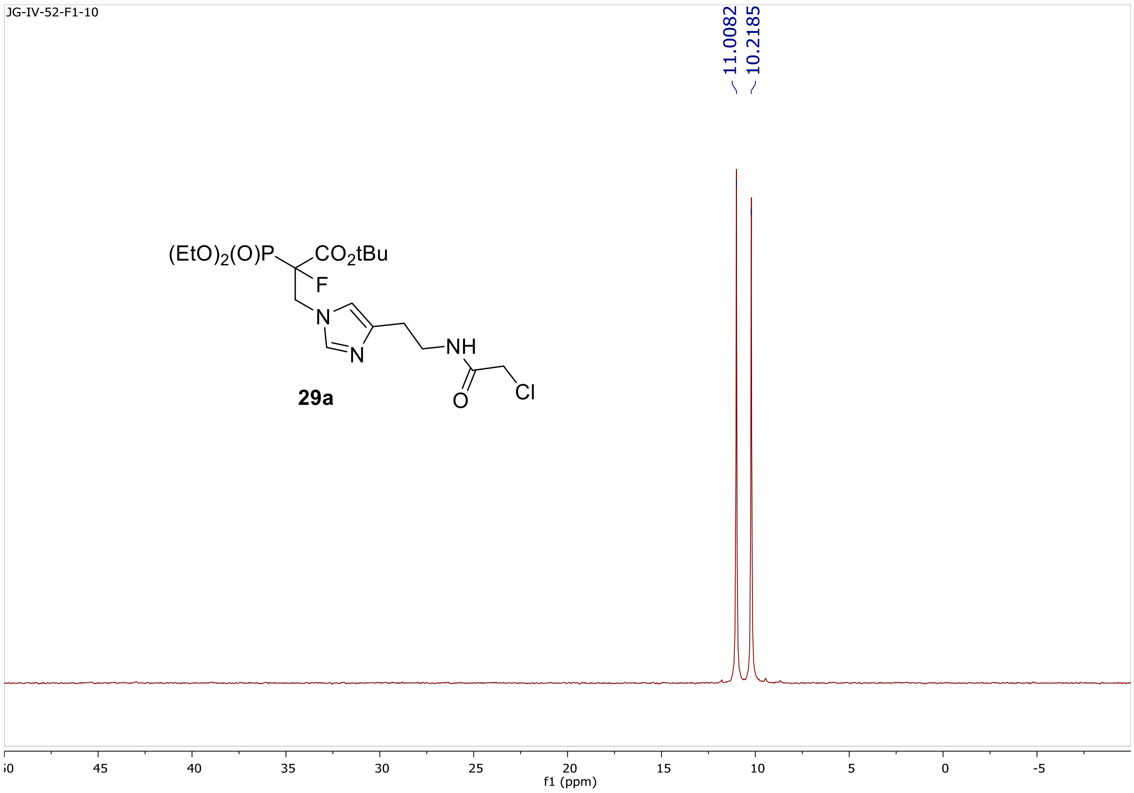

Figure S187. ^{31}P NMR (101 MHz, $CDCl_3$) of compound **29a**.

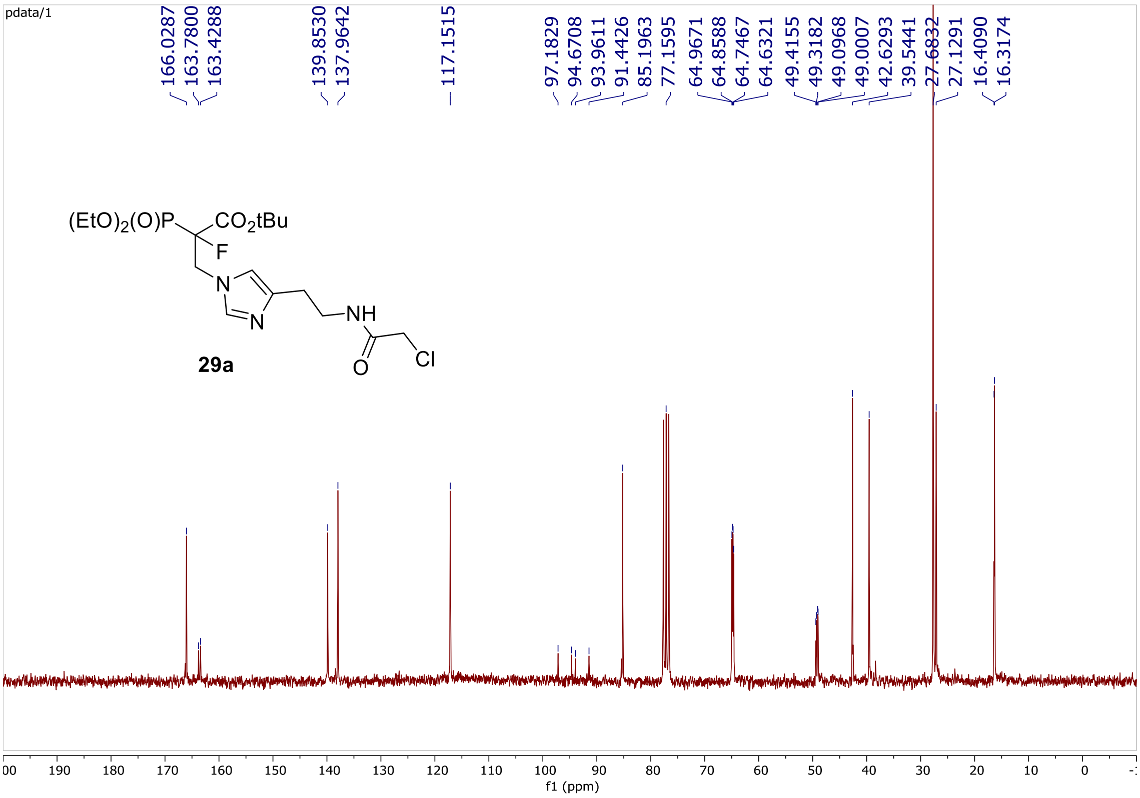

Figure S188. ^{13}C NMR (63 MHz, $CDCl_3$) of compound **29a**.

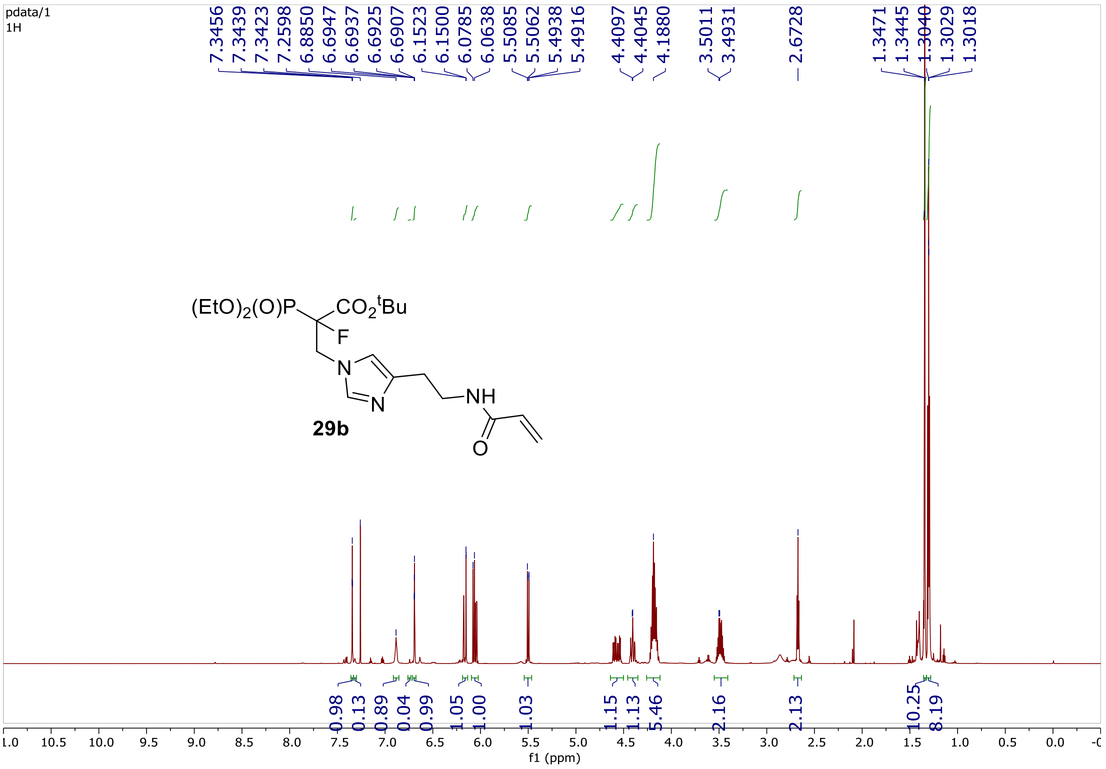

Figure S189. ^{1}H NMR (700 MHz, $CDCl_3$) of compound **29b**.

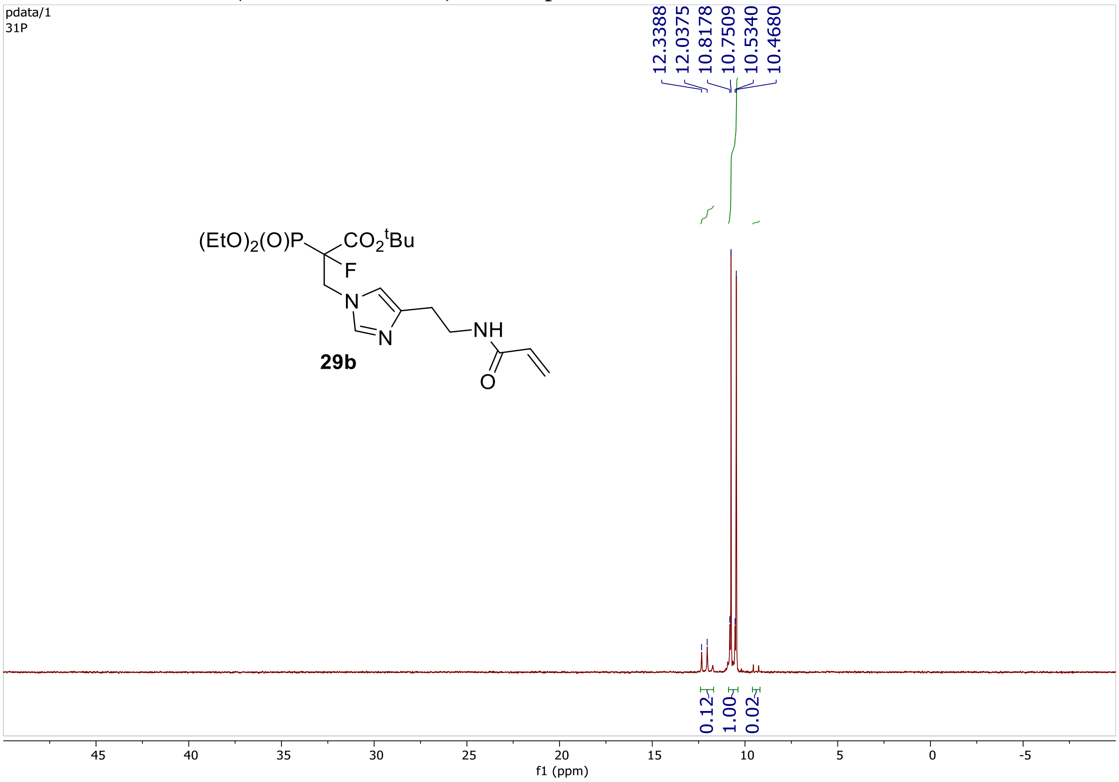

Figure S190. ^{31}P NMR (283 MHz, $CDCl_3$) of compound **29b**.

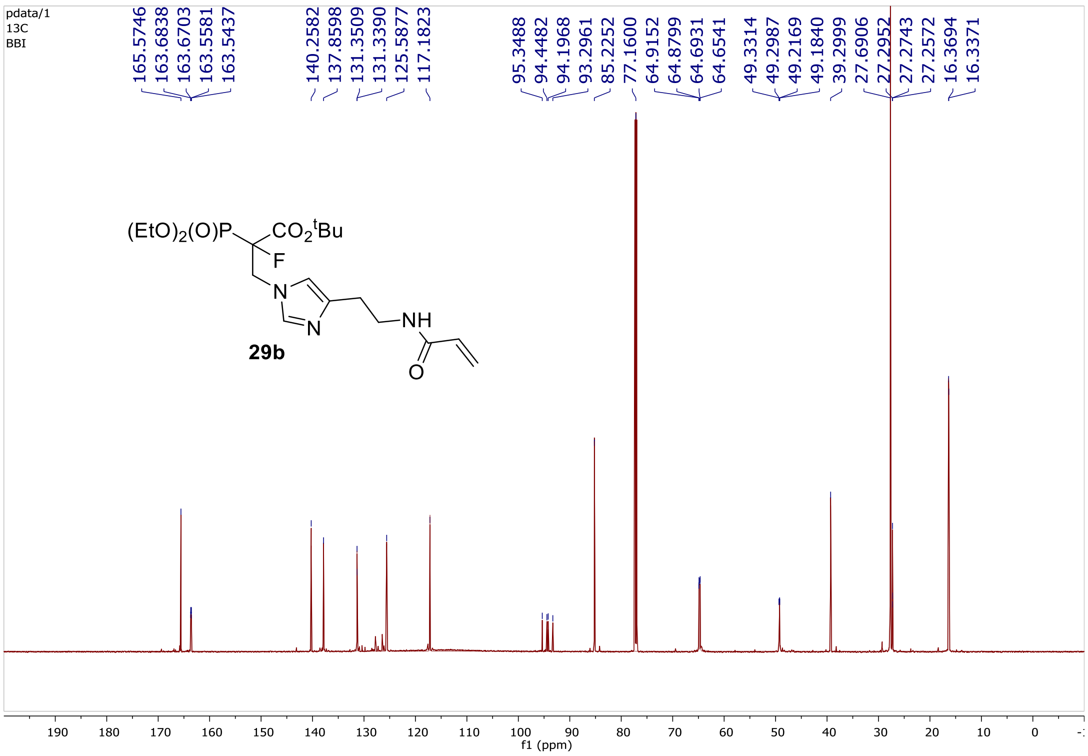

Figure S191. ^{13}C NMR (176 MHz, $CDCl_3$) of compound **29b**.

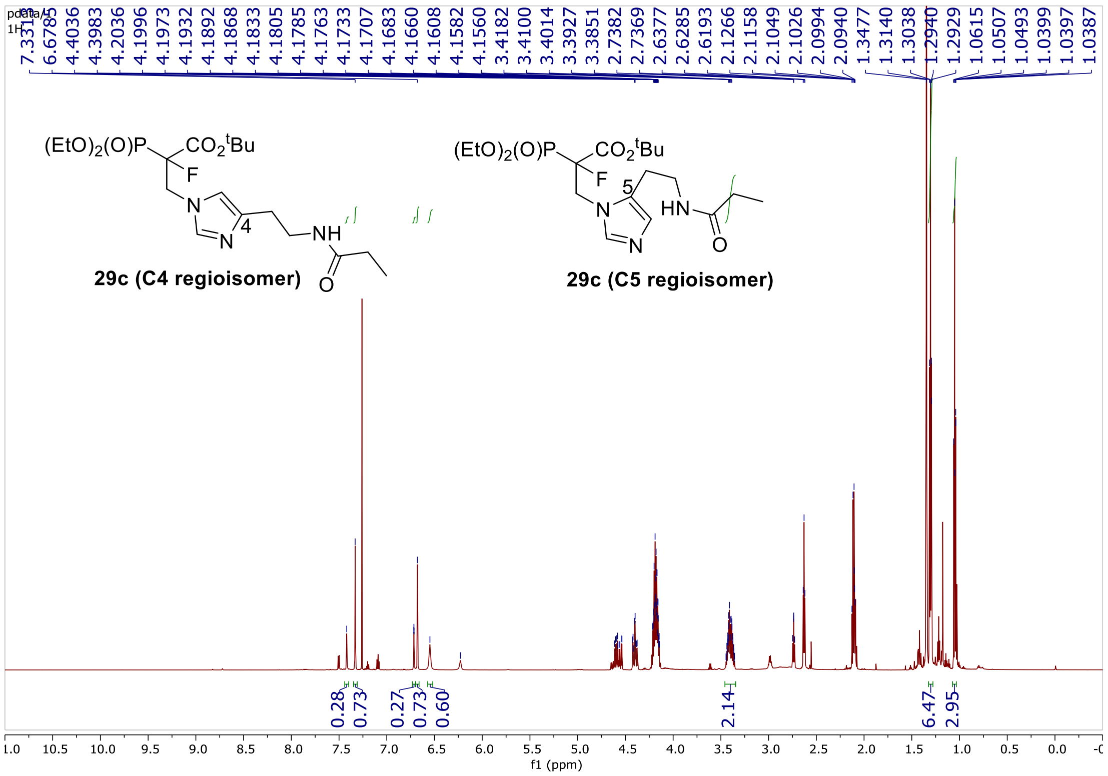

Figure S192. ^{1}H NMR (700 MHz, $CDCl_3$) of compound **29c** (mixture of regioisomers C4 and C5).

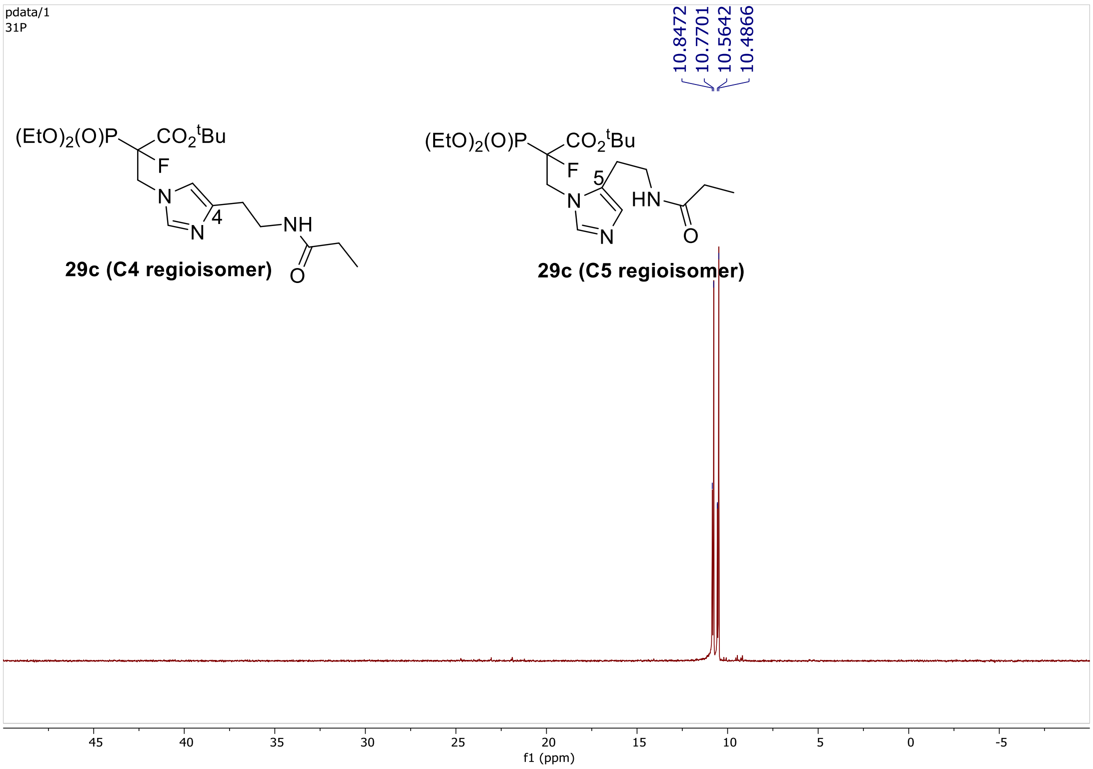

Figure S193. ^{31}P NMR (283 MHz, $CDCl_3$) of compound **29c** (mixture of regioisomers C4 and C5).

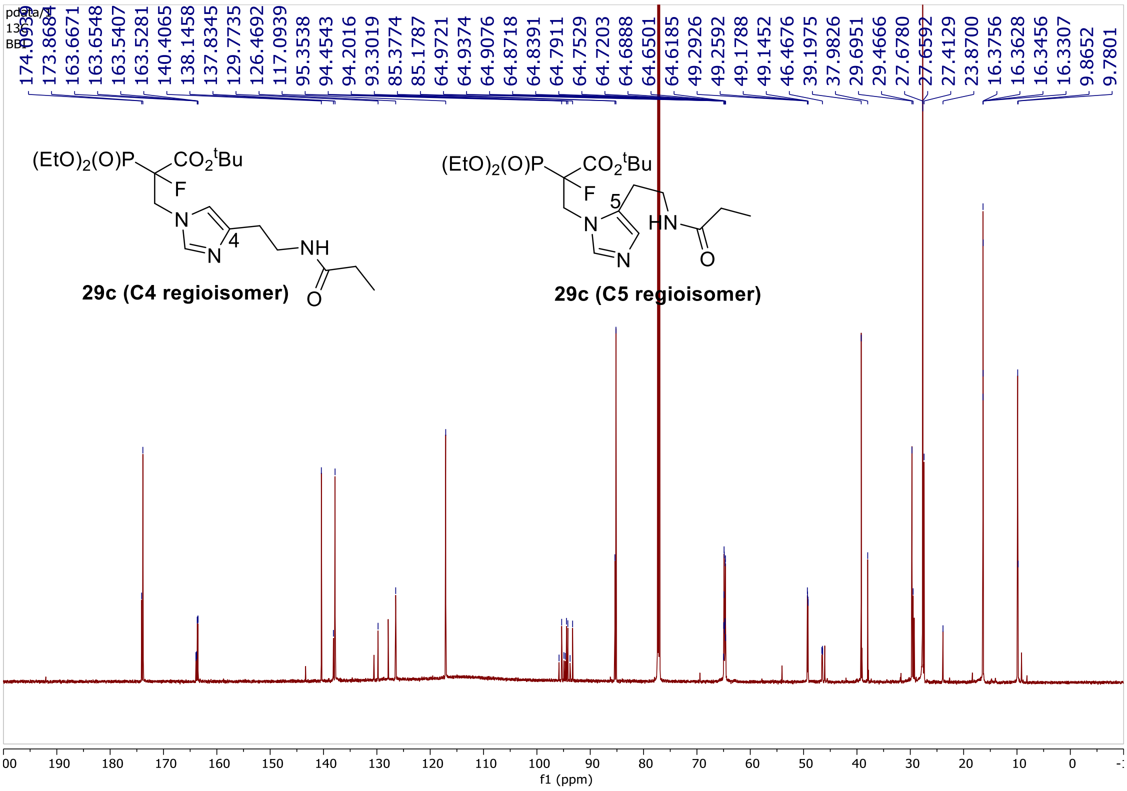

Figure S194. ^{13}C NMR (176 MHz, $CDCl_3$) of compound **29c** (mixture of regioisomers C4 and C5).

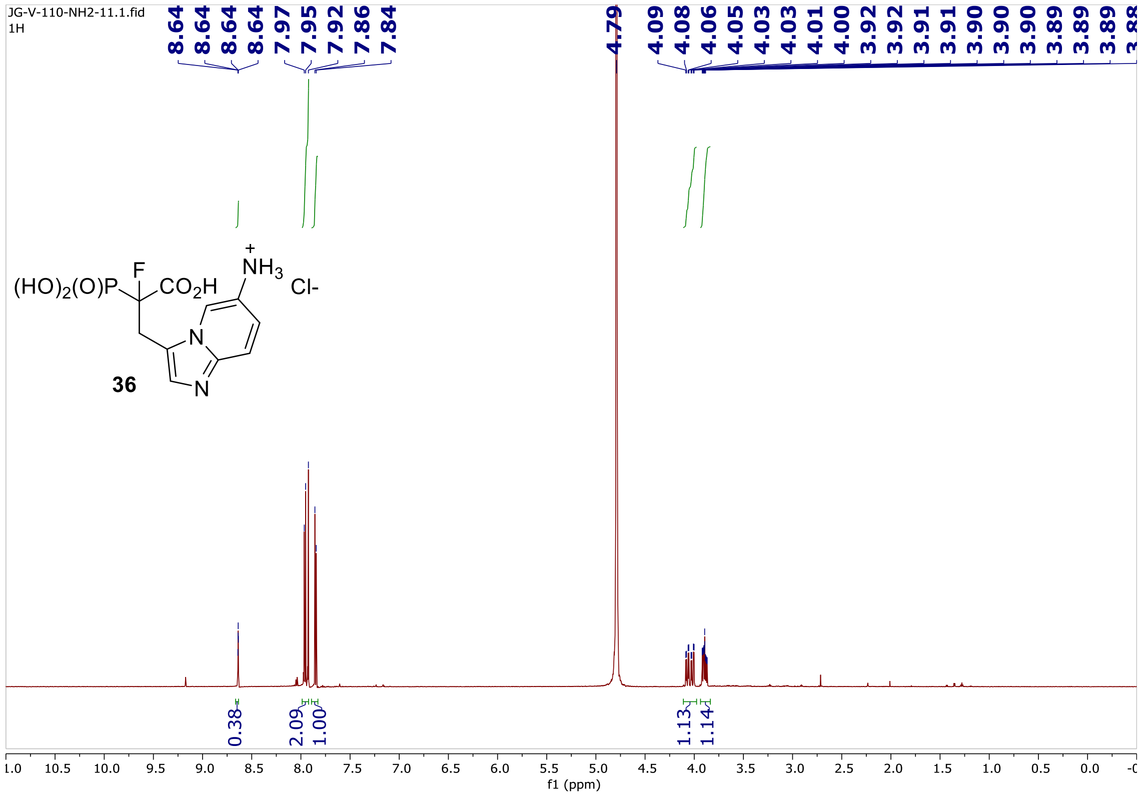

Figure S195. ^{1}H NMR (700 MHz, D_2O, pH 2) of compound **36**.

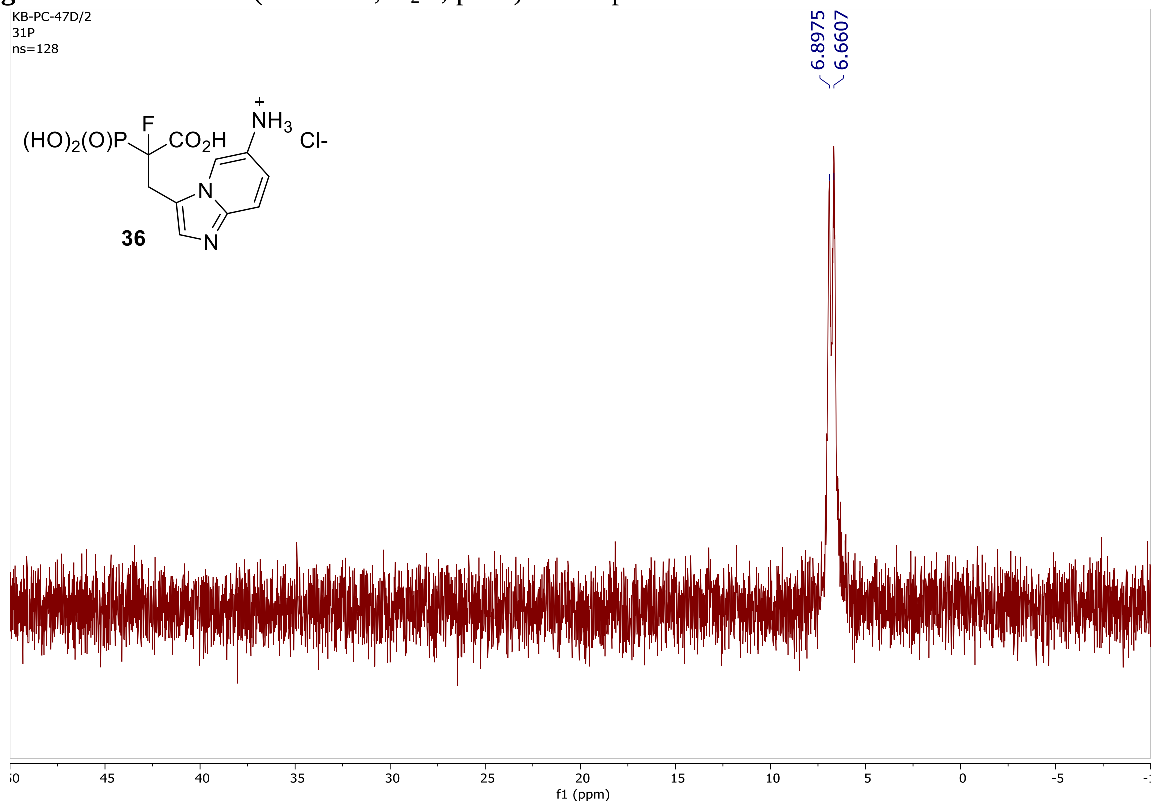

Figure S196. ^{31}P NMR (283 MHz, D_2O, pH 2) of compound **36.**

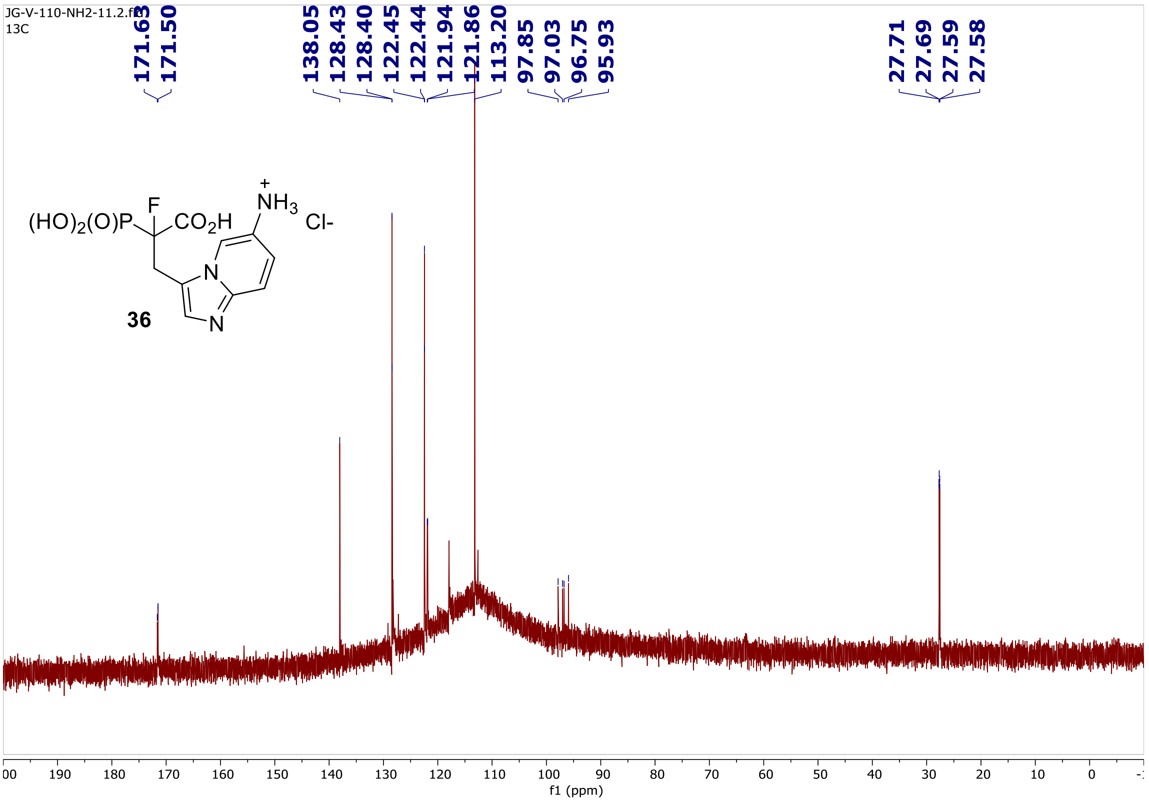

Figure S197. ^{13}C NMR (176 MHz, D_2O, pH 2) of compound **36.**

References

1. Joachimiak Ł, Marchwicka A, Gendaszewska-Darmach E, et al. Synthesis and Biological Evaluation of Imidazole-Bearing α-Phosphonocarboxylates as Inhibitors of Rab Geranylgeranyl Transferase (RGGT). ChemMedChem. 2018;13:842–851.

2. Kusy D, Maniukiewicz W, Błażewska KM. Microwave-assisted synthesis of 3-formyl substituted imidazo[1,2-a]pyridines. Tetrahedron Lett. 2019;151244.

3. Kusy D, Wojciechowska A, Małolepsza J, et al. Functionalization of the imidazo[1,2-a]pyridine ring in α-phosphonoacrylates and α-phosphonopropionates via microwave-assisted Mizoroki-Heck reaction. Beilstein J Org Chem. 2020;16:15–21.

4. Tsou HR, Overbeek-Klumpers EG, Hallett WA, et al. Optimization of 6,7-disubstituted-4-(arylamino)quinoline-3-carbonitriles as orally active, irreversible inhibitors of human epidermal growth factor receptor-2 kinase activity. J Med Chem. 2005;48:1107–1131.

5. Qin M, Wang T, Xu B, et al. Novel hydrazone moiety-bearing aminopyrimidines as selective inhibitors of epidermal growth factor receptor T790M mutant. Eur J Med Chem. 2015;104:115–126.
